# Tailoring Carbene–Metal–Amides for Thermally Activated Delayed Fluorescence: A Computationally Guided Study on the Effect of Cyclic (Alkyl)(amino)carbenes

**DOI:** 10.3390/molecules28114398

**Published:** 2023-05-28

**Authors:** Nguyen Le Phuoc, Alexander C. Brannan, Alexander S. Romanov, Mikko Linnolahti

**Affiliations:** 1 Department of Chemistry, University of Eastern Finland, FI-80101 Joensuu, Finland; nguyen.le.phuoc@uef.fi; 2 Department of Chemistry, University of Manchester, Oxford Rd., Manchester M13 9PL, UK; alexander.brannan@manchester.ac.uk

**Keywords:** carbene–metal–amide, thermally activated delayed fluorescence, cyclic (alkyl)(amino) carbene, photoluminescence, computational

## Abstract

Gold-centered carbene–metal–amides (CMAs) containing cyclic (alkyl)(amino)carbenes (CAACs) are promising emitters for thermally activated delayed fluorescence (TADF). Aiming at the design and optimization of new TADF emitters, we report a density functional theory study of over 60 CMAs with various CAAC ligands, systematically evaluating computed parameters in relation to photoluminescence properties. The CMA structures were primarily selected based on experimental synthesis prospects. We demonstrate that TADF efficiency of the CMA materials originates from a compromise between oscillator strength coefficients and exchange energy (ΔE_ST_). The latter is governed by the overlap of HOMO and LUMO orbitals, where HOMO is localized on the amide and LUMO over the Au–carbene bond. The S_0_ ground and excited T_1_ states of the CMAs adopt approximately coplanar geometry of carbene and amide ligands, but rotate perpendicular in the excited S_1_ states, resulting in degeneracy or near-degeneracy of S_1_ and T_1_, accompanied by a decrease in the S_1_-S_0_ oscillator strength from its maximum at coplanar geometries to near zero at rotated geometries. Based on the computations, promising new TADF emitters are proposed and synthesized. Bright CMA complex (^Et2^CAAC)Au(carbazolide) is obtained and fully characterized in order to demonstrate that excellent stability and high radiative rates up to 10^6^ s^−1^ can be obtained for the gold–CMA complexes with small CAAC–carbene ligands.

## 1. Introduction

Organic light-emitting diodes (OLEDs) are widely employed in display technology due to their low power consumption and high efficiency in light production, along with other desirable properties. The efficiency of OLEDs relies on the selection of appropriate materials, especially for the emitting layer, which converts electrical energy into light. Emitters operating via fluorescence demonstrate a maximum internal quantum efficiency (IQE) of 25% due to exclusive harvesting of the singlet excitons, while 75% of the “dark” triplet states are lost via non-radiative pathways. Phosphorescent emitters are capable of harvesting both singlet and triplet excitons to achieve 100% efficiency due to large spin–orbit coupling, which originates from the heavy metals in the structure of the phosphorescent organometallic compounds [1]. Unlike commercial red and green PhOLED devices, blue PhOLEDs suffer from poor device operating stability, which makes them incompatible with commercial applications. The blue PhOLED problem is associated with chemical bond dissociation or degradation of the blue phosphorescent material [1], which has a typical excited state lifetime of several microseconds. This is long enough for the bimolecular quenching event to occur, resulting in the formation of high energy excitons (>3 eV) and the degradation of the material [1]. Therefore, in order to develop efficient and stable materials for OLEDs, thermally activated delayed fluorescence (TADF) emitters [2] have been extensively studied, as they demonstrate energy efficiency similar to the phosphorescent emitters in OLED devices [3]. The molecular design for TADF emitters is diverse, holding great promise for the realization of deep blue, energy efficient, and stable OLEDs by designing TADF materials with submicrosecond excited state lifetimes [4].

Carbene–metal–amides (CMAs) [5,6,7,8,9,10,11,12,13,14,15,16,17,18,19,20] have emerged as a promising class of TADF emitters, thanks to their exceptional photoluminescence (PL) behaviors. These complexes are composed of carbene and amide ligands bridged by a coinage metal, such that the carbene acts as an electron acceptor and the amide as an electron donor. The two-coordinate metal center mediates the electron transfer between the donor and acceptor moieties, while inducing rotational flexibility [5]. The TADF mechanism manifests in CMAs when the singlet excitons decay radiatively with prompt fluorescence and transform into triplet excitons via an intersystem crossing (ISC) process. Simultaneously, low-energy triplet excitons undergo a fast reverse ISC (RISC) to the emissive singlet level, followed by a delayed fluorescence to the ground (S_0_) state. The efficiency of TADF emitters depends on the energy difference between the lowest excited singlet (S_1_) and triplet (T_1_) states, referred to as the exchange energy or singlet–triplet energy gap (ΔE_ST_) [21].

**Figure 1:**
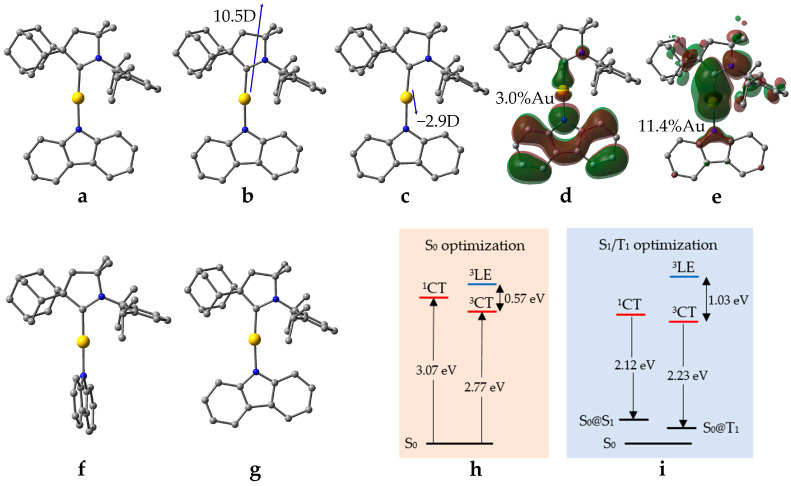
Computed structures and properties of CMA1: (**a**) optimized S_0_ ground state; (**b**) dipole moments for S_0_ and (**c**) S_1_@S_0_ geometry; (**d**) the highest occupied molecular (HOMO) and (**e**) lowest unoccupied molecular (LUMO) orbitals; (**f**) optimized S_1_; (**g**) optimized T_1_; (**h**) vertical excitation energies indicated by the upward arrows; (**i**) energy levels at optimized S_1_ and T_1_ geometries with fluorescence (**left**) and phosphorescence (**right**) indicated by the downward arrows.

Cyclic (alkyl)(amino)carbenes (CAACs) [22] have recently attracted attention as effective π-acceptor ligands in the design of CMA materials [5,6,7,8,9,10,11,12,13,14,15,16]. The most prominent complex, CMA1 (Figure 1), contains a gold atom as a bridge between a carbazolide ligand and a five-membered CAAC. CMA1 demonstrates a considerably short excited state lifetime of 0.95 µs at ambient temperature with near unity photoluminescence quantum yields (PLQY). The CMA1 OLED emits green light with an external quantum efficiency surpassing 25% [6]. Inspired by CMA1, similar coinage metal complexes have been recently reported [5,7,8,9,10,11,12,13,14,15,16], including CMA-based emitters with a CAAC substituted by groups such as methyl, ethyl (Et), menthyl, adamantyl (Ad), and 2,6-diisopropylphenyl (Dipp) [7,8,9]. For example, the menthyl-CAAC–copper complex exhibits broad emission with near unity quantum efficiency of 100% and a 2.5 µs lifetime, resulting in an efficient blue-emitting OLED device [7], whereas the silver analogue exhibits a significantly shorter emission lifetime (0.33 µs) [8]. Furthermore, CMAs of Cu, Ag, and Au metals based on 6-membered monocyclic and bicyclic CAACs have been reported to manifest quantum yields of up to 100%. Among these, a gold complex contains a bicyclic carbene ligand displays remarkable photostability when subjected to hard and soft UV light [10].

This paper reports a systematic computational study of the effect of CAACs on the PL properties of CMAs. We focus on the design and optimization of new TADF emitters based on carbene–gold–carbazolide complexes, employing density functional theory (DFT) calculations in order to explore the electronic structures of the ground and excited states of the molecules as a function of the structure of CAACs. We describe how the structural modifications of the CAACs affect the calculated parameters that directly relate to PL quantum yield (PLQY), thereby ultimately making proposals for new promising TADF emitters within the CMA family, as well as synthesizing one.

## 2. Results and Discussion

We begin from CMA1 (Figure 1), the archetypical emitter in the CMA family, which exhibits high PLQY (80–90%) [6]. This molecule has been thoroughly studied elsewhere, both experimentally and computationally [6,23,24,25,26]. We consider CMA1 as our reference structure, summarizing the relevant parameters for evaluation of its PL behavior, which are recomputed here for this purpose using the MN15 [27]/def2-TZVP [28,29] method, with Tamm–Dancoff approximation [30] for time-dependent DFT (TD-DFT) calculations.

The HOMO and LUMO orbitals of CMA1 are visualized in Figure 1, including the percentages of calculated Au contributions to the frontier orbitals, which illustrate localization of HOMO on carbazole (Cz) and LUMO on Au–carbene. These two orbitals mainly contribute to the charge transfer (CT) between the S_0_ state and the excited S_1_ and T_1_ states, as indicated by the Natural Transition Orbitals (NTOs) of the S_0_-S_1_ and S_0_-T_1_ excitations having HOMO-LUMO characters of 97% and 95%, for S_0_-S_1_ and S_0_-T_1_ transfer, respectively.

An overlap integral of the HOMO and LUMO orbitals provides a quantitative measure of the spatial separation of the orbitals participating in CT, which is connected to the ΔE_ST_, such that a lower overlap integral indicates a smaller gap [31]. For CMA1, the HOMO and LUMO overlap integral is calculated as 0.36, which appears to be low enough for an efficient TADF mechanism [32], as is observed in the experiments. Vertical excitation energies on optimized S_0_ geometry are calculated as 3.07 eV for S_1_ and 2.77 eV for T_1_, thus obtaining ΔE_ST_ = 0.30 eV. The calculations are consistent with broad peaks in the steady-state absorption spectra: namely, at 390 nm (3.15 eV) for amorphous or crystallized CMA1 thin film, and 405 nm (3.06 eV) for CMA1 in toluene solution [26].

The localized triplet excitation (^3^LE) within the Cz ligand, ^3^LE(Cz), corresponds to the T_2_ state, with 92% HOMO-LUMO+3 character, and is calculated as having vertical excitation energy of 3.34 eV—in agreement with the experimental peak at 370 nm (3.35 eV) [26]. The energy of the ^3^LE state relative to the CT states is of major importance, as it contributes to the emission mechanism. For the sake of efficient TADF, the ^3^LE state should lie well above the CT states [5].

Upon geometry optimization of the S_1_ and T_1_ states of CMA1, the calculated ΔE_ST_ decreases from 0.3 eV to 0.16 eV, which is low enough to enable the RISC process [21]. Due to the Stokes shift [33], the calculated energies for fluorescence (S_1_-S_0_@S_1_) and phosphorescence (T_1_-S_0_@T_1_) are lower than the corresponding absorption energies: 2.12 eV vs. 3.07 eV and 2.23 eV vs. 2.77 eV for S_1_ and T_1_, respectively. Like the S_0_ state, the carbene and amide remain nearly coplanar in the relaxed T_1_ geometry, but adopt a perpendicular orientation in the relaxed S_1_ geometry (Figure 1). High PLQY requires high oscillator strength coefficients (*f* or probability to emit electromagnetic radiation) in order for the radiative decay to outpace the nonradiative decay [34], which is hence compromised upon rotation. This is due to the lowering of the oscillator strength coefficients with an increasing torsion angle between the ligands of CMA1. In the nearly coplanar S_0_ geometry, the S_0_-S_1_ oscillator strength (0.1927) is larger compared to the maximum S_1_-S_0_ oscillator strength (0.1110) in the twisted S_1_ geometry. This is due to *f* being directly proportional to the squared value of the transition dipole moment (|*µ*_S1→S0_|^2^) [35], which, in turn, is directly proportional to the overlap integral between HOMO and LUMO. The fully rotated S_1_ state geometry possesses lower values for both HOMO-LUMO overlap integral and oscillator strength coefficient compared to S_1_ state in near co-planar geometry, while remaining significantly high at twisted S_1_ geometries, thereby explaining radiative transitions. This parallels with the lowest calculated ΔE_ST_ for the fully rotated S_1_ geometry (0.16 eV) and the largest (0.29 eV) for the S_1_ geometry fixed to coplanar orientation between the ligands.

Two more measurements are useful to add for the purpose of this work: namely, electrostatic dipole moments together with Au-C and Au-N bond dissociation energies. In the S_0_ ground state of CMA1, a dipole of 10.5D is calculated roughly along the C(carbene)–Au–N(amide) axis towards the carbene, reversing its direction in the S_1_@S_0_ state, while decreasing in magnitude down to 2.9D. The change arises from the CT character of the S_1_ state, leading to the electron displacement from the Cz moiety to the carbene moiety [24]. The bond dissociation energies provide measures for the stability of the complexes. For CMA1, the dissociation energies are 394.9 kJ/mol vs. 376.5 kJ/mol for the Au-C and Au-N bond, respectively.

**Figure 2:**
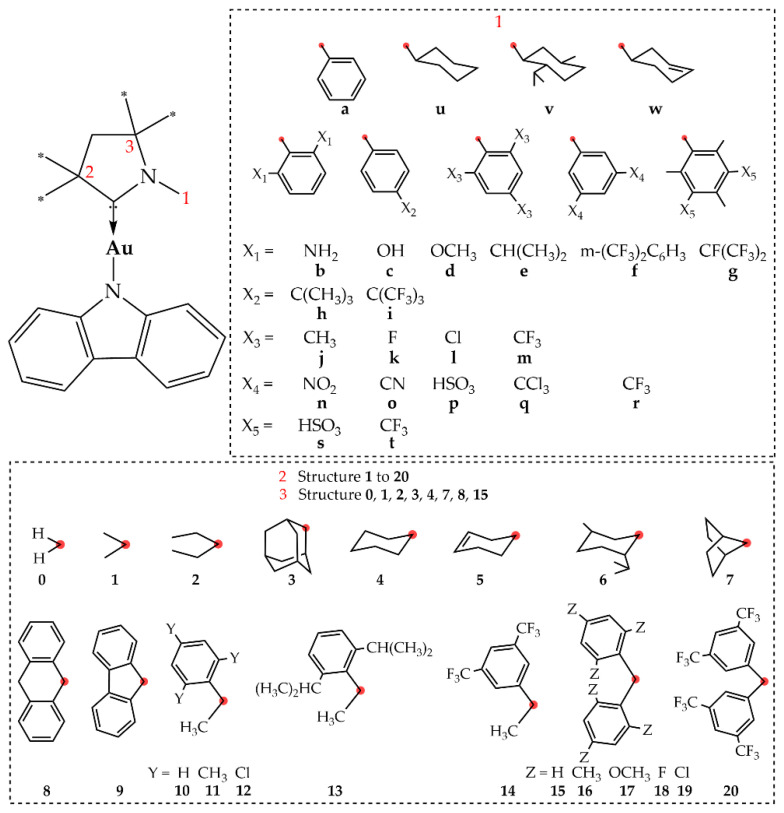
Employed structural modifications of CMA1, which are labeled as position 1-position 2-position 3, such that, e.g., **e-3-1** = CMA1.

Targeting molecular design strategies for improved PLQY via structure–property relationships, we systematically modified the CAAC ligand of CMA1, as detailed in Figure 2. Since our primary focus was on experimental synthetic prospects, we made the structure selection based on experimentally available carbenes, extending the dataset by hypothetical structures for the sake of a more thorough structural analysis. The structures were modified at positions 1, 2, and 3, while retaining the CAAC-5 backbone.

Regarding position 1, much of the previous work on CAACs has focused on the Dipp structure [6,7,8,11,12,13,14,15,36,37], while the 1,3,5-trimethylphenyl group (Mes) has been reported for N-heterocyclic carbenes (NHC) [17] and for diamidocarbenes [38]. Li et al. [17] synthesized a two-coordinate NHC(Mes)-Cu-Cz complex, which displays unique dual-emissive features from fluorescence (11.24 ns) and long-lived phosphorescence (32 ms) with total PLQY of 12.7%. Therefore, we included an aromatic C_6_-ring at position 1, substituted with various electron withdrawing and/or electron donating groups in order to evaluate the influence of electron density on the calculated parameters. We also included cyclohexanyl and cyclohexenyl substituents in the same position for comparison with the aromatic ring. Regarding position 2, CMA emitters with groups such as methyl, ethyl, menthyl, and adamantyl substituted on CAAC have been reported [7,8,9]. Particularly, the experimentally synthesized **e-6-1** gold complex in the 2-MeTHF solution exhibits a high PLQY of 95% and a 1.2 µs lifetime [8]. In our work, we supplemented the previously reported functional groups with various others in order to evaluate, e.g., π systems at this position. Correspondingly, we evaluated a smaller subset of substituents at position 3, where previous reports concerning CAACs have focused on substituent **1** [5,6,7,8,9,11,12,13,14,15,16].

### 2.1. Composition of the Frontier Orbitals

All the studied molecules have nearly identical HOMO orbitals, localized on the Cz moiety, which is the same for all complexes. Hence, HOMO is only slightly affected by the studied variations in the CAAC moiety where the calculated Au contributions are around 3%, as in CMA1. The effect is the strongest for position 1, where combining the phenyl group with m-(CF_3_)_2_C_6_H_3_ substituents at ortho-position (**f-1-1**) provides the lowest (2.5%), and HSO_3_ groups at meta-positions (**p-1-1** and **s-1-1**) the second highest (3.4%) contribution of Au.

Because the HOMO remains nearly unchanged, the spatial separation of the frontier orbitals, measured in terms of the HOMO-LUMO overlap integral, depends mainly on the LUMO orbital. Since the LUMO is localized on Au–carbene, the percentage of Au contribution varies strongly as a function of CAAC, ranging from 1% to 16%, and consequently the overlap integral ranges between 0.14 and 0.38. It follows that the Au contribution of LUMO and the overlap integral are strongly correlated—decreasing Au contribution indicates localization of LUMO towards CAAC, and, hence, farther away from the Cz-localized HOMO, increasing spatial separation of the orbitals. Therefore, we simplify the discussion of the effects of structural variations so as to focus on HOMO-LUMO overlap integrals. The results are tabulated in Tables S1–S3.

Position 1 has the strongest influence on the overlap integral, followed by position 2, while position 3 makes little contribution to the LUMO orbital and, hence, to the orbital overlap, as it is located far from the N$-\ddot{C}\to$ Au center. Concerning position 1, electron-withdrawing and electron-donating substituents play an expectedly major role. The former substituents have a stronger effect, arising from electron withdrawal from Au such that LUMO becomes primarily localized on CAAC at position 1. Combining the phenyl group with electron-withdrawing meta-substituents leads to a reduction in the overlap integral, from 0.31 to 0.14, in the order CF_3_ (**r-1-1**) > CCl_3_ (**q-1-1**) HSO_3_ > (**p-1-1**) > CN (**o-1-1**) > NO_2_ (**n-1-1**), due to the gradual shift in the LUMO distribution from N$-\ddot{C}$ to the substituted phenyl ring (Figure S1). The order of CF_3_ > HSO_3_ is reproduced in the presence of methyl-substituents (**t-1-1** vs. **s-1-1**). Electron-donating ortho-NH_2_-substituents (**b-1-1**) increase the overlap integral to its maximum value of 0.38 for this dataset. Ortho-substituents, being closest to the CAAC-5 backbone, have a stronger impact than the para-substituents. This is best exemplified by electron-withdrawing fluoroalkyl groups in ortho vs. para positions, where the two ortho-substituents decrease the overlap integral from 0.36 of CMA1 down to 0.23 **(f-1-1)** or 0.20 (**g-1-1**), while remaining at 0.33 with the para-substituent (**i-1-1**), the combination of ortho- and para-substituents (**m-1-1**) resulting in an overlap integral of 0.21. Direct comparison of CF_3_ substituents (**m-1-1**, **r-1-1**) indicates a stronger effect for the ortho- than for the meta-position. Chloride (**l-1-1**) has a weaker withdrawing effect, while fluoride (**k-1-1**), hydroxyl (**c-1-1**), methoxy (**d-1-1**), and alkyl groups (**e-1-1**, **h-1-1**, and **j-1-1**) have little effect on the overlap integral, and the same is true for saturation of the phenyl ring (**u-1-1**, **v-1-1**, **w-1-1**).

Concerning position 2, alkyl (**1**,**2**) and cycloalkyl (**3**–**7**) groups have no significant effect on the overlap integral. A modest effect arises from the bulky adamantyl (**3**) at position 2 canceling some of the electron withdrawal at position 1. This is best exemplified by **f-1-1** vs. **f-3-1**, where the overlap integral consequently increases from 0.23 to 0.27. More notable effects are observed for the conjugated rings at position 2, which we explored in conjunction with substituent **e** at position 1. These substituents tend to shift the LUMO orbital towards position 2, hence lowering the overlap integral, and the effect can be strengthened by ring substitutions. The effect is particularly strong for chlorides, where the maximum effect among the dataset is obtained for **e-19-1**, the resulting electron withdrawal decreasing the orbital overlap from 0.36 to 0.21. A similar but weaker effect is seen for **e-12-1** having fewer chlorides.

### 2.2. Vertical Excitations

As summarized in Tables S1–S3, vertical S_0_-S_1_ excitation energies at optimized S_0_ geometries are within the visible region, ranging from 1.73 eV to 3.36 eV, i.e., ca. 400–700 nm, while the corresponding S_0_-T_1_ energies range from 1.68 eV to 3.14 eV. Based on the NTOs, both the S_0_-S_1_ and S_0_-T_1_ excitations are characterized primarily as CT from HOMO to LUMO. For S_1_, HOMO-LUMO accounts for 97 ± 2% of CT in most cases, vs. 97% for CMA1, hence remaining practically unaffected by any of the structural modifications employed in this dataset. T_1_ shows slightly more variation, but only concerning position 1, where the range of HOMO-LUMO character is 70–95%, the upper limit roughly representing the contribution in CMA1 and in any of its employed structural modifications at positions 2 and 3. Hence, the effects of structural modifications are limited to the substitutions at position 1 having the potential to lower the HOMO-LUMO character of the S_0_-T_1_ excitation, where the lower limit of 70% is obtained for **f-1-1**.

**Figure 3:**
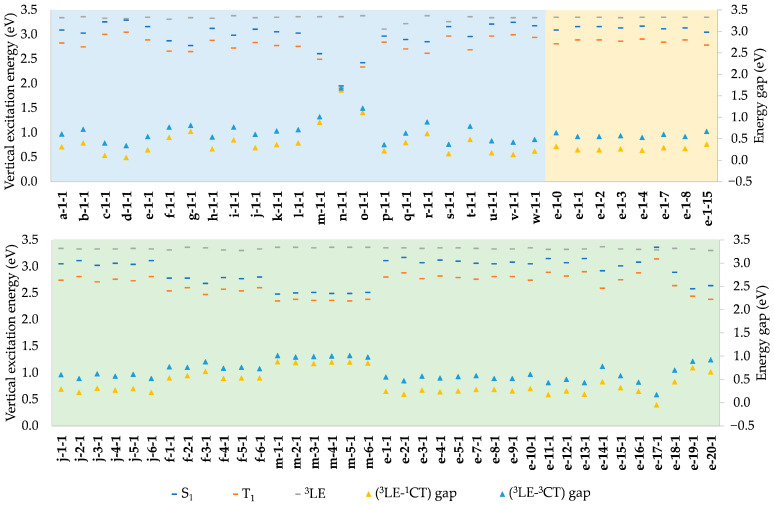
Vertical excitation energies and (^3^LE-^1^CT) and (^3^LE-^3^CT) gaps of complexes modified at positions 1 (blue), 2 (green), and 3 (yellow), where ^1^CT and ^3^CT are singlet and triplet CT characters, respectively.

A general overview of S_0_-S_1_ and S_0_-T_1_ vertical excitation energies is presented in Figure 3. Structural modifications at positions 1, 2, and 3 generally result in S_1_ states being above the T_1_ states by an approximate factor of 1.1, indicating that excitation energies are directly proportional to the S_1_-T_1_ energy gap. Hence, a low ΔE_ST_, which is desirable for efficient TADF, is most easily achieved when the excitation energies at the lower end of the desired color range. For our dataset, ΔE_ST_ ranges between 0.05 and 0.33 eV, vs. 0.30 eV for CMA1, which is known to operate via TADF. From that point of view, TADF cannot be excluded for any of the molecules included here.

**Figure 4:**
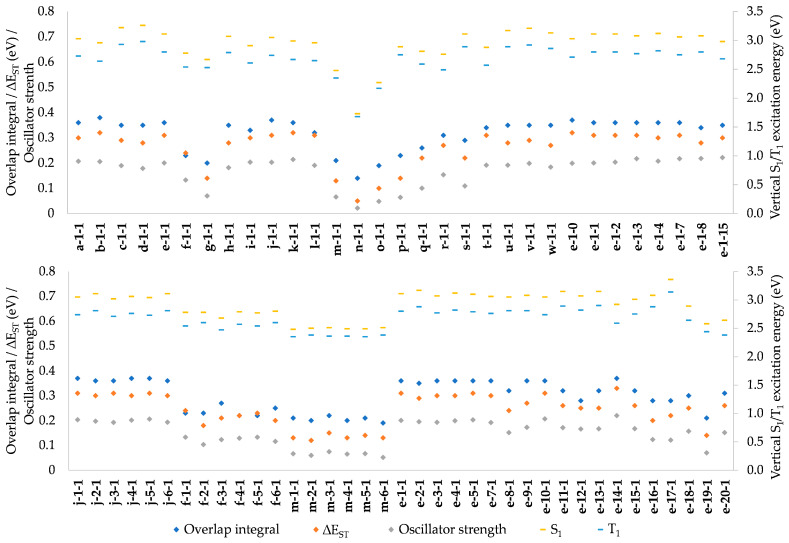
Comparison of the HOMO-LUMO overlap integral, S_0_-S_1_ oscillator strength, ∆E_ST_, and vertical excitation energies.

As illustrated in Figure 4, ΔE_ST_ is strongly correlated with the orbital integral discussed above; thus, it is unnecessary to repeat the discussion of the effects of structural modifications in this context. Likewise, ΔE_ST_ is strongly correlated with S_0_-S_1_ and S_0_-T_1_ excitation energies, as well as with S_0_-S_1_ oscillator strength, with a major implication of the desired low ΔE_ST_ being unachievable with the desired high oscillator strength. Therefore, compromises are necessary in molecular design for improved PLQY.

In addition, one needs to consider the ^3^LE(Cz), due to its interference on TADF. This usually corresponds to the T_2_ state, but is, in some cases, found at higher triplet states, T_3_-T_6_. The employed modifications of CAAC have little effect on ^3^LE(Cz), the energies averaging at 3.34 eV with a standard deviation of 0.04 eV, and are thus much higher than the energy of S_1_ (Figure 3, Tables S1–S3), which is beneficial for the TADF mechanism [5]. The few exceptions are those having high S_1_ energy, while for **e-17-1**, with the highest S_1_ energy, the order becomes reversed. The ^3^LE-^1^CT (and ^3^LE-^3^CT) gap is hence strongly correlated to vertical S_0_-S_1_ (and S_0_-T_1_) excitation energies, as illustrated in Figure 3—the larger the excitation energy, the smaller the gap.

### 2.3. Electrostatic Dipole Moments

The transition dipole moment, which represents the difference between the ground and excited states, is directly proportional to the square root of the oscillator strength coefficient [35]. Therefore, maximizing the transition dipole moment effectively enhances the oscillator strength. This facilitates the prospect of devising and prognosticating bright CMA materials using this value as a selection criterion.

Our calculated results indicate that, in the S_0_ ground state, the dipole moment vector points toward the carbene roughly along the $\ddot{C}\to$ Au−N axis, with a magnitude ranging between 8.7D and 12.7D for this dataset. The direction reverses for the S_1_@S_0_ state, originating from the amide–carbene electron rearrangement due to the CT character of the S1 state, as was discussed for CMA1 [24]. At the same time, the magnitude extends over a wider range, 2.5D–14.3D, (Tables S1–S3), but it is usually below 6D, and therefore lower than in the S_0_ state. Exceptions where S_1_@S_0_ is above 6D and comparable in magnitude to S_0_ arise from ortho-substitution of the phenyl group at position 1 by electron-withdrawing fluoroalkyls (**f-1-1**, **g-1-1**, and **m-1-1**). In the case of strongly electron-withdrawing meta-substituents on the phenyl group at position 1, the magnitude of the S_1_@S_0_ dipole reaches its maximum within the dataset of over 13D, hence exceeding the magnitude of the opposite S_0_ dipole (**n-1-1**, **o-1-1**, and **p-1-1**). Regarding position 2, approximately equal magnitudes of S_0_ and of S_1_@S_0_ dipoles are calculated for Cl- and CF_3_-substituted **e-19-1** and **e-20-1**, while substitutions at position 3 have, in practice, no effect.

### 2.4. Au-C and Au-N Bond Dissociation Energies

The calculated bond dissociation energy of the Au-N bond varies between 355 and 398 kJ/mol, while the corresponding range for the Au-C bond is somewhat wider, 365–420 kJ/mol, which is due to focus of this work on the modification of CAAC, rather than on the amide ligand. Both bonds are stabilized particularly by HSO_3_ groups on the phenyl ring at position 1 (**p-1-1** and **s-1-1**) and OCH_3_ groups on the aromatic rings at position 2 (**e-17-1**), and correspondingly destabilized by halogens and electron-withdrawing fluoroalkyls substituted to the rings. Comparing the Au-C and Au-N bond dissociation energies, the former are systematically higher, by 5% on average.

### 2.5. S_1_ and T_1_ Excited State Optimizations

**Figure 5:**
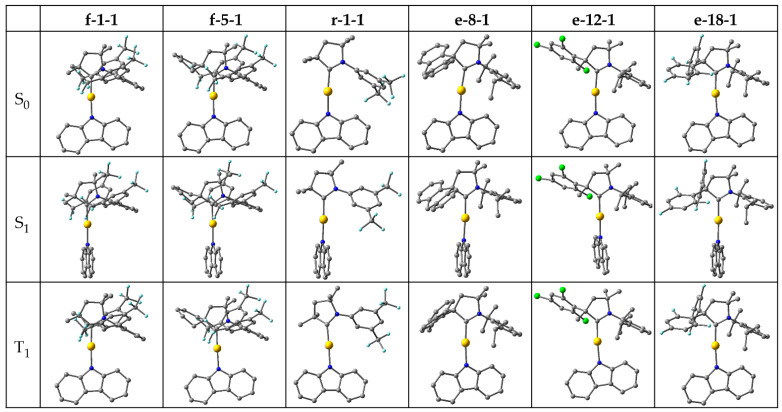
Optimized structures in S_0_, S_1_, and T_1_ states.

Subsequently, we selected promising candidates for the study of emissions by fluorescence and phosphorescence, which required geometry optimizations of the excited S_1_ and T_1_ states, respectively. The selection was made based on consideration of ∆E_ST_, oscillator strength and the HOMO-LUMO overlap integral, while the fourth decisive criterion, that ^3^LE must not interfere with the charge transfer, is satisfied by almost all studied complexes (Figure 3). For ∆E_ST_, we set a threshold of 0.27 eV so as to be measurably lower than that of our reference, CMA1 (0.30 eV). The threshold for oscillator strength was set at 0.13, which is lower than for CMA1 (0.1927), thus compromising oscillator strength in favor of ∆E_ST_. For the overlap integral, strongly correlating with ∆E_ST_, the threshold was set at 0.32, and hence measurably lower than for CMA1 (0.36). Based on these three criteria, six complexes were selected for excited state geometry optimizations: **f-1-1**, **f-5-1**, **r-1-1**, **e-8-1**, **e-12-1**, and **e-18-1** (Figure 5).

The optimized excited state energies are summarized in Table 1, with corresponding vertical excitations included for comparison. The carbene and amide ligands remain nearly coplanar in the optimized T_1_ geometries, analogous to the S_0_ ground state, but rotate perpendicular to each other in the S_1_ geometries. The rotation significantly lowers the S_1_ energy such that S_1_ and T_1_ energy levels reach degeneracy for **f-1-1**, **f-5-1,** and **r-1-1** and near-degeneracy for **e-8-1**, **e-12-1,** and **e-18-1**, where ΔE_ST_ ranges from 0.10 eV to 0.14 eV, and is thus lower than for the reference CMA1 (0.16 eV). The ^3^LE energy remains nearly constant and desirably high above the CT states. The S_1_-S_0_ oscillator strength decreases to almost zero at full rotation, while reaching its maximum value at a fixed coplanar orientation of carbene and amide, such that twisted geometry is required for efficient fluorescence. Among the studied complexes, a full rotation to coplanarity requires 0.12–0.27 eV energy, thus bringing ΔE_ST_ to the range of 0.2–0.3 eV, i.e., the magnitude calculated for the vertical excitations, which is low enough for efficient TADF.

For complexes lacking a bulky group in position 2 (**f-1-1**, **f-5-1**, and **r-1-1**), the oscillator strength remains lower at the coplanar orientation, while for the bulky **e**-substituents (**e-8-1**, **e-12-1**, and **e-18-1**), the maximum oscillator strength is calculated as being comparable to or even higher than that for CMA1 (0.1110). The latter substituents are calculated to emit fluorescence well within the visible region, 2.0–2.3 eV, corresponding roughly to an orange–green color.

### 2.6. Synthesis, Structure, and Photophysical Properties for e-2-1

**Figure 6:**
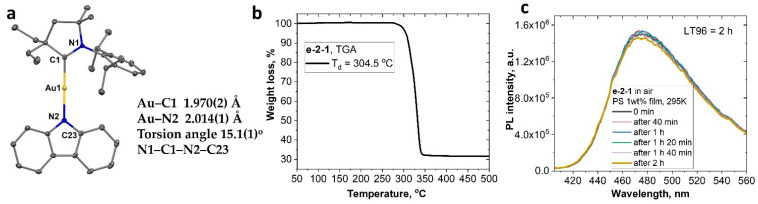
Single-crystal X-ray structure of **e-2-1** (**a**). The ellipsoids are shown at 50% probability. Thermogravimetric analysis curve for complex **e-2-1** (**b**); a 290 nm UV light photostability test for complex **e-2-1** in polystyrene (PS) 1% matrix. LT_96_ denotes the time after initial CT intensity dropped by 4% (**c**).

Finally, we develop our discussion and focus more on the factor of the steric protection of the metal atom in the CMA complexes. We and others recently demonstrated the importance of the bulky groups in position 2 in order to obtain bright CMA emitters [6,7,39]. It has been demonstrated that an increase in the steric protection of position 2 (methyl < ethyl < cyclohexyl < adamantyl < menthyl) results in an increase in the PLQY values, from 30% up to 100% in a series of copper CMA complexes analogous to CMA1. Distortion of the linear geometry around the metal atom (or Renner–Teller distortion [10,18]) results in poor quantum yields in the case of the less sterically protected CMA–copper complexes. We synthesized and fully characterized the gold complex **e-2-1**, with two ethyl groups in position 2, comparing it with the reference CMA1, which contains a bulky adamantyl, ^Ad^CAAC-carbene. Complex **e-2-1** is obtained from the (^Et2^CAAC)AuCl and carbazole in the presence of the KO*^t^*Bu base in 82% yield. It is fully spectroscopically characterized and demonstrates stability in air for months. The molecular structure of **e-2-1** was confirmed by single-crystal X-ray diffraction (Figure 6). Complexes **e-2-1** and CMA1 have a similar two-coordinate geometry for the gold atom, with negligible deviation from linearity, with differences in Au-C and Au-N bond lengths laying within the error of the experiment. The torsion angle (C1-N1-N2-C23, Figure 6) between carbene and carbazole ligands for **e-2-1** is ca. 15.1(1)°, which is close to the 17.7(2)° of CMA1, indicating the near coplanar orientation of the ligands. 

The thermal stability of the complex was evaluated with thermogravimetric analysis (TGA, under nitrogen, Figure 6b). The decomposition temperature (*T_d_*) for the gold complex **e-2-1** is 304.5 °C, which is 30 °C lower than that for CMA1 (334 °C). Thereafter, we performed the photostability test for complex **e-2-1** in a PS matrix with 1% concentration by weight under nitrogen, while irradiating the sample with hard UV light at 290 nm using a 75 W xenon lamp. Complex **e-2-1** shows excellent photostability LT_96_ = 120 min (Figure 6c), without any noticeable degradation. Similar CMA complexes [10] with monocyclic CAAC (LT_50_ = 90 min) and bicyclic BiCAAC (LT_85_ = 150 min) carbene ligands exhibit greater or comparable photostability to the 290 nm UV light exposure when compared with complex **e-2-1**. Our results indicate that complex **e-2-1** is highly suitable for the fabrication of the OLED, while holding a great promise for improving the operational stability of the OLED device.

**Figure 7:**
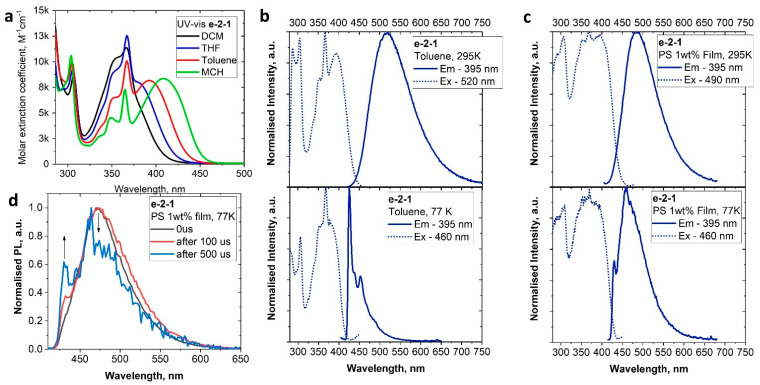
UV-vis spectra in various solvents for complex **e-2-1** (**a**); PL (em—emission; ex—excitation spectrum) of **e-2-1** in toluene solution at 295K ((**b**), **top**) and 77K ((**b**), **bottom**); PL in PS matrix with 1% concentration by weight at 295K ((**c**), **top** PS 1%wt), and 77K ((**c**), **bottom**). The PL profiles after various delays (0, 100, and 500 µs) for **e-2-1** at 77K in PS 1%wt film (**d**) demonstrating a contribution of the ^3^LE(Cz) phosphorescence.

The redox behavior of **e-2-1** was analyzed in THF solution using [*^n^*Bu_4_N]PF_6_ as the supporting electrolyte (Figure S5, Table S5). Complex **e-2-1** shows a quasi-reversible, carbene ligand-centered reduction process at *E*_1/2_ value −2.72 V. All oxidation processes are centered on the carbazole (similar to CMA1) and irreversible, with *E_p_* values at +0.15, +0.37, and +0.93 V (Table S5). The redox potentials and energy levels of HOMO (−5.61 eV) and LUMO (−2.67 eV) for **e-2-1** are similar to those of CMA1. The UV-vis absorption spectra in solvents with different polarity (Figure 7a) show a broad L(M)L charge transfer absorption. Negative solvatochromism for the L(M)L CT band is characteristic for the CMA complexes. We measured a 65 nm blue shift upon an increase in the solvent polarity from methylcyclohexane (MCH) to dichloromethane (DCM, Figure 7a).

The PL spectra were measured in toluene solution and PS matrix at 295 and 77K (Table 2 and Figure 7b,c). Compound **e-2-1** shows a broad CT PL profile with up to 14 nm blue shift in both toluene (484 nm) and PS matrix (518 nm) compared to CMA1 (Table 2). The excited state lifetime is ca. 1.1 µs, which is only slightly shorter compared to that of CMA1 (ca. 1.2 µs). Upon cooling to 77K, the **e-2-1** PL spectra are blue-shifted to 425 nm, while exhibiting a vibronically resolved profile indicative of a significant contribution in PL from both CT and ^3^LE(Cz) states, where the ^3^LE(Cz) contribution is clearly visible after applying a 500 µs delay (Figure 7d). The frozen toluene solution for the excited state lifetime of **e-2-1** increased to several hundred microseconds, although it is shorter than that of CMA1 (301 µs at 77K). The energy difference between CT and ^3^LE(Cz) states is slightly smaller for compound **e-2-1** (0.16 eV) compared with CMA1 (0.19 eV), while the ^3^LE(Cz) state is higher in energy compared with CT state for both complexes (Table 2). The steric protection of the metal center in the CMA complexes is usually considered as an important factor to rationalize high observed PLQY (Φ) values in solutions. However, the less sterically protected complex **e-2-1** shows unity PLQY values in toluene solution similar to those of the archetype CMA1 complex (Table 2), or three-fold higher PLQY values compared to copper-based analogue of **e-2-1** [7]. Unity PLQY values, together with a shorter excited state lifetime for the **e-2-1** complex, result in spectacular radiative rates approaching 10^6^ s^−1^. Such a difference in PL performance for **e-2-1** can be associated with a stronger preference for the gold(I) complexes to retain linear geometry, thus avoiding Renner–Teller distortion. This, together with the higher spin–orbit coupling values for gold compared with copper complexes, may outcompete the importance of the steric protection for the gold complexes. This enables the use of much more affordable CAAC carbenes with smaller substituents compared with bulky and expensive adamantyl-substituted CAAC carbenes.

## 3. Materials and Methods

### 3.1. Computational Details

Gas-phase DFT calculations of CMAs were carried out using the global hybrid MN15 functional by Truhlar and coworkers [27], in combination with the def2-TZVP basis set by Ahlrichs and coworkers [28,29]. The relativistic effective core potential of 60 electrons was used to describe the core electron of Au [40]. The ground states were studied by DFT, and the excited states by TD-DFT [41], using Tamm–Dancoff approximation [30]. The employed method provides excited state energies that do not suffer from the underestimation typical for TD-DFT [42,43], as indicated by previous calculations of CMAs [7,11,16], as well as by earlier comparison to T_1_ energies calculated by unrestricted DFT, such that the unrestricted and TD-DFT T_1_ energies differed by only 0.004 eV [25]. All calculations were carried out by Gaussian 16 [44]. Metal contributions to HOMO and LUMO were calculated by the Mulliken population analysis, and HOMO-LUMO overlap integrals were calculated using the Multiwfn program [45].

### 3.2. Experimental Details

Synthesis of **e-2-1**: In a Schlenk flask, (^Et2^CAAC)AuCl [39] (1 mmol, 545 mg), carbazole (1 mmol, 168 mg), and tBuOK (1 mmol, 112 mg) in THF (20 mL) were stirred for 8 h. The mixture was evaporated, extracted with CH_2_Cl_2_, and filtered through Celite^®^. The filtrate was concentrated and washed with hexane to afford the pure product as an off-white solid. Yield: 554 mg (0.82 mmol; 82%).

^1^H NMR (500 MHz, CD_2_Cl_2_): δ 7.94 (d, *J* = 7.6 Hz, 2H, CH Cz), 7.65 (t, *J* = 7.8 Hz, 1H, CH-aromatic CAAC), 7.45 (d, *J* = 7.6 Hz, 2H, CH-aromatic CAAC), 7.12 (t, *J* = 9 Hz, 2H, CH Cz), 6.94–6.91 (m, 4H, overlapping CH Cz), 2.97 (sept, *J* = 6.7 Hz, 2H, C*H*(CH_3_)_2_), 2.17 (s, 2H, C*H*_2_), 2.13–2.00 (m, 4H, CH_2_CH_3_), 1.48 (s, 6H, 2CH_3_), 1.38 (d, *J* = 6.7 Hz, 6H, CH(C*H*_3_)_2_), 1.35 (d, *J* = 6.7 Hz, 6H, CH(CH_3_)_2_), 1.22 (t, *J* = 7.5 Hz, 6H, CH_2_CH_3_) ppm. ^13^C NMR (125 MHz, CD_2_Cl_2_): δ 242.2 (C carbene), 150.0 (C*_ipso_*, Cz), 146.2 (C*_ipso_*), 135.6 (C*_ipso_*), 130.1 (*p*-CH CAAC), 125.6 (*m*-CH), 124.3 (C*_ipso_*, Cz), 123.7 (CH, Cz), 119.5 (CH, Cz), 116.2 (CH, Cz), 114.1 (CH, Cz), 80.5 (C_q_), 62.7 (C_q_), 42.7 (CH_2_), 32.2 (CH_2_), 29.6 (CH_3_), 29.5 (CH_3_), 26.7 (CH), 23.1 (CH_3_), 9.8 (CH_2_CH_3_) ppm. Anal. Calcd. for C_34_H_43_AuN_2_ (676.70): C, 60.35; H, 6.41; N, 4.14. Found: C, 60.61; H, 6.53; N, 4.20. C_34_H_44_AuN_2_ theoretical [M+H^+^] = 677.3165, HRMS (APCI(ASAP)) = 677.3172.

## 4. Conclusions

In summary, we have carried out a DFT study of over 60 CMA complexes in order to evaluate their potential as TADF emitters, focusing on the specific effects of the CAAC ligand. Several parameters that have been previously shown to be important for the PL properties of this family of complexes were studied as a function of CAAC, where the choice of its structural modifications was primarily based on experimental synthesis prospects. As a consequence, a diverse dataset of structures was generated, encompassing their predicted photoluminescence properties, and serving as the basis for making selections for the synthesis of efficient CMA emitters for OLED devices. However, we note that modification of the CAAC ligand alone offers limited design strategies, such that future computational work will need to focus on the combined effect of the CAAC and amide ligands.

We demonstrated that the properties of the CMAs are governed by the overlap integral between the HOMO and LUMO orbitals, where the HOMO is localized on the amide and the LUMO along the metal–carbene bond. The HOMO-LUMO transition accounts for nearly 100% of the charge transfer between the ground state and the lowest excited states. The states corresponding to localized triplet excitations usually lie well above the CT states. The HOMO-LUMO overlap was shown to be strongly correlated with vertical S_0_-S_1_ and S_0_-T_1_ excitation energies, exchange energies, and S_0_-S_1_ oscillator strength coefficients. The optimal combination of low exchange energy and high oscillator strength serves as a compromise required in order to design a bright CMA emitter.

Prioritizing low exchange energy over oscillator strength in making the compromise, we selected six CMAs for the geometry optimization of T_1_ and S_1_ states. The former relaxes to geometry analogous to S_0_, having carbene and amide nearly coplanar, while the latter rotate to perpendicular orientation, lowering both exchange energy and oscillator strength to near zero. Efficient fluorescence thus requires rotation toward coplanarity, which comes at the cost of increasing exchange energy. The highest oscillator strength coefficients are calculated for the CMA complexes with bulky substituents on the CAAC ligand.

Guided by the computational results, we synthesized and obtained a novel CMA complex, **e-2-1**, with spectacular luminescence properties (up to unity PLQY values and one microsecond excited state lifetime). We demonstrated that we could use much more affordable CAAC ligands with little steric protection. For instance, an ethyl-substituted CAAC-carbene is already sufficient to obtain gold-based CMA complexes with excellent performance. This finding paves the way towards more environmentally friendly and more sustainable production of the CMA complexes by eliminating the need for complex, multistep synthesis towards CAAC-carbenes with bulky substituents (such as adamantyl, menthyl, and others). Excellent photo and thermal stability of the CMA complex **e-2-1** makes it highly suitable for the fabrication of the OLED, while holding great promise for improving the operational stability of the OLED device.

## Figures and Tables

**Table 1:** S_1_ and T_1_ excited state optimizations.

		f-1-1	f-5-1	r-1-1	e-8-1	e-12-1	e-18-1
Vertical S_1_ excitation energy (eV)		2.78	2.77	2.76	3.05	3.07	2.89
Vertical T_1_ excitation energy (eV)		2.54	2.54	2.49	2.81	2.82	2.64
∆E_ST_ (eV) ^a^		0.24	0.23	0.27	0.24	0.25	0.25
Energy relative to optimized S_0_ (eV)	Optimized coplanar S_1_	2.49	2.49	2.38	2.82	2.77	2.63
	Optimized rotated S_1_	2.27	2.28	2.11	2.67	2.59	2.51
	Optimized coplanar T_1_	2.29	2.29	2.11	2.57	2.48	2.37
∆E_ST_ (eV) ^b^		−0.02	−0.01	0.00	0.10	0.11	0.14
^3^LE energy (eV) ^c^		3.20	3.19	3.27	3.24	3.25	3.26
Maximum S_1_-S_0_ oscillator strength ^d^		0.0256	0.0252	0.0330	0.0986	0.1347	0.1035
Fluorescence (S_1_-S_0_@S_1_) (eV)		1.85	1.87	1.47	2.29	2.06	2.01
Phosphorescence (T_1_-S_0_@T_1_) (eV)		2.00	2.00	1.42	2.28	2.14	2.07

^a^ Vertical S_1_ and T_1_ excitations. ^b^ Optimized S_1_ and T_1_ geometries. ^c^ Optimized T_1_ geometry. ^d^ Carbene and amide fixed coplanar.

**Table 2:** Photophysical properties of complexes **e-2-1** and CMA1 in various media.

		λ_em_ (nm)	τ (µs)	Φ (%) ^a^	k_r_ (10^5^ s^−1^) ^b^	k_nr_ (10^5^ s^−1^) ^c^	CT/^3^LE(Cz) (eV) ^d^	λ_em_ (nm, 77 K)	τ (µs, 77 K)
Toluene Solution	**e-2-1**	518	1.09	99	9.1	0.09	2.79/2.95	425	48.6 (45%), 196.6 (55%)
	CMA1	528	1.25	98	7.8	0.16	2.76/2.95	426	302
Polystyrene Matrix	**e-2-1** (1 wt%)	484	1.07	82	7.66	1.68	2.92/2.95	458	48.0 (63%), 188.3 (32%)
	CMA1 (5 wt%)	498	1.2	73	6.1	2.3	2.86/–	–	–

^a^ Quantum yields determined using an integrating sphere. ^b^ Radiative rate constant k_r_ = Φ/τ. ^c^ Nonradiative constant k_nr_ = (1 − Φ)/τ. ^d^ CT/^3^LE(Cz) energies based on the onset values of the emission spectra blue edge in MeTHF glasses at 77 K and 295 K.

## Data Availability

Cartesian coordinates of optimized structures for reproduction of the computations are available as Supplementary Materials.

## Supplementary Materials

The following supporting information can be downloaded at: https://www.mdpi.com/article/10.3390/molecules28114398/s1, Figure S1: LUMO orbitals of complexes modified at positions 1 and 3; Figure S2: Schematic energy of **e-8-1**, **e-12-1**, and **e-18-1** complexes: (a) vertical excitation energies indicated by the upward arrows; (b) energy levels at optimized S_1_ and T_1_ geometries with fluorescence (left) and phosphorescence (right) indicated by the downward arrows; Table S1: Calculated parameters for complexes modified at position 1; Table S2: Calculated parameters for complexes modified at position 2; Table S3: Calculated parameters for complexes modified at position 3; Figure S3: ^1^H NMR (500 MHz, CD_2_Cl_2_) for **e-2-1**; Figure S4: ^13^C NMR (125 MHz, CD_2_Cl_2_) for **e-2-1**; Table S4: UV-vis data for **e-2-1** in various solvents; Figure S5: Full range cyclic voltammogram for **e-2-1**. Recorded using a glassy carbon electrode in THF solution (1.4 mM) with [n-Bu4N]PF6 as supporting electrolyte (0.13 M), scan rate 0.1 Vs^−1^; Table S5: Formal electrode potentials (peak position *E*_p_ for irreversible and *E*_1/2_ for quasi-reversible processes (*), *V*, vs. FeCp_2_), onset potentials (*E, V*, vs. FeCp_2_), peak-to-peak separation, in parentheses for quasi-reversible processes (Δ*E_p_* in mV), *E_HOMO_/E_LUMO_* (eV), and band gap values (Δ*E*, eV) for the redox changes exhibited. Refs. [46,47] cited in SM file.

## Sample Availability

Samples of complex **e-2-1** are available from the authors.
