# Supplementary material for: Tailoring Carbene–Metal–Amides for Thermally Activated Delayed Fluorescence: A Computationally Guided Study on the Effect of Cyclic (Alkyl)(amino)carbenes
Source: Molecules. 2023 May 28;28(11):4398. doi: 10.3390/molecules28114398 (PMC10254582; doi:10.3390/molecules28114398)
Supplement: Supplementary file 1 [file molecules-28-04398-s001.zip › molecules-2403095-supplementary.pdf]

# Supplementary Materials

# Tailoring Carbene-Metal-Amides for Thermally Activated Delayed Fluorescence: A Computationally Guided Study on the Effect of Cyclic (Alkyl)(amino)carbenes

**Nguyen Le Phuoc [1], Alexander C. Brannan [2], Alexander S. Romanov [2], Mikko Linnolahti [1*]**

[1] Department of Chemistry, University of Eastern Finland, FI-80101 Joensuu, Finland
[2] Department of Chemistry, University of Manchester, Oxford Rd. Manchester, M13 9PL, United Kingdom
* Correspondence: mikko.linnolahti@uef.fi

## Computational Results

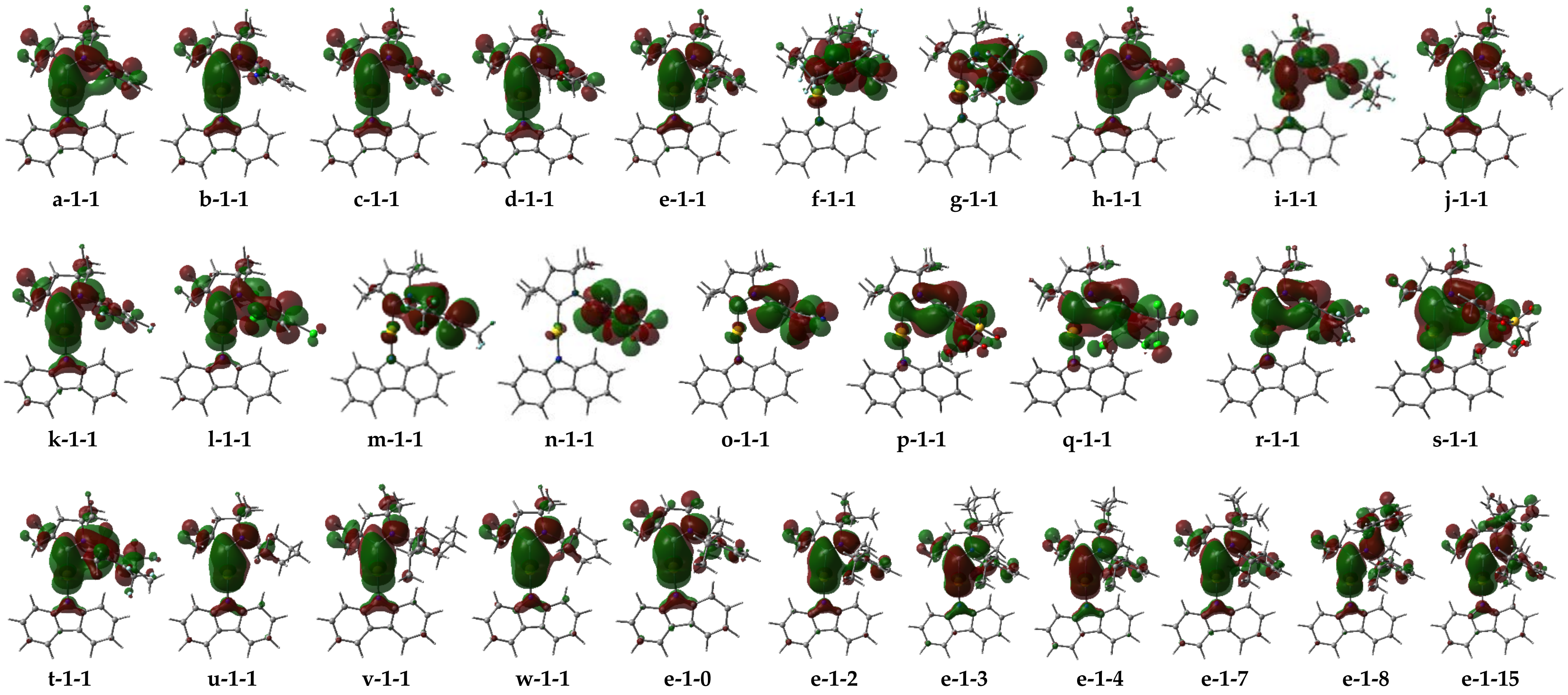


**Figure S1.** LUMO orbitals of complexes modified at positions 1 and 3

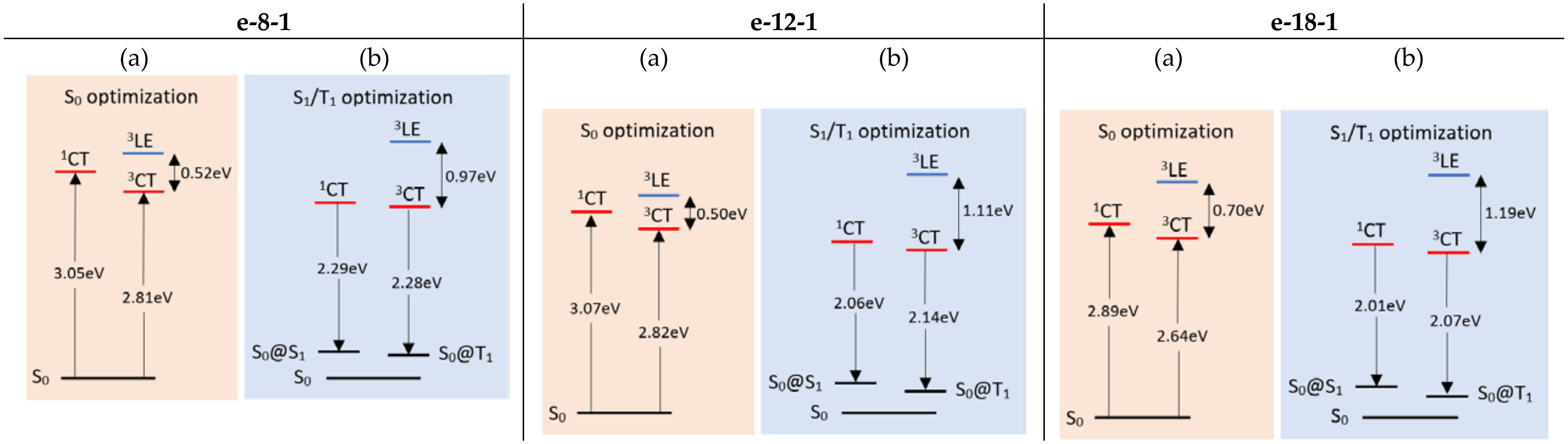


**Figure S2.** Schematic energy of **e-8-1**, **e-12-1**, and **e-18-1** complexes: (a) vertical excitation energies indicated by the upward arrows; (b) energy levels at optimized $S_1$ and $T_1$ geometries with fluorescence (left) and phosphorescence (right) indicated by the downward arrows.

**Table S1.** Calculated parameters for complexes modified at position 1

| | %Au in HOMO | %Au in LUMO | Overlap integral | Vertical excitations | | | | | | Dipole moments (D) | | Bond dissociation energy | | | |
|---|---|---|---|---|---|---|---|---|---|---|---|---|---|---|---|
| | | | | $^1$CT - $S_1$ | $^3$CT - $T_1$ | $^3$LE | | $\Delta E_{ST}$ | Oscillator | | | Au-N bond | | Au-C bond | |
| | | | | E (eV) | E (eV) | E (eV) | Character | (eV) | strength | $S_0$ | $S_1$@$S_0$ | kJ/mol | eV | kJ/mol | eV |
| **a-1-1** | 2.9 | 13.9 | 0.36 | 3.03 | 2.73 | 3.34 | $T_2$ ($^3$LE(Cz)) | 0.30 | 0.2073 | 11.7 | -2.5 | 367.4 | 3.81 | 385.9 | 4.00 |
| **b-1-1** | 2.8 | 15.2 | 0.38 | 2.96 | 2.64 | 3.36 | $T_2$ ($^3$LE(Cz)) | 0.32 | 0.2061 | 9.6 | -3.6 | 366.6 | 3.80 | 391.4 | 4.06 |
| **c-1-1** | 3.0 | 13.9 | 0.35 | 3.22 | 2.93 | 3.33 | $T_2$ ($^3$LE(Cz)) | 0.29 | 0.1900 | 10.7 | -4.8 | 372.8 | 3.86 | 398.2 | 4.13 |
| **d-1-1** | 3.1 | 14.4 | 0.35 | 3.26 | 2.98 | 3.32 | $T_2$ ($^3$LE(Cz)) | 0.28 | 0.1791 | 10.2 | -4.3 | 373.8 | 3.87 | 403.4 | 4.18 |
| **e-1-1** | 2.8 | 12.9 | 0.36 | 3.11 | 2.80 | 3.35 | $T_2$ ($^3$LE(Cz)) | 0.31 | 0.2005 | 10.8 | -2.7 | 376.7 | 3.90 | 401.3 | 4.16 |
| **f-1-1** | 2.5 | 1.9 | 0.23 | 2.78 | 2.54 | 3.31 | $T_6$ (mixed $^3$CT and $^3$LE(Cz)) | 0.24 | 0.1331 | 9.0 | -7.6 | 357.4 | 3.70 | 374.2 | 3.88 |
| **g-1-1** | 2.7 | 2.3 | 0.20 | 2.67 | 2.53 | 3.34 | $T_5$ ($^3$LE(Cz)) | 0.14 | 0.0699 | 10.4 | -6.6 | 370.0 | 3.83 | 374.4 | 3.88 |
| **h-1-1** | 2.9 | 14.5 | 0.35 | 3.07 | 2.79 | 3.33 | $T_2$ ($^3$LE(Cz)) | 0.28 | 0.1820 | 11.4 | -2.7 | 371.4 | 3.85 | 390.9 | 4.05 |
| **i-1-1** | 2.8 | 10.0 | 0.33 | 2.91 | 2.61 | 3.38 | $T_3$ (mixed $^3$CT and $^3$LE(Cz)) | 0.30 | 0.2037 | 12.2 | -3.9 | 364.1 | 3.77 | 378.7 | 3.92 |
| **j-1-1** | 2.9 | 13.9 | 0.37 | 3.05 | 2.74 | 3.34 | $T_2$ ($^3$LE(Cz)) | 0.31 | 0.2033 | 11.3 | -2.6 | 371.9 | 3.85 | 395.3 | 4.10 |
| **k-1-1** | 2.7 | 12.3 | 0.36 | 2.99 | 2.67 | 3.35 | $T_2$ ($^3$LE(Cz)) | 0.32 | 0.2146 | 11.2 | -2.6 | 364.5 | 3.78 | 379.7 | 3.94 |
| **l-1-1** | 2.7 | 7.9 | 0.32 | 2.96 | 2.65 | 3.36 | $T_4$ ($^3$LE(Cz)) | 0.31 | 0.1910 | 11.1 | -2.9 | 367.0 | 3.80 | 384.5 | 3.99 |
| **m-1-1** | 2.7 | 2.2 | 0.21 | 2.48 | 2.35 | 3.36 | $T_6$ ($^3$LE(Cz)) | 0.13 | 0.0661 | 10.9 | -8.9 | 364.8 | 3.78 | 380.0 | 3.94 |
| **n-1-1** | 2.8 | 1.0 | 0.14 | 1.73 | 1.68 | 3.36 | $T_6$ (mixed $^3$CT and $^3$LE(Cz)) | 0.05 | 0.0219 | 12.6 | -14.3 | 355.6 | 3.69 | 369.3 | 3.83 |
| **o-1-1** | 2.7 | 2.3 | 0.19 | 2.27 | 2.17 | 3.38 | $T_6$ ($^3$LE(Cz)) | 0.10 | 0.0483 | 12.7 | -13.1 | 355.2 | 3.68 | 368.5 | 3.82 |
| **p-1-1** | 3.4 | 4.5 | 0.23 | 2.89 | 2.75 | 3.11 | $T_2$ (mixed $^3$CT and $^3$LE(Cz)) | 0.14 | 0.0645 | 9.4 | -13.4 | 390.4 | 4.05 | 412.5 | 4.28 |
| **q-1-1** | 3.1 | 5.8 | 0.26 | 2.81 | 2.59 | 3.22 | $T_3$ (mixed $^3$CT and $^3$LE(Cz)) | 0.22 | 0.1008 | 11.0 | -6.6 | 373.9 | 3.87 | 390.9 | 4.05 |
| **r-1-1** | 2.8 | 8.0 | 0.31 | 2.76 | 2.49 | 3.38 | $T_5$ ($^3$LE(Cz)) | 0.27 | 0.1539 | 11.8 | -5.8 | 361.4 | 3.75 | 375.5 | 3.89 |
| **s-1-1** | 3.4 | 7.7 | 0.29 | 3.11 | 2.89 | 3.26 | $T_2$ (mixed $^3$CT and $^3$LE(Cz)) | 0.22 | 0.1095 | 8.8 | -9.5 | 398.0 | 4.12 | 419.9 | 4.35 |
| **t-1-1** | 2.8 | 9.3 | 0.34 | 2.88 | 2.57 | 3.36 | $T_3$ ($^3$LE(Cz)) | 0.31 | 0.1916 | 11.3 | -4.0 | 366.1 | 3.79 | 385.6 | 4.00 |
| **u-1-1** | 3.0 | 16.1 | 0.35 | 3.17 | 2.89 | 3.34 | $T_2$ ($^3$LE(Cz)) | 0.28 | 0.1916 | 11.8 | -2.9 | 362.9 | 3.76 | 386.6 | 4.01 |
| **v-1-1** | 3.0 | 14.0 | 0.35 | 3.21 | 2.92 | 3.34 | $T_2$ ($^3$LE(Cz)) | 0.29 | 0.1991 | 11.4 | -3.0 | 361.1 | 3.74 | 387.3 | 4.01 |
| **w-1-1** | 3.0 | 16.1 | 0.35 | 3.13 | 2.86 | 3.34 | $T_2$ ($^3$LE(Cz)) | 0.27 | 0.1841 | 11.6 | -2.9 | 362.6 | 3.76 | 387.0 | 4.01 |

**Table S2.** Calculated parameters for complexes modified at position 2

| | %Au in HOMO | %Au in LUMO | Overlap integral | Vertical excitations | | | | | | Dipole moments (D) | | Bond dissociation energy | | | |
|---|---|---|---|---|---|---|---|---|---|---|---|---|---|---|---|
| | | | | $^{1}$CT - $S_1$ | $^{3}$CT - $T_1$ | $^{3}$LE | | $\Delta E_{ST}$ | Oscillator | | | Au-N bond | | Au-C bond | |
| | | | | E (eV) | E (eV) | E (eV) | Character | (eV) | strength | $S_0$ | $S_1$@$S_0$ | kJ/mol | eV | kJ/mol | eV |
| **j-1-1** | 2.9 | 13.9 | 0.37 | 3.05 | 2.74 | 3.34 | $T_2$ ($^{3}$LE(Cz)) | 0.31 | 0.2033 | 11.3 | -2.6 | 371.9 | 3.85 | 395.3 | 4.10 |
| **j-2-1** | 2.9 | 11.9 | 0.36 | 3.11 | 2.81 | 3.33 | $T_2$ ($^{3}$LE(Cz)) | 0.30 | 0.1976 | 11.2 | -2.8 | 372.1 | 3.86 | 397.4 | 4.12 |
| **j-3-1** | 3.0 | 12.4 | 0.36 | 3.02 | 2.71 | 3.33 | $T_2$ ($^{3}$LE(Cz)) | 0.31 | 0.1922 | 10.8 | -2.8 | 370.9 | 3.84 | 389.8 | 4.04 |
| **j-4-1** | 2.9 | 13.7 | 0.37 | 3.06 | 2.76 | 3.33 | $T_2$ ($^{3}$LE(Cz)) | 0.30 | 0.2015 | 11.3 | -2.6 | 372.8 | 3.86 | 396.8 | 4.11 |
| **j-5-1** | 2.9 | 13.5 | 0.37 | 3.04 | 2.73 | 3.34 | $T_2$ ($^{3}$LE(Cz)) | 0.31 | 0.2058 | 11.2 | -2.8 | 372.3 | 3.86 | 395.8 | 4.10 |
| **j-6-1** | 3.0 | 12.8 | 0.36 | 3.11 | 2.81 | 3.33 | $T_2$ ($^{3}$LE(Cz)) | 0.30 | 0.1935 | 11.0 | -2.8 | 373.7 | 3.87 | 401.1 | 4.16 |
| **f-1-1** | 2.5 | 1.9 | 0.23 | 2.78 | 2.54 | 3.31 | $T_6$ (mixed $^{3}$CT and $^{3}$LE(Cz)) | 0.24 | 0.1331 | 9.0 | -7.6 | 357.4 | 3.70 | 374.2 | 3.88 |
| **f-2-1** | 2.6 | 2.8 | 0.23 | 2.78 | 2.60 | 3.36 | $T_5$ (mixed $^{3}$CT and $^{3}$LE(Cz)) | 0.18 | 0.1030 | 9.1 | -6.3 | 370.2 | 3.84 | 389.0 | 4.03 |
| **f-3-1** | 2.7 | 3.7 | 0.27 | 2.68 | 2.47 | 3.35 | $T_5$ (mixed $^{3}$CT and $^{3}$LE(Cz)) | 0.21 | 0.1228 | 8.7 | -6.1 | 362.7 | 3.76 | 370.5 | 3.84 |
| **f-4-1** | 2.6 | 1.7 | 0.22 | 2.79 | 2.57 | 3.31 | $T_5$ (mixed $^{3}$CT and $^{3}$LE(Cz)) | 0.22 | 0.1288 | 8.9 | -7.7 | 358.2 | 3.71 | 376.0 | 3.90 |
| **f-5-1** | 2.5 | 1.8 | 0.22 | 2.77 | 2.54 | 3.30 | $T_6$ (mixed $^{3}$CT and $^{3}$LE(Cz)) | 0.23 | 0.1330 | 8.8 | -7.8 | 357.5 | 3.71 | 376.0 | 3.90 |
| **f-6-1** | 3.0 | 3.6 | 0.25 | 2.80 | 2.60 | 3.33 | $T_3$ (mixed $^{3}$CT and $^{3}$LE(Cz)) | 0.20 | 0.1160 | 8.8 | -5.8 | 371.5 | 3.85 | 396.9 | 4.11 |
| **m-1-1** | 2.7 | 2.2 | 0.21 | 2.48 | 2.35 | 3.36 | $T_6$ ($^{3}$LE(Cz)) | 0.13 | 0.0661 | 10.9 | -8.9 | 364.8 | 3.78 | 380.0 | 3.94 |
| **m-2-1** | 2.7 | 2.0 | 0.20 | 2.50 | 2.38 | 3.36 | $T_6$ ($^{3}$LE(Cz)) | 0.12 | 0.0596 | 10.8 | -9.2 | 365.5 | 3.79 | 382.6 | 3.97 |
| **m-3-1** | 2.8 | 2.4 | 0.22 | 2.51 | 2.36 | 3.35 | $T_6$ ($^{3}$ (Cz)) | 0.15 | 0.0742 | 10.7 | -8.8 | 366.4 | 3.80 | 377.2 | 3.91 |
| **m-4-1** | 2.7 | 2.1 | 0.20 | 2.49 | 2.36 | 3.36 | $T_6$ ($^{3}$LE(Cz)) | 0.13 | 0.0648 | 11.0 | -9.2 | 365.7 | 3.79 | 382.1 | 3.96 |
| **m-5-1** | 2.7 | 2.2 | 0.21 | 2.49 | 2.35 | 3.36 | $T_6$ ($^{3}$LE(Cz)) | 0.14 | 0.0666 | 10.9 | -9.0 | 364.9 | 3.78 | 380.7 | 3.95 |
| **m-6-1** | 2.8 | 2.0 | 0.19 | 2.51 | 2.38 | 3.36 | $T_6$ ($^{3}$LE(Cz)) | 0.13 | 0.0512 | 10.6 | -9.2 | 366.6 | 3.80 | 389.6 | 4.04 |
| **e-1-1** | 2.8 | 12.9 | 0.36 | 3.11 | 2.80 | 3.35 | $T_2$ ($^{3}$LE(Cz)) | 0.31 | 0.2005 | 10.8 | -2.7 | 376.7 | 3.90 | 401.3 | 4.16 |
| **e-2-1** | 2.8 | 10.8 | 0.35 | 3.17 | 2.88 | 3.35 | $T_2$ ($^{3}$LE(Cz)) | 0.29 | 0.1952 | 10.7 | -3.0 | 375.5 | 3.89 | 403.2 | 4.18 |
| **e-3-1** | 3.0 | 11.4 | 0.36 | 3.07 | 2.77 | 3.34 | $T_2$ ($^{3}$LE(Cz)) | 0.30 | 0.1927 | 10.5 | -2.9 | 376.5 | 3.90 | 394.9 | 4.09 |
| **e-4-1** | 2.8 | 12.7 | 0.36 | 3.12 | 2.82 | 3.35 | $T_2$ ($^{3}$LE(Cz)) | 0.30 | 0.1994 | 10.8 | -2.8 | 377.3 | 3.91 | 403.1 | 4.18 |
| **e-5-1** | 2.8 | 12.6 | 0.36 | 3.10 | 2.79 | 3.35 | $T_2$ ($^{3}$LE(Cz)) | 0.31 | 0.2029 | 10.7 | -2.9 | 376.5 | 3.90 | 401.8 | 4.16 |
| **e-7-1** | 3.0 | 11.9 | 0.36 | 3.06 | 2.76 | 3.34 | $T_2$ ($^{3}$LE(Cz)) | 0.30 | 0.1920 | 10.6 | -2.9 | 372.5 | 3.86 | 395.8 | 4.10 |
| **e-8-1** | 3.0 | 8.6 | 0.32 | 3.05 | 2.81 | 3.33 | $T_2$ ($^{3}$LE(Cz)) | 0.24 | 0.1516 | 9.7 | -4.7 | 376.6 | 3.90 | 397.2 | 4.12 |

| | | | | | | | | | | | | | | | |
|---|---|---|---|---|---|---|---|---|---|---|---|---|---|---|---|
| **e-9-1** | 2.7 | 10.4 | 0.36 | 3.08 | 2.81 | 3.33 | $T_2$ ($^3$LE(Cz)) | 0.27 | 0.1726 | 10.7 | -3.1 | 381.5 | 3.95 | 398.8 | 4.13 |
| **e-10-1** | 2.7 | 10.4 | 0.36 | 3.05 | 2.74 | 3.35 | $T_2$ ($^3$LE(Cz)) | 0.31 | 0.2068 | 10.7 | -3.1 | 371.3 | 3.85 | 395.2 | 4.10 |
| **e-11-1** | 3.0 | 7.6 | 0.32 | 3.15 | 2.89 | 3.32 | $T_2$ ($^3$LE(Cz)) | 0.26 | 0.1713 | 11.0 | -3.7 | 369.8 | 3.83 | 386.7 | 4.01 |
| **e-12-1** | 2.9 | 5.4 | 0.28 | 3.07 | 2.82 | 3.32 | $T_2$ ($^3$LE(Cz)) | 0.25 | 0.1659 | 10.8 | -5.0 | 370.3 | 3.84 | 384.9 | 3.99 |
| **e-13-1** | 3.1 | 8.4 | 0.32 | 3.15 | 2.90 | 3.33 | $T_2$ ($^3$LE(Cz)) | 0.25 | 0.1669 | 10.7 | -4.0 | 374.5 | 3.88 | 390.1 | 4.04 |
| **e-14-1** | 2.6 | 9.9 | 0.37 | 2.92 | 2.59 | 3.37 | $T_3$ ($^3$LE(Cz)) | 0.33 | 0.2199 | 10.1 | -5.6 | 368.0 | 3.81 | 385.9 | 4.00 |
| **e-15-1** | 3.0 | 6.7 | 0.32 | 3.01 | 2.75 | 3.33 | $T_2$ ($^3$LE(Cz)) | 0.26 | 0.1672 | 10.4 | -4.4 | 373.1 | 3.87 | 390.9 | 4.05 |
| **e-16-1** | 3.2 | 3.9 | 0.28 | 3.08 | 2.88 | 3.32 | $T_2$ ($^3$LE(Cz)) | 0.20 | 0.1233 | 10.3 | -5.0 | 377.2 | 3.91 | 376.1 | 3.90 |
| **e-17-1** | 3.9 | 8.4 | 0.28 | 3.36 | 3.14 | 3.31 | $T_2$ ($^3$LE(Cz)) | 0.22 | 0.1212 | 11.1 | -4.1 | 387.4 | 4.01 | 417.0 | 4.32 |
| **e-18-1** | 2.9 | 5.1 | 0.30 | 2.89 | 2.64 | 3.34 | $T_2$ ($^3$LE(Cz)) | 0.25 | 0.1567 | 10.2 | -5.6 | 371.7 | 3.85 | 374.8 | 3.88 |
| **e-19-1** | 2.9 | 1.8 | 0.21 | 2.58 | 2.44 | 3.33 | $T_4$ (mixed $^3$CT and $^3$LE(Cz)) | 0.14 | 0.0696 | 10.0 | -9.4 | 374.3 | 3.88 | 365.3 | 3.79 |
| **e-20-1** | 2.9 | 4.9 | 0.31 | 2.64 | 2.38 | 3.30 | $T_4$ (mixed $^3$CT and $^3$LE(Cz)) | 0.26 | 0.1514 | 10.7 | -8.0 | 363.9 | 3.77 | 372.6 | 3.86 |

**Table S3.** Calculated parameters for complexes modified at position 3

| | %Au in HOMO | %Au in LUMO | Overlap integral | Vertical excitations | | | | | | Dipole moments (D) | | Bond dissociation energy | | | |
|---|---|---|---|---|---|---|---|---|---|---|---|---|---|---|---|
| | | | | $^1$CT - $S_1$ | $^3$CT - $T_1$ | $^3$LE | | $\Delta E_{ST}$ | Oscillator | | | Au-N bond | | Au-C bond | |
| | | | | E (eV) | E (eV) | E (eV) | Character | (eV) | strength | $S_0$ | $S_1$@$S_0$ | kJ/mol | eV | kJ/mol | eV |
| **e-1-0** | 2.8 | 13.5 | 0.37 | 3.03 | 2.71 | 3.35 | $T_2$ ($^3$LE(Cz)) | 0.32 | 0.1994 | 10.6 | -2.6 | 369.4 | 3.83 | 392.1 | 4.06 |
| **e-1-1** | 2.8 | 12.9 | 0.36 | 3.11 | 2.80 | 3.35 | $T_2$ ($^3$LE(Cz)) | 0.31 | 0.2005 | 10.8 | -2.7 | 376.7 | 3.90 | 401.3 | 4.16 |
| **e-1-2** | 2.8 | 12.9 | 0.36 | 3.11 | 2.80 | 3.35 | $T_2$ ($^3$LE(Cz)) | 0.31 | 0.2037 | 11.0 | -2.7 | 374.8 | 3.88 | 402.8 | 4.18 |
| **e-1-3** | 2.7 | 12.0 | 0.36 | 3.08 | 2.77 | 3.34 | $T_2$ ($^3$LE(Cz)) | 0.31 | 0.2176 | 11.5 | -2.6 | 372.9 | 3.87 | 393.7 | 4.08 |
| **e-1-4** | 2.8 | 13.0 | 0.36 | 3.12 | 2.82 | 3.35 | $T_2$ ($^3$LE(Cz)) | 0.30 | 0.2078 | 11.3 | -2.5 | 377.3 | 3.91 | 402.7 | 4.17 |
| **e-1-7** | 2.8 | 12.3 | 0.36 | 3.06 | 2.75 | 3.35 | $T_2$ ($^3$LE(Cz)) | 0.31 | 0.2176 | 11.2 | -2.6 | 372.9 | 3.86 | 395.1 | 4.09 |
| **e-1-8** | 2.8 | 10.2 | 0.34 | 3.08 | 2.80 | 3.35 | $T_2$ ($^3$LE(Cz)) | 0.28 | 0.2182 | 11.5 | -3.7 | 375.0 | 3.89 | 393.2 | 4.08 |
| **e-1-15** | 2.7 | 10.5 | 0.35 | 2.98 | 2.68 | 3.35 | $T_2$ ($^3$LE(Cz)) | 0.30 | 0.2218 | 11.3 | -3.2 | 371.9 | 3.85 | 386.7 | 4.01 |

## X-Ray Crystallography

Crystals suitable for X-ray diffraction study were obtained by slow layer diffusion of hexanes/petroleum ether into dichloromethane solution for **e-2-1** at room temperature. Crystals were mounted in oil on glass fiber and fixed on the diffractometer in a cold nitrogen stream. Data were collected using XtaLAB AFC11 (RINC): quarter-chi single diffractometer at 100 K. Data were processed using the CrystAlisPro-CCD and –RED software. Multi-scan absorption correction was applied for all crystals. For the final refinement, the contribution of severely disordered $CH_2Cl_2$ molecules in the crystals **e-2-1** was accounted for by applying a solvent void mask calculated using BYPASS, implemented through Olex2. Structures were solved by direct method/intrinsic phasing and refined by the full-matrix least-squares against $F^2$ in an anisotropic (for non-hydrogen atoms) approximation. All hydrogen atoms were positioned geometrically and constrained to ride on their parent atoms with C-H = 0.95-1.00 Å, and $U_{iso}$ = 1.2–1.5 $U_{eq}$ (parent atom). All calculations were performed using the SHELXL software and Olex2 graphical user interface.

**e-2-1**, CCDC number 2259783, $C_{35}H_{47}AuN_2$ ($M$ =676.67 g/mol): monoclinic, space group $P2_1/c$ (no. 14), $a$ = 9.71163(5) Å, $b$ = 18.40218(10) Å, $c$ = 17.11055(10) Å, $\beta$ = 95.3962(5)°, $V$ = 3044.36(3) Å$^3$, $Z$ = 4, $T$ = 100.00(10) K, μ(Cu K$\alpha$) = 9.241 mm$^{-1}$, $Dcalc$ = 1.476 g/cm$^3$, 20350 reflections measured (7.072° ≤ 2Θ ≤ 151.65°), 6090 unique ($R_{int}$ = 0.0181, $R_{sigma}$ = 0.0150) which were used in all calculations. The final $R_1$ was 0.0207 (I > 2σ(I)) and $wR_2$ was 0.0564 (all data).

## Experimental Details

**General Considerations.** Unless stated otherwise, all reactions were carried out in air. Solvents were distilled and dried as required. Potassium *tert*-butoxide and carbazole were purchased from FluoroChem. The carbene complex ($^{Et}$L)AuCl [1] were obtained according to literature procedures. $^1$H and $^{13}$C{$^1$H} NMR spectra were recorded using a Bruker Avance 500 MHz NMR spectrometer. $^1$H NMR spectra were referenced to $CD_2Cl_2$ at δ 5.32 ($^{13}$C, δ 53.84) ppm. All electrochemical experiments were performed using an Autolab PGSTAT 302N computer-controlled potentiostat. Cyclic voltammetry (CV) was performed using a three-electrode configuration consisting of either a glassy carbon macrodisk working electrode (GCE) (diameter of 3 mm; BASi, Indiana, USA) combined with a Pt wire counter electrode (99.99 %; GoodFellow, Cambridge, UK) and an Ag wire pseudo-reference electrode (99.99 %; GoodFellow, Cambridge, UK). The GCE was polished between experiments using alumina slurry (0.3 μm), rinsed in distilled water and subjected to brief sonication to remove any adhering alumina microparticles. The metal electrodes were then dried in an oven at 100 °C to remove residual traces of water, the GCE was left to air dry and residual traces of water were removed under vacuum. The Ag wire pseudoreference electrodes were calibrated to the ferrocene/ferrocenium couple in THF at the end of each run to allow for any drift in potential, following IUPAC recommendations [2]. All electrochemical measurements were performed at ambient temperatures under an inert Ar atmosphere in THF containing complex under study (0.14 mM) and supporting electrolyte [n-$Bu_4$N][$PF_6$] (0.13 mM). Data were recorded with Autolab NOVA software (v. 1.11). Elemental analyses were performed by the University of Manchester. UV-visible absorption spectra were recorded using a Perkin-Elmer Lambda 35 UV/vis spectrometer. Mass spectrometry data was obtained using APCI(ASAP) (Atmospheric Solids Analysis Probe) at the University of Manchester.

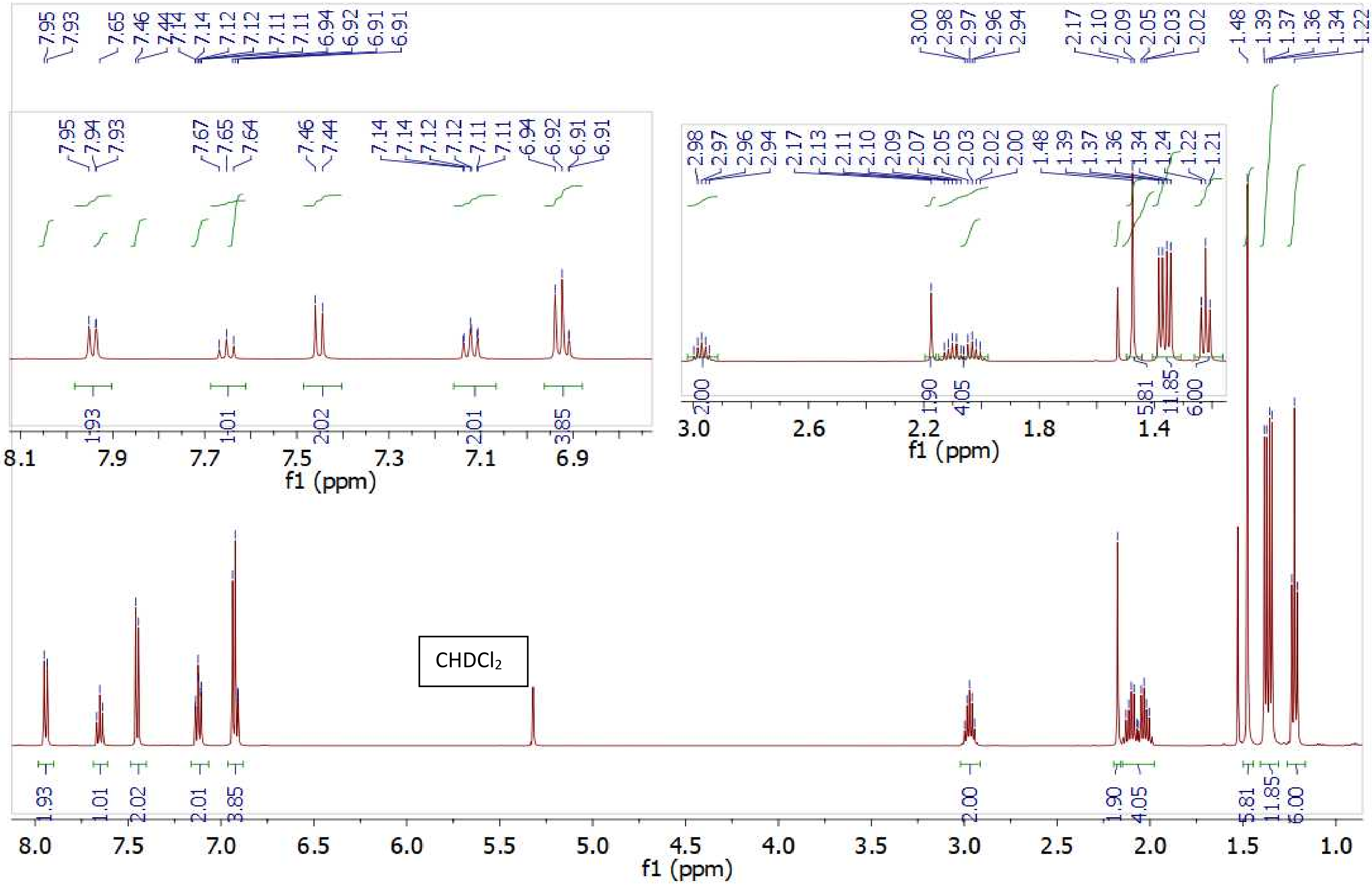

**Figure S3.** $^1$H NMR (500 MHz, $CD_2Cl_2$) for **e-2-1**

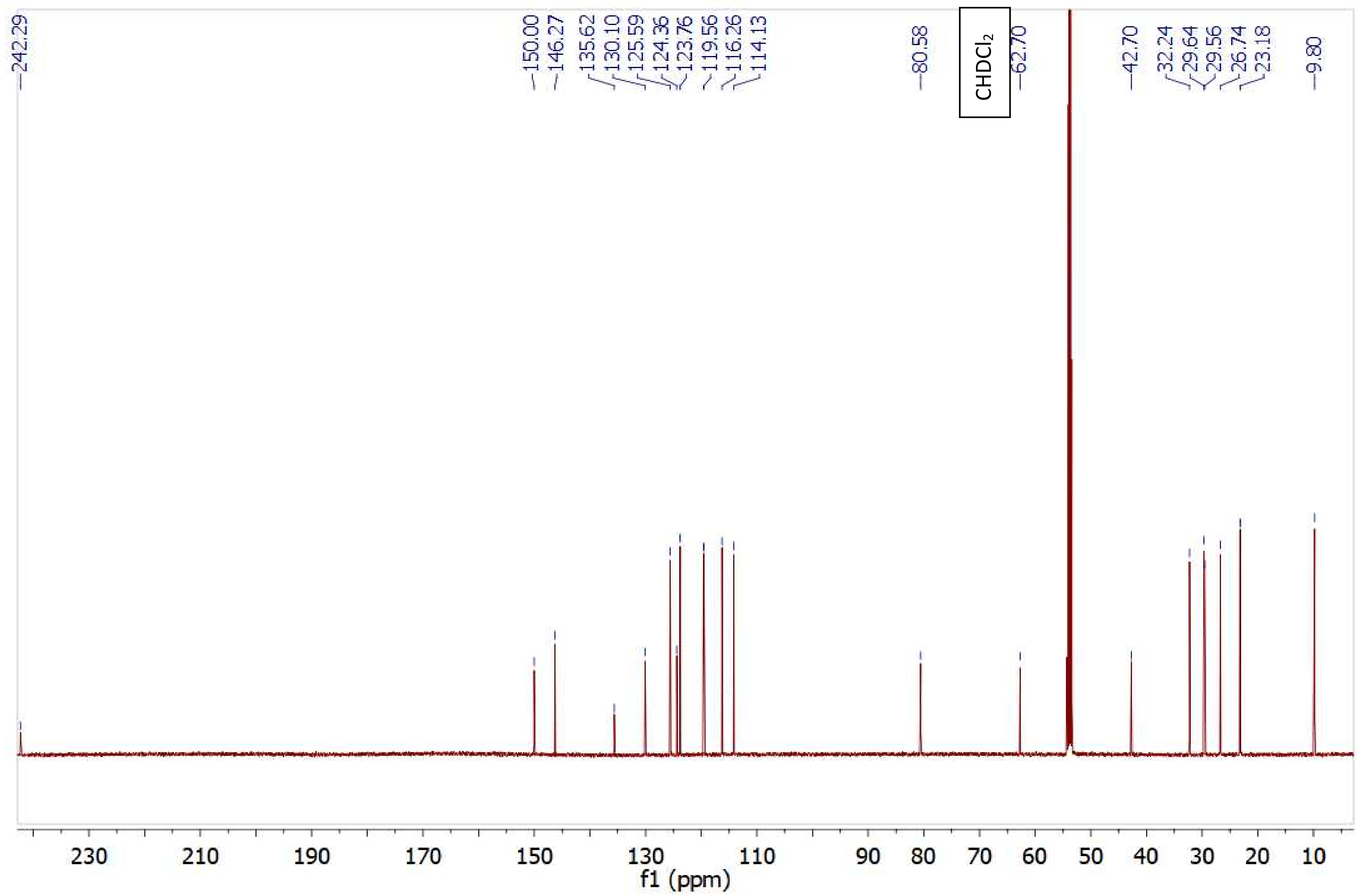


**Figure S4.** $^{13}C$ NMR (125 MHz, $CD_2Cl_2$) for **e-2-1**

**Table S4.** UV-vis data for **e-2-1** in various solvents

| | $\lambda_{abs}$ [nm], ($10^3$ $\varepsilon$/$M^{-1}$ $cm^{-1}$) | | | |
|---|---|---|---|---|
| | DCM | THF | Toluene | MCH |
| **e-2-1** | 387 (sh) (4.4)<br>367 (11.3) | 379 (8.0)<br>367 (12.5) | 392 (8.2)<br>367 (10.1) | 408 (8.4)<br>365 (7.3) |

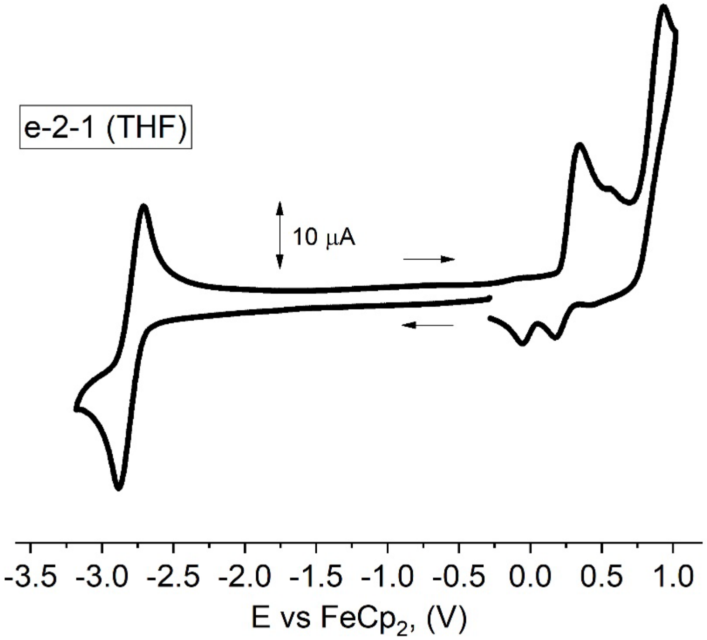


**Figure S5**. Full range cyclic voltammogram for **e-2-1**. Recorded using a glassy carbon electrode in THF solution (1.4 mM) with [n-$Bu_4N$]$PF_6$ as supporting electrolyte (0.13 M), scan rate 0.1 $Vs^{-1}$.

**Table S5.** Formal electrode potentials (peak position $E_p$ for irreversible and $E_{1/2}$ for quasi-reversible processes (*), *V*, *vs*. $FeCp_2$), onset potentials (*E*, *V*, *vs*. $FeCp_2$), peak-to-peak separation in parentheses for quasi-reversible processes ($\Delta E_p$ in mV), $E_{HOMO}/E_{LUMO}$ (*e*V) and band gap values ($\Delta E$, *e*V) for the redox changes exhibited.[a]

| | Reduction | | $E_{LUMO}$ (eV) | Oxidation | | | | $E_{HOMO}$ (eV) | $\Delta E$ (eV) |
|---|---|---|---|---|---|---|---|---|---|
| | $E_{1st}$ | $E_{onset\ red}$ | | $E_{1st}$ | $E_{onset\ ox}$ | $E_{2nd}$ | $E_{3rd}$ | | |
| **e-2-1** | -2.79 (176) | -2.72 | -2.67 | 0.15 (397) | 0.22 | 0.37 (384) | 0.93 | -5.61 | 2.94 |

[a] In tetrahydrofuran solution, recorded using a glassy carbon electrode, concentration 1.4 m*M*, supporting electrolyte $[^nBu_4N][PF_6]$ (0.13 *M*), measured at 0.1 $Vs^{-1}$.
[b] In difluorobenzene solution, recorded using a glassy carbon electrode, concentration 1.4 m*M*, supporting electrolyte $[^nBu_4N][PF_6]$ (0.13 *M*), measured at 0.1 $Vs^{-1}$. $E_{HOMO} = -(E_{onset\ ox\ Fc/Fc+} + 5.39)$ *e*V; $E_{LUMO} = -(E_{onset\ red\ Fc/Fc+} + 5.39)$ eV [3].

**Photophysical Characterization**

UV-visible absorption spectra were recorded using a Varian Cary 5000 UV-Vis-NIR spectrometer. Photoluminescence measurements were recorded on an Edinburgh Instruments FLS980 spectrometer with a solids mount attachment where appropriate. Absolute photoluminescence quantum yields were recorded using a Hamamatsu Quantaurus-QY C11347-11. Quantum yields have been measured in air for solid samples and under nitrogen for solutions. Time resolved luminescence data were collected on a time-correlated single photon counting (TCSPC) Edinburgh Instruments FLS980 spectrometer using F-900 software. A xenon flash lamp and EPL pulsed diode lasers were used as excitation sources. The collected data were analyzed using F-900 software.

**Coordinates of optimized structures in zyz-format**

56
a-1-1

| | | | |
|---|---|---|---|
| Au | -0.02661400 | -0.61993800 | -0.07452500 |
| N | -1.98046200 | -0.20384200 | -0.05935200 |
| C | -4.27500500 | -0.31421500 | 0.03955300 |
| C | 2.06245200 | 1.95972800 | 1.25555300 |
| N | 2.79966300 | 0.05385200 | -0.06958700 |
| C | 4.23441200 | -0.35346500 | -0.00916100 |
| C | 1.65735300 | 3.28433300 | 1.34654800 |
| H | 1.37056600 | 3.69387900 | 2.30594700 |
| C | -4.30423700 | -3.08933000 | 0.09946400 |
| H | -4.33587700 | -4.17220700 | 0.12315500 |

| | | | |
|---|---|---|---|
| C | 1.92175300 | -0.91806600 | -0.08267200 |
| C | -2.47960200 | 1.08233600 | -0.06651700 |
| C | 2.34101800 | 2.21068600 | -1.13125300 |
| C | 1.59808900 | 4.07311500 | 0.20329200 |
| C | -3.06647900 | -1.05042800 | 0.00404800 |
| C | 2.40864700 | 1.43458900 | 0.01702500 |
| C | -4.59221800 | 2.28258300 | 0.00093000 |
| H | -5.67570300 | 2.28823800 | 0.04799100 |
| C | -1.77042300 | 2.28404200 | -0.12639400 |
| H | -0.68550900 | 2.27736300 | -0.18244800 |
| C | -3.89206900 | 1.07603300 | -0.00529200 |
| C | -3.08090900 | -2.44621300 | 0.03435000 |
| H | -2.14971300 | -3.00298700 | 0.00626700 |
| C | 4.11980700 | -1.86016500 | -0.27950500 |
| H | 4.79996500 | -2.42995400 | 0.35627800 |
| H | 4.38734400 | -2.06203700 | -1.31998300 |
| C | -5.49685300 | -0.98335000 | 0.10527800 |
| H | -6.42674500 | -0.42591600 | 0.13266000 |
| C | -2.48489300 | 3.46883700 | -0.11742600 |
| H | -1.95068100 | 4.41174600 | -0.16378800 |
| C | 1.93367600 | 3.53549800 | -1.03343700 |
| H | 1.86415500 | 4.14212500 | -1.92671000 |
| C | -5.50712300 | -2.36769300 | 0.13495300 |
| H | -6.44802800 | -2.90125800 | 0.18565900 |
| C | 4.79197100 | -0.02738300 | 1.37141100 |
| H | 4.76171300 | 1.04839700 | 1.55551200 |
| H | 5.83143300 | -0.35389100 | 1.42803700 |
| H | 4.22709700 | -0.53191000 | 2.15664100 |
| C | -3.88695800 | 3.47375700 | -0.05347200 |
| H | -4.41833400 | 4.41732500 | -0.04911800 |
| C | 5.04254600 | 0.36673400 | -1.07698100 |
| H | 4.61503200 | 0.19895600 | -2.06723700 |
| H | 6.06225900 | -0.02185200 | -1.07246400 |
| H | 5.08535800 | 1.44057200 | -0.88601100 |

| | | | |
|---|---|---|---|
| C | 2.64657700 | -2.23877200 | -0.04363600 |
| C | 2.40354300 | -2.84680900 | 1.34214600 |
| H | 1.33697100 | -3.02165800 | 1.49434000 |
| H | 2.93143300 | -3.80019300 | 1.41652600 |
| H | 2.75621300 | -2.19262800 | 2.14174700 |
| C | 2.10493200 | -3.18519400 | -1.10950800 |
| H | 2.66276000 | -4.12436200 | -1.08867000 |
| H | 1.05026800 | -3.39935400 | -0.92530200 |
| H | 2.19467900 | -2.74998300 | -2.10662500 |
| H | 2.08791100 | 1.31754700 | 2.12809800 |
| H | 1.27123500 | 5.10247100 | 0.27398000 |
| H | 2.58223300 | 1.76947800 | -2.09022200 |

60

b-1-1

| | | | |
|---|---|---|---|
| Au | -0.16753900 | -0.71498500 | -0.04359200 |
| N | -2.09996300 | -0.21758400 | -0.02712100 |
| C | -4.39848000 | -0.22055000 | 0.00791000 |
| C | 2.02117600 | 1.88132900 | 1.22848400 |
| N | 2.65563700 | -0.10284500 | -0.06589300 |
| C | 4.09336200 | -0.52732700 | -0.04977600 |
| C | 1.74960200 | 3.25007100 | 1.26878500 |
| H | 1.56430700 | 3.72556100 | 2.22399000 |
| C | -4.56160200 | -2.99153100 | -0.02941700 |
| H | -4.64535600 | -4.07174500 | -0.04407900 |
| C | 1.77272800 | -1.06937800 | -0.05746100 |
| C | -2.53740600 | 1.09239300 | -0.00215300 |
| C | 2.08750700 | 1.98983700 | -1.20862000 |
| C | -3.22634300 | -1.01312100 | -0.02135700 |
| C | 2.27919700 | 1.28477000 | -0.01330900 |
| C | -4.59263100 | 2.38836700 | 0.04920700 |
| H | -5.67555200 | 2.44385800 | 0.06711600 |
| C | -1.77044800 | 2.25962400 | 0.00157100 |
| H | -0.68512300 | 2.20829000 | -0.01873100 |
| C | -3.94922300 | 1.15076800 | 0.02083500 |

| | | | |
|---|---|---|---|
| C | -3.30762100 | -2.40658800 | -0.04020200 |
| H | -2.40366600 | -3.00678000 | -0.06321100 |
| C | 3.95095500 | -2.03772200 | -0.28715500 |
| H | 4.64582900 | -2.60442200 | 0.33532700 |
| H | 4.17828200 | -2.26320200 | -1.33244300 |
| C | -5.65206400 | -0.83132500 | 0.01837000 |
| H | -6.55473800 | -0.23067800 | 0.04083400 |
| C | -2.42931600 | 3.47591600 | 0.03051900 |
| H | -1.84890600 | 4.39212800 | 0.03408200 |
| C | 1.81289600 | 3.35654500 | -1.14437500 |
| H | 1.67808400 | 3.91461100 | -2.06265200 |
| C | -5.72900700 | -2.21386700 | -0.00027700 |
| H | -6.69527900 | -2.70249600 | 0.00745600 |
| C | 4.72307100 | -0.18214300 | 1.29414500 |
| H | 4.67569200 | 0.89291400 | 1.47490100 |
| H | 5.77087700 | -0.48670400 | 1.27715900 |
| H | 4.22505800 | -0.68715900 | 2.12087400 |
| C | -3.83097800 | 3.54542500 | 0.05442100 |
| H | -4.31759300 | 4.51259900 | 0.07653800 |
| C | 4.86972600 | 0.16658500 | -1.15782600 |
| H | 4.44918500 | -0.05377500 | -2.13789800 |
| H | 5.90365700 | -0.18171500 | -1.12911800 |
| H | 4.86655900 | 1.24920300 | -1.01570600 |
| C | 2.48337600 | -2.39533300 | 0.00714200 |
| C | 2.27781700 | -2.93530300 | 1.42742800 |
| H | 1.21468800 | -3.08780400 | 1.62379000 |
| H | 2.79515400 | -3.89158000 | 1.53134800 |
| H | 2.67112700 | -2.24994700 | 2.18189900 |
| C | 1.89964600 | -3.38090000 | -0.99791600 |
| H | 2.44583200 | -4.32585600 | -0.95269400 |
| H | 0.84781600 | -3.57275700 | -0.77556400 |
| H | 1.96735000 | -2.99218000 | -2.01624600 |
| N | 2.04779900 | 1.11933300 | 2.38971300 |
| H | 1.70319400 | 0.17138500 | 2.27806600 |

| | | | |
|---|---|---|---|
| H | 1.62419200 | 1.57558200 | 3.18646100 |
| N | 2.17901100 | 1.32951000 | -2.42668300 |
| H | 1.79319200 | 1.84656000 | -3.20497300 |
| H | 1.85014000 | 0.37069000 | -2.41188800 |
| C | 1.67404000 | 3.97308100 | 0.08913600 |
| H | 1.45432300 | 5.03260800 | 0.13034200 |

58

c-1-1

| | | | |
|---|---|---|---|
| Au | -0.16255800 | -0.67805100 | -0.08318600 |
| N | -2.10850500 | -0.20929500 | -0.06293800 |
| C | -4.40623100 | -0.26826300 | 0.01722000 |
| C | 2.01455100 | 1.82067400 | 1.26010600 |
| N | 2.69003600 | -0.10650900 | -0.07684300 |
| C | 4.11089000 | -0.56691900 | -0.03166900 |
| C | 1.61787000 | 3.14954200 | 1.36211700 |
| H | 1.37045300 | 3.56268300 | 2.33256000 |
| C | -4.49926600 | -3.04249200 | 0.00873900 |
| H | -4.55593400 | -4.12461800 | 0.00548100 |
| C | 1.77538100 | -1.04280700 | -0.08569600 |
| C | -2.57770100 | 1.08635000 | -0.04152800 |
| C | 2.19105600 | 2.05162800 | -1.13447500 |
| C | -3.21412200 | -1.03102800 | -0.02771800 |
| C | 2.33672300 | 1.27560300 | 0.01539100 |
| C | -4.66127900 | 2.33610700 | 0.04027700 |
| H | -5.74469000 | 2.36724700 | 0.07933500 |
| C | -1.83986100 | 2.27197200 | -0.06460100 |
| H | -0.75520900 | 2.23839200 | -0.10989100 |
| C | -3.99090200 | 1.11297900 | 0.00924100 |
| C | -3.26111100 | -2.42677500 | -0.03195000 |
| H | -2.34266900 | -3.00391900 | -0.06734500 |
| C | 3.93225000 | -2.07281200 | -0.26992800 |
| H | 4.59642600 | -2.65690600 | 0.36988900 |
| H | 4.17597100 | -2.30665800 | -1.30938200 |
| C | -5.64373600 | -0.91051400 | 0.05777200 |

| | | | |
|---|---|---|---|
| H | -6.56086700 | -0.33260700 | 0.09210400 |
| C | -2.52500000 | 3.47350800 | -0.03163000 |
| H | -1.96753700 | 4.40414200 | -0.04927200 |
| C | 1.79485500 | 3.38170700 | -1.03452500 |
| H | 1.68631800 | 3.97683500 | -1.93352400 |
| C | -5.68588000 | -2.29438600 | 0.05340000 |
| H | -6.63905800 | -2.80731300 | 0.08424800 |
| C | 4.71941200 | -0.23757700 | 1.32593100 |
| H | 4.71145800 | 0.84092700 | 1.49815000 |
| H | 5.75581500 | -0.57881600 | 1.34019500 |
| H | 4.17627800 | -0.71776300 | 2.13825800 |
| C | -3.92729600 | 3.51107700 | 0.02094200 |
| H | -4.43601600 | 4.46680200 | 0.04477900 |
| C | 4.92197900 | 0.10271700 | -1.12955100 |
| H | 4.49495600 | -0.10052000 | -2.11042100 |
| H | 5.94268300 | -0.28237900 | -1.09757200 |
| H | 4.96089000 | 1.18454700 | -0.98138700 |
| C | 2.44896600 | -2.38680600 | -0.00753100 |
| C | 2.19641100 | -2.90627200 | 1.41358200 |
| H | 1.12785100 | -3.06440400 | 1.57075300 |
| H | 2.71842900 | -3.85613900 | 1.55003700 |
| H | 2.54531100 | -2.19921700 | 2.16868000 |
| C | 1.86302900 | -3.36434300 | -1.01860500 |
| H | 2.38163900 | -4.32361300 | -0.95067800 |
| H | 0.80163700 | -3.52558700 | -0.81832400 |
| H | 1.96432500 | -2.98575000 | -2.03732400 |
| C | 1.51786600 | 3.91901100 | 0.21310600 |
| H | 1.19839100 | 4.95023900 | 0.28946400 |
| O | 2.42253500 | 1.44839800 | -2.32224000 |
| H | 2.15648800 | 2.02769200 | -3.04702200 |
| O | 2.08808600 | 0.99704300 | 2.33102800 |
| H | 1.66990800 | 1.40856700 | 3.09757200 |

64

d-1-1

| | | | |
|---|---|---|---|
| Au | -0.26373000 | -0.74333000 | -0.28800600 |
| N | -2.18706900 | -0.18298000 | -0.31705200 |
| C | -4.47428300 | -0.13940300 | -0.07551200 |
| C | 1.69549300 | 1.53284200 | 1.37597600 |
| N | 2.59475500 | -0.31448300 | 0.07025100 |
| C | 3.95183000 | -0.85939700 | 0.38163000 |
| C | 1.35235000 | 2.87660700 | 1.50807500 |
| H | 0.88247700 | 3.23205500 | 2.41405800 |
| C | -4.71271100 | -2.89750100 | -0.27554900 |
| H | -4.82620400 | -3.97236400 | -0.35283700 |
| C | 1.64985700 | -1.19949500 | -0.11742500 |
| C | -2.58572400 | 1.12807600 | -0.16701900 |
| C | 2.47986600 | 1.94959400 | -0.88010100 |
| C | 1.58990500 | 3.73902800 | 0.45086200 |
| C | -3.32957000 | -0.95198600 | -0.26064800 |
| C | 2.29520100 | 1.07825000 | 0.19794600 |
| C | -4.58904700 | 2.46368900 | 0.16885200 |
| H | -5.66375900 | 2.54276500 | 0.29123900 |
| C | -1.79002500 | 2.27636600 | -0.16170100 |
| H | -0.71708900 | 2.19885900 | -0.31243100 |
| C | -3.98865200 | 1.21707100 | -0.00924500 |
| C | -3.44953000 | -2.33985000 | -0.36021200 |
| H | -2.56718100 | -2.95517700 | -0.50369200 |
| C | 3.75252200 | -2.34002200 | 0.03429400 |
| H | 4.26193600 | -2.98714200 | 0.75076200 |
| H | 4.17135200 | -2.53946000 | -0.95550300 |
| C | -5.73827800 | -0.72351200 | 0.00698000 |
| H | -6.61945800 | -0.10711300 | 0.14777500 |
| C | -2.40612300 | 3.50176800 | 0.02318500 |
| H | -1.80303300 | 4.40353900 | 0.02960100 |
| C | 2.13714900 | 3.29401500 | -0.74549800 |
| H | 2.27686600 | 3.98376600 | -1.56470900 |
| C | -5.85292100 | -2.09952600 | -0.09352600 |
| H | -6.82740600 | -2.56772600 | -0.03250300 |

| | | | |
|---|---|---|---|
| C | 4.26509700 | -0.62184900 | 1.85463300 |
| H | 4.26615600 | 0.44735000 | 2.07757100 |
| H | 5.25743200 | -1.01922700 | 2.07412000 |
| H | 3.53695400 | -1.10374000 | 2.50452000 |
| C | -3.79639300 | 3.60007300 | 0.18960400 |
| H | -4.25048800 | 4.57332900 | 0.32847300 |
| C | 5.01894200 | -0.19974400 | -0.47576900 |
| H | 4.81126800 | -0.33837100 | -1.53546000 |
| H | 5.98454900 | -0.65039200 | -0.23930300 |
| H | 5.08141200 | 0.87055700 | -0.26633100 |
| C | 2.23346300 | -2.58193700 | 0.00709600 |
| C | 1.69796400 | -3.15889900 | 1.32400700 |
| H | 0.61476900 | -3.28249300 | 1.26556900 |
| H | 2.15558800 | -4.13403300 | 1.50590600 |
| H | 1.91452200 | -2.50339500 | 2.16978800 |
| C | 1.79721700 | -3.47306700 | -1.14966000 |
| H | 2.24047400 | -4.46578900 | -1.04106400 |
| H | 0.70993000 | -3.57458700 | -1.15932900 |
| H | 2.11055000 | -3.05623000 | -2.10859300 |
| H | 1.31934700 | 4.78272400 | 0.54887500 |
| O | 1.45362000 | 0.60918700 | 2.32963100 |
| O | 2.95416600 | 1.40081900 | -2.01638500 |
| C | 0.30121300 | 0.80949600 | 3.13557200 |
| H | -0.54671200 | 1.11361200 | 2.51414700 |
| H | 0.08255200 | -0.14635600 | 3.60491300 |
| H | 0.48312800 | 1.55869200 | 3.90928500 |
| C | 3.03068800 | 2.21633100 | -3.16585600 |
| H | 3.37511300 | 1.57799100 | -3.97455700 |
| H | 2.04987500 | 2.62611100 | -3.41895000 |
| H | 3.74065300 | 3.03538200 | -3.02314200 |

74

e-1-1

| | | | |
|---|---|---|---|
| Au | -0.39371800 | -0.88896000 | -0.04159300 |
| N | -2.28825700 | -0.24781800 | -0.02118100 |

| | | | |
|---|---|---|---|
| C | -4.57717000 | -0.03395800 | -0.00517000 |
| C | 1.97420800 | 1.59881500 | 1.27429900 |
| N | 2.45334700 | -0.41214700 | -0.05946800 |
| C | 3.86652200 | -0.91234100 | -0.08232500 |
| C | 1.78469400 | 2.97926500 | 1.31352100 |
| H | 1.64418300 | 3.46199900 | 2.27342900 |
| C | -4.99894300 | -2.77663100 | -0.08302400 |
| H | -5.18369600 | -3.84388900 | -0.11357600 |
| C | 1.52529200 | -1.33362100 | -0.06723300 |
| C | -2.60045600 | 1.09640700 | 0.01795600 |
| C | 0.39824500 | 0.94516500 | 3.08185700 |
| H | -0.32910000 | 0.62368900 | 2.33366600 |
| H | 0.17582700 | 1.98401000 | 3.33785200 |
| H | 0.26417700 | 0.33907000 | 3.97996600 |
| C | 2.00497000 | 1.72636600 | -1.17414000 |
| C | 1.73262100 | 3.73299100 | 0.15288900 |
| H | 1.58484400 | 4.80420000 | 0.20677700 |
| C | -3.48432600 | -0.93302900 | -0.03596000 |
| C | 2.15085200 | 1.00001000 | 0.01706700 |
| C | 0.47678300 | 1.21074400 | -3.06233900 |
| H | -0.24477700 | 0.77069100 | -2.37039800 |
| H | 0.37784200 | 0.71568800 | -4.03039700 |
| H | 0.21527700 | 2.26410800 | -3.19037600 |
| C | -4.52375100 | 2.58121900 | 0.06936000 |
| H | -5.59660700 | 2.73942700 | 0.07899800 |
| C | -1.72641400 | 2.18673600 | 0.04368300 |
| H | -0.65063900 | 2.03290200 | 0.03265700 |
| C | 1.83660600 | 0.81985200 | 2.56845600 |
| H | 2.02257500 | -0.23712400 | 2.36258600 |
| C | 1.91131400 | 1.07903100 | -2.54159600 |
| H | 2.12891400 | 0.01243800 | -2.44087900 |
| C | -4.00112400 | 1.28857800 | 0.03014400 |
| C | -3.69558700 | -2.31230300 | -0.07514800 |
| H | -2.85155500 | -2.99378000 | -0.09918300 |

| | | | |
|---|---|---|---|
| C | 3.64569200 | -2.40496900 | -0.36415300 |
| H | 4.31878600 | -3.02546600 | 0.23048600 |
| H | 3.84879900 | -2.60756200 | -1.41915600 |
| C | -5.88276900 | -0.52442300 | -0.01359000 |
| H | -6.72537200 | 0.15792900 | 0.00989900 |
| C | 2.80172400 | 1.29244700 | 3.65477300 |
| H | 3.84431100 | 1.25477400 | 3.33803100 |
| H | 2.69099100 | 0.67336700 | 4.54704500 |
| H | 2.57723700 | 2.32160800 | 3.94361600 |
| C | -2.26738700 | 3.46015900 | 0.08276300 |
| H | -1.60243600 | 4.31698200 | 0.10330800 |
| C | 1.81098400 | 3.10255300 | -1.07818000 |
| H | 1.69101700 | 3.68111100 | -1.98653000 |
| C | -6.08864700 | -1.89312400 | -0.05241300 |
| H | -7.09653700 | -2.28890500 | -0.05951300 |
| C | 2.88410500 | 1.69080100 | -3.54855900 |
| H | 3.91373500 | 1.68301000 | -3.18808600 |
| H | 2.61301800 | 2.72699500 | -3.76208300 |
| H | 2.84251000 | 1.14329900 | -4.49202000 |
| C | 4.54286500 | -0.64212700 | 1.25487700 |
| H | 4.57765200 | 0.43164400 | 1.44810700 |
| H | 5.56801600 | -1.01320100 | 1.21308000 |
| H | 4.03296000 | -1.13364800 | 2.08362700 |
| C | -3.65606900 | 3.66131500 | 0.09577700 |
| H | -4.04954400 | 4.66973900 | 0.12636200 |
| C | 4.67155800 | -0.23335200 | -1.17926100 |
| H | 4.24882800 | -0.42852600 | -2.16449900 |
| H | 5.68827200 | -0.62959400 | -1.16051100 |
| H | 4.72248800 | 0.84603800 | -1.01988500 |
| C | 2.16618200 | -2.69573200 | -0.06250200 |
| C | 1.95082700 | -3.28935900 | 1.33375400 |
| C | 1.52031300 | -3.59985400 | -1.10595600 |
| H | 0.88368300 | -3.37911400 | 1.54519700 |
| H | 2.39993400 | -2.66782600 | 2.11156100 |

| | | | |
|---|---|---|---|
| H | 0.46496400 | -3.75500700 | -0.87304800 |
| H | 1.58700800 | -3.15801700 | -2.10273600 |
| H | 2.40443000 | -4.28192300 | 1.38039700 |
| H | 2.02378200 | -4.56933000 | -1.12111300 |

80

e-2-1

| | | | |
|---|---|---|---|
| Au | -0.41221100 | -0.74941600 | 0.02092500 |
| N | -2.36243500 | -0.30240300 | 0.01483000 |
| C | -4.66107200 | -0.31763800 | 0.00712800 |
| C | 1.74893300 | 2.10146200 | 1.11043500 |
| N | 2.37735000 | 0.00440300 | 0.00235200 |
| C | 3.83291400 | -0.35220500 | -0.00529100 |
| C | 1.40840900 | 3.44849000 | 1.01018400 |
| H | 1.25409700 | 4.02045000 | 1.91754200 |
| C | -4.80699700 | -3.08940600 | 0.05896200 |
| H | -4.88441300 | -4.17002800 | 0.07930700 |
| C | 1.54502900 | -1.00233200 | 0.03126900 |
| C | -2.80703000 | 1.00410300 | -0.01062000 |
| C | 0.33786000 | 1.40776800 | 3.03332000 |
| H | -0.33644100 | 0.88059800 | 2.35396600 |
| H | -0.05418000 | 2.41880700 | 3.17086600 |
| H | 0.32318700 | 0.90184400 | 4.00124300 |
| C | 1.66133200 | 1.94344900 | -1.33459900 |
| C | 1.22959600 | 4.05346000 | -0.22313500 |
| H | 0.96841600 | 5.10255800 | -0.28175100 |
| C | -3.48379200 | -1.10356700 | 0.02614600 |
| C | 1.93392300 | 1.37971500 | -0.07819600 |
| C | 0.12265900 | 1.12898100 | -3.11123100 |
| H | -0.56029200 | 0.75046500 | -2.34797500 |
| H | 0.03208100 | 0.50177100 | -4.00024100 |
| H | -0.19969800 | 2.13906400 | -3.37562100 |
| C | -4.86892900 | 2.29039600 | -0.04315200 |
| H | -5.95224600 | 2.34099100 | -0.04828600 |
| C | -2.04601500 | 2.17630200 | -0.02899000 |

| | | | |
|---|---|---|---|
| H | -0.96023200 | 2.13060000 | -0.02264400 |
| C | 1.76580000 | 1.45864300 | 2.48228900 |
| H | 2.11307800 | 0.42908900 | 2.37969800 |
| C | 1.57438900 | 1.14395800 | -2.62079200 |
| H | 1.86215900 | 0.11036200 | -2.41218400 |
| C | -4.22000000 | 1.05592600 | -0.01718400 |
| C | -3.55642700 | -2.49746400 | 0.05217500 |
| H | -2.64826300 | -3.09114900 | 0.06697700 |
| C | 3.78129000 | -1.87761000 | 0.17941900 |
| H | 4.16968300 | -2.14784400 | 1.16329200 |
| H | 4.41039700 | -2.37184000 | -0.56361000 |
| C | -5.91117800 | -0.93595200 | 0.01433600 |
| H | -6.81769600 | -0.34083300 | -0.00012600 |
| C | 2.67718300 | 2.18724200 | 3.46781300 |
| H | 3.69611800 | 2.28906500 | 3.09132900 |
| H | 2.71162600 | 1.64656300 | 4.41557400 |
| H | 2.29617300 | 3.18872600 | 3.67899000 |
| C | -2.71133600 | 3.38969300 | -0.05532700 |
| H | -2.13506200 | 4.30861700 | -0.07029300 |
| C | 1.32887700 | 3.29645900 | -1.37862900 |
| H | 1.11326300 | 3.75195400 | -2.33794700 |
| C | -5.97948900 | -2.31868000 | 0.04014600 |
| H | -6.94285700 | -2.81307300 | 0.04614900 |
| C | 2.47283600 | 1.69784000 | -3.72590600 |
| H | 3.51961600 | 1.75772400 | -3.42713900 |
| H | 2.14917000 | 2.70061500 | -4.01362500 |
| H | 2.40401300 | 1.06673600 | -4.61401400 |
| C | 4.57396000 | 0.34351600 | 1.12575300 |
| H | 4.49399900 | 1.42918000 | 1.03739500 |
| H | 5.62964600 | 0.07311700 | 1.06803400 |
| H | 4.19675200 | 0.03630300 | 2.10201300 |
| C | -4.11324500 | 3.45172000 | -0.06243100 |
| H | -4.60527900 | 4.41617300 | -0.08284700 |
| C | 4.45930800 | 0.05647700 | -1.33104100 |

| | | | |
|---|---|---|---|
| H | 3.98457200 | -0.44864600 | -2.17365500 |
| H | 5.51653700 | -0.21293800 | -1.31994000 |
| H | 4.38291100 | 1.13610300 | -1.47422000 |
| C | 2.30677300 | -2.29910500 | 0.03521200 |
| C | 1.81867400 | -3.18881600 | 1.18633000 |
| C | 2.03088700 | -2.96734000 | -1.32719600 |
| H | 0.81850400 | -3.55583500 | 0.92986900 |
| H | 0.94694200 | -3.00749000 | -1.47322200 |
| H | 2.42631300 | -2.31333800 | -2.11419100 |
| H | 2.47761200 | -4.06012400 | 1.24324600 |
| C | 2.62666100 | -4.36168000 | -1.45970400 |
| H | 2.51695900 | -4.72825800 | -2.48020800 |
| H | 3.69183000 | -4.37244500 | -1.21560300 |
| H | 2.12569500 | -5.07048200 | -0.79987100 |
| C | 1.77083400 | -2.47860900 | 2.53307300 |
| H | 1.53966200 | -3.17737200 | 3.33674400 |
| H | 2.72819300 | -2.00585400 | 2.77470200 |
| H | 1.00197900 | -1.70125000 | 2.53550900 |

89

e-3-1

| | | | |
|---|---|---|---|
| Au | 0.39528100 | 0.44852800 | -0.13882000 |
| N | 2.36117200 | 0.79909400 | -0.06890800 |
| C | 4.44137100 | 1.77553900 | -0.02073900 |
| C | -0.45722700 | -2.71329800 | 1.38024100 |
| N | -1.85029900 | -1.31410300 | -0.08876400 |
| C | -2.75736800 | 1.88574400 | -1.33438200 |
| H | -2.57529600 | 1.39659300 | -2.29908300 |
| C | -1.92718100 | 3.62284700 | 0.26443000 |
| H | -1.13754400 | 4.35612400 | 0.45144000 |
| C | -3.31265100 | -1.61532300 | -0.11982000 |
| C | 0.37755500 | -3.82109300 | 1.51911600 |
| H | 0.71859400 | -4.09929000 | 2.50934100 |
| C | -2.99503700 | 1.51647600 | 1.12060700 |
| H | -2.97340300 | 0.77460000 | 1.92709700 |

| | | | |
|---|---|---|---|
| C | 3.41498200 | 4.35266700 | -0.15352400 |
| H | 3.03389600 | 5.36581600 | -0.20260000 |
| C | -1.52284800 | -0.04732900 | -0.17517500 |
| C | 3.30830300 | -0.19983200 | 0.01593000 |
| C | -1.69102300 | 2.95222700 | -1.08703800 |
| H | -1.75642500 | 3.69951900 | -1.88550700 |
| H | -0.68728100 | 2.52160300 | -1.13289900 |
| C | 0.57478100 | -1.24670100 | 3.10113000 |
| H | 1.07995100 | -0.68636700 | 2.31185100 |
| H | 1.25835600 | -2.02675500 | 3.44538000 |
| H | 0.38190300 | -0.57291500 | 3.93837300 |
| C | -0.38210800 | -3.02923900 | -1.05002700 |
| C | 0.80702300 | -4.54335000 | 0.41834200 |
| H | 1.45397000 | -5.40146900 | 0.54993400 |
| C | -1.90830900 | 2.56860200 | 1.37174900 |
| H | -2.10796000 | 3.04603700 | 2.33713500 |
| H | -0.91870800 | 2.10772800 | 1.43715000 |
| C | -3.28845000 | 4.31574500 | 0.24125900 |
| H | -3.47221400 | 4.82133200 | 1.19534400 |
| H | -3.30725700 | 5.07583200 | -0.54699200 |
| C | 3.04442500 | 1.99584500 | -0.08628000 |
| C | -0.88127900 | -2.37375300 | 0.08552700 |
| C | -2.78303600 | 0.79402600 | -0.24182300 |
| C | 0.72455600 | -1.96578000 | -3.00203800 |
| H | 1.11133400 | -1.16754600 | -2.36470900 |
| H | 0.58574200 | -1.57089000 | -4.01049000 |
| H | 1.48127700 | -2.75326900 | -3.04549600 |
| C | 5.71566600 | -0.50609100 | 0.13217600 |
| H | 6.72129700 | -0.10074800 | 0.15562200 |
| C | 3.10250500 | -1.58020300 | 0.07576400 |
| H | 2.09538500 | -1.98706500 | 0.05705200 |
| C | -4.12497900 | 2.57946900 | -1.35176000 |
| H | -4.13536000 | 3.32097300 | -2.15690700 |
| H | -4.92645000 | 1.86452700 | -1.56323600 |

| | | | |
|---|---|---|---|
| C | -0.73661500 | -1.86950900 | 2.60966700 |
| H | -1.40747300 | -1.05297400 | 2.32944200 |
| C | -0.59516900 | -2.52799000 | -2.46462200 |
| H | -1.31863600 | -1.70840200 | -2.44163700 |
| C | 4.61306700 | 0.34405700 | 0.04650200 |
| C | 2.52717900 | 3.29117300 | -0.14976900 |
| H | 1.45321800 | 3.45112900 | -0.19106600 |
| C | -3.86347700 | -0.26108000 | -0.55770400 |
| H | -4.82684200 | -0.06343700 | -0.09092900 |
| H | -4.02215900 | -0.27574200 | -1.64158100 |
| C | 5.31725600 | 2.86070000 | -0.02608900 |
| H | 6.38882000 | 2.70122900 | 0.02320900 |
| C | -1.36901700 | -2.66443600 | 3.75137000 |
| H | -2.29698400 | -3.15714400 | 3.46104300 |
| H | -1.58063400 | -2.00235600 | 4.59318700 |
| H | -0.67987000 | -3.43305600 | 4.10821500 |
| C | 4.21112000 | -2.40419600 | 0.16210000 |
| H | 4.06996700 | -3.47862600 | 0.21045600 |
| C | 0.45322900 | -4.12689600 | -0.85473400 |
| H | 0.85265700 | -4.64378600 | -1.71934500 |
| C | 4.80121000 | 4.14413000 | -0.09385100 |
| H | 5.46920500 | 4.99634800 | -0.09880300 |
| C | -1.10859700 | -3.61872500 | -3.40378400 |
| H | -2.00115700 | -4.11312000 | -3.01779600 |
| H | -0.34385900 | -4.38319500 | -3.55715300 |
| H | -1.34221900 | -3.19347400 | -4.38163600 |
| C | -4.34486000 | 2.24164400 | 1.11886200 |
| H | -5.18198300 | 1.54576400 | 1.02525000 |
| H | -4.46435600 | 2.74918300 | 2.08188800 |
| C | -3.79641100 | -2.05339100 | 1.25647000 |
| H | -3.29631200 | -2.97735000 | 1.55209600 |
| H | -4.86874700 | -2.24916000 | 1.20749500 |
| H | -3.61881700 | -1.29552500 | 2.01946800 |
| C | -4.37659900 | 3.27284900 | -0.01027900 |

| | | | |
|---|---|---|---|
| H | -5.35629500 | 3.75826400 | -0.03100500 |
| C | 5.51091700 | -1.87546800 | 0.18892400 |
| H | 6.35876700 | -2.54577100 | 0.25622600 |
| C | -3.63938500 | -2.71532600 | -1.11783700 |
| H | -3.34888600 | -2.43607200 | -2.12994300 |
| H | -4.71815500 | -2.88121500 | -1.10766400 |
| H | -3.14744600 | -3.65292400 | -0.85004900 |

81

e-4-1

| | | | |
|---|---|---|---|
| Au | 0.38344100 | 0.65876400 | -0.06985600 |
| N | 2.37580100 | 0.48175300 | -0.02651100 |
| C | 4.64986100 | 0.81731000 | 0.00620300 |
| C | -1.34465800 | -2.28444500 | 1.29328400 |
| N | -2.27132700 | -0.47218500 | -0.08744400 |
| C | -3.76040300 | -0.32040600 | -0.12601100 |
| C | -0.83653700 | -3.58044600 | 1.36511800 |
| H | -0.59614300 | -3.99630100 | 2.33648000 |
| C | 4.41016800 | 3.57962500 | -0.13014000 |
| H | 4.33705000 | 4.65943600 | -0.18334800 |
| C | -1.58756900 | 0.64263500 | -0.11186300 |
| C | 2.99721600 | -0.74879300 | 0.04465800 |
| C | 0.01639600 | -1.23922900 | 3.09077400 |
| H | 0.65337500 | -0.77130400 | 2.33747200 |
| H | 0.47541800 | -2.19053900 | 3.37103800 |
| H | -0.00384700 | -0.59942700 | 3.97521000 |
| C | -1.31927000 | -2.46695100 | -1.15167700 |
| C | -0.59695400 | -4.32510000 | 0.22226800 |
| H | -0.20225900 | -5.33018600 | 0.30138200 |
| C | 3.37528000 | 1.43057100 | -0.05040900 |
| C | -1.64457400 | -1.77055500 | 0.02185400 |
| C | 0.06364900 | -1.64816700 | -3.04520300 |
| H | 0.65593800 | -1.03623100 | -2.36120500 |
| H | 0.05250600 | -1.16482900 | -4.02413000 |
| H | 0.56572900 | -2.61344400 | -3.14797200 |

| | | | |
|---|---|---|---|
| C | 5.21722200 | -1.73363700 | 0.13956800 |
| H | 6.29684700 | -1.63250500 | 0.15825900 |
| C | 2.40638600 | -2.01460700 | 0.09103700 |
| H | 1.32502100 | -2.12078900 | 0.07138800 |
| C | -1.40643700 | -1.46762800 | 2.56987100 |
| H | -1.83444300 | -0.48889500 | 2.33948500 |
| C | -1.36662400 | -1.84468600 | -2.53308100 |
| H | -1.82833000 | -0.85668300 | -2.45750800 |
| C | 4.40343800 | -0.60302000 | 0.06831300 |
| C | 3.25402200 | 2.81963500 | -0.11904900 |
| H | 2.27259400 | 3.28056300 | -0.16268300 |
| C | -3.90090000 | 1.18020300 | -0.42041700 |
| H | -4.70216500 | 1.61957100 | 0.17614800 |
| H | -4.15988200 | 1.32415000 | -1.47369800 |
| C | 5.80193900 | 1.60323900 | -0.00609500 |
| H | 6.78200200 | 1.14089600 | 0.03716700 |
| C | -2.24238000 | -2.13119600 | 3.66331300 |
| H | -3.26179000 | -2.34672700 | 3.34229000 |
| H | -2.28815200 | -1.48455800 | 4.54165500 |
| H | -1.78440500 | -3.07213000 | 3.97623700 |
| C | 3.23346900 | -3.12214100 | 0.16174600 |
| H | 2.79041300 | -4.11161400 | 0.19865900 |
| C | -0.80824200 | -3.75665800 | -1.02323100 |
| H | -0.54628500 | -4.30972100 | -1.91751900 |
| C | 5.67790600 | 2.98066900 | -0.07412700 |
| H | 6.56324600 | 3.60406400 | -0.08453100 |
| C | -2.16002800 | -2.68808400 | -3.52996000 |
| H | -3.16696100 | -2.91215000 | -3.17500500 |
| H | -1.65322400 | -3.63719300 | -3.71757400 |
| H | -2.23717700 | -2.16663700 | -4.48588400 |
| C | -4.36622100 | -0.72704700 | 1.21038200 |
| H | -4.14551300 | -1.77506800 | 1.42137200 |
| H | -5.44986100 | -0.61176300 | 1.15639700 |
| H | -3.99555900 | -0.11459100 | 2.03293400 |

| | | | |
|---|---|---|---|
| C | 4.63011000 | -2.98791600 | 0.18618200 |
| H | 5.25104500 | -3.87338100 | 0.24166300 |
| C | -4.37180000 | -1.18101000 | -1.22042900 |
| H | -3.99578500 | -0.90231300 | -2.20461700 |
| H | -5.45331600 | -1.03481300 | -1.21494600 |
| H | -4.16918600 | -2.24035600 | -1.04744600 |
| C | -2.52790600 | 1.81447700 | -0.13746700 |
| C | -2.44444200 | 2.49283200 | 1.24365000 |
| C | -2.10700200 | 2.82239100 | -1.21044100 |
| C | -3.27795300 | 3.76773600 | 1.30213900 |
| H | -1.39158400 | 2.73263600 | 1.43348900 |
| H | -2.75431400 | 1.78829100 | 2.02275300 |
| C | -2.97986400 | 4.06930500 | -1.16461500 |
| H | -1.06092300 | 3.09736100 | -1.02951400 |
| H | -2.14555000 | 2.34203400 | -2.19355800 |
| C | -2.86868200 | 4.73740800 | 0.20041400 |
| H | -3.16595600 | 4.22979900 | 2.28504500 |
| H | -4.34133000 | 3.52619400 | 1.19055400 |
| H | -2.68623500 | 4.75914000 | -1.95792800 |
| H | -4.02624500 | 3.79685600 | -1.35296200 |
| H | -3.48070000 | 5.64069000 | 0.24073200 |
| H | -1.82892900 | 5.04523900 | 0.36117400 |

79

e-5-1

| | | | |
|---|---|---|---|
| Au | 0.38751000 | 0.69019300 | -0.05596900 |
| N | 2.37799100 | 0.49662000 | -0.02135900 |
| C | 4.65547500 | 0.80883800 | 0.00081400 |
| C | -1.36723900 | -2.24903700 | 1.28771700 |
| N | -2.27683500 | -0.42233300 | -0.08206900 |
| C | -3.76615600 | -0.25657400 | -0.12187900 |
| C | -0.86945000 | -3.54935600 | 1.35338900 |
| H | -0.63330700 | -3.97226500 | 2.32273700 |
| C | 4.44353000 | 3.57439300 | -0.10985600 |
| H | 4.38113800 | 4.65530800 | -0.15277200 |

| | | | |
|---|---|---|---|
| C | -1.58261300 | 0.68650700 | -0.09277300 |
| C | 2.98735100 | -0.74090400 | 0.03593000 |
| C | 0.00018500 | -1.22524100 | 3.09245500 |
| H | 0.64319700 | -0.76168000 | 2.34152000 |
| H | 0.44903200 | -2.18195200 | 3.37068800 |
| H | -0.01505200 | -0.58798000 | 3.97881700 |
| C | -1.34087100 | -2.41915600 | -1.15832000 |
| C | -0.63452500 | -4.28982400 | 0.20679700 |
| H | -0.24770500 | -5.29833000 | 0.28110000 |
| C | 3.38710600 | 1.43561500 | -0.04293200 |
| C | -1.66169600 | -1.72665300 | 0.01855400 |
| C | 0.05046900 | -1.59958400 | -3.04543600 |
| H | 0.64637500 | -0.99531400 | -2.35776700 |
| H | 0.04394600 | -1.11135900 | -4.02198000 |
| H | 0.54593100 | -2.56781700 | -3.15234800 |
| C | 5.19785800 | -1.74865200 | 0.10863300 |
| H | 6.27852600 | -1.65840900 | 0.12139600 |
| C | 2.38419600 | -2.00110900 | 0.07583300 |
| H | 1.30174500 | -2.09667600 | 0.06273900 |
| C | -1.42389400 | -1.43778400 | 2.56819800 |
| H | -1.84245400 | -0.45364400 | 2.34222000 |
| C | -1.38180300 | -1.78849900 | -2.53604400 |
| H | -1.83621300 | -0.79756200 | -2.45481400 |
| C | 4.39502600 | -0.60943700 | 0.05217900 |
| C | 3.27962900 | 2.82633300 | -0.09865700 |
| H | 2.30297400 | 3.29793000 | -0.13202000 |
| C | -3.88747200 | 1.24545800 | -0.41535700 |
| H | -4.69160500 | 1.70902000 | 0.15976400 |
| H | -4.11313200 | 1.38575800 | -1.47710000 |
| C | 5.81559000 | 1.58284800 | -0.01149000 |
| H | 6.79102900 | 1.11004800 | 0.02196600 |
| C | -2.26823900 | -2.09907500 | 3.65650300 |
| H | -3.28931500 | -2.30184500 | 3.33252100 |
| H | -2.30848300 | -1.45746200 | 4.53879100 |

| | | | |
|---|---|---|---|
| H | -1.82058000 | -3.04668900 | 3.96411300 |
| C | 3.20054200 | -3.11746500 | 0.13175600 |
| H | 2.74772600 | -4.10270100 | 0.16337400 |
| C | -0.84004600 | -3.71343700 | -1.03604500 |
| H | -0.58129800 | -4.26389000 | -1.93286700 |
| C | 5.70532400 | 2.96198700 | -0.06669800 |
| H | 6.59694500 | 3.57636100 | -0.07676600 |
| C | -2.18056800 | -2.61993400 | -3.53864000 |
| H | -3.18951800 | -2.83839800 | -3.18592300 |
| H | -1.68079000 | -3.57174400 | -3.73130000 |
| H | -2.25280800 | -2.09236400 | -4.49158000 |
| C | -4.38098700 | -0.66966700 | 1.20847600 |
| H | -4.17616700 | -1.72375000 | 1.40548900 |
| H | -5.46257500 | -0.53801200 | 1.15283500 |
| H | -4.00496700 | -0.07633500 | 2.04187900 |
| C | 4.59854000 | -2.99740100 | 0.14818300 |
| H | 5.21093000 | -3.88943500 | 0.19212200 |
| C | -4.38164100 | -1.10687200 | -1.22241500 |
| H | -4.00016700 | -0.82750500 | -2.20423800 |
| H | -5.46174700 | -0.95066100 | -1.21945200 |
| H | -4.18941600 | -2.16877600 | -1.05341200 |
| C | -2.51620800 | 1.86206800 | -0.09941600 |
| C | -2.46055300 | 2.50749100 | 1.29447700 |
| C | -2.09016400 | 2.91207400 | -1.12603600 |
| C | -3.15710200 | 3.83619200 | 1.30928600 |
| H | -1.41071600 | 2.61788100 | 1.59345300 |
| H | -2.91585400 | 1.83887200 | 2.03315100 |
| C | -3.10282800 | 4.04873000 | -1.17031800 |
| H | -1.10560700 | 3.29253700 | -0.83501000 |
| H | -1.97877200 | 2.44691800 | -2.11000700 |
| C | -3.46247900 | 4.51643500 | 0.20799700 |
| H | -3.41981100 | 4.24187800 | 2.28040300 |
| H | -2.70052500 | 4.88305000 | -1.74984700 |
| H | -4.01060000 | 3.73407200 | -1.70039300 |

| | | | |
|---|---|---|---|
| H | -3.98745700 | 5.46183800 | 0.29482800 |

82

e-7-1

| | | | |
|---|---|---|---|
| Au | 0.37798500 | 0.62023700 | -0.12339300 |
| N | 2.36543300 | 0.40676500 | -0.06359500 |
| C | 4.64147900 | 0.72984700 | -0.03103700 |
| C | -1.31383000 | -2.20254200 | 1.37826900 |
| N | -2.25822700 | -0.48903000 | -0.11282400 |
| C | -3.74975900 | -0.37817000 | -0.16462800 |
| C | -0.81470400 | -3.49534300 | 1.53099300 |
| H | -0.57128700 | -3.84881000 | 2.52598600 |
| C | 4.41827600 | 3.49318800 | -0.17569100 |
| H | 4.35115600 | 4.57331000 | -0.22963800 |
| C | -1.60186400 | 0.64676300 | -0.16644200 |
| C | 2.97973600 | -0.82594400 | 0.02239700 |
| C | 0.06321800 | -1.06275700 | 3.10536100 |
| H | 0.71001500 | -0.66636700 | 2.32015200 |
| H | 0.50430400 | -1.99781500 | 3.45944700 |
| H | 0.05383200 | -0.35701400 | 3.93841700 |
| C | -1.30747200 | -2.54705700 | -1.04942000 |
| C | -0.58903500 | -4.31595900 | 0.43853600 |
| H | -0.20168300 | -5.31683200 | 0.58166700 |
| C | 3.37032600 | 1.35014400 | -0.09186300 |
| C | -1.61761500 | -1.77061100 | 0.07693400 |
| C | 0.06286700 | -1.82579300 | -2.98933100 |
| H | 0.63907300 | -1.15241300 | -2.35094700 |
| H | 0.04560300 | -1.41736700 | -4.00178500 |
| H | 0.58560200 | -2.78537200 | -3.01716400 |
| C | 5.19319000 | -1.82326200 | 0.12906800 |
| H | 6.27346800 | -1.72905100 | 0.14570000 |
| C | 2.38068200 | -2.08612200 | 0.09066300 |
| H | 1.29898000 | -2.18224200 | 0.07860100 |
| C | -1.36384700 | -1.30680200 | 2.60202700 |
| H | -1.78488800 | -0.33960100 | 2.31357800 |

| | | | |
|---|---|---|---|
| C | -1.36473800 | -2.01509400 | -2.46728800 |
| H | -1.84519300 | -1.03328300 | -2.45339200 |
| C | 4.38665200 | -0.68835800 | 0.04387600 |
| C | 3.25767500 | 2.73977700 | -0.16200500 |
| H | 2.27940200 | 3.20881000 | -0.19963700 |
| C | -3.90340900 | 1.06996100 | -0.62157400 |
| H | -4.79111400 | 1.52709200 | -0.18431900 |
| H | -4.01380100 | 1.09808400 | -1.71103400 |
| C | 5.79801700 | 1.50889200 | -0.04671800 |
| H | 6.77524900 | 1.04088500 | -0.00070200 |
| C | -2.19847900 | -1.89321500 | 3.74009600 |
| H | -3.22298400 | -2.11718800 | 3.44273500 |
| H | -2.22942500 | -1.19339000 | 4.57736300 |
| H | -1.74732800 | -2.81797200 | 4.10656500 |
| C | 3.20020200 | -3.19814300 | 0.17581900 |
| H | 2.75044800 | -4.18383300 | 0.23036100 |
| C | -0.80471500 | -3.82921700 | -0.84047000 |
| H | -0.55350200 | -4.44180200 | -1.69828500 |
| C | 5.68231600 | 2.88688200 | -0.12007500 |
| H | 6.57136400 | 3.50489600 | -0.13285600 |
| C | -2.14001200 | -2.93486000 | -3.40963900 |
| H | -3.13509200 | -3.17341700 | -3.03114500 |
| H | -1.60400700 | -3.87529400 | -3.55459500 |
| H | -2.24408300 | -2.46701400 | -4.39039200 |
| C | -4.33911500 | -0.62712600 | 1.21703900 |
| H | -4.10709100 | -1.64111200 | 1.54655900 |
| H | -5.42427000 | -0.52630600 | 1.16479600 |
| H | -3.96007800 | 0.08076300 | 1.95525000 |
| C | 4.59784700 | -3.07288100 | 0.19388100 |
| H | 5.21312700 | -3.96150100 | 0.26079400 |
| C | -4.35875500 | -1.36562000 | -1.14585300 |
| H | -4.00019000 | -1.19457700 | -2.16020600 |
| H | -5.44213600 | -1.23300200 | -1.14167700 |
| H | -4.14042300 | -2.39653000 | -0.85893300 |

| | | | |
|---|---|---|---|
| C | -2.59544600 | 1.77385900 | -0.23836100 |
| C | -2.24426800 | 2.98231200 | -1.12898700 |
| C | -2.63758400 | 2.63465700 | 1.05902900 |
| C | -3.45121800 | 3.88707100 | -0.84847200 |
| C | -1.03008300 | 3.61823500 | -0.44271600 |
| H | -2.09620700 | 2.74232900 | -2.18287800 |
| C | -3.67728800 | 3.70056600 | 0.67484600 |
| C | -1.27101300 | 3.32667000 | 1.05890800 |
| H | -2.86499900 | 2.09175900 | 1.97813000 |
| H | -3.24279700 | 4.92157200 | -1.12307200 |
| H | -4.32708300 | 3.56727400 | -1.41705200 |
| H | -0.09545000 | 3.17154700 | -0.78654500 |
| H | -0.98341100 | 4.68682300 | -0.66050400 |
| H | -3.50110400 | 4.61783800 | 1.23863900 |
| H | -4.69757000 | 3.38198500 | 0.89326200 |
| H | -0.49063400 | 2.67655800 | 1.46015100 |
| H | -1.29448200 | 4.23101000 | 1.66858200 |

89

e-8-1

| | | | |
|---|---|---|---|
| Au | 0.47878700 | 0.29923400 | 0.06794800 |
| N | 2.47656300 | 0.21189700 | 0.17352500 |
| C | 4.73220600 | 0.63874200 | 0.29369500 |
| C | -1.30479000 | -2.89173700 | 0.89561700 |
| N | -2.10472900 | -0.93644300 | -0.34069900 |
| C | -3.57315700 | -0.84476100 | -0.60694500 |
| C | -0.71887000 | -4.15564900 | 0.88380600 |
| H | -0.62023800 | -4.69797700 | 1.81689900 |
| C | 4.40874800 | 3.36422400 | -0.12166500 |
| H | 4.30342600 | 4.42877700 | -0.29472000 |
| C | -1.48140900 | 0.19158200 | -0.12307200 |
| C | 3.13801900 | -0.98513900 | 0.35977300 |
| C | -0.37899200 | -1.97603000 | 3.01220900 |
| H | 0.29222600 | -1.32517500 | 2.44751200 |
| H | 0.15722000 | -2.90374900 | 3.22877200 |

| | | | |
|---|---|---|---|
| H | -0.61788800 | -1.48833900 | 3.95966300 |
| C | -0.83853400 | -2.70219100 | -1.50509100 |
| C | -0.22974500 | -4.70818200 | -0.28883900 |
| H | 0.22094700 | -5.69260100 | -0.27950400 |
| C | 3.44112700 | 1.19374400 | 0.11701400 |
| C | -1.41511700 | -2.20885200 | -0.32501200 |
| C | -2.46749200 | 1.34421500 | -0.14407600 |
| C | 0.80507700 | -1.63042300 | -3.03641800 |
| H | 1.29734700 | -1.17343300 | -2.17612000 |
| H | 0.92567400 | -0.96805000 | -3.89581900 |
| H | 1.31928100 | -2.56903500 | -3.25726600 |
| C | 5.38405700 | -1.87195700 | 0.63705500 |
| H | 6.45637200 | -1.72621200 | 0.70843200 |
| C | 2.59325800 | -2.26959000 | 0.44546200 |
| H | 1.52029300 | -2.42159000 | 0.36324100 |
| C | -1.66262700 | -2.26988700 | 2.23083500 |
| H | -2.15556300 | -1.31216700 | 2.05265800 |
| C | -0.68304700 | -1.88663000 | -2.77447700 |
| H | -1.16189800 | -0.91405400 | -2.62880600 |
| C | 4.53429900 | -0.78180900 | 0.44880100 |
| C | 3.28054300 | 2.56301400 | -0.10469900 |
| H | 2.29294600 | 2.97595400 | -0.28580700 |
| C | -3.81887900 | 0.67372800 | -0.49686600 |
| H | -4.57141500 | 0.88861500 | 0.25924000 |
| H | -4.21013100 | 1.06132900 | -1.43546200 |
| C | 5.85512500 | 1.46525800 | 0.27418200 |
| H | 6.84621400 | 1.04680700 | 0.41130500 |
| C | -2.58860100 | -3.15199600 | 3.06586500 |
| H | -3.49298200 | -3.43364300 | 2.52422100 |
| H | -2.88037500 | -2.62846300 | 3.97858800 |
| H | -2.07954000 | -4.07025900 | 3.36693400 |
| C | 3.45572900 | -3.33646900 | 0.62758300 |
| H | 3.04920400 | -4.34000100 | 0.69334000 |
| C | -0.26561300 | -3.97216300 | -1.46167700 |

| | | | |
|---|---|---|---|
| H | 0.18756000 | -4.37321000 | -2.36058700 |
| C | 5.68936000 | 2.82525600 | 0.07237800 |
| H | 6.55216700 | 3.47926300 | 0.05380300 |
| C | -1.29317700 | -2.57298100 | -3.99631500 |
| H | -2.34862800 | -2.81206400 | -3.86475600 |
| H | -0.76454600 | -3.50471100 | -4.21049200 |
| H | -1.19121600 | -1.93191500 | -4.87416600 |
| C | -4.36942000 | -1.66953700 | 0.39665400 |
| H | -4.09043800 | -2.72333200 | 0.32781600 |
| H | -5.42977800 | -1.58211200 | 0.15459900 |
| H | -4.22488200 | -1.34288000 | 1.42464200 |
| C | 4.84230800 | -3.14401700 | 0.72692500 |
| H | 5.49138100 | -3.99878100 | 0.87103400 |
| C | -3.89061800 | -1.38806100 | -1.99396300 |
| H | -3.35263200 | -0.85633700 | -2.77803700 |
| H | -4.96129600 | -1.28041100 | -2.17509800 |
| H | -3.63867200 | -2.44869200 | -2.05485300 |
| C | -1.94418800 | 2.30049800 | -1.21304800 |
| C | -2.18744600 | 2.04407100 | -2.56414400 |
| C | -1.10662700 | 3.36335400 | -0.87637200 |
| C | -1.64989200 | 2.84059300 | -3.56248900 |
| H | -2.80168600 | 1.19995600 | -2.85248800 |
| C | -0.57991400 | 4.16940400 | -1.88506000 |
| C | -0.84480100 | 3.91985300 | -3.22061200 |
| H | -1.85726800 | 2.61734300 | -4.60114800 |
| H | 0.06010300 | 4.99914000 | -1.60434200 |
| H | -0.42030000 | 4.55364000 | -3.98843100 |
| C | -2.44376800 | 1.94895600 | 1.25989300 |
| C | -3.19146800 | 1.37308500 | 2.28986100 |
| C | -1.62193000 | 3.03232600 | 1.56509500 |
| C | -3.15602700 | 1.86994000 | 3.58264500 |
| H | -3.82166900 | 0.51766900 | 2.08678400 |
| C | -1.60602700 | 3.54245500 | 2.86288900 |
| C | -2.36308900 | 2.97347800 | 3.87173800 |

| | | | |
|---|---|---|---|
| H | -3.74967900 | 1.40100500 | 4.35693900 |
| H | -0.97180700 | 4.39616900 | 3.07545800 |
| H | -2.33186000 | 3.38098300 | 4.87400700 |
| C | -0.70726100 | 3.62898600 | 0.54270400 |
| H | -0.61106800 | 4.70517300 | 0.70831900 |
| H | 0.29410600 | 3.20751100 | 0.70496800 |

86

e-9-1

| | | | |
|---|---|---|---|
| Au | 0.44694800 | 0.19723900 | 0.00016300 |
| N | 2.40869300 | -0.17218300 | 0.00021800 |
| C | 4.70098500 | -0.01981800 | -0.00016200 |
| C | -1.66345900 | -2.59074800 | 1.22571800 |
| N | -2.35434000 | -0.57645500 | 0.00009300 |
| C | -3.80432700 | -0.20937600 | 0.00013200 |
| C | -1.31070700 | -3.93849800 | 1.19721500 |
| H | -1.11771400 | -4.45024200 | 2.13276400 |
| C | 4.67672600 | 2.75729300 | -0.00013300 |
| H | 4.68976500 | 3.84095100 | -0.00012700 |
| C | -1.50727600 | 0.41843500 | 0.00015700 |
| C | 2.92747300 | -1.44951900 | 0.00010000 |
| C | -0.18847800 | -1.83884900 | 3.08115400 |
| H | 0.49769600 | -1.39802800 | 2.35500700 |
| H | 0.15696800 | -2.85302500 | 3.29682400 |
| H | -0.13488900 | -1.25841700 | 4.00449000 |
| C | -1.66338400 | -2.59030500 | -1.22617300 |
| C | -1.16436000 | -4.61870600 | -0.00056600 |
| H | -0.89053300 | -5.66617800 | -0.00076000 |
| C | 3.47880200 | 0.69461300 | 0.00004800 |
| C | -1.90556600 | -1.95235700 | -0.00012100 |
| C | -0.18841000 | -1.83790200 | -3.08145600 |
| H | 0.49789200 | -1.39756600 | -2.35514200 |
| H | -0.13483200 | -1.25698100 | -4.00448300 |
| H | 0.15687400 | -2.85200900 | -3.29772300 |
| C | 5.06127100 | -2.61261100 | -0.00029300 |

| | | | |
|---|---|---|---|
| H | 6.14575100 | -2.60054600 | -0.00046400 |
| C | 2.23514800 | -2.66416100 | 0.00017200 |
| H | 1.14765400 | -2.68075100 | 0.00033300 |
| C | -1.62752500 | -1.86513300 | 2.55632100 |
| H | -1.93417700 | -0.82877200 | 2.39722900 |
| C | -1.62741700 | -1.86425800 | -2.55652500 |
| H | -1.93398600 | -0.82792200 | -2.39709500 |
| C | 4.34163500 | -1.41773700 | -0.00013000 |
| C | 3.46406600 | 2.09111500 | 0.00006900 |
| H | 2.51850800 | 2.62707400 | 0.00021600 |
| C | -3.74644800 | 1.33156100 | 0.00049500 |
| H | -4.25009800 | 1.73229400 | 0.88142700 |
| H | -4.25063400 | 1.73281900 | -0.87988600 |
| C | 5.91100100 | 0.67398000 | -0.00036300 |
| H | 6.85262200 | 0.13588100 | -0.00052300 |
| C | -2.54164300 | -2.50032300 | 3.60271500 |
| H | -3.57653200 | -2.57241200 | 3.26622500 |
| H | -2.51836600 | -1.91565300 | 4.52449200 |
| H | -2.19911200 | -3.50799100 | 3.84840200 |
| C | 2.97097500 | -3.83645400 | -0.00000400 |
| H | 2.45109200 | -4.78833600 | 0.00003600 |
| C | -1.31060100 | -3.93806200 | -1.19810500 |
| H | -1.11749400 | -4.44947900 | -2.13381100 |
| C | 5.89416000 | 2.05865900 | -0.00034700 |
| H | 6.82565200 | 2.61092600 | -0.00049200 |
| C | -2.54162100 | -2.49906100 | -3.60310000 |
| H | -3.57652300 | -2.57106200 | -3.26664700 |
| H | -2.19924000 | -3.50672900 | -3.84899600 |
| H | -2.51823100 | -1.91418100 | -4.52474100 |
| C | -4.49290600 | -0.76327000 | 1.23817100 |
| H | -4.42240100 | -1.85259600 | 1.26457500 |
| H | -5.54843100 | -0.48858900 | 1.20788900 |
| H | -4.05965300 | -0.35763900 | 2.15380900 |
| C | 4.37424900 | -3.81607400 | -0.00023300 |

| | | | |
|---|---|---|---|
| H | 4.92168000 | -4.75044700 | -0.00035000 |
| C | -4.49280400 | -0.76275000 | -1.23818800 |
| H | -4.05939900 | -0.35683500 | -2.15363000 |
| H | -5.54829600 | -0.48795300 | -1.20791300 |
| H | -4.42240300 | -1.85207100 | -1.26496400 |
| C | -2.26210400 | 1.72868600 | 0.00025700 |
| C | -1.73583000 | 2.52476300 | -1.17546100 |
| C | -1.73555300 | 2.52479400 | 1.17582900 |
| C | -1.94385400 | 2.30173700 | -2.52404200 |
| C | -0.87464200 | 3.53431600 | -0.73239700 |
| C | -1.94320300 | 2.30171300 | 2.52445800 |
| C | -0.87447000 | 3.53433500 | 0.73255700 |
| C | -1.27680500 | 3.10804600 | -3.44264800 |
| H | -2.61228000 | 1.51515100 | -2.86234900 |
| C | -0.19866600 | 4.32814200 | -1.64957100 |
| C | -1.27587700 | 3.10798300 | 3.44290100 |
| H | -2.61156400 | 1.51514600 | 2.86293300 |
| C | -0.19819900 | 4.32810700 | 1.64956400 |
| C | -0.40778400 | 4.10558300 | -3.00607000 |
| H | -1.42701000 | 2.95552300 | -4.50366700 |
| H | 0.47975700 | 5.10516000 | -1.31766800 |
| C | -0.40694700 | 4.10550600 | 3.00611400 |
| H | -1.42580000 | 2.95543300 | 4.50395700 |
| H | 0.48017700 | 5.10509200 | 1.31749200 |
| H | 0.11166500 | 4.71467200 | -3.73473600 |
| H | 0.11274200 | 4.71453500 | 3.73465900 |

81

e-10-1

| | | | |
|---|---|---|---|
| Au | -0.46589600 | -0.58525600 | -0.37938500 |
| N | -2.43838600 | -0.64458500 | -0.06342800 |
| C | -4.66658800 | -1.19226300 | 0.07311300 |
| C | 1.29469500 | 2.10733700 | 1.49964700 |
| N | 2.06342300 | 0.80999900 | -0.44086600 |
| C | 3.52518000 | 0.84926300 | -0.78035400 |

| | | | |
|---|---|---|---|
| C | 0.68451400 | 3.25782500 | 1.99659500 |
| H | 0.60708600 | 3.38874400 | 3.06947600 |
| C | -4.26748200 | -3.75483800 | -0.91654300 |
| H | -4.13162700 | -4.75884300 | -1.30070500 |
| C | 1.47138500 | -0.33596500 | -0.66002100 |
| C | -3.12519700 | 0.44402600 | 0.43542600 |
| C | 0.47608500 | 0.39754500 | 3.10862800 |
| H | -0.20882500 | 0.01256900 | 2.34877100 |
| H | -0.07050000 | 1.13503000 | 3.70207700 |
| H | 0.76454600 | -0.42428600 | 3.76882700 |
| C | 0.76744500 | 2.86800000 | -0.77096200 |
| C | 0.14823400 | 4.21114200 | 1.14742100 |
| H | -0.32204400 | 5.09802200 | 1.55306900 |
| C | -3.37064900 | -1.63622800 | -0.28334200 |
| C | 1.37210600 | 1.95624200 | 0.10727300 |
| C | 2.48988500 | -1.32529000 | -1.19024000 |
| C | -0.84302900 | 2.38705400 | -2.60678900 |
| H | -1.25378400 | 1.54377600 | -2.04770600 |
| H | -0.95323800 | 2.18478100 | -3.67402800 |
| H | -1.44152800 | 3.26876600 | -2.36449500 |
| C | -5.37746100 | 1.13970000 | 1.02498500 |
| H | -6.43907600 | 0.93502400 | 1.10992300 |
| C | -2.61424600 | 1.68877300 | 0.81244700 |
| H | -1.55064500 | 1.89635700 | 0.73162800 |
| C | 1.71887600 | 1.03344400 | 2.47998800 |
| H | 2.25012500 | 0.25277500 | 1.93427400 |
| C | 0.62850600 | 2.63517000 | -2.26224100 |
| H | 1.19077900 | 1.73591500 | -2.53211500 |
| C | -4.50672300 | 0.16348600 | 0.54078400 |
| C | -3.16886600 | -2.92467500 | -0.78110200 |
| H | -2.17159900 | -3.25652000 | -1.05068500 |
| C | 3.62020600 | -0.39311600 | -1.66410300 |
| H | 4.60965700 | -0.84726200 | -1.61729700 |
| H | 3.42185700 | -0.10610100 | -2.70204500 |

| | | | |
|---|---|---|---|
| C | -5.75956000 | -2.04627000 | -0.07199800 |
| H | -6.75558300 | -1.71365700 | 0.19897700 |
| C | 2.63722100 | 1.56297200 | 3.57993200 |
| H | 3.50630900 | 2.08697700 | 3.17920400 |
| H | 2.98948000 | 0.73735300 | 4.20101400 |
| H | 2.10148800 | 2.25634100 | 4.23191300 |
| C | -3.49690700 | 2.64255800 | 1.28891200 |
| H | -3.11658000 | 3.61420700 | 1.58557700 |
| C | 0.17094100 | 4.00081700 | -0.22170300 |
| H | -0.30777100 | 4.71442900 | -0.88181600 |
| C | -5.55598400 | -3.32366800 | -0.56604700 |
| H | -6.39462800 | -3.99838100 | -0.68421300 |
| C | 1.15311400 | 3.81440600 | -3.08200200 |
| H | 2.16856700 | 4.09731300 | -2.80066700 |
| H | 0.51429900 | 4.68917800 | -2.94265400 |
| H | 1.14090100 | 3.56951300 | -4.14568000 |
| C | 4.37162700 | 0.74646200 | 0.48228900 |
| H | 4.16660000 | 1.59055600 | 1.14336800 |
| H | 5.42552600 | 0.78360300 | 0.20199500 |
| H | 4.19130600 | -0.18459800 | 1.02116400 |
| C | -4.87019200 | 2.37472200 | 1.39613300 |
| H | -5.53609700 | 3.14092300 | 1.77320700 |
| C | 3.88915100 | 2.12165700 | -1.52500400 |
| H | 3.34031700 | 2.20640000 | -2.46190300 |
| H | 4.95521500 | 2.09473900 | -1.75656400 |
| H | 3.69442600 | 3.00723100 | -0.91616200 |
| C | 2.94039000 | -2.28292900 | -0.08645300 |
| C | 4.06065000 | -3.08376700 | -0.31529000 |
| C | 2.26562400 | -2.42237000 | 1.12517700 |
| C | 4.50916400 | -3.97984700 | 0.64368300 |
| H | 4.58793400 | -3.01043900 | -1.26066800 |
| C | 2.71655700 | -3.31726200 | 2.09068100 |
| H | 1.37150200 | -1.84170600 | 1.32185700 |
| C | 3.84110700 | -4.09489400 | 1.85768400 |

| | | | |
|---|---|---|---|
| H | 5.38080300 | -4.58995400 | 0.44242700 |
| H | 2.17608900 | -3.40680300 | 3.02489300 |
| H | 4.19058500 | -4.79102900 | 2.60930300 |
| C | 1.88957000 | -2.13015600 | -2.34418200 |
| H | 1.07665400 | -2.76116500 | -1.98170100 |
| H | 1.49141300 | -1.46056100 | -3.11049900 |
| H | 2.65450100 | -2.76630100 | -2.79437500 |

90

e-11-1

| | | | |
|---|---|---|---|
| Au | -0.49768100 | -0.52476000 | -0.24455800 |
| N | -2.31962100 | -1.34331600 | -0.13430200 |
| C | -4.13855100 | -2.74803400 | -0.10961400 |
| C | -0.28983400 | 2.75340100 | 1.51965100 |
| N | 1.30325800 | 1.72390400 | -0.06878300 |
| C | 2.67553000 | 2.31177700 | -0.18573200 |
| C | -1.39349800 | 3.58068100 | 1.72457400 |
| H | -1.74564400 | 3.74361900 | 2.73631100 |
| C | -2.61746600 | -4.99488700 | -0.69924200 |
| H | -2.04083900 | -5.88317600 | -0.92859200 |
| C | 1.23590800 | 0.44145900 | -0.33313500 |
| C | -3.44270400 | -0.59625300 | 0.15051900 |
| C | -0.64654600 | 0.99931300 | 3.24942900 |
| H | -0.91898200 | 0.27948400 | 2.47290000 |
| H | -1.56779100 | 1.46901700 | 3.60358200 |
| H | -0.19559300 | 0.45778700 | 4.08419700 |
| C | -0.54901600 | 3.08675600 | -0.89288700 |
| C | -2.06059900 | 4.16521800 | 0.66254900 |
| H | -2.91897500 | 4.80037200 | 0.84137200 |
| C | -2.73790400 | -2.64615800 | -0.29282400 |
| C | 0.13591400 | 2.53737000 | 0.20403900 |
| C | 2.55836600 | -0.02625400 | -0.94654300 |
| C | -1.27781600 | 1.85974100 | -2.94080800 |
| H | -1.37645000 | 0.92788500 | -2.37985500 |
| H | -1.03229000 | 1.62041500 | -3.97798800 |

| | | | |
|---|---|---|---|
| H | -2.25020700 | 2.35891700 | -2.92765700 |
| C | -5.84107600 | -0.84016300 | 0.45255400 |
| H | -6.73415700 | -1.45498600 | 0.47760200 |
| C | -3.52545200 | 0.77760700 | 0.38980800 |
| H | -2.63136700 | 1.39398600 | 0.36247000 |
| C | 0.32029100 | 2.05703600 | 2.71477500 |
| H | 1.22510400 | 1.54914300 | 2.38526200 |
| C | -0.21118900 | 2.77723600 | -2.33842500 |
| H | 0.74777700 | 2.25092500 | -2.36930400 |
| C | -4.59787100 | -1.41121600 | 0.17932300 |
| C | -1.97245000 | -3.77578800 | -0.58887600 |
| H | -0.89959400 | -3.68821400 | -0.72689800 |
| C | 3.30710700 | 1.31979800 | -1.15543200 |
| H | 4.37662000 | 1.24207100 | -0.96780000 |
| H | 3.15695800 | 1.66889700 | -2.18362200 |
| C | -4.76701200 | -3.98770800 | -0.22567500 |
| H | -5.83911200 | -4.07429300 | -0.08666000 |
| C | 0.71190400 | 3.03632000 | 3.81930400 |
| H | 1.36283600 | 3.83009900 | 3.44575900 |
| H | 1.23242000 | 2.51024400 | 4.62155600 |
| H | -0.17091200 | 3.50792100 | 4.25583600 |
| C | -4.76898400 | 1.32199400 | 0.65908100 |
| H | -4.85168900 | 2.38715700 | 0.84760800 |
| C | -1.64597800 | 3.90413100 | -0.63371700 |
| H | -2.19604000 | 4.32495600 | -1.46690000 |
| C | -4.00485800 | -5.10573400 | -0.51993900 |
| H | -4.47893200 | -6.07482500 | -0.61317900 |
| C | -0.09216600 | 4.04275300 | -3.18951800 |
| H | 0.56663100 | 4.78724600 | -2.73922400 |
| H | -1.07062800 | 4.50701900 | -3.32771900 |
| H | 0.28968200 | 3.79279500 | -4.18098700 |
| C | 3.37336100 | 2.27371400 | 1.16925800 |
| H | 2.86784300 | 2.91804200 | 1.89078700 |
| H | 4.39593500 | 2.63538500 | 1.04960000 |

| | | | |
|---|---|---|---|
| H | 3.41581200 | 1.25389800 | 1.56010400 |
| C | -5.92212300 | 0.52239700 | 0.69164600 |
| H | -6.88155900 | 0.97707300 | 0.90497000 |
| C | 2.65540000 | 3.73485000 | -0.70967700 |
| H | 2.19185300 | 3.79573400 | -1.69304200 |
| H | 3.68524400 | 4.08522900 | -0.79670400 |
| H | 2.12462400 | 4.40032600 | -0.02584700 |
| C | 3.43486700 | -1.06049200 | -0.21408400 |
| C | 4.62860300 | -1.47013200 | -0.85513900 |
| C | 3.14386400 | -1.62366500 | 1.04501400 |
| C | 5.45967900 | -2.40884500 | -0.25006400 |
| C | 4.01093600 | -2.56470400 | 1.60247900 |
| C | 5.17021300 | -2.98576900 | 0.97541700 |
| H | 6.37234000 | -2.69342800 | -0.76642200 |
| H | 3.76034000 | -2.97488700 | 2.57598200 |
| C | 2.06263000 | -0.62979700 | -2.28281700 |
| H | 1.53632700 | -1.56472800 | -2.08656800 |
| H | 1.35686700 | 0.05208300 | -2.76740300 |
| H | 2.87886000 | -0.82049500 | -2.97283900 |
| C | 5.12998300 | -1.00046900 | -2.20583700 |
| H | 6.21820500 | -0.93246600 | -2.17816200 |
| H | 4.87650000 | -1.73109200 | -2.97813800 |
| H | 4.74927200 | -0.03958100 | -2.53563600 |
| C | 6.06472200 | -4.02443000 | 1.58714500 |
| H | 5.78801400 | -5.02368400 | 1.24340900 |
| H | 7.10693100 | -3.85982800 | 1.31193200 |
| H | 5.98923000 | -4.01883900 | 2.67468900 |
| C | 1.93941500 | -1.30498000 | 1.89071400 |
| H | 1.68274600 | -0.24865500 | 1.89361900 |
| H | 1.05120800 | -1.84314500 | 1.55228300 |
| H | 2.13230600 | -1.59791600 | 2.92325800 |

81

e-12-1

| | | | |
|---|---|---|---|
| Au | -0.66576800 | -0.50437500 | -0.28882800 |

| | | | |
|---|---|---|---|
| N | -2.31990300 | -1.62539200 | -0.21981300 |
| C | -3.88006600 | -3.31210000 | -0.27076600 |
| C | -1.00690200 | 2.66479300 | 1.62704600 |
| N | 0.72202900 | 2.01881900 | -0.01682800 |
| C | 1.97464400 | 2.83529100 | -0.10074900 |
| C | -2.22741400 | 3.28948700 | 1.88135000 |
| H | -2.59282800 | 3.32948400 | 2.90074800 |
| C | -2.00300200 | -5.25160000 | -0.92177600 |
| H | -1.28529900 | -6.02276100 | -1.17555100 |
| C | 0.86779900 | 0.75518500 | -0.32669800 |
| C | -3.55390800 | -1.08593100 | 0.07572800 |
| C | -1.08779800 | 0.78529700 | 3.25579600 |
| H | -1.28533500 | 0.08386800 | 2.44082400 |
| H | -2.05245500 | 1.10315800 | 3.65969300 |
| H | -0.54588100 | 0.25986000 | 4.04613000 |
| C | -1.33683500 | 3.10156000 | -0.76118900 |
| C | -2.98835900 | 3.82382000 | 0.85650500 |
| H | -3.93619500 | 4.30008100 | 1.07326100 |
| C | -2.51456900 | -2.97257600 | -0.43109600 |
| C | -0.56219600 | 2.60843100 | 0.30150500 |
| C | 2.24873500 | 0.55335300 | -0.96374600 |
| C | -1.87240400 | 1.87053000 | -2.86234900 |
| H | -1.80649800 | 0.91149800 | -2.34381900 |
| H | -1.60244100 | 1.72267600 | -3.91044600 |
| H | -2.91469600 | 2.19748500 | -2.82199700 |
| C | -5.88056500 | -1.73631700 | 0.33558700 |
| H | -6.65861300 | -2.49179700 | 0.32650700 |
| C | -3.86706200 | 0.24535300 | 0.36171100 |
| H | -3.08905500 | 1.00352800 | 0.36865400 |
| C | -0.28378400 | 1.99426100 | 2.77265900 |
| H | 0.67369600 | 1.62967100 | 2.40379900 |
| C | -0.96854900 | 2.92660700 | -2.22159700 |
| H | 0.06524600 | 2.57211500 | -2.28239200 |
| C | -4.55746300 | -2.08197500 | 0.05980000 |

| | | | |
|---|---|---|---|
| C | -1.57027500 | -3.94770900 | -0.75795100 |
| H | -0.52580200 | -3.67842200 | -0.87612800 |
| C | 2.76966400 | 2.00759500 | -1.10517900 |
| H | 3.83789000 | 2.10390600 | -0.92205300 |
| H | 2.56867400 | 2.36147200 | -2.12136200 |
| C | -4.29315500 | -4.63339900 | -0.44046500 |
| H | -5.33696300 | -4.90157800 | -0.31905400 |
| C | -0.02036300 | 2.95336700 | 3.93185900 |
| H | 0.50067100 | 3.85626100 | 3.60580500 |
| H | 0.58383900 | 2.46239400 | 4.69691300 |
| H | -0.95641700 | 3.26279200 | 4.40186100 |
| C | -5.18610800 | 0.56486800 | 0.63254500 |
| H | -5.44651500 | 1.59385300 | 0.85692800 |
| C | -2.54970600 | 3.71197500 | -0.45358000 |
| H | -3.16934700 | 4.08598300 | -1.25978000 |
| C | -3.35399700 | -5.59758700 | -0.76538900 |
| H | -3.65989800 | -6.62752000 | -0.90034800 |
| C | -1.07462900 | 4.23352600 | -3.00872200 |
| H | -0.54212900 | 5.05446500 | -2.52533600 |
| H | -2.11864100 | 4.53407000 | -3.11686300 |
| H | -0.67130000 | 4.09954600 | -4.01400800 |
| C | 2.65949100 | 2.86250700 | 1.26052600 |
| H | 2.04328800 | 3.38047600 | 1.99719000 |
| H | 3.60661800 | 3.39700100 | 1.17154200 |
| H | 2.86233600 | 1.85052900 | 1.61925000 |
| C | -6.18988000 | -0.41605500 | 0.62115600 |
| H | -7.21339200 | -0.13564000 | 0.83681800 |
| C | 1.71584300 | 4.25426500 | -0.56975000 |
| H | 1.25821400 | 4.27618700 | -1.55738600 |
| H | 2.67137300 | 4.77827900 | -0.62622000 |
| H | 1.07295300 | 4.79020300 | 0.13114500 |
| C | 3.26721100 | -0.36281300 | -0.27718000 |
| C | 4.51053300 | -0.60943200 | -0.89756600 |
| C | 3.10076800 | -1.03568700 | 0.94340300 |

| | | | |
|---|---|---|---|
| C | 5.45478600 | -1.49615200 | -0.40596600 |
| C | 4.02810200 | -1.93608500 | 1.45633900 |
| C | 5.19806600 | -2.17732100 | 0.76871300 |
| H | 6.38269500 | -1.64269000 | -0.94027500 |
| H | 3.82576800 | -2.43024500 | 2.39596000 |
| C | 1.85548300 | -0.05948700 | -2.33046700 |
| H | 1.50179700 | -1.08119400 | -2.18815600 |
| H | 1.03024800 | 0.51608000 | -2.75936400 |
| H | 2.68152400 | -0.05101400 | -3.03456900 |
| Cl | 5.02201200 | 0.21824300 | -2.33201500 |
| Cl | 1.75291300 | -0.76966000 | 1.98394300 |
| Cl | 6.34806600 | -3.29219800 | 1.38428800 |

99

e-13-1

| | | | |
|---|---|---|---|
| Au | -0.67063700 | -0.44651100 | -0.38020200 |
| N | -2.52978700 | -1.18380200 | -0.44342600 |
| C | -4.38692600 | -2.53760500 | -0.41201600 |
| C | -0.56248700 | 2.74503800 | 1.45478800 |
| N | 1.18818900 | 1.70891700 | 0.05066900 |
| C | 2.58621100 | 2.23020000 | 0.14741600 |
| C | -1.62466200 | 3.64379700 | 1.55112800 |
| H | -2.10613800 | 3.78431500 | 2.51177600 |
| C | -2.91206500 | -4.84908500 | -0.85061200 |
| H | -2.35398400 | -5.76160500 | -1.02356300 |
| C | 1.10426600 | 0.43398900 | -0.23951300 |
| C | -3.64006700 | -0.39492100 | -0.22856400 |
| C | -1.33941900 | 0.97064000 | 3.01120700 |
| H | -1.55083500 | 0.29152400 | 2.18097500 |
| H | -2.25600000 | 1.52767900 | 3.22136600 |
| H | -1.09728500 | 0.37690500 | 3.89587600 |
| C | -0.45553200 | 3.20921000 | -0.94901500 |
| C | -2.09374600 | 4.32499100 | 0.44186000 |
| H | -2.92314500 | 5.01437300 | 0.53699900 |
| C | -2.97884100 | -2.48132700 | -0.55457500 |

| | | | |
|---|---|---|---|
| C | 0.03534600 | 2.56974300 | 0.20116100 |
| C | 2.48182400 | -0.09208300 | -0.65306100 |
| C | -0.97117300 | 2.06858200 | -3.10339100 |
| H | -1.17813300 | 1.13008100 | -2.58428900 |
| H | -0.61063500 | 1.84105700 | -4.10908000 |
| H | -1.91453300 | 2.61338400 | -3.19441000 |
| C | -6.05178300 | -0.56228200 | 0.01347400 |
| H | -6.96181600 | -1.15169100 | 0.03859200 |
| C | -3.69181900 | 0.99019700 | -0.05332900 |
| H | -2.78131900 | 1.58232000 | -0.08638000 |
| C | -0.19572500 | 1.93215300 | 2.67731000 |
| H | 0.69048400 | 1.34095700 | 2.44514800 |
| C | 0.05263800 | 2.91942800 | -2.34811800 |
| H | 0.98030900 | 2.34362500 | -2.27246400 |
| C | -4.81754300 | -1.17714900 | -0.19864900 |
| C | -2.23685100 | -3.64339500 | -0.77662500 |
| H | -1.15918100 | -3.59090600 | -0.89486900 |
| C | 3.30223900 | 1.22457000 | -0.74577100 |
| H | 4.33557900 | 1.10738400 | -0.42394200 |
| H | 3.29849100 | 1.58780500 | -1.78018300 |
| C | -5.04579800 | -3.76416000 | -0.49103900 |
| H | -6.12369100 | -3.81577600 | -0.38300500 |
| C | 0.12692400 | 2.80546900 | 3.88816200 |
| H | 0.88448800 | 3.55856300 | 3.66134300 |
| H | 0.49242100 | 2.18313700 | 4.70834700 |
| H | -0.76504300 | 3.32676000 | 4.24260300 |
| C | -4.92663700 | 1.57790400 | 0.15880300 |
| H | -4.98534700 | 2.65225600 | 0.29745500 |
| C | -1.52365300 | 4.08929800 | -0.79962000 |
| H | -1.92585800 | 4.58239900 | -1.67648200 |
| C | -4.30643900 | -4.91470200 | -0.70877000 |
| H | -4.80436000 | -5.87414400 | -0.77265300 |
| C | 0.34070400 | 4.19493600 | -3.14077100 |
| H | 0.97313200 | 4.89291200 | -2.58931600 |

| | | | |
|---|---|---|---|
| H | -0.58778100 | 4.71339600 | -3.38824800 |
| H | 0.83305500 | 3.94752300 | -4.08288200 |
| C | 3.06493100 | 2.11993300 | 1.58909900 |
| H | 2.46934000 | 2.75215800 | 2.24998700 |
| H | 4.10502800 | 2.44472900 | 1.64876500 |
| H | 3.00682100 | 1.08461300 | 1.93231200 |
| C | -6.10167200 | 0.81083700 | 0.19308700 |
| H | -7.05374500 | 1.29937400 | 0.35926000 |
| C | 2.71399900 | 3.66309000 | -0.33160600 |
| H | 2.40519200 | 3.77130300 | -1.37016800 |
| H | 3.76151700 | 3.95945200 | -0.25565900 |
| H | 2.12179500 | 4.34061200 | 0.28674200 |
| C | 3.21005300 | -1.17460300 | 0.17757300 |
| C | 4.45486200 | -1.63545100 | -0.33418800 |
| C | 2.72733000 | -1.75902000 | 1.36903900 |
| C | 5.11287700 | -2.68930700 | 0.29235000 |
| C | 3.42367800 | -2.82470300 | 1.94678700 |
| C | 4.59854200 | -3.30884400 | 1.41583900 |
| H | 6.05668600 | -3.03184000 | -0.11547300 |
| H | 3.01888400 | -3.27884500 | 2.84322200 |
| H | 5.11630200 | -4.13997700 | 1.87736100 |
| C | 2.14087200 | -0.68754900 | -2.04255100 |
| H | 1.51031900 | -1.56736600 | -1.90641800 |
| H | 1.57365900 | 0.03801900 | -2.63465900 |
| H | 3.02365300 | -0.97628000 | -2.60448900 |
| C | 5.21717400 | -1.05368900 | -1.52510500 |
| H | 4.65706800 | -0.24015700 | -1.97681100 |
| C | 1.49787000 | -1.33555700 | 2.15398400 |
| H | 1.06165600 | -0.45147000 | 1.69393400 |
| C | 6.55563100 | -0.46467600 | -1.07340500 |
| H | 7.06537800 | 0.00797300 | -1.91571500 |
| H | 6.42207000 | 0.28113200 | -0.28642700 |
| H | 7.21253800 | -1.24222500 | -0.67906800 |
| C | 5.45097700 | -2.09868400 | -2.61770300 |

| | | | |
|---|---|---|---|
| H | 5.92665100 | -1.63798000 | -3.48606500 |
| H | 6.10883100 | -2.89492900 | -2.26471500 |
| H | 4.51665200 | -2.56259400 | -2.93892100 |
| C | 1.84781000 | -0.96063500 | 3.59686200 |
| H | 2.17700600 | -1.83264700 | 4.16501800 |
| H | 2.64247900 | -0.21319900 | 3.64847500 |
| H | 0.96525800 | -0.55869600 | 4.10021600 |
| C | 0.42391600 | -2.42386900 | 2.14636500 |
| H | -0.49699100 | -2.05718400 | 2.60648400 |
| H | 0.18572300 | -2.74571300 | 1.13073300 |
| H | 0.76184200 | -3.29817000 | 2.70883100 |

87

e-14-1

| | | | |
|---|---|---|---|
| Au | -1.00809900 | -0.39558500 | -0.63068900 |
| N | -2.64372600 | -1.50128900 | -0.34587100 |
| C | -4.28465100 | -3.11048600 | -0.35019500 |
| C | -0.68766000 | 2.48078300 | 1.72320000 |
| N | 0.45662200 | 2.08743900 | -0.41908200 |
| C | 1.66293100 | 2.92260700 | -0.73712800 |
| C | -1.74502500 | 3.07014600 | 2.41474000 |
| H | -1.78562100 | 2.97081500 | 3.49288100 |
| C | -2.74475600 | -4.91361500 | -1.79334100 |
| H | -2.15868800 | -5.63117500 | -2.35506900 |
| C | 0.49574400 | 0.85685100 | -0.85986900 |
| C | -3.73989900 | -1.00785300 | 0.33466200 |
| C | -0.42913500 | 0.35563800 | 2.98271600 |
| H | -0.86914300 | -0.20377100 | 2.15279200 |
| H | -1.24064700 | 0.61995000 | 3.66557300 |
| H | 0.26508400 | -0.29673900 | 3.51560200 |
| C | -1.71601100 | 3.22276500 | -0.37688200 |
| C | -2.75543800 | 3.74204800 | 1.74809500 |
| H | -3.56816200 | 4.19188700 | 2.30421700 |
| C | -2.97318500 | -2.77465200 | -0.76173700 |
| C | -0.66479300 | 2.61629800 | 0.32752800 |

| | | | |
|---|---|---|---|
| C | 1.80566300 | 0.63542300 | -1.59103800 |
| C | -3.01549000 | 2.28526500 | -2.28177900 |
| H | -2.89224400 | 1.27237900 | -1.89277100 |
| H | -3.10418900 | 2.23303300 | -3.36871700 |
| H | -3.95272200 | 2.68118300 | -1.88298400 |
| C | -5.98429700 | -1.65225300 | 1.00424800 |
| H | -6.79222600 | -2.37502500 | 1.03271500 |
| C | -3.88896800 | 0.24177700 | 0.94135700 |
| H | -3.08057900 | 0.96787700 | 0.91751400 |
| C | 0.28787500 | 1.62024000 | 2.50004900 |
| H | 1.09533400 | 1.31695400 | 1.83141200 |
| C | -1.84750800 | 3.19406600 | -1.88698500 |
| H | -0.93377300 | 2.76814000 | -2.31363500 |
| C | -4.78428000 | -1.95942900 | 0.36329900 |
| C | -2.19774100 | -3.68005400 | -1.48715100 |
| H | -1.19230200 | -3.41409600 | -1.79657600 |
| C | 2.28230500 | 2.07747600 | -1.85022900 |
| H | 3.36748500 | 2.17956900 | -1.88107000 |
| H | 1.87937600 | 2.40684900 | -2.81317100 |
| C | -4.81587100 | -4.35964500 | -0.66971700 |
| H | -5.81992600 | -4.62514800 | -0.35774400 |
| C | 0.90835400 | 2.34578600 | 3.69248500 |
| H | 1.37169900 | 3.29209200 | 3.40806700 |
| H | 1.66821500 | 1.71293700 | 4.15460000 |
| H | 0.15376100 | 2.55899300 | 4.45258200 |
| C | -5.08897800 | 0.52553100 | 1.57042500 |
| H | -5.22171600 | 1.49097800 | 2.04678000 |
| C | -2.74905900 | 3.79240400 | 0.36355200 |
| H | -3.57467800 | 4.25948000 | -0.16003000 |
| C | -4.04415600 | -5.25584900 | -1.38990100 |
| H | -4.44248300 | -6.22979100 | -1.64516300 |
| C | -2.06553400 | 4.58884400 | -2.47457300 |
| H | -1.31602500 | 5.30534800 | -2.13513400 |
| H | -3.04618400 | 4.97399900 | -2.18745900 |

| | | | |
|---|---|---|---|
| H | -2.04013300 | 4.54693400 | -3.56498200 |
| C | 2.55978200 | 3.04599400 | 0.48844600 |
| H | 2.02259200 | 3.53869300 | 1.30091800 |
| H | 3.42531100 | 3.65882300 | 0.23239500 |
| H | 2.91579700 | 2.07540000 | 0.83663000 |
| C | -6.13232400 | -0.41185800 | 1.60426200 |
| H | -7.05924500 | -0.16146600 | 2.10494600 |
| C | 1.27275700 | 4.31067600 | -1.21307400 |
| H | 0.66906400 | 4.26683200 | -2.11834200 |
| H | 2.18324900 | 4.86852500 | -1.43773800 |
| H | 0.72117600 | 4.85320000 | -0.44250100 |
| C | 2.78820200 | -0.14520000 | -0.72422800 |
| C | 4.10047400 | -0.28776500 | -1.16998000 |
| C | 2.44362900 | -0.72457300 | 0.49385900 |
| C | 5.04185000 | -0.96871400 | -0.41325700 |
| H | 4.40116000 | 0.13956500 | -2.12127500 |
| C | 3.40013600 | -1.38558200 | 1.25539100 |
| H | 1.42451200 | -0.67086500 | 0.86280100 |
| C | 4.70677000 | -1.51771300 | 0.81545300 |
| H | 5.44439600 | -2.03577500 | 1.41197900 |
| C | 1.55053200 | -0.12826100 | -2.89232400 |
| H | 1.19893200 | -1.13849100 | -2.67809200 |
| H | 0.78697000 | 0.38237500 | -3.48339600 |
| H | 2.46669100 | -0.18979100 | -3.48321000 |
| C | 6.43781800 | -1.08662300 | -0.96328600 |
| C | 2.99518100 | -1.89614200 | 2.61212300 |
| F | 7.26458400 | -1.70148100 | -0.11869600 |
| F | 3.88323200 | -2.75357000 | 3.11485000 |
| F | 1.80876800 | -2.50378700 | 2.58120600 |
| F | 2.88838400 | -0.87900500 | 3.48599600 |
| F | 6.45095200 | -1.77107600 | -2.11336700 |
| F | 6.95607500 | 0.12017800 | -1.23016200 |

88

e-15-1

| | | | |
|---|---|---|---|
| Au | -0.51337300 | 0.39482600 | 0.02856500 |
| N | -2.49215600 | 0.49115000 | -0.22999500 |
| C | -4.68061000 | 1.15595000 | -0.45235400 |
| C | 1.03905700 | -2.78302300 | -0.91362900 |
| N | 1.91795300 | -1.05248000 | 0.57494300 |
| C | 3.37096500 | -1.09951200 | 0.92449600 |
| C | 0.36989400 | -3.99555900 | -1.06770200 |
| H | 0.27784100 | -4.42116500 | -2.06016900 |
| C | -4.05700800 | 3.85092600 | -0.21033200 |
| H | -3.83416400 | 4.90708500 | -0.11546400 |
| C | 1.42051000 | 0.13385000 | 0.33889900 |
| C | -3.27409900 | -0.63412900 | -0.38782500 |
| C | 0.28532000 | -1.62355000 | -2.98558300 |
| H | -0.41023600 | -1.01977100 | -2.39805000 |
| H | -0.25599500 | -2.50041600 | -3.35022100 |
| H | 0.60879700 | -1.03696500 | -3.84867500 |
| C | 0.48300900 | -2.83911900 | 1.47631100 |
| C | -0.20659000 | -4.64187500 | 0.01410200 |
| H | -0.72125200 | -5.58388700 | -0.12753700 |
| C | -3.34145000 | 1.57568500 | -0.26609000 |
| C | 1.14146500 | -2.25715600 | 0.38458500 |
| C | 2.54745500 | 1.16228000 | 0.36385000 |
| C | -1.13388700 | -1.76219900 | 3.02889900 |
| H | -1.50178800 | -1.12651700 | 2.22087600 |
| H | -1.25049100 | -1.22749900 | 3.97384300 |
| H | -1.76503900 | -2.65365200 | 3.06388000 |
| C | -5.59452700 | -1.28444800 | -0.69917100 |
| H | -6.64254100 | -1.02812200 | -0.80941900 |
| C | -2.86788500 | -1.97090300 | -0.41675400 |
| H | -1.81775800 | -2.23041700 | -0.31062200 |
| C | 1.50220600 | -2.04889400 | -2.15852200 |
| H | 2.02381500 | -1.13544400 | -1.86423700 |
| C | 0.33213300 | -2.15507400 | 2.82057000 |
| H | 0.92216800 | -1.23407800 | 2.80890100 |

| | | | |
|---|---|---|---|
| C | -4.63688600 | -0.28443100 | -0.52980200 |
| C | -3.02638900 | 2.93018500 | -0.14110000 |
| H | -1.99737900 | 3.24097300 | 0.01219700 |
| C | 3.65318600 | 0.39690900 | 1.11693300 |
| H | 4.66451700 | 0.67611500 | 0.82557300 |
| H | 3.54772400 | 0.63338100 | 2.17841200 |
| C | -5.70287600 | 2.10240500 | -0.51892800 |
| H | -6.73152700 | 1.79009600 | -0.66219400 |
| C | 2.44026600 | -2.88685300 | -3.02603400 |
| H | 3.29676700 | -3.26622300 | -2.46747200 |
| H | 2.81073200 | -2.28394300 | -3.85813300 |
| H | 1.91105700 | -3.74320900 | -3.45053500 |
| C | -3.83591400 | -2.94591300 | -0.58358200 |
| H | -3.53839600 | -3.98876000 | -0.60772800 |
| C | -0.17403900 | -4.04993000 | 1.26628900 |
| H | -0.69432700 | -4.51625500 | 2.09467100 |
| C | -5.38719100 | 3.44537300 | -0.39905100 |
| H | -6.17018700 | 4.19173900 | -0.44887100 |
| C | 0.79599100 | -3.03097900 | 3.98323000 |
| H | 1.82267900 | -3.37786900 | 3.86141400 |
| H | 0.15482000 | -3.91036600 | 4.07623300 |
| H | 0.72688400 | -2.47738900 | 4.92155700 |
| C | 4.16797400 | -1.78137400 | -0.18076000 |
| H | 3.86984600 | -2.82976500 | -0.25434500 |
| H | 5.22843600 | -1.74807900 | 0.07654900 |
| H | 4.03049700 | -1.31270400 | -1.15206700 |
| C | -5.19129400 | -2.60999100 | -0.72418900 |
| H | -5.92538000 | -3.39541200 | -0.85441300 |
| C | 3.59610300 | -1.87616200 | 2.21310000 |
| H | 3.06588400 | -1.42664000 | 3.05277000 |
| H | 4.66373700 | -1.87101600 | 2.44022800 |
| H | 3.27698900 | -2.91447600 | 2.10100100 |
| C | 2.83727600 | 1.43748200 | -1.11541200 |
| C | 4.06483500 | 1.18183600 | -1.71826800 |

| | | | |
|---|---|---|---|
| C | 1.80766100 | 1.95537200 | -1.90901300 |
| C | 4.24801000 | 1.38829000 | -3.08322800 |
| H | 4.89978200 | 0.81324100 | -1.13769500 |
| C | 1.98389900 | 2.15625400 | -3.26882400 |
| H | 0.85883700 | 2.20550700 | -1.44709900 |
| C | 3.20515500 | 1.86000600 | -3.86594400 |
| H | 5.21229400 | 1.17906100 | -3.52910800 |
| H | 1.16640400 | 2.54661700 | -3.86131300 |
| H | 3.34529200 | 2.01210300 | -4.92853100 |
| C | 2.13790800 | 2.42202100 | 1.11126800 |
| C | 1.36851100 | 2.30251300 | 2.26912500 |
| C | 2.55200600 | 3.68681900 | 0.70852900 |
| C | 1.00652000 | 3.42338200 | 3.00113900 |
| H | 1.03719600 | 1.31846500 | 2.59247600 |
| C | 2.19866000 | 4.81213100 | 1.44569300 |
| H | 3.14658200 | 3.79643300 | -0.19076200 |
| C | 1.42206400 | 4.68473300 | 2.58879500 |
| H | 0.39450900 | 3.31392800 | 3.88722200 |
| H | 2.52663000 | 5.79082700 | 1.11871700 |
| H | 1.13726200 | 5.56255800 | 3.15460700 |

106

e-16-1

| | | | |
|---|---|---|---|
| Au | 0.68104100 | 0.42370000 | -0.09764000 |
| N | 2.54123800 | 1.07119000 | 0.26182100 |
| C | 4.38299900 | 2.32742600 | 0.82311300 |
| C | 0.36789900 | -3.10260200 | 0.35703900 |
| N | -1.11117800 | -1.61461600 | -0.93030100 |
| C | -2.43619000 | -2.17294500 | -1.32526000 |
| C | 1.38427400 | -4.05667200 | 0.36056300 |
| H | 1.63737000 | -4.54394200 | 1.29469900 |
| C | 2.93259900 | 4.69077900 | 0.96612900 |
| H | 2.38460400 | 5.62401700 | 1.02128800 |
| C | -1.10078900 | -0.37185600 | -0.50977600 |
| C | 3.63825100 | 0.23796800 | 0.31062200 |

| | | | |
|---|---|---|---|
| C | 0.82354100 | -2.06127300 | 2.56983400 |
| H | 1.23235900 | -1.16358300 | 2.10022800 |
| H | 1.65166500 | -2.75051700 | 2.75420300 |
| H | 0.39215400 | -1.77941800 | 3.53337100 |
| C | 0.82205300 | -2.71495100 | -2.01760900 |
| C | 2.09306300 | -4.36749300 | -0.78752800 |
| H | 2.87699200 | -5.11378800 | -0.75914700 |
| C | 2.98954400 | 2.33736200 | 0.56845900 |
| C | 0.05179300 | -2.48563500 | -0.86718600 |
| C | -2.56562000 | 0.13863100 | -0.37899300 |
| C | 1.93109000 | -0.94587300 | -3.35830000 |
| H | 2.03902800 | -0.33250600 | -2.46055600 |
| H | 1.84171600 | -0.28550100 | -4.22408700 |
| H | 2.84638700 | -1.53331200 | -3.46943400 |
| C | 6.02672100 | 0.29139800 | 0.75521700 |
| H | 6.92865300 | 0.83376700 | 1.01718700 |
| C | 3.68690600 | -1.13755400 | 0.07283800 |
| H | 2.78353300 | -1.67986700 | -0.18872300 |
| C | -0.24392300 | -2.72604700 | 1.69417500 |
| H | -1.04172800 | -1.99658700 | 1.53741100 |
| C | 0.71321100 | -1.87068400 | -3.26873500 |
| H | -0.16996700 | -1.24199600 | -3.17520600 |
| C | 4.80570300 | 0.95871000 | 0.65324300 |
| C | 2.26060100 | 3.52711500 | 0.63459100 |
| H | 1.19731200 | 3.53130400 | 0.41685500 |
| C | -3.31245900 | -0.91107000 | -1.23838900 |
| H | -4.31882300 | -1.12843000 | -0.90680800 |
| H | -3.41066100 | -0.49209800 | -2.24124800 |
| C | 5.03853900 | 3.51288300 | 1.15443900 |
| H | 6.10511700 | 3.51316500 | 1.35107400 |
| C | -0.82084000 | -3.93001100 | 2.44012800 |
| H | -1.51335700 | -4.51057000 | 1.82925500 |
| H | -1.35272700 | -3.58768300 | 3.33172400 |
| H | -0.02294300 | -4.60006100 | 2.76904700 |

| | | | |
|---|---|---|---|
| C | 4.90864300 | -1.77850000 | 0.17907800 |
| H | 4.96365300 | -2.84653900 | -0.00311300 |
| C | 1.83154800 | -3.67332200 | -1.95622900 |
| H | 2.43813500 | -3.85146700 | -2.83624300 |
| C | 4.31110800 | 4.68909400 | 1.22757600 |
| H | 4.80678900 | 5.61724000 | 1.48342800 |
| C | 0.59928800 | -2.69468200 | -4.54953400 |
| H | -0.22460600 | -3.40869400 | -4.51485000 |
| H | 1.51945700 | -3.25312300 | -4.73419800 |
| H | 0.44572100 | -2.03370100 | -5.40479800 |
| C | -2.78896800 | -3.33093200 | -0.39993400 |
| H | -2.06151100 | -4.13428000 | -0.53695200 |
| H | -3.77210600 | -3.72330600 | -0.66544700 |
| H | -2.78555200 | -3.04106100 | 0.64847800 |
| C | 6.07394500 | -1.07290800 | 0.51775200 |
| H | 7.01586000 | -1.60192300 | 0.59346100 |
| C | -2.41505700 | -2.72476300 | -2.74546400 |
| H | -2.15895400 | -1.96547400 | -3.48453300 |
| H | -3.41204000 | -3.10311400 | -2.97960000 |
| H | -1.71033000 | -3.55444600 | -2.82593200 |
| C | -2.72888700 | -0.00791900 | 1.16003200 |
| C | -3.60355700 | -0.91799300 | 1.80766000 |
| C | -1.86139500 | 0.74860900 | 1.98811900 |
| C | -3.37225400 | -1.24160000 | 3.14575800 |
| C | -1.67122600 | 0.37981300 | 3.31788600 |
| C | -2.36196900 | -0.66813700 | 3.90350600 |
| H | -4.03615900 | -1.95968100 | 3.61857700 |
| H | -0.95874400 | 0.94743100 | 3.90880900 |
| C | -2.80480900 | 1.56107100 | -0.91695300 |
| C | -2.11788800 | 2.01724500 | -2.06155100 |
| C | -3.71370500 | 2.44719400 | -0.30303100 |
| C | -2.24291500 | 3.34286600 | -2.46953400 |
| C | -3.80632200 | 3.76300000 | -0.75255800 |
| C | -3.05352900 | 4.25163600 | -1.80854300 |

| | | | |
|---|---|---|---|
| H | -1.68981400 | 3.66477200 | -3.34691300 |
| H | -4.50784900 | 4.42607800 | -0.25489300 |
| C | -1.25554100 | 1.14940000 | -2.94099900 |
| H | -0.22822800 | 1.07373800 | -2.57566900 |
| H | -1.64631200 | 0.13726500 | -3.03193000 |
| H | -1.21958300 | 1.57514300 | -3.94426700 |
| C | -3.12904400 | 5.69000900 | -2.23016300 |
| H | -4.09458700 | 6.12648700 | -1.97330600 |
| H | -2.35703600 | 6.27929500 | -1.73027100 |
| H | -2.97609300 | 5.79531000 | -3.30461800 |
| C | -4.65473000 | 2.08523200 | 0.81810400 |
| H | -5.11341000 | 1.11159800 | 0.66807900 |
| H | -4.16836700 | 2.06770600 | 1.79536700 |
| H | -5.45597800 | 2.82334100 | 0.85822900 |
| C | -2.07216300 | -1.12456600 | 5.30221100 |
| H | -2.97131500 | -1.49961600 | 5.79224100 |
| H | -1.33935300 | -1.93727500 | 5.29671300 |
| H | -1.65728900 | -0.31661600 | 5.90495900 |
| C | -1.22315000 | 2.06518700 | 1.60955100 |
| H | -0.27277300 | 2.18863400 | 2.13169200 |
| H | -1.05050900 | 2.21607000 | 0.54834200 |
| H | -1.89161300 | 2.86993200 | 1.93835300 |
| C | -4.90467700 | -1.49031800 | 1.27220800 |
| H | -5.47182700 | -0.77087200 | 0.68410900 |
| H | -4.79940900 | -2.38990400 | 0.67047600 |
| H | -5.52786200 | -1.75363400 | 2.12660900 |

112

e-17-1

| | | | |
|---|---|---|---|
| Au | -1.03498800 | 0.04267300 | -0.07737100 |
| N | -2.87292200 | 0.36705800 | -0.80410700 |
| C | -4.81702100 | 1.35596600 | -1.52625300 |
| C | 0.22519500 | -3.41152500 | 0.03864600 |
| N | 0.86481000 | -1.48291200 | 1.41139800 |
| C | 2.08810300 | -1.60727200 | 2.25839100 |

| | | | |
|---|---|---|---|
| C | -0.53511600 | -4.56944300 | -0.11363000 |
| H | -0.37001700 | -5.19239300 | -0.98504800 |
| C | -3.82941600 | 3.92952000 | -1.16697400 |
| H | -3.46570500 | 4.94023800 | -1.02394600 |
| C | 0.70289300 | -0.33457300 | 0.80047600 |
| C | -3.77875100 | -0.62682200 | -1.10105600 |
| C | 0.32569500 | -2.68507400 | -2.33740800 |
| H | -0.35503200 | -1.85136800 | -2.14159300 |
| H | -0.27508700 | -3.54485800 | -2.64552200 |
| H | 0.98424300 | -2.41158700 | -3.16546200 |
| C | -1.05654500 | -2.89947300 | 2.06132300 |
| C | -1.51013000 | -4.92044000 | 0.80492700 |
| H | -2.08461100 | -5.82839300 | 0.67111200 |
| C | -3.50013800 | 1.56663000 | -1.04619300 |
| C | -0.00488800 | -2.61732100 | 1.17649000 |
| C | 1.98894200 | 0.50963500 | 0.97514700 |
| C | -2.94183600 | -1.44395900 | 2.76441200 |
| H | -2.94977700 | -0.90843400 | 1.81253200 |
| H | -3.32641600 | -0.78104400 | 3.54371500 |
| H | -3.63231200 | -2.28618100 | 2.67985400 |
| C | -6.05168900 | -0.92006800 | -1.91160400 |
| H | -6.99105300 | -0.50952600 | -2.26573900 |
| C | -3.61043600 | -2.01030800 | -0.98647400 |
| H | -2.67061400 | -2.42317300 | -0.62582900 |
| C | 1.15661400 | -3.02522000 | -1.09627600 |
| H | 1.71095800 | -2.12660800 | -0.81989500 |
| C | -1.53723500 | -1.93844400 | 3.13027700 |
| H | -0.86334600 | -1.07666000 | 3.14134500 |
| C | -4.99863200 | -0.07511400 | -1.55941300 |
| C | -3.00556600 | 2.86100600 | -0.85553300 |
| H | -2.00423300 | 3.01413600 | -0.45907700 |
| C | 2.56410700 | -0.15013100 | 2.24747600 |
| H | 3.63453600 | -0.04248900 | 2.36508100 |
| H | 2.07103100 | 0.35526600 | 3.08120700 |

| | | | |
|---|---|---|---|
| C | -5.62632200 | 2.44912100 | -1.83382900 |
| H | -6.63491600 | 2.29834800 | -2.20312200 |
| C | 2.16184500 | -4.12286900 | -1.44282000 |
| H | 2.74445200 | -4.43970700 | -0.57605000 |
| H | 2.85079300 | -3.75449500 | -2.20844400 |
| H | 1.65773600 | -5.00274800 | -1.84966700 |
| C | -4.67136700 | -2.82666400 | -1.33674200 |
| H | -4.56292900 | -3.90230300 | -1.25325200 |
| C | -1.78261100 | -4.07268100 | 1.86445100 |
| H | -2.59600900 | -4.30508300 | 2.54176600 |
| C | -5.12903600 | 3.73025800 | -1.65711900 |
| H | -5.74799600 | 4.58759400 | -1.89057500 |
| C | -1.58302000 | -2.57046700 | 4.52153700 |
| H | -0.63112300 | -3.01127200 | 4.81669600 |
| H | -2.34144200 | -3.35620400 | 4.55653700 |
| H | -1.85955400 | -1.81925500 | 5.26445500 |
| C | 3.02915600 | -2.65774500 | 1.67900700 |
| H | 2.57046300 | -3.64552000 | 1.77451400 |
| H | 3.96141300 | -2.65566600 | 2.24690700 |
| H | 3.25732900 | -2.49021800 | 0.62923500 |
| C | -5.88410600 | -2.29048100 | -1.79888900 |
| H | -6.69412700 | -2.95741700 | -2.06699600 |
| C | 1.73099300 | -2.04064600 | 3.67274300 |
| H | 1.04383500 | -1.33754100 | 4.14396900 |
| H | 2.64755600 | -2.07540700 | 4.26522800 |
| H | 1.28771000 | -3.03855300 | 3.67397100 |
| C | 2.70474600 | 0.20515400 | -0.36036100 |
| C | 3.93994700 | -0.44325100 | -0.49967300 |
| C | 2.07740000 | 0.56269700 | -1.57762900 |
| C | 4.37064400 | -0.97614300 | -1.72029600 |
| C | 2.48835500 | 0.05306600 | -2.80324200 |
| C | 3.60167200 | -0.77271700 | -2.85876600 |
| H | 5.29645500 | -1.52578700 | -1.77444800 |
| H | 1.96086500 | 0.27199300 | -3.71947200 |

| | | | |
|---|---|---|---|
| C | 1.73284400 | 1.99174300 | 1.18473300 |
| C | 0.60321200 | 2.43976200 | 1.89134400 |
| C | 2.55290000 | 2.97468100 | 0.64150400 |
| C | 0.23252800 | 3.77549200 | 1.90651600 |
| C | 2.21566200 | 4.32931500 | 0.65220300 |
| C | 1.03430400 | 4.71803700 | 1.26479200 |
| H | -0.65507600 | 4.12319100 | 2.41380300 |
| H | 2.89233000 | 5.04330300 | 0.20480200 |
| O | 0.60115000 | 5.99910700 | 1.32319900 |
| O | 3.73352700 | 2.58061000 | 0.09465800 |
| O | -0.09627200 | 1.49472000 | 2.57078400 |
| O | 3.89416500 | -1.30708700 | -4.06691900 |
| O | 1.08282600 | 1.46761300 | -1.50260600 |
| O | 4.73846100 | -0.53077900 | 0.59185500 |
| C | 5.02350400 | -2.14046600 | -4.16918500 |
| H | 4.94436100 | -2.99869100 | -3.49406700 |
| H | 5.05867500 | -2.49357400 | -5.19613800 |
| H | 5.94379200 | -1.59254200 | -3.94506700 |
| C | 5.91530400 | -1.29615100 | 0.53017800 |
| H | 6.34884000 | -1.27505000 | 1.52757400 |
| H | 5.70261300 | -2.33417700 | 0.25188600 |
| H | 6.63167600 | -0.87242400 | -0.17906600 |
| C | 4.09476100 | 3.12142500 | -1.15861100 |
| H | 3.20613400 | 3.31309900 | -1.76668500 |
| H | 4.66486200 | 4.04774400 | -1.04684000 |
| H | 4.71526800 | 2.37644900 | -1.65924900 |
| C | 1.37497200 | 6.98492700 | 0.68505400 |
| H | 1.47901600 | 6.77571000 | -0.38391700 |
| H | 0.85105500 | 7.92777400 | 0.81756100 |
| H | 2.37081800 | 7.05973000 | 1.13256900 |
| C | -1.41226900 | 1.81936100 | 2.97485000 |
| H | -1.40765100 | 2.59584700 | 3.74372500 |
| H | -2.00982400 | 2.14755700 | 2.11799500 |
| H | -1.84567200 | 0.91305100 | 3.38627000 |

| | | | |
|---|---|---|---|
| C | 0.25865100 | 1.67811800 | -2.63332600 |
| H | -0.16760000 | 0.73322500 | -2.98399100 |
| H | -0.54912100 | 2.33060700 | -2.31053000 |
| H | 0.81950000 | 2.15511300 | -3.44216700 |

88

e-18-1

| | | | |
|---|---|---|---|
| Au | 0.71088900 | 0.34163100 | -0.01111400 |
| N | 2.58180700 | 0.90207900 | 0.39932900 |
| C | 4.49479200 | 2.08389900 | 0.87516000 |
| C | 0.09000700 | -3.17826800 | 0.52188700 |
| N | -1.20070800 | -1.61365400 | -0.85834800 |
| C | -2.55918800 | -2.04342300 | -1.30611700 |
| C | 1.05102400 | -4.18453000 | 0.59849300 |
| H | 1.22997400 | -4.66550300 | 1.55299800 |
| C | 3.15472700 | 4.51193200 | 1.01521300 |
| H | 2.65004100 | 5.46896000 | 1.07480700 |
| C | -1.07306000 | -0.36822600 | -0.48131100 |
| C | 3.63948200 | 0.01776400 | 0.44383200 |
| C | 0.47404000 | -2.10391800 | 2.73798100 |
| H | 1.00498500 | -1.28109500 | 2.25375000 |
| H | 1.21398800 | -2.85071700 | 3.03638200 |
| H | -0.00756000 | -1.72382800 | 3.64247300 |
| C | 0.69081700 | -2.85872400 | -1.83497400 |
| C | 1.79931900 | -4.55372800 | -0.50745900 |
| H | 2.53930900 | -5.33978300 | -0.42499300 |
| C | 3.09714600 | 2.15335200 | 0.66255600 |
| C | -0.12132500 | -2.57285100 | -0.72855100 |
| C | -2.47523500 | 0.28908600 | -0.47295300 |
| C | 1.98175700 | -1.21471700 | -3.17743400 |
| H | 2.10360300 | -0.57121600 | -2.30367000 |
| H | 1.97980800 | -0.58848300 | -4.07189200 |
| H | 2.84952500 | -1.87775200 | -3.22627700 |
| C | 6.03912200 | -0.02938100 | 0.81971900 |
| H | 6.97277300 | 0.47662600 | 1.03968700 |

| | | | |
|---|---|---|---|
| C | 3.61524800 | -1.36555200 | 0.25191100 |
| H | 2.67931300 | -1.87397600 | 0.03815200 |
| C | -0.57349900 | -2.72723800 | 1.81022400 |
| H | -1.30597900 | -1.94953200 | 1.58163100 |
| C | 0.68779500 | -2.03137600 | -3.10396500 |
| H | -0.14098000 | -1.32477500 | -3.05384600 |
| C | 4.84839500 | 0.69194200 | 0.73174800 |
| C | 2.42137900 | 3.37294500 | 0.73288200 |
| H | 1.34889700 | 3.41324500 | 0.56989000 |
| C | -3.27101400 | -0.68525600 | -1.37222500 |
| H | -4.32883900 | -0.74523600 | -1.14622900 |
| H | -3.17335000 | -0.31337800 | -2.39324900 |
| C | 5.21328200 | 3.24546900 | 1.15766000 |
| H | 6.28441400 | 3.20214400 | 1.32173400 |
| C | -1.28328300 | -3.86680300 | 2.54096200 |
| H | -1.98970500 | -4.39746000 | 1.90171300 |
| H | -1.82534400 | -3.47268300 | 3.40413000 |
| H | -0.55861300 | -4.59411000 | 2.91404100 |
| C | 4.80859500 | -2.06057500 | 0.34288100 |
| H | 4.80818800 | -3.13539300 | 0.19616700 |
| C | 1.63835200 | -3.87194200 | -1.70214100 |
| H | 2.27984100 | -4.10342300 | -2.54423400 |
| C | 4.54088400 | 4.45410400 | 1.22634400 |
| H | 5.08524200 | 5.36433200 | 1.44482300 |
| C | 0.54732200 | -2.88008700 | -4.36642600 |
| H | -0.33212400 | -3.52485100 | -4.34159700 |
| H | 1.42485500 | -3.51614700 | -4.50208700 |
| H | 0.47656800 | -2.23445600 | -5.24377300 |
| C | -3.12851800 | -3.06795800 | -0.33267800 |
| H | -2.54882400 | -3.99123700 | -0.40338400 |
| H | -4.16219400 | -3.28652100 | -0.60190000 |
| H | -3.10428900 | -2.73239000 | 0.70041900 |
| C | 6.01519200 | -1.40103600 | 0.62348900 |
| H | 6.93310700 | -1.97184600 | 0.68910500 |

| | | | |
|---|---|---|---|
| C | -2.51137600 | -2.68844500 | -2.68394400 |
| H | -2.12305700 | -2.00359500 | -3.43785100 |
| H | -3.52706900 | -2.97239400 | -2.96585800 |
| H | -1.89900100 | -3.59185500 | -2.66706500 |
| C | -2.82136900 | 0.27456000 | 1.02396500 |
| C | -3.81848700 | -0.47840600 | 1.64433600 |
| C | -2.03940300 | 1.00963200 | 1.92800100 |
| C | -3.92482800 | -0.65254400 | 3.01419500 |
| C | -2.09038600 | 0.88229400 | 3.30075000 |
| C | -3.02607100 | 0.01076100 | 3.82321300 |
| H | -4.71096200 | -1.27002500 | 3.42389100 |
| H | -1.43386900 | 1.46541000 | 3.92990300 |
| C | -2.45908600 | 1.68112800 | -1.08228000 |
| C | -1.72633200 | 1.92854800 | -2.24294700 |
| C | -3.17000700 | 2.78056300 | -0.60844200 |
| C | -1.63298000 | 3.15481900 | -2.86815400 |
| C | -3.12528400 | 4.03664500 | -1.18519300 |
| C | -2.34049500 | 4.20150400 | -2.30860800 |
| H | -1.03394100 | 3.28141200 | -3.75782500 |
| H | -3.69063900 | 4.85481900 | -0.76357800 |
| F | -2.27763000 | 5.40164400 | -2.88113300 |
| F | -1.09111000 | 0.89756900 | -2.81885400 |
| F | -3.95889100 | 2.64177900 | 0.46161500 |
| F | -4.77875300 | -1.07003900 | 0.92141200 |
| F | -1.22476800 | 1.94768700 | 1.44824100 |
| F | -3.09781700 | -0.15274100 | 5.14151500 |

88

e-19-1

| | | | |
|---|---|---|---|
| Au | 0.93412300 | 0.44504800 | 0.00430300 |
| N | 2.67395500 | 1.25127400 | 0.57061600 |
| C | 4.32475000 | 2.61832300 | 1.40169400 |
| C | 1.09725900 | -3.13133500 | -0.28515600 |
| N | -0.51497300 | -1.60513800 | -1.35091900 |
| C | -1.73434900 | -2.21389700 | -1.96066100 |

| | | | |
|---|---|---|---|
| C | 2.23872600 | -3.92335800 | -0.39996700 |
| H | 2.51988400 | -4.55558700 | 0.43379500 |
| C | 2.57119900 | 4.71206800 | 1.90004700 |
| H | 1.90306900 | 5.54221300 | 2.09654000 |
| C | -0.67970800 | -0.48512000 | -0.68995200 |
| C | 3.87341700 | 0.57334300 | 0.50832700 |
| C | 1.31075500 | -2.52686700 | 2.12160500 |
| H | 1.61060700 | -1.50563700 | 1.87439100 |
| H | 2.21847900 | -3.12767600 | 2.21717100 |
| H | 0.80892200 | -2.52054100 | 3.09275800 |
| C | 1.59024500 | -2.20762700 | -2.49982100 |
| C | 3.03314800 | -3.89316100 | -1.53336600 |
| H | 3.91474600 | -4.51833600 | -1.59743000 |
| C | 2.94561800 | 2.49184400 | 1.10632900 |
| C | 0.75019500 | -2.32214100 | -1.38178200 |
| C | -2.21364300 | -0.20235200 | -0.57588900 |
| C | 2.45401300 | -0.05252200 | -3.37946500 |
| H | 2.39974300 | 0.39119900 | -2.38268100 |
| H | 2.31070600 | 0.73777300 | -4.12037000 |
| H | 3.45966500 | -0.46202300 | -3.50680100 |
| C | 6.22831300 | 0.86027100 | 1.03451400 |
| H | 7.04574800 | 1.46107200 | 1.41793900 |
| C | 4.11066200 | -0.71813800 | 0.03248400 |
| H | 3.29214900 | -1.31866400 | -0.35225500 |
| C | 0.38483300 | -3.12220400 | 1.05521600 |
| H | -0.49615900 | -2.47986100 | 0.98630700 |
| C | 1.40497800 | -1.15235100 | -3.56836700 |
| H | 0.42367300 | -0.70275000 | -3.42788000 |
| C | 4.92932700 | 1.36824400 | 1.01049700 |
| C | 2.06462600 | 3.54710500 | 1.35108100 |
| H | 1.01297800 | 3.45029200 | 1.10172300 |
| C | -2.74905800 | -1.08742400 | -1.72194100 |
| H | -3.76607000 | -1.43454800 | -1.59227100 |
| H | -2.73689900 | -0.45164400 | -2.61030900 |

| | | | |
|---|---|---|---|
| C | 4.81303000 | 3.80192800 | 1.95426500 |
| H | 5.86791100 | 3.90571400 | 2.18363000 |
| C | -0.05890800 | -4.51639600 | 1.49916900 |
| H | -0.67000200 | -5.02079900 | 0.74941700 |
| H | -0.63331500 | -4.44382300 | 2.42674600 |
| H | 0.80779900 | -5.14880200 | 1.70401600 |
| C | 5.40714000 | -1.20124000 | 0.06449200 |
| H | 5.60830700 | -2.20225800 | -0.30214600 |
| C | 2.72379900 | -3.01512400 | -2.55785300 |
| H | 3.38571700 | -2.92746900 | -3.41105100 |
| C | 3.93461500 | 4.84294100 | 2.20400100 |
| H | 4.29928500 | 5.76809700 | 2.63247900 |
| C | 1.49041700 | -1.70737000 | -4.98858600 |
| H | 0.80558400 | -2.54048800 | -5.15302500 |
| H | 2.50161400 | -2.05534800 | -5.20958500 |
| H | 1.25534500 | -0.92173300 | -5.70917900 |
| C | -2.00570400 | -3.56453500 | -1.31002600 |
| H | -1.20951000 | -4.25912700 | -1.58824300 |
| H | -2.95350800 | -3.96209900 | -1.67284400 |
| H | -2.04272700 | -3.51094200 | -0.22542800 |
| C | 6.46252500 | -0.42109700 | 0.56183400 |
| H | 7.46663400 | -0.82643400 | 0.57489500 |
| C | -1.56545900 | -2.45710300 | -3.45496000 |
| H | -1.38690500 | -1.53241900 | -4.00405800 |
| H | -2.48637300 | -2.90682900 | -3.83116600 |
| H | -0.74765400 | -3.15483000 | -3.64227100 |
| C | -2.44176000 | -0.68206100 | 0.88117800 |
| C | -3.14908600 | -1.82926200 | 1.29314800 |
| C | -1.76927600 | -0.03057500 | 1.93961800 |
| C | -2.94164600 | -2.43911400 | 2.52309600 |
| C | -1.54474300 | -0.61744700 | 3.17544300 |
| C | -2.07551300 | -1.86610100 | 3.43409000 |
| H | -3.48117100 | -3.34395200 | 2.76619500 |
| H | -0.96529700 | -0.08952600 | 3.91983100 |

| | | | |
|---|---|---|---|
| C | -2.64280600 | 1.24421800 | -0.82062600 |
| C | -1.97256500 | 2.08982400 | -1.71629100 |
| C | -3.77345400 | 1.81784700 | -0.22167000 |
| C | -2.27825400 | 3.43288300 | -1.87340000 |
| C | -4.11260800 | 3.15484300 | -0.36316800 |
| C | -3.33605200 | 3.96964100 | -1.16441700 |
| H | -1.70317800 | 4.03881200 | -2.55910400 |
| H | -4.98165200 | 3.54458000 | 0.14755800 |
| Cl | -0.75537500 | 1.49492400 | -2.78369500 |
| Cl | -3.72309100 | 5.63333800 | -1.32746800 |
| Cl | -4.87805500 | 0.88745800 | 0.71986700 |
| Cl | -1.27112800 | 1.61509800 | 1.83599500 |
| Cl | -1.73121600 | -2.66545000 | 4.91172600 |
| Cl | -4.47411700 | -2.52029500 | 0.41924200 |

100

e-20-1

| | | | |
|---|---|---|---|
| Au | -1.02929500 | 0.39667700 | -0.11513600 |
| N | -2.38099900 | 1.11286600 | -1.38775700 |
| C | -3.46100100 | 2.05561900 | -3.18286900 |
| C | -2.07580100 | -2.66692100 | 1.45240100 |
| N | -0.21278100 | -1.22614400 | 2.13269000 |
| C | 0.83525500 | -1.79694200 | 3.03320200 |
| C | -3.39721000 | -3.06732500 | 1.63995200 |
| H | -3.78631100 | -3.88475700 | 1.04440300 |
| C | -1.11382700 | 3.05491200 | -4.28082100 |
| H | -0.20838100 | 3.44881100 | -4.72635700 |
| C | 0.20447800 | -0.44560800 | 1.17350800 |
| C | -3.72798000 | 0.83003500 | -1.28084700 |
| C | -1.92265100 | -3.01392700 | -1.01664700 |
| H | -2.05966500 | -1.94240500 | -1.17700100 |
| H | -2.90359100 | -3.49025800 | -1.08678800 |
| H | -1.29841900 | -3.40557500 | -1.82379100 |
| C | -2.44803700 | -0.86849700 | 3.08508800 |
| C | -4.22585700 | -2.41853500 | 2.54155600 |

| | | | |
|---|---|---|---|
| H | -5.24913000 | -2.74780700 | 2.67037600 |
| C | -2.21626100 | 1.84715900 | -2.54414600 |
| C | -1.60531100 | -1.60495300 | 2.24196200 |
| C | 1.73321000 | -0.42058900 | 1.17713200 |
| C | -2.79240600 | 1.59293400 | 3.02022200 |
| H | -2.61225500 | 1.59604000 | 1.94242800 |
| H | -2.47591100 | 2.55516000 | 3.42797400 |
| H | -3.86945700 | 1.49270300 | 3.17657500 |
| C | -5.82692300 | 1.21712300 | -2.43818600 |
| H | -6.38950000 | 1.64490600 | -3.26058600 |
| C | -4.37727900 | 0.08847100 | -0.29160100 |
| H | -3.81452600 | -0.34859000 | 0.52928900 |
| C | -1.27468700 | -3.31728900 | 0.33867900 |
| H | -0.27128300 | -2.88268900 | 0.32120300 |
| C | -2.04505100 | 0.45030300 | 3.71460900 |
| H | -0.97700500 | 0.60616800 | 3.54193100 |
| C | -4.44574800 | 1.39323300 | -2.36004500 |
| C | -1.03560300 | 2.34674500 | -3.09474400 |
| H | -0.08365500 | 2.18033900 | -2.59882900 |
| C | 2.07435500 | -1.01944000 | 2.55712500 |
| H | 2.97772400 | -1.62832700 | 2.56099000 |
| H | 2.24854400 | -0.18982900 | 3.24537600 |
| C | -3.51707400 | 2.77534000 | -4.37594100 |
| H | -4.46638100 | 2.94094400 | -4.87315300 |
| C | -1.14991300 | -4.83160100 | 0.50698300 |
| H | -0.74765000 | -5.11643300 | 1.47941800 |
| H | -0.49557000 | -5.23903100 | -0.26706700 |
| H | -2.12654600 | -5.30842000 | 0.39638400 |
| C | -5.74818100 | -0.07655600 | -0.39167000 |
| H | -6.27004400 | -0.65066800 | 0.36628100 |
| C | -3.76112500 | -1.31075300 | 3.23241000 |
| H | -4.43452700 | -0.75570900 | 3.87480900 |
| C | -2.34403100 | 3.27196700 | -4.91921100 |
| H | -2.37168700 | 3.83112100 | -5.84595400 |

| | | | |
|---|---|---|---|
| C | -2.30753100 | 0.49548900 | 5.21818600 |
| H | -1.82293900 | -0.32542700 | 5.74869500 |
| H | -3.37879900 | 0.44026400 | 5.42261500 |
| H | -1.94531800 | 1.43755100 | 5.63343400 |
| C | 0.91099400 | -3.31011500 | 2.86985900 |
| H | -0.01610800 | -3.76362500 | 3.22679800 |
| H | 1.73145400 | -3.69201700 | 3.48001300 |
| H | 1.06747900 | -3.62410100 | 1.84030400 |
| C | -6.47216500 | 0.48455200 | -1.45418800 |
| H | -7.54407500 | 0.33986100 | -1.50486700 |
| C | 0.52321200 | -1.49941100 | 4.49296600 |
| H | 0.49217300 | -0.42801600 | 4.69212600 |
| H | 1.30598300 | -1.94004300 | 5.11279000 |
| H | -0.43053700 | -1.94559000 | 4.78149200 |
| C | 2.08755100 | -1.29033900 | -0.02704200 |
| C | 2.64282500 | -2.55618400 | 0.08033400 |
| C | 1.71369900 | -0.84371700 | -1.29862400 |
| C | 2.74883500 | -3.38149500 | -1.03641700 |
| H | 2.98658700 | -2.93934000 | 1.03209100 |
| C | 1.81783900 | -1.67084400 | -2.40271400 |
| H | 1.31756500 | 0.15924000 | -1.42428400 |
| C | 2.32164800 | -2.96050200 | -2.28283500 |
| H | 2.38885800 | -3.61154100 | -3.14327700 |
| C | 2.25178900 | 1.00047000 | 1.04934100 |
| C | 1.56425900 | 2.02726200 | 1.70179500 |
| C | 3.41686600 | 1.30524200 | 0.36610600 |
| C | 2.03773900 | 3.32402400 | 1.66106500 |
| H | 0.64557800 | 1.81008900 | 2.23776500 |
| C | 3.88816100 | 2.61663500 | 0.33252700 |
| H | 3.96962700 | 0.52863200 | -0.15138500 |
| C | 3.20715500 | 3.63384000 | 0.97176400 |
| H | 3.57269500 | 4.65196300 | 0.93895400 |
| C | 1.29893600 | 4.43887700 | 2.35284900 |
| C | 5.16450100 | 2.88592200 | -0.41894500 |

| | | | |
|---|---|---|---|
| C | 3.30555800 | -4.76185200 | -0.82272300 |
| C | 1.30448800 | -1.15087700 | -3.72203700 |
| F | 4.55100900 | -4.71830600 | -0.33521600 |
| F | 2.56889600 | -5.43802200 | 0.07493700 |
| F | 3.33206000 | -5.48015000 | -1.94241500 |
| F | 1.64182800 | -1.95129600 | -4.73565200 |
| F | 1.78998900 | 0.06570500 | -3.98350500 |
| F | -0.02637800 | -1.05298500 | -3.70772100 |
| F | 2.09683300 | 5.08049700 | 3.21586700 |
| F | 0.24386500 | 3.99255900 | 3.03950700 |
| F | 0.85959500 | 5.34856800 | 1.48074600 |
| F | 5.49881500 | 4.17537300 | -0.40130100 |
| F | 5.06260300 | 2.50866200 | -1.69769200 |
| F | 6.18595300 | 2.19612400 | 0.10620600 |

88

f-1-1

| | | | |
|---|---|---|---|
| Au | -0.10132700 | 1.51343000 | -0.44816000 |
| N | -0.17677800 | 3.25427900 | 0.51612000 |
| C | -0.26303800 | 5.47897100 | 1.08722000 |
| C | -1.10834700 | -1.47836900 | 1.52086600 |
| N | 0.10804100 | -1.39599900 | -0.62447500 |
| C | 0.19645600 | -2.64484300 | -1.46119900 |
| C | -1.08203900 | -1.47851700 | 2.91646200 |
| H | -2.02414200 | -1.52007400 | 3.44925500 |
| C | -0.29674600 | 6.32863700 | -1.55474300 |
| H | -0.31026100 | 6.67947800 | -2.57965900 |
| C | -0.00243100 | -0.27310800 | -1.29662500 |
| C | -0.17853900 | 3.35033200 | 1.89338800 |
| C | 1.32290900 | -1.33383300 | 1.51500800 |
| C | 0.11200500 | -1.39585800 | 3.61175700 |
| C | -0.22708500 | 4.54402600 | 0.02654100 |
| C | 0.10516300 | -1.40325100 | 0.81976200 |
| C | -0.24205900 | 5.01404700 | 3.66225700 |
| H | -0.28259600 | 6.04766100 | 3.98752800 |

| | | | |
|---|---|---|---|
| C | -0.13564400 | 2.31759900 | 2.83175500 |
| H | -0.09372000 | 1.28220000 | 2.50466200 |
| C | -0.23145900 | 4.70170200 | 2.30309000 |
| C | -0.24375200 | 4.96929100 | -1.30234900 |
| H | -0.21519500 | 4.24502800 | -2.11008400 |
| C | 0.41604400 | -2.03562900 | -2.85257500 |
| H | -0.11990200 | -2.59426500 | -3.62173900 |
| H | 1.48332900 | -2.06859200 | -3.09153100 |
| C | -0.31639900 | 6.84467900 | 0.80993500 |
| H | -0.34434000 | 7.56805100 | 1.61726700 |
| C | -0.14701200 | 2.64981400 | 4.17528800 |
| H | -0.11442300 | 1.86092800 | 4.91908600 |
| C | 1.30337400 | -1.32769300 | 2.91043800 |
| H | 2.24517500 | -1.24901400 | 3.43950500 |
| C | -0.33315800 | 7.26373500 | -0.50952400 |
| H | -0.37438900 | 8.32060000 | -0.74108700 |
| C | -1.08952600 | -3.45602200 | -1.35180400 |
| H | -1.20865200 | -3.85629400 | -0.34290800 |
| H | -1.02667500 | -4.29958000 | -2.04058200 |
| H | -1.97524400 | -2.86951700 | -1.60046200 |
| C | -0.19993200 | 3.98807800 | 4.59273400 |
| H | -0.20761800 | 4.21760900 | 5.65085400 |
| C | 1.36406800 | -3.52466200 | -1.04177500 |
| H | 2.32033800 | -3.02084600 | -1.18632600 |
| H | 1.35524400 | -4.42234600 | -1.66216000 |
| H | 1.27494500 | -3.83725900 | 0.00171900 |
| C | -0.03542200 | -0.57017200 | -2.77108000 |
| C | -1.47564200 | -0.35999700 | -3.25244000 |
| H | -1.80457500 | 0.65941500 | -3.03910700 |
| H | -1.51964400 | -0.52153600 | -4.33156200 |
| H | -2.17155600 | -1.05164900 | -2.77500800 |
| C | 0.89179400 | 0.37234000 | -3.53041100 |
| H | 0.90011100 | 0.10648800 | -4.58975900 |
| H | 0.55170900 | 1.40530800 | -3.43108900 |

| | | | |
|---|---|---|---|
| H | 1.91416700 | 0.31077300 | -3.15287500 |
| C | 2.65273800 | -1.33996900 | 0.85192500 |
| C | 3.56830700 | -2.31512700 | 1.23610200 |
| C | 3.02810700 | -0.42127900 | -0.13019600 |
| C | 4.81241800 | -2.40231900 | 0.62482900 |
| H | 3.29577400 | -3.03651600 | 1.99906100 |
| C | 4.27381100 | -0.51618700 | -0.73011900 |
| H | 2.35753300 | 0.38489900 | -0.40434100 |
| C | 5.17426400 | -1.51259000 | -0.37073100 |
| H | 6.14357600 | -1.57780600 | -0.84693900 |
| C | -2.44114300 | -1.60934100 | 0.87264200 |
| C | -3.21331300 | -2.72704800 | 1.17098900 |
| C | -2.96460100 | -0.64624300 | 0.00932500 |
| C | -4.45679800 | -2.90929200 | 0.57850500 |
| H | -2.82827400 | -3.48011900 | 1.85069200 |
| C | -4.20985300 | -0.83331100 | -0.56790500 |
| H | -2.40409800 | 0.25757900 | -0.19852400 |
| C | -4.96371800 | -1.97102400 | -0.30256200 |
| H | -5.93308700 | -2.10967800 | -0.76359200 |
| C | -5.21643500 | -4.16725300 | 0.90196900 |
| C | -4.77895000 | 0.18697000 | -1.51942900 |
| F | -6.42540900 | -4.18615100 | 0.34317400 |
| F | -4.55577000 | -5.25112100 | 0.47063000 |
| F | -5.37459600 | -4.31442200 | 2.22191700 |
| F | -4.85034400 | -0.30935600 | -2.76513700 |
| F | -4.04416100 | 1.29533700 | -1.57328900 |
| F | -6.02174800 | 0.53389100 | -1.17374200 |
| C | 5.73606400 | -3.51140400 | 1.05144100 |
| C | 4.66829400 | 0.44423800 | -1.82146600 |
| F | 5.23773700 | -4.70884800 | 0.71371300 |
| F | 5.90037400 | -3.52376300 | 2.37856600 |
| F | 6.94122200 | -3.40756300 | 0.49328300 |
| F | 5.94426900 | 0.81863400 | -1.70920700 |
| F | 3.91601600 | 1.54271300 | -1.82387000 |

| | | | |
|---|---|---|---|
| F | 4.53824800 | -0.13020600 | -3.02986200 |
| H | 0.11330900 | -1.37284600 | 4.69344800 |

94

f-2-1

| | | | |
|---|---|---|---|
| Au | -0.60256000 | 0.88291700 | -0.60111600 |
| N | -1.70008400 | 2.41159600 | 0.07438000 |
| C | -3.34725600 | 3.98726200 | 0.37525900 |
| C | -0.20013100 | -1.56027200 | 1.95001400 |
| N | 1.01596000 | -1.50739900 | -0.20504300 |
| C | 1.73936700 | -2.70266000 | -0.77643500 |
| C | -0.21757000 | -1.33408700 | 3.32534300 |
| H | -1.12121700 | -1.57097500 | 3.87311200 |
| C | -3.97270500 | 4.09004400 | -2.32635200 |
| H | -4.23296500 | 4.14285100 | -3.37668500 |
| C | 0.44345000 | -0.72359000 | -1.08741600 |
| C | -1.77942800 | 2.70686400 | 1.41980400 |
| C | 2.03138500 | -0.59914500 | 1.85807600 |
| C | 0.87651600 | -0.79252100 | 3.97921700 |
| C | -2.65245100 | 3.18512500 | -0.55921800 |
| C | 0.95322100 | -1.23239400 | 1.21620600 |
| C | -3.03266700 | 4.09515200 | 2.97045100 |
| H | -3.80015400 | 4.83461900 | 3.17009400 |
| C | -1.02530900 | 2.17374400 | 2.46885100 |
| H | -0.23770600 | 1.45008700 | 2.26944400 |
| C | -2.78362500 | 3.67213600 | 1.66598300 |
| C | -2.96707200 | 3.23272400 | -1.91762000 |
| H | -2.43330200 | 2.60647400 | -2.62504700 |
| C | 1.42113300 | -2.57326700 | -2.27552400 |
| H | 0.78256000 | -3.39896500 | -2.59353300 |
| H | 2.33986000 | -2.62886300 | -2.86239400 |
| C | -4.35782700 | 4.84498200 | -0.05883200 |
| H | -4.89597800 | 5.46344400 | 0.65085500 |
| C | -1.29551200 | 2.60350600 | 3.75785300 |
| H | -0.71851400 | 2.20161100 | 4.58373100 |

| | | | |
|---|---|---|---|
| C | 1.98216400 | -0.41134600 | 3.24077900 |
| H | 2.81679100 | 0.08374100 | 3.72234800 |
| C | -4.66485600 | 4.89388000 | -1.40770500 |
| H | -5.44668000 | 5.55438600 | -1.76092300 |
| C | 1.18492900 | -3.99180600 | -0.19651200 |
| H | 1.25396700 | -4.00186400 | 0.89450000 |
| H | 1.77106500 | -4.82839400 | -0.57990200 |
| H | 0.14868200 | -4.13776200 | -0.50118800 |
| C | -2.29130600 | 3.55709500 | 4.01139000 |
| H | -2.47755600 | 3.87711100 | 5.02881700 |
| C | 3.23678200 | -2.63741800 | -0.51245600 |
| H | 3.68130900 | -1.72409600 | -0.91376900 |
| H | 3.70171600 | -3.49081400 | -1.00849500 |
| H | 3.45908400 | -2.70891000 | 0.55368900 |
| C | 0.71869400 | -1.22085700 | -2.47791300 |
| C | -0.59151200 | -1.33125500 | -3.26783100 |
| C | 1.66864400 | -0.18529100 | -3.11503400 |
| H | 1.23481100 | 0.81138800 | -2.98603500 |
| H | -0.36670500 | -1.79911600 | -4.23051800 |
| C | -1.67488800 | -2.11360200 | -2.53788400 |
| H | -1.34002600 | -3.11749600 | -2.25808000 |
| H | -1.97285000 | -1.59388700 | -1.62380500 |
| H | -2.56444800 | -2.22659800 | -3.15756400 |
| H | -0.94790400 | -0.31620500 | -3.47732300 |
| C | 1.96332200 | -0.43713100 | -4.58599300 |
| H | 1.08093300 | -0.26657100 | -5.20319700 |
| H | 2.74599600 | 0.23872200 | -4.93091300 |
| H | 2.30429500 | -1.46037700 | -4.76237800 |
| H | 2.60458500 | -0.19665000 | -2.54344200 |
| H | 0.84793100 | -0.62623400 | 5.04789400 |
| C | 3.21337100 | -0.04491300 | 1.14946300 |
| C | 4.49319400 | -0.39207100 | 1.56664500 |
| C | 3.07288600 | 0.86506200 | 0.10013100 |
| C | 5.61085200 | 0.11315600 | 0.91428400 |

| | | | |
|---|---|---|---|
| H | 4.62199000 | -1.08993700 | 2.38749400 |
| C | 4.19459100 | 1.36889600 | -0.53758200 |
| H | 2.08430400 | 1.18912900 | -0.20663100 |
| C | 5.47435300 | 0.98892600 | -0.14831600 |
| H | 6.34463500 | 1.38496800 | -0.65530300 |
| C | -1.48850800 | -1.99673500 | 1.33426900 |
| C | -1.79375100 | -3.31599100 | 1.04016300 |
| C | -2.47133200 | -1.02166800 | 1.13593800 |
| C | -3.02089200 | -3.64426300 | 0.46960800 |
| H | -1.08994400 | -4.10487800 | 1.26974800 |
| C | -3.68592000 | -1.35789400 | 0.56163500 |
| H | -2.28456600 | 0.00268500 | 1.44123400 |
| C | -3.96836500 | -2.67180700 | 0.20633500 |
| H | -4.91398800 | -2.92811600 | -0.25209900 |
| C | 6.96743000 | -0.34276100 | 1.37942300 |
| C | 4.05972100 | 2.33497500 | -1.68612800 |
| C | -3.25732700 | -5.08514300 | 0.11677600 |
| C | -4.66364300 | -0.26306200 | 0.22104300 |
| F | -3.12754800 | -5.88300500 | 1.18347600 |
| F | -4.46530200 | -5.29459500 | -0.40172900 |
| F | -5.92614200 | -0.70591400 | 0.24913400 |
| F | -4.57096500 | 0.76697300 | 1.05600400 |
| F | -2.35389800 | -5.50946500 | -0.78533600 |
| F | -4.43606400 | 0.19455800 | -1.01700900 |
| F | 7.12662800 | -1.65877600 | 1.18089000 |
| F | 7.12865100 | -0.13033800 | 2.69022800 |
| F | 7.95699700 | 0.28107700 | 0.74210700 |
| F | 4.84732500 | 3.40097300 | -1.51949200 |
| F | 2.81102400 | 2.76808400 | -1.83999000 |
| F | 4.43131100 | 1.76067100 | -2.84295400 |

103

f-3-1

| | | | |
|---|---|---|---|
| Au | -0.56658900 | -0.78730900 | 0.53667000 |
| N | -1.59980900 | -2.49483000 | 0.44396900 |

| | | | |
|---|---|---|---|
| C | -3.22802000 | -4.09744200 | 0.70256700 |
| C | 0.06715700 | 0.40033500 | -2.65128700 |
| N | 1.16281100 | 1.23009300 | -0.59665600 |
| C | 1.91490200 | 2.53241200 | -0.50533500 |
| C | 0.12030100 | -0.36531300 | -3.81485100 |
| H | -0.74576300 | -0.37334100 | -4.46504300 |
| C | -4.11823400 | -3.04498200 | 3.11087100 |
| H | -4.48292100 | -2.64643500 | 4.04995200 |
| C | 0.43274100 | 0.92558600 | 0.45819500 |
| C | -1.55616300 | -3.33888400 | -0.64560200 |
| C | 2.26817700 | -0.44177600 | -2.04423300 |
| C | 1.23517500 | -1.12716300 | -4.12465900 |
| C | -2.61727700 | -2.95002600 | 1.25981100 |
| C | 1.17279800 | 0.39842100 | -1.78084000 |
| C | -2.67143200 | -5.29467700 | -1.55678700 |
| H | -3.42348900 | -6.07278300 | -1.48842500 |
| C | -0.70354500 | -3.27845600 | -1.75032200 |
| H | 0.06579900 | -2.51225400 | -1.80667900 |
| C | -2.54186700 | -4.34760800 | -0.54230700 |
| C | -3.06691400 | -2.41595000 | 2.46805600 |
| H | -2.60434000 | -1.52417700 | 2.88112100 |
| C | 1.71941600 | 2.84667600 | 0.97379500 |
| H | 1.66525500 | 3.92143500 | 1.13620200 |
| H | 2.58336700 | 2.47007000 | 1.53505000 |
| C | -4.28618600 | -4.71590200 | 1.36773200 |
| H | -4.76002700 | -5.59588400 | 0.94719500 |
| C | -0.85539100 | -4.22533800 | -2.74993400 |
| H | -0.20093300 | -4.19559900 | -3.61445100 |
| C | 2.28865700 | -1.17857700 | -3.22974400 |
| H | 3.13257100 | -1.83137900 | -3.41743000 |
| C | -4.72471500 | -4.18889900 | 2.57044800 |
| H | -5.54519600 | -4.65725500 | 3.09930000 |
| C | 1.30911200 | 3.58732200 | -1.42050800 |
| H | 1.33355400 | 3.25307200 | -2.46086500 |

| | | | |
|---|---|---|---|
| H | 1.91136000 | 4.49402900 | -1.34918700 |
| H | 0.28802700 | 3.83975500 | -1.14108600 |
| C | -1.83100000 | -5.22815800 | -2.65760700 |
| H | -1.92396100 | -5.95734000 | -3.45255600 |
| C | 3.39137400 | 2.40506000 | -0.85165400 |
| H | 3.91478900 | 1.72556600 | -0.17973500 |
| H | 3.83910200 | 3.39435900 | -0.74220700 |
| H | 3.54051100 | 2.08501700 | -1.88543300 |
| C | 3.39537300 | -0.62438800 | -1.09847600 |
| C | 4.70486400 | -0.54812100 | -1.55846800 |
| C | 3.17490700 | -0.86628500 | 0.25919900 |
| C | 5.77141000 | -0.65194200 | -0.67362400 |
| H | 4.89894200 | -0.36029100 | -2.60920000 |
| C | 4.24445200 | -0.95370200 | 1.13304700 |
| H | 2.16388400 | -0.99244900 | 0.62862700 |
| C | 5.55451900 | -0.83674500 | 0.68052500 |
| H | 6.38496800 | -0.90239400 | 1.37092800 |
| C | -1.24504200 | 1.03258800 | -2.32149800 |
| C | -1.52816900 | 2.37902500 | -2.47607100 |
| C | -2.26490300 | 0.18068000 | -1.87837200 |
| C | -2.76935500 | 2.88630300 | -2.09424200 |
| H | -0.79703200 | 3.04813400 | -2.90687600 |
| C | -3.49411800 | 0.69401400 | -1.50364900 |
| H | -2.08831600 | -0.88929200 | -1.83342100 |
| C | -3.75155800 | 2.05863800 | -1.58389100 |
| H | -4.70996600 | 2.45729600 | -1.27848200 |
| C | -2.98264000 | 4.36866100 | -2.21094800 |
| C | -4.54133000 | -0.21995100 | -0.92142600 |
| F | -4.23554700 | 4.72929800 | -1.94245100 |
| F | -2.18418900 | 5.03610900 | -1.35616700 |
| F | -2.68116800 | 4.81706200 | -3.43479400 |
| F | -5.77268500 | 0.15390200 | -1.29261400 |
| F | -4.51056500 | -0.18066100 | 0.41658500 |
| F | -4.36701600 | -1.48305100 | -1.29514800 |

| | | | |
|---|---|---|---|
| C | 7.16445500 | -0.52796600 | -1.22968400 |
| C | 4.00120300 | -1.11048500 | 2.61066900 |
| F | 7.33303600 | 0.64794800 | -1.84955000 |
| F | 7.41120600 | -1.47925600 | -2.13738500 |
| F | 8.09721100 | -0.62040700 | -0.28281600 |
| F | 4.86301500 | -1.96408100 | 3.16729000 |
| F | 2.76823100 | -1.53621400 | 2.87867800 |
| F | 4.15996900 | 0.06677200 | 3.24102800 |
| H | 1.25944100 | -1.71500900 | -5.03264200 |
| C | 0.47735700 | 2.06877600 | 1.44645000 |
| C | -0.85870000 | 2.86433900 | 1.32903900 |
| C | 0.63433900 | 1.65843000 | 2.92633300 |
| H | -1.03766300 | 3.11967600 | 0.28000000 |
| C | -0.77058500 | 4.13934800 | 2.17155600 |
| C | -2.04668000 | 2.04497100 | 1.84790800 |
| H | 1.54955500 | 1.06181900 | 3.02136500 |
| C | 0.75868700 | 2.94070600 | 3.75914800 |
| C | -0.55590000 | 0.86560500 | 3.46423600 |
| H | -1.72097700 | 4.67602500 | 2.08189700 |
| H | 0.00488100 | 4.81628000 | 1.80451200 |
| C | -0.52216000 | 3.77207800 | 3.63607100 |
| H | -2.19819600 | 1.13921300 | 1.25281300 |
| H | -2.95368400 | 2.65107200 | 1.73891100 |
| C | -1.83887600 | 1.68115700 | 3.31772000 |
| H | 1.62692400 | 3.52989400 | 3.44860200 |
| H | 0.91800600 | 2.66585000 | 4.80642200 |
| H | -0.64836700 | -0.09796800 | 2.95558700 |
| H | -0.37457500 | 0.64867500 | 4.52245400 |
| H | -0.41901200 | 4.68599300 | 4.22741000 |
| C | -1.71027600 | 2.95746700 | 4.14774700 |
| H | -2.69091600 | 1.09109800 | 3.66708900 |
| H | -1.56414500 | 2.70285400 | 5.20274300 |
| H | -2.62746700 | 3.55125300 | 4.07286100 |

95

f-4-1

| | | | |
|---|---|---|---|
| Au | 0.06205600 | 1.50488900 | 0.20707300 |
| N | 0.10194700 | 3.24363000 | -0.76411400 |
| C | 0.15735700 | 5.46901600 | -1.33618800 |
| C | 1.07532100 | -1.47557800 | -1.76398200 |
| N | -0.10429300 | -1.40564300 | 0.40194100 |
| C | -0.15780400 | -2.65128400 | 1.24286800 |
| C | 1.02336900 | -1.48136600 | -3.15887200 |
| H | 1.95601400 | -1.50985100 | -3.70890800 |
| C | 0.24708500 | 6.31789700 | 1.30489300 |
| H | 0.28214200 | 6.66860400 | 2.32937600 |
| C | -0.00036400 | -0.27897300 | 1.06898900 |
| C | 0.07071000 | 3.33982900 | -2.14072600 |
| C | -1.35776200 | -1.36677700 | -1.71480500 |
| C | -0.18434500 | -1.42115900 | -3.83264000 |
| C | 0.15370000 | 4.53335300 | -0.27539600 |
| C | -0.12678900 | -1.41687700 | -1.04154500 |
| C | 0.07923000 | 5.00426500 | -3.91018600 |
| H | 0.10377500 | 6.03820100 | -4.23607200 |
| C | 0.01398800 | 2.30682500 | -3.07813000 |
| H | -0.01185200 | 1.27117800 | -2.75019500 |
| C | 0.10326100 | 4.69173100 | -2.55119900 |
| C | 0.19856000 | 4.95827800 | 1.05306100 |
| H | 0.19489300 | 4.23312100 | 1.86067800 |
| C | -0.33486800 | -2.04642000 | 2.64300100 |
| H | 0.25207100 | -2.59522700 | 3.38096100 |
| H | -1.38696000 | -2.11616500 | 2.93747400 |
| C | 0.20674800 | 6.83497600 | -1.05935400 |
| H | 0.21000100 | 7.55891200 | -1.86667100 |
| C | -0.00901900 | 2.63932300 | -4.42140200 |
| H | -0.05297900 | 1.85032300 | -5.16448200 |
| C | -1.36392200 | -1.36765700 | -3.11030700 |
| H | -2.31623300 | -1.30508500 | -3.62247100 |
| C | 0.25152000 | 7.25366600 | 0.25959200 |

| | | | |
|---|---|---|---|
| H | 0.29014000 | 8.31073700 | 0.49071500 |
| C | 1.13159600 | -3.45094000 | 1.09853100 |
| H | 1.23400500 | -3.84263600 | 0.08443900 |
| H | 1.09104600 | -4.29987600 | 1.78243000 |
| H | 2.01546000 | -2.85589800 | 1.33532000 |
| C | 0.02330400 | 3.97806500 | -4.83964100 |
| H | 0.00403900 | 4.20771000 | -5.89759700 |
| C | -1.33164900 | -3.53986700 | 0.86139200 |
| H | -2.28583400 | -3.03903600 | 1.02931700 |
| H | -1.30146200 | -4.43339600 | 1.48709500 |
| H | -1.27017600 | -3.85851500 | -0.18228900 |
| C | -2.67467000 | -1.38114100 | -1.02622300 |
| C | -3.58699700 | -2.37037800 | -1.37993000 |
| C | -3.04100700 | -0.45278100 | -0.04953800 |
| C | -4.81825200 | -2.46201600 | -0.74315000 |
| H | -3.32187000 | -3.09885800 | -2.13877100 |
| C | -4.27374400 | -0.55155700 | 0.57522700 |
| H | -2.37336700 | 0.36312400 | 0.20167700 |
| C | -5.17033800 | -1.56233800 | 0.24679500 |
| H | -6.12981100 | -1.63068100 | 0.74236400 |
| C | 2.42144500 | -1.58514600 | -1.13917600 |
| C | 3.20673400 | -2.68924700 | -1.45431700 |
| C | 2.94348500 | -0.61806700 | -0.27939400 |
| C | 4.46177100 | -2.85460600 | -0.88185600 |
| H | 2.82299800 | -3.44569200 | -2.13102500 |
| C | 4.19991800 | -0.78870100 | 0.27911400 |
| H | 2.37241100 | 0.27608800 | -0.05856300 |
| C | 4.96726700 | -1.91306000 | -0.00326300 |
| H | 5.94553500 | -2.03900700 | 0.44219000 |
| C | 5.23680900 | -4.09774500 | -1.22530400 |
| C | 4.76348500 | 0.23427000 | 1.23126400 |
| F | 6.44940800 | -4.10663700 | -0.67390900 |
| F | 4.59407300 | -5.19613500 | -0.80387900 |
| F | 5.38933200 | -4.22635000 | -2.54791800 |

| | | | |
|---|---|---|---|
| F | 4.81539500 | -0.25263800 | 2.48299400 |
| F | 4.03602000 | 1.34778800 | 1.26812900 |
| F | 6.01338300 | 0.56992900 | 0.90070700 |
| C | -5.73764100 | -3.58724400 | -1.13491800 |
| C | -4.66341400 | 0.42349700 | 1.65543100 |
| F | -5.22604200 | -4.77329900 | -0.77708500 |
| F | -5.91715200 | -3.62992900 | -2.45946900 |
| F | -6.93718800 | -3.48089700 | -0.56507900 |
| F | -5.93098900 | 0.82077400 | 1.52262000 |
| F | -3.89150700 | 1.50794900 | 1.66098800 |
| F | -4.56238500 | -0.14493800 | 2.86929600 |
| H | -0.20546200 | -1.40326400 | -4.91423100 |
| C | 0.06496000 | -0.56623100 | 2.54076200 |
| C | 1.51255800 | -0.29444400 | 2.99579900 |
| C | -0.88200400 | 0.35792400 | 3.31222100 |
| C | 1.67344000 | -0.43411900 | 4.50459900 |
| H | 1.77726000 | 0.72564500 | 2.69134900 |
| H | 2.19629700 | -0.97108300 | 2.47487000 |
| C | -0.73617600 | 0.16157300 | 4.81479500 |
| H | -0.64010900 | 1.39580500 | 3.05195100 |
| H | -1.91060000 | 0.17642600 | 2.98619600 |
| C | 0.69327900 | 0.46528800 | 5.24676500 |
| H | 2.70454700 | -0.19743700 | 4.77513500 |
| H | 1.50277400 | -1.47474500 | 4.80378800 |
| H | -1.44608400 | 0.80127600 | 5.34159500 |
| H | -0.98654000 | -0.87369500 | 5.07923200 |
| H | 0.80638300 | 0.34343000 | 6.32569500 |
| H | 0.91962600 | 1.51256600 | 5.01534500 |

93

f-5-1

| | | | |
|---|---|---|---|
| Au | 0.09804100 | 1.53712400 | 0.24000200 |
| N | 0.15440300 | 3.29348700 | -0.69702300 |
| C | 0.22521400 | 5.52909000 | -1.22647700 |
| C | 1.06285600 | -1.42783600 | -1.77160300 |

| | | | |
|---|---|---|---|
| N | -0.11971400 | -1.37500500 | 0.39018500 |
| C | -0.20206800 | -2.62954400 | 1.21896600 |
| C | 1.01749500 | -1.39972800 | -3.16628400 |
| H | 1.95176400 | -1.43407700 | -3.71321100 |
| C | 0.32500700 | 6.32658800 | 1.43011300 |
| H | 0.36451300 | 6.65725000 | 2.46105200 |
| C | 0.01318700 | -0.25849200 | 1.07005000 |
| C | 0.12177800 | 3.41643200 | -2.07164000 |
| C | -1.36816000 | -1.27880700 | -1.72906100 |
| C | -0.18619800 | -1.29937800 | -3.84281200 |
| C | 0.21636700 | 4.57351600 | -0.18377300 |
| C | -0.14082400 | -1.36322600 | -1.05298500 |
| C | 0.13999700 | 5.11412900 | -3.80885000 |
| H | 0.17172600 | 6.15385600 | -4.11507600 |
| C | 0.05649600 | 2.40194900 | -3.02834000 |
| H | 0.02415400 | 1.36056800 | -2.71974700 |
| C | 0.16363300 | 4.77559700 | -2.45611400 |
| C | 0.26640400 | 4.97239700 | 1.15245700 |
| H | 0.25922200 | 4.23221800 | 1.94616000 |
| C | -0.39178200 | -2.02828200 | 2.61839300 |
| H | 0.14351600 | -2.59520400 | 3.38239500 |
| H | -1.45839500 | -2.04490500 | 2.86874900 |
| C | 0.28475800 | 6.88915400 | -0.92375100 |
| H | 0.29206700 | 7.62833000 | -1.71709800 |
| C | 0.03396700 | 2.76004100 | -4.36504200 |
| H | -0.01643000 | 1.98580000 | -5.12308300 |
| C | -1.36796000 | -1.24408500 | -3.12418100 |
| H | -2.31666300 | -1.15343900 | -3.63884400 |
| C | 0.33442900 | 7.28212000 | 0.40289800 |
| H | 0.38098100 | 8.33427100 | 0.65413200 |
| C | 1.07324600 | -3.45295600 | 1.07791000 |
| H | 1.16584600 | -3.84943000 | 0.06455800 |
| H | 1.01654300 | -4.29897100 | 1.76411300 |
| H | 1.97071100 | -2.87768800 | 1.30916300 |

| | | | |
|---|---|---|---|
| C | 0.07529700 | 4.10625700 | -4.75762700 |
| H | 0.05634700 | 4.35623800 | -5.81096800 |
| C | -1.38375500 | -3.49856300 | 0.81535500 |
| H | -2.33463400 | -2.99179300 | 0.98340000 |
| H | -1.36688900 | -4.40155700 | 1.42779900 |
| H | -1.31695600 | -3.80292200 | -0.23223700 |
| C | -2.68591200 | -1.30498100 | -1.04327500 |
| C | -3.60544500 | -2.27224600 | -1.43810000 |
| C | -3.04166900 | -0.42415200 | -0.01993900 |
| C | -4.83185400 | -2.39127900 | -0.79747700 |
| H | -3.34740100 | -2.96614200 | -2.23104500 |
| C | -4.26849100 | -0.55347900 | 0.61210800 |
| H | -2.36943300 | 0.37665600 | 0.26593800 |
| C | -5.17152400 | -1.54323300 | 0.24134400 |
| H | -6.12496300 | -1.63657300 | 0.74381400 |
| C | 2.40181800 | -1.58403400 | -1.14116600 |
| C | 3.15457100 | -2.70721900 | -1.46775400 |
| C | 2.94794000 | -0.64499700 | -0.26514100 |
| C | 4.40009400 | -2.91964600 | -0.89027900 |
| H | 2.75172600 | -3.44143600 | -2.15766400 |
| C | 4.19636600 | -0.86137700 | 0.29612400 |
| H | 2.40342200 | 0.26391300 | -0.03716500 |
| C | 4.92952000 | -2.00577200 | 0.00321800 |
| H | 5.90147400 | -2.16727200 | 0.45090400 |
| C | 5.13773600 | -4.18230200 | -1.24499600 |
| C | 4.78874600 | 0.13310500 | 1.26094100 |
| F | 6.34887400 | -4.23284000 | -0.69276900 |
| F | 4.46161200 | -5.26450400 | -0.83420200 |
| F | 5.28750300 | -4.30290100 | -2.56860000 |
| F | 4.78822900 | -0.35404600 | 2.51376700 |
| F | 4.11665200 | 1.28155500 | 1.28116400 |
| F | 6.06046100 | 0.40614200 | 0.95840600 |
| C | -5.76249800 | -3.48441000 | -1.24932700 |
| C | -4.62797700 | 0.35339600 | 1.75943900 |

| | | | |
|---|---|---|---|
| F | -5.20486800 | -4.69103400 | -1.07973000 |
| F | -6.04737300 | -3.37230700 | -2.55131500 |
| F | -6.91411700 | -3.47649000 | -0.57971700 |
| F | -5.92401700 | 0.66971700 | 1.75215000 |
| F | -3.92810100 | 1.48602900 | 1.74667300 |
| F | -4.38350500 | -0.25114500 | 2.93624600 |
| H | -0.20233700 | -1.25365400 | -4.92367100 |
| C | 0.07826700 | -0.57011200 | 2.53525700 |
| C | 1.53240400 | -0.38006700 | 2.99613900 |
| C | -0.80041400 | 0.38159800 | 3.34889900 |
| C | 1.64027300 | -0.41506000 | 4.49184300 |
| H | 1.91124900 | 0.57374400 | 2.60694600 |
| H | 2.16832200 | -1.15842300 | 2.56314800 |
| C | -0.79632700 | -0.02636700 | 4.81559100 |
| H | -0.40058100 | 1.39508400 | 3.23745300 |
| H | -1.81761700 | 0.38785000 | 2.94913600 |
| C | 0.59749700 | -0.28136600 | 5.30637200 |
| H | 2.63324400 | -0.56475000 | 4.90214800 |
| H | -1.26340200 | 0.75595400 | 5.41809400 |
| H | -1.41446700 | -0.92026100 | 4.96636700 |
| H | 0.74810700 | -0.34327400 | 6.37881800 |

107

f-6-1

| | | | |
|---|---|---|---|
| Au | -0.48277500 | 1.02065500 | 0.38919200 |
| N | -1.54630600 | 2.64393500 | -0.07852500 |
| C | -3.25050300 | 4.18701800 | -0.10004800 |
| C | 2.03912000 | -0.36605900 | -2.22021600 |
| N | 1.00836300 | -1.41006200 | -0.22774000 |
| C | 1.67917200 | -2.67333500 | 0.25251000 |
| C | 1.97105400 | -0.03761900 | -3.57568300 |
| H | 2.81994400 | 0.46230400 | -4.02639600 |
| C | -3.62082200 | 4.05915200 | 2.64814100 |
| H | -3.78209200 | 4.02229400 | 3.71882400 |
| C | 0.48530900 | -0.66763700 | 0.72068200 |

| | | | |
|---|---|---|---|
| C | -1.76719100 | 3.02900300 | -1.38417300 |
| C | -0.23468100 | -1.21301500 | -2.35304700 |
| C | 0.83205200 | -0.28698800 | -4.31971800 |
| C | -2.44944300 | 3.33921600 | 0.69960500 |
| C | 0.94539500 | -1.00867100 | -1.61750900 |
| C | -3.19651200 | 4.48761300 | -2.69978200 |
| H | -3.99514500 | 5.21762700 | -2.76999800 |
| C | -1.11075900 | 2.58985000 | -2.53727100 |
| H | -0.29422900 | 1.87439700 | -2.46622700 |
| C | -2.81067800 | 3.98092300 | -1.45957800 |
| C | -2.63630600 | 3.26928200 | 2.08086800 |
| H | -2.01847000 | 2.61158000 | 2.68511900 |
| C | 1.22258200 | -2.70886400 | 1.71969500 |
| H | 2.04001100 | -3.02329200 | 2.36991400 |
| H | 0.41915400 | -3.43781400 | 1.82611800 |
| C | -4.23520800 | 4.97625300 | 0.49327800 |
| H | -4.85415500 | 5.62969700 | -0.11141100 |
| C | -1.51652700 | 3.10128800 | -3.75887400 |
| H | -1.01762300 | 2.77413600 | -4.66459900 |
| C | -0.27200700 | -0.84480300 | -3.69688400 |
| H | -1.19567300 | -0.98901700 | -4.24364800 |
| C | -4.41505000 | 4.90980100 | 1.86456900 |
| H | -5.17589700 | 5.51578500 | 2.34009200 |
| C | 3.19507500 | -2.59132500 | 0.11581300 |
| H | 3.49525000 | -2.59899300 | -0.93322000 |
| H | 3.62358000 | -3.47573500 | 0.58966200 |
| H | 3.61184000 | -1.70540100 | 0.59749400 |
| C | -2.55149000 | 4.04369400 | -3.84381000 |
| H | -2.84459200 | 4.42842300 | -4.81246100 |
| C | 1.19070900 | -3.89240100 | -0.50945000 |
| H | 0.14922100 | -4.10596900 | -0.27442800 |
| H | 1.78543200 | -4.75473100 | -0.20402900 |
| H | 1.31114000 | -3.76630400 | -1.58884100 |
| C | -1.51742000 | -1.69999800 | -1.76530600 |

| | | | |
|---|---|---|---|
| C | -1.80109200 | -3.04779600 | -1.60153600 |
| C | -2.51275200 | -0.76381300 | -1.46979200 |
| C | -3.01307700 | -3.45079400 | -1.05074100 |
| H | -1.08656200 | -3.79546000 | -1.91668200 |
| C | -3.73098900 | -1.17911700 | -0.95323100 |
| H | -2.33694400 | 0.29080600 | -1.66147200 |
| C | -3.98349200 | -2.52373500 | -0.71294000 |
| H | -4.92388400 | -2.83919300 | -0.28123600 |
| C | 3.25995200 | 0.06332500 | -1.49129700 |
| C | 4.51166600 | -0.31922400 | -1.95786300 |
| C | 3.18373500 | 0.90123000 | -0.37672100 |
| C | 5.66580300 | 0.08036600 | -1.29482500 |
| H | 4.58910300 | -0.96205600 | -2.82861400 |
| C | 4.34124700 | 1.30366400 | 0.26733400 |
| H | 2.21805100 | 1.24698600 | -0.02428000 |
| C | 5.59309700 | 0.88783000 | -0.17438000 |
| H | 6.49093200 | 1.20206900 | 0.34205100 |
| C | 6.98814800 | -0.40687000 | -1.82222800 |
| C | 4.28766700 | 2.21220000 | 1.46733500 |
| F | 8.01831800 | 0.07310400 | -1.12730600 |
| F | 7.05992600 | -1.74447900 | -1.77904500 |
| F | 7.16027400 | -0.05461500 | -3.10138200 |
| F | 4.85290900 | 1.62477200 | 2.53362400 |
| F | 3.04273100 | 2.54162000 | 1.80345200 |
| F | 4.96525800 | 3.34348600 | 1.25013600 |
| C | -3.23940900 | -4.92264200 | -0.84685200 |
| C | -4.80648100 | -0.15206200 | -0.70073800 |
| F | -2.19177900 | -5.48977900 | -0.22531900 |
| F | -3.38459100 | -5.56804700 | -2.01010400 |
| F | -4.32223100 | -5.17185200 | -0.11076900 |
| F | -5.45461500 | 0.14498000 | -1.83402600 |
| F | -4.30649600 | 0.98205800 | -0.21982500 |
| F | -5.72077400 | -0.60071400 | 0.16726000 |
| H | 0.78943000 | -0.01077000 | -5.36491300 |

| | | | |
|---|---|---|---|
| C | 0.70890400 | -1.30425500 | 2.06200500 |
| C | 1.76684900 | -0.45552600 | 2.79640400 |
| C | -0.59820100 | -1.25858800 | 2.87869200 |
| C | 2.00954400 | -0.92616100 | 4.22771900 |
| H | 1.41662700 | 0.58489800 | 2.82069800 |
| H | 2.70940800 | -0.45901800 | 2.23414900 |
| C | -0.35719900 | -1.77351400 | 4.29185100 |
| H | -0.81770800 | -0.18240400 | 2.96817000 |
| C | 0.69584900 | -0.93230600 | 4.99818300 |
| H | 2.38713800 | -1.95725000 | 4.20165800 |
| H | -1.29704600 | -1.75534600 | 4.85098300 |
| H | -0.03403700 | -2.82238600 | 4.25543300 |
| H | 0.86579800 | -1.29338900 | 6.01607400 |
| H | 0.33682500 | 0.10208400 | 5.08060400 |
| C | 3.04803800 | -0.04532900 | 4.90531600 |
| H | 4.01103700 | -0.09633700 | 4.39565800 |
| H | 2.72108500 | 0.99845300 | 4.89557200 |
| H | 3.19395900 | -0.34361700 | 5.94530800 |
| C | -1.82865200 | -1.88972200 | 2.18007600 |
| H | -1.63558900 | -1.90657600 | 1.09635900 |
| C | -3.03771600 | -0.99122600 | 2.43129700 |
| H | -3.17139600 | -0.84329100 | 3.50814800 |
| H | -2.90771400 | -0.00726400 | 1.96909300 |
| H | -3.95680200 | -1.43646300 | 2.04440100 |
| C | -2.18080800 | -3.31297600 | 2.61358800 |
| H | -2.47034300 | -3.33806900 | 3.66683400 |
| H | -3.03872000 | -3.66592700 | 2.03534100 |
| H | -1.37486000 | -4.03513200 | 2.47329500 |

74

g-1-1

| | | | |
|---|---|---|---|
| Au | 1.06841900 | 0.06866800 | 1.10852700 |
| N | 2.84680900 | 0.08005800 | 0.19300700 |
| C | 5.07428700 | 0.13564200 | -0.37340100 |
| C | -1.61519300 | -1.31212400 | -1.07299100 |

| | | | |
|---|---|---|---|
| N | -1.82881500 | -0.03988300 | 1.06335700 |
| C | -3.14755000 | -0.04889500 | 1.80552400 |
| C | -1.67566900 | -1.31886800 | -2.46675800 |
| H | -1.63458200 | -2.25955600 | -2.99633400 |
| C | 5.91415300 | 0.20244200 | 2.27101600 |
| H | 6.26155300 | 0.22912500 | 3.29697900 |
| C | -0.76184600 | 0.07850500 | 1.82044000 |
| C | 2.94701800 | 0.06477400 | -1.18217100 |
| C | -1.75538200 | 1.10621500 | -1.11949500 |
| C | -1.78931100 | -0.14828800 | -3.18684700 |
| C | 4.13397200 | 0.12522200 | 0.68423900 |
| C | -1.72768900 | -0.09299600 | -0.37434700 |
| C | 4.61714300 | 0.08818700 | -2.94887100 |
| H | 5.65243000 | 0.11214700 | -3.27078700 |
| C | 1.91807300 | 0.03012900 | -2.12761900 |
| H | 0.87990200 | 0.01161200 | -1.80612000 |
| C | 4.30082000 | 0.09618100 | -1.59071200 |
| C | 4.55542500 | 0.15826000 | 2.01456600 |
| H | 3.82728900 | 0.14913900 | 2.81892000 |
| C | -2.69270800 | 0.50145300 | 3.15997200 |
| H | -3.25241000 | 0.04630100 | 3.97896400 |
| H | -2.86557000 | 1.58088900 | 3.18507500 |
| C | 6.43957000 | 0.17971000 | -0.09158400 |
| H | 7.16629900 | 0.18820400 | -0.89647100 |
| C | 2.25436800 | 0.02241200 | -3.47021000 |
| H | 1.46682100 | -0.00440800 | -4.21607100 |
| C | -1.80134300 | 1.05513000 | -2.51162900 |
| H | -1.85375300 | 1.97391100 | -3.07779000 |
| C | 6.85398400 | 0.21314000 | 1.22894400 |
| H | 7.91037300 | 0.24792600 | 1.46402100 |
| C | -3.71695800 | -1.45867400 | 1.92704700 |
| H | -3.94204900 | -1.87732200 | 0.94702700 |
| H | -4.65611100 | -1.39123800 | 2.47839500 |
| H | -3.05570100 | -2.13480000 | 2.46099200 |

| | | | |
|---|---|---|---|
| C | 3.59462300 | 0.05046300 | -3.88356100 |
| H | 3.82824300 | 0.04412600 | -4.94079700 |
| C | -4.19879400 | 0.81720200 | 1.13325000 |
| H | -3.92054300 | 1.86763800 | 1.11358300 |
| H | -5.12346500 | 0.73232500 | 1.70589800 |
| H | -4.40722500 | 0.47317400 | 0.11798100 |
| C | -1.40764500 | -2.68593100 | -0.45333100 |
| C | -1.71701400 | 2.52276300 | -0.55671600 |
| C | -1.18183600 | 0.23394900 | 3.25568700 |
| C | -0.81667800 | -1.05526000 | 3.99939900 |
| H | 0.26477100 | -1.20104100 | 3.97422500 |
| H | -1.13527500 | -0.97415700 | 5.04086100 |
| H | -1.28160300 | -1.93727700 | 3.55865000 |
| C | -0.43752400 | 1.40647700 | 3.88997400 |
| H | -0.80206200 | 1.56242900 | 4.90822800 |
| H | 0.63423000 | 1.20324700 | 3.92690400 |
| H | -0.59244100 | 2.32396600 | 3.31871000 |
| H | -1.84626000 | -0.17185100 | -4.26602400 |
| C | -2.57032100 | -3.67090600 | -0.84151500 |
| C | -0.03917500 | -3.34132600 | -0.84796800 |
| C | -2.93590400 | 3.38232400 | -1.06588500 |
| C | -0.39312200 | 3.28217600 | -0.91406500 |
| F | 0.94697800 | -2.67734500 | -0.28088300 |
| F | 0.17667000 | -3.37744900 | -2.15509500 |
| F | -0.02910300 | -4.59044500 | -0.38632600 |
| F | -2.28948100 | -4.33288200 | -1.96126800 |
| F | -2.77142900 | -4.54556600 | 0.12748400 |
| F | -3.71276700 | -3.01585700 | -1.04916500 |
| F | -2.63798700 | 4.02573500 | -2.19257600 |
| F | -4.00174900 | 2.62614900 | -1.31570600 |
| F | -3.27264800 | 4.27426600 | -0.15014200 |
| F | -0.10508200 | 3.25258800 | -2.20665600 |
| F | -0.52281400 | 4.55306200 | -0.53837700 |
| F | 0.61438300 | 2.75327800 | -0.24926900 |

| | | | |
|---|---|---|---|
| F | -1.74973100 | 2.58138000 | 0.80379800 |
| F | -1.35463500 | -2.66218300 | 0.90357100 |

68

h-1-1

| | | | |
|---|---|---|---|
| Au | -0.60150400 | -1.02500100 | -0.02242300 |
| N | -2.22325400 | 0.14473000 | 0.01301700 |
| C | -4.40226900 | 0.87932700 | 0.05438500 |
| C | 2.53468600 | 0.35586800 | 1.20284200 |
| N | 2.23921700 | -1.63937500 | -0.16370000 |
| C | 3.35479300 | -2.62770300 | -0.22038700 |
| C | 2.70903700 | 1.72418100 | 1.31412700 |
| H | 2.73612300 | 2.16867100 | 2.30146400 |
| C | -5.41706300 | -1.64175200 | 0.61597200 |
| H | -5.83177300 | -2.61848400 | 0.83554300 |
| C | 1.02831700 | -2.13275000 | -0.11216900 |
| C | -2.22992300 | 1.49947800 | -0.24588400 |
| C | 2.60704700 | 0.55260100 | -1.19278200 |
| C | 2.82420600 | 2.54048700 | 0.18444200 |
| C | -3.53743700 | -0.23180600 | 0.19276400 |
| C | 2.48529900 | -0.22798100 | -0.05670900 |
| C | -3.78346800 | 3.35715800 | -0.49362200 |
| H | -4.79411500 | 3.75051900 | -0.48864200 |
| C | -1.14516600 | 2.34182500 | -0.50520600 |
| H | -0.13095900 | 1.94973000 | -0.50404900 |
| C | -3.55125100 | 2.00645400 | -0.23600400 |
| C | -4.04801500 | -1.50071900 | 0.47509300 |
| H | -3.37828700 | -2.34844700 | 0.58065400 |
| C | 2.57846900 | -3.92984500 | -0.46388500 |
| H | 2.98818100 | -4.75243800 | 0.12524500 |
| H | 2.65455600 | -4.20469600 | -1.51912500 |
| C | -5.77946200 | 0.71465600 | 0.20090800 |
| H | -6.44766700 | 1.56242400 | 0.09605900 |
| C | -1.40155000 | 3.67701200 | -0.76744800 |
| H | -0.57546400 | 4.34520500 | -0.98767500 |

| | | | |
|---|---|---|---|
| C | 2.77424500 | 1.92805000 | -1.06660500 |
| H | 2.84026500 | 2.52316700 | -1.96711000 |
| C | -6.28175500 | -0.54471400 | 0.48119400 |
| H | -7.34858600 | -0.68877500 | 0.59807200 |
| C | 4.11278800 | -2.61434200 | 1.10170400 |
| H | 4.56751700 | -1.63719000 | 1.27606400 |
| H | 4.90861500 | -3.35995300 | 1.06941100 |
| H | 3.45341600 | -2.84766100 | 1.93911500 |
| C | -2.70841300 | 4.18767800 | -0.76224300 |
| H | -2.87292000 | 5.23736300 | -0.97112600 |
| C | 4.29941700 | -2.29079500 | -1.36339800 |
| H | 3.75735300 | -2.23049100 | -2.30901000 |
| H | 5.05299300 | -3.07559400 | -1.44722500 |
| H | 4.81005200 | -1.34221300 | -1.18727100 |
| C | 2.99185200 | 4.04427800 | 0.36331100 |
| C | 1.10815000 | -3.63795100 | -0.11290800 |
| C | 0.72865200 | -4.10862800 | 1.29544100 |
| H | -0.29181500 | -3.80140900 | 1.53217700 |
| H | 0.78809600 | -5.19833600 | 1.34154600 |
| H | 1.39218400 | -3.69320400 | 2.05635000 |
| C | 0.13357200 | -4.23472500 | -1.12222900 |
| H | 0.23353700 | -5.32265400 | -1.13454300 |
| H | -0.89391000 | -3.97975400 | -0.85474700 |
| H | 0.32629200 | -3.85671900 | -2.12805500 |
| C | 4.35976300 | 4.31840600 | 0.99715700 |
| H | 4.48828900 | 5.39264100 | 1.15275100 |
| H | 4.45092200 | 3.82124200 | 1.96542000 |
| H | 5.16711100 | 3.96332300 | 0.35261500 |
| C | 1.89543100 | 4.58339500 | 1.28928600 |
| H | 1.98321300 | 4.18065000 | 2.29950300 |
| H | 1.97945500 | 5.67067300 | 1.35905100 |
| H | 0.90148000 | 4.33434600 | 0.91178200 |
| C | 2.90833300 | 4.79313300 | -0.96342600 |
| H | 2.99888100 | 5.86496300 | -0.77636700 |

| | | | |
|---|---|---|---|
| H | 3.71330300 | 4.50567600 | -1.64381000 |
| H | 1.95122000 | 4.61634100 | -1.46145600 |
| H | 2.40408500 | -0.26464600 | 2.08179400 |
| H | 2.53573000 | 0.09165100 | -2.17049400 |

68

i-1-1

| | | | |
|---|---|---|---|
| Au | 2.20805600 | 0.49235300 | 0.04756400 |
| N | 2.68298000 | -1.44509000 | 0.07607800 |
| C | 3.92000000 | -3.38291700 | 0.00503900 |
| C | -1.39342700 | 1.99188500 | 1.06711700 |
| N | 0.55632800 | 2.91114100 | -0.09660000 |
| C | 0.45637600 | 4.39528200 | -0.24371400 |
| C | -2.51643900 | 1.18057300 | 1.06763000 |
| H | -3.09487100 | 1.11194500 | 1.97555900 |
| C | 6.34375700 | -2.04398300 | 0.18885800 |
| H | 7.29936300 | -1.53826200 | 0.26057000 |
| C | 1.76624100 | 2.40974700 | -0.03966200 |
| C | 1.81252600 | -2.51222300 | -0.02775300 |
| C | -0.93516100 | 1.33531700 | -1.19435800 |
| C | -2.87990300 | 0.44458000 | -0.06390000 |
| C | 3.95880100 | -1.97244200 | 0.09504200 |
| C | -0.61271400 | 2.08320800 | -0.07448600 |
| C | 1.83405700 | -4.94077300 | -0.18826200 |
| H | 2.37491800 | -5.87994500 | -0.22688800 |
| C | 0.41656500 | -2.49842600 | -0.08333300 |
| H | -0.12825600 | -1.55886600 | -0.03180100 |
| C | 2.52358400 | -3.73416100 | -0.07706600 |
| C | 5.17804800 | -1.29820500 | 0.18750000 |
| H | 5.20141100 | -0.21494400 | 0.25998800 |
| C | 1.92844400 | 4.76034900 | -0.48593500 |
| H | 2.20884300 | 5.66373500 | 0.05853000 |
| H | 2.08234700 | 4.95083700 | -1.55122900 |
| C | 5.10637900 | -4.11561100 | 0.00832900 |
| H | 5.08302500 | -5.19767200 | -0.05986600 |

| | | | |
|---|---|---|---|
| C | -0.24775800 | -3.70808300 | -0.19499700 |
| H | -1.33014000 | -3.71876500 | -0.23747000 |
| C | -2.05241800 | 0.51273100 | -1.18590900 |
| H | -2.24013000 | -0.07734700 | -2.06729300 |
| C | 6.31374600 | -3.44364300 | 0.10009500 |
| H | 7.24351600 | -3.99857000 | 0.10420800 |
| C | -0.11469200 | 5.02848300 | 1.01926700 |
| H | -1.15599100 | 4.73860800 | 1.16819500 |
| H | -0.08295700 | 6.11408700 | 0.91590400 |
| H | 0.46214300 | 4.74980800 | 1.90239500 |
| C | 0.45095700 | -4.92314800 | -0.24906200 |
| H | -0.09969000 | -5.85143900 | -0.33575300 |
| C | -0.42865000 | 4.72693900 | -1.43594900 |
| H | -0.06753100 | 4.22662700 | -2.33671100 |
| H | -0.41102300 | 5.80425700 | -1.60758300 |
| H | -1.46282500 | 4.42580400 | -1.25692800 |
| C | -4.10863100 | -0.47285400 | 0.01566000 |
| C | 2.76298600 | 3.54137600 | -0.05704100 |
| C | 3.31660900 | 3.67340600 | 1.36627900 |
| H | 3.81820300 | 2.75023300 | 1.66249600 |
| H | 4.03905700 | 4.49188400 | 1.39829000 |
| H | 2.52744400 | 3.87894300 | 2.09195900 |
| C | 3.90958300 | 3.24992200 | -1.01885100 |
| H | 4.58970800 | 4.10431400 | -1.05140100 |
| H | 4.46835300 | 2.37036500 | -0.69196400 |
| H | 3.54043100 | 3.06097100 | -2.02867000 |
| C | -5.41972300 | 0.33410900 | 0.26512800 |
| C | -3.90312700 | -1.47600900 | 1.19942100 |
| C | -4.31689000 | -1.30933300 | -1.28005500 |
| H | -1.10769500 | 2.52358600 | 1.96488300 |
| H | -0.29065000 | 1.37387300 | -2.06379700 |
| F | -2.64028800 | -1.87301600 | 1.25631900 |
| F | -4.66540400 | -2.55490800 | 1.09269700 |
| F | -4.20144000 | -0.88943000 | 2.36141100 |

| | | | |
|---|---|---|---|
| F | -6.39895900 | -0.45964900 | 0.68482400 |
| F | -5.26384600 | 1.28642500 | 1.17927500 |
| F | -5.81579900 | 0.92819500 | -0.85623400 |
| F | -5.46716200 | -1.96893900 | -1.26668400 |
| F | -4.32883000 | -0.55065000 | -2.37416400 |
| F | -3.32864200 | -2.19346200 | -1.40408200 |

65

j-1-1

| | | | |
|---|---|---|---|
| Au | -0.22353800 | -0.84962700 | -0.03635500 |
| N | -2.14127100 | -0.28767100 | -0.02433600 |
| C | -4.43895300 | -0.20501800 | 0.00789800 |
| C | 2.06430900 | 1.66947800 | 1.22221300 |
| N | 2.62810700 | -0.34838700 | -0.05582400 |
| C | 4.04174200 | -0.83697400 | -0.04282300 |
| C | 1.75774800 | 3.02693400 | 1.23036000 |
| H | 1.55348700 | 3.50549200 | 2.18301800 |
| C | -4.70333500 | -2.96830000 | -0.00372500 |
| H | -4.82675500 | -4.04483700 | -0.00844400 |
| C | 1.70254400 | -1.27324600 | -0.04874800 |
| C | -2.53075600 | 1.03601500 | -0.01288500 |
| C | 2.12772500 | 1.75192500 | -1.21628700 |
| C | 1.65151900 | 3.76916500 | 0.05991400 |
| C | -3.29597600 | -1.04033700 | -0.01242000 |
| C | 2.29824500 | 1.05580800 | -0.01461800 |
| C | -4.53404200 | 2.41080300 | 0.02292000 |
| H | -5.61401100 | 2.50877400 | 0.03909500 |
| C | -1.71991600 | 2.17289700 | -0.02092900 |
| H | -0.63872600 | 2.07391700 | -0.04026800 |
| C | -3.93956300 | 1.14860000 | 0.00795600 |
| C | -3.42878300 | -2.43018300 | -0.01830000 |
| H | -2.54725100 | -3.06292000 | -0.03452100 |
| C | 3.83607100 | -2.33960000 | -0.28349600 |
| H | 4.49976600 | -2.93690300 | 0.34458000 |
| H | 4.06355800 | -2.57418300 | -1.32668300 |

| | | | |
|---|---|---|---|
| C | -5.71420900 | -0.76935900 | 0.02250100 |
| H | -6.59431800 | -0.13582800 | 0.03812900 |
| C | -2.33037800 | 3.41447400 | -0.00543100 |
| H | -1.71551100 | 4.30876000 | -0.01220000 |
| C | 1.81800000 | 3.10716700 | -1.15061600 |
| H | 1.66345800 | 3.64879200 | -2.07861700 |
| C | -5.84178300 | -2.14813000 | 0.01667900 |
| H | -6.82530800 | -2.60104400 | 0.02770200 |
| C | 4.68863400 | -0.52770200 | 1.30140300 |
| H | 4.71393100 | 0.55035000 | 1.47487500 |
| H | 5.71558600 | -0.89602900 | 1.29342300 |
| H | 4.15693600 | -1.00515500 | 2.12443000 |
| C | -3.72858300 | 3.53808800 | 0.01667100 |
| H | -4.17811200 | 4.52325900 | 0.02809400 |
| C | 4.85283900 | -0.18093600 | -1.14928700 |
| H | 4.44396400 | -0.41934500 | -2.13064300 |
| H | 5.87645700 | -0.55649200 | -1.10464100 |
| H | 4.88053200 | 0.90419900 | -1.02655100 |
| C | 2.04964700 | 0.91796200 | 2.52520700 |
| H | 2.98933400 | 1.03230100 | 3.06958900 |
| H | 1.25281700 | 1.30948600 | 3.15712800 |
| H | 1.86430900 | -0.14681600 | 2.38348800 |
| C | 2.18930700 | 1.08808800 | -2.56412400 |
| H | 1.39368200 | 1.48072100 | -3.19713000 |
| H | 3.13852700 | 1.28898000 | -3.06549900 |
| H | 2.05851700 | 0.00802400 | -2.49817100 |
| C | 1.32128700 | 5.23330200 | 0.10195300 |
| H | 2.23099800 | 5.83507500 | 0.16276700 |
| H | 0.78142500 | 5.53925500 | -0.79450800 |
| H | 0.70865500 | 5.47141300 | 0.97192300 |
| C | 2.35149200 | -2.63145600 | -0.00318900 |
| C | 2.11366800 | -3.19830900 | 1.40047600 |
| H | 1.04329100 | -3.28992100 | 1.59434600 |
| H | 2.57154500 | -4.18718800 | 1.47545600 |

| | | | |
|---|---|---|---|
| H | 2.54574900 | -2.55923700 | 2.17378600 |
| C | 1.72910700 | -3.56056300 | -1.03921300 |
| H | 2.23713600 | -4.52768100 | -1.02477500 |
| H | 0.67029000 | -3.71592700 | -0.82245100 |
| H | 1.81204700 | -3.13991800 | -2.04393000 |

71

j-2-1

| | | | |
|---|---|---|---|
| Au | -0.23784700 | -0.69846200 | 0.02063700 |
| N | -2.21508900 | -0.40572300 | 0.01253600 |
| C | -4.50222700 | -0.63795500 | -0.02702100 |
| C | 1.73949900 | 2.23014600 | 1.15947900 |
| N | 2.51701600 | 0.19309100 | 0.02988500 |
| C | 3.98526200 | -0.08923400 | 0.01467300 |
| C | 1.24522000 | 3.52871500 | 1.07054900 |
| H | 1.02637700 | 4.06296000 | 1.98986400 |
| C | -4.38585200 | -3.41142500 | -0.04557700 |
| H | -4.36078300 | -4.49472800 | -0.05295400 |
| C | 1.73150400 | -0.85222600 | 0.02964500 |
| C | -2.78188200 | 0.85213500 | 0.01293100 |
| C | 1.66729600 | 2.08931800 | -1.27537900 |
| C | 0.97605800 | 4.13517600 | -0.14984100 |
| C | -3.25555000 | -1.30916400 | -0.01129800 |
| C | 1.99428400 | 1.53828100 | -0.02926200 |
| C | -4.95444800 | 1.94025500 | -0.01555100 |
| H | -6.03764500 | 1.88976200 | -0.03418900 |
| C | -2.13415900 | 2.08915800 | 0.03458000 |
| H | -1.04958500 | 2.13878500 | 0.05558100 |
| C | -4.19286900 | 0.77110500 | -0.01147800 |
| C | -3.19694100 | -2.70420100 | -0.02058100 |
| H | -2.23713500 | -3.21051200 | -0.00794900 |
| C | 4.00894300 | -1.62047100 | 0.15247600 |
| H | 4.39937600 | -1.90093100 | 1.13270000 |
| H | 4.66964400 | -2.06087200 | -0.59703300 |
| C | -5.68817100 | -1.37141300 | -0.05213800 |

| | | | |
|---|---|---|---|
| H | -6.64668200 | -0.86415200 | -0.06429000 |
| C | -2.90881900 | 3.23556600 | 0.02981700 |
| H | -2.42214900 | 4.20544700 | 0.04842700 |
| C | 1.17366300 | 3.38954600 | -1.30652000 |
| H | 0.89695200 | 3.81474000 | -2.26619100 |
| C | -5.62585500 | -2.75463200 | -0.06143200 |
| H | -6.53811100 | -3.33771400 | -0.08081700 |
| C | 4.67557700 | 0.60462700 | 1.17834500 |
| H | 4.54625100 | 1.68803700 | 1.12456500 |
| H | 5.74346800 | 0.38411900 | 1.13654800 |
| H | 4.28767900 | 0.24525600 | 2.13224000 |
| C | -4.31081500 | 3.16695000 | 0.00477300 |
| H | -4.89084600 | 4.08145400 | 0.00218100 |
| C | 4.59228500 | 0.38812300 | -1.29791100 |
| H | 4.15160900 | -0.13321200 | -2.14861800 |
| H | 5.66421400 | 0.18491700 | -1.28686600 |
| H | 4.44810200 | 1.46334300 | -1.42544400 |
| C | 1.89962300 | 1.61093700 | 2.52005900 |
| H | 2.81501700 | 1.95020700 | 3.00982500 |
| H | 1.06071500 | 1.90382000 | 3.15131300 |
| H | 1.92024700 | 0.52277000 | 2.47614300 |
| C | 1.75327600 | 1.32156200 | -2.56598500 |
| H | 0.90474700 | 1.58372800 | -3.19749700 |
| H | 2.66377900 | 1.56326400 | -3.11842700 |
| H | 1.72532400 | 0.24353700 | -2.40778800 |
| C | 0.44415300 | 5.53760500 | -0.21837300 |
| H | 1.25653300 | 6.25507500 | -0.35492400 |
| H | -0.24356300 | 5.65432900 | -1.05655400 |
| H | -0.08183700 | 5.80280800 | 0.69897000 |
| C | 2.55814500 | -2.10933800 | -0.02043700 |
| C | 2.11724400 | -3.08498200 | 1.07793900 |
| C | 2.32287000 | -2.72129300 | -1.41723200 |
| H | 1.24295000 | -2.80461000 | -1.57510900 |
| H | 2.82863000 | -3.91580500 | 1.09610900 |

| | | | |
|---|---|---|---|
| C | 2.01546100 | -2.44938700 | 2.45802900 |
| H | 2.92805100 | -1.90501700 | 2.72139200 |
| H | 1.18085100 | -1.74597900 | 2.49699100 |
| H | 1.85074500 | -3.20323100 | 3.22754200 |
| H | 1.14371100 | -3.50004300 | 0.79340000 |
| C | 2.98379200 | -4.07847800 | -1.61288600 |
| H | 2.51200500 | -4.84164000 | -0.99352800 |
| H | 2.89816200 | -4.39804800 | -2.65132900 |
| H | 4.04661800 | -4.05199000 | -1.35990900 |
| H | 2.69361300 | -2.01298900 | -2.16798900 |

80

j-3-1

| | | | |
|---|---|---|---|
| Au | -0.26612500 | -0.35991200 | -0.15960800 |
| N | -2.12362100 | -1.08913700 | -0.10762100 |
| C | -3.93438500 | -2.49819900 | 0.04011300 |
| C | -0.03917300 | 2.84188900 | 1.31796600 |
| N | 1.66359800 | 1.75045800 | -0.07316200 |
| C | 3.12645200 | -1.23411300 | -1.29017600 |
| H | 2.90439600 | -0.75953200 | -2.25395200 |
| C | 2.53328700 | -3.11936100 | 0.24375200 |
| H | 1.86921100 | -3.97659500 | 0.38652200 |
| C | 3.05187900 | 2.28868600 | -0.03228000 |
| C | -1.12505900 | 3.70555100 | 1.40923700 |
| H | -1.57891400 | 3.86525800 | 2.38239100 |
| C | 3.20613300 | -0.88607300 | 1.17768700 |
| H | 3.03298700 | -0.17635500 | 1.99502600 |
| C | -2.35688400 | -4.78194100 | 0.13259000 |
| H | -1.75851200 | -5.68450700 | 0.17196600 |
| C | 1.54797200 | 0.44703100 | -0.16126500 |
| C | -3.27079000 | -0.32477200 | -0.10748700 |
| C | 2.23944400 | -2.46462000 | -1.10431700 |
| H | 2.45470600 | -3.17233300 | -1.91239400 |
| H | 1.18103600 | -2.20211100 | -1.18157100 |
| C | -0.05401700 | 3.16289900 | -1.10148700 |

| | | | |
|---|---|---|---|
| C | -1.67467600 | 4.32644900 | 0.29236300 |
| C | 2.29944000 | -2.10859700 | 1.36666000 |
| H | 2.53952900 | -2.56869600 | 2.33124000 |
| H | 1.24495700 | -1.82027600 | 1.40042700 |
| C | 3.98924000 | -3.58034400 | 0.26500100 |
| H | 4.21647800 | -4.07198100 | 1.21702200 |
| H | 4.16253500 | -4.30812600 | -0.53484000 |
| C | -2.52257500 | -2.40488000 | -0.01405300 |
| C | 0.51783000 | 2.61786800 | 0.05169800 |
| C | 2.93142800 | -0.17658700 | -0.18033500 |
| C | -5.68741200 | -0.55445300 | -0.00432200 |
| H | -6.57756800 | -1.17038900 | 0.06230700 |
| C | -3.37891700 | 1.06514200 | -0.17851900 |
| H | -2.48542600 | 1.67792300 | -0.24598400 |
| C | 4.58892500 | -1.69521300 | -1.26228100 |
| H | 4.75083400 | -2.40716900 | -2.07784700 |
| H | 5.27019400 | -0.85497000 | -1.42942100 |
| C | -4.42160500 | -1.14125200 | -0.01999900 |
| C | -1.72869400 | -3.55275800 | 0.03555600 |
| H | -0.64549300 | -3.47160600 | 0.00100500 |
| C | 3.84012800 | 1.04575200 | -0.43768700 |
| H | 4.79582800 | 1.00430000 | 0.08203600 |
| H | 4.05394400 | 1.09531500 | -1.51077000 |
| C | -4.54566700 | -3.74780100 | 0.13772100 |
| H | -5.62640000 | -3.82815900 | 0.17933300 |
| C | -4.64377900 | 1.62572400 | -0.16009100 |
| H | -4.74822200 | 2.70427800 | -0.21844500 |
| C | -1.13693100 | 4.02604100 | -0.95226000 |
| H | -1.59701700 | 4.43905900 | -1.84466700 |
| C | -3.75540500 | -4.88431200 | 0.18225800 |
| H | -4.21613300 | -5.86129900 | 0.25816400 |
| C | 4.65575400 | -1.38155700 | 1.22024800 |
| H | 5.37083000 | -0.55685300 | 1.17177900 |
| H | 4.81997100 | -1.88409500 | 2.17925500 |

| | | | |
|---|---|---|---|
| C | 3.38453200 | 2.78233200 | 1.37100900 |
| H | 2.72151000 | 3.60251500 | 1.65465800 |
| H | 4.41008800 | 3.15481300 | 1.38121400 |
| H | 3.29792300 | 1.98655100 | 2.11062900 |
| C | 4.89979400 | -2.36812300 | 0.07719200 |
| H | 5.94617400 | -2.68566600 | 0.08930100 |
| C | -5.79397400 | 0.82513300 | -0.07409200 |
| H | -6.77075500 | 1.29262700 | -0.06309500 |
| C | 3.23473600 | 3.43445000 | -1.01571100 |
| H | 3.04634400 | 3.11114500 | -2.03866700 |
| H | 4.26609700 | 3.78586800 | -0.95356700 |
| H | 2.57389000 | 4.27043700 | -0.77643900 |
| C | -2.84990400 | 5.25079000 | 0.43029900 |
| H | -2.53210400 | 6.23709500 | 0.77568200 |
| H | -3.36214500 | 5.37986800 | -0.52312000 |
| H | -3.56601700 | 4.86317600 | 1.15622400 |
| C | 0.39165000 | 2.79620800 | -2.49010600 |
| H | -0.48393600 | 2.59958000 | -3.10933600 |
| H | 0.94997700 | 3.60973500 | -2.95835600 |
| H | 1.01324100 | 1.90121300 | -2.50185600 |
| C | 0.42925800 | 2.13482600 | 2.56063200 |
| H | 1.07019400 | 2.77367600 | 3.17191700 |
| H | -0.43514000 | 1.85920600 | 3.16446200 |
| H | 0.97672300 | 1.21996700 | 2.33317400 |

72

j-4-1

| | | | |
|---|---|---|---|
| Au | -0.20218000 | -0.58480100 | -0.07523400 |
| N | -2.19882000 | -0.67152300 | -0.04215800 |
| C | -4.39743100 | -1.34233400 | 0.01772300 |
| C | 1.15371300 | 2.50487600 | 1.23070500 |
| N | 2.33666900 | 0.80476700 | -0.08554200 |
| C | 3.83033600 | 0.79746800 | -0.07518000 |
| C | 0.42560100 | 3.69044400 | 1.26492900 |
| H | 0.07943200 | 4.05737800 | 2.22611000 |

| | | | |
|---|---|---|---|
| C | -3.74729600 | -4.04064600 | -0.04151300 |
| H | -3.51345600 | -5.09848800 | -0.06448800 |
| C | 1.75958000 | -0.36966700 | -0.09911100 |
| C | -2.99743800 | 0.45262400 | -0.00117400 |
| C | 1.18274900 | 2.65453800 | -1.20530300 |
| C | 0.08344500 | 4.38298800 | 0.10940200 |
| C | -3.04515000 | -1.75913200 | -0.03115400 |
| C | 1.57122800 | 2.02571300 | -0.01718000 |
| C | -5.33818700 | 1.09954300 | 0.08391200 |
| H | -6.39087500 | 0.84040000 | 0.11412100 |
| C | -2.60078200 | 1.79154100 | 0.00368800 |
| H | -1.54654800 | 2.04966900 | -0.02996200 |
| C | -4.36577300 | 0.09997700 | 0.03771500 |
| C | -2.71806500 | -3.11623900 | -0.06097400 |
| H | -1.67874000 | -3.42659600 | -0.09877100 |
| C | 4.12461400 | -0.68841600 | -0.33121600 |
| H | 4.93938800 | -1.03772600 | 0.30532700 |
| H | 4.43887400 | -0.82678500 | -1.36994500 |
| C | -5.41876000 | -2.29189000 | 0.03664100 |
| H | -6.45687000 | -1.98020600 | 0.07403400 |
| C | -3.58165300 | 2.76628000 | 0.05050100 |
| H | -3.29145200 | 3.81204700 | 0.05419300 |
| C | 0.45229600 | 3.83555000 | -1.11362500 |
| H | 0.12948000 | 4.31766400 | -2.03113200 |
| C | -5.09030900 | -3.63675300 | 0.00700700 |
| H | -5.87240000 | -4.38556100 | 0.02110900 |
| C | 4.34351500 | 1.27804800 | 1.27634700 |
| H | 4.01658100 | 2.30210300 | 1.46971100 |
| H | 5.43446400 | 1.26369700 | 1.26698000 |
| H | 3.99619400 | 0.63670800 | 2.08682700 |
| C | -4.94349200 | 2.42759000 | 0.09074100 |
| H | -5.68873100 | 3.21252000 | 0.12647000 |
| C | 4.38640500 | 1.69350100 | -1.17069100 |
| H | 4.07524100 | 1.34801000 | -2.15610400 |

| | | | |
|---|---|---|---|
| H | 5.47647400 | 1.66705100 | -1.12713900 |
| H | 4.06310400 | 2.72816500 | -1.03496500 |
| C | 1.38369100 | 1.76172400 | 2.51821600 |
| H | 2.23128900 | 2.16784300 | 3.07424900 |
| H | 0.50009100 | 1.85468900 | 3.14930600 |
| H | 1.56074300 | 0.69877000 | 2.35401600 |
| C | 1.45310300 | 2.07374700 | -2.56573700 |
| H | 0.57467500 | 2.20722500 | -3.19690500 |
| H | 2.29010100 | 2.57576800 | -3.05578600 |
| H | 1.67177600 | 1.00680100 | -2.52185400 |
| C | -0.70153500 | 5.66105700 | 0.17943000 |
| H | -0.03448600 | 6.52322600 | 0.25111100 |
| H | -1.31709000 | 5.79222200 | -0.71087000 |
| H | -1.35257200 | 5.67304900 | 1.05399100 |
| C | 2.80764400 | -1.44718600 | -0.08451400 |
| C | 2.74714100 | -2.12728600 | 1.29664200 |
| C | 2.51542700 | -2.49381900 | -1.16373500 |
| C | 3.69621300 | -3.31672800 | 1.38638400 |
| H | 1.71675500 | -2.46658200 | 1.45581600 |
| H | 2.96511100 | -1.39493500 | 2.08137700 |
| C | 3.49963400 | -3.65315300 | -1.08588800 |
| H | 1.49482700 | -2.86525500 | -1.01157600 |
| H | 2.53784600 | -2.01521400 | -2.14830000 |
| C | 3.41263800 | -4.32447900 | 0.27929200 |
| H | 3.60072800 | -3.78418200 | 2.36843700 |
| H | 4.73442700 | -2.97500400 | 1.30304100 |
| H | 3.29469300 | -4.36982800 | -1.88324300 |
| H | 4.52092100 | -3.28443500 | -1.24605600 |
| H | 4.10610600 | -5.16544500 | 0.34228700 |
| H | 2.40254700 | -4.72907800 | 0.41233300 |

70

j-5-1

| | | | |
|---|---|---|---|
| Au | -0.20917100 | -0.61443800 | -0.06090400 |
| N | -2.20664500 | -0.66242500 | -0.03385000 |

| | | | |
|---|---|---|---|
| C | -4.41916700 | -1.28687000 | 0.01136800 |
| C | 1.22208200 | 2.45621800 | 1.22699500 |
| N | 2.35927200 | 0.72500300 | -0.08589600 |
| C | 3.85419800 | 0.68334700 | -0.08584300 |
| C | 0.51737800 | 3.65572900 | 1.26022400 |
| H | 0.18536500 | 4.03441000 | 2.22183000 |
| C | -3.82567300 | -3.99830600 | -0.03340900 |
| H | -3.61392700 | -5.06087200 | -0.05058800 |
| C | 1.75499900 | -0.43607400 | -0.08383600 |
| C | -2.98190400 | 0.47855300 | -0.00304300 |
| C | 1.23716600 | 2.59300300 | -1.21004700 |
| C | 0.18083800 | 4.34906100 | 0.10335200 |
| C | -3.07580400 | -1.73225900 | -0.02557900 |
| C | 1.62109800 | 1.96274900 | -0.02148000 |
| C | -5.30934800 | 1.17408300 | 0.06113800 |
| H | -6.36734400 | 0.93677700 | 0.08404900 |
| C | -2.55763700 | 1.80896300 | 0.00048700 |
| H | -1.49805500 | 2.04544700 | -0.02476500 |
| C | -4.35747800 | 0.15455400 | 0.02636900 |
| C | -2.77699200 | -3.09594200 | -0.04812700 |
| H | -1.74435000 | -3.42846100 | -0.07646700 |
| C | 4.10741100 | -0.80969500 | -0.34194600 |
| H | 4.92867200 | -1.19377900 | 0.26663200 |
| H | 4.37690000 | -0.95277000 | -1.39288000 |
| C | -5.46051400 | -2.21451300 | 0.02566000 |
| H | -6.49205600 | -1.88087000 | 0.05397600 |
| C | -3.51851400 | 2.80398000 | 0.03555000 |
| H | -3.20672400 | 3.84351400 | 0.03787100 |
| C | 0.53081100 | 3.78876700 | -1.11930800 |
| H | 0.21118000 | 4.27264800 | -2.03697900 |
| C | -5.16019900 | -3.56602900 | 0.00325300 |
| H | -5.95795400 | -4.29818500 | 0.01395100 |
| C | 4.39203800 | 1.16591300 | 1.25533400 |
| H | 4.09409100 | 2.20109100 | 1.43651700 |

| | | | |
|---|---|---|---|
| H | 5.48198800 | 1.12270900 | 1.23595900 |
| H | 4.03657000 | 0.54804400 | 2.07961500 |
| C | -4.88730800 | 2.49371200 | 0.06599100 |
| H | -5.61636100 | 3.29403900 | 0.09280500 |
| C | 4.41985300 | 1.56052800 | -1.19206900 |
| H | 4.09072800 | 1.21774600 | -2.17252600 |
| H | 5.50934200 | 1.50832400 | -1.15910900 |
| H | 4.12269200 | 2.60327500 | -1.05840700 |
| C | 1.44894900 | 1.71580600 | 2.51675000 |
| H | 2.31193500 | 2.10573200 | 3.06054800 |
| H | 0.57504800 | 1.83318600 | 3.15713500 |
| H | 1.59999900 | 0.64778100 | 2.35799100 |
| C | 1.48591100 | 1.99959300 | -2.56904300 |
| H | 0.60642100 | 2.14869900 | -3.19520600 |
| H | 2.33040200 | 2.48065500 | -3.06716400 |
| H | 1.68163900 | 0.92842500 | -2.52050100 |
| C | -0.57891300 | 5.64232300 | 0.17263000 |
| H | 0.10477300 | 6.49079400 | 0.25055800 |
| H | -1.18695100 | 5.78805700 | -0.72051400 |
| H | -1.23426200 | 5.66492400 | 1.04379100 |
| C | 2.78168800 | -1.53166900 | -0.05070000 |
| C | 2.74357400 | -2.16492000 | 1.34917000 |
| C | 2.46697900 | -2.62423000 | -1.07345900 |
| C | 3.54568800 | -3.43186100 | 1.39789200 |
| H | 1.69897500 | -2.35857200 | 1.62335400 |
| H | 3.12359400 | -1.45370700 | 2.09068300 |
| C | 3.56758400 | -3.67693300 | -1.07918300 |
| H | 1.50890700 | -3.07907700 | -0.80109000 |
| H | 2.34312000 | -2.18110700 | -2.06613200 |
| C | 3.93130400 | -4.09769700 | 0.31311500 |
| H | 3.81699100 | -3.80366500 | 2.38012800 |
| H | 3.24585600 | -4.54765100 | -1.65551600 |
| H | 4.45954600 | -3.29800600 | -1.59368200 |
| H | 4.52880700 | -4.99630500 | 0.42450700 |

84
j-6-1

| | | | |
|---|---|---|---|
| Au | 0.33831100 | -0.39612800 | -0.15938600 |
| N | 2.25175100 | -0.96847000 | -0.19084200 |
| C | 4.20382300 | -2.17834300 | -0.08982200 |
| C | -0.36452000 | 3.06362300 | -1.14554900 |
| N | -1.80111400 | 1.53292700 | 0.12126200 |
| C | -3.25059600 | 1.85464200 | 0.28520300 |
| C | 0.63039400 | 4.03651700 | -1.15533500 |
| H | 0.96657200 | 4.42255600 | -2.11257500 |
| C | 2.87931400 | -4.61841900 | -0.02383200 |
| H | 2.38093400 | -5.58021700 | 0.00315800 |
| C | -1.51629800 | 0.26687700 | -0.04707400 |
| C | 3.31148500 | -0.08571200 | -0.18984300 |
| C | -0.13557200 | 2.92638700 | 1.27891100 |
| C | 1.23923300 | 4.48518500 | 0.01075600 |
| C | 2.78951200 | -2.23526200 | -0.12605000 |
| C | -0.77448700 | 2.54960600 | 0.09201700 |
| C | 5.74016700 | -0.05881400 | -0.11929100 |
| H | 6.69141200 | -0.57782000 | -0.07499600 |
| C | 3.27083500 | 1.30953800 | -0.23554000 |
| H | 2.31752800 | 1.82826200 | -0.28133300 |
| C | 4.54357700 | -0.77642200 | -0.13120300 |
| C | 2.12259100 | -3.46185300 | -0.09187100 |
| H | 1.03732600 | -3.49555900 | -0.12097600 |
| C | -3.87266900 | 0.44785100 | 0.33876700 |
| H | -4.77472600 | 0.39969400 | -0.27385500 |
| H | -4.16190800 | 0.22185300 | 1.36575800 |
| C | 4.94577300 | -3.35725000 | -0.02247200 |
| H | 6.02939800 | -3.32280600 | 0.00512500 |
| C | 4.46942500 | 2.00058900 | -0.22016600 |
| H | 4.45792900 | 3.08516000 | -0.25582200 |
| C | 0.85484600 | 3.90199900 | 1.21140000 |
| H | 1.36985300 | 4.17929300 | 2.12582700 |

| | | | |
|---|---|---|---|
| C | 4.28133100 | -4.57194500 | 0.01012100 |
| H | 4.84427300 | -5.49547800 | 0.06256500 |
| C | -3.74808000 | 2.68403900 | -0.89070600 |
| H | -3.19406600 | 3.62292500 | -0.96168400 |
| H | -4.80218600 | 2.92018500 | -0.73735400 |
| H | -3.64987600 | 2.14221300 | -1.83156900 |
| C | 5.69900600 | 1.32529500 | -0.16326700 |
| H | 6.62077000 | 1.89364100 | -0.15409500 |
| C | -3.47704200 | 2.62079300 | 1.57926600 |
| H | -3.16455300 | 2.02665000 | 2.43920000 |
| H | -4.54187100 | 2.83745100 | 1.68009800 |
| H | -2.93169100 | 3.56750400 | 1.57878200 |
| C | -0.89224900 | 2.56194700 | -2.46150500 |
| H | -1.71648900 | 3.17644300 | -2.82900900 |
| H | -0.09785400 | 2.59876300 | -3.20627000 |
| H | -1.23819400 | 1.53014900 | -2.39741300 |
| C | -0.40835100 | 2.27263800 | 2.60446000 |
| H | 0.52713800 | 2.16472800 | 3.15285200 |
| H | -1.08464300 | 2.87181600 | 3.21779300 |
| H | -0.84083300 | 1.27977300 | 2.48805200 |
| C | 2.31304500 | 5.53370100 | -0.03121400 |
| H | 1.88291900 | 6.53435300 | 0.05306000 |
| H | 3.01720000 | 5.40559300 | 0.79125500 |
| H | 2.86672700 | 5.49012600 | -0.96951500 |
| C | -2.78851500 | -0.53359400 | -0.13745900 |
| C | -2.94780300 | -0.89882700 | -1.62724900 |
| C | -2.66656900 | -1.83222000 | 0.67820000 |
| C | -4.12935200 | -1.82576600 | -1.89240800 |
| H | -2.02668800 | -1.39757800 | -1.95815800 |
| H | -3.04646600 | 0.01508900 | -2.22504200 |
| C | -3.86572000 | -2.74002000 | 0.43170600 |
| H | -1.78939400 | -2.33993800 | 0.24623400 |
| C | -3.99189900 | -3.08339600 | -1.04518000 |
| H | -5.05411400 | -1.31556200 | -1.59026700 |

| | | | |
|---|---|---|---|
| H | -3.76075600 | -3.65172300 | 1.02682700 |
| H | -4.78405400 | -2.24494000 | 0.77380200 |
| H | -4.84451800 | -3.74533000 | -1.22012100 |
| H | -3.09335700 | -3.62413700 | -1.37055600 |
| C | -4.21571600 | -2.15961300 | -3.37395100 |
| H | -5.06223700 | -2.81710500 | -3.58056500 |
| H | -4.32623900 | -1.25947900 | -3.98212600 |
| H | -3.30531500 | -2.67265900 | -3.69587200 |
| C | -2.34780600 | -1.60005800 | 2.17581300 |
| C | -3.53332200 | -1.75417800 | 3.12888000 |
| H | -3.87302800 | -2.79225100 | 3.15652500 |
| H | -3.22599800 | -1.48649600 | 4.14153400 |
| H | -4.39292400 | -1.13521200 | 2.86718300 |
| C | -1.24132800 | -2.55613900 | 2.61144600 |
| H | -1.56248500 | -3.59016300 | 2.44700700 |
| H | -0.32511400 | -2.39061300 | 2.03870900 |
| H | -1.01073500 | -2.44343400 | 3.67255600 |
| H | -1.96749300 | -0.57316500 | 2.27803700 |

56

k-1-1

| | | | |
|---|---|---|---|
| Au | -0.23949000 | -0.81266100 | -0.08092700 |
| N | -2.15445900 | -0.25105200 | -0.06855900 |
| C | -4.45166600 | -0.20153500 | 0.02027400 |
| C | 2.05977900 | 1.61705100 | 1.21782700 |
| N | 2.63544700 | -0.37343100 | -0.08482600 |
| C | 4.03545300 | -0.89475700 | -0.03065500 |
| C | 1.73216700 | 2.95311900 | 1.33441200 |
| H | 1.49687100 | 3.38976100 | 2.29376800 |
| C | -4.67618500 | -2.96774900 | 0.05880900 |
| H | -4.78395400 | -4.04579100 | 0.07404400 |
| C | 1.67473600 | -1.26933900 | -0.08233000 |
| C | -2.56289800 | 1.06678800 | -0.06812800 |
| C | 2.26616200 | 1.81871800 | -1.14038100 |
| C | 1.68725000 | 3.69838900 | 0.16870400 |

| | | | |
|---|---|---|---|
| C | -3.29823100 | -1.02055000 | -0.01564900 |
| C | 2.34982900 | 1.01954200 | -0.00581100 |
| C | -4.58551300 | 2.41172600 | 0.00016600 |
| H | -5.66605300 | 2.49306900 | 0.04293100 |
| C | -1.77279500 | 2.21671700 | -0.11542700 |
| H | -0.69131200 | 2.13024700 | -0.16768600 |
| C | -3.97217400 | 1.15900500 | -0.01261400 |
| C | -3.41032900 | -2.41181400 | 0.00385300 |
| H | -2.52068700 | -3.03294700 | -0.02456300 |
| C | 3.78863300 | -2.39170200 | -0.26362900 |
| H | 4.42446300 | -3.00218000 | 0.37993500 |
| H | 4.02443000 | -2.64011100 | -1.30139700 |
| C | -5.71785600 | -0.78319900 | 0.07571400 |
| H | -6.60631500 | -0.16199800 | 0.10341900 |
| C | -2.40020400 | 3.44992100 | -0.10015000 |
| H | -1.80172100 | 4.35375500 | -0.13555900 |
| C | 1.94401000 | 3.16089400 | -1.08050000 |
| H | 1.87361900 | 3.75826100 | -1.97756200 |
| C | -5.82531600 | -2.16355100 | 0.09468900 |
| H | -6.80152800 | -2.63005200 | 0.13736400 |
| C | 4.64243600 | -0.58345900 | 1.33165600 |
| H | 4.69285300 | 0.49577000 | 1.49407200 |
| H | 5.65896700 | -0.97754500 | 1.36784800 |
| H | 4.06425900 | -1.02811000 | 2.14086400 |
| C | -3.79859800 | 3.55128800 | -0.04194700 |
| H | -4.26278100 | 4.52946800 | -0.03201700 |
| C | 4.87750000 | -0.26471000 | -1.12838100 |
| H | 4.44052900 | -0.44499300 | -2.11017600 |
| H | 5.87658500 | -0.70237000 | -1.10308000 |
| H | 4.97844400 | 0.81289500 | -0.97778200 |
| C | 2.29161100 | -2.63960500 | -0.00324600 |
| C | 2.01400700 | -3.15553000 | 1.41477000 |
| H | 0.93934600 | -3.26043700 | 1.57317100 |
| H | 2.48681900 | -4.13195200 | 1.54113400 |

| | | | |
|---|---|---|---|
| H | 2.40123200 | -2.47726200 | 2.17735400 |
| C | 1.66415600 | -3.58682700 | -1.01926800 |
| H | 2.14249300 | -4.56681100 | -0.95484800 |
| H | 0.59725700 | -3.70468100 | -0.81902500 |
| H | 1.78097600 | -3.20922200 | -2.03659100 |
| F | 2.48549700 | 1.25670100 | -2.32407700 |
| F | 1.36784400 | 4.98617300 | 0.25096600 |
| F | 2.08801200 | 0.85621700 | 2.30697600 |

56

l-1-1

| | | | |
|---|---|---|---|
| Au | -0.43711500 | -0.96666600 | -0.03528600 |
| N | -2.30474200 | -0.26405200 | -0.02392400 |
| C | -4.59318600 | -0.04112000 | 0.00003100 |
| C | 2.06436700 | 1.35094000 | 1.18839600 |
| N | 2.45706600 | -0.73063800 | -0.06172700 |
| C | 3.82438300 | -1.35062800 | -0.05252200 |
| C | 1.86878900 | 2.72279900 | 1.23256900 |
| H | 1.72584700 | 3.21996300 | 2.18133200 |
| C | -5.02720600 | -2.78245400 | -0.03636600 |
| H | -5.21656900 | -3.84920400 | -0.05092900 |
| C | 1.43950100 | -1.56018100 | -0.04725300 |
| C | -2.61184600 | 1.08075000 | -0.00299800 |
| C | 2.11301200 | 1.41819600 | -1.20396100 |
| C | 1.81979900 | 3.43309500 | 0.04399100 |
| C | -3.50443000 | -0.94482800 | -0.02281400 |
| C | 2.24515500 | 0.68055000 | -0.02463700 |
| C | -4.52768400 | 2.57469400 | 0.03697800 |
| H | -5.59974200 | 2.73767700 | 0.04996100 |
| C | -1.73550800 | 2.16750800 | 0.00179700 |
| H | -0.66250000 | 1.99921400 | -0.01455400 |
| C | -4.01110200 | 1.27920000 | 0.01343600 |
| C | -3.72189400 | -2.32354200 | -0.04118300 |
| H | -2.88134100 | -3.00995500 | -0.05940500 |
| C | 3.46159700 | -2.82291600 | -0.29328200 |

| | | | |
|---|---|---|---|
| H | 4.07508600 | -3.48549800 | 0.31986500 |
| H | 3.64152300 | -3.07256900 | -1.34197400 |
| C | -5.90084400 | -0.52575400 | 0.00465100 |
| H | -6.74027100 | 0.16061200 | 0.02210300 |
| C | -2.26775200 | 3.44472500 | 0.02554200 |
| H | -1.60126500 | 4.30056100 | 0.02956800 |
| C | 1.91712000 | 2.79016600 | -1.17973000 |
| H | 1.81281300 | 3.33994000 | -2.10418900 |
| C | -6.11283000 | -1.89395800 | -0.01351000 |
| H | -7.12240100 | -2.28538500 | -0.01042200 |
| C | 4.51244300 | -1.11664000 | 1.28678700 |
| H | 4.63532100 | -0.04879100 | 1.48149200 |
| H | 5.50439800 | -1.56944200 | 1.24948500 |
| H | 3.95920400 | -1.55939500 | 2.11283700 |
| C | -3.65564200 | 3.65133200 | 0.04347600 |
| H | -4.04442600 | 4.66174700 | 0.06180000 |
| C | 4.69484100 | -0.77762500 | -1.16028800 |
| H | 4.27030000 | -0.97156300 | -2.14312000 |
| H | 5.67748700 | -1.24883600 | -1.10715700 |
| H | 4.83244600 | 0.29975200 | -1.03818800 |
| C | 1.96114000 | -2.96935700 | 0.01594900 |
| C | 1.69181700 | -3.46649200 | 1.44194100 |
| H | 0.61781600 | -3.49131900 | 1.63441800 |
| H | 2.09441500 | -4.47551800 | 1.55448000 |
| H | 2.15214400 | -2.81889700 | 2.19068000 |
| C | 1.23988400 | -3.86675300 | -0.98270600 |
| H | 1.65638700 | -4.87583800 | -0.94172700 |
| H | 0.17490300 | -3.91753900 | -0.74716000 |
| H | 1.34623500 | -3.48708300 | -2.00076200 |
| Cl | 1.57455600 | 5.13012400 | 0.08645100 |
| Cl | 1.98143200 | 0.47468800 | 2.65930300 |
| Cl | 2.09427500 | 0.62263400 | -2.72146100 |

65

m-1-1

| | | | |
|---|---|---|---|
| Au | -1.00023600 | -1.08602200 | 0.02324300 |
| N | -2.61186000 | 0.08695500 | 0.04059700 |
| C | -4.74840000 | 0.93605700 | 0.01831700 |
| C | 2.05653500 | 0.59905900 | 1.19419800 |
| N | 1.85383100 | -1.55536700 | -0.00325600 |
| C | 3.04229400 | -2.47370500 | -0.05223100 |
| C | 2.24614700 | 1.97638800 | 1.16117700 |
| H | 2.29584600 | 2.52867800 | 2.09063900 |
| C | -5.92563300 | -1.57702500 | -0.02577500 |
| H | -6.40352200 | -2.54923500 | -0.04329800 |
| C | 0.67158400 | -2.12568400 | -0.01505400 |
| C | -2.53441400 | 1.46469700 | 0.05507400 |
| C | 1.98718200 | 0.56815600 | -1.21112500 |
| C | 2.33000500 | 2.64375000 | -0.04621300 |
| C | -3.95374400 | -0.23451500 | 0.01701500 |
| C | 1.97870800 | -0.12977700 | 0.00208200 |
| C | -3.95914400 | 3.43164700 | 0.05265700 |
| H | -4.94345900 | 3.88650400 | 0.04296500 |
| C | -1.39147900 | 2.26558500 | 0.07756500 |
| H | -0.40864600 | 1.80382100 | 0.08797200 |
| C | -3.82318700 | 2.04355200 | 0.04247200 |
| C | -4.54437300 | -1.49871500 | -0.00521000 |
| H | -3.92749000 | -2.39153600 | -0.00601500 |
| C | 2.34407000 | -3.79038700 | -0.41818100 |
| H | 2.79500900 | -4.63543900 | 0.10484900 |
| H | 2.44617600 | -3.96200400 | -1.49315300 |
| C | -6.13903300 | 0.83378800 | -0.00234900 |
| H | -6.75503000 | 1.72620500 | -0.00123100 |
| C | -1.54689800 | 3.64062900 | 0.08696900 |
| H | -0.67030600 | 4.27982000 | 0.10514800 |
| C | 2.17098300 | 1.94513500 | -1.22882900 |
| H | 2.16448900 | 2.47113700 | -2.17521800 |
| C | -6.72189700 | -0.42192500 | -0.02422400 |
| H | -7.80027200 | -0.51776600 | -0.04037300 |

| | | | |
|---|---|---|---|
| C | 3.74007400 | -2.51902200 | 1.30158900 |
| H | 4.12478800 | -1.53441700 | 1.57511100 |
| H | 4.59123000 | -3.19752000 | 1.22927000 |
| H | 3.08376100 | -2.87206700 | 2.09390400 |
| C | -2.82263700 | 4.22422500 | 0.07464800 |
| H | -2.91580100 | 5.30280800 | 0.08242600 |
| C | 4.05107400 | -2.02921200 | -1.10119800 |
| H | 3.62758600 | -2.02225600 | -2.10286900 |
| H | 4.88436500 | -2.73329000 | -1.09350100 |
| H | 4.45470400 | -1.03971400 | -0.87243900 |
| C | 1.88835900 | 0.03611100 | 2.59463100 |
| C | 1.75173300 | -0.02320500 | -2.59155300 |
| C | 2.58319300 | 4.12985100 | -0.07602700 |
| C | 0.85312000 | -3.61722900 | -0.07354500 |
| C | 0.47628700 | -4.18585000 | 1.29910000 |
| H | -0.56225400 | -3.94415400 | 1.53234000 |
| H | 0.58746200 | -5.27208200 | 1.27969900 |
| H | 1.10314100 | -3.78545600 | 2.09648000 |
| C | -0.06002100 | -4.21590100 | -1.13932400 |
| H | 0.12377000 | -5.28980100 | -1.21918200 |
| H | -1.10741600 | -4.05807600 | -0.87494300 |
| H | 0.11964600 | -3.75676300 | -2.11337100 |
| F | 2.12047400 | 4.72755200 | 1.02134100 |
| F | 3.89474000 | 4.38688000 | -0.14724800 |
| F | 2.00886300 | 4.70205000 | -1.13333100 |
| F | 3.06540600 | 0.00901700 | 3.23838700 |
| F | 1.08265900 | 0.82799200 | 3.30094300 |
| F | 2.85835700 | 0.10842000 | -3.33929700 |
| F | 1.40957100 | -1.31022700 | -2.62412600 |
| F | 0.78457900 | 0.65565500 | -3.20503100 |
| F | 1.37692000 | -1.18975000 | 2.64583800 |

71

m-2-1

| | | | |
|---|---|---|---|
| Au | -1.00708600 | -0.92245100 | 0.05278600 |

| | | | |
|---|---|---|---|
| N | -2.63394000 | 0.23074700 | 0.05958300 |
| C | -4.77938100 | 1.05470700 | -0.00278400 |
| C | 2.06945600 | 0.84062900 | 1.16296600 |
| N | 1.85332300 | -1.33451700 | 0.01749700 |
| C | 3.06241200 | -2.22808600 | -0.01000300 |
| C | 2.24145400 | 2.21946400 | 1.12084900 |
| H | 2.31901100 | 2.77277300 | 2.04797400 |
| C | -5.92745400 | -1.47228900 | -0.00579000 |
| H | -6.39419500 | -2.45007800 | -0.00726100 |
| C | 0.68443800 | -1.93538000 | 0.03373300 |
| C | -2.57193500 | 1.60924000 | 0.04746500 |
| C | 1.92091100 | 0.79727000 | -1.23486600 |
| C | 2.28060600 | 2.88439200 | -0.08887000 |
| C | -3.97153600 | -0.10646500 | 0.02956300 |
| C | 1.95597000 | 0.09773800 | -0.01969100 |
| C | -4.01891800 | 3.55930800 | -0.01242700 |
| H | -5.00820400 | 4.00229700 | -0.04331300 |
| C | -1.43863700 | 2.42405900 | 0.07109000 |
| H | -0.45059800 | 1.97508100 | 0.11105800 |
| C | -3.86703900 | 2.17292300 | 0.00782700 |
| C | -4.54748300 | -1.37770100 | 0.02793500 |
| H | -3.92030000 | -2.26297000 | 0.05310500 |
| C | 2.40355000 | -3.59699100 | 0.22644000 |
| H | 2.57952900 | -3.90978100 | 1.25773600 |
| H | 2.84119500 | -4.35319800 | -0.42820000 |
| C | -6.16847900 | 0.93588200 | -0.03659600 |
| H | -6.79454800 | 1.82092500 | -0.06156200 |
| C | -1.60999700 | 3.79704200 | 0.04939000 |
| H | -0.74107700 | 4.44673100 | 0.06850500 |
| C | 2.09457400 | 2.17654900 | -1.26041600 |
| H | 2.05593300 | 2.69821700 | -2.20783100 |
| C | -6.73677600 | -0.32666400 | -0.03794300 |
| H | -7.81375300 | -0.43529500 | -0.06388700 |
| C | 4.04900400 | -1.86580600 | 1.08986300 |

| | | | |
|---|---|---|---|
| H | 4.41970300 | -0.84391700 | 0.97743500 |
| H | 4.90615200 | -2.53650400 | 1.01373400 |
| H | 3.61477500 | -1.98392700 | 2.08035300 |
| C | -2.89198900 | 4.36542800 | 0.00701400 |
| H | -2.99752100 | 5.44278600 | -0.00867400 |
| C | 3.77274300 | -2.12139200 | -1.35398200 |
| H | 3.13595400 | -2.41799300 | -2.18463700 |
| H | 4.64472900 | -2.77655300 | -1.33801300 |
| H | 4.12833900 | -1.10313400 | -1.52780000 |
| C | 1.98011900 | 0.29898700 | 2.57779600 |
| C | 1.66395600 | 0.20894200 | -2.61208000 |
| C | 2.51741200 | 4.37272500 | -0.12948700 |
| C | 0.89565700 | -3.42462100 | -0.03027300 |
| C | 0.00844200 | -4.16493400 | 0.97944500 |
| H | -1.02316600 | -4.11441000 | 0.61200000 |
| H | 0.29960700 | -5.21905600 | 0.95962800 |
| C | 0.50703300 | -3.85379100 | -1.46596900 |
| H | -0.47363500 | -3.42599000 | -1.69728600 |
| H | 1.21353200 | -3.40638300 | -2.16773100 |
| F | 2.05044400 | 4.97142000 | 0.96580000 |
| F | 3.82588100 | 4.64387600 | -0.20446100 |
| F | 1.93435900 | 4.93231600 | -1.18847600 |
| F | 3.17855200 | 0.36257300 | 3.17820200 |
| F | 1.14988000 | 1.05574300 | 3.29538100 |
| F | 2.79470500 | 0.20274500 | -3.33521000 |
| F | 1.18231000 | -1.02877100 | -2.61885500 |
| F | 0.79114200 | 0.97162900 | -3.26897500 |
| F | 1.55079600 | -0.95392200 | 2.67618800 |
| C | 0.47848000 | -5.36300300 | -1.66779900 |
| H | -0.34902800 | -5.82670200 | -1.13155300 |
| H | 0.35760800 | -5.59664100 | -2.72545500 |
| H | 1.40441000 | -5.83427500 | -1.32806800 |
| C | 0.06430400 | -3.63878300 | 2.40520900 |
| H | -0.35818300 | -2.63512100 | 2.46860300 |

| | | | |
|---|---|---|---|
| H | -0.50742900 | -4.28414100 | 3.07193600 |
| H | 1.08724200 | -3.59746300 | 2.78754400 |

80

m-3-1

| | | | |
|---|---|---|---|
| Au | 0.55724600 | 0.81920500 | 0.10026500 |
| N | -0.35145900 | 2.59084000 | 0.07579600 |
| C | -0.78836100 | 4.84631300 | -0.00210900 |
| C | -1.54192300 | -1.76952100 | -1.25711200 |
| N | 0.58051500 | -2.03746300 | -0.01617800 |
| C | 1.24305400 | -3.37922900 | -0.03693600 |
| C | -2.93174000 | -1.73235400 | -1.25934100 |
| H | -3.45850200 | -1.61834500 | -2.19819500 |
| C | 1.90111600 | 5.53228800 | -0.08485000 |
| H | 2.94506000 | 5.82012200 | -0.12001000 |
| C | 1.36684800 | -0.98884800 | 0.09132700 |
| C | -1.71842600 | 2.76994900 | 0.08913400 |
| C | -1.56612900 | -1.88828400 | 1.14569600 |
| C | -3.63747600 | -1.80369100 | -0.07271000 |
| C | 0.21417800 | 3.84783400 | 0.01709500 |
| C | -0.84287800 | -1.89986900 | -0.05224400 |
| C | -3.38776500 | 4.53257200 | 0.04676300 |
| H | -3.65391300 | 5.58319900 | 0.01193800 |
| C | -2.71503700 | 1.79499400 | 0.14037600 |
| H | -2.43902300 | 0.74611000 | 0.18215600 |
| C | -2.04835800 | 4.14306200 | 0.04329400 |
| C | 1.56655500 | 4.19062900 | -0.02823400 |
| H | 2.33000900 | 3.41705600 | -0.02288500 |
| C | 2.63083300 | -2.97611700 | 0.45408000 |
| H | 3.39758400 | -3.60189800 | 0.00113100 |
| H | 2.67749700 | -3.12372300 | 1.53832800 |
| C | -0.42806200 | 6.19208500 | -0.05964700 |
| H | -1.18943200 | 6.96405700 | -0.07402600 |
| C | -4.03777200 | 2.20106900 | 0.14098000 |
| H | -4.82747800 | 1.45814600 | 0.18091200 |

| | | | |
|---|---|---|---|
| C | -2.95494600 | -1.85223700 | 1.12881400 |
| H | -3.49821700 | -1.83481300 | 2.06532700 |
| C | 0.91454500 | 6.52952400 | -0.09904000 |
| H | 1.20960000 | 7.57033300 | -0.14355100 |
| C | 1.25000800 | -3.95866200 | -1.44701700 |
| H | 0.23234200 | -4.10551900 | -1.81488800 |
| H | 1.73406900 | -4.93596900 | -1.41629200 |
| H | 1.78984400 | -3.32759300 | -2.14940800 |
| C | -4.37587900 | 3.56209500 | 0.09428300 |
| H | -5.41877800 | 3.85274200 | 0.09684700 |
| C | 0.55256800 | -4.37119800 | 0.88780300 |
| H | 0.53557100 | -4.03109400 | 1.92075200 |
| H | 1.10742200 | -5.30995200 | 0.85368100 |
| H | -0.46813000 | -4.58035800 | 0.55857600 |
| C | -0.92377300 | -1.58908600 | -2.63203100 |
| C | -0.98317700 | -1.82891100 | 2.54784000 |
| C | -5.14480000 | -1.81223600 | -0.08932600 |
| F | -5.62643900 | -1.13489900 | -1.13066100 |
| F | -5.61347500 | -3.06301000 | -0.17650700 |
| F | -5.64910700 | -1.27665900 | 1.02223400 |
| F | -1.12983300 | -2.68078200 | -3.38554600 |
| F | -1.51587600 | -0.57271600 | -3.25750900 |
| F | -1.36406100 | -2.90883900 | 3.24781000 |
| F | 0.34473600 | -1.76192400 | 2.62735300 |
| F | -1.46937700 | -0.76308700 | 3.18189100 |
| F | 0.38278500 | -1.34536900 | -2.63443100 |
| C | 2.80128300 | -1.46583400 | 0.16725100 |
| C | 3.53760500 | -1.14845600 | -1.16722400 |
| C | 3.60951200 | -0.79636700 | 1.30394100 |
| H | 2.96616200 | -1.56268200 | -2.00321500 |
| C | 4.94065300 | -1.76290800 | -1.13925400 |
| C | 3.69715300 | 0.36338400 | -1.36848100 |
| H | 3.08307500 | -0.97994600 | 2.24777000 |
| C | 4.99837000 | -1.44559900 | 1.34379000 |

| | | | |
|---|---|---|---|
| C | 3.79764800 | 0.70642000 | 1.10032300 |
| H | 5.44148700 | -1.51847700 | -2.08180300 |
| H | 4.90627500 | -2.85334900 | -1.07657900 |
| C | 5.73800700 | -1.18265800 | 0.02939600 |
| H | 2.72351200 | 0.85672500 | -1.44355000 |
| H | 4.21570000 | 0.52973300 | -2.31850200 |
| C | 4.51012300 | 0.96841400 | -0.22420900 |
| H | 4.92522700 | -2.52208900 | 1.52772700 |
| H | 5.56377200 | -1.01623400 | 2.17673900 |
| H | 2.83957300 | 1.23227300 | 1.13335600 |
| H | 4.40123500 | 1.09366000 | 1.92826600 |
| H | 6.72343500 | -1.65496900 | 0.06713200 |
| C | 5.89401000 | 0.32301800 | -0.17912800 |
| H | 4.60680000 | 2.04655100 | -0.38058600 |
| H | 6.48135000 | 0.75673900 | 0.63701500 |
| H | 6.43149000 | 0.51477100 | -1.11388600 |

72

m-4-1

| | | | |
|---|---|---|---|
| Au | 0.70108600 | 1.03239300 | -0.01635400 |
| N | -0.54597200 | 2.58810300 | -0.04292000 |
| C | -1.49275400 | 4.68321300 | -0.02492000 |
| C | -0.83516700 | -2.09256400 | -1.19177400 |
| N | 1.30079800 | -1.79553100 | 0.01835700 |
| C | 2.27010300 | -2.94001700 | 0.07239900 |
| C | -2.20246500 | -2.34554000 | -1.16717000 |
| H | -2.74646000 | -2.41851200 | -2.09999700 |
| C | 0.96338100 | 5.97487100 | 0.03195900 |
| H | 1.91245400 | 6.49706700 | 0.05442400 |
| C | 1.81969500 | -0.58939500 | 0.03142000 |
| C | -1.91844200 | 2.44746200 | -0.06397500 |
| C | -0.82170100 | -2.02574000 | 1.21380500 |
| C | -2.87230500 | -2.46242100 | 0.03605300 |
| C | -0.28674900 | 3.94315400 | -0.01782300 |
| C | -0.11741000 | -1.98355600 | 0.00474500 |

| | | | |
|---|---|---|---|
| C | -3.94931200 | 3.77965300 | -0.07106900 |
| H | -4.44923600 | 4.74188900 | -0.06373800 |
| C | -2.66565500 | 1.26879400 | -0.08996300 |
| H | -2.15922100 | 0.30844000 | -0.09811100 |
| C | -2.55639300 | 3.70810600 | -0.05438900 |
| C | 0.94886400 | 4.59146200 | 0.01077900 |
| H | 1.86901000 | 4.01599100 | 0.01600100 |
| C | 3.55562700 | -2.18614600 | 0.43991300 |
| H | 4.41445300 | -2.60814100 | -0.08398500 |
| H | 3.73820000 | -2.28533700 | 1.51431600 |
| C | -1.45461800 | 6.07701300 | -0.00354800 |
| H | -2.37438600 | 6.65134500 | -0.00898200 |
| C | -4.04625300 | 1.36030500 | -0.10571200 |
| H | -4.64395900 | 0.45494400 | -0.12659600 |
| C | -2.18878400 | -2.27286700 | 1.22300400 |
| H | -2.72017000 | -2.29163700 | 2.16620800 |
| C | -0.22716000 | 6.71712500 | 0.02476200 |
| H | -0.18110700 | 7.79877300 | 0.04155200 |
| C | 2.35433200 | -3.63298300 | -1.28212100 |
| H | 1.38855000 | -4.05612900 | -1.56591000 |
| H | 3.06620900 | -4.45617400 | -1.20619000 |
| H | 2.68817800 | -2.95896500 | -2.06839000 |
| C | -4.68837400 | 2.60772600 | -0.09639400 |
| H | -5.77006800 | 2.65098100 | -0.10918100 |
| C | 1.86839300 | -3.96815900 | 1.11961300 |
| H | 1.83729400 | -3.54501800 | 2.12098000 |
| H | 2.61033800 | -4.76795000 | 1.11554300 |
| H | 0.89955100 | -4.41713500 | 0.88683200 |
| C | -0.27355900 | -1.89417000 | -2.58889800 |
| C | -0.25021200 | -1.76371200 | 2.59788600 |
| C | -4.34571200 | -2.78177200 | 0.05654200 |
| F | -4.95672700 | -2.34516400 | -1.04427900 |
| F | -4.54519700 | -4.10347900 | 0.12547900 |
| F | -4.94951600 | -2.23419300 | 1.11056100 |

| | | | |
|---|---|---|---|
| F | -0.19489900 | -3.06627000 | -3.23790300 |
| F | -1.09634300 | -1.11941700 | -3.29484600 |
| F | -0.33346000 | -2.87589200 | 3.34459900 |
| F | 1.01852400 | -1.36027000 | 2.63841900 |
| F | -0.97839400 | -0.83069700 | 3.20798100 |
| F | 0.92955500 | -1.33140700 | -2.63350600 |
| C | 3.31439400 | -0.70179700 | 0.10362000 |
| C | 3.88733100 | -0.26959800 | -1.26084500 |
| C | 3.85821600 | 0.23844200 | 1.18570500 |
| C | 5.41153400 | -0.24115100 | -1.24840900 |
| H | 3.50278200 | 0.73403000 | -1.47726700 |
| H | 3.51548300 | -0.92605700 | -2.05181600 |
| C | 5.38032300 | 0.21476100 | 1.21246300 |
| H | 3.50998800 | 1.25393500 | 0.96213100 |
| H | 3.43355500 | -0.03973900 | 2.15471900 |
| C | 5.93521200 | 0.65802900 | -0.13555000 |
| H | 5.77357900 | 0.09701300 | -2.22125200 |
| H | 5.80373200 | -1.25474500 | -1.10587100 |
| H | 5.74706700 | 0.85927100 | 2.01323500 |
| H | 5.73216200 | -0.80015400 | 1.43738400 |
| H | 7.02686300 | 0.65558900 | -0.12525700 |
| H | 5.61739700 | 1.68929300 | -0.32809800 |

70

m-5-1

| | | | |
|---|---|---|---|
| Au | -0.23801200 | -1.25575300 | 0.01656200 |
| N | -2.22126500 | -1.45777300 | 0.03126500 |
| C | -4.38047600 | -2.24753400 | 0.01678100 |
| C | 0.90792600 | 2.03150500 | 1.19548200 |
| N | 2.20007400 | 0.30299100 | -0.00719300 |
| C | 3.69821400 | 0.41199100 | -0.05036500 |
| C | 0.12918400 | 3.18335100 | 1.16887600 |
| H | -0.20577000 | 3.62059300 | 2.10054100 |
| C | -3.58507900 | -4.90625500 | -0.02058600 |
| H | -3.29405000 | -5.94979700 | -0.03509800 |

| | | | |
|---|---|---|---|
| C | 1.70113600 | -0.91151000 | -0.01520900 |
| C | -3.08128400 | -0.37864000 | 0.04489700 |
| C | 0.88378700 | 1.97979300 | -1.21059900 |
| C | -0.24991800 | 3.74528300 | -0.03571000 |
| C | -3.00801100 | -2.59149100 | 0.01322800 |
| C | 1.34075800 | 1.44671600 | 0.00000300 |
| C | -5.45398500 | 0.13984200 | 0.04779600 |
| H | -6.49118200 | -0.17646600 | 0.04187000 |
| C | -2.76208700 | 0.98004500 | 0.06320300 |
| H | -1.72153500 | 1.29032000 | 0.06963500 |
| C | -4.42813200 | -0.80516800 | 0.03699700 |
| C | -2.60668100 | -3.92798500 | -0.00554000 |
| H | -1.55192000 | -4.18322100 | -0.00774800 |
| C | 4.05917400 | -1.03676600 | -0.40657000 |
| H | 4.95755300 | -1.37033000 | 0.11630400 |
| H | 4.24951400 | -1.09921000 | -1.48255200 |
| C | -5.34979400 | -3.24994200 | 0.00155800 |
| H | -6.40363100 | -2.99442800 | 0.00441700 |
| C | -3.79364000 | 1.90233400 | 0.07341400 |
| H | -3.56539700 | 2.96299500 | 0.08836500 |
| C | 0.10130300 | 3.12779600 | -1.22202100 |
| H | -0.25246100 | 3.52199000 | -2.16637800 |
| C | -4.94838900 | -4.57491300 | -0.01704300 |
| H | -5.68896400 | -5.36469200 | -0.02884200 |
| C | 4.24312800 | 0.85804100 | 1.30085900 |
| H | 3.87246700 | 1.85182600 | 1.56084100 |
| H | 5.33012900 | 0.91845000 | 1.23220800 |
| H | 3.98580700 | 0.16787500 | 2.10113700 |
| C | -5.13399500 | 1.48803700 | 0.06584000 |
| H | -5.92185400 | 2.23052300 | 0.07421200 |
| C | 4.15627800 | 1.40786300 | -1.10602200 |
| H | 3.84077200 | 1.12113100 | -2.10642500 |
| H | 5.24664500 | 1.44074700 | -1.09363600 |
| H | 3.79440400 | 2.41578000 | -0.88790100 |

| | | | |
|---|---|---|---|
| C | 1.15599700 | 1.48953300 | 2.59267100 |
| C | 1.10811000 | 1.39086000 | -2.59374400 |
| C | -1.05329900 | 5.02103000 | -0.05829000 |
| F | -1.80039400 | 5.14896400 | 1.03764800 |
| F | -0.24816000 | 6.08847100 | -0.11877600 |
| F | -1.85958800 | 5.07193400 | -1.11785300 |
| F | 2.04811200 | 2.25151500 | 3.24382300 |
| F | 0.02565400 | 1.53510200 | 3.29658500 |
| F | 1.85564600 | 2.22667500 | -3.33104300 |
| F | 1.70283400 | 0.19957600 | -2.62922800 |
| F | -0.06203300 | 1.26408300 | -3.21646500 |
| F | 1.59476800 | 0.23527800 | 2.63718600 |
| C | 2.83041300 | -1.89626000 | -0.06168800 |
| C | 2.93219100 | -2.57991700 | 1.31143900 |
| C | 2.56305400 | -2.97774800 | -1.11204100 |
| C | 3.85416900 | -3.76263300 | 1.26049300 |
| H | 1.92828000 | -2.88459000 | 1.63168600 |
| H | 3.28139900 | -1.86597600 | 2.06295700 |
| C | 3.76237600 | -3.90955900 | -1.22225900 |
| H | 1.67562400 | -3.53947300 | -0.80279100 |
| H | 2.33429300 | -2.51280500 | -2.07496000 |
| C | 4.24345500 | -4.34404700 | 0.12953700 |
| H | 4.21466700 | -4.14204100 | 2.21052700 |
| H | 3.49674000 | -4.78527800 | -1.81899200 |
| H | 4.58048900 | -3.42138300 | -1.76645500 |
| H | 4.93246400 | -5.18084400 | 0.17096400 |

84

m-6-1

| | | | |
|---|---|---|---|
| Au | 0.50584900 | 0.94326300 | -0.20533400 |
| N | -0.54887000 | 2.63169400 | -0.30601700 |
| C | -1.23096600 | 4.82623200 | -0.25072600 |
| C | -1.35697400 | -2.10240300 | -1.10031000 |
| N | 0.76399500 | -1.91166800 | 0.16315400 |
| C | 1.60348300 | -3.13920800 | 0.38319100 |

| | | | |
|---|---|---|---|
| C | -2.74332200 | -2.20103200 | -1.12368800 |
| H | -3.25093900 | -2.31121500 | -2.07307100 |
| C | 1.36533700 | 5.80776700 | -0.31172700 |
| H | 2.37102500 | 6.21003800 | -0.33682500 |
| C | 1.42572400 | -0.78769300 | -0.01674800 |
| C | -1.92790300 | 2.65877000 | -0.26245800 |
| C | -1.43646500 | -1.81095600 | 1.28228300 |
| C | -3.47594000 | -2.12398200 | 0.04502100 |
| C | -0.12521500 | 3.94452500 | -0.29753400 |
| C | -0.67207300 | -1.95045200 | 0.11615500 |
| C | -3.77885100 | 4.22918500 | -0.17705200 |
| H | -4.15665400 | 5.24523600 | -0.14920900 |
| C | -2.81379300 | 1.57978900 | -0.25260700 |
| H | -2.43080700 | 0.56415000 | -0.29187400 |
| C | -2.40586700 | 3.98795700 | -0.22707300 |
| C | 1.18086100 | 4.43635000 | -0.32956800 |
| H | 2.02355300 | 3.75288500 | -0.37368900 |
| C | 2.98003700 | -2.48453800 | 0.59229200 |
| H | 3.77462100 | -3.09883900 | 0.16621400 |
| H | 3.15902200 | -2.39686500 | 1.66550300 |
| C | -1.02141000 | 6.20469200 | -0.23450000 |
| H | -1.86305000 | 6.88751100 | -0.19875800 |
| C | -4.17188800 | 1.83977600 | -0.20089900 |
| H | -4.87621800 | 1.01432100 | -0.19500400 |
| C | -2.82336300 | -1.90551500 | 1.24103400 |
| H | -3.38992000 | -1.78209400 | 2.15498100 |
| C | 0.27534900 | 6.68968600 | -0.26419900 |
| H | 0.45397500 | 7.75752400 | -0.25215100 |
| C | 1.52741000 | -4.06942900 | -0.82240800 |
| H | 0.50732400 | -4.43562900 | -0.95983900 |
| H | 2.16401400 | -4.93476100 | -0.63320500 |
| H | 1.85493100 | -3.59767900 | -1.74512100 |
| C | -4.65562900 | 3.15628600 | -0.16251800 |
| H | -5.72332100 | 3.33122200 | -0.12343500 |

| | | | |
|---|---|---|---|
| C | 1.14844600 | -3.92197200 | 1.60686500 |
| H | 1.24212600 | -3.34443700 | 2.52304300 |
| H | 1.78090100 | -4.80626000 | 1.69901200 |
| H | 0.11681100 | -4.26708300 | 1.50045200 |
| C | -0.73881500 | -2.14964600 | -2.48701600 |
| C | -0.91886700 | -1.51334900 | 2.67706000 |
| C | -4.97566800 | -2.27150500 | 0.00543800 |
| F | -5.48397800 | -1.77308900 | -1.12145500 |
| F | -5.32906100 | -3.56093100 | 0.06448700 |
| F | -5.55642500 | -1.65177400 | 1.03162400 |
| F | -0.74287600 | -3.40514400 | -2.96097900 |
| F | -1.46794500 | -1.41959400 | -3.33132900 |
| F | -1.10321700 | -2.57770500 | 3.47375800 |
| F | 0.36586900 | -1.18387300 | 2.74919800 |
| F | -1.61206700 | -0.50768400 | 3.20872000 |
| F | 0.50767100 | -1.69970600 | -2.56206300 |
| C | 2.90472600 | -1.08026600 | -0.02990200 |
| C | 3.33423200 | -1.06583500 | -1.51576400 |
| C | 3.69564900 | 0.03228400 | 0.68867400 |
| C | 4.83715700 | -1.23990800 | -1.71238900 |
| H | 3.02911300 | -0.10334300 | -1.94790700 |
| H | 2.80659700 | -1.83949400 | -2.07406200 |
| C | 5.19715000 | -0.17099500 | 0.52060100 |
| H | 3.43299900 | 0.94221900 | 0.12514500 |
| C | 5.59107100 | -0.16364400 | -0.94707800 |
| H | 5.13476700 | -2.21553900 | -1.30430600 |
| H | 5.72882900 | 0.61586900 | 1.06309700 |
| H | 5.49557300 | -1.12326000 | 0.97837800 |
| H | 6.66886200 | -0.30477000 | -1.06525700 |
| H | 5.34426500 | 0.81232400 | -1.38528100 |
| C | 5.17355500 | -1.20895600 | -3.19581100 |
| H | 6.24482900 | -1.34111100 | -3.35812300 |
| H | 4.64570000 | -1.99185100 | -3.74405900 |
| H | 4.88492000 | -0.24567800 | -3.62519900 |

| | | | |
|---|---|---|---|
| C | 3.27796200 | 0.29592100 | 2.15248000 |
| H | 2.25594700 | -0.07184400 | 2.27571500 |
| C | 4.15738500 | -0.36897200 | 3.21204000 |
| H | 4.28912200 | -1.44241900 | 3.06719700 |
| H | 5.15215600 | 0.08308100 | 3.22727500 |
| H | 3.71738400 | -0.21827400 | 4.19935000 |
| C | 3.25784500 | 1.80088400 | 2.40538600 |
| H | 4.24231700 | 2.23073400 | 2.19238000 |
| H | 2.52269300 | 2.29888300 | 1.76762900 |
| H | 3.01461100 | 2.02492400 | 3.44554700 |

60

n-1-1

| | | | |
|---|---|---|---|
| Au | 0.51996800 | -1.14621200 | 0.30076600 |
| N | 2.25956000 | -0.18214300 | 0.43002300 |
| C | 4.45113500 | 0.44083000 | 0.11699900 |
| C | -1.71909800 | 0.78288800 | -1.29097300 |
| N | -2.35208100 | -1.22976300 | -0.08389800 |
| C | -3.60620900 | -1.98518100 | -0.38791400 |
| C | -1.62662600 | 2.16104700 | -1.34405600 |
| C | 5.35746300 | -2.18046900 | 0.01883800 |
| H | 5.73046100 | -3.19688600 | -0.02082300 |
| C | -1.26484700 | -1.94974500 | 0.08452300 |
| C | 2.32377800 | 1.19719300 | 0.40689500 |
| C | -2.76927700 | 0.98274200 | 0.87216100 |
| C | -2.09061400 | 2.98300400 | -0.33315900 |
| C | 3.55046600 | -0.64105800 | 0.25521400 |
| C | -2.29664200 | 0.19865800 | -0.16834900 |
| C | 3.92162400 | 3.00456400 | 0.12988000 |
| H | 4.93563200 | 3.35456300 | -0.02795400 |
| C | 1.28048900 | 2.11271900 | 0.55728200 |
| H | 0.27488800 | 1.75832900 | 0.76732800 |
| C | 3.65060400 | 1.63880700 | 0.20737400 |
| C | 4.00415000 | -1.95953300 | 0.20472800 |
| H | 3.30635100 | -2.78344100 | 0.31292700 |

| | | | |
|---|---|---|---|
| C | -3.16058900 | -3.41595100 | -0.05654400 |
| H | -3.55801100 | -4.12969900 | -0.78007300 |
| H | -3.54122300 | -3.69262900 | 0.92988500 |
| C | 5.81096800 | 0.19541500 | -0.06989200 |
| H | 6.50785200 | 1.01919200 | -0.17670900 |
| C | 1.56839900 | 3.46403300 | 0.46776700 |
| H | 0.77096600 | 4.19108400 | 0.58107100 |
| C | -2.66022400 | 2.36038100 | 0.75949500 |
| C | 6.25849700 | -1.11402800 | -0.11759700 |
| H | 7.31149300 | -1.32097700 | -0.26111600 |
| C | -3.95568100 | -1.78127100 | -1.85743700 |
| H | -4.16520800 | -0.72921300 | -2.06401900 |
| H | -4.84970100 | -2.35631300 | -2.10164700 |
| H | -3.14428200 | -2.10887800 | -2.50866900 |
| C | 2.87999600 | 3.91073900 | 0.25037600 |
| H | 3.07713500 | 4.97306400 | 0.18540700 |
| C | -4.75693200 | -1.52160300 | 0.49054200 |
| H | -4.49267500 | -1.58132700 | 1.54784000 |
| H | -5.61539400 | -2.17207000 | 0.31673800 |
| H | -5.05931100 | -0.49972200 | 0.25311700 |
| C | -1.62112000 | -3.40741200 | -0.03150800 |
| C | -1.00008000 | -3.92250500 | -1.33559100 |
| H | 0.08646100 | -3.82735000 | -1.29898300 |
| H | -1.25732000 | -4.97608300 | -1.46381200 |
| H | -1.35797300 | -3.37122300 | -2.20695600 |
| C | -1.04813100 | -4.19504200 | 1.14253200 |
| H | -1.33788500 | -5.24478200 | 1.05819400 |
| H | 0.04153400 | -4.13149600 | 1.14511400 |
| H | -1.41554600 | -3.80925700 | 2.09519500 |
| H | -1.32306000 | 0.17775700 | -2.09610600 |
| H | -1.99913400 | 4.05776300 | -0.39214500 |
| H | -3.19384000 | 0.54826700 | 1.76595300 |
| N | -0.99230600 | 2.78134300 | -2.52147700 |
| O | -0.86944700 | 3.98675500 | -2.50945800 |

| | | | |
|---|---|---|---|
| O | -0.65094900 | 2.03827500 | -3.41764800 |
| N | -3.16705200 | 3.19463300 | 1.86398100 |
| O | -3.02411200 | 4.39370800 | 1.76342900 |
| O | -3.69743100 | 2.61854400 | 2.79239700 |

58

o-1-1

| | | | |
|---|---|---|---|
| Au | -0.27025800 | -0.96494000 | -0.18917600 |
| N | -2.13327300 | -0.26159100 | -0.23115500 |
| C | -4.40702500 | 0.00533100 | -0.01991100 |
| C | 1.91399600 | 1.23404900 | 1.30325400 |
| N | 2.61155600 | -0.67451800 | -0.02563100 |
| C | 3.97663200 | -1.26384800 | 0.13063000 |
| C | 1.66294300 | 2.59857700 | 1.43305000 |
| C | -4.88222800 | -2.72470800 | 0.13550500 |
| H | -5.08767800 | -3.78671000 | 0.19706600 |
| C | 1.61479600 | -1.52668100 | -0.11278400 |
| C | -2.41873500 | 1.08918300 | -0.25888400 |
| C | 2.59524900 | 1.57495200 | -0.98453900 |
| C | 1.87935000 | 3.45892500 | 0.35832000 |
| C | -3.33867600 | -0.91920500 | -0.08780400 |
| C | 2.38396000 | 0.73485600 | 0.09580800 |
| C | -4.30135600 | 2.61802400 | -0.12247900 |
| H | -5.36507600 | 2.80146300 | -0.01992900 |
| C | -1.53166100 | 2.15771300 | -0.40105100 |
| H | -0.46989700 | 1.96886900 | -0.53482100 |
| C | -3.80730000 | 1.31390500 | -0.12753100 |
| C | -3.57582100 | -2.29203200 | -0.00906800 |
| H | -2.75045300 | -2.99463400 | -0.06275400 |
| C | 3.68637700 | -2.73724700 | -0.18768000 |
| H | 4.24204200 | -3.40102600 | 0.47683200 |
| H | 3.99808800 | -2.95300100 | -1.21276400 |
| C | -5.71597700 | -0.45252000 | 0.12634300 |
| H | -6.54037800 | 0.24982800 | 0.17927900 |
| C | -2.04171300 | 3.44428800 | -0.39010400 |

| | | | |
|---|---|---|---|
| H | -1.36919600 | 4.28763200 | -0.50505800 |
| C | 2.34356600 | 2.94222700 | -0.84745900 |
| C | -5.94824500 | -1.81540100 | 0.20312900 |
| H | -6.95907500 | -2.18646700 | 0.31622600 |
| C | 4.45551700 | -1.03327800 | 1.55912600 |
| H | 4.55126900 | 0.03437100 | 1.76925200 |
| H | 5.43659400 | -1.49120300 | 1.69105700 |
| H | 3.76912200 | -1.47178800 | 2.28472100 |
| C | -3.41753400 | 3.67758800 | -0.24874700 |
| H | -3.78842200 | 4.69460000 | -0.24485100 |
| C | 4.95577500 | -0.64277600 | -0.85259700 |
| H | 4.58994200 | -0.73024900 | -1.87728200 |
| H | 5.90742400 | -1.17184800 | -0.78416500 |
| H | 5.14050000 | 0.40912400 | -0.62564800 |
| C | 2.16386200 | -2.92737100 | -0.06126200 |
| C | 1.74790300 | -3.52385100 | 1.28878200 |
| H | 0.65995200 | -3.56816200 | 1.36203700 |
| H | 2.14821100 | -4.53641600 | 1.37299700 |
| H | 2.11952300 | -2.93474200 | 2.12924900 |
| C | 1.58220300 | -3.77705500 | -1.18649100 |
| H | 2.01284300 | -4.78024100 | -1.15118100 |
| H | 0.49878500 | -3.85692600 | -1.07988300 |
| H | 1.79789500 | -3.34107300 | -2.16363500 |
| H | 1.67881200 | 4.51678000 | 0.45839700 |
| H | 1.72598600 | 0.55721100 | 2.12750900 |
| H | 2.93210600 | 1.17521800 | -1.93162700 |
| C | 1.18513900 | 3.12037000 | 2.68223400 |
| C | 2.56904200 | 3.81745600 | -1.96306200 |
| N | 0.82606200 | 3.53974300 | 3.69278300 |
| N | 2.76583200 | 4.51506000 | -2.85807100 |

64

p-1-1

| | | | |
|---|---|---|---|
| Au | -0.75525400 | 1.41857100 | 0.55221500 |
| N | -2.27977900 | 0.15012200 | 0.87165900 |

| | | | |
|---|---|---|---|
| C | -4.25670400 | -0.91064800 | 0.34850900 |
| C | 1.32871800 | -0.18846400 | -1.46021300 |
| N | 1.95010900 | 1.87230000 | -0.32775400 |
| C | 3.04082500 | 2.76295700 | -0.82810700 |
| C | 1.32158600 | -1.57273700 | -1.51881600 |
| C | -5.68993000 | 1.45858600 | 0.23541000 |
| H | -6.26831400 | 2.37329600 | 0.18796000 |
| C | 0.84862900 | 2.46520400 | 0.06432000 |
| C | -2.04717400 | -1.20457300 | 0.81564100 |
| C | 2.70112600 | -0.29563000 | 0.52186800 |
| C | 2.01038900 | -2.33839700 | -0.58900200 |
| C | -3.62179400 | 0.32848400 | 0.59348100 |
| C | 2.02587700 | 0.44367600 | -0.43506700 |
| C | -3.17963300 | -3.29411000 | 0.29152800 |
| H | -4.07011900 | -3.84162100 | 0.00484400 |
| C | -0.84483000 | -1.88449800 | 1.02873100 |
| H | 0.04579700 | -1.32914400 | 1.31095300 |
| C | -3.22724400 | -1.91184000 | 0.47347800 |
| C | -4.34160300 | 1.52318000 | 0.53356800 |
| H | -3.84736900 | 2.46991100 | 0.72198900 |
| C | 2.51204000 | 4.13426400 | -0.38334200 |
| H | 2.68496300 | 4.89084400 | -1.15045900 |
| H | 3.03896500 | 4.44849600 | 0.52107600 |
| C | -5.61891300 | -0.95322300 | 0.04771800 |
| H | -6.11186600 | -1.90022600 | -0.14075900 |
| C | -0.82074900 | -3.25959800 | 0.84882100 |
| H | 0.10653400 | -3.81187900 | 0.97803200 |
| C | 2.67821900 | -1.68331800 | 0.43209900 |
| C | -6.32935300 | 0.23186800 | -0.00559900 |
| H | -7.38708300 | 0.21882300 | -0.23533800 |
| C | 3.14032100 | 2.60838100 | -2.34099500 |
| H | 3.41220400 | 1.58535000 | -2.60985200 |
| H | 3.91459700 | 3.27470000 | -2.72315200 |
| H | 2.19716100 | 2.85685600 | -2.82959700 |

| | | | |
|---|---|---|---|
| C | -1.97533000 | -3.96432700 | 0.46875600 |
| H | -1.92247900 | -5.03483500 | 0.32116600 |
| C | 4.36865600 | 2.41174700 | -0.17701100 |
| H | 4.29198300 | 2.44162500 | 0.91138300 |
| H | 5.11727400 | 3.14235400 | -0.48675400 |
| H | 4.71716800 | 1.42366700 | -0.48346900 |
| C | 1.01412300 | 3.95397200 | -0.07189700 |
| C | 0.10695100 | 4.40336800 | -1.22356200 |
| H | -0.93613800 | 4.18010100 | -0.99231000 |
| H | 0.21297500 | 5.48105200 | -1.36436500 |
| H | 0.35923700 | 3.90600600 | -2.16173300 |
| C | 0.58875700 | 4.66160200 | 1.21044000 |
| H | 0.73596000 | 5.73848600 | 1.10325500 |
| H | -0.46626400 | 4.47107800 | 1.41558700 |
| H | 1.17208200 | 4.31597900 | 2.06581900 |
| H | 0.76967800 | 0.39610000 | -2.18265700 |
| H | 1.99319500 | -3.41928300 | -0.64414800 |
| H | 3.20679600 | 0.18891000 | 1.34810300 |
| S | 0.32957100 | -2.37562600 | -2.73648800 |
| O | 0.49634200 | -1.68293300 | -3.96166700 |
| O | 0.55750600 | -3.77323100 | -2.61552100 |
| S | 3.37616100 | -2.61862200 | 1.74464400 |
| O | 3.35032400 | -3.99090100 | 1.37747800 |
| O | 4.54885600 | -1.96582900 | 2.19532700 |
| O | 2.31118700 | -2.39046000 | 2.88624500 |
| H | 1.55668100 | -2.99781700 | 2.79365500 |
| O | -1.11850000 | -2.03950000 | -2.25962600 |
| H | -1.40603300 | -2.59766500 | -1.50448300 |

62

q-1-1

| | | | |
|---|---|---|---|
| Au | 1.49041900 | 1.18840800 | -0.56938800 |
| N | 2.50231700 | -0.48585000 | -0.98576000 |
| C | 4.06462800 | -2.13999700 | -0.63984700 |
| C | -1.03918600 | 0.34071200 | 1.35933000 |

| | | | |
|---|---|---|---|
| N | -0.87667400 | 2.54971700 | 0.37897600 |
| C | -1.54603100 | 3.74870300 | 0.96629900 |
| C | -1.55421200 | -0.95440900 | 1.36267900 |
| C | 6.16814800 | -0.34161400 | -0.43569400 |
| H | 7.00254000 | 0.34490900 | -0.35540000 |
| C | 0.36914100 | 2.70938300 | -0.00138500 |
| C | 1.85882500 | -1.70797700 | -1.00958700 |
| C | -2.44976300 | 0.90234800 | -0.51763700 |
| C | -2.52691300 | -1.30553000 | 0.44512000 |
| C | 3.84004900 | -0.74898100 | -0.76524300 |
| C | -1.48263400 | 1.24951800 | 0.41879700 |
| C | 2.31000500 | -4.07808000 | -0.70574800 |
| H | 3.00113900 | -4.89232700 | -0.51800900 |
| C | 0.49996400 | -1.96745000 | -1.20604600 |
| H | -0.19252900 | -1.15464800 | -1.41330000 |
| C | 2.77120900 | -2.76365900 | -0.78188200 |
| C | 4.89703400 | 0.15672800 | -0.65969500 |
| H | 4.71581200 | 1.22198000 | -0.75775800 |
| C | -0.54660100 | 4.84918500 | 0.58393800 |
| H | -0.42102400 | 5.56761100 | 1.39589200 |
| H | -0.92275100 | 5.39195200 | -0.28708500 |
| C | 5.35423000 | -2.62087200 | -0.41327900 |
| H | 5.53414800 | -3.68597500 | -0.31754700 |
| C | 0.06350300 | -3.27819400 | -1.12133000 |
| H | -0.98959100 | -3.49908100 | -1.25943400 |
| C | -2.98264800 | -0.37483800 | -0.49025500 |
| C | 6.40071100 | -1.72003900 | -0.31337700 |
| H | 7.40799600 | -2.07750200 | -0.14018300 |
| C | -1.67825300 | 3.54300400 | 2.47103800 |
| H | -2.30793200 | 2.67748100 | 2.68896900 |
| H | -2.14221400 | 4.42268900 | 2.91916800 |
| H | -0.70406400 | 3.38880300 | 2.93713500 |
| C | 0.95701200 | -4.32922300 | -0.86450900 |
| H | 0.58369800 | -5.34365100 | -0.80153200 |

| | | | |
|---|---|---|---|
| C | -2.91738400 | 3.97377300 | 0.34991200 |
| H | -2.85114600 | 4.04361800 | -0.73744900 |
| H | -3.32290000 | 4.91335200 | 0.72851900 |
| H | -3.61259400 | 3.17513000 | 0.61563800 |
| C | 0.77268700 | 4.14240500 | 0.22130300 |
| C | 1.79099600 | 4.15399600 | 1.36720200 |
| H | 2.67472400 | 3.57561100 | 1.09165300 |
| H | 2.09274200 | 5.18382200 | 1.57040500 |
| H | 1.38080900 | 3.72659500 | 2.28393900 |
| C | 1.42228400 | 4.71715800 | -1.03333800 |
| H | 1.68826700 | 5.76324700 | -0.86529600 |
| H | 2.32790700 | 4.15963100 | -1.27907900 |
| H | 0.74579700 | 4.66454500 | -1.88843700 |
| C | -1.00397800 | -1.92088200 | 2.39718900 |
| C | -3.99040600 | -0.84235800 | -1.52397000 |
| H | -2.92556300 | -2.31074000 | 0.43246700 |
| H | -0.26951400 | 0.63160200 | 2.06541200 |
| H | -2.75180400 | 1.61874700 | -1.26768800 |
| Cl | -1.44632600 | -1.31306700 | 4.01176300 |
| Cl | 0.75295600 | -1.98768400 | 2.28456700 |
| Cl | -1.64332900 | -3.54772900 | 2.22581500 |
| Cl | -5.30799000 | -1.72821900 | -0.74470000 |
| Cl | -4.70175500 | 0.48991800 | -2.43377200 |
| Cl | -3.17114700 | -1.92472200 | -2.65965900 |

62

r-1-1

| | | | |
|---|---|---|---|
| Au | 0.86404900 | 1.20772100 | -0.30502100 |
| N | 2.39917400 | -0.05891700 | -0.44081600 |
| C | 4.44527900 | -1.08231000 | -0.20910200 |
| C | -1.70765300 | -0.27011600 | 1.30161600 |
| N | -1.94764800 | 1.80457700 | 0.06391500 |
| C | -3.05018300 | 2.77314700 | 0.34524400 |
| C | -1.84532100 | -1.64928900 | 1.38999000 |
| C | 5.81692500 | 1.32512400 | -0.04343200 |

| | | | |
|---|---|---|---|
| H | 6.36993900 | 2.25456700 | 0.02201600 |
| C | -0.75030500 | 2.31925200 | -0.10067900 |
| C | 2.21008400 | -1.42641900 | -0.46804100 |
| C | -2.71999600 | -0.31805300 | -0.88710500 |
| C | -2.42718000 | -2.36737600 | 0.35986400 |
| C | 3.75417800 | 0.15022000 | -0.28482500 |
| C | -2.14819700 | 0.38808200 | 0.16369300 |
| C | 3.45727200 | -3.50451400 | -0.30882000 |
| H | 4.39366600 | -4.03927500 | -0.19450200 |
| C | 1.01384800 | -2.12946900 | -0.62306600 |
| H | 0.08252400 | -1.59177700 | -0.77723200 |
| C | 3.43803800 | -2.10987600 | -0.32236900 |
| C | 4.44261700 | 1.36122100 | -0.20033300 |
| H | 3.90466100 | 2.30170300 | -0.25996400 |
| C | -2.35640800 | 4.09990200 | 0.00783900 |
| H | -2.62825900 | 4.87981500 | 0.72123800 |
| H | -2.67245500 | 4.43017200 | -0.98499800 |
| C | 5.83064800 | -1.09519000 | -0.05027200 |
| H | 6.36749200 | -2.03553000 | 0.00848900 |
| C | 1.05335300 | -3.51268900 | -0.59996700 |
| H | 0.13341800 | -4.07490800 | -0.72427800 |
| C | -2.85789100 | -1.69293400 | -0.77607800 |
| C | 6.51087300 | 0.10810100 | 0.03131700 |
| H | 7.58667500 | 0.11439700 | 0.15373100 |
| C | -3.44879200 | 2.65120800 | 1.81144100 |
| H | -3.83884000 | 1.65364400 | 2.02513400 |
| H | -4.23227700 | 3.37602000 | 2.03637200 |
| H | -2.60089700 | 2.84190700 | 2.47074600 |
| C | 2.26598400 | -4.20008700 | -0.44034800 |
| H | 2.26820100 | -5.28266900 | -0.42895500 |
| C | -4.25457500 | 2.50895500 | -0.54404100 |
| H | -3.97104100 | 2.51228700 | -1.59809400 |
| H | -4.98917900 | 3.29998600 | -0.38572300 |
| H | -4.72994000 | 1.55612800 | -0.30328700 |

| | | | |
|---|---|---|---|
| C | -0.84273700 | 3.81854400 | -0.00003100 |
| C | -0.15338700 | 4.22719700 | 1.30709400 |
| H | 0.89873000 | 3.93802700 | 1.28438800 |
| H | -0.21871200 | 5.31102300 | 1.42480700 |
| H | -0.61409800 | 3.75688200 | 2.17771600 |
| C | -0.12781300 | 4.48152300 | -1.17293300 |
| H | -0.22709600 | 5.56688800 | -1.09967300 |
| H | 0.93307600 | 4.22490300 | -1.16345500 |
| H | -0.54887800 | 4.15820700 | -2.12669200 |
| C | -1.33755000 | -2.34417100 | 2.62774400 |
| C | -3.46264600 | -2.50126100 | -1.89396300 |
| H | -2.51749500 | -3.44427800 | 0.42409100 |
| H | -1.22449300 | 0.29106500 | 2.09336300 |
| H | -3.02364500 | 0.19507500 | -1.78943100 |
| F | -0.13108700 | -1.89854900 | 2.97144600 |
| F | -2.15258400 | -2.11304300 | 3.66893000 |
| F | -1.26525800 | -3.66287400 | 2.46371700 |
| F | -3.71817400 | -1.75589700 | -2.97029900 |
| F | -2.65133800 | -3.49341300 | -2.26634700 |
| F | -4.61627600 | -3.06246500 | -1.51116300 |

73

s-1-1

| | | | |
|---|---|---|---|
| Au | 1.33815700 | 1.17875500 | -0.56758000 |
| N | 2.40423500 | -0.48946000 | -0.91417600 |
| C | 3.99012400 | -2.07560500 | -0.38644400 |
| C | -1.21340800 | 0.10920600 | 1.44804900 |
| N | -1.05983500 | 2.34570300 | 0.46568300 |
| C | -1.83329100 | 3.48258500 | 1.06354200 |
| C | -1.77947700 | -1.17614300 | 1.40501800 |
| C | 6.06215100 | -0.23487400 | -0.36239300 |
| H | 6.88591100 | 0.46857200 | -0.34974200 |
| C | 0.13705700 | 2.63316400 | 0.02319000 |
| C | 1.78164200 | -1.71354100 | -0.81135100 |
| C | -2.30189600 | 0.60525300 | -0.69789200 |

| | | | |
|---|---|---|---|
| C | -2.73922100 | -1.53415100 | 0.44741500 |
| C | 3.74474600 | -0.71040300 | -0.65995500 |
| C | -1.56972500 | 0.99949900 | 0.42981900 |
| C | 2.26225300 | -4.03414000 | -0.25444200 |
| H | 2.95966100 | -4.81333700 | 0.03108500 |
| C | 0.42861000 | -2.01522700 | -0.99184500 |
| H | -0.27057900 | -1.23247800 | -1.27294800 |
| C | 2.70815000 | -2.73000400 | -0.46729300 |
| C | 4.78699700 | 0.21860000 | -0.64477900 |
| H | 4.59067000 | 1.26426300 | -0.85489500 |
| C | -0.97493500 | 4.67125300 | 0.60833200 |
| H | -0.90191300 | 5.43029200 | 1.38907200 |
| H | -1.43730800 | 5.13496400 | -0.26747600 |
| C | 5.28501500 | -2.51209600 | -0.10283400 |
| H | 5.47979900 | -3.55779100 | 0.10705500 |
| C | 0.00665600 | -3.31961000 | -0.78352800 |
| H | -1.04441500 | -3.56921500 | -0.88506600 |
| C | -2.88833300 | -0.66795300 | -0.65312000 |
| C | 6.31531100 | -1.58961300 | -0.09359200 |
| H | 7.32658400 | -1.90983600 | 0.12234400 |
| C | -1.88953200 | 3.32517900 | 2.57959200 |
| H | -2.37268500 | 2.38446000 | 2.85330100 |
| H | -2.48043200 | 4.14247100 | 2.99507100 |
| H | -0.89943100 | 3.35452700 | 3.03256200 |
| C | 0.91178100 | -4.32535300 | -0.40303300 |
| H | 0.55005100 | -5.33055400 | -0.23040300 |
| C | -3.25689800 | 3.53915800 | 0.52840200 |
| H | -3.28592300 | 3.70927700 | -0.54649800 |
| H | -3.77456200 | 4.36728200 | 1.01443100 |
| H | -3.80222700 | 2.62147600 | 0.75976100 |
| C | -0.24602700 | 0.52020200 | 2.52242300 |
| H | -0.73034500 | 0.51508900 | 3.49876100 |
| H | 0.58360700 | -0.18220100 | 2.57224100 |
| H | 0.16824500 | 1.50402400 | 2.32843300 |

| | | | |
|---|---|---|---|
| C | -2.37387600 | 1.52009700 | -1.89092000 |
| H | -1.83951000 | 1.07268100 | -2.72793900 |
| H | -3.39789200 | 1.68516800 | -2.22237700 |
| H | -1.91225400 | 2.47846600 | -1.67700400 |
| C | -3.61649600 | -2.74999100 | 0.60653900 |
| H | -3.86809200 | -2.88319900 | 1.65506300 |
| H | -4.53788600 | -2.62823500 | 0.04601200 |
| H | -3.12309600 | -3.66259400 | 0.27869700 |
| C | 0.40219700 | 4.10070600 | 0.21672200 |
| C | 1.45156700 | 4.23881700 | 1.32468300 |
| H | 2.37510800 | 3.73431400 | 1.03427800 |
| H | 1.66638400 | 5.29656700 | 1.49053400 |
| H | 1.11175900 | 3.80487900 | 2.26708200 |
| C | 0.94121500 | 4.70981800 | -1.07365100 |
| H | 1.09057700 | 5.78385100 | -0.94265900 |
| H | 1.89550600 | 4.25152200 | -1.33954400 |
| H | 0.24544800 | 4.55567900 | -1.90170000 |
| S | -1.18520000 | -2.37522700 | 2.58471300 |
| O | -1.31817300 | -1.83300200 | 3.88922600 |
| O | -1.70129000 | -3.66260300 | 2.27667500 |
| S | -3.83663400 | -1.24155000 | -2.04136700 |
| O | -5.21002400 | -1.28173900 | -1.69447300 |
| O | -3.40744400 | -0.58358000 | -3.22791700 |
| O | -3.36111400 | -2.74077000 | -2.18020900 |
| H | -2.59388900 | -2.78180000 | -2.77597300 |
| O | 0.35073500 | -2.39992100 | 2.28067300 |
| H | 0.55158500 | -2.95153100 | 1.49142900 |

71

t-1-1

| | | | |
|---|---|---|---|
| Au | 1.22002900 | 1.04765100 | -0.06351600 |
| N | 2.58956400 | -0.40064000 | -0.03344000 |
| C | 4.55974100 | -1.58386400 | 0.03059400 |
| C | -2.06759600 | 0.13226700 | 1.22944900 |
| N | -1.47874300 | 2.09888900 | -0.09639400 |

| | | | |
|---|---|---|---|
| C | -2.43516600 | 3.25156400 | -0.11384300 |
| C | -2.52947500 | -1.18913000 | 1.26719800 |
| C | 6.12666200 | 0.70450300 | 0.13221000 |
| H | 6.75482000 | 1.58643400 | 0.17212500 |
| C | -0.20549500 | 2.40718500 | -0.09893100 |
| C | 2.28954700 | -1.74796100 | -0.06857700 |
| C | -2.14041300 | 0.03629600 | -1.22332500 |
| C | -2.71581700 | -1.94111900 | 0.10088900 |
| C | 3.96475800 | -0.30071200 | 0.02550300 |
| C | -1.92628000 | 0.73151500 | -0.02743500 |
| C | 3.38565800 | -3.91803100 | -0.06148200 |
| H | 4.28563800 | -4.52229300 | -0.03334100 |
| C | 1.03385000 | -2.35571800 | -0.13755600 |
| H | 0.13308500 | -1.74809600 | -0.16985300 |
| C | 3.46891200 | -2.52627700 | -0.03134000 |
| C | 4.75181700 | 0.85092800 | 0.07654400 |
| H | 4.28758800 | 1.83206300 | 0.07220800 |
| C | -1.47541000 | 4.41881300 | -0.38828900 |
| H | -1.73187800 | 5.29228300 | 0.21388500 |
| H | -1.54661200 | 4.70615200 | -1.44055000 |
| C | 5.94747200 | -1.70861300 | 0.08738200 |
| H | 6.41063100 | -2.68912300 | 0.09183600 |
| C | 0.97453900 | -3.73829900 | -0.16877600 |
| H | 0.01053800 | -4.23050400 | -0.22937800 |
| C | -2.59311300 | -1.28550600 | -1.13132400 |
| C | 6.72537700 | -0.56424600 | 0.13800300 |
| H | 7.80424200 | -0.64455700 | 0.18246000 |
| C | -3.14735400 | 3.36533400 | 1.22812000 |
| H | -3.72988400 | 2.46466000 | 1.43467800 |
| H | -3.83492000 | 4.21141800 | 1.19101800 |
| H | -2.44566900 | 3.52812600 | 2.04583600 |
| C | 2.13960300 | -4.51874600 | -0.13007300 |
| H | 2.05978500 | -5.59817500 | -0.15649100 |
| C | -3.47000100 | 3.08555800 | -1.21587100 |

| | | | |
|---|---|---|---|
| H | -2.99938900 | 3.05274800 | -2.19764700 |
| H | -4.14675700 | 3.94102700 | -1.19146800 |
| H | -4.06355500 | 2.17997500 | -1.07092700 |
| C | -1.58199600 | 0.83715200 | 2.47050100 |
| H | -2.39534500 | 1.26316700 | 3.05567300 |
| H | -1.04857300 | 0.13550400 | 3.10977000 |
| H | -0.88582200 | 1.63072400 | 2.20923200 |
| C | -1.73711000 | 0.65249600 | -2.53840800 |
| H | -1.19736600 | -0.07798500 | -3.13965500 |
| H | -2.59452100 | 0.98664900 | -3.12031300 |
| H | -1.07259600 | 1.49820700 | -2.37694000 |
| C | -2.88288100 | -3.44168900 | 0.16847700 |
| H | -3.92768500 | -3.74195600 | 0.22796500 |
| H | -2.44489000 | -3.90997900 | -0.70889800 |
| H | -2.36346300 | -3.83572900 | 1.03887000 |
| C | -0.05542800 | 3.90493500 | -0.09185100 |
| C | 0.44152500 | 4.30318800 | 1.30236900 |
| H | 1.40161900 | 3.82777100 | 1.51194600 |
| H | 0.56890500 | 5.38704700 | 1.34523500 |
| H | -0.26216100 | 4.00902000 | 2.08417500 |
| C | 0.96006800 | 4.34744800 | -1.13965600 |
| H | 1.02474100 | 5.43777600 | -1.15666300 |
| H | 1.94664200 | 3.94032800 | -0.90919000 |
| H | 0.67362300 | 4.00149800 | -2.13545600 |
| C | -2.90664400 | -2.06345400 | -2.39624000 |
| C | -2.78889600 | -1.84650700 | 2.61078200 |
| F | -3.78687300 | -2.73496000 | 2.55289800 |
| F | -3.16202400 | -0.96461200 | 3.54603500 |
| F | -3.92119600 | -2.91783000 | -2.21880800 |
| F | -3.28698900 | -1.27649300 | -3.40839400 |
| F | -1.85421700 | -2.77261100 | -2.82363100 |
| F | -1.70998000 | -2.48485400 | 3.07925400 |

62

u-1-1

| | | | |
|---|---|---|---|
| Au | -0.18147800 | -0.57498200 | 0.10857500 |
| N | -2.11708200 | -0.06865600 | 0.11049500 |
| C | -4.40528900 | -0.15213900 | -0.10708600 |
| N | 2.74794400 | -0.23023500 | 0.02439600 |
| C | 4.09867700 | -0.87424300 | -0.00506300 |
| C | -4.43348100 | -2.89511300 | -0.52646700 |
| H | -4.46422500 | -3.96555700 | -0.69176000 |
| C | 1.73969600 | -1.06266500 | 0.08593400 |
| C | -2.61467700 | 1.20855500 | 0.26146800 |
| C | -3.20034500 | -0.89324000 | -0.11128600 |
| C | -4.72872700 | 2.41490800 | 0.25186100 |
| H | -5.80878000 | 2.42695000 | 0.15455300 |
| C | -1.91820800 | 2.39320400 | 0.51439800 |
| H | -0.83789900 | 2.38035400 | 0.63231900 |
| C | -4.02415400 | 1.21738900 | 0.13427700 |
| C | -3.21429500 | -2.27353000 | -0.32311100 |
| H | -2.28607800 | -2.83665600 | -0.32517200 |
| C | 3.72192300 | -2.34522600 | -0.24231500 |
| H | 4.36354100 | -3.01766600 | 0.33000400 |
| H | 3.85080600 | -2.58199800 | -1.30212300 |
| C | -5.62344400 | -0.79870300 | -0.31479300 |
| H | -6.55059900 | -0.23611300 | -0.31235300 |
| C | -2.63677300 | 3.57077900 | 0.62673200 |
| H | -2.11219300 | 4.49821200 | 0.82481000 |
| C | -5.63324700 | -2.16740600 | -0.52325000 |
| H | -6.57094200 | -2.68394100 | -0.68569500 |
| C | 4.83189500 | -0.69262100 | 1.32396600 |
| H | 5.18581300 | 0.32730300 | 1.46186700 |
| H | 5.70276300 | -1.35031000 | 1.33128500 |
| H | 4.19473500 | -0.95839200 | 2.16793500 |
| C | -4.03324400 | 3.58761000 | 0.49502300 |
| H | -4.56716100 | 4.52490400 | 0.58899800 |
| C | 4.95874400 | -0.35508700 | -1.14902700 |
| H | 4.43682900 | -0.45063000 | -2.10224400 |

| | | | |
|---|---|---|---|
| H | 5.86664900 | -0.95994800 | -1.19427000 |
| H | 5.25644100 | 0.68420600 | -1.00802700 |
| C | 2.24158000 | -2.48228000 | 0.13166100 |
| C | 2.02509300 | -3.01196800 | 1.55292000 |
| H | 0.96213500 | -2.99638200 | 1.80094100 |
| H | 2.38436100 | -4.04173800 | 1.61407800 |
| H | 2.55560900 | -2.41590500 | 2.29704200 |
| C | 1.47428800 | -3.36163200 | -0.85095200 |
| H | 1.90039300 | -4.36784800 | -0.85484200 |
| H | 0.42255000 | -3.42931600 | -0.56404000 |
| H | 1.52482600 | -2.95862300 | -1.86440300 |
| C | 2.48938500 | 1.22347000 | -0.06532700 |
| C | 3.40517900 | 2.12155100 | 0.75266600 |
| C | 2.39134700 | 1.68908900 | -1.51520200 |
| H | 1.48713900 | 1.33269000 | 0.36439700 |
| C | 2.84879900 | 3.54405700 | 0.70538400 |
| H | 4.42146100 | 2.12318900 | 0.34416500 |
| H | 3.45806400 | 1.76938800 | 1.78506600 |
| C | 1.84661100 | 3.11225600 | -1.55218600 |
| H | 3.37748200 | 1.66474000 | -1.98938200 |
| H | 1.73461300 | 1.00406200 | -2.05917200 |
| C | 2.73036000 | 4.04249300 | -0.72995400 |
| H | 3.48246600 | 4.20854300 | 1.29551500 |
| H | 1.85623500 | 3.55141300 | 1.17172200 |
| H | 1.77343400 | 3.45834500 | -2.58447400 |
| H | 0.82883900 | 3.11489900 | -1.14370800 |
| H | 2.33519100 | 5.05986100 | -0.74614200 |
| H | 3.73011900 | 4.08072600 | -1.18019200 |

74

v-1-1

| | | | |
|---|---|---|---|
| Au | 0.56228300 | -0.73527200 | -0.20301200 |
| N | 2.40862500 | 0.03488600 | -0.18986000 |
| C | 4.68877000 | 0.27635500 | -0.01470900 |
| N | -2.39564000 | -0.77213300 | -0.24258300 |

| | | | |
|---|---|---|---|
| C | -3.63170400 | -1.61567300 | -0.34477200 |
| C | 5.14172600 | -2.46131300 | 0.03780300 |
| H | 5.33835500 | -3.52666000 | 0.05951100 |
| C | -1.28134500 | -1.45983900 | -0.18904800 |
| C | 2.70221800 | 1.38209400 | -0.17388700 |
| C | 3.60983200 | -0.63521100 | -0.09271300 |
| C | 4.60924900 | 2.88751900 | -0.02846200 |
| H | 5.67779500 | 3.05300400 | 0.05482700 |
| C | 1.82628500 | 2.46866200 | -0.25026400 |
| H | 0.75552400 | 2.30757300 | -0.35080300 |
| C | 4.09798600 | 1.59096300 | -0.06544500 |
| C | 3.83703800 | -2.01277300 | -0.06584900 |
| H | 3.00380700 | -2.70623700 | -0.12753200 |
| C | -3.09683300 | -2.96586400 | 0.14453600 |
| H | -3.58929600 | -3.79849800 | -0.36093400 |
| H | -3.29556000 | -3.05658500 | 1.21716800 |
| C | 5.99635600 | -0.19762800 | 0.08912800 |
| H | 6.82825300 | 0.49555200 | 0.14891800 |
| C | 2.35512800 | 3.74731800 | -0.21078900 |
| H | 1.68987800 | 4.60083500 | -0.27046700 |
| C | 6.21792100 | -1.56408900 | 0.11483000 |
| H | 7.22735600 | -1.94762000 | 0.19490700 |
| C | -4.10646800 | -1.70019200 | -1.79695400 |
| H | -4.56500300 | -0.77185200 | -2.13229000 |
| H | -4.85667200 | -2.48950500 | -1.87167300 |
| H | -3.28393100 | -1.94095800 | -2.47016300 |
| C | 3.73678900 | 3.96106200 | -0.09918200 |
| H | 4.11984200 | 4.97342600 | -0.07148400 |
| C | -4.77326500 | -1.13664700 | 0.53812500 |
| H | -4.44722600 | -1.00083200 | 1.57094800 |
| H | -5.55038100 | -1.90368200 | 0.52507100 |
| H | -5.21644100 | -0.20905600 | 0.17436700 |
| C | -1.58136800 | -2.93390000 | -0.08571700 |
| C | -1.12592800 | -3.64617600 | -1.36118800 |

| | | | |
|---|---|---|---|
| H | -0.05449700 | -3.49470700 | -1.50816700 |
| H | -1.31646000 | -4.71747400 | -1.26529900 |
| H | -1.64348700 | -3.28344900 | -2.24951400 |
| C | -0.81851100 | -3.51679700 | 1.10380700 |
| H | -1.11715200 | -4.55705000 | 1.25584100 |
| H | 0.25843000 | -3.48181700 | 0.92608200 |
| H | -1.03068400 | -2.95606800 | 2.01774700 |
| C | -2.37568600 | 0.70423000 | -0.22659900 |
| C | -3.11463700 | 1.33164200 | -1.40123300 |
| C | -2.83012200 | 1.33062700 | 1.10342000 |
| H | -1.31277100 | 0.94714900 | -0.34370800 |
| C | -2.83127900 | 2.82917600 | -1.47369300 |
| H | -4.19850900 | 1.19753800 | -1.28640900 |
| H | -2.81470000 | 0.84495600 | -2.33406700 |
| C | -2.52061300 | 2.82740600 | 1.01675400 |
| H | -3.92157400 | 1.23871400 | 1.16894400 |
| C | -3.23437200 | 3.48123700 | -0.15708200 |
| H | -1.74754900 | 2.96047600 | -1.60096800 |
| H | -2.79493200 | 3.31362600 | 1.95659500 |
| H | -1.43904500 | 2.96320200 | 0.89525800 |
| H | -3.01860100 | 4.55233500 | -0.19014400 |
| H | -4.32144600 | 3.37780100 | -0.03599900 |
| C | -3.55392000 | 3.45834400 | -2.65381100 |
| H | -4.63527400 | 3.33426800 | -2.54184300 |
| H | -3.34547800 | 4.52751800 | -2.71828400 |
| H | -3.25719600 | 2.99642100 | -3.59726100 |
| C | -2.22547100 | 0.67371000 | 2.35723300 |
| H | -2.20815200 | -0.41224600 | 2.19343800 |
| C | -3.11034200 | 0.94084000 | 3.57068000 |
| H | -2.69227600 | 0.47701700 | 4.46579300 |
| H | -3.18761500 | 2.01422400 | 3.76362900 |
| H | -4.12222800 | 0.55282600 | 3.42524100 |
| C | -0.79730100 | 1.12777700 | 2.64601900 |
| H | -0.34685300 | 0.49804400 | 3.41475800 |

H -0.15214400 1.08052600 1.76442000
H -0.78812500 2.15733500 3.01294200
60
w-1-1
Au -0.10897100 -0.57446600 0.15996600
N -2.06144500 -0.14015700 0.16386400
C -4.33767000 -0.27554800 -0.13560900
N 2.78653500 -0.07219200 0.00965600
C 4.17207600 -0.62791000 -0.06211400
C -4.28114600 -3.00841300 -0.61419300
H -4.27881000 -4.07539600 -0.80300600
C 1.83452700 -0.96387000 0.11874400
C -2.59655400 1.12118100 0.32503400
C -3.11483800 -0.98634100 -0.11354200
C -4.73922000 2.27435300 0.26385900
H -5.81505000 2.26152800 0.12761800
C -1.94021100 2.31618800 0.63266900
H -0.86686000 2.33010800 0.80143900
C -4.00034800 1.09760300 0.14723300
C -3.08589400 -2.36134500 -0.35566000
H -2.14386900 -2.90069100 -0.33779100
C 3.88484600 -2.12768800 -0.24063900
H 4.58427000 -2.73693000 0.33483400
H 4.00025400 -2.39303200 -1.29513200
C -5.53105900 -0.94750300 -0.39898500
H -6.47190700 -0.40841200 -0.41656100
C -2.69283500 3.47284600 0.74216900
H -2.19932900 4.40701000 0.98383700
C -5.49862700 -2.31107700 -0.63666300
H -6.41674600 -2.84716400 -0.84191800
C 4.94393800 -0.36154600 1.22908100
H 5.18949100 0.69090700 1.35620100
H 5.88072300 -0.92044800 1.19514600
H 4.37854500 -0.69497800 2.09975100

| | | | |
|---|---|---|---|
| C | -4.08313400 | 3.45845900 | 0.55653900 |
| H | -4.64410300 | 4.37980800 | 0.65077400 |
| C | 4.93443600 | -0.08514800 | -1.26364600 |
| H | 4.36195700 | -0.23149300 | -2.18108400 |
| H | 5.87200800 | -0.63714900 | -1.35374000 |
| H | 5.17797500 | 0.97269700 | -1.16410400 |
| C | 2.42654100 | -2.34705400 | 0.18208700 |
| C | 2.28441400 | -2.85334200 | 1.62116300 |
| H | 1.23001900 | -2.89817300 | 1.90007700 |
| H | 2.70988000 | -3.85661800 | 1.69591200 |
| H | 2.79648500 | -2.20627700 | 2.33505700 |
| C | 1.68710400 | -3.29574500 | -0.75641800 |
| H | 2.17230900 | -4.27475900 | -0.74822400 |
| H | 0.64953900 | -3.41769000 | -0.43765500 |
| H | 1.68579200 | -2.91692600 | -1.78046300 |
| C | 2.42697200 | 1.35406400 | -0.12478700 |
| C | 3.32480900 | 2.35927700 | 0.57569500 |
| C | 2.21495600 | 1.75966900 | -1.58043000 |
| H | 1.44949100 | 1.42452100 | 0.36366200 |
| C | 2.76040500 | 3.73699600 | 0.34857900 |
| H | 4.35336900 | 2.31851700 | 0.20370100 |
| H | 3.36680100 | 2.14989800 | 1.64803500 |
| C | 1.36395400 | 3.02303700 | -1.59572700 |
| H | 3.17933000 | 1.95157800 | -2.06083500 |
| H | 1.71498600 | 0.94942400 | -2.11678400 |
| C | 1.86922600 | 4.02249400 | -0.59762700 |
| H | 3.09895200 | 4.51847700 | 1.01965700 |
| H | 1.35623900 | 3.46758800 | -2.59348600 |
| H | 0.32042600 | 2.76856900 | -1.37041600 |
| H | 1.48088300 | 5.03297600 | -0.66733000 |

68

e-1-0

| | | | |
|---|---|---|---|
| Au | -0.24879400 | -0.89696700 | -0.11303200 |
| N | -2.10862000 | -0.16966700 | -0.15113700 |

| | | | |
|---|---|---|---|
| C | -4.38442700 | 0.14911000 | -0.09375200 |
| C | 2.09631000 | 1.46247200 | 1.36839400 |
| N | 2.60395400 | -0.54410200 | 0.06212800 |
| C | 3.95762800 | -1.10502500 | 0.16950800 |
| C | 1.94754600 | 2.84734700 | 1.40355600 |
| H | 1.70674700 | 3.32797600 | 2.34457900 |
| C | -4.93399500 | -2.57117600 | -0.14836300 |
| H | -5.16838300 | -3.62886200 | -0.16982400 |
| C | 1.64480400 | -1.42782500 | -0.04025900 |
| C | -2.35819600 | 1.18803400 | -0.11376500 |
| C | 0.49247200 | 0.90063500 | 3.20146000 |
| H | -0.27939900 | 0.70701600 | 2.45273700 |
| H | 0.38487700 | 1.93538600 | 3.53491200 |
| H | 0.31487900 | 0.25235100 | 4.06139000 |
| C | 2.51999100 | 1.61921500 | -1.04365800 |
| C | 2.07751500 | 3.61084200 | 0.25416100 |
| H | 1.94754600 | 4.68471400 | 0.30116800 |
| C | -3.33505500 | -0.79957800 | -0.13985100 |
| C | 2.40042000 | 0.87825600 | 0.13567900 |
| C | 1.45142400 | 1.06387200 | -3.21380200 |
| H | 0.60738700 | 0.61806200 | -2.68157200 |
| H | 1.56725900 | 0.55903800 | -4.17476900 |
| H | 1.20815500 | 2.11194900 | -3.40610200 |
| C | -4.21019200 | 2.75916200 | -0.02849400 |
| H | -5.27430000 | 2.96618900 | 0.00202100 |
| C | -1.43474600 | 2.23683100 | -0.11496700 |
| H | -0.36782600 | 2.03373100 | -0.15792200 |
| C | 1.89087700 | 0.65761600 | 2.63296700 |
| H | 1.96498900 | -0.40463800 | 2.38394600 |
| C | 2.73726400 | 0.96523500 | -2.39110300 |
| H | 2.95368500 | -0.09600100 | -2.23396200 |
| C | -3.74783000 | 1.44396000 | -0.07451100 |
| C | -3.61059800 | -2.16777100 | -0.16677500 |
| H | -2.79977700 | -2.88790900 | -0.20256600 |

| | | | |
|---|---|---|---|
| C | 3.74182300 | -2.53020300 | -0.31941100 |
| H | 4.41556700 | -3.24951700 | 0.14612000 |
| H | 3.88782900 | -2.57147400 | -1.40215800 |
| C | -5.71116900 | -0.28016400 | -0.07552600 |
| H | -6.52064300 | 0.44069100 | -0.04045600 |
| C | 2.96428800 | 0.98510300 | 3.67006100 |
| H | 3.96850000 | 0.79603500 | 3.28347900 |
| H | 2.82069100 | 0.38624400 | 4.57132100 |
| H | 2.90962700 | 2.03839400 | 3.95578900 |
| C | -1.91618100 | 3.53354000 | -0.06581300 |
| H | -1.21302500 | 4.35946900 | -0.06531800 |
| C | 2.34893300 | 2.99841200 | -0.96032700 |
| H | 2.41423200 | 3.59595000 | -1.86212500 |
| C | -5.98089900 | -1.63802700 | -0.10307000 |
| H | -7.00596100 | -1.98672300 | -0.08993400 |
| C | 3.91571500 | 1.57844500 | -3.14367000 |
| H | 4.83506000 | 1.53238000 | -2.55605900 |
| H | 3.72320500 | 2.62604200 | -3.38439200 |
| H | 4.07928000 | 1.05014300 | -4.08452100 |
| C | -3.29354800 | 3.79820400 | -0.02185600 |
| H | -3.63958700 | 4.82368600 | 0.01437600 |
| C | 2.26367100 | -2.80527700 | 0.00788600 |
| C | 2.09871100 | -3.30111200 | 1.44921400 |
| C | 1.59510100 | -3.76540600 | -0.96237700 |
| H | 1.04448400 | -3.30806500 | 1.73236700 |
| H | 2.63887500 | -2.66468200 | 2.15581000 |
| H | 0.54547800 | -3.90365300 | -0.69563900 |
| H | 1.63688800 | -3.38298300 | -1.98421300 |
| H | 2.49155300 | -4.31673300 | 1.53197600 |
| H | 2.09407600 | -4.73677200 | -0.93296400 |
| H | 4.64716400 | -0.51655200 | -0.43819300 |
| H | 4.28231400 | -1.04242300 | 1.21410300 |

80

e-1-2

| | | | |
|---|---|---|---|
| Au | -0.64709800 | -0.88168500 | -0.06108500 |
| N | -2.54884900 | -0.26337800 | 0.00846100 |
| C | -4.83970100 | -0.08047900 | 0.07391100 |
| C | 1.71278700 | 1.63032800 | 1.24559200 |
| N | 2.20028200 | -0.38870000 | -0.08019700 |
| C | 3.62089500 | -0.88842900 | -0.10837700 |
| C | 1.52499700 | 3.01177000 | 1.27044300 |
| H | 1.41162400 | 3.50845700 | 2.22683900 |
| C | -5.22661300 | -2.82743100 | -0.03023600 |
| H | -5.39782100 | -3.89663100 | -0.07051700 |
| C | 1.27478800 | -1.31248800 | -0.12937400 |
| C | -2.87798200 | 1.07578100 | 0.07081900 |
| C | 0.15208400 | 0.98323100 | 3.06478500 |
| H | -0.57213500 | 0.62944600 | 2.32825800 |
| H | -0.08636100 | 2.02523500 | 3.29237700 |
| H | 0.03061900 | 0.39961700 | 3.97947900 |
| C | 1.66548800 | 1.71975500 | -1.20342400 |
| C | 1.43777900 | 3.74782100 | 0.10064500 |
| H | 1.29462700 | 4.82017100 | 0.14204900 |
| C | -3.73584400 | -0.96425900 | 0.00927600 |
| C | 1.86209700 | 1.01507000 | -0.00595500 |
| C | 0.12884900 | 1.18391300 | -3.08428300 |
| H | -0.58889500 | 0.74202400 | -2.38982200 |
| H | 0.03135500 | 0.68569000 | -4.05091200 |
| H | -0.13886400 | 2.23531200 | -3.21560000 |
| C | -4.81905700 | 2.53416000 | 0.17966800 |
| H | -5.89346500 | 2.67795200 | 0.21333100 |
| C | -2.01803100 | 2.17733400 | 0.09084900 |
| H | -0.94097800 | 2.03765200 | 0.05547000 |
| C | 1.59078000 | 0.86636000 | 2.54989800 |
| H | 1.78587800 | -0.19076100 | 2.35505000 |
| C | 1.56489400 | 1.06330500 | -2.56627100 |
| H | 1.79306300 | -0.00187300 | -2.46398000 |
| C | -4.28055200 | 1.24912100 | 0.11406500 |

| | | | |
|---|---|---|---|
| C | -3.92963700 | -2.34569000 | -0.04304700 |
| H | -3.07723300 | -3.01527000 | -0.09253500 |
| C | 3.38497900 | -2.34441400 | -0.53383700 |
| H | 4.07169700 | -3.02979200 | -0.03520200 |
| H | 3.55873000 | -2.42870500 | -1.61132800 |
| C | -6.13858900 | -0.58839600 | 0.08599500 |
| H | -6.98950300 | 0.08215600 | 0.13547300 |
| C | 2.55266000 | 1.36349800 | 3.62800000 |
| H | 3.59288000 | 1.35994500 | 3.30021700 |
| H | 2.47017100 | 0.73890000 | 4.51935300 |
| H | 2.30060000 | 2.38454800 | 3.92307400 |
| C | -2.57459700 | 3.44291900 | 0.15630200 |
| H | -1.92064100 | 4.30826700 | 0.17272700 |
| C | 1.46915800 | 3.09621800 | -1.12202400 |
| H | 1.31854900 | 3.66074300 | -2.03459000 |
| C | -6.32708800 | -1.95916500 | 0.03393200 |
| H | -7.32957400 | -2.36842900 | 0.04245700 |
| C | 2.53007300 | 1.68219700 | -3.57819500 |
| H | 3.55570200 | 1.71716100 | -3.20600400 |
| H | 2.22973000 | 2.70507400 | -3.81554100 |
| H | 2.51606000 | 1.11421900 | -4.51016900 |
| C | 4.23321300 | -0.75591800 | 1.28795000 |
| H | 4.20147900 | 0.30049500 | 1.56829000 |
| H | 3.60385900 | -1.28788500 | 2.00485200 |
| C | -3.96522200 | 3.62537400 | 0.20095200 |
| H | -4.37118500 | 4.62801800 | 0.25178200 |
| C | 4.48275800 | -0.16435200 | -1.15067800 |
| H | 3.92231300 | -0.14118400 | -2.08820300 |
| H | 5.33394000 | -0.82758600 | -1.33858300 |
| C | 1.91432700 | -2.66901300 | -0.23341200 |
| C | 1.70393000 | -3.39498800 | 1.09864500 |
| C | 1.26084900 | -3.47194400 | -1.35425100 |
| H | 0.63651700 | -3.51670300 | 1.29245100 |
| H | 2.13828500 | -2.84799300 | 1.93751000 |

| | | | |
|---|---|---|---|
| H | 0.20952400 | -3.65863500 | -1.12696800 |
| H | 1.31309000 | -2.93261200 | -2.30317700 |
| H | 2.16743100 | -4.38302900 | 1.05259700 |
| H | 1.77254600 | -4.43036900 | -1.47064800 |
| C | 5.66117300 | -1.27640100 | 1.39272600 |
| H | 6.34760700 | -0.73336700 | 0.74267100 |
| H | 6.02361000 | -1.17095800 | 2.41525800 |
| H | 5.72517300 | -2.33399600 | 1.13158300 |
| C | 5.01013700 | 1.22464700 | -0.80403900 |
| H | 4.21528800 | 1.93397400 | -0.57192200 |
| H | 5.68967400 | 1.19793100 | 0.04882600 |
| H | 5.57104300 | 1.62384600 | -1.65068100 |

89

e-1-3

| | | | |
|---|---|---|---|
| Au | 1.17895200 | -0.82071300 | -0.08414700 |
| N | 3.10714000 | -0.28923500 | -0.05218100 |
| C | 5.40338600 | -0.20855300 | 0.01876100 |
| C | -1.15801300 | 1.79447500 | -1.14900500 |
| N | -1.70457200 | -0.32706500 | 0.00878000 |
| C | -0.85299400 | 3.15092500 | -1.05715900 |
| H | -0.72966000 | 3.72308600 | -1.96868700 |
| C | 5.66467000 | -2.97266500 | 0.00572200 |
| H | 5.78712400 | -4.04929900 | 0.00140000 |
| C | -0.75007100 | -1.22719200 | -0.05926100 |
| C | 3.49657100 | 1.03381600 | -0.02830700 |
| C | 0.24791500 | 1.00249900 | -3.04240700 |
| H | 0.85731900 | 0.37925900 | -2.38632200 |
| H | 0.72570500 | 1.98409400 | -3.10467000 |
| H | 0.24508500 | 0.56002500 | -4.04082900 |
| C | -1.02867800 | 1.65244300 | 1.29283100 |
| C | -0.67414600 | 3.76917800 | 0.16982500 |
| H | -0.44343600 | 4.82585500 | 0.21886200 |
| C | 4.26034000 | -1.04320700 | -0.02219900 |
| C | -1.31990700 | 1.07523800 | 0.04736400 |

| | | | |
|---|---|---|---|
| C | 0.50513800 | 0.90550400 | 3.10763700 |
| H | 1.21074400 | 0.53428000 | 2.36148400 |
| H | 0.59677300 | 0.29529100 | 4.00837500 |
| H | 0.79617100 | 1.92756500 | 3.36171000 |
| C | 5.49812400 | 2.40801700 | 0.04638500 |
| H | 6.57759000 | 2.50699600 | 0.07993100 |
| C | 2.68396500 | 2.16994100 | -0.04118600 |
| H | 1.60237000 | 2.07148700 | -0.07548700 |
| C | -1.18716200 | 1.15965500 | -2.52798500 |
| H | -1.63801900 | 0.16645000 | -2.44455200 |
| C | -0.93690100 | 0.87435400 | 2.59149900 |
| H | -1.19794400 | -0.17027400 | 2.39758300 |
| C | 4.90503500 | 1.14581200 | 0.01561600 |
| C | 4.39105600 | -2.43304700 | -0.02845000 |
| H | 3.50861700 | -3.06412500 | -0.05960600 |
| C | -2.74913200 | -2.35715700 | 0.46532800 |
| H | -3.46372300 | -3.07818000 | 0.07517500 |
| H | -2.73687400 | -2.46619300 | 1.55608600 |
| C | 6.67751200 | -0.77459500 | 0.05252500 |
| H | 7.55815500 | -0.14241000 | 0.08358600 |
| C | -1.96933700 | 1.97340100 | -3.56289900 |
| H | -2.95786900 | 2.27517400 | -3.21811200 |
| H | -2.08790200 | 1.38670600 | -4.47546100 |
| H | -1.41753100 | 2.87578000 | -3.83476400 |
| C | 3.29411300 | 3.41175400 | -0.00880600 |
| H | 2.67878700 | 4.30513000 | -0.01763600 |
| C | -0.73054900 | 3.01367100 | 1.32837900 |
| H | -0.51440400 | 3.47849000 | 2.28301800 |
| C | 6.80336800 | -2.15361100 | 0.04568700 |
| H | 7.78610200 | -2.60763200 | 0.07143600 |
| C | -1.87142800 | 1.42845100 | 3.66786300 |
| H | -2.90659000 | 1.49527300 | 3.33007100 |
| H | -1.55448200 | 2.43045000 | 3.96581100 |
| H | -1.83603500 | 0.79683400 | 4.55761700 |

| | | | |
|---|---|---|---|
| C | 4.69152900 | 3.53492300 | 0.03441700 |
| H | 5.14030000 | 4.52018600 | 0.05866500 |
| C | -1.33758200 | -2.60572500 | -0.05595100 |
| C | -1.29148500 | -3.13391100 | -1.49355500 |
| C | -0.54780800 | -3.54207100 | 0.85133300 |
| H | -0.25572700 | -3.19779000 | -1.83297700 |
| H | -1.83487500 | -2.48924100 | -2.18619900 |
| H | 0.46288900 | -3.68911800 | 0.46499800 |
| H | -0.46806200 | -3.13265400 | 1.86105400 |
| H | -1.73326000 | -4.13244500 | -1.52866600 |
| H | -1.04763200 | -4.51242200 | 0.90671400 |
| C | -3.11430600 | -0.89685800 | 0.14194400 |
| C | -3.94203600 | -0.30017900 | 1.29859600 |
| C | -3.96099200 | -0.73092600 | -1.14101700 |
| H | -3.35231100 | -0.37519200 | 2.21672700 |
| C | -5.20877200 | -1.16124100 | 1.44982500 |
| C | -4.40224100 | 1.14354900 | 1.07306000 |
| H | -3.38641900 | -1.07933800 | -2.00337900 |
| C | -5.25087000 | -1.55453600 | -1.02599300 |
| C | -4.38048800 | 0.72760700 | -1.35598600 |
| H | -5.78357000 | -0.77857700 | 2.29932700 |
| H | -4.95523300 | -2.19910700 | 1.67884500 |
| C | -6.06235200 | -1.08630200 | 0.18228600 |
| H | -3.56164000 | 1.83583400 | 1.00387600 |
| H | -5.00040100 | 1.44892400 | 1.93952000 |
| C | -5.24308300 | 1.22336400 | -0.19798800 |
| H | -5.04843500 | -2.62526200 | -0.96829100 |
| H | -5.83584700 | -1.39679100 | -1.93835300 |
| H | -3.50959300 | 1.37483800 | -1.43509500 |
| H | -4.92967100 | 0.79636300 | -2.30122400 |
| H | -6.94382900 | -1.72218500 | 0.29977000 |
| C | -6.49059000 | 0.35974400 | -0.05006700 |
| H | -5.52800400 | 2.26267000 | -0.38073500 |
| H | -7.10690700 | 0.42648500 | -0.95286600 |

| | | | |
|---|---|---|---|
| H | -7.09386600 | 0.71585100 | 0.79171100 |

81

e-1-4

| | | | |
|---|---|---|---|
| Au | 0.76221800 | -0.87459900 | 0.03496500 |
| N | 2.68401000 | -0.31836500 | 0.00674700 |
| C | 4.98024700 | -0.20597200 | 0.00116200 |
| C | -1.49867500 | 1.70694600 | -1.27193200 |
| N | -2.06307200 | -0.27739400 | 0.06984300 |
| C | -1.24506500 | 3.07684800 | -1.31687300 |
| H | -1.09204300 | 3.55022100 | -2.27954600 |
| C | 5.27992200 | -2.96421700 | 0.09177200 |
| H | 5.41718600 | -4.03847200 | 0.12741600 |
| C | -1.17427900 | -1.23662800 | 0.07263200 |
| C | 3.05575100 | 1.01043000 | -0.03594200 |
| C | 0.02901100 | 0.98037700 | -3.09302700 |
| H | 0.74860500 | 0.63000500 | -2.35025200 |
| H | 0.29467300 | 2.00728900 | -3.35591400 |
| H | 0.12773000 | 0.36530100 | -3.98963800 |
| C | -1.49372000 | 1.83974600 | 1.17572500 |
| C | -1.14540500 | 3.82990900 | -0.15893100 |
| H | -0.94897400 | 4.89307100 | -0.21695500 |
| C | 3.84844100 | -1.05571800 | 0.02998600 |
| C | -1.69220400 | 1.11911800 | -0.01166300 |
| C | 0.03390300 | 1.27420200 | 3.05072800 |
| H | 0.73305600 | 0.81051800 | 2.35137700 |
| H | 0.12471700 | 0.77415700 | 4.01702900 |
| H | 0.33357800 | 2.31738800 | 3.17818000 |
| C | 5.04291100 | 2.40882800 | -0.08213000 |
| H | 6.12175000 | 2.51951700 | -0.08667000 |
| C | 2.23087800 | 2.13816600 | -0.06999800 |
| H | 1.14942800 | 2.03197700 | -0.06453800 |
| C | -1.40855700 | 0.91994200 | -2.56562400 |
| H | -1.63883100 | -0.12721800 | -2.35531100 |
| C | -1.40944300 | 1.19255100 | 2.54404500 |

| | | | |
|---|---|---|---|
| H | -1.66543600 | 0.13430000 | 2.44801200 |
| C | 4.46360000 | 1.14058700 | -0.04169600 |
| C | 3.99839500 | -2.44284200 | 0.07570700 |
| H | 3.12480800 | -3.08605700 | 0.09829400 |
| C | -3.33795400 | -2.22185600 | 0.37405400 |
| H | -4.02571000 | -2.81555300 | -0.23076800 |
| H | -3.55997900 | -2.43175700 | 1.42469000 |
| C | 6.26280300 | -0.75354500 | 0.01797700 |
| H | 7.13486000 | -0.10918800 | -0.00387500 |
| C | -2.36238200 | 1.43125500 | -3.64433900 |
| H | -3.40328600 | 1.43391200 | -3.31928000 |
| H | -2.28284300 | 0.80876500 | -4.53759800 |
| H | -2.09988300 | 2.45108300 | -3.93439700 |
| C | 2.82756700 | 3.38634300 | -0.10984100 |
| H | 2.20103000 | 4.27149800 | -0.13656700 |
| C | -1.23694400 | 3.20529200 | 1.07407700 |
| H | -1.07783000 | 3.77857400 | 1.97977800 |
| C | 6.40780600 | -2.12985700 | 0.06308700 |
| H | 7.39713400 | -2.56985300 | 0.07675300 |
| C | -2.34913300 | 1.84022800 | 3.55994000 |
| H | -3.38377700 | 1.86019800 | 3.21457500 |
| H | -2.04418200 | 2.86943900 | 3.76156100 |
| H | -2.31013100 | 1.29818700 | 4.50669400 |
| C | 4.22386400 | 3.52605600 | -0.11608000 |
| H | 4.66166500 | 4.51602700 | -0.14754300 |
| C | -1.87000500 | -2.57008800 | 0.07559800 |
| C | -1.68210200 | -3.17878900 | -1.31815300 |
| C | -1.26045400 | -3.49438600 | 1.12335100 |
| H | -0.62006600 | -3.30916900 | -1.53426200 |
| H | -2.11258300 | -2.54388500 | -2.09606900 |
| H | -0.21234800 | -3.69322500 | 0.89086400 |
| H | -1.30884100 | -3.04513300 | 2.11791100 |
| H | -2.17278600 | -4.15396800 | -1.35772900 |
| H | -1.80282100 | -4.44259900 | 1.14348200 |

| | | | |
|---|---|---|---|
| C | -3.49745000 | -0.71739100 | 0.11058100 |
| C | -4.24729200 | -0.01203900 | 1.23726500 |
| C | -4.17800600 | -0.38907800 | -1.21884400 |
| C | -5.71848300 | -0.41162500 | 1.25196200 |
| H | -4.16783400 | 1.07224300 | 1.09344700 |
| H | -3.77610000 | -0.25599200 | 2.19222300 |
| C | -5.66157600 | -0.74218400 | -1.21456000 |
| H | -4.06847300 | 0.68847900 | -1.38705500 |
| H | -3.66664000 | -0.90159900 | -2.03771900 |
| C | -6.38384800 | -0.05007900 | -0.06795100 |
| H | -6.22071300 | 0.07552300 | 2.08965900 |
| H | -5.80349100 | -1.49258900 | 1.41763000 |
| H | -6.10009100 | -0.46632300 | -2.17552700 |
| H | -5.78598700 | -1.82632300 | -1.11504500 |
| H | -7.44046600 | -0.32397200 | -0.05780800 |
| H | -6.33258700 | 1.03569200 | -0.21026000 |

82

e-1-7

| | | | |
|---|---|---|---|
| Au | -0.83150500 | -0.84837600 | -0.05632300 |
| N | -2.75014600 | -0.28374200 | -0.04376000 |
| C | -5.04606100 | -0.16432200 | -0.03116100 |
| C | 1.40879600 | 1.58742000 | 1.32048900 |
| N | 2.03131600 | -0.33883800 | -0.07563100 |
| C | 1.14104800 | 2.95180200 | 1.40649500 |
| H | 0.94743000 | 3.38923400 | 2.37898300 |
| C | -5.35425300 | -2.92206300 | -0.11343200 |
| H | -5.49492000 | -3.99597300 | -0.14585100 |
| C | 1.09419600 | -1.25842400 | -0.08817600 |
| C | -3.11793700 | 1.04576300 | -0.00375400 |
| C | -0.12796300 | 0.81120200 | 3.11011100 |
| H | -0.85927400 | 0.56644400 | 2.33723200 |
| H | -0.35065000 | 1.81762300 | 3.47342400 |
| H | -0.25536200 | 0.11714000 | 3.94289600 |
| C | 1.51418800 | 1.81559800 | -1.11897300 |

| | | | |
|---|---|---|---|
| C | 1.08397800 | 3.74752300 | 0.27258100 |
| H | 0.87397700 | 4.80582800 | 0.36312300 |
| C | -3.91689100 | -1.01770900 | -0.06171900 |
| C | 1.66381700 | 1.05570100 | 0.04715100 |
| C | 0.12458400 | 1.25255400 | -3.09401800 |
| H | -0.59308900 | 0.74368000 | -2.44629300 |
| H | 0.10487700 | 0.77955900 | -4.07782200 |
| H | -0.20606800 | 2.28818200 | -3.20717000 |
| C | -5.09844600 | 2.45182000 | 0.04721000 |
| H | -6.17670600 | 2.56773400 | 0.05563100 |
| C | -2.28759800 | 2.16892700 | 0.02348100 |
| H | -1.20714700 | 2.05501200 | 0.01155300 |
| C | 1.30938400 | 0.74615200 | 2.58064800 |
| H | 1.52837200 | -0.29389800 | 2.32447300 |
| C | 1.53893600 | 1.21030600 | -2.50768900 |
| H | 1.83664300 | 0.16103000 | -2.42697200 |
| C | -4.52501800 | 1.18088500 | 0.00698200 |
| C | -4.07110900 | -2.40445800 | -0.10319200 |
| H | -3.19938900 | -3.05035200 | -0.12714200 |
| C | 3.15767600 | -2.32063500 | -0.54079300 |
| H | 3.87084200 | -3.01641900 | -0.10022300 |
| H | 3.23073100 | -2.41166100 | -1.63038700 |
| C | -6.33023200 | -0.70816800 | -0.04188400 |
| H | -7.20031800 | -0.06122000 | -0.01845400 |
| C | 2.26417400 | 1.18348700 | 3.69223300 |
| H | 3.31359700 | 1.04611900 | 3.43161700 |
| H | 2.06245300 | 0.60339500 | 4.59481800 |
| H | 2.11114800 | 2.23657600 | 3.93947200 |
| C | -2.87833300 | 3.41988300 | 0.06414000 |
| H | -2.24830600 | 4.30271100 | 0.08605400 |
| C | 1.23597200 | 3.17374800 | -0.97964400 |
| H | 1.11548000 | 3.78179300 | -1.86850400 |
| C | -6.47945400 | -2.08420300 | -0.08288400 |
| H | -7.47014900 | -2.52123400 | -0.09180600 |

| | | | |
|---|---|---|---|
| C | 2.51151700 | 1.92273100 | -3.44620800 |
| H | 3.52633700 | 1.95464200 | -3.04599400 |
| H | 2.18997600 | 2.95088500 | -3.62608300 |
| H | 2.53775700 | 1.41617600 | -4.41283500 |
| C | -4.27404300 | 3.56541900 | 0.07619800 |
| H | -4.70742700 | 4.55733300 | 0.10767400 |
| C | 1.72541500 | -2.62020700 | -0.10651400 |
| C | 1.66192500 | -3.15680000 | 1.32820800 |
| C | 0.99632900 | -3.56873400 | -1.04713700 |
| H | 0.62813900 | -3.18896700 | 1.67834000 |
| H | 2.23932000 | -2.53239100 | 2.01408800 |
| H | -0.02642000 | -3.73469200 | -0.70210100 |
| H | 0.94992400 | -3.15984500 | -2.05859600 |
| H | 2.07331000 | -4.16845200 | 1.35400300 |
| H | 1.51450800 | -4.53000700 | -1.08277400 |
| C | 3.43234300 | -0.85877400 | -0.15964900 |
| C | 4.33344600 | -0.60741400 | 1.07520100 |
| C | 4.41408800 | -0.17513000 | -1.13615600 |
| C | 5.62420500 | -1.31310200 | 0.62884900 |
| C | 4.64405000 | 0.89598300 | 1.01177700 |
| H | 3.93461500 | -0.95008400 | 2.03136600 |
| C | 5.64566600 | -1.06620000 | -0.90591600 |
| C | 4.74541600 | 1.18135700 | -0.50544300 |
| H | 4.08209300 | -0.14424200 | -2.17417100 |
| H | 6.48785800 | -0.87638000 | 1.13120100 |
| H | 5.62078600 | -2.37759800 | 0.86363800 |
| H | 3.86397500 | 1.50720200 | 1.45629500 |
| H | 5.57029700 | 1.11351500 | 1.54623300 |
| H | 6.55426500 | -0.56452500 | -1.23905100 |
| H | 5.56890800 | -2.00477300 | -1.45655100 |
| H | 4.05054600 | 1.96692400 | -0.79790400 |
| H | 5.74489800 | 1.50222800 | -0.80461400 |

89

e-1-8

| | | | |
|---|---|---|---|
| Au | -1.40559800 | -0.88686500 | 0.00057900 |
| N | -3.27438300 | -0.17076300 | 0.00068800 |
| C | -5.55277600 | 0.13422100 | 0.00010700 |
| C | 1.05823300 | 1.57647800 | 1.22585100 |
| N | 1.45545800 | -0.51516400 | 0.00020900 |
| C | 2.87044800 | -1.05301000 | 0.00026800 |
| C | 0.96231200 | 2.96679800 | 1.19669800 |
| H | 0.85915300 | 3.50451800 | 2.13154300 |
| C | -6.08384900 | -2.59037700 | 0.00071300 |
| H | -6.31102500 | -3.64988300 | 0.00095800 |
| C | 0.49776700 | -1.40283400 | 0.00050100 |
| C | -3.53262000 | 1.18570300 | 0.00017100 |
| C | -0.59329200 | 1.04994700 | 3.00588800 |
| H | -1.30166400 | 0.65361900 | 2.27592000 |
| H | -0.81926000 | 2.11029000 | 3.14614200 |
| H | -0.75385200 | 0.53954000 | 3.95781100 |
| C | 1.05715800 | 1.57555600 | -1.22682100 |
| C | 0.95500300 | 3.66304500 | -0.00118000 |
| H | 0.88610900 | 4.74366400 | -0.00159200 |
| C | -4.49648700 | -0.80807600 | 0.00055400 |
| C | 1.19713800 | 0.90798600 | -0.00027800 |
| C | -0.59526200 | 1.04794000 | -3.00590300 |
| H | -1.30351700 | 0.65211700 | -2.27555500 |
| H | -0.75611100 | 0.53698000 | -3.95748200 |
| H | -0.82118700 | 2.10821300 | -3.14676100 |
| C | -5.39738400 | 2.74550100 | -0.00077600 |
| H | -6.46342800 | 2.94480800 | -0.00106500 |
| C | -2.61713700 | 2.24228200 | -0.00001600 |
| H | -1.54753200 | 2.04939900 | 0.00032600 |
| C | 0.86033100 | 0.87562500 | 2.55458900 |
| H | 1.05074600 | -0.19287400 | 2.42212500 |
| C | 0.85849300 | 0.87382700 | -2.55499300 |
| H | 1.04895700 | -0.19459600 | -2.42193900 |
| C | -4.92481100 | 1.43318300 | -0.00017600 |

| | | | |
|---|---|---|---|
| C | -4.76294900 | -2.17843300 | 0.00085500 |
| H | -3.94676700 | -2.89343700 | 0.00120000 |
| C | 2.59831900 | -2.57882100 | 0.00105000 |
| H | 3.05641700 | -3.05071200 | 0.86548500 |
| H | 3.05662800 | -3.05157200 | -0.86282200 |
| C | -6.87685600 | -0.30366400 | -0.00003200 |
| H | -7.69146200 | 0.41229000 | -0.00039300 |
| C | 1.79384600 | 1.41216300 | 3.63600200 |
| H | 2.82897900 | 1.44258000 | 3.29839600 |
| H | 1.73803300 | 0.78548500 | 4.52823300 |
| H | 1.49871500 | 2.42280400 | 3.92856800 |
| C | -3.10889000 | 3.53607200 | -0.00062600 |
| H | -2.41108300 | 4.36657000 | -0.00079500 |
| C | 0.96117300 | 2.96588000 | -1.19854800 |
| H | 0.85712000 | 3.50294500 | -2.13367200 |
| C | -7.13742800 | -1.66360900 | 0.00027400 |
| H | -8.16033900 | -2.01886500 | 0.00018800 |
| C | 1.79159200 | 1.40967500 | -3.63714000 |
| H | 2.82681000 | 1.44052000 | -3.29985600 |
| H | 1.49620900 | 2.42005700 | -3.93035200 |
| H | 1.73564000 | 0.78232900 | -4.52889400 |
| C | -4.48869400 | 3.79147900 | -0.00101000 |
| H | -4.84263400 | 4.81491900 | -0.00147300 |
| C | 1.07032200 | -2.78857200 | 0.00118300 |
| C | 0.57283100 | -3.52045100 | 1.24907100 |
| C | 0.57242600 | -3.52241200 | -1.24529400 |
| H | -0.51275600 | -3.62757600 | 1.21533700 |
| H | 0.82384300 | -2.97297200 | 2.16090500 |
| H | -0.51312800 | -3.62977900 | -1.21083100 |
| H | 0.82268200 | -2.97632100 | -2.15815900 |
| H | 1.02607000 | -4.51310900 | 1.30020300 |
| H | 1.02583100 | -4.51505000 | -1.29521800 |
| C | 3.61592700 | -0.59677900 | -1.26200600 |
| C | 3.50487500 | -1.30345900 | -2.46250500 |

| | | | |
|---|---|---|---|
| C | 4.45501800 | 0.51910000 | -1.23566500 |
| C | 4.25714400 | -0.96999200 | -3.57762700 |
| H | 2.81906000 | -2.13713800 | -2.54198300 |
| C | 5.22465600 | 0.84162500 | -2.35306800 |
| C | 5.14726700 | 0.09480100 | -3.51617100 |
| H | 4.15300200 | -1.54722300 | -4.48725600 |
| H | 5.89493400 | 1.69180900 | -2.29444100 |
| H | 5.75988500 | 0.34969700 | -4.37134500 |
| C | 3.61665100 | -0.59565400 | 1.26165200 |
| C | 3.50665400 | -1.30131200 | 2.46286600 |
| C | 4.45575300 | 0.52014700 | 1.23361000 |
| C | 4.25982600 | -0.96684100 | 3.57706300 |
| H | 2.82098100 | -2.13497700 | 2.54370400 |
| C | 5.22637200 | 0.84364600 | 2.35009000 |
| C | 5.14989800 | 0.09790200 | 3.51394600 |
| H | 4.15642100 | -1.54326000 | 4.48729200 |
| H | 5.89666800 | 1.69371600 | 2.29010200 |
| H | 5.76324000 | 0.35352700 | 4.36838300 |
| C | 4.54405700 | 1.36110400 | -0.00141500 |
| H | 5.47048100 | 1.93811600 | -0.00182000 |
| H | 3.71952000 | 2.08834500 | -0.00167900 |

88

e-1-15

| | | | |
|---|---|---|---|
| Au | -1.31104100 | -0.79069900 | -0.31683000 |
| N | -3.23606900 | -0.27076400 | -0.15330800 |
| C | -5.53240300 | -0.17976300 | -0.09921600 |
| C | 0.80268400 | 1.18262600 | 1.77472100 |
| N | 1.54928600 | -0.27466500 | -0.11471400 |
| C | 2.96426200 | -0.68993800 | -0.53095100 |
| C | 0.55404400 | 2.48156600 | 2.22089700 |
| H | 0.28327000 | 2.63012300 | 3.25947900 |
| C | -5.82075700 | -2.80637900 | -0.95069600 |
| H | -5.95366900 | -3.82935100 | -1.28229700 |
| C | 0.62005300 | -1.12248400 | -0.49860900 |

| | | | |
|---|---|---|---|
| C | -3.61289800 | 0.99232900 | 0.25615900 |
| C | -0.95223500 | -0.00442300 | 3.10603700 |
| H | -1.57657200 | -0.17404800 | 2.22857800 |
| H | -1.27343000 | 0.93388000 | 3.56483100 |
| H | -1.13403500 | -0.81043400 | 3.82005200 |
| C | 1.10230200 | 2.09086200 | -0.48214900 |
| C | 0.60089800 | 3.56845300 | 1.36569300 |
| H | 0.40403800 | 4.56495300 | 1.74048800 |
| C | -4.39738900 | -0.98023600 | -0.37289200 |
| C | 1.16755400 | 1.02030900 | 0.42594900 |
| C | -0.24490500 | 2.02414500 | -2.55972500 |
| H | -0.92313400 | 1.28950500 | -2.11908300 |
| H | -0.22903200 | 1.88118300 | -3.64234300 |
| H | -0.65297700 | 3.01700000 | -2.35218800 |
| C | -5.60421700 | 2.30852500 | 0.70986000 |
| H | -6.68326500 | 2.40646300 | 0.75671400 |
| C | -2.79128900 | 2.07394000 | 0.58452400 |
| H | -1.70972800 | 1.98361600 | 0.52558100 |
| C | 0.54038200 | 0.05713900 | 2.76333300 |
| H | 0.82295800 | -0.89428800 | 2.30271500 |
| C | 1.17123700 | 1.91130100 | -1.98627400 |
| H | 1.54978800 | 0.91069500 | -2.20859800 |
| C | -5.02090600 | 1.10541600 | 0.31249000 |
| C | -4.54140600 | -2.30125800 | -0.80010300 |
| H | -3.66509000 | -2.90738900 | -1.00562200 |
| C | 2.58952100 | -1.68038000 | -1.63528800 |
| H | 3.35689700 | -2.42392500 | -1.83861400 |
| H | 2.40326200 | -1.12386500 | -2.55842600 |
| C | -6.81253500 | -0.71022200 | -0.25677100 |
| H | -7.68723600 | -0.10369400 | -0.04904100 |
| C | 1.31203800 | 0.24041700 | 4.07727100 |
| H | 2.35335400 | 0.53354600 | 3.93362200 |
| H | 1.28846100 | -0.68449800 | 4.65659300 |
| H | 0.83906300 | 1.01554100 | 4.68420800 |

| | | | |
|---|---|---|---|
| C | -3.39194700 | 3.25699900 | 0.97857200 |
| H | -2.76884800 | 4.10629000 | 1.23769300 |
| C | 0.83400700 | 3.36250500 | 0.01588900 |
| H | 0.78501700 | 4.19685700 | -0.67317500 |
| C | -6.95180900 | -2.02065700 | -0.68209300 |
| H | -7.93930900 | -2.44626300 | -0.80949400 |
| C | 2.08608400 | 2.92421600 | -2.67002200 |
| H | 3.06950000 | 2.96597400 | -2.20212500 |
| H | 1.64782900 | 3.92447300 | -2.64082100 |
| H | 2.21375300 | 2.65837800 | -3.72120500 |
| C | -4.78853500 | 3.37797500 | 1.04354500 |
| H | -5.22940800 | 4.31718900 | 1.35370900 |
| C | 1.25801700 | -2.29090100 | -1.19613700 |
| C | 1.37768400 | -3.43824600 | -0.18582200 |
| C | 0.43101700 | -2.74612100 | -2.39153600 |
| H | 0.37879600 | -3.78766200 | 0.08241200 |
| H | 1.89286500 | -3.13985800 | 0.72790400 |
| H | -0.53304100 | -3.13895500 | -2.06318800 |
| H | 0.24147300 | -1.91552800 | -3.07561400 |
| H | 1.92744500 | -4.26752400 | -0.63732300 |
| H | 0.96347300 | -3.53220000 | -2.93302000 |
| C | 3.85591200 | 0.46542900 | -0.98149100 |
| C | 4.49046800 | 0.43627000 | -2.22034600 |
| C | 4.18073600 | 1.49507000 | -0.09411200 |
| C | 5.41674100 | 1.41350100 | -2.57205600 |
| H | 4.28572700 | -0.36144100 | -2.92258800 |
| C | 5.09329100 | 2.47697900 | -0.44738500 |
| H | 3.72516200 | 1.52766200 | 0.88840800 |
| C | 5.71982700 | 2.43822600 | -1.68892900 |
| H | 5.90166900 | 1.36555100 | -3.53876300 |
| H | 5.32326300 | 3.26882600 | 0.25388700 |
| H | 6.44014900 | 3.19909000 | -1.96072300 |
| C | 3.73267600 | -1.37298200 | 0.60372100 |
| C | 4.72088900 | -2.31731400 | 0.32749600 |

| | | | |
|---|---|---|---|
| C | 3.53451400 | -0.99775300 | 1.92418700 |
| C | 5.44021100 | -2.91018900 | 1.35646100 |
| H | 4.95394600 | -2.58945400 | -0.69402300 |
| C | 4.24618100 | -1.58979500 | 2.95916200 |
| H | 2.82392500 | -0.21779400 | 2.13937200 |
| C | 5.19598900 | -2.56220300 | 2.67935800 |
| H | 6.19758000 | -3.64698100 | 1.12040300 |
| H | 4.05762600 | -1.28367000 | 3.98124100 |
| H | 5.75228800 | -3.03250400 | 3.47977300 |

88

f-1-1 coplanar S1

| | | | |
|---|---|---|---|
| Au | -0.13313400 | 1.48592600 | -0.47994800 |
| N | 0.04228400 | 3.26197300 | 0.53549400 |
| C | 0.18859500 | 5.47342400 | 1.13221500 |
| C | -1.14636900 | -1.36559800 | 1.60002800 |
| N | -0.04862800 | -1.40713700 | -0.59414800 |
| C | -0.00812500 | -2.68587600 | -1.39642500 |
| C | -1.08518900 | -1.44884200 | 2.99550100 |
| H | -2.00369900 | -1.44538300 | 3.56664200 |
| C | -0.29220600 | 6.34289900 | -1.47141600 |
| H | -0.47574800 | 6.70710100 | -2.47299400 |
| C | -0.20663500 | -0.33093300 | -1.31004000 |
| C | 0.29818900 | 3.30864700 | 1.86912000 |
| C | 1.32253900 | -1.42159900 | 1.46499800 |
| C | 0.16578000 | -1.56851700 | 3.62265100 |
| C | -0.02681000 | 4.55227900 | 0.07568100 |
| C | 0.03360000 | -1.36421900 | 0.84918400 |
| C | 0.66822000 | 4.90968500 | 3.64322600 |
| H | 0.75747100 | 5.92217400 | 4.01655700 |
| C | 0.45426100 | 2.21987900 | 2.74091300 |
| H | 0.36831700 | 1.20138400 | 2.38045000 |
| C | 0.40475800 | 4.65540500 | 2.31888400 |
| C | -0.26820900 | 4.97474100 | -1.23056900 |
| H | -0.42845500 | 4.24956000 | -2.01837000 |

| | | | |
|---|---|---|---|
| C | 0.13580200 | -2.12185400 | -2.81642000 |
| H | -0.40696000 | -2.72596300 | -3.54553700 |
| H | 1.19557200 | -2.12065100 | -3.09014800 |
| C | 0.16108100 | 6.82621500 | 0.87575800 |
| H | 0.32332100 | 7.55134200 | 1.66342700 |
| C | 0.72063500 | 2.50081100 | 4.07111000 |
| H | 0.84900300 | 1.68513200 | 4.76915400 |
| C | 1.32021100 | -1.57021100 | 2.88316200 |
| H | 2.27544200 | -1.63111100 | 3.38982500 |
| C | -0.08198700 | 7.25394600 | -0.43822400 |
| H | -0.10575700 | 8.31392600 | -0.65315200 |
| C | -1.30097000 | -3.46726700 | -1.18664500 |
| H | -1.35990000 | -3.83276200 | -0.15987400 |
| H | -1.29537600 | -4.33082600 | -1.85338600 |
| H | -2.19160800 | -2.87264500 | -1.39391600 |
| C | 0.82604700 | 3.81863400 | 4.51745900 |
| H | 1.03593300 | 4.00873900 | 5.56156700 |
| C | 1.15947200 | -3.58024100 | -1.01061000 |
| H | 2.12121400 | -3.11243200 | -1.21819400 |
| H | 1.08624000 | -4.49695500 | -1.59955000 |
| H | 1.11407000 | -3.85001700 | 0.04649300 |
| C | -0.36142200 | -0.66959800 | -2.76558400 |
| C | -1.83765100 | -0.50768700 | -3.14583500 |
| H | -2.17282100 | 0.51196400 | -2.93769600 |
| H | -1.95468900 | -0.69468300 | -4.21557100 |
| H | -2.48672500 | -1.19557600 | -2.60411600 |
| C | 0.47380400 | 0.26436900 | -3.63421600 |
| H | 0.41234300 | -0.05306600 | -4.67753300 |
| H | 0.09888400 | 1.28835000 | -3.56180300 |
| H | 1.52155700 | 0.25719700 | -3.33343000 |
| C | 2.58516400 | -1.43794900 | 0.75857100 |
| C | 3.62374600 | -2.28658300 | 1.23182600 |
| C | 2.88999700 | -0.68031000 | -0.40183500 |
| C | 4.82567000 | -2.39743800 | 0.57602000 |

| | | | |
|---|---|---|---|
| H | 3.44354100 | -2.91085800 | 2.09729900 |
| C | 4.10594000 | -0.80923000 | -1.04540000 |
| H | 2.19154200 | 0.05567600 | -0.77205800 |
| C | 5.10367200 | -1.66974000 | -0.59008000 |
| H | 6.05601200 | -1.75408000 | -1.09321000 |
| C | -2.50730200 | -1.42911500 | 1.00023800 |
| C | -3.29699700 | -2.54041700 | 1.28259700 |
| C | -3.05163400 | -0.42305400 | 0.20215900 |
| C | -4.56617200 | -2.67469900 | 0.73203500 |
| H | -2.89741200 | -3.32514700 | 1.91609100 |
| C | -4.32145600 | -0.56112000 | -0.33883700 |
| H | -2.48429700 | 0.47749700 | 0.00909700 |
| C | -5.08803000 | -1.69320800 | -0.09342500 |
| H | -6.07479100 | -1.79723500 | -0.52578000 |
| C | -5.33565300 | -3.93164700 | 1.02778100 |
| C | -4.90218800 | 0.50953100 | -1.22036400 |
| F | -6.55916800 | -3.91441900 | 0.49566900 |
| F | -4.70424700 | -5.01101100 | 0.54391100 |
| F | -5.47107500 | -4.12615000 | 2.34476800 |
| F | -5.10005900 | 0.05977900 | -2.46913600 |
| F | -4.11032000 | 1.58252800 | -1.30906800 |
| F | -6.09229000 | 0.92618500 | -0.77336400 |
| C | 5.88566300 | -3.33250600 | 1.07845500 |
| C | 4.34601700 | -0.02595300 | -2.30144800 |
| F | 6.18904500 | -4.27289100 | 0.16903600 |
| F | 5.53166500 | -3.97247400 | 2.19804400 |
| F | 7.03123300 | -2.68463000 | 1.34268100 |
| F | 5.63881800 | 0.24347000 | -2.50066700 |
| F | 3.69266400 | 1.14684500 | -2.31224000 |
| F | 3.92551200 | -0.69805400 | -3.39385800 |
| H | 0.22068100 | -1.65563400 | 4.70101700 |

88

f-1-1 rotated S1

| | | | |
|---|---|---|---|
| Au | 0.09566000 | 1.54334900 | 0.38922400 |

| | | | |
|---|---|---|---|
| N | 0.06219900 | 3.44723400 | -0.38138000 |
| C | 0.72882600 | 5.46770200 | -1.25983500 |
| C | 1.12873300 | -1.52170400 | -1.64946600 |
| N | -0.08461700 | -1.44525500 | 0.49255800 |
| C | -0.14479900 | -2.64803800 | 1.35884100 |
| C | 1.10329800 | -1.58062000 | -3.04273700 |
| H | 2.04524600 | -1.62813900 | -3.57648200 |
| C | 3.41792500 | 4.74529300 | -1.20930600 |
| H | 4.46891200 | 4.49093900 | -1.19664000 |
| C | 0.12162700 | -0.26374000 | 1.17939700 |
| C | -1.05770900 | 4.21135800 | -0.58201500 |
| C | -1.29654800 | -1.48662100 | -1.63427300 |
| C | -0.09768600 | -1.58393500 | -3.73647300 |
| C | 1.13796400 | 4.19472700 | -0.78389000 |
| C | -0.07883800 | -1.47684600 | -0.92850500 |
| C | -1.71270200 | 6.39432500 | -1.39847300 |
| H | -1.48106000 | 7.36750000 | -1.81341500 |
| C | -2.38156000 | 3.85996400 | -0.30809800 |
| H | -2.61284700 | 2.88958300 | 0.11166400 |
| C | -0.72254400 | 5.47872400 | -1.12648100 |
| C | 2.48382900 | 3.82403800 | -0.75371500 |
| H | 2.77343300 | 2.85128700 | -0.37896300 |
| C | -0.36276400 | -1.99331600 | 2.72924400 |
| H | 0.06698000 | -2.59043800 | 3.53709600 |
| H | -1.44193600 | -1.90858000 | 2.90211700 |
| C | 1.66707500 | 6.36885700 | -1.70820900 |
| H | 1.37859900 | 7.34628500 | -2.07469600 |
| C | -3.36838600 | 4.79679100 | -0.58757200 |
| H | -4.40341600 | 4.55797900 | -0.38479800 |
| C | -1.28546700 | -1.54600300 | -3.03205300 |
| H | -2.23145500 | -1.54388200 | -3.56036300 |
| C | 3.01952200 | 5.99540300 | -1.67931200 |
| H | 3.76849300 | 6.69436100 | -2.02754300 |
| C | 1.14455100 | -3.46922800 | 1.28909400 |

| | | | |
|---|---|---|---|
| H | 1.23664300 | -3.94359200 | 0.30845700 |
| H | 1.11120100 | -4.26011800 | 2.04134500 |
| H | 2.03437500 | -2.86373600 | 1.45958800 |
| C | -3.04320900 | 6.04122600 | -1.12360600 |
| H | -3.83220500 | 6.75186200 | -1.33162600 |
| C | -1.29572600 | -3.58180600 | 1.00131200 |
| H | -2.26622500 | -3.10914500 | 1.15683000 |
| H | -1.24152100 | -4.46748800 | 1.63761600 |
| H | -1.22321000 | -3.91485000 | -0.03806000 |
| C | 0.22669900 | -0.57577700 | 2.65308400 |
| C | 1.68124700 | -0.51619700 | 3.13870900 |
| H | 2.07689600 | 0.49111100 | 2.98549100 |
| H | 1.73671800 | -0.75214100 | 4.20624700 |
| H | 2.32616000 | -1.21237600 | 2.60274300 |
| C | -0.58356900 | 0.40527300 | 3.49718700 |
| H | -0.55648200 | 0.11787100 | 4.55226100 |
| H | -0.16793500 | 1.41204000 | 3.40594600 |
| H | -1.62385400 | 0.43540200 | 3.16709900 |
| C | -2.61440000 | -1.52723900 | -0.95571500 |
| C | -3.55330200 | -2.46686400 | -1.37717700 |
| C | -2.96091800 | -0.67605200 | 0.09650900 |
| C | -4.79379500 | -2.56513800 | -0.75924800 |
| H | -3.30104700 | -3.15789800 | -2.17431500 |
| C | -4.20041300 | -0.78492400 | 0.70539800 |
| H | -2.25069800 | 0.06958500 | 0.44093800 |
| C | -5.13249400 | -1.73126600 | 0.29121700 |
| H | -6.09918100 | -1.80558600 | 0.76966700 |
| C | 2.46431700 | -1.63302000 | -1.00121800 |
| C | 3.21536000 | -2.77880300 | -1.23888900 |
| C | 3.01579700 | -0.63057300 | -0.20114200 |
| C | 4.46038400 | -2.95350100 | -0.64485200 |
| H | 2.80835500 | -3.56188600 | -1.87013600 |
| C | 4.26286400 | -0.80999500 | 0.37764400 |
| H | 2.45503300 | 0.27730900 | -0.02061300 |

| | | | |
|---|---|---|---|
| C | 4.99188700 | -1.97647900 | 0.17784000 |
| H | 5.96076000 | -2.10842700 | 0.64200500 |
| C | 5.19178700 | -4.24065100 | -0.90006400 |
| C | 4.87015100 | 0.25580500 | 1.24698200 |
| F | 6.40122600 | -4.25828100 | -0.33687900 |
| F | 4.51169200 | -5.29160800 | -0.42081500 |
| F | 5.35557100 | -4.45952500 | -2.21169700 |
| F | 5.03088200 | -0.16608900 | 2.50731100 |
| F | 4.12811700 | 1.36681700 | 1.29020400 |
| F | 6.08677100 | 0.61054700 | 0.80724600 |
| C | -5.74828500 | -3.61520100 | -1.25580600 |
| C | -4.52870000 | 0.10100800 | 1.87493000 |
| F | -5.23861700 | -4.84606800 | -1.11435600 |
| F | -6.01052100 | -3.45919800 | -2.56076600 |
| F | -6.91459500 | -3.58935500 | -0.60835700 |
| F | -5.84172200 | 0.33779700 | 1.96880400 |
| F | -3.91537800 | 1.28889500 | 1.79629500 |
| F | -4.15244600 | -0.45461700 | 3.03591800 |
| H | -0.10393800 | -1.61882700 | -4.81808800 |

88

f-1-1 coplanar T1

| | | | |
|---|---|---|---|
| Au | -0.25877400 | 1.54128300 | -0.44710900 |
| N | -0.39872400 | 3.34802400 | 0.51619800 |
| C | -0.65736700 | 5.57451100 | 1.02225900 |
| C | -0.93949200 | -1.44961800 | 1.59848400 |
| N | 0.21734200 | -1.37296500 | -0.57552100 |
| C | 0.37432800 | -2.58729700 | -1.41354600 |
| C | -0.87947900 | -1.42563100 | 2.99165200 |
| H | -1.80152200 | -1.52877800 | 3.55149300 |
| C | -1.07052300 | 6.25137400 | -1.65149300 |
| H | -1.23424900 | 6.54387100 | -2.67969400 |
| C | -0.15629600 | -0.24732700 | -1.28072400 |
| C | -0.26033000 | 3.49038100 | 1.87170800 |
| C | 1.47512200 | -1.20110200 | 1.51636800 |

| | | | |
|---|---|---|---|
| C | 0.33458300 | -1.28881600 | 3.65297600 |
| C | -0.63861600 | 4.58864400 | -0.00068100 |
| C | 0.24551900 | -1.34428900 | 0.84532300 |
| C | -0.29615400 | 5.19107400 | 3.58906900 |
| H | -0.40245000 | 6.21817400 | 3.91555200 |
| C | -0.00690500 | 2.47771600 | 2.79883900 |
| H | 0.09758500 | 1.44851300 | 2.47463200 |
| C | -0.40631500 | 4.84599700 | 2.26139600 |
| C | -0.84427900 | 4.91677100 | -1.34289700 |
| H | -0.82426900 | 4.14364500 | -2.10012800 |
| C | 0.48897400 | -1.94705200 | -2.80380700 |
| H | 0.10669200 | -2.60741800 | -3.58584500 |
| H | 1.54843200 | -1.75318800 | -3.00671900 |
| C | -0.88250700 | 6.89186700 | 0.69792000 |
| H | -0.90151600 | 7.66403100 | 1.45693100 |
| C | 0.10132700 | 2.84477500 | 4.13410300 |
| H | 0.29747900 | 2.08639300 | 4.88053800 |
| C | 1.49951700 | -1.18631900 | 2.91676000 |
| H | 2.45346600 | -1.07536700 | 3.41864100 |
| C | -1.08983100 | 7.22238700 | -0.65133300 |
| H | -1.26853100 | 8.25517000 | -0.91992200 |
| C | -0.83016400 | -3.52268100 | -1.29025700 |
| H | -0.84902700 | -3.98618400 | -0.30054800 |
| H | -0.74635100 | -4.32003800 | -2.03169800 |
| H | -1.77626700 | -3.00328500 | -1.44154800 |
| C | -0.04013700 | 4.17544400 | 4.52423000 |
| H | 0.04876000 | 4.43324300 | 5.57137600 |
| C | 1.61767500 | -3.39672200 | -1.06528900 |
| H | 2.53523800 | -2.84125100 | -1.26237200 |
| H | 1.62941000 | -4.30292300 | -1.67425100 |
| H | 1.60459800 | -3.70299300 | -0.01525800 |
| C | -0.24870700 | -0.59789100 | -2.74422300 |
| C | -1.70766500 | -0.69820700 | -3.20833100 |
| H | -2.20680500 | 0.26247000 | -3.05714800 |

| | | | |
|---|---|---|---|
| H | -1.75241200 | -0.94876500 | -4.27290300 |
| H | -2.26827700 | -1.45309000 | -2.65732200 |
| C | 0.44445500 | 0.44997800 | -3.61242100 |
| H | 0.43032600 | 0.14973400 | -4.66407200 |
| H | -0.07177000 | 1.40995200 | -3.52306400 |
| H | 1.48085000 | 0.59154500 | -3.30006700 |
| C | 2.77258400 | -1.15839500 | 0.79997600 |
| C | 3.80219100 | -1.98910500 | 1.23681300 |
| C | 3.01022600 | -0.33611500 | -0.30499500 |
| C | 5.02782300 | -2.01223400 | 0.58207200 |
| H | 3.63523500 | -2.65765000 | 2.07457800 |
| C | 4.23484900 | -0.37104100 | -0.95046000 |
| H | 2.23101100 | 0.33199500 | -0.65813300 |
| C | 5.25861600 | -1.21062800 | -0.52087400 |
| H | 6.21240100 | -1.22737600 | -1.02973600 |
| C | -2.26747700 | -1.73199900 | 0.98542400 |
| C | -2.86137700 | -2.95865800 | 1.25803300 |
| C | -2.95546200 | -0.82091800 | 0.18142500 |
| C | -4.08126200 | -3.30419500 | 0.68586900 |
| H | -2.34749600 | -3.67095000 | 1.89518700 |
| C | -4.17623000 | -1.17167900 | -0.37548900 |
| H | -2.53014200 | 0.15368700 | -0.01398000 |
| C | -4.74339400 | -2.41982000 | -0.14678300 |
| H | -5.69110300 | -2.68546400 | -0.59735100 |
| C | -4.63294300 | -4.67146300 | 0.97078100 |
| C | -4.92627400 | -0.20820900 | -1.25342100 |
| F | -5.83047200 | -4.86309700 | 0.41490900 |
| F | -3.81873400 | -5.63150200 | 0.50837300 |
| F | -4.76164400 | -4.88488200 | 2.28742800 |
| F | -5.07235000 | -0.69015400 | -2.49614700 |
| F | -4.31746700 | 0.97429800 | -1.35262000 |
| F | -6.16335600 | 0.01650100 | -0.78739300 |
| C | 6.08956100 | -2.93944200 | 1.10443100 |
| C | 4.44700100 | 0.47147900 | -2.17809600 |

| | | | |
|---|---|---|---|
| F | 7.20248100 | -2.90442800 | 0.36981900 |
| F | 5.66279000 | -4.20912800 | 1.12685200 |
| F | 6.43117000 | -2.63071600 | 2.36347500 |
| F | 5.73145200 | 0.81028300 | -2.33409900 |
| F | 3.73467400 | 1.59990000 | -2.14640000 |
| F | 4.08529200 | -0.18891300 | -3.29086200 |
| H | 0.36797900 | -1.27120400 | 4.73493300 |

93

f-5-1 coplanar S1

| | | | |
|---|---|---|---|
| Au | 0.07529500 | 1.52570400 | 0.28624000 |
| N | -0.14732100 | 3.32412900 | -0.67815000 |
| C | -0.37648600 | 5.54704300 | -1.20199800 |
| C | 1.09767700 | -1.25916700 | -1.86965300 |
| N | 0.05092600 | -1.37154700 | 0.34434800 |
| C | 0.05574400 | -2.66478900 | 1.12364200 |
| C | 1.00599500 | -1.29876500 | -3.26638700 |
| H | 1.91087700 | -1.26643200 | -3.85799900 |
| C | 0.04651900 | 6.34596300 | 1.43417000 |
| H | 0.20667500 | 6.68293100 | 2.44919200 |
| C | 0.19664700 | -0.30536900 | 1.07679200 |
| C | -0.39600900 | 3.40563200 | -2.01150800 |
| C | -1.36754000 | -1.35432200 | -1.68044100 |
| C | -0.25784600 | -1.41265300 | -3.86757000 |
| C | -0.13421700 | 4.59989700 | -0.17470700 |
| C | -0.06527500 | -1.29599700 | -1.09495100 |
| C | -0.81534900 | 5.04949400 | -3.73460500 |
| H | -0.94075500 | 6.06967100 | -4.07516200 |
| C | -0.50778400 | 2.34111800 | -2.91897300 |
| H | -0.38928600 | 1.31513000 | -2.59039500 |
| C | -0.55095700 | 4.76156600 | -2.41728200 |
| C | 0.07899600 | 4.98684100 | 1.14735100 |
| H | 0.26107900 | 4.24214700 | 1.91193400 |
| C | -0.07742300 | -2.12974000 | 2.55628500 |
| H | 0.49077800 | -2.72823100 | 3.27088200 |

| | | | |
|---|---|---|---|
| H | -1.13382700 | -2.15809900 | 2.84511800 |
| C | -0.40462400 | 6.89040800 | -0.90007000 |
| H | -0.58836400 | 7.63493100 | -1.66457400 |
| C | -0.77601500 | 2.65558800 | -4.24116600 |
| H | -0.87129200 | 1.85913100 | -4.96621400 |
| C | -1.39553300 | -1.45356900 | -3.10338600 |
| H | -2.36086200 | -1.51043700 | -3.59088600 |
| C | -0.19036400 | 7.28251800 | 0.42999000 |
| H | -0.21037900 | 8.33473100 | 0.68051600 |
| C | 1.36202500 | -3.41338200 | 0.87826400 |
| H | 1.40681400 | -3.76918500 | -0.15262900 |
| H | 1.39035200 | -4.28197800 | 1.53773800 |
| H | 2.24359100 | -2.80044400 | 1.07103100 |
| C | -0.92663300 | 3.98277700 | -4.64515000 |
| H | -1.13683900 | 4.19925700 | -5.68405100 |
| C | -1.09574100 | -3.58128900 | 0.73987300 |
| H | -2.06558400 | -3.14676700 | 0.97912600 |
| H | -0.98425000 | -4.51002100 | 1.30331900 |
| H | -1.06514300 | -3.82318400 | -0.32454600 |
| C | -2.61020200 | -1.43091400 | -0.94307700 |
| C | -3.63582300 | -2.29159300 | -1.42377800 |
| C | -2.90562000 | -0.73504400 | 0.25874500 |
| C | -4.80900800 | -2.47907500 | -0.73514600 |
| H | -3.46349100 | -2.86758000 | -2.32362200 |
| C | -4.09163900 | -0.94402700 | 0.93730800 |
| H | -2.22396000 | 0.01101900 | 0.64042500 |
| C | -5.07130100 | -1.82116500 | 0.47619500 |
| H | -5.99944500 | -1.96977300 | 1.00847800 |
| C | 2.47001400 | -1.33367700 | -1.29765100 |
| C | 3.26097000 | -2.42664200 | -1.64226800 |
| C | 3.02127300 | -0.36282900 | -0.46064100 |
| C | 4.53753100 | -2.58111400 | -1.11562400 |
| H | 2.85507600 | -3.18360400 | -2.30487300 |
| C | 4.30005200 | -0.52012000 | 0.05497300 |

| | | | |
|---|---|---|---|
| H | 2.45329700 | 0.52581700 | -0.21874000 |
| C | 5.06709300 | -1.63649400 | -0.25280900 |
| H | 6.06092400 | -1.75503700 | 0.15885200 |
| C | 5.30828500 | -3.81886500 | -1.48123800 |
| C | 4.88987900 | 0.50999400 | 0.97827800 |
| F | 6.53370100 | -3.82760100 | -0.95324900 |
| F | 4.68055600 | -4.92377600 | -1.05408900 |
| F | 5.43959200 | -3.94181100 | -2.80727600 |
| F | 5.02131800 | 0.03177100 | 2.22693200 |
| F | 4.14036700 | 1.61325000 | 1.05747900 |
| F | 6.11250200 | 0.88283500 | 0.58529600 |
| C | -5.85530500 | -3.42145100 | -1.25169000 |
| C | -4.30919000 | -0.23384700 | 2.24011100 |
| F | -5.49674700 | -4.03191800 | -2.38615400 |
| F | -7.01318300 | -2.78760100 | -1.49750100 |
| F | -6.13874500 | -4.38483800 | -0.36026600 |
| F | -5.60188600 | -0.08586700 | 2.53834300 |
| F | -3.75721200 | 0.98986400 | 2.25992300 |
| F | -3.75657000 | -0.90814800 | 3.27286200 |
| H | -0.33671800 | -1.46478700 | -4.94679200 |
| C | 0.38233700 | -0.66547500 | 2.51800600 |
| C | 1.85937300 | -0.46917800 | 2.89155800 |
| C | -0.44804100 | 0.23799600 | 3.43168000 |
| C | 2.06644600 | -0.56588200 | 4.37486700 |
| H | 2.19417300 | 0.50982000 | 2.52262200 |
| H | 2.48385900 | -1.20976600 | 2.38453400 |
| C | -0.34977800 | -0.24562300 | 4.87137000 |
| H | -0.05292300 | 1.25755600 | 3.35296400 |
| H | -1.48660100 | 0.26032700 | 3.09776800 |
| C | 1.07664800 | -0.49508800 | 5.26072500 |
| H | 3.08641300 | -0.71573600 | 4.71295400 |
| H | -0.79416400 | 0.49262100 | 5.54308900 |
| H | -0.94361300 | -1.15810000 | 5.00562800 |
| H | 1.29797600 | -0.60812300 | 6.31680100 |

93

f-5-1 rotated S1

| | | | |
|---|---|---|---|
| Au | 0.07811000 | 1.56135600 | 0.20850800 |
| N | 0.02506800 | 3.47774500 | -0.52855200 |
| C | 0.66671300 | 5.50121100 | -1.41816900 |
| C | 1.08434000 | -1.43441900 | -1.90125100 |
| N | -0.10523600 | -1.42598100 | 0.25565300 |
| C | -0.13834000 | -2.64586200 | 1.09910500 |
| C | 1.04050600 | -1.45134400 | -3.29504400 |
| H | 1.97544000 | -1.47443500 | -3.84257300 |
| C | 3.35483300 | 4.77522500 | -1.45694600 |
| H | 4.40535100 | 4.51956700 | -1.47867900 |
| C | 0.13223800 | -0.26257600 | 0.95950300 |
| C | -1.09922300 | 4.24435900 | -0.68880600 |
| C | -1.34194300 | -1.42291600 | -1.85391200 |
| C | -0.17016400 | -1.44690500 | -3.97276900 |
| C | 1.08899200 | 4.22547200 | -0.96210100 |
| C | -0.11486000 | -1.42318000 | -1.16533600 |
| C | -1.77642900 | 6.43211300 | -1.47361600 |
| H | -1.55672200 | 7.40675500 | -1.89154200 |
| C | -2.41431300 | 3.89362200 | -0.37371800 |
| H | -2.63351600 | 2.92151800 | 0.04883600 |
| C | -0.77961900 | 5.51387000 | -1.23773800 |
| C | 2.43439200 | 3.85288400 | -0.97620800 |
| H | 2.73496700 | 2.87812900 | -0.61522100 |
| C | -0.33696800 | -2.02048700 | 2.48570200 |
| H | 0.10822900 | -2.62735100 | 3.27789600 |
| H | -1.41498100 | -1.94599500 | 2.67641800 |
| C | 1.59127900 | 6.40323800 | -1.89228200 |
| H | 1.29265000 | 7.38273300 | -2.24481400 |
| C | -3.40790400 | 4.83314000 | -0.61711000 |
| H | -4.43635000 | 4.59504000 | -0.38253600 |
| C | -1.34925300 | -1.44255600 | -3.25287600 |
| H | -2.30199500 | -1.43505100 | -3.76890500 |

| | | | |
|---|---|---|---|
| C | 2.94340300 | 6.02784000 | -1.90842200 |
| H | 3.68183400 | 6.72739600 | -2.27727900 |
| C | 1.15860900 | -3.45068300 | 0.99376600 |
| H | 1.23756500 | -3.91383800 | 0.00682300 |
| H | 1.15039500 | -4.24876900 | 1.73898400 |
| H | 2.04320900 | -2.83404000 | 1.15306000 |
| C | -3.09793700 | 6.07958500 | -1.15773800 |
| H | -3.89210300 | 6.79224300 | -1.33708600 |
| C | -1.28565100 | -3.58375200 | 0.74259200 |
| H | -2.25814400 | -3.12488700 | 0.92497700 |
| H | -1.21062300 | -4.48164000 | 1.35935200 |
| H | -1.22833200 | -3.89425300 | -0.30467200 |
| C | -2.64686700 | -1.49332000 | -1.15380100 |
| C | -3.58701300 | -2.42757100 | -1.58455600 |
| C | -2.97710800 | -0.67896300 | -0.06755900 |
| C | -4.81219700 | -2.55760500 | -0.94195200 |
| H | -3.34527400 | -3.09259900 | -2.40660600 |
| C | -4.20039400 | -0.82071000 | 0.56694800 |
| H | -2.26673700 | 0.06361200 | 0.28382100 |
| C | -5.13323500 | -1.76258300 | 0.14356000 |
| H | -6.08636200 | -1.86423000 | 0.64354200 |
| C | 2.42708300 | -1.56455500 | -1.27046100 |
| C | 3.16590400 | -2.71058900 | -1.54172400 |
| C | 2.99411400 | -0.58647500 | -0.45048600 |
| C | 4.41201700 | -2.91354900 | -0.95849400 |
| H | 2.74715000 | -3.47366800 | -2.18956000 |
| C | 4.24319200 | -0.79320100 | 0.11673900 |
| H | 2.44635700 | 0.32445700 | -0.24874200 |
| C | 4.95747400 | -1.96298400 | -0.11414000 |
| H | 5.92745000 | -2.11648800 | 0.34091800 |
| C | 5.12755300 | -4.20193800 | -1.24837400 |
| C | 4.86525900 | 0.24447300 | 1.00903400 |
| F | 6.33809000 | -4.24811300 | -0.68904500 |
| F | 4.43627800 | -5.25687200 | -0.79410900 |

| | | | |
|---|---|---|---|
| F | 5.28566100 | -4.38964800 | -2.56559000 |
| F | 4.95857900 | -0.18148100 | 2.27595700 |
| F | 4.17185000 | 1.38791400 | 1.02487900 |
| F | 6.11189500 | 0.54358200 | 0.61729200 |
| C | -5.77818200 | -3.58602900 | -1.46120800 |
| C | -4.50499300 | 0.02140100 | 1.77447600 |
| F | -5.21190000 | -4.79732600 | -1.53448900 |
| F | -6.19452900 | -3.28423600 | -2.69924800 |
| F | -6.86375900 | -3.69682000 | -0.69327600 |
| F | -5.81899300 | 0.20826800 | 1.93598500 |
| F | -3.93756500 | 1.23251300 | 1.70069500 |
| F | -4.05457700 | -0.54990200 | 2.90207900 |
| H | -0.19015400 | -1.45027100 | -5.05478800 |
| C | 0.23973800 | -0.59692100 | 2.42458800 |
| C | 1.69433300 | -0.51717600 | 2.91869500 |
| C | -0.57390200 | 0.36389400 | 3.29827200 |
| C | 1.80159900 | -0.62955000 | 4.41194900 |
| H | 2.11595900 | 0.43740400 | 2.57750200 |
| H | 2.30370700 | -1.29353900 | 2.44786500 |
| C | -0.60382900 | -0.10218500 | 4.74839500 |
| H | -0.11112500 | 1.35550900 | 3.23507500 |
| H | -1.58625800 | 0.45350200 | 2.89490700 |
| C | 0.76767100 | -0.46626800 | 5.23298400 |
| H | 2.77902300 | -0.86630500 | 4.82089500 |
| H | -1.02649800 | 0.67881500 | 5.38620000 |
| H | -1.27672100 | -0.96306900 | 4.85471400 |
| H | 0.90835800 | -0.58949600 | 6.30216200 |

93

f-5-1 coplanar T1

| | | | |
|---|---|---|---|
| Au | 0.26627800 | 1.56749400 | 0.24879600 |
| N | 0.41593000 | 3.39667000 | -0.66888300 |
| C | 0.70600700 | 5.63176800 | -1.11685700 |
| C | 0.85720200 | -1.37615200 | -1.86823600 |
| N | -0.25189300 | -1.34296600 | 0.32981500 |

| | | | |
|---|---|---|---|
| C | -0.39805600 | -2.57056000 | 1.14970200 |
| C | 0.76769700 | -1.30796000 | -3.25839700 |
| H | 1.67572600 | -1.40543500 | -3.84166700 |
| C | 1.21518500 | 6.21927500 | 1.56144000 |
| H | 1.41676000 | 6.47720100 | 2.59216000 |
| C | 0.15888000 | -0.23794300 | 1.04481900 |
| C | 0.23945900 | 3.58253700 | -2.01455100 |
| C | -1.55300000 | -1.10290700 | -1.72602700 |
| C | -0.45896000 | -1.13638500 | -3.88790200 |
| C | 0.69804300 | 4.61625000 | -0.12333500 |
| C | -0.31067800 | -1.27952600 | -1.08846400 |
| C | 0.25865800 | 5.33262000 | -3.68156100 |
| H | 0.37639400 | 6.36665900 | -3.98107600 |
| C | -0.06316700 | 2.60327200 | -2.96242900 |
| H | -0.17928900 | 1.56733300 | -2.66490300 |
| C | 0.40199400 | 4.94601200 | -2.36856600 |
| C | 0.95184800 | 4.89927700 | 1.22081800 |
| H | 0.93953900 | 4.10409500 | 1.95511300 |
| C | -0.49021700 | -1.95315500 | 2.55176200 |
| H | -0.10280100 | -2.62405000 | 3.32236500 |
| H | -1.54723200 | -1.75558700 | 2.76932400 |
| C | 0.96833400 | 6.93404600 | -0.76094000 |
| H | 0.98007600 | 7.72826100 | -1.49699300 |
| C | -0.20409900 | 3.01193000 | -4.28240200 |
| H | -0.43853800 | 2.28032600 | -5.04436100 |
| C | -1.60743400 | -1.04478100 | -3.12440400 |
| H | -2.57049000 | -0.90961200 | -3.60240300 |
| C | 1.22380300 | 7.21943800 | 0.59032900 |
| H | 1.43210000 | 8.23987900 | 0.88362500 |
| C | 0.80327800 | -3.50569200 | 0.99545600 |
| H | 0.79891100 | -3.96857000 | 0.00539400 |
| H | 0.73775700 | -4.30310500 | 1.73858300 |
| H | 1.75216300 | -2.98483800 | 1.12432300 |
| C | -0.04677500 | 4.35062100 | -4.63759800 |

| | | | |
|---|---|---|---|
| H | -0.16203100 | 4.64134300 | -5.67348700 |
| C | -1.64692400 | -3.37307400 | 0.80557100 |
| H | -2.56194800 | -2.82483400 | 1.03241100 |
| H | -1.64578600 | -4.29362100 | 1.39263800 |
| H | -1.65233200 | -3.65413500 | -0.25159600 |
| C | -2.83192100 | -1.07790700 | -0.97571300 |
| C | -3.87572500 | -1.88981200 | -1.41458400 |
| C | -3.03619500 | -0.29844600 | 0.16631900 |
| C | -5.08175400 | -1.93769200 | -0.72575500 |
| H | -3.73419500 | -2.52571800 | -2.28189400 |
| C | -4.24111700 | -0.35958500 | 0.84709500 |
| H | -2.24627400 | 0.35666400 | 0.52078400 |
| C | -5.27811900 | -1.18139300 | 0.41527900 |
| H | -6.21594100 | -1.21925500 | 0.95167400 |
| C | 2.18936700 | -1.70506300 | -1.28758600 |
| C | 2.74685600 | -2.93680500 | -1.60920700 |
| C | 2.91098200 | -0.84082700 | -0.46036500 |
| C | 3.96182600 | -3.33637700 | -1.06231500 |
| H | 2.20653200 | -3.61220900 | -2.26440700 |
| C | 4.12731900 | -1.24533800 | 0.07088000 |
| H | 2.51722100 | 0.13935500 | -0.23026400 |
| C | 4.65612600 | -2.50055000 | -0.20612600 |
| H | 5.60107900 | -2.80754400 | 0.22333900 |
| C | 4.47207400 | -4.70748300 | -1.40039200 |
| C | 4.91247600 | -0.33605300 | 0.97548900 |
| F | 5.66747500 | -4.95281800 | -0.86129200 |
| F | 3.63391800 | -5.66006900 | -0.96605700 |
| F | 4.58539000 | -4.87715800 | -2.72482200 |
| F | 4.97805000 | -0.82235600 | 2.22458600 |
| F | 4.38727300 | 0.88759900 | 1.05204900 |
| F | 6.17864500 | -0.20339700 | 0.55617200 |
| C | -6.15957300 | -2.84386500 | -1.25212600 |
| C | -4.41040400 | 0.43344300 | 2.11385000 |
| F | -5.73902400 | -4.11372000 | -1.32510800 |

| | | | |
|---|---|---|---|
| F | -6.52933900 | -2.49377400 | -2.49218200 |
| F | -7.25459700 | -2.82839100 | -0.49042000 |
| F | -5.69434400 | 0.71099800 | 2.36232800 |
| F | -3.74890400 | 1.59266800 | 2.07516000 |
| F | -3.94937200 | -0.24358400 | 3.18011400 |
| H | -0.51522000 | -1.08470400 | -4.96784600 |
| C | 0.25208000 | -0.60649200 | 2.50030900 |
| C | 1.71311600 | -0.70653900 | 2.96942200 |
| C | -0.42916900 | 0.43380700 | 3.39586600 |
| C | 1.82986300 | -0.84166400 | 4.46010500 |
| H | 2.24363000 | 0.19073800 | 2.62437000 |
| H | 2.21563100 | -1.54863300 | 2.48578300 |
| C | -0.49216000 | -0.04093800 | 4.84161100 |
| H | 0.14889600 | 1.36388300 | 3.33274600 |
| H | -1.42897700 | 0.64907200 | 3.01025100 |
| C | 0.83512100 | -0.56715300 | 5.29990200 |
| H | 2.77946200 | -1.19428700 | 4.85061900 |
| H | -0.81207100 | 0.77655500 | 5.49341000 |
| H | -1.26012100 | -0.81798300 | 4.94920400 |
| H | 0.97776800 | -0.71625100 | 6.36551500 |

62

r-1-1 coplanar S1

| | | | |
|---|---|---|---|
| Au | 0.97647400 | 1.15378900 | -0.02922600 |
| N | 2.51878000 | -0.19971300 | 0.12573100 |
| C | 4.56902500 | -1.23693300 | 0.17945100 |
| C | -2.29741400 | -0.07463900 | 1.15987000 |
| N | -1.82219600 | 1.83092900 | -0.25274100 |
| C | -2.92696200 | 2.83143300 | -0.35921700 |
| C | -2.66229800 | -1.45804600 | 1.28928300 |
| C | 5.93709300 | 1.17360100 | 0.44282400 |
| H | 6.49122800 | 2.09700600 | 0.54532900 |
| C | -0.62035800 | 2.32085400 | -0.22304700 |
| C | 2.30472900 | -1.52882200 | 0.00264000 |
| C | -2.09953300 | -0.36332300 | -1.25677000 |

| | | | |
|---|---|---|---|
| C | -2.72485700 | -2.25951600 | 0.15986100 |
| C | 3.87763500 | -0.00109400 | 0.22416000 |
| C | -2.08378200 | 0.41781500 | -0.11217100 |
| C | 3.51581600 | -3.61841100 | -0.09974700 |
| H | 4.42940500 | -4.19971600 | -0.08663800 |
| C | 1.06449200 | -2.17278800 | -0.15514700 |
| H | 0.14582000 | -1.60101500 | -0.17122100 |
| C | 3.53454800 | -2.25175600 | 0.02870900 |
| C | 4.54919300 | 1.21017800 | 0.35478900 |
| H | 3.99603500 | 2.14064200 | 0.38473800 |
| C | -2.13213000 | 4.10719200 | -0.67749100 |
| H | -2.53998700 | 4.97167700 | -0.15068600 |
| H | -2.18987700 | 4.30998400 | -1.75016100 |
| C | 5.94475300 | -1.25543300 | 0.26775700 |
| H | 6.49641300 | -2.18685600 | 0.23495900 |
| C | 1.07305200 | -3.54959300 | -0.29407200 |
| H | 0.13801700 | -4.07503700 | -0.43208100 |
| C | -2.44374000 | -1.75000000 | -1.09755200 |
| C | 6.62453400 | -0.03692900 | 0.40079800 |
| H | 7.70421300 | -0.03732400 | 0.47108700 |
| C | -3.68369900 | 2.91657700 | 0.96159500 |
| H | -4.18864200 | 1.97528500 | 1.17859900 |
| H | -4.43343700 | 3.70649200 | 0.88820000 |
| H | -3.01480000 | 3.15011400 | 1.79125100 |
| C | 2.27287200 | -4.26202900 | -0.26269100 |
| H | 2.25110500 | -5.33828700 | -0.37069200 |
| C | -3.88014800 | 2.43974500 | -1.47614800 |
| H | -3.34591900 | 2.32093900 | -2.42029300 |
| H | -4.62956200 | 3.22475500 | -1.59413200 |
| H | -4.38836500 | 1.50328300 | -1.24357700 |
| C | -0.67046200 | 3.82433100 | -0.28823400 |
| C | -0.30400900 | 4.36220700 | 1.09840100 |
| H | 0.70850100 | 4.05357400 | 1.36705100 |
| H | -0.34533900 | 5.45375100 | 1.08651900 |

| | | | |
|---|---|---|---|
| H | -0.98417000 | 3.99761300 | 1.86932900 |
| C | 0.32262700 | 4.35954400 | -1.31395800 |
| H | 0.23919000 | 5.44683500 | -1.37927500 |
| H | 1.34430300 | 4.10600500 | -1.02215100 |
| H | 0.13466000 | 3.93764100 | -2.30302700 |
| C | -2.98254200 | -1.97401700 | 2.63467500 |
| C | -2.43553500 | -2.61155200 | -2.29697100 |
| H | -3.00291200 | -3.30046200 | 0.26177900 |
| H | -2.22930300 | 0.57322900 | 2.02325800 |
| H | -1.91873100 | 0.06983800 | -2.23061700 |
| F | -1.95720800 | -1.82450400 | 3.50615700 |
| F | -4.01722100 | -1.31955400 | 3.21335500 |
| F | -3.30095000 | -3.27498900 | 2.63706900 |
| F | -1.20365500 | -2.69238500 | -2.86259500 |
| F | -2.81359000 | -3.87244800 | -2.04442800 |
| F | -3.23435000 | -2.15161000 | -3.28280100 |

62

r-1-1 rotated S1

| | | | |
|---|---|---|---|
| Au | 0.93085700 | 1.03580600 | -0.17116600 |
| N | 2.70326700 | 0.08731200 | 0.26866300 |
| C | 4.56620400 | -0.64177200 | 1.40656500 |
| C | -3.39312500 | -0.24259600 | 0.64382200 |
| N | -2.03479000 | 1.50750100 | -0.39836500 |
| C | -3.02101900 | 2.61668900 | -0.35244000 |
| C | -3.66296400 | -1.59103300 | 0.81575800 |
| C | 3.87349000 | 0.77767600 | 3.70065600 |
| H | 3.62802900 | 1.31789800 | 4.60483000 |
| C | -0.75309600 | 1.97098700 | -0.62049600 |
| C | 3.41448300 | -0.73218200 | -0.56612700 |
| C | -1.62071700 | -0.82448300 | -0.86758400 |
| C | -2.92754600 | -2.57735100 | 0.17565800 |
| C | 3.38547100 | 0.14501800 | 1.45466300 |
| C | -2.35985900 | 0.17621800 | -0.20923900 |
| C | 5.43333100 | -2.06787700 | -0.60675900 |

| | | | |
|---|---|---|---|
| H | 6.33129000 | -2.45575300 | -0.14196000 |
| C | 3.08967300 | -1.09137900 | -1.87642000 |
| H | 2.18609100 | -0.71391100 | -2.33574800 |
| C | 4.58524900 | -1.22233600 | 0.07007000 |
| C | 3.03045000 | 0.86080900 | 2.60007600 |
| H | 2.12527100 | 1.45299500 | 2.61019000 |
| C | -2.32988600 | 3.64251300 | -1.26130500 |
| H | -2.59443600 | 4.66372800 | -0.97653900 |
| H | -2.67226800 | 3.48078800 | -2.28805600 |
| C | 5.39094000 | -0.71223600 | 2.50544000 |
| H | 6.29654800 | -1.30598100 | 2.49237900 |
| C | 3.95588900 | -1.94588100 | -2.54479900 |
| H | 3.73228300 | -2.24810600 | -3.55865800 |
| C | -1.90042100 | -2.15923600 | -0.67005000 |
| C | 5.03338300 | 0.00629600 | 3.65694500 |
| H | 5.67234100 | -0.04062900 | 4.52885600 |
| C | -3.16208700 | 3.15271000 | 1.07182300 |
| H | -3.68876400 | 2.45324300 | 1.72204200 |
| H | -3.72472900 | 4.08847100 | 1.06105000 |
| H | -2.17736900 | 3.33886600 | 1.50209900 |
| C | 5.10790100 | -2.42603100 | -1.92439900 |
| H | 5.76412200 | -3.09274500 | -2.46802200 |
| C | -4.37664100 | 2.24672300 | -0.93911400 |
| H | -4.24824600 | 1.71718400 | -1.88506100 |
| H | -4.92743600 | 3.16911000 | -1.13457000 |
| H | -4.98565900 | 1.63243300 | -0.27743500 |
| C | -0.81951600 | 3.37248700 | -1.17707000 |
| C | -0.08771500 | 4.38084600 | -0.28602500 |
| H | 0.96201800 | 4.09294400 | -0.18998500 |
| H | -0.13242400 | 5.38137400 | -0.72664100 |
| H | -0.51564600 | 4.42452800 | 0.71621400 |
| C | -0.18137600 | 3.42985300 | -2.56931400 |
| H | -0.29785000 | 4.42599700 | -3.00867900 |
| H | 0.88539000 | 3.20328600 | -2.50183300 |

| | | | |
|---|---|---|---|
| H | -0.64474800 | 2.69723700 | -3.23365200 |
| C | -4.80431000 | -1.96482200 | 1.71959200 |
| C | -1.11830800 | -3.18439200 | -1.44089800 |
| H | -3.13153600 | -3.62538000 | 0.33747300 |
| H | -3.96398000 | 0.47693600 | 1.21187400 |
| H | -0.84273300 | -0.52399600 | -1.55752500 |
| F | -4.79983500 | -1.24956900 | 2.85360600 |
| F | -5.99090600 | -1.73635300 | 1.13455100 |
| F | -4.78109100 | -3.25484100 | 2.06365700 |
| F | 0.12885200 | -2.77080000 | -1.71061300 |
| F | -1.01656300 | -4.34118300 | -0.77768200 |
| F | -1.68933300 | -3.46661100 | -2.62205200 |

62

r-1-1 coplanar T1

| | | | |
|---|---|---|---|
| Au | 1.10589400 | 0.87357500 | 0.10861600 |
| N | 2.77689000 | -0.27179600 | -0.24229400 |
| C | 4.92742200 | -0.88746300 | -0.78075700 |
| C | -1.79712300 | -0.51815900 | 0.85989600 |
| N | -1.77136900 | 1.77699800 | 0.09837500 |
| C | -2.56125200 | 3.02792200 | -0.08858000 |
| C | -2.31983600 | -1.79207100 | 0.81660700 |
| C | 5.68740900 | 1.74545700 | -1.28257500 |
| H | 6.00969300 | 2.75816900 | -1.48346500 |
| C | -0.45766900 | 2.03277900 | 0.44572300 |
| C | 2.86902600 | -1.62973900 | -0.11753600 |
| C | -3.38860200 | 0.16932200 | -0.80200900 |
| C | -3.37617800 | -2.12947200 | -0.03257400 |
| C | 4.00702700 | 0.18355500 | -0.63909600 |
| C | -2.31673400 | 0.50607700 | 0.03824500 |
| C | 4.46327200 | -3.43110300 | -0.36850600 |
| H | 5.45045300 | -3.80820600 | -0.60552600 |
| C | 1.85328600 | -2.50996800 | 0.26319700 |
| H | 0.86575600 | -2.13393300 | 0.49531000 |
| C | 4.17475000 | -2.08777000 | -0.43559600 |

| | | | |
|---|---|---|---|
| C | 4.37758200 | 1.50622400 | -0.88624900 |
| H | 3.65551300 | 2.30415800 | -0.76733300 |
| C | -1.59252000 | 4.10248900 | 0.45252000 |
| H | -2.10910900 | 4.78799600 | 1.12999300 |
| H | -1.20150700 | 4.68969200 | -0.38258600 |
| C | 6.22152700 | -0.63244700 | -1.17359800 |
| H | 6.94250700 | -1.43224200 | -1.28936100 |
| C | 2.16130800 | -3.86164900 | 0.32713800 |
| H | 1.39642200 | -4.56773100 | 0.61941000 |
| C | -3.89640900 | -1.12150800 | -0.82435900 |
| C | 6.59449300 | 0.69689700 | -1.42448300 |
| H | 7.60882100 | 0.91171500 | -1.73404300 |
| C | -3.86402300 | 2.99196400 | 0.70077000 |
| H | -4.53367200 | 2.20353800 | 0.35517000 |
| H | -4.37942900 | 3.94666500 | 0.58081000 |
| H | -3.66891200 | 2.83718500 | 1.76320700 |
| C | 3.44282300 | -4.31558800 | 0.01756800 |
| H | 3.65723900 | -5.37464200 | 0.07480900 |
| C | -2.82489500 | 3.33017900 | -1.56541800 |
| H | -1.90751000 | 3.18036600 | -2.13879800 |
| H | -3.12062400 | 4.37763400 | -1.65926500 |
| H | -3.62196000 | 2.73025300 | -2.00237500 |
| C | -0.42969500 | 3.36968800 | 1.13120700 |
| C | -0.71364900 | 3.16405200 | 2.62626600 |
| H | 0.09541800 | 2.59169200 | 3.08536400 |
| H | -0.80177700 | 4.12578000 | 3.14240500 |
| H | -1.64334500 | 2.60765300 | 2.76984400 |
| C | 0.88950600 | 4.10780700 | 0.96331900 |
| H | 0.84266500 | 5.09795800 | 1.42436600 |
| H | 1.69913000 | 3.54747800 | 1.43893900 |
| H | 1.12986800 | 4.22314200 | -0.09673900 |
| C | -1.77209000 | -2.87043400 | 1.70560100 |
| C | -5.05813000 | -1.38987000 | -1.74005100 |
| H | -3.77380800 | -3.13392500 | -0.06201500 |

| | | | |
|---|---|---|---|
| H | -1.00961900 | -0.26962100 | 1.55979400 |
| H | -3.82392200 | 0.90343500 | -1.46216600 |
| F | -0.84147800 | -2.42345800 | 2.55452200 |
| F | -2.72949300 | -3.45503900 | 2.43372800 |
| F | -1.19584000 | -3.85362500 | 0.98467600 |
| F | -4.81258500 | -0.97192600 | -2.98993900 |
| F | -5.36615600 | -2.68688900 | -1.80811700 |
| F | -6.16181800 | -0.74195700 | -1.33586800 |

89

e-8-1 coplanar S1

| | | | |
|---|---|---|---|
| Au | 0.50034700 | -0.27315100 | 0.02869900 |
| N | 2.58398500 | -0.14848200 | -0.06019800 |
| C | 4.81681300 | -0.61197200 | -0.32964500 |
| C | -1.33447000 | 2.86565800 | -0.91900900 |
| N | -2.13318900 | 0.94329400 | 0.37271200 |
| C | -3.58031000 | 0.80155200 | 0.61597000 |
| C | -0.75228800 | 4.13243600 | -0.93986100 |
| H | -0.66312100 | 4.65501800 | -1.88613200 |
| C | 4.44590000 | -3.30502600 | 0.30071100 |
| H | 4.33395500 | -4.35297900 | 0.54476100 |
| C | -1.48074700 | -0.20603000 | 0.01435600 |
| C | 3.20916600 | 1.00996200 | -0.40268600 |
| C | -0.45496200 | 1.92766700 | -3.05243500 |
| H | 0.21658400 | 1.26621300 | -2.50081600 |
| H | 0.08030000 | 2.85353900 | -3.28453800 |
| H | -0.71852500 | 1.44024800 | -3.99382600 |
| C | -0.89728000 | 2.75019800 | 1.48238400 |
| C | -0.27747200 | 4.72396100 | 0.22113600 |
| H | 0.15619700 | 5.71631500 | 0.18999700 |
| C | 3.52758200 | -1.12876000 | -0.01914300 |
| C | -1.46757100 | 2.20568800 | 0.31712100 |
| C | -2.43449900 | -1.37629900 | 0.09979300 |
| C | 0.69885700 | 1.73965700 | 3.11264400 |
| H | 1.21880900 | 1.24744100 | 2.28820300 |

| | | | |
|---|---|---|---|
| H | 0.78949200 | 1.10840200 | 3.99934200 |
| H | 1.20197500 | 2.68849400 | 3.31968900 |
| C | 5.41087300 | 1.87432000 | -0.91255900 |
| H | 6.47830900 | 1.75343900 | -1.05196000 |
| C | 2.61866900 | 2.27242400 | -0.54960100 |
| H | 1.55213200 | 2.40396400 | -0.39891800 |
| C | -1.71588300 | 2.22331200 | -2.23753000 |
| H | -2.18888000 | 1.26322000 | -2.02605800 |
| C | -0.77759600 | 1.97472100 | 2.77969800 |
| H | -1.24145000 | 0.99626200 | 2.63119000 |
| C | 4.60640500 | 0.80747300 | -0.58206100 |
| C | 3.33174000 | -2.47592600 | 0.30015300 |
| H | 2.33912300 | -2.83649500 | 0.54523200 |
| C | -3.78673500 | -0.72533700 | 0.49437300 |
| H | -4.55718800 | -0.94711200 | -0.24250300 |
| H | -4.14638900 | -1.13322200 | 1.43797600 |
| C | 5.90731600 | -1.45245500 | -0.32524600 |
| H | 6.90034900 | -1.08698400 | -0.55715000 |
| C | -2.66862100 | 3.08889000 | -3.06073300 |
| H | -3.55576200 | 3.37747400 | -2.49544000 |
| H | -2.98901200 | 2.54742200 | -3.95362600 |
| H | -2.17237800 | 4.00361600 | -3.39478700 |
| C | 3.44952500 | 3.33221400 | -0.88516300 |
| H | 3.02591600 | 4.32103100 | -1.00515200 |
| C | -0.32872500 | 4.02271400 | 1.41472300 |
| H | 0.09513100 | 4.45817300 | 2.31301800 |
| C | 5.71081800 | -2.80616900 | -0.00964600 |
| H | 6.56062100 | -3.47617800 | -0.00484000 |
| C | -1.43795800 | 2.68832400 | 3.95937900 |
| H | -2.48787400 | 2.91614200 | 3.77830900 |
| H | -0.92340300 | 3.62975400 | 4.16931300 |
| H | -1.36961800 | 2.07141500 | 4.85811000 |
| C | -4.40854900 | 1.58537400 | -0.40182600 |
| H | -4.16986800 | 2.65038200 | -0.34078900 |

| | | | |
|---|---|---|---|
| H | -5.46995700 | 1.46131600 | -0.17652300 |
| H | -4.23419300 | 1.25373900 | -1.42408100 |
| C | 4.81967300 | 3.13977800 | -1.06547100 |
| H | 5.44409300 | 3.98447300 | -1.32596300 |
| C | -3.97016300 | 1.32783400 | 1.99413700 |
| H | -3.42854900 | 0.82496700 | 2.79509700 |
| H | -5.04025700 | 1.17680600 | 2.15216200 |
| H | -3.76612000 | 2.39946400 | 2.05565300 |
| C | -1.87859500 | -2.29836400 | 1.18042400 |
| C | -2.06632200 | -1.99728000 | 2.53464000 |
| C | -1.05666000 | -3.38068800 | 0.85797600 |
| C | -1.47628300 | -2.74341700 | 3.53893100 |
| H | -2.66840300 | -1.14052900 | 2.81150900 |
| C | -0.46970100 | -4.13372100 | 1.87435700 |
| C | -0.66721900 | -3.82797300 | 3.21034300 |
| H | -1.64359900 | -2.47905500 | 4.57572400 |
| H | 0.15630000 | -4.97698000 | 1.59770200 |
| H | -0.20447800 | -4.42609600 | 3.98493900 |
| C | -2.46922500 | -2.03572300 | -1.27683800 |
| C | -3.25064000 | -1.49720600 | -2.30603600 |
| C | -1.66097300 | -3.13200200 | -1.57895700 |
| C | -3.25604900 | -2.03355200 | -3.58108700 |
| H | -3.86836200 | -0.63032400 | -2.11008300 |
| C | -1.67534100 | -3.67553000 | -2.86449900 |
| C | -2.46393400 | -3.14244700 | -3.86751300 |
| H | -3.87911200 | -1.59134400 | -4.34865800 |
| H | -1.04542400 | -4.53553000 | -3.06892400 |
| H | -2.46426000 | -3.58057900 | -4.85747900 |
| C | -0.71818500 | -3.70348900 | -0.56583200 |
| H | -0.64152200 | -4.78709900 | -0.69478200 |
| H | 0.28311400 | -3.30172100 | -0.77898200 |

89

e-8-1 rotated S1

| | | | |
|---|---|---|---|
| Au | -0.54491000 | -0.03320600 | -0.09402100 |

| | | | |
|---|---|---|---|
| N | -2.56270300 | -0.39122000 | -0.09416500 |
| C | -4.72153400 | -0.60900900 | 0.67622000 |
| C | 2.29226600 | -2.70438900 | -0.56835900 |
| N | 2.42032300 | -0.40549600 | 0.26348500 |
| C | 3.74429100 | 0.23912300 | 0.29281600 |
| C | 2.23188800 | -4.06950100 | -0.29472600 |
| H | 2.28244500 | -4.77549000 | -1.11670500 |
| C | -4.06016800 | 0.38448600 | 3.19096400 |
| H | -3.83214400 | 0.77525200 | 4.17329600 |
| C | 1.36537800 | 0.41895100 | -0.04807100 |
| C | -3.33293600 | -0.86995500 | -1.12212200 |
| C | 0.95215900 | -2.61043000 | -2.65638200 |
| H | 0.12723100 | -2.20798600 | -2.06502000 |
| H | 0.83519200 | -3.69621500 | -2.71836500 |
| H | 0.89224400 | -2.20082200 | -3.66793400 |
| C | 1.99987400 | -2.25509300 | 1.81248000 |
| C | 2.08833000 | -4.53436100 | 1.00278700 |
| H | 2.05361100 | -5.59896900 | 1.19937600 |
| C | -3.39295200 | -0.21814100 | 0.98468300 |
| C | 2.24449500 | -1.79875800 | 0.50571200 |
| C | 1.87581300 | 1.83537600 | -0.24884200 |
| C | 0.22688200 | -1.49783900 | 3.38261800 |
| H | -0.44242200 | -1.41100800 | 2.52436700 |
| H | -0.05183600 | -0.74118800 | 4.12123800 |
| H | 0.08071000 | -2.48358500 | 3.83360000 |
| C | -5.61436600 | -1.51809300 | -1.60815600 |
| H | -6.64880700 | -1.65898200 | -1.31984600 |
| C | -2.91730400 | -1.16699600 | -2.42128300 |
| H | -1.88490900 | -1.01542800 | -2.70831900 |
| C | 2.29299500 | -2.24909500 | -2.01342500 |
| H | 2.37105000 | -1.16036200 | -2.02492800 |
| C | 1.68503300 | -1.31170400 | 2.95531300 |
| H | 1.78602600 | -0.28969100 | 2.58456100 |
| C | -4.68209300 | -1.03968400 | -0.71599000 |

| | | | |
|---|---|---|---|
| C | -3.05189600 | 0.29149600 | 2.23972700 |
| H | -2.03642100 | 0.61100900 | 2.44030400 |
| C | 3.40807000 | 1.71284300 | -0.02676200 |
| H | 3.95291400 | 2.04226300 | -0.91073700 |
| H | 3.73051800 | 2.35983300 | 0.78842800 |
| C | -5.70753300 | -0.51107700 | 1.63136400 |
| H | -6.72804500 | -0.80545900 | 1.41933800 |
| C | 3.42924400 | -2.85491800 | -2.83497700 |
| H | 4.40770400 | -2.66068000 | -2.39504200 |
| H | 3.42007900 | -2.44638100 | -3.84814000 |
| H | 3.30960900 | -3.93809700 | -2.91900400 |
| C | -3.87204800 | -1.64431900 | -3.31013900 |
| H | -3.58515400 | -1.88271800 | -4.32531200 |
| C | 1.94529200 | -3.62873100 | 2.04164500 |
| H | 1.77180000 | -3.98926600 | 3.04991300 |
| C | -5.36320000 | -0.01302400 | 2.89692800 |
| H | -6.12785000 | 0.07037000 | 3.65811400 |
| C | 2.59243200 | -1.51319300 | 4.16778800 |
| H | 3.64922900 | -1.42747100 | 3.91488800 |
| H | 2.43298200 | -2.50181600 | 4.60598500 |
| H | 2.36150200 | -0.77366200 | 4.93816200 |
| C | 4.70061500 | -0.37052300 | -0.73177900 |
| H | 4.85906600 | -1.42943700 | -0.51227100 |
| H | 5.66608000 | 0.13680200 | -0.67394400 |
| H | 4.32899700 | -0.28334000 | -1.75179600 |
| C | -5.19616000 | -1.82115700 | -2.91261800 |
| H | -5.91813900 | -2.19795400 | -3.62516900 |
| C | 4.41925900 | 0.08273200 | 1.65456100 |
| H | 3.82659200 | 0.50968700 | 2.46252800 |
| H | 5.39031400 | 0.58237500 | 1.63913000 |
| H | 4.58454800 | -0.97577100 | 1.87109800 |
| C | 1.20023600 | 2.70395000 | 0.80905800 |
| C | 1.62694700 | 2.64030800 | 2.14135400 |
| C | 0.08177900 | 3.48490000 | 0.51941100 |

| | | | |
|---|---|---|---|
| C | 0.98016700 | 3.32385500 | 3.15394700 |
| H | 2.47764400 | 2.02037300 | 2.39440600 |
| C | -0.56639100 | 4.17835700 | 1.54401100 |
| C | -0.13379600 | 4.10672000 | 2.85551400 |
| H | 1.34236700 | 3.24965400 | 4.17203700 |
| H | -1.43132100 | 4.78349200 | 1.29045200 |
| H | -0.64726400 | 4.65649700 | 3.63421800 |
| C | 1.52325600 | 2.26320100 | -1.67000000 |
| C | 2.26694100 | 1.77617000 | -2.75157600 |
| C | 0.41415200 | 3.06082500 | -1.94793700 |
| C | 1.94508400 | 2.08027900 | -4.06112100 |
| H | 3.11371100 | 1.12870000 | -2.56167700 |
| C | 0.09533300 | 3.37120100 | -3.27201200 |
| C | 0.84647400 | 2.89404500 | -4.32987400 |
| H | 2.54790200 | 1.68691000 | -4.87039700 |
| H | -0.76752700 | 4.00242000 | -3.46061500 |
| H | 0.58537000 | 3.15061100 | -5.34880600 |
| C | -0.49697100 | 3.54413400 | -0.86127400 |
| H | -0.82868300 | 4.56354000 | -1.08080000 |
| H | -1.40434700 | 2.92454000 | -0.87705000 |

89

e-8-1 coplanar T1

| | | | |
|---|---|---|---|
| Au | 0.52775000 | -0.27908200 | 0.04165300 |
| N | 2.54986900 | -0.13327200 | -0.20589400 |
| C | 4.80482600 | -0.54317700 | -0.37043300 |
| C | -1.48087100 | 2.81482600 | -0.80676900 |
| N | -2.11144400 | 0.89697400 | 0.56231000 |
| C | -3.50614000 | 0.69788700 | 0.99051700 |
| C | -0.92254700 | 4.08627900 | -0.91592800 |
| H | -0.93975200 | 4.58792000 | -1.87766200 |
| C | 4.47102600 | -3.25025600 | 0.20768200 |
| H | 4.37370400 | -4.30293700 | 0.43622300 |
| C | -1.41587800 | -0.26334500 | 0.31220600 |
| C | 3.17324300 | 1.05416600 | -0.48654300 |

| | | | |
|---|---|---|---|
| C | -0.84669800 | 1.80782800 | -3.00151700 |
| H | -0.09759300 | 1.19036300 | -2.50003800 |
| H | -0.36589000 | 2.72444400 | -3.35788500 |
| H | -1.21763200 | 1.25439400 | -3.86819300 |
| C | -0.80606000 | 2.75222700 | 1.53814500 |
| C | -0.34424800 | 4.70933200 | 0.18035200 |
| H | 0.06853900 | 5.70688100 | 0.08816600 |
| C | 3.51928600 | -1.09333200 | -0.12287100 |
| C | -1.47899100 | 2.17176100 | 0.44678400 |
| C | -2.39407000 | -1.41927600 | 0.21310400 |
| C | 0.96802500 | 1.82842500 | 3.03263700 |
| H | 1.41511800 | 1.30923700 | 2.18305500 |
| H | 1.16070600 | 1.24033300 | 3.93254100 |
| H | 1.46668800 | 2.79488500 | 3.14759300 |
| C | 5.37650000 | 1.96312900 | -0.88484600 |
| H | 6.44999700 | 1.86025000 | -0.98507000 |
| C | 2.56567100 | 2.30414500 | -0.63017100 |
| H | 1.49158400 | 2.41431700 | -0.52263300 |
| C | -2.00105300 | 2.13559700 | -2.05354200 |
| H | -2.44235100 | 1.18756100 | -1.75177300 |
| C | -0.53920200 | 2.01800600 | 2.83806700 |
| H | -0.98663200 | 1.02458500 | 2.76175000 |
| C | 4.57507500 | 0.87906000 | -0.60772100 |
| C | 3.33922800 | -2.44581600 | 0.17409200 |
| H | 2.34765500 | -2.83085100 | 0.38301800 |
| C | -3.71322400 | -0.81867300 | 0.76248300 |
| H | -4.54310100 | -0.99979500 | 0.08120800 |
| H | -3.99349000 | -1.29399700 | 1.69993600 |
| C | 5.91415600 | -1.35645100 | -0.33473500 |
| H | 6.90653900 | -0.96464000 | -0.52073900 |
| C | -3.05489200 | 2.96459400 | -2.78438100 |
| H | -3.86103700 | 3.27990400 | -2.11981400 |
| H | -3.48696800 | 2.38039900 | -3.60123600 |
| H | -2.61146300 | 3.86259700 | -3.22281000 |

| | | | |
|---|---|---|---|
| C | 3.39198500 | 3.38670000 | -0.90465900 |
| H | 2.95608600 | 4.37061100 | -1.01847000 |
| C | -0.26975100 | 4.03268500 | 1.38633900 |
| H | 0.23080600 | 4.49497400 | 2.23016900 |
| C | 5.73478500 | -2.71854300 | -0.04571400 |
| H | 6.59870600 | -3.36944700 | -0.01429500 |
| C | -1.09415400 | 2.75260500 | 4.05897400 |
| H | -2.15862100 | 2.96793100 | 3.97551000 |
| H | -0.57277000 | 3.70346800 | 4.19786600 |
| H | -0.93411900 | 2.15763100 | 4.96072600 |
| C | -4.47193500 | 1.56974100 | 0.19283700 |
| H | -4.21639400 | 2.62554600 | 0.31846000 |
| H | -5.48875300 | 1.42032700 | 0.56174400 |
| H | -4.45547400 | 1.34347200 | -0.87194200 |
| C | 4.77019000 | 3.22125200 | -1.03292000 |
| H | 5.38952700 | 4.08211800 | -1.24862600 |
| C | -3.69499000 | 1.04624200 | 2.46507000 |
| H | -3.01775200 | 0.46956300 | 3.09781700 |
| H | -4.72203000 | 0.82653200 | 2.76611700 |
| H | -3.51325000 | 2.11032800 | 2.62658800 |
| C | -1.82729300 | -2.56160100 | 1.05179500 |
| C | -2.00474800 | -2.59538000 | 2.43748700 |
| C | -0.99533900 | -3.52180500 | 0.47447600 |
| C | -1.42152800 | -3.57324900 | 3.22590100 |
| H | -2.59101100 | -1.82462000 | 2.92299100 |
| C | -0.41063200 | -4.50688900 | 1.27209400 |
| C | -0.62051600 | -4.54735900 | 2.63939200 |
| H | -1.58611400 | -3.57087100 | 4.29621000 |
| H | 0.21856400 | -5.25314500 | 0.79629100 |
| H | -0.16489100 | -5.32300100 | 3.24201500 |
| C | -2.50771000 | -1.79849700 | -1.26595900 |
| C | -3.40366300 | -1.15184500 | -2.12557000 |
| C | -1.65467700 | -2.75423800 | -1.82836000 |
| C | -3.43445600 | -1.40781000 | -3.48745600 |

| | | | |
|---|---|---|---|
| H | -4.09506000 | -0.41913000 | -1.73369300 |
| C | -1.70189300 | -3.02315900 | -3.19318100 |
| C | -2.57507800 | -2.35231900 | -4.03522300 |
| H | -4.14048800 | -0.87776900 | -4.11570000 |
| H | -1.03309000 | -3.77780800 | -3.59480200 |
| H | -2.59619000 | -2.57241300 | -5.09489700 |
| C | -0.64913200 | -3.47186300 | -0.98386600 |
| H | -0.49456500 | -4.48437100 | -1.36754000 |
| H | 0.31528400 | -2.95380400 | -1.09205200 |

81

e-12-1 coplanar S1

| | | | |
|---|---|---|---|
| Au | 0.70209400 | 0.53262400 | -0.24661900 |
| N | 2.49519400 | 1.59947400 | -0.04981700 |
| C | 4.19329600 | 3.13710500 | -0.20942200 |
| C | 0.85038700 | -2.83315800 | 1.47259500 |
| N | -0.82955200 | -1.97127300 | -0.08664200 |
| C | -2.11094200 | -2.67748100 | -0.26998500 |
| C | 2.05728600 | -3.49992500 | 1.69012900 |
| H | 2.38865000 | -3.67117400 | 2.70879700 |
| C | 2.45991700 | 5.12011100 | -1.13274400 |
| H | 1.81211500 | 5.90782800 | -1.49375100 |
| C | -0.88685300 | -0.65552800 | -0.47418100 |
| C | 3.64720800 | 0.98552700 | 0.32668000 |
| C | 0.88164000 | -1.17320200 | 3.33125800 |
| H | 1.09001000 | -0.36903000 | 2.61950500 |
| H | 1.83709300 | -1.54609200 | 3.71254700 |
| H | 0.32103300 | -0.75451300 | 4.17079200 |
| C | 1.22268300 | -3.01681100 | -0.93312900 |
| C | 2.83929500 | -3.92420300 | 0.62960000 |
| H | 3.77137800 | -4.44583900 | 0.81141000 |
| C | 2.80418200 | 2.87915500 | -0.39000500 |
| C | 0.41875600 | -2.62047500 | 0.15422400 |
| C | -2.24325500 | -0.35609000 | -1.06927800 |
| C | 1.77940100 | -1.72799600 | -3.01022100 |

| | | | |
|---|---|---|---|
| H | 1.73876000 | -0.77929600 | -2.47178700 |
| H | 1.50103500 | -1.55036300 | -4.05193100 |
| H | 2.81115600 | -2.09125200 | -2.99345900 |
| C | 6.00672200 | 1.43824000 | 0.62316900 |
| H | 6.86634800 | 2.09678700 | 0.59128900 |
| C | 3.79919100 | -0.35024900 | 0.72298900 |
| H | 2.94638500 | -1.01989300 | 0.73972300 |
| C | 0.08839600 | -2.29602800 | 2.66222800 |
| H | -0.83916500 | -1.86374500 | 2.29049700 |
| C | 0.84561900 | -2.76128700 | -2.37888900 |
| H | -0.16853500 | -2.35377200 | -2.39651900 |
| C | 4.75283000 | 1.88105300 | 0.26681600 |
| C | 1.92985700 | 3.86760000 | -0.85405400 |
| H | 0.87941500 | 3.64119200 | -0.98635000 |
| C | -2.77959300 | -1.78935100 | -1.32545500 |
| H | -3.86241800 | -1.88075200 | -1.27234300 |
| H | -2.46159100 | -2.10549700 | -2.32461900 |
| C | 4.69758100 | 4.38718700 | -0.49155800 |
| H | 5.74962000 | 4.61084400 | -0.36192100 |
| C | -0.25107100 | -3.39159800 | 3.66975300 |
| H | -0.78174900 | -4.22043400 | 3.19552900 |
| H | -0.87834800 | -2.99249000 | 4.46946700 |
| H | 0.65378600 | -3.79679900 | 4.13000800 |
| C | 5.07312000 | -0.77371600 | 1.07495700 |
| H | 5.22348900 | -1.80035800 | 1.38369100 |
| C | 2.42546100 | -3.66829900 | -0.66996400 |
| H | 3.04555100 | -3.98396500 | -1.50164400 |
| C | 3.81918600 | 5.37876100 | -0.95552200 |
| H | 4.20561200 | 6.36395400 | -1.18161000 |
| C | 0.88323800 | -4.04367000 | -3.21380900 |
| H | 0.33010100 | -4.86000600 | -2.74687200 |
| H | 1.91269200 | -4.38138900 | -3.35513300 |
| H | 0.46269200 | -3.85989400 | -4.20457500 |
| C | -2.93350000 | -2.69161200 | 1.01876900 |

| | | | |
|---|---|---|---|
| H | -2.46145000 | -3.32385100 | 1.77381400 |
| H | -3.92910200 | -3.09219200 | 0.81357900 |
| H | -3.04104400 | -1.68535700 | 1.42662800 |
| C | 6.15829100 | 0.10223500 | 1.03090900 |
| H | 7.14056900 | -0.25458700 | 1.31216400 |
| C | -1.92543300 | -4.10591700 | -0.75080600 |
| H | -1.40271000 | -4.13913400 | -1.70593000 |
| H | -2.90459000 | -4.57156100 | -0.87803300 |
| H | -1.36110500 | -4.69212200 | -0.02096300 |
| C | -3.27385900 | 0.48774800 | -0.26867200 |
| C | -4.52174600 | 0.80646200 | -0.84701200 |
| C | -3.08493000 | 1.06811600 | 0.99606900 |
| C | -5.44293200 | 1.67673300 | -0.28561700 |
| C | -3.98686500 | 1.94961700 | 1.58320300 |
| C | -5.15686900 | 2.26826000 | 0.92911400 |
| H | -6.37497600 | 1.87676500 | -0.79530000 |
| H | -3.76505700 | 2.36466900 | 2.55636600 |
| C | -1.92724800 | 0.43627600 | -2.35940000 |
| H | -1.36194200 | 1.32851500 | -2.08042100 |
| H | -1.28458900 | -0.17510400 | -2.99761100 |
| H | -2.80249900 | 0.74213200 | -2.92698400 |
| Cl | -5.08573000 | 0.09157500 | -2.33002000 |
| Cl | -1.74599500 | 0.70124000 | 2.03006200 |
| Cl | -6.27311300 | 3.37021700 | 1.63663900 |

81

e-12-1 rotated S1

| | | | |
|---|---|---|---|
| Au | 0.84565600 | 0.19911500 | -0.36611100 |
| N | 2.59270300 | 1.28235700 | -0.38911500 |
| C | 4.18163600 | 2.83481000 | 0.22394500 |
| C | 0.53771600 | -2.52245100 | 2.23169500 |
| N | -1.03658000 | -1.95625000 | 0.45099900 |
| C | -2.41308700 | -2.46748200 | 0.40529300 |
| C | 1.65983300 | -3.22933200 | 2.66192000 |
| H | 2.00397500 | -3.10455100 | 3.68326700 |

| | | | |
|---|---|---|---|
| C | 2.55963900 | 3.89973200 | 2.21775500 |
| H | 1.94777100 | 4.33412800 | 2.99656500 |
| C | -0.87105000 | -0.78789600 | -0.27869600 |
| C | 3.68468400 | 1.11762800 | -1.20151500 |
| C | 0.79639400 | -0.41505600 | 3.55382000 |
| H | 1.15351400 | 0.09159500 | 2.65285800 |
| H | 1.66697500 | -0.78007700 | 4.10578600 |
| H | 0.27849700 | 0.31375600 | 4.18340500 |
| C | 0.79093600 | -3.52910800 | 0.02455100 |
| C | 2.34258900 | -4.07338800 | 1.80320000 |
| H | 3.21328700 | -4.61673000 | 2.14941700 |
| C | 2.88370700 | 2.31566000 | 0.46618200 |
| C | 0.09366300 | -2.68786900 | 0.91203100 |
| C | -2.14961500 | -0.49604900 | -1.04079400 |
| C | 1.39100100 | -3.04546900 | -2.36466300 |
| H | 1.42384400 | -1.97169800 | -2.17213600 |
| H | 1.10520700 | -3.20738000 | -3.40727200 |
| H | 2.39043600 | -3.46433200 | -2.21437300 |
| C | 5.89223400 | 2.03997700 | -1.58453500 |
| H | 6.68736600 | 2.73815300 | -1.35363300 |
| C | 3.84228400 | 0.18894700 | -2.23172300 |
| H | 3.04817200 | -0.50744900 | -2.46131300 |
| C | -0.13990200 | -1.56432000 | 3.18397900 |
| H | -0.99365900 | -1.13635400 | 2.66073100 |
| C | 0.38592400 | -3.70897100 | -1.42526100 |
| H | -0.57302300 | -3.20671800 | -1.57007000 |
| C | 4.71076600 | 2.04414500 | -0.87922800 |
| C | 2.06065900 | 2.84680000 | 1.46145700 |
| H | 1.06776100 | 2.44618300 | 1.61821500 |
| C | -2.91671900 | -1.83842200 | -0.89775500 |
| H | -3.99977100 | -1.73404700 | -0.87906600 |
| H | -2.65608300 | -2.48215800 | -1.74426900 |
| C | 4.66110000 | 3.87828500 | 0.98223600 |
| H | 5.64938100 | 4.28830400 | 0.81422600 |

| | | | |
|---|---|---|---|
| C | -0.64097300 | -2.27581000 | 4.43855300 |
| H | -1.29732600 | -3.11186000 | 4.18614800 |
| H | -1.19369400 | -1.58368600 | 5.07772400 |
| H | 0.19382000 | -2.67411700 | 5.02144300 |
| C | 5.03983700 | 0.19829000 | -2.93501000 |
| H | 5.19264700 | -0.50866800 | -3.73911800 |
| C | 1.90904700 | -4.21399100 | 0.49323400 |
| H | 2.45081500 | -4.86688400 | -0.18274800 |
| C | 3.83674700 | 4.40725100 | 1.98647200 |
| H | 4.19956700 | 5.22785500 | 2.59133100 |
| C | 0.23999500 | -5.18534000 | -1.79845400 |
| H | -0.37864100 | -5.73071700 | -1.08395000 |
| H | 1.21676900 | -5.67410200 | -1.82942900 |
| H | -0.20482200 | -5.28308500 | -2.79108400 |
| C | -3.23518000 | -1.96062200 | 1.59259600 |
| H | -2.87189000 | -2.39586000 | 2.52639400 |
| H | -4.28259100 | -2.24598400 | 1.46785900 |
| H | -3.17803200 | -0.87459600 | 1.67346000 |
| C | 6.04922500 | 1.10579700 | -2.61920700 |
| H | 6.97268100 | 1.09039000 | -3.18296700 |
| C | -2.48604400 | -3.98537900 | 0.38999100 |
| H | -2.00652300 | -4.40751400 | -0.49161100 |
| H | -3.53332500 | -4.29350900 | 0.38977600 |
| H | -2.00964100 | -4.40252900 | 1.28123900 |
| C | -3.04915900 | 0.69533100 | -0.60495600 |
| C | -4.20705200 | 1.00882500 | -1.35194000 |
| C | -2.81952200 | 1.59374900 | 0.45112900 |
| C | -4.98512400 | 2.13900100 | -1.15534700 |
| C | -3.57791500 | 2.73963200 | 0.67082900 |
| C | -4.64687700 | 3.02260200 | -0.15016700 |
| H | -5.85027800 | 2.31204700 | -1.77975300 |
| H | -3.32988600 | 3.38813400 | 1.49920000 |
| C | -1.68666600 | -0.19702600 | -2.48568300 |
| H | -1.00127700 | 0.65286500 | -2.45802800 |

| | | | |
|---|---|---|---|
| H | -1.12738800 | -1.06111400 | -2.85309000 |
| H | -2.48862700 | 0.02792400 | -3.18396600 |
| Cl | -4.84504300 | -0.03366900 | -2.59085600 |
| Cl | -1.62850300 | 1.35527500 | 1.69048000 |
| Cl | -5.58139900 | 4.44584500 | 0.10112900 |

81

e-12-1 coplanar T1

| | | | |
|---|---|---|---|
| Au | 0.72985700 | 0.50692800 | -0.34399600 |
| N | 2.46355200 | 1.58548300 | -0.22161500 |
| C | 4.07138300 | 3.22912900 | -0.22180100 |
| C | 0.93254700 | -2.68376000 | 1.62352800 |
| N | -0.77733600 | -1.98427500 | 0.01948700 |
| C | -2.05170400 | -2.70120800 | -0.11006300 |
| C | 2.14102100 | -3.33060900 | 1.88674600 |
| H | 2.48716000 | -3.40505100 | 2.91212300 |
| C | 2.22672100 | 5.19338500 | -0.93815500 |
| H | 1.53378700 | 5.97651500 | -1.21457600 |
| C | -0.86269100 | -0.66401700 | -0.40356000 |
| C | 3.66010400 | 1.00712500 | 0.11621100 |
| C | 0.98338100 | -0.87024000 | 3.33473800 |
| H | 1.20071700 | -0.13588300 | 2.55368200 |
| H | 1.93315500 | -1.21532300 | 3.75418200 |
| H | 0.42290800 | -0.37209200 | 4.12993800 |
| C | 1.26732900 | -3.09679900 | -0.75656600 |
| C | 2.90081900 | -3.86768200 | 0.86121600 |
| H | 3.83028900 | -4.38028200 | 1.07942400 |
| C | 2.70050000 | 2.91587800 | -0.42410400 |
| C | 0.47577800 | -2.59996800 | 0.29823900 |
| C | -2.22080800 | -0.42733300 | -1.03364400 |
| C | 1.83599500 | -1.97890700 | -2.92231000 |
| H | 1.81766300 | -0.99500400 | -2.44932400 |
| H | 1.55377100 | -1.86542600 | -3.97189200 |
| H | 2.86034200 | -2.36200200 | -2.88770900 |
| C | 5.98496500 | 1.57139700 | 0.45753200 |

| | | | |
|---|---|---|---|
| H | 6.80345900 | 2.28031700 | 0.48142500 |
| C | 3.88156400 | -0.33892600 | 0.41289000 |
| H | 3.06477700 | -1.05156500 | 0.38982900 |
| C | 0.18208100 | -2.04208000 | 2.76750700 |
| H | -0.74653800 | -1.64099500 | 2.36469100 |
| C | 0.88576600 | -2.94697200 | -2.21561400 |
| H | -0.12056800 | -2.52199800 | -2.25702900 |
| C | 4.70516500 | 1.96449600 | 0.13705000 |
| C | 1.76860100 | 3.89185000 | -0.78231700 |
| H | 0.73033700 | 3.62222900 | -0.92581500 |
| C | -2.73153100 | -1.88251500 | -1.21175900 |
| H | -3.81249500 | -1.99191800 | -1.15645700 |
| H | -2.40415800 | -2.24712700 | -2.19128000 |
| C | 4.50760400 | 4.52426900 | -0.38104400 |
| H | 5.54684900 | 4.79062800 | -0.23290100 |
| C | -0.15199700 | -3.04567200 | 3.86868200 |
| H | -0.68829600 | -3.91090900 | 3.47272300 |
| H | -0.77204500 | -2.57694200 | 4.63568500 |
| H | 0.75605200 | -3.41184300 | 4.35497800 |
| C | 5.17910200 | -0.71669800 | 0.73477900 |
| H | 5.38517800 | -1.75259300 | 0.97076900 |
| C | 2.46905300 | -3.73361100 | -0.45044500 |
| H | 3.07507000 | -4.13075500 | -1.25750900 |
| C | 3.57135100 | 5.50590400 | -0.74225400 |
| H | 3.90137400 | 6.52839700 | -0.87078000 |
| C | 0.90188400 | -4.28664000 | -2.95546800 |
| H | 0.34164800 | -5.06008400 | -2.42833700 |
| H | 1.92667800 | -4.64643500 | -3.07609100 |
| H | 0.47875600 | -4.17052200 | -3.95536500 |
| C | -2.86074700 | -2.63418700 | 1.18678200 |
| H | -2.37329100 | -3.21431900 | 1.97367400 |
| H | -3.85950000 | -3.04740400 | 1.02634600 |
| H | -2.96089800 | -1.60343800 | 1.53106000 |
| C | 6.21247600 | 0.21868400 | 0.75715500 |

| | | | |
|---|---|---|---|
| H | 7.21391300 | -0.10327600 | 1.01145100 |
| C | -1.87423900 | -4.15739700 | -0.50204000 |
| H | -1.37598600 | -4.25089000 | -1.46601700 |
| H | -2.85398900 | -4.63360100 | -0.57407700 |
| H | -1.28933600 | -4.69405700 | 0.24954900 |
| C | -3.28023900 | 0.43378100 | -0.28882700 |
| C | -4.54083400 | 0.66388800 | -0.88366800 |
| C | -3.11925500 | 1.10223600 | 0.93732100 |
| C | -5.50665300 | 1.51573100 | -0.37023000 |
| C | -4.06751200 | 1.96893900 | 1.47376100 |
| C | -5.25379800 | 2.18633600 | 0.80935400 |
| H | -6.44506900 | 1.64186000 | -0.89147800 |
| H | -3.86574600 | 2.45314400 | 2.41884900 |
| C | -1.92199900 | 0.29657400 | -2.36604600 |
| H | -1.38369200 | 1.21981200 | -2.13923400 |
| H | -1.25642400 | -0.33360500 | -2.96102800 |
| H | -2.80079100 | 0.54392200 | -2.95640900 |
| Cl | -5.06529000 | -0.13669200 | -2.33736400 |
| Cl | -1.76138500 | 0.89043000 | 1.99112300 |
| Cl | -6.42769900 | 3.26428800 | 1.45930300 |

88

e-18-1 coplanar S1

| | | | |
|---|---|---|---|
| Au | 0.76750000 | 0.36271500 | -0.13727600 |
| N | 2.77399200 | 0.78162000 | 0.26865600 |
| C | 4.71842100 | 1.74877600 | 1.01537000 |
| C | -0.15852100 | -3.17925600 | 0.40701200 |
| N | -1.28325100 | -1.49622000 | -0.98639100 |
| C | -2.64936600 | -1.77887400 | -1.46877300 |
| C | 0.73714200 | -4.24271900 | 0.49697200 |
| H | 0.83914800 | -4.76282600 | 1.44309200 |
| C | 3.62166600 | 4.31185800 | 0.89239800 |
| H | 3.22170600 | 5.31648400 | 0.85982100 |
| C | -1.10579300 | -0.26506500 | -0.42345400 |
| C | 3.66894600 | -0.21542700 | 0.50686800 |

| | | | |
|---|---|---|---|
| C | 0.13090000 | -2.16570700 | 2.66622900 |
| H | 0.64492500 | -1.29860900 | 2.24277400 |
| H | 0.88107600 | -2.91520200 | 2.93479100 |
| H | -0.38056400 | -1.85270500 | 3.57985000 |
| C | 0.53759500 | -2.85282900 | -1.91409800 |
| C | 1.49717200 | -4.64201400 | -0.59301800 |
| H | 2.17201300 | -5.48532300 | -0.50829700 |
| C | 3.37569300 | 1.95899700 | 0.59243500 |
| C | -0.30204200 | -2.52530700 | -0.83337700 |
| C | -2.43900400 | 0.47244900 | -0.42732800 |
| C | 1.98704400 | -1.31549800 | -3.23539800 |
| H | 2.15357000 | -0.68407400 | -2.36015500 |
| H | 2.05000700 | -0.68827000 | -4.12738400 |
| H | 2.79201800 | -2.05496100 | -3.28258700 |
| C | 5.95406900 | -0.55225400 | 1.23071800 |
| H | 6.91477200 | -0.18366500 | 1.56942900 |
| C | 3.46158700 | -1.59165600 | 0.34296300 |
| H | 2.50088000 | -1.96895300 | 0.00976600 |
| C | -0.87671200 | -2.74209900 | 1.66935600 |
| H | -1.56999900 | -1.93954400 | 1.41583000 |
| C | 0.62275700 | -2.00843500 | -3.17039300 |
| H | -0.13748900 | -1.22897900 | -3.10282500 |
| C | 4.91335000 | 0.30717300 | 0.95864500 |
| C | 2.81596400 | 3.23922100 | 0.53460300 |
| H | 1.78460400 | 3.36535500 | 0.22898200 |
| C | -3.26689300 | -0.37312900 | -1.42989600 |
| H | -4.33231200 | -0.38674600 | -1.23033100 |
| H | -3.12502000 | 0.07417300 | -2.41341800 |
| C | 5.50016400 | 2.82713200 | 1.36546300 |
| H | 6.52619300 | 2.69656800 | 1.68752100 |
| C | -1.65855400 | -3.87851000 | 2.32727100 |
| H | -2.33145800 | -4.37333500 | 1.62560500 |
| H | -2.25212900 | -3.48560800 | 3.15688200 |
| H | -0.98282100 | -4.63454900 | 2.73517000 |

| | | | |
|---|---|---|---|
| C | 4.52319200 | -2.43851100 | 0.62812200 |
| H | 4.39381400 | -3.50694700 | 0.51207900 |
| C | 1.41236200 | -3.93150300 | -1.77851200 |
| H | 2.05133400 | -4.19834000 | -2.61318300 |
| C | 4.94080000 | 4.11276200 | 1.29979800 |
| H | 5.54532600 | 4.96693500 | 1.57540200 |
| C | 0.41718200 | -2.81971300 | -4.44899800 |
| H | -0.51762200 | -3.37985400 | -4.44269300 |
| H | 1.23390800 | -3.53330100 | -4.58557700 |
| H | 0.41495100 | -2.15770500 | -5.31743200 |
| C | -3.34252000 | -2.81610700 | -0.58719900 |
| H | -2.82039800 | -3.77262300 | -0.67770200 |
| H | -4.37316300 | -2.95267600 | -0.91924800 |
| H | -3.35413900 | -2.53561400 | 0.46281700 |
| C | 5.74890700 | -1.93148600 | 1.06154400 |
| H | 6.56065000 | -2.61491100 | 1.27429600 |
| C | -2.65424400 | -2.31987800 | -2.89339500 |
| H | -2.18356800 | -1.62198300 | -3.58693400 |
| H | -3.68625200 | -2.48285600 | -3.21211800 |
| H | -2.13268900 | -3.27849800 | -2.93648700 |
| C | -2.87480000 | 0.37224600 | 1.04628000 |
| C | -3.95365300 | -0.34372600 | 1.56837400 |
| C | -2.06716500 | 0.94226200 | 2.04460900 |
| C | -4.11392000 | -0.65110800 | 2.91468500 |
| C | -2.18527400 | 0.70764200 | 3.39446200 |
| C | -3.20450500 | -0.13879900 | 3.80851200 |
| H | -4.95533600 | -1.24742700 | 3.23769900 |
| H | -1.50924500 | 1.17408600 | 4.09582700 |
| C | -2.32235100 | 1.90021600 | -0.94753500 |
| C | -1.60770000 | 2.16563000 | -2.12105400 |
| C | -2.89630600 | 3.03209800 | -0.37224400 |
| C | -1.41218000 | 3.42390400 | -2.65581000 |
| C | -2.73768600 | 4.32151800 | -0.85391200 |
| C | -1.98189800 | 4.49368000 | -1.99280100 |

| | | | |
|---|---|---|---|
| H | -0.84124000 | 3.55352200 | -3.56349600 |
| H | -3.20351900 | 5.15471400 | -0.34818000 |
| F | -1.80946400 | 5.72548800 | -2.48101300 |
| F | -1.09243800 | 1.14223400 | -2.81528000 |
| F | -3.66706800 | 2.90701500 | 0.71379400 |
| F | -4.94829800 | -0.77193300 | 0.76969200 |
| F | -1.12249000 | 1.81167300 | 1.66837800 |
| F | -3.32719800 | -0.41845000 | 5.11069100 |

88

e-18-1 rotated S1

| | | | |
|---|---|---|---|
| Au | 0.83678200 | -0.08940600 | 0.11801700 |
| N | 2.78831000 | -0.20881500 | 0.72801200 |
| C | 5.07893200 | 0.02337400 | 0.68346400 |
| C | -1.61077700 | -3.08743200 | -0.27253700 |
| N | -1.59836300 | -0.88754600 | -1.34885200 |
| C | -2.80930900 | -0.46298600 | -2.06861900 |
| C | -1.26434500 | -4.43562100 | -0.31182300 |
| H | -1.61305300 | -5.09235900 | 0.47793800 |
| C | 5.03297200 | 1.26057000 | -1.80961800 |
| H | 5.04465400 | 1.74470100 | -2.77672400 |
| C | -1.01784200 | 0.07420800 | -0.54750300 |
| C | 3.28931700 | -0.73151100 | 1.89064400 |
| C | -1.49723000 | -2.62538300 | 2.17309900 |
| H | -0.62060200 | -1.98698300 | 2.03415300 |
| H | -1.16055700 | -3.64752100 | 2.36752600 |
| H | -2.05128400 | -2.27321200 | 3.04689700 |
| C | -0.29418200 | -2.73375200 | -2.29835200 |
| C | -0.47979000 | -4.94676000 | -1.33433500 |
| H | -0.23259400 | -6.00104800 | -1.35698100 |
| C | 3.85722800 | 0.24965200 | -0.00058100 |
| C | -1.16982000 | -2.24658600 | -1.31192500 |
| C | -1.95751100 | 1.28225400 | -0.51156000 |
| C | 1.92568200 | -1.84249200 | -2.98832600 |
| H | 2.12961500 | -1.61474700 | -1.94037800 |

| | | | |
|---|---|---|---|
| H | 2.44243500 | -1.10851800 | -3.61247600 |
| H | 2.34675300 | -2.82710100 | -3.21009200 |
| C | 5.39651000 | -1.09640100 | 3.02571300 |
| H | 6.47585900 | -1.02658500 | 3.08084700 |
| C | 2.56741100 | -1.30594400 | 2.93822000 |
| H | 1.48921600 | -1.37291900 | 2.88074200 |
| C | -2.38256400 | -2.57414900 | 0.92725300 |
| H | -2.62672500 | -1.52580500 | 0.75549200 |
| C | 0.42157200 | -1.82299000 | -3.27664800 |
| H | 0.06462100 | -0.80471100 | -3.10719800 |
| C | 4.70405000 | -0.62506500 | 1.93406100 |
| C | 3.82241200 | 0.87191600 | -1.25039600 |
| H | 2.87430600 | 1.03488900 | -1.74849200 |
| C | -2.79938900 | 1.04843800 | -1.79314100 |
| H | -3.79352200 | 1.47949000 | -1.76352200 |
| H | -2.26336000 | 1.52455900 | -2.61333000 |
| C | 6.26963500 | 0.41504200 | 0.11486500 |
| H | 7.21448000 | 0.25253300 | 0.61862300 |
| C | -3.67526500 | -3.34795000 | 1.18012700 |
| H | -4.29974700 | -3.40220700 | 0.28737200 |
| H | -4.24888300 | -2.86212900 | 1.97408500 |
| H | -3.46378400 | -4.36996900 | 1.50482100 |
| C | 3.28115800 | -1.77601400 | 4.03266000 |
| H | 2.75356500 | -2.22752800 | 4.86174000 |
| C | 0.01827000 | -4.09258000 | -2.30347900 |
| H | 0.68139100 | -4.47845400 | -3.07033300 |
| C | 6.23603900 | 1.03657200 | -1.14210200 |
| H | 7.16399700 | 1.35006200 | -1.60197200 |
| C | 0.19964200 | -2.21570000 | -4.73679300 |
| H | -0.85518600 | -2.24674600 | -5.00673000 |
| H | 0.62415700 | -3.20380900 | -4.93318500 |
| H | 0.70238700 | -1.50684400 | -5.39871400 |
| C | -4.05460200 | -1.20697700 | -1.58788300 |
| H | -3.94754600 | -2.27013100 | -1.82080300 |

| | | | |
|---|---|---|---|
| H | -4.93578700 | -0.82807800 | -2.10894900 |
| H | -4.22205000 | -1.11279000 | -0.51834500 |
| C | 4.67069900 | -1.67490100 | 4.07798300 |
| H | 5.20172900 | -2.05006600 | 4.94289000 |
| C | -2.69445700 | -0.71503300 | -3.56876500 |
| H | -1.82378000 | -0.20876700 | -3.98782300 |
| H | -3.58971700 | -0.34093400 | -4.07102300 |
| H | -2.61792400 | -1.78655900 | -3.76677200 |
| C | -2.74009600 | 1.13533600 | 0.80860500 |
| C | -4.10704300 | 0.91174300 | 0.97122300 |
| C | -2.04066300 | 1.10496400 | 2.02469400 |
| C | -4.70215500 | 0.49751300 | 2.15808000 |
| C | -2.56818600 | 0.73539700 | 3.24089200 |
| C | -3.91026400 | 0.39162900 | 3.27570400 |
| H | -5.76427600 | 0.30143700 | 2.19121500 |
| H | -1.95258100 | 0.72570200 | 4.12827700 |
| C | -1.20477600 | 2.60465400 | -0.63950900 |
| C | -0.21923900 | 2.77346100 | -1.62147500 |
| C | -1.41147900 | 3.74396300 | 0.13719600 |
| C | 0.53819300 | 3.91700000 | -1.78860700 |
| C | -0.68573800 | 4.91939000 | 0.02407300 |
| C | 0.29338900 | 4.97938100 | -0.94131300 |
| H | 1.28262900 | 3.97210200 | -2.56949200 |
| H | -0.89772200 | 5.75515400 | 0.67476900 |
| F | 1.01189200 | 6.09771400 | -1.07741500 |
| F | 0.01385400 | 1.79393600 | -2.50728300 |
| F | -2.37585100 | 3.75385800 | 1.06470300 |
| F | -4.96750400 | 1.11278800 | -0.04167400 |
| F | -0.76469500 | 1.50470400 | 2.02834500 |
| F | -4.44934100 | -0.00609500 | 4.43320200 |

88

e-18-1 coplanar T1

| | | | |
|---|---|---|---|
| Au | 0.76566700 | 0.35585400 | -0.04712400 |
| N | 2.69679100 | 0.80059500 | 0.44277400 |

| | | | |
|---|---|---|---|
| C | 4.66122600 | 1.85318200 | 1.00408900 |
| C | -0.10291200 | -3.19838200 | 0.40121400 |
| N | -1.22686000 | -1.52943000 | -1.00769200 |
| C | -2.57316100 | -1.82558300 | -1.52144000 |
| C | 0.81347300 | -4.24317800 | 0.50775400 |
| H | 0.89856000 | -4.77223200 | 1.45076500 |
| C | 3.45404600 | 4.36703900 | 0.97764200 |
| H | 3.01310000 | 5.35463700 | 0.97650500 |
| C | -1.05827900 | -0.26227300 | -0.48625100 |
| C | 3.66633600 | -0.15981000 | 0.57728700 |
| C | 0.09781100 | -2.22242400 | 2.68863200 |
| H | 0.62928500 | -1.35114800 | 2.29658600 |
| H | 0.83435800 | -2.97610300 | 2.98208600 |
| H | -0.45142600 | -1.91777400 | 3.58307900 |
| C | 0.65174200 | -2.83098500 | -1.89272300 |
| C | 1.61370500 | -4.61313500 | -0.56359800 |
| H | 2.30358500 | -5.44323700 | -0.46863300 |
| C | 3.28224300 | 2.00811500 | 0.70113000 |
| C | -0.22889100 | -2.53414600 | -0.83571100 |
| C | -2.41706300 | 0.43312600 | -0.47729700 |
| C | 2.10040600 | -1.25749100 | -3.17304600 |
| H | 2.23655500 | -0.63181800 | -2.28857400 |
| H | 2.16844900 | -0.61973200 | -4.05721200 |
| H | 2.92006700 | -1.98115800 | -3.21289700 |
| C | 6.01588600 | -0.38950300 | 1.09343200 |
| H | 6.98236900 | 0.02331700 | 1.35519400 |
| C | 3.50778700 | -1.53681200 | 0.40890900 |
| H | 2.54135500 | -1.95510800 | 0.15009800 |
| C | -0.86765900 | -2.78726100 | 1.64458800 |
| H | -1.55537500 | -1.98473200 | 1.37520200 |
| C | 0.74793600 | -1.97497400 | -3.14024300 |
| H | -0.02768600 | -1.20993200 | -3.08079300 |
| C | 4.91442300 | 0.41810400 | 0.92053300 |
| C | 2.66637200 | 3.26100000 | 0.68652800 |

| | | | |
|---|---|---|---|
| H | 1.61137400 | 3.34340600 | 0.45706000 |
| C | -3.20846000 | -0.42609500 | -1.49890300 |
| H | -4.27921700 | -0.45110400 | -1.33017900 |
| H | -3.04372300 | 0.02138900 | -2.47878800 |
| C | 5.42599900 | 2.96082700 | 1.28962300 |
| H | 6.48001100 | 2.87294800 | 1.52251800 |
| C | -1.66132500 | -3.94066800 | 2.25764200 |
| H | -2.30458400 | -4.43173700 | 1.52666200 |
| H | -2.28666900 | -3.56725800 | 3.07289600 |
| H | -0.99090500 | -4.69584900 | 2.67620000 |
| C | 4.63051600 | -2.33525300 | 0.58621000 |
| H | 4.54250100 | -3.40669600 | 0.46169000 |
| C | 1.54781400 | -3.89050900 | -1.74318400 |
| H | 2.21513300 | -4.13505500 | -2.56255800 |
| C | 4.80885100 | 4.22163500 | 1.27360300 |
| H | 5.39887500 | 5.10073500 | 1.49723000 |
| C | 0.58357200 | -2.78037400 | -4.42859700 |
| H | -0.34134500 | -3.35630700 | -4.44541900 |
| H | 1.41525400 | -3.47909800 | -4.55226600 |
| H | 0.58799500 | -2.11255000 | -5.29266700 |
| C | -3.28155200 | -2.87604900 | -0.66800900 |
| H | -2.74675500 | -3.82624100 | -0.75350600 |
| H | -4.30185900 | -3.02196200 | -1.02728400 |
| H | -3.32209300 | -2.60641800 | 0.38403300 |
| C | 5.86196200 | -1.77441300 | 0.92187500 |
| H | 6.72029600 | -2.42008400 | 1.05434200 |
| C | -2.53805300 | -2.35907100 | -2.94997800 |
| H | -2.05399600 | -1.65283800 | -3.62583000 |
| H | -3.55897500 | -2.53004900 | -3.29923900 |
| H | -2.00563900 | -3.31233200 | -2.98422100 |
| C | -2.88965200 | 0.31573200 | 0.98447100 |
| C | -3.96721100 | -0.41916600 | 1.47689600 |
| C | -2.11997800 | 0.89656800 | 2.00460600 |
| C | -4.15734800 | -0.73461600 | 2.81806600 |

| | | | |
|---|---|---|---|
| C | -2.26569400 | 0.65330200 | 3.35076900 |
| C | -3.27971200 | -0.21039300 | 3.73600500 |
| H | -4.99538700 | -1.34799500 | 3.11667500 |
| H | -1.61555600 | 1.12841300 | 4.07053600 |
| C | -2.34120000 | 1.86966500 | -0.98642900 |
| C | -1.62238800 | 2.17333800 | -2.14936000 |
| C | -2.96407800 | 2.97608600 | -0.41163200 |
| C | -1.46636300 | 3.44503400 | -2.66767400 |
| C | -2.84722600 | 4.27568400 | -0.87806500 |
| C | -2.08278300 | 4.48707400 | -2.00406800 |
| H | -0.89034800 | 3.60310700 | -3.56764500 |
| H | -3.35148900 | 5.08513900 | -0.37069100 |
| F | -1.94857200 | 5.72952000 | -2.47843500 |
| F | -1.06481600 | 1.18340000 | -2.85687100 |
| F | -3.74715400 | 2.81633500 | 0.66179800 |
| F | -4.93382400 | -0.86345300 | 0.65472900 |
| F | -1.18813300 | 1.79267700 | 1.65789900 |
| F | -3.43083500 | -0.49679400 | 5.03360500 |

21

Carbazole fragment

| | | | |
|---|---|---|---|
| N | 0.00007100 | -1.69133800 | 0.00010400 |
| C | 0.73099100 | 0.51379400 | 0.00000100 |
| C | 3.40236100 | -0.25621400 | -0.00003900 |
| H | 4.45025500 | -0.52704200 | -0.00005800 |
| C | -1.09108900 | -0.86151200 | 0.00002800 |
| C | 1.09102500 | -0.86158500 | 0.00001900 |
| C | -1.70139500 | 1.49024700 | 0.00003500 |
| H | -1.44319800 | 2.54266800 | 0.00003700 |
| C | -2.43095000 | -1.25107400 | -0.00006500 |
| H | -2.68877800 | -2.30208600 | -0.00016600 |
| C | -0.73098300 | 0.51379600 | 0.00010000 |
| C | 2.43101700 | -1.25105700 | -0.00002200 |
| H | 2.68902000 | -2.30202900 | -0.00002000 |
| C | 1.70134800 | 1.49027700 | -0.00001800 |

| | | | |
|---|---|---|---|
| H | 1.44304000 | 2.54266600 | 0.00000700 |
| C | -3.40239000 | -0.25624700 | -0.00005400 |
| H | -4.45028600 | -0.52704100 | -0.00010300 |
| C | 3.04536400 | 1.09164500 | -0.00003300 |
| H | 3.82274400 | 1.84522100 | -0.00003700 |
| C | -3.04539000 | 1.09156900 | -0.00001000 |
| H | -3.82275200 | 1.84517100 | -0.00003200 |

22

Au-Carbazole fragment

| | | | |
|---|---|---|---|
| Au | -1.88698800 | -0.00020200 | -0.04737400 |
| N | 0.03709700 | -0.00010400 | 0.28714700 |
| C | 2.18124000 | -0.72254700 | -0.00757100 |
| C | 1.48617600 | -3.40990600 | 0.04991600 |
| H | 1.23294700 | -4.46266500 | 0.06387900 |
| C | 0.83590400 | 1.12328000 | 0.13995900 |
| C | 0.83619200 | -1.12315900 | 0.13998600 |
| C | 3.17968100 | 1.68969500 | -0.11037900 |
| H | 4.21790700 | 1.39544600 | -0.21245800 |
| C | 0.47739600 | 2.46763200 | 0.16437800 |
| H | -0.56261700 | 2.75884300 | 0.26442500 |
| C | 2.18103100 | 0.72302100 | -0.00755500 |
| C | 0.47822300 | -2.46769800 | 0.16439300 |
| H | -0.56166600 | -2.75934800 | 0.26445900 |
| C | 3.18023200 | -1.68883500 | -0.11041400 |
| H | 4.21831900 | -1.39409900 | -0.21250000 |
| C | 1.48501500 | 3.41022500 | 0.04992300 |
| H | 1.23147800 | 4.46290600 | 0.06388200 |
| C | 2.82768400 | -3.02775800 | -0.08333000 |
| H | 3.59239200 | -3.78914900 | -0.16761200 |
| C | 2.82664000 | 3.02848500 | -0.08330300 |
| H | 3.59110400 | 3.79013100 | -0.16756000 |

34

a-1-1 carbene fragment

| | | | |
|---|---|---|---|
| C | -1.82793800 | -0.97569600 | 0.83999900 |

| | | | |
|---|---|---|---|
| N | 0.19554900 | -0.09318900 | -0.18370900 |
| C | 0.82417200 | 1.14596200 | 0.38709600 |
| C | -3.20774000 | -1.12295900 | 0.86910600 |
| H | -3.67002000 | -1.70999900 | 1.65245900 |
| C | 0.97268900 | -1.08457200 | -0.54764900 |
| C | -2.01131500 | 0.36288900 | -1.15424200 |
| C | -3.99198200 | -0.52544300 | -0.11070900 |
| C | -1.23301900 | -0.22963700 | -0.16896300 |
| C | 2.29488700 | 0.88700100 | 0.05154200 |
| H | 2.94890100 | 1.17434800 | 0.87850500 |
| H | 2.58008700 | 1.48429000 | -0.81939700 |
| C | -3.39196900 | 0.21261700 | -1.12341900 |
| H | -3.99848600 | 0.66556100 | -1.89738900 |
| C | 0.54573600 | 1.20532600 | 1.88523000 |
| H | -0.52740300 | 1.27897100 | 2.07577400 |
| H | 1.02863100 | 2.08523500 | 2.31446000 |
| H | 0.92781700 | 0.31801400 | 2.39241200 |
| C | 0.29348800 | 2.40646300 | -0.27928900 |
| H | 0.41607000 | 2.35361500 | -1.36294200 |
| H | 0.85742100 | 3.26664300 | 0.08724000 |
| H | -0.76107400 | 2.56920100 | -0.04822800 |
| C | 2.39129700 | -0.61366400 | -0.28996800 |
| C | 2.91427700 | -1.44387100 | 0.88566600 |
| H | 2.89324800 | -2.50525700 | 0.63482600 |
| H | 3.94363500 | -1.15477100 | 1.11506500 |
| H | 2.30973500 | -1.29888400 | 1.78378300 |
| C | 3.26452900 | -0.87231900 | -1.51190600 |
| H | 4.28925800 | -0.53829600 | -1.32482900 |
| H | 3.27913100 | -1.93781700 | -1.74570600 |
| H | 2.88367800 | -0.33802100 | -2.38509500 |
| H | -1.19347100 | -1.44794900 | 1.58070500 |
| H | -5.06794400 | -0.64303300 | -0.09061700 |
| H | -1.53074500 | 0.91788500 | -1.95002100 |

35

a-1-1 carbene-Au fragment

| | | | |
|---|---|---|---|
| Au | 1.22505900 | -1.34095900 | 0.00114400 |
| C | -2.02660300 | -0.67642300 | 1.04566300 |
| N | -0.67046800 | 0.96276200 | -0.13767700 |
| C | -0.86150200 | 2.40345600 | 0.21087600 |
| C | -3.09452600 | -1.56218800 | 1.07890300 |
| H | -3.28731100 | -2.13735300 | 1.97506900 |
| C | 0.57046700 | 0.57187700 | -0.32913600 |
| C | -2.57094600 | -0.11105400 | -1.23743100 |
| C | -3.90161800 | -1.72135300 | -0.04138900 |
| C | -1.77568300 | 0.04926900 | -0.11207600 |
| C | 0.51432800 | 2.96837500 | -0.16062400 |
| H | 0.83471400 | 3.73572300 | 0.54691200 |
| H | 0.45755900 | 3.42842000 | -1.15115300 |
| C | -3.63754100 | -0.99994300 | -1.19910600 |
| H | -4.25633400 | -1.13475600 | -2.07683200 |
| C | -1.18791600 | 2.52498200 | 1.69518000 |
| H | -2.13642100 | 2.03275500 | 1.92027400 |
| H | -1.27840400 | 3.57867200 | 1.96498600 |
| H | -0.40801300 | 2.07368900 | 2.30984100 |
| C | -1.97951800 | 3.02053500 | -0.61404800 |
| H | -1.79636900 | 2.88290400 | -1.68138200 |
| H | -2.02282600 | 4.09157300 | -0.40803800 |
| H | -2.94792000 | 2.58442300 | -0.36185300 |
| C | 1.48247200 | 1.77046600 | -0.20176900 |
| C | 2.30578900 | 1.64189000 | 1.08412100 |
| H | 2.95666300 | 0.76801200 | 1.02903700 |
| H | 2.91934000 | 2.53819600 | 1.20812100 |
| H | 1.67305600 | 1.52750500 | 1.96589000 |
| C | 2.43323200 | 1.82652400 | -1.39353200 |
| H | 3.08497500 | 2.70036900 | -1.31324100 |
| H | 3.05097700 | 0.92646700 | -1.41685800 |
| H | 1.88232300 | 1.88952100 | -2.33409400 |
| H | -1.36462600 | -0.55748600 | 1.89529400 |

| | | | |
|---|---|---|---|
| H | -4.73056700 | -2.41700500 | -0.01576800 |
| H | -2.33980000 | 0.44629800 | -2.13662200 |

38

b-1-1 carbene fragment

| | | | |
|---|---|---|---|
| C | 1.69341000 | 1.27282500 | -0.19216900 |
| N | -0.33752900 | -0.09327200 | -0.12001900 |
| C | -0.98295300 | -0.29508200 | 1.22867400 |
| C | 3.08741900 | 1.36010600 | -0.19790100 |
| H | 3.55894600 | 2.33469300 | -0.15382300 |
| C | -1.11186200 | 0.08962300 | -1.16163700 |
| C | 1.85992500 | -1.14298300 | -0.41709200 |
| C | 1.09252100 | 0.00896000 | -0.21648800 |
| C | -2.44207300 | -0.48324300 | 0.80671100 |
| H | -3.12422200 | 0.03411000 | 1.48563400 |
| H | -2.69448800 | -1.54748000 | 0.83012100 |
| C | 3.25260700 | -1.03851600 | -0.42324200 |
| H | 3.85224400 | -1.93154100 | -0.55358800 |
| C | -0.75849200 | 0.93290300 | 2.10392900 |
| H | 0.30862400 | 1.10537800 | 2.25654800 |
| H | -1.22497100 | 0.76411200 | 3.07659400 |
| H | -1.18646100 | 1.83203000 | 1.66243100 |
| C | -0.41679900 | -1.51363800 | 1.94136300 |
| H | -0.56851100 | -2.42290600 | 1.36132600 |
| H | -0.92208900 | -1.62258600 | 2.90328400 |
| H | 0.65323800 | -1.39724300 | 2.12681200 |
| C | -2.53523300 | 0.04044000 | -0.63986700 |
| C | -3.05777500 | 1.47912600 | -0.70426200 |
| H | -3.02869500 | 1.84556000 | -1.73183100 |
| H | -4.09019200 | 1.51653600 | -0.34641400 |
| H | -2.46063600 | 2.15351900 | -0.08506200 |
| C | -3.40172700 | -0.84676700 | -1.52445300 |
| H | -4.42835300 | -0.87392400 | -1.14819200 |
| H | -3.41214900 | -0.46711400 | -2.54735800 |
| H | -3.01996100 | -1.87039600 | -1.54525600 |

| | | | |
|---|---|---|---|
| N | 0.90038500 | 2.41276900 | -0.15198100 |
| H | 0.01921900 | 2.31498200 | -0.64601600 |
| H | 1.38695600 | 3.26377600 | -0.39645000 |
| N | 1.23127600 | -2.36812600 | -0.59755000 |
| H | 1.81369800 | -3.08081400 | -1.01345000 |
| H | 0.31736500 | -2.30699500 | -1.03038300 |
| C | 3.84817000 | 0.20562500 | -0.29062300 |
| H | 4.92869500 | 0.28043700 | -0.29572400 |

39

b-1-1 carbene-Au fragment

| | | | |
|---|---|---|---|
| Au | 1.37288300 | -1.32628000 | -0.06385300 |
| C | -1.97797600 | -0.65379800 | 1.15673800 |
| N | -0.54640400 | 0.94604600 | -0.03181400 |
| C | -0.76708300 | 2.40011900 | 0.27086600 |
| C | -3.11726400 | -1.46142600 | 1.16453800 |
| H | -3.39907200 | -1.97003700 | 2.07857900 |
| C | 0.69256900 | 0.59423400 | -0.28896900 |
| C | -2.31662200 | -0.24377600 | -1.22314300 |
| C | -1.63990500 | 0.01633200 | -0.02573700 |
| C | 0.59596200 | 2.98113000 | -0.12574700 |
| H | 0.90930700 | 3.76963200 | 0.56140500 |
| H | 0.52365800 | 3.41698100 | -1.12636600 |
| C | -3.45053000 | -1.05578600 | -1.19380100 |
| H | -3.99331900 | -1.24553100 | -2.11195800 |
| C | -1.10507100 | 2.59523700 | 1.74381300 |
| H | -2.02451800 | 2.06660400 | 1.99881300 |
| H | -1.25159700 | 3.66100600 | 1.93021900 |
| H | -0.31372900 | 2.23117200 | 2.39770900 |
| C | -1.90195100 | 2.95852300 | -0.57397200 |
| H | -1.70885300 | 2.82655600 | -1.63760900 |
| H | -2.00295600 | 4.02423800 | -0.36002100 |
| H | -2.84662700 | 2.46790100 | -0.33013100 |
| C | 1.58672500 | 1.80310000 | -0.15046100 |
| C | 2.37057100 | 1.67135100 | 1.16055800 |

| | | | |
|---|---|---|---|
| H | 3.03728100 | 0.80854300 | 1.11708900 |
| H | 2.96485900 | 2.57498600 | 1.31848100 |
| H | 1.70716300 | 1.53848900 | 2.01793500 |
| C | 2.56817900 | 1.88000400 | -1.31387200 |
| H | 3.20561100 | 2.76189400 | -1.21235400 |
| H | 3.19926200 | 0.98879700 | -1.32848000 |
| H | 2.04147100 | 1.94417900 | -2.26876500 |
| N | -1.19581500 | -0.49882800 | 2.29430100 |
| H | -0.19967900 | -0.42737000 | 2.09885500 |
| H | -1.37621500 | -1.19501100 | 3.00532200 |
| N | -1.86202600 | 0.31906300 | -2.40987300 |
| H | -2.24240600 | -0.10842200 | -3.24268600 |
| H | -0.85574500 | 0.42987800 | -2.46162500 |
| C | -3.84966800 | -1.63321500 | 0.00154300 |
| H | -4.72951200 | -2.26447800 | 0.01752400 |

36

c-1-1 carbene fragment

| | | | |
|---|---|---|---|
| C | 1.67645700 | 1.25440700 | -0.11703400 |
| N | -0.35347200 | -0.10592500 | -0.11366100 |
| C | -1.03136400 | -0.33436300 | 1.20688900 |
| C | 3.05884700 | 1.38036900 | -0.21978500 |
| H | 3.51324700 | 2.36293900 | -0.15913000 |
| C | -1.08692700 | 0.09321600 | -1.18265300 |
| C | 1.86434700 | -1.12386600 | -0.40453600 |
| C | 1.06894600 | -0.00005800 | -0.18390500 |
| C | -2.48363800 | -0.47757400 | 0.74406300 |
| H | -3.16915200 | 0.04986400 | 1.41214300 |
| H | -2.76484600 | -1.53452800 | 0.74491600 |
| C | 3.24763900 | -1.00386400 | -0.50783700 |
| H | 3.85019900 | -1.88982500 | -0.67440900 |
| C | -0.80689800 | 0.85579000 | 2.13242400 |
| H | 0.25845600 | 0.98980200 | 2.33512200 |
| H | -1.31346900 | 0.67071900 | 3.08186000 |
| H | -1.19144500 | 1.77812600 | 1.69964300 |

| | | | |
|---|---|---|---|
| C | -0.50617900 | -1.59528800 | 1.87687600 |
| H | -0.64132400 | -2.46321000 | 1.23297500 |
| H | -1.05014600 | -1.75553500 | 2.81014900 |
| H | 0.55635100 | -1.49916400 | 2.11555700 |
| C | -2.52308900 | 0.06893700 | -0.69720700 |
| C | -2.98709300 | 1.52876100 | -0.74737700 |
| H | -2.95348500 | 1.89802500 | -1.77340900 |
| H | -4.01317800 | 1.60509500 | -0.37661000 |
| H | -2.34589400 | 2.17222800 | -0.14070700 |
| C | -3.39968800 | -0.77309600 | -1.61409900 |
| H | -4.43663100 | -0.76386500 | -1.26529800 |
| H | -3.36810000 | -0.37856700 | -2.63092000 |
| H | -3.05554100 | -1.80922600 | -1.64023500 |
| C | 3.83421300 | 0.24772200 | -0.41055400 |
| H | 4.90928000 | 0.34263600 | -0.49301800 |
| O | 1.23906300 | -2.31806000 | -0.53329800 |
| H | 1.88440900 | -3.00356900 | -0.74342300 |
| O | 0.86859600 | 2.33084500 | 0.03537400 |
| H | 1.39087100 | 3.14069000 | -0.00778600 |

37

c-1-1 carbene-Au fragment

| | | | |
|---|---|---|---|
| Au | 1.23511000 | -1.40327100 | -0.11269700 |
| C | -1.84819800 | -0.52746600 | 1.19729400 |
| N | -0.49021300 | 1.04646000 | -0.07123300 |
| C | -0.61491400 | 2.51372600 | 0.19474700 |
| C | -2.86811800 | -1.46922600 | 1.26938900 |
| H | -3.04490000 | -1.99578600 | 2.19932300 |
| C | 0.72065700 | 0.58490200 | -0.25538600 |
| C | -2.37345300 | -0.12608100 | -1.12092500 |
| C | -1.60900000 | 0.16275100 | 0.00783400 |
| C | 0.80951300 | 2.98066300 | -0.13184500 |
| H | 1.15109800 | 3.73906500 | 0.57554100 |
| H | 0.82561400 | 3.42186000 | -1.13200400 |
| C | -3.39913000 | -1.06273600 | -1.04955000 |

| | | | |
|---|---|---|---|
| H | -3.98978900 | -1.27771200 | -1.93272000 |
| C | -1.01349400 | 2.74942600 | 1.64637600 |
| H | -1.97955200 | 2.28571800 | 1.85801100 |
| H | -1.10384600 | 3.82346200 | 1.81886800 |
| H | -0.27994900 | 2.33874800 | 2.33784700 |
| C | -1.65533500 | 3.13395100 | -0.72404100 |
| H | -1.40424000 | 2.96357100 | -1.76984200 |
| H | -1.69623100 | 4.20854900 | -0.53650200 |
| H | -2.64598200 | 2.71564500 | -0.52947500 |
| C | 1.69798100 | 1.72239200 | -0.11021000 |
| C | 2.40313300 | 1.52592700 | 1.23791100 |
| H | 3.00221000 | 0.61391900 | 1.21649200 |
| H | 3.05629600 | 2.38054500 | 1.43208000 |
| H | 1.68688800 | 1.43222400 | 2.05660900 |
| C | 2.73146200 | 1.70418000 | -1.22911100 |
| H | 3.43794900 | 2.52824500 | -1.10089300 |
| H | 3.28180600 | 0.76118400 | -1.20986400 |
| H | 2.25510900 | 1.80389400 | -2.20626900 |
| C | -3.63493700 | -1.72714500 | 0.14398700 |
| H | -4.42620400 | -2.46375900 | 0.19542400 |
| O | -2.05194000 | 0.50886600 | -2.27256700 |
| H | -2.56410200 | 0.14302600 | -3.00386100 |
| O | -1.04389900 | -0.24477900 | 2.24958000 |
| H | -1.01464400 | -0.99347200 | 2.85799500 |

42

d-1-1 carbene fragment

| | | | |
|---|---|---|---|
| C | 1.39547000 | 1.34023700 | -0.15220200 |
| N | -0.53141100 | -0.15720000 | -0.06972400 |
| C | -1.22439200 | -0.31796400 | 1.25339100 |
| C | 2.77258700 | 1.54962400 | -0.22796300 |
| H | 3.18174900 | 2.54923600 | -0.24324400 |
| C | -1.25149200 | -0.10003100 | -1.16375100 |
| C | 1.74942700 | -1.04975600 | -0.23011800 |
| C | 3.61766900 | 0.45260500 | -0.29559200 |

| | | | |
|---|---|---|---|
| C | 0.88256500 | 0.04015900 | -0.12307500 |
| C | -2.65251200 | -0.59994800 | 0.77886700 |
| H | -3.38809100 | -0.06795600 | 1.38757600 |
| H | -2.86117200 | -1.67023500 | 0.86436000 |
| C | 3.12750300 | -0.84418700 | -0.30513300 |
| H | 3.80928800 | -1.67861000 | -0.38057800 |
| C | -1.10452700 | 0.95836500 | 2.07885400 |
| H | -0.05588000 | 1.18328900 | 2.28808200 |
| H | -1.61935500 | 0.81675800 | 3.03139400 |
| H | -1.54270100 | 1.81180600 | 1.56360800 |
| C | -0.63490100 | -1.47675700 | 2.04396300 |
| H | -0.70444800 | -2.40663500 | 1.48142500 |
| H | -1.18651300 | -1.58512200 | 2.98030200 |
| H | 0.41464200 | -1.29142200 | 2.28735500 |
| C | -2.69439600 | -0.18029700 | -0.70383300 |
| C | -3.25311000 | 1.23487600 | -0.88640800 |
| H | -3.21795600 | 1.51912000 | -1.93917000 |
| H | -4.29096200 | 1.27244700 | -0.54342000 |
| H | -2.67076000 | 1.96795300 | -0.32345200 |
| C | -3.49185700 | -1.15399100 | -1.56059800 |
| H | -4.53491900 | -1.18680600 | -1.23192000 |
| H | -3.46306100 | -0.84520300 | -2.60675600 |
| H | -3.07868600 | -2.16267700 | -1.49225800 |
| H | 4.68691700 | 0.61290200 | -0.35368900 |
| O | 0.48815600 | 2.33910000 | -0.11916100 |
| O | 1.17257300 | -2.26901700 | -0.27615300 |
| C | 0.95190500 | 3.66376500 | -0.22245400 |
| H | 1.50124800 | 3.81748300 | -1.15525900 |
| H | 0.07153800 | 4.30100200 | -0.21530100 |
| H | 1.59624000 | 3.92783500 | 0.62143600 |
| C | 2.00420000 | -3.39269900 | -0.43825500 |
| H | 1.34955800 | -4.25926600 | -0.47677900 |
| H | 2.57522100 | -3.33010500 | -1.36869700 |
| H | 2.69732300 | -3.50004600 | 0.40140400 |

43
d-1-1 carbene-Au fragment

| | | | |
|---|---|---|---|
| Au | 1.36171100 | -1.20958100 | -0.61054700 |
| C | -1.40331300 | -0.83963700 | 1.35943100 |
| N | -0.42079800 | 1.05942600 | 0.20955900 |
| C | -0.54662400 | 2.43436900 | 0.78633600 |
| C | -2.33854100 | -1.86956500 | 1.42809400 |
| H | -2.29435500 | -2.59654200 | 2.22634600 |
| C | 0.75363800 | 0.72923100 | -0.26158900 |
| C | -2.43371200 | -0.03223800 | -0.68028700 |
| C | -3.31789800 | -1.95361200 | 0.45169300 |
| C | -1.46413500 | 0.08918600 | 0.31724100 |
| C | 0.77768900 | 3.05591700 | 0.32435000 |
| H | 1.21174800 | 3.68952400 | 1.10054800 |
| H | 0.59822800 | 3.67829600 | -0.55644400 |
| C | -3.38073600 | -1.05179100 | -0.60151000 |
| H | -4.14248200 | -1.15925000 | -1.35968500 |
| C | -0.68379300 | 2.35574100 | 2.30215300 |
| H | -1.57290000 | 1.78342600 | 2.57579000 |
| H | -0.78975000 | 3.36662700 | 2.70027300 |
| H | 0.18100400 | 1.88147200 | 2.76209500 |
| C | -1.75709700 | 3.14961900 | 0.20798900 |
| H | -1.69411900 | 3.20861300 | -0.87745900 |
| H | -1.79888200 | 4.15993500 | 0.61943500 |
| H | -2.68066800 | 2.63174600 | 0.47779900 |
| C | 1.70068300 | 1.88287500 | -0.05517000 |
| C | 2.63674700 | 1.47718800 | 1.09087600 |
| H | 3.25787200 | 0.63377600 | 0.78402300 |
| H | 3.28005300 | 2.32199700 | 1.34995300 |
| H | 2.07713200 | 1.17092700 | 1.97729100 |
| C | 2.52369600 | 2.15260600 | -1.30806300 |
| H | 3.21503100 | 2.98080700 | -1.13257500 |
| H | 3.09889100 | 1.26266700 | -1.57215400 |
| H | 1.88157000 | 2.40958100 | -2.15269800 |

| | | | |
|---|---|---|---|
| H | -4.04820900 | -2.75112300 | 0.50373000 |
| O | -0.40022500 | -0.68097300 | 2.24669100 |
| O | -2.35639600 | 0.84787500 | -1.69996600 |
| C | 0.06878700 | -1.83644400 | 2.91611500 |
| H | 0.24114800 | -2.64236600 | 2.19770500 |
| H | 1.01336900 | -1.56226900 | 3.37929600 |
| H | -0.63209300 | -2.16127300 | 3.68961600 |
| C | -3.23669000 | 0.68105400 | -2.78821200 |
| H | -2.97795900 | 1.44601100 | -3.51518500 |
| H | -3.11566700 | -0.30600800 | -3.24160800 |
| H | -4.27780600 | 0.81215400 | -2.48027700 |

52

e-1-1 carbene fragment

| | | | |
|---|---|---|---|
| C | 1.27722400 | 1.43127400 | -0.05080100 |
| N | -0.60405200 | -0.14046500 | 0.03339300 |
| C | -1.29570000 | -0.29243500 | 1.36643600 |
| C | 2.65466200 | 1.64149200 | -0.03055300 |
| H | 3.03505700 | 2.65599400 | -0.06125700 |
| C | -1.32488000 | -0.20534500 | -1.05756800 |
| C | 0.42665500 | 3.03023200 | -1.73490900 |
| H | 0.15408100 | 2.19633300 | -2.38400900 |
| H | 1.44275400 | 3.34881500 | -1.98409000 |
| H | -0.25070300 | 3.86483200 | -1.93013900 |
| C | 1.69002100 | -0.97921900 | -0.06957800 |
| C | 3.54122400 | 0.57689500 | -0.00443000 |
| H | 4.60852100 | 0.75923700 | 0.01510400 |
| C | 0.81243500 | 0.11077200 | -0.01521200 |
| C | 1.43538700 | -2.76728800 | -1.76044700 |
| H | 0.91267800 | -2.06941400 | -2.41703600 |
| H | 1.07454900 | -3.77838900 | -1.96223500 |
| H | 2.50261200 | -2.73155100 | -1.99596500 |
| C | 0.34977400 | 2.60899600 | -0.26610100 |
| H | -0.67289000 | 2.28025400 | -0.08284100 |
| C | 1.20442000 | -2.39620900 | -0.29438300 |

| | | | |
|---|---|---|---|
| H | 0.12678200 | -2.42370900 | -0.12651200 |
| C | -2.67360500 | -0.76838100 | 0.90129800 |
| H | -3.47511700 | -0.30663800 | 1.48312500 |
| H | -2.74787400 | -1.85197000 | 1.03455600 |
| C | 0.64980900 | 3.79033200 | 0.65119500 |
| H | 0.65203900 | 3.49829600 | 1.70327100 |
| H | -0.09997000 | 4.57178600 | 0.51309400 |
| H | 1.62262100 | 4.23159800 | 0.42291700 |
| C | 3.05845600 | -0.72172700 | -0.05045800 |
| H | 3.75415000 | -1.55178100 | -0.09572000 |
| C | 1.86746600 | -3.40921200 | 0.63381000 |
| H | 1.76167800 | -3.12493600 | 1.68277200 |
| H | 2.93387600 | -3.50367100 | 0.41712600 |
| H | 1.42019000 | -4.39567600 | 0.49612200 |
| C | -1.33805500 | 1.04147800 | 2.10576700 |
| H | -0.32729200 | 1.42831500 | 2.25824700 |
| H | -1.79336200 | 0.88948000 | 3.08608100 |
| H | -1.92524900 | 1.78916800 | 1.57285600 |
| C | -0.59341300 | -1.30479000 | 2.26191700 |
| H | -0.58860800 | -2.30199600 | 1.82279500 |
| H | -1.12715900 | -1.35995600 | 3.21266200 |
| H | 0.43644400 | -1.00308200 | 2.46879400 |
| C | -2.75410200 | -0.42716400 | -0.60083400 |
| C | -3.50404300 | 0.88092800 | -0.86619300 |
| C | -3.39355100 | -1.54965700 | -1.40892900 |
| H | -3.46182300 | 1.12907700 | -1.92789300 |
| H | -3.07064000 | 1.71566000 | -0.30994000 |
| H | -3.40839500 | -1.29141500 | -2.46880100 |
| H | -2.83060000 | -2.47957100 | -1.29411800 |
| H | -4.55133100 | 0.77816400 | -0.56920900 |
| H | -4.41977400 | -1.72583900 | -1.07367400 |

53

e-1-1 carbene-Au fragment

| | | | |
|---|---|---|---|
| Au | -1.62915200 | -0.76276200 | -0.97998200 |

| | | | |
|---|---|---|---|
| C | 1.86984900 | 0.92099000 | -0.99139500 |
| N | 0.34210400 | 0.51941900 | 0.88273100 |
| C | 0.61427700 | 1.23733200 | 2.17608800 |
| C | 2.96619500 | 0.50536900 | -1.74332600 |
| H | 3.32967300 | 1.14357700 | -2.54041400 |
| C | -0.91343700 | 0.43450700 | 0.53079800 |
| C | 0.44563700 | 1.96318200 | -2.73715200 |
| H | -0.24299700 | 1.11688000 | -2.67857500 |
| H | 1.17975200 | 1.75427500 | -3.51981600 |
| H | -0.11651900 | 2.85347300 | -3.02723100 |
| C | 1.96417200 | -1.20198800 | 0.23029000 |
| C | 3.57591000 | -0.71615200 | -1.50943800 |
| H | 4.42956900 | -1.01945700 | -2.10242300 |
| C | 1.41924700 | 0.07416200 | 0.03150900 |
| C | 0.75580000 | -3.37588000 | 0.32327400 |
| H | 0.01328300 | -3.00163100 | -0.38548600 |
| H | 0.27580000 | -4.10745000 | 0.97677100 |
| H | 1.54201400 | -3.88750500 | -0.23815900 |
| C | 1.15003100 | 2.19144000 | -1.39793300 |
| H | 0.37642600 | 2.40663500 | -0.65922500 |
| C | 1.35300800 | -2.23703800 | 1.15392800 |
| H | 0.53139400 | -1.77087200 | 1.70352600 |
| C | -0.80185600 | 1.34106700 | 2.75333200 |
| H | -0.96803100 | 2.31094000 | 3.22687500 |
| H | -0.94022600 | 0.56856500 | 3.51476100 |
| C | 2.07982900 | 3.39894400 | -1.49552800 |
| H | 2.63211100 | 3.57253700 | -0.57066600 |
| H | 1.50308500 | 4.29700400 | -1.72538100 |
| H | 2.80687800 | 3.26572300 | -2.29967000 |
| C | 3.05957400 | -1.56879200 | -0.54782000 |
| H | 3.49634800 | -2.55122700 | -0.41036800 |
| C | 2.36462300 | -2.80727900 | 2.14754200 |
| H | 2.87238000 | -2.03047300 | 2.72035400 |
| H | 3.12766100 | -3.38747800 | 1.62354400 |

| | | | |
|---|---|---|---|
| H | 1.86592800 | -3.48256100 | 2.84566300 |
| C | 1.24908200 | 2.59447300 | 1.89571500 |
| H | 2.18795300 | 2.46895000 | 1.35221400 |
| H | 1.46880900 | 3.08310300 | 2.84631000 |
| H | 0.59288600 | 3.24724800 | 1.32022700 |
| C | 1.55186900 | 0.44677000 | 3.07353900 |
| H | 1.13115500 | -0.52139300 | 3.34289800 |
| H | 1.71509700 | 1.01371400 | 3.99189900 |
| H | 2.52010100 | 0.29506200 | 2.59095000 |
| C | -1.77176800 | 1.08559400 | 1.58448500 |
| C | -2.32741100 | 2.37522800 | 0.97198400 |
| C | -2.92965700 | 0.17859300 | 1.98642200 |
| H | -2.91029300 | 2.13976100 | 0.07925900 |
| H | -1.53152100 | 3.06668400 | 0.68647300 |
| H | -3.59063000 | 0.00433000 | 1.13570400 |
| H | -2.56448300 | -0.79116000 | 2.33230900 |
| H | -2.97485300 | 2.87574300 | 1.69604300 |
| H | -3.50032500 | 0.64615500 | 2.79321400 |

58

e-2-1 carbene fragment

| | | | |
|---|---|---|---|
| C | -1.98439600 | -1.01330900 | -0.06970200 |
| N | 0.24820600 | -0.03080400 | 0.15501600 |
| C | 0.84603500 | -0.06972500 | 1.53964000 |
| C | -3.36288800 | -0.83777700 | -0.16273500 |
| H | -4.00505700 | -1.70946000 | -0.21552100 |
| C | 1.04870700 | -0.09363200 | -0.87780500 |
| C | -1.47300500 | -2.83518300 | -1.66035700 |
| H | -0.94817000 | -2.11724900 | -2.29445200 |
| H | -2.51452400 | -2.88948200 | -1.98928200 |
| H | -1.01918700 | -3.82032100 | -1.79362200 |
| C | -1.70928700 | 1.41462700 | -0.14768400 |
| C | -3.91947400 | 0.43031700 | -0.21999100 |
| H | -4.99369200 | 0.54978900 | -0.28743000 |
| C | -1.17478700 | 0.12753600 | -0.00789900 |

| | | | |
|---|---|---|---|
| C | -0.83569800 | 2.99101300 | -1.84198400 |
| H | -0.47386300 | 2.14805100 | -2.43336300 |
| H | -0.19262100 | 3.85418600 | -2.02884300 |
| H | -1.84776900 | 3.24039300 | -2.17293300 |
| C | -1.39984200 | -2.40394900 | -0.19444600 |
| H | -0.34320500 | -2.35681700 | 0.06686300 |
| C | -0.83702000 | 2.63529600 | -0.35401400 |
| H | 0.18757500 | 2.37513900 | -0.09011200 |
| C | 2.31958300 | -0.33543400 | 1.20972500 |
| H | 2.59793300 | -1.34493500 | 1.52138700 |
| H | 2.96643700 | 0.35805300 | 1.75304600 |
| C | -2.07589500 | -3.42795200 | 0.71095800 |
| H | -2.06546800 | -3.11042400 | 1.75569400 |
| H | -1.56352000 | -4.38937900 | 0.63823800 |
| H | -3.11557100 | -3.59164000 | 0.41870000 |
| C | -3.09372800 | 1.54304300 | -0.23857200 |
| H | -3.52654400 | 2.53051400 | -0.35031800 |
| C | -1.26371900 | 3.83260100 | 0.48927100 |
| H | -1.31135600 | 3.58436800 | 1.55144000 |
| H | -2.24590900 | 4.20146800 | 0.18535200 |
| H | -0.55553500 | 4.65341900 | 0.35966600 |
| C | 0.22771000 | -1.17512300 | 2.38603200 |
| H | -0.85356800 | -1.04529500 | 2.47868500 |
| H | 0.66116800 | -1.13509300 | 3.38714600 |
| H | 0.43211700 | -2.16320000 | 1.97124300 |
| C | 0.63396000 | 1.26094600 | 2.25421300 |
| H | 1.15541400 | 2.07716000 | 1.75247200 |
| H | 1.02555000 | 1.18442300 | 3.27018800 |
| H | -0.42956200 | 1.50457800 | 2.31715900 |
| C | 2.45681400 | -0.16864600 | -0.31991900 |
| C | 3.20590800 | -1.33405900 | -0.97487100 |
| C | 3.11461300 | 1.17573900 | -0.68355100 |
| H | 3.41351000 | -1.06372200 | -2.01548900 |
| H | 2.94584100 | 1.35651700 | -1.74917600 |

| | | | |
|---|---|---|---|
| H | 2.58327200 | 1.97230700 | -0.14772000 |
| H | 4.17246900 | -1.45914500 | -0.47553900 |
| C | 4.60307500 | 1.24774400 | -0.36977800 |
| H | 4.98550700 | 2.25594200 | -0.53307100 |
| H | 4.80982600 | 0.97854100 | 0.66924800 |
| H | 5.17419700 | 0.57135600 | -1.00718900 |
| C | 2.42374500 | -2.64045100 | -0.93658800 |
| H | 3.01119700 | -3.46994800 | -1.33192200 |
| H | 2.13194000 | -2.90272300 | 0.08562500 |
| H | 1.51126100 | -2.55347900 | -1.53093900 |

59

e-2-1 carbene-Au fragment

| | | | |
|---|---|---|---|
| Au | 0.92503000 | -0.21157400 | -1.77061300 |
| C | -2.27181000 | -1.02590900 | 0.29198200 |
| N | -0.21761800 | 0.09614700 | 1.01392800 |
| C | -0.00184600 | 0.22065700 | 2.49850800 |
| C | -3.57366900 | -0.93424200 | -0.19285600 |
| H | -4.15332500 | -1.84125400 | -0.31946700 |
| C | 0.85604900 | -0.01116000 | 0.28694100 |
| C | -1.44423300 | -3.07825200 | -0.83986600 |
| H | -0.81583500 | -2.46551800 | -1.49208700 |
| H | -2.40023700 | -3.24277100 | -1.34423200 |
| H | -0.96172900 | -4.04841200 | -0.69959800 |
| C | -2.04375600 | 1.39910300 | 0.01282900 |
| C | -4.12330100 | 0.28837700 | -0.54261800 |
| H | -5.13879600 | 0.34103100 | -0.91455400 |
| C | -1.54216300 | 0.16145500 | 0.43943900 |
| C | -0.97808800 | 3.01630800 | -1.54774700 |
| H | -0.51102800 | 2.18860900 | -2.08697400 |
| H | -0.33248700 | 3.89347900 | -1.62758600 |
| H | -1.92909800 | 3.25024500 | -2.03336000 |
| C | -1.66681800 | -2.39711400 | 0.51214200 |
| H | -0.68625200 | -2.27259700 | 0.97309700 |
| C | -1.20657200 | 2.66024100 | -0.07664100 |

| | | | |
|---|---|---|---|
| H | -0.22716600 | 2.46224600 | 0.36378000 |
| C | 1.51593400 | 0.00816200 | 2.60698900 |
| H | 1.72649200 | -0.94546500 | 3.09553100 |
| H | 1.96753200 | 0.79245900 | 3.21831500 |
| C | -2.52166600 | -3.28570100 | 1.41296500 |
| H | -2.74156100 | -2.81202500 | 2.37114000 |
| H | -2.00728700 | -4.22949300 | 1.60464400 |
| H | -3.47236400 | -3.52615600 | 0.93189800 |
| C | -3.35327800 | 1.43663000 | -0.46155400 |
| H | -3.76301700 | 2.38244400 | -0.79676500 |
| C | -1.84366500 | 3.84705800 | 0.64433500 |
| H | -2.05794000 | 3.63456700 | 1.69235800 |
| H | -2.78200200 | 4.13038200 | 0.16205300 |
| H | -1.18002800 | 4.71275100 | 0.59757500 |
| C | -0.79391000 | -0.82795200 | 3.26396400 |
| H | -1.86339300 | -0.73633000 | 3.06145200 |
| H | -0.63492900 | -0.67103500 | 4.33227600 |
| H | -0.46937600 | -1.83971800 | 3.01834500 |
| C | -0.43594700 | 1.60127000 | 2.97117900 |
| H | 0.14289400 | 2.39306700 | 2.49366000 |
| H | -0.28287900 | 1.66824300 | 4.04949500 |
| H | -1.49637500 | 1.76135300 | 2.76595900 |
| C | 2.07368800 | 0.02461600 | 1.16913000 |
| C | 2.97385400 | -1.17887800 | 0.86014200 |
| C | 2.79719400 | 1.34691300 | 0.84561900 |
| H | 3.43868800 | -1.00960900 | -0.11731400 |
| H | 2.93395300 | 1.39807400 | -0.23930200 |
| H | 2.13115100 | 2.17600100 | 1.11571900 |
| H | 3.77518100 | -1.20456500 | 1.60491200 |
| C | 4.13601200 | 1.50326500 | 1.55246100 |
| H | 4.53923800 | 2.50324000 | 1.39204500 |
| H | 4.04543300 | 1.35060600 | 2.63101400 |
| H | 4.86777400 | 0.78866400 | 1.17458700 |
| C | 2.22791400 | -2.50677900 | 0.84228300 |

| | | | |
|---|---|---|---|
| H | 2.91863400 | -3.34433600 | 0.74497000 |
| H | 1.65305400 | -2.66008600 | 1.76171600 |
| H | 1.53352100 | -2.54519100 | -0.00210600 |

67

e-3-1 carbene fragment

| | | | |
|---|---|---|---|
| C | 2.45253700 | 1.41294200 | -0.19241700 |
| N | 0.64825700 | -0.14594400 | 0.36518800 |
| C | -2.50803600 | -1.41169600 | -0.34539700 |
| H | -1.95512700 | -2.35987400 | -0.35565600 |
| C | -3.63433400 | 0.30705400 | -1.76872400 |
| H | -3.88344100 | 0.59272800 | -2.79477000 |
| C | 0.32273000 | -0.32367000 | 1.82412800 |
| C | 3.78854100 | 1.61752600 | -0.53114900 |
| H | 4.15493800 | 2.63059200 | -0.65070000 |
| C | -2.39530700 | 0.99208000 | 0.29619100 |
| H | -1.77080700 | 1.77656100 | 0.74028700 |
| C | -0.33373200 | -0.14952100 | -0.49882200 |
| C | -2.87310500 | -1.01792800 | -1.77713100 |
| H | -3.50190000 | -1.80181000 | -2.21525900 |
| H | -1.96645900 | -0.92732100 | -2.37771200 |
| C | 1.18979400 | 3.02280100 | -1.58172300 |
| H | 0.76421400 | 2.18725900 | -2.14083700 |
| H | 2.10115000 | 3.35249200 | -2.08861400 |
| H | 0.47659200 | 3.85057000 | -1.58662600 |
| C | 2.82956500 | -0.99911200 | -0.34301800 |
| C | 4.64398600 | 0.54882400 | -0.74788900 |
| H | 5.68048700 | 0.72681000 | -1.00623200 |
| C | -2.75801700 | 1.39030100 | -1.13856200 |
| H | -3.30465700 | 2.34030700 | -1.11498600 |
| H | -1.85127400 | 1.53230400 | -1.73123800 |
| C | -4.91674800 | 0.14487700 | -0.95386300 |
| H | -5.48423200 | 1.08237400 | -0.95317600 |
| H | -5.55056100 | -0.62639100 | -1.40569900 |
| C | 2.00521500 | 0.09340000 | -0.04889300 |

| | | | |
|---|---|---|---|
| C | -1.60718600 | -0.34428700 | 0.30566500 |
| C | 2.13042200 | -2.75950800 | -1.93399400 |
| H | 1.46533400 | -2.04668200 | -2.42516900 |
| H | 1.71710800 | -3.76401000 | -2.04938500 |
| H | 3.10128700 | -2.72910600 | -2.43614300 |
| C | -3.78859800 | -1.56590100 | 0.47690400 |
| H | -4.40848000 | -2.35722400 | 0.04163500 |
| H | -3.55384700 | -1.86502100 | 1.50468200 |
| C | 1.50638400 | 2.59480100 | -0.14719600 |
| H | 0.56748000 | 2.26909500 | 0.30043400 |
| C | 2.28850300 | -2.40937900 | -0.45315300 |
| H | 1.28987400 | -2.43161500 | -0.01454800 |
| C | -1.13441600 | -0.77011900 | 1.71328600 |
| H | -1.73164500 | -0.35956000 | 2.52876900 |
| H | -1.18554000 | -1.86231500 | 1.78931300 |
| C | 2.04403200 | 3.76972800 | 0.66318900 |
| H | 2.31893300 | 3.47097500 | 1.67682800 |
| H | 1.28931000 | 4.55579900 | 0.72927800 |
| H | 2.92629800 | 4.20720400 | 0.19052200 |
| C | 4.15737600 | -0.74732500 | -0.67968000 |
| H | 4.81120600 | -1.57952200 | -0.91444700 |
| C | 3.15758000 | -3.44076600 | 0.25952700 |
| H | 3.32577200 | -3.17335100 | 1.30470000 |
| H | 4.13237200 | -3.53624000 | -0.22400800 |
| H | 2.68145900 | -4.42277600 | 0.22630400 |
| C | -3.69278700 | 0.85141600 | 1.09585400 |
| H | -3.49389500 | 0.62822400 | 2.14778900 |
| H | -4.22980700 | 1.80624500 | 1.06811300 |
| C | 0.49266400 | 0.99314100 | 2.57694500 |
| H | 1.51313300 | 1.36926000 | 2.46654900 |
| H | 0.30832000 | 0.82265100 | 3.63922000 |
| H | -0.20379500 | 1.75680000 | 2.23104000 |
| C | -4.56193500 | -0.24558700 | 0.48050200 |
| H | -5.47694900 | -0.35917900 | 1.06942400 |

| | | | |
|---|---|---|---|
| C | 1.21153300 | -1.36518900 | 2.49080200 |
| H | 1.08548100 | -2.35260000 | 2.04782900 |
| H | 0.93845800 | -1.43396800 | 3.54556900 |
| H | 2.26543000 | -1.08148700 | 2.43231000 |

68

e-3-1 carbene-Au fragment

| | | | |
|---|---|---|---|
| Au | -0.12521200 | -1.72112100 | 0.79545200 |
| C | -2.26275600 | 1.37584800 | 1.05486000 |
| N | -0.62170300 | 0.77218300 | -0.65572500 |
| C | 2.60848100 | -0.62128200 | -1.26080600 |
| H | 2.06580600 | -1.22865800 | -1.99515300 |
| C | 3.83117400 | -0.67315700 | 0.92096100 |
| H | 4.14356500 | -1.31179400 | 1.75136900 |
| C | -0.23098600 | 1.81401000 | -1.65662100 |
| C | -3.58067300 | 1.36559100 | 1.50428300 |
| H | -3.82590800 | 1.87815100 | 2.42729300 |
| C | 2.44203700 | 1.31873400 | 0.29197300 |
| H | 1.79312000 | 2.10483600 | 0.69001900 |
| C | 0.35723300 | 0.04002300 | -0.17383900 |
| C | 3.10400200 | -1.51403200 | -0.12345400 |
| H | 3.78947100 | -2.25910800 | -0.54313900 |
| H | 2.27521800 | -2.05936600 | 0.33741500 |
| C | -0.98138700 | 1.14843800 | 3.17430600 |
| H | -0.70746900 | 0.12716900 | 2.89711300 |
| H | -1.89441300 | 1.10569100 | 3.77425200 |
| H | -0.18615400 | 1.57034300 | 3.79343600 |
| C | -2.93711300 | -0.05411600 | -0.81773700 |
| C | -4.57090100 | 0.69314600 | 0.80737800 |
| H | -5.59138700 | 0.70041900 | 1.16926300 |
| C | 2.88184500 | 0.40628700 | 1.44186600 |
| H | 3.38946200 | 1.01586400 | 2.19760500 |
| H | 2.01112500 | -0.05873300 | 1.91607600 |
| C | 5.05488800 | -0.02833600 | 0.27234000 |
| H | 5.60444400 | 0.57209000 | 1.00594200 |

| | | | |
|---|---|---|---|
| H | 5.73228300 | -0.80716700 | -0.09416100 |
| C | -1.96822700 | 0.70036600 | -0.13943200 |
| C | 1.66672200 | 0.50521900 | -0.78465900 |
| C | -2.85863300 | -2.43691700 | -1.55493300 |
| H | -2.26388200 | -2.69620900 | -0.67617000 |
| H | -2.58745200 | -3.11072900 | -2.37056100 |
| H | -3.91382400 | -2.59911900 | -1.31986900 |
| C | 3.83613100 | 0.02491100 | -1.91550600 |
| H | 4.48752500 | -0.76516400 | -2.30289400 |
| H | 3.54736700 | 0.64928900 | -2.76722400 |
| C | -1.20271500 | 2.01226100 | 1.93115200 |
| H | -0.26208200 | 2.02953800 | 1.38059700 |
| C | -2.62484300 | -0.98314600 | -1.97497900 |
| H | -1.56491700 | -0.88253300 | -2.22250100 |
| C | 1.18905700 | 1.35929400 | -1.97847200 |
| H | 1.82939700 | 2.21081300 | -2.20490300 |
| H | 1.16626900 | 0.71945100 | -2.86784200 |
| C | -1.54730300 | 3.44290800 | 2.33883100 |
| H | -1.75159600 | 4.07869200 | 1.47568600 |
| H | -0.71988700 | 3.88080300 | 2.90081800 |
| H | -2.42639400 | 3.46407000 | 2.98665500 |
| C | -4.24150400 | -0.02549000 | -0.32946000 |
| H | -5.00492300 | -0.60070000 | -0.84060800 |
| C | -3.47258500 | -0.68300100 | -3.21193500 |
| H | -3.41117600 | 0.35909900 | -3.52535100 |
| H | -4.52294700 | -0.90865900 | -3.01279300 |
| H | -3.15736300 | -1.31432300 | -4.04524100 |
| C | 3.69076000 | 1.95356600 | -0.32489200 |
| H | 3.43388600 | 2.67776400 | -1.10207700 |
| H | 4.22168300 | 2.50637300 | 0.45793700 |
| C | -0.30124600 | 3.20829300 | -1.03982200 |
| H | -1.31302900 | 3.41077900 | -0.68126600 |
| H | -0.06060200 | 3.94433100 | -1.80875200 |
| H | 0.39716000 | 3.33928000 | -0.21459000 |

| | | | |
|---|---|---|---|
| C | 4.60091900 | 0.86099700 | -0.88439800 |
| H | 5.47117000 | 1.31977900 | -1.36250600 |
| C | -1.13202100 | 1.80003900 | -2.88020900 |
| H | -1.10432200 | 0.83915300 | -3.39206400 |
| H | -0.78392400 | 2.56608500 | -3.57566800 |
| H | -2.16371400 | 2.03296500 | -2.60756700 |

59

e-4-1 carbene fragment

| | | | |
|---|---|---|---|
| C | 2.03659800 | 1.33042500 | -0.16042800 |
| N | 0.05729000 | -0.08136200 | 0.16023400 |
| C | -0.49442900 | -0.16338500 | 1.56002500 |
| C | 3.42039000 | 1.42809700 | -0.29275000 |
| H | 3.87467200 | 2.40782400 | -0.38564700 |
| C | -0.78233900 | -0.09009900 | -0.84440300 |
| C | 1.14215200 | 2.97495400 | -1.77646800 |
| H | 0.73647500 | 2.15887700 | -2.37684700 |
| H | 2.14808600 | 3.20739300 | -2.13723300 |
| H | 0.51638200 | 3.85958900 | -1.91552300 |
| C | 2.25224700 | -1.10572300 | -0.17277800 |
| C | 4.21725100 | 0.29542400 | -0.33952100 |
| H | 5.29143100 | 0.39073800 | -0.43861600 |
| C | 1.47519700 | 0.05248600 | -0.04674500 |
| C | 1.67419400 | -2.88624600 | -1.79037800 |
| H | 1.14150100 | -2.15536000 | -2.40147900 |
| H | 1.21328700 | -3.86642800 | -1.93202900 |
| H | 2.70909200 | -2.94116300 | -2.13923400 |
| C | 1.18824900 | 2.57724400 | -0.29980900 |
| H | 0.16761200 | 2.33508400 | -0.00430800 |
| C | 1.63328900 | -2.48073900 | -0.31583200 |
| H | 0.58129300 | -2.41780800 | -0.03390600 |
| C | -1.95397900 | -0.51517200 | 1.25892800 |
| H | -2.63233000 | 0.02974900 | 1.91954000 |
| H | -2.11802300 | -1.58381500 | 1.43347800 |
| C | 1.67762600 | 3.74004200 | 0.55778600 |

| | | | |
|---|---|---|---|
| H | 1.76616600 | 3.46086900 | 1.60963200 |
| H | 0.98342800 | 4.57928100 | 0.48236500 |
| H | 2.65384200 | 4.09650700 | 0.22143300 |
| C | 3.63080600 | -0.96015500 | -0.30639100 |
| H | 4.24913000 | -1.84456800 | -0.40911900 |
| C | 2.30882400 | -3.53403500 | 0.55674900 |
| H | 2.33807000 | -3.23086300 | 1.60532400 |
| H | 3.33525800 | -3.71685400 | 0.23097100 |
| H | 1.77249400 | -4.48245500 | 0.48596200 |
| C | -0.34086500 | 1.17557900 | 2.27515800 |
| H | 0.70971500 | 1.47482700 | 2.31079700 |
| H | -0.69909600 | 1.07389300 | 3.30121500 |
| H | -0.91657800 | 1.96537000 | 1.79211100 |
| C | 0.20832200 | -1.22757500 | 2.39268700 |
| H | 0.07529200 | -2.22491400 | 1.97431300 |
| H | -0.22047700 | -1.22543000 | 3.39666800 |
| H | 1.27758000 | -1.01913600 | 2.48068700 |
| C | -2.16544700 | -0.19624300 | -0.23621000 |
| C | -2.86135700 | 1.15558700 | -0.47104200 |
| C | -2.96199700 | -1.28826900 | -0.95371500 |
| C | -4.32452400 | 1.13561800 | -0.04214100 |
| H | -2.79477500 | 1.37938800 | -1.54170200 |
| H | -2.31644400 | 1.95038100 | 0.04994000 |
| C | -4.40933000 | -1.32764600 | -0.48092900 |
| H | -2.92836400 | -1.08044400 | -2.02878600 |
| H | -2.46800000 | -2.25381800 | -0.79925600 |
| C | -5.08220200 | 0.01450800 | -0.74340700 |
| H | -4.78495900 | 2.10331400 | -0.25506900 |
| H | -4.39464200 | 0.98891300 | 1.04205400 |
| H | -4.94977300 | -2.13424700 | -0.98144500 |
| H | -4.44151200 | -1.54440800 | 0.59467200 |
| H | -6.12537700 | -0.00326600 | -0.41939900 |
| H | -5.08354800 | 0.20446700 | -1.82352600 |

60

e-4-1 carbene-Au fragment

| | | | |
|---|---|---|---|
| Au | -0.40251400 | -1.68608100 | -0.96682400 |
| C | 2.13763000 | 1.20853900 | -0.90275400 |
| N | 0.24691100 | 0.79065400 | 0.60201400 |
| C | -0.20146400 | 1.91500800 | 1.49222300 |
| C | 3.50182400 | 1.09080200 | -1.15887400 |
| H | 3.90931100 | 1.54346500 | -2.05545400 |
| C | -0.70334700 | 0.06291500 | 0.08159600 |
| C | 1.20614800 | 0.95994100 | -3.19127700 |
| H | 0.85322900 | -0.04241300 | -2.93683000 |
| H | 2.20158700 | 0.87082600 | -3.63430600 |
| H | 0.53746000 | 1.38715200 | -3.94167100 |
| C | 2.44299800 | -0.18233700 | 1.09280900 |
| C | 4.33426600 | 0.38243500 | -0.30816300 |
| H | 5.39253300 | 0.30531400 | -0.52377800 |
| C | 1.63835200 | 0.61084200 | 0.26325000 |
| C | 2.00700400 | -2.51783600 | 1.83080100 |
| H | 1.48580300 | -2.72354600 | 0.89270700 |
| H | 1.57685700 | -3.14568000 | 2.61404700 |
| H | 3.05689500 | -2.79762600 | 1.71022700 |
| C | 1.25615200 | 1.85669700 | -1.95207700 |
| H | 0.23923300 | 1.92480200 | -1.56172200 |
| C | 1.89326600 | -1.04047100 | 2.21513100 |
| H | 0.83011300 | -0.81806500 | 2.33555800 |
| C | -1.68822600 | 1.58055200 | 1.66417200 |
| H | -2.29747600 | 2.48516600 | 1.61627000 |
| H | -1.85117400 | 1.12703300 | 2.64660800 |
| C | 1.72567200 | 3.25653300 | -2.34281300 |
| H | 1.81148500 | 3.92183800 | -1.48243900 |
| H | 1.02509500 | 3.70101300 | -3.05248200 |
| H | 2.70120000 | 3.21521800 | -2.83246800 |
| C | 3.80003400 | -0.26819500 | 0.79172200 |
| H | 4.44028500 | -0.87711300 | 1.41952400 |
| C | 2.60953600 | -0.79962100 | 3.54334900 |

| | | | |
|---|---|---|---|
| H | 2.61453600 | 0.25268700 | 3.83019900 |
| H | 3.64784800 | -1.13386900 | 3.48350500 |
| H | 2.12926200 | -1.37315100 | 4.33863400 |
| C | 0.02884300 | 3.25158200 | 0.79754400 |
| H | 1.08818100 | 3.38318100 | 0.56758400 |
| H | -0.27626300 | 4.05504700 | 1.46991400 |
| H | -0.54719900 | 3.33951400 | -0.12402600 |
| C | 0.55080200 | 1.92255300 | 2.81278000 |
| H | 0.38403800 | 1.00556500 | 3.37706500 |
| H | 0.19198400 | 2.76072800 | 3.41288300 |
| H | 1.62327700 | 2.05378300 | 2.65157500 |
| C | -2.03818400 | 0.56070200 | 0.56273400 |
| C | -2.75013600 | 1.18609900 | -0.65125700 |
| C | -2.89071200 | -0.59715400 | 1.09086900 |
| C | -4.16904500 | 1.63042300 | -0.31455300 |
| H | -2.77601800 | 0.42647400 | -1.44168800 |
| H | -2.16199100 | 2.02605200 | -1.03641100 |
| C | -4.28642300 | -0.12053900 | 1.46962700 |
| H | -2.95799300 | -1.35831200 | 0.30503500 |
| H | -2.37992900 | -1.06296700 | 1.94012200 |
| C | -4.98380000 | 0.47408700 | 0.25223700 |
| H | -4.64565600 | 2.03705600 | -1.20907500 |
| H | -4.14157900 | 2.44315500 | 0.42082600 |
| H | -4.86530500 | -0.95066700 | 1.87904200 |
| H | -4.22062500 | 0.63962800 | 2.25885100 |
| H | -5.99244000 | 0.80698700 | 0.50629800 |
| H | -5.08349800 | -0.30382100 | -0.51356800 |

57

e-5-1 carbene fragment

| | | | |
|---|---|---|---|
| C | 1.97628800 | 1.33290300 | -0.11985200 |
| N | -0.00388100 | -0.08898800 | 0.12188600 |
| C | -0.60295400 | -0.18003900 | 1.50388400 |
| C | 3.36290400 | 1.44056400 | -0.20501300 |
| H | 3.81371400 | 2.42384400 | -0.27433000 |

| | | | |
|---|---|---|---|
| C | -0.80842600 | -0.09306600 | -0.91132600 |
| C | 1.12475300 | 2.98704700 | -1.74971600 |
| H | 0.74621000 | 2.17375100 | -2.37133300 |
| H | 2.14029100 | 3.23057500 | -2.07435900 |
| H | 0.49709900 | 3.86829000 | -1.90145200 |
| C | 2.20781800 | -1.10179600 | -0.14628000 |
| C | 4.16816500 | 0.31315300 | -0.23505300 |
| H | 5.24439500 | 0.41598900 | -0.29748800 |
| C | 1.41984600 | 0.05032200 | -0.03613700 |
| C | 1.70326500 | -2.87520900 | -1.79619200 |
| H | 1.19077100 | -2.14337800 | -2.42316800 |
| H | 1.25348300 | -3.85683800 | -1.96151100 |
| H | 2.75107300 | -2.92285700 | -2.10524900 |
| C | 1.12435700 | 2.57545500 | -0.27612100 |
| H | 0.09587000 | 2.32391600 | -0.01743800 |
| C | 1.60319400 | -2.47923700 | -0.32184700 |
| H | 0.54095100 | -2.42374600 | -0.08006800 |
| C | -2.05152900 | -0.52436900 | 1.14775600 |
| H | -2.76710000 | 0.00325700 | 1.78360200 |
| H | -2.21120000 | -1.59874500 | 1.28971800 |
| C | 1.57611700 | 3.73309500 | 0.60860700 |
| H | 1.62893700 | 3.44453000 | 1.66028200 |
| H | 0.87995900 | 4.56902700 | 0.51626200 |
| H | 2.56142100 | 4.09819100 | 0.31025600 |
| C | 3.58917100 | -0.94623200 | -0.23250500 |
| H | 4.21632000 | -1.82583800 | -0.32233800 |
| C | 2.25120800 | -3.53423800 | 0.56937800 |
| H | 2.23678300 | -3.23877000 | 1.62041200 |
| H | 3.29102900 | -3.70731800 | 0.28311800 |
| H | 1.72503600 | -4.48584800 | 0.47067700 |
| C | -0.45691300 | 1.14453300 | 2.24679600 |
| H | 0.59637900 | 1.42644900 | 2.32289200 |
| H | -0.84993600 | 1.02850200 | 3.25838600 |
| H | -1.00289400 | 1.95411700 | 1.76338300 |

| | | | |
|---|---|---|---|
| C | 0.06685900 | -1.26148600 | 2.34211300 |
| H | -0.05631700 | -2.25105200 | 1.90283900 |
| H | -0.39544400 | -1.27356800 | 3.33100100 |
| H | 1.13345700 | -1.05961100 | 2.46919700 |
| C | -2.20957600 | -0.17457400 | -0.34466200 |
| C | -2.85848900 | 1.20037100 | -0.55914900 |
| C | -3.04275800 | -1.21302100 | -1.09416500 |
| C | -4.34150100 | 1.16213600 | -0.33503800 |
| H | -2.62980600 | 1.54333900 | -1.57539700 |
| H | -2.40297100 | 1.93666700 | 0.11237000 |
| C | -4.43951400 | -1.31934800 | -0.49498200 |
| H | -3.10364800 | -0.90701800 | -2.14259700 |
| H | -2.53377600 | -2.18173500 | -1.06874000 |
| C | -5.04741200 | 0.03558700 | -0.28578300 |
| H | -4.84621000 | 2.11379700 | -0.20132800 |
| H | -5.08492600 | -1.91674700 | -1.14448500 |
| H | -4.40609300 | -1.85733500 | 0.46112300 |
| H | -6.11492400 | 0.08376000 | -0.09572300 |

58

e-5-1 carbene-Au fragment

| | | | |
|---|---|---|---|
| Au | -0.38388600 | -1.69490900 | -1.01270800 |
| C | 2.05582900 | 1.33116800 | -0.83919900 |
| N | 0.18022500 | 0.75096500 | 0.62274800 |
| C | -0.31898500 | 1.79754600 | 1.57921100 |
| C | 3.42342600 | 1.28415000 | -1.09851100 |
| H | 3.81305600 | 1.80581500 | -1.96500200 |
| C | -0.74147200 | 0.04562500 | 0.01842900 |
| C | 1.10900500 | 1.17415800 | -3.12871000 |
| H | 0.77868100 | 0.15352600 | -2.92101500 |
| H | 2.10334500 | 1.12753300 | -3.58066900 |
| H | 0.42670400 | 1.62324000 | -3.85377700 |
| C | 2.41489900 | -0.16332300 | 1.07037600 |
| C | 4.28334400 | 0.56124900 | -0.28794900 |
| H | 5.34417700 | 0.54047300 | -0.50380100 |

| | | | |
|---|---|---|---|
| C | 1.57904600 | 0.64452300 | 0.28701000 |
| C | 2.10090600 | -2.55677400 | 1.67895300 |
| H | 1.60389200 | -2.74135700 | 0.72374600 |
| H | 1.69409700 | -3.24643800 | 2.42159900 |
| H | 3.16600700 | -2.77391600 | 1.56278000 |
| C | 1.14796500 | 2.00843700 | -1.84655300 |
| H | 0.13338900 | 2.02905600 | -1.44544000 |
| C | 1.90125700 | -1.10979100 | 2.13734000 |
| H | 0.82606200 | -0.95155100 | 2.25288000 |
| C | -1.79516000 | 1.40143200 | 1.69689700 |
| H | -2.44990600 | 2.27503600 | 1.73200200 |
| H | -1.94099300 | 0.83661600 | 2.62315100 |
| C | 1.57375200 | 3.44008600 | -2.16419600 |
| H | 1.65037200 | 4.05679700 | -1.26726900 |
| H | 0.85210600 | 3.90354600 | -2.83982000 |
| H | 2.54398000 | 3.45573100 | -2.66557500 |
| C | 3.77502100 | -0.17403800 | 0.76987700 |
| H | 4.43931300 | -0.79158600 | 1.36340200 |
| C | 2.59108100 | -0.90495100 | 3.48574500 |
| H | 2.53559400 | 0.12725400 | 3.83295100 |
| H | 3.64692400 | -1.17736600 | 3.41800000 |
| H | 2.13741400 | -1.54923500 | 4.24152700 |
| C | -0.11037200 | 3.18895800 | 0.99154600 |
| H | 0.95009400 | 3.36245800 | 0.79554900 |
| H | -0.44822900 | 3.93074600 | 1.71693600 |
| H | -0.66730300 | 3.33841100 | 0.06700700 |
| C | 0.40653300 | 1.73301300 | 2.91311200 |
| H | 0.26503600 | 0.77036900 | 3.40332500 |
| H | 0.00471200 | 2.50982800 | 3.56621600 |
| H | 1.47576600 | 1.91627800 | 2.78470300 |
| C | -2.09662800 | 0.49009700 | 0.49416000 |
| C | -2.77021400 | 1.23266500 | -0.67055900 |
| C | -2.98776500 | -0.69356300 | 0.87403500 |
| C | -4.22084300 | 1.49524600 | -0.39132200 |

| | | | |
|---|---|---|---|
| H | -2.65157400 | 0.63187200 | -1.58121400 |
| H | -2.25275600 | 2.17881200 | -0.86244400 |
| C | -4.31635400 | -0.19755000 | 1.42990800 |
| H | -3.15225600 | -1.29840800 | -0.02297300 |
| H | -2.46967900 | -1.33059100 | 1.59698900 |
| C | -4.90814400 | 0.87202300 | 0.56170600 |
| H | -4.71337600 | 2.24106700 | -1.00646100 |
| H | -5.01713800 | -1.03160800 | 1.51627400 |
| H | -4.18929200 | 0.18575000 | 2.45035200 |
| H | -5.94955300 | 1.13057600 | 0.72297900 |

60

e-7-1 carbene fragment

| | | | |
|---|---|---|---|
| C | 1.87216400 | 1.47793100 | -0.06936600 |
| N | 0.13742500 | -0.21614700 | 0.26022000 |
| C | -0.30949100 | -0.48473900 | 1.67587300 |
| C | 3.21648600 | 1.78437300 | -0.27051100 |
| H | 3.52012900 | 2.82371500 | -0.32000100 |
| C | -0.77741400 | -0.17525200 | -0.67755000 |
| C | 0.64527700 | 3.07479100 | -1.50828200 |
| H | 0.32737500 | 2.24501500 | -2.14218800 |
| H | 1.57823800 | 3.48242100 | -1.90781900 |
| H | -0.11446500 | 3.85926900 | -1.54629500 |
| C | 2.43191600 | -0.89524300 | -0.26073900 |
| C | 4.16223000 | 0.78525600 | -0.43852200 |
| H | 5.20314400 | 1.04291300 | -0.58977900 |
| C | 1.50726000 | 0.12598300 | -0.01323900 |
| C | 2.01014900 | -2.63140700 | -1.97047700 |
| H | 1.34917900 | -1.94337500 | -2.50085600 |
| H | 1.67859600 | -3.65471000 | -2.16034100 |
| H | 3.02027400 | -2.51863100 | -2.37365900 |
| C | 0.84891400 | 2.59511900 | -0.06971500 |
| H | -0.10590600 | 2.19093600 | 0.26712400 |
| C | 2.00258600 | -2.33206800 | -0.47012000 |
| H | 0.97134000 | -2.43524300 | -0.13085500 |

| | | | |
|---|---|---|---|
| C | -1.72503400 | -0.99292900 | 1.40889900 |
| H | -2.41211300 | -0.68820800 | 2.20107200 |
| H | -1.72212500 | -2.08817800 | 1.37295600 |
| C | 1.22670800 | 3.76003200 | 0.83999100 |
| H | 1.42275300 | 3.43017000 | 1.86224000 |
| H | 0.41799900 | 4.49305800 | 0.86465400 |
| H | 2.11874400 | 4.27463000 | 0.47580500 |
| C | 3.76448500 | -0.54234900 | -0.45926100 |
| H | 4.49446100 | -1.31907400 | -0.65676400 |
| C | 2.86893500 | -3.33322100 | 0.28739000 |
| H | 2.91775000 | -3.09967600 | 1.35282600 |
| H | 3.88966000 | -3.34433400 | -0.10143400 |
| H | 2.46736000 | -4.34209900 | 0.17298100 |
| C | -0.29822400 | 0.80969400 | 2.48364700 |
| H | 0.69944400 | 1.25608600 | 2.48189200 |
| H | -0.57009600 | 0.59021200 | 3.51768400 |
| H | -1.01039500 | 1.53630300 | 2.09125100 |
| C | 0.57205400 | -1.50219900 | 2.38462300 |
| H | 0.53928700 | -2.47825400 | 1.90139600 |
| H | 0.21416000 | -1.62307500 | 3.40888200 |
| H | 1.61006200 | -1.16273300 | 2.42786300 |
| C | -2.08545000 | -0.44324700 | 0.02153800 |
| C | -3.08900000 | -1.28437300 | -0.78580300 |
| C | -2.98952700 | 0.82023800 | -0.01216800 |
| C | -4.34201200 | -1.18083800 | 0.09119500 |
| C | -3.30526800 | -0.43711000 | -2.04404900 |
| H | -2.76687400 | -2.30811900 | -0.98512200 |
| C | -4.29973500 | 0.28772900 | 0.58604700 |
| C | -3.21445800 | 1.01674800 | -1.51518500 |
| H | -2.59070100 | 1.70152500 | 0.49451400 |
| H | -5.24783700 | -1.41495900 | -0.47087600 |
| H | -4.28820800 | -1.87640100 | 0.93239500 |
| H | -2.51927100 | -0.62498500 | -2.77410300 |
| H | -4.26933000 | -0.65869600 | -2.50726300 |

| | | | |
|---|---|---|---|
| H | -5.14921900 | 0.87526300 | 0.23212500 |
| H | -4.30343000 | 0.34102700 | 1.67632400 |
| H | -2.37185200 | 1.53842900 | -1.97036600 |
| H | -4.12142200 | 1.59529200 | -1.70310900 |

61

e-7-1 carbene-Au fragment

| | | | |
|---|---|---|---|
| Au | 0.32224200 | -1.75389200 | 0.74365600 |
| C | -1.85879800 | 1.42109200 | 1.09022800 |
| N | -0.31710500 | 0.73371500 | -0.67239300 |
| C | 0.05450500 | 1.72607800 | -1.73146700 |
| C | -3.15381800 | 1.45646600 | 1.60057500 |
| H | -3.34450500 | 2.00302800 | 2.51693300 |
| C | 0.68861400 | 0.05579000 | -0.15419500 |
| C | -0.47789500 | 1.21876700 | 3.14620100 |
| H | -0.23592400 | 0.18735300 | 2.87791700 |
| H | -1.36129900 | 1.20705900 | 3.79031900 |
| H | 0.35434500 | 1.63809700 | 3.71664100 |
| C | -2.65306400 | -0.05078700 | -0.70104900 |
| C | -4.19126300 | 0.78740400 | 0.97150200 |
| H | -5.19319700 | 0.83177500 | 1.37973300 |
| C | -1.63708500 | 0.70328600 | -0.09618500 |
| C | -2.65018800 | -2.45983700 | -1.34243000 |
| H | -2.02145400 | -2.69338000 | -0.48053500 |
| H | -2.42501000 | -3.17239400 | -2.13882200 |
| H | -3.69695800 | -2.59233000 | -1.05626500 |
| C | -0.74335800 | 2.05860400 | 1.89519800 |
| H | 0.17101400 | 2.04442500 | 1.30038900 |
| C | -2.40919400 | -1.02937000 | -1.83291400 |
| H | -1.35894900 | -0.95915100 | -2.12707600 |
| C | 1.46879800 | 1.25363200 | -2.06410400 |
| H | 2.10324700 | 2.09100500 | -2.35854100 |
| H | 1.43053300 | 0.54695200 | -2.90024100 |
| C | -1.03983000 | 3.50568300 | 2.28166100 |
| H | -1.27168300 | 4.12247200 | 1.41158900 |

| | | | |
|---|---|---|---|
| H | -0.17775200 | 3.94024700 | 2.79175300 |
| H | -1.88641700 | 3.56307300 | 2.96931700 |
| C | -3.93264700 | 0.02461000 | -0.15474600 |
| H | -4.73205100 | -0.54868600 | -0.61016300 |
| C | -3.30358400 | -0.76340500 | -3.04414000 |
| H | -3.23109100 | 0.26170300 | -3.40711600 |
| H | -4.34927200 | -0.95309500 | -2.79006000 |
| H | -3.04027300 | -1.43877300 | -3.86074400 |
| C | 0.02096700 | 3.14225400 | -1.16402200 |
| H | -0.97385300 | 3.36965700 | -0.77406300 |
| H | 0.24346100 | 3.85096500 | -1.96345200 |
| H | 0.75097800 | 3.28491700 | -0.36790900 |
| C | -0.87896400 | 1.66977500 | -2.92754700 |
| H | -0.87415100 | 0.68788300 | -3.39918900 |
| H | -0.54663500 | 2.40373100 | -3.66416200 |
| H | -1.90039900 | 1.92269600 | -2.63484300 |
| C | 1.96014100 | 0.51745500 | -0.81065400 |
| C | 3.06051100 | -0.53328100 | -1.05020500 |
| C | 2.85240800 | 1.35477600 | 0.15256300 |
| C | 4.16815700 | 0.35534800 | -1.62573800 |
| C | 3.50454500 | -0.94448200 | 0.36222000 |
| H | 2.75978300 | -1.36574900 | -1.68813800 |
| C | 4.09166100 | 1.61341600 | -0.72181900 |
| C | 3.23801500 | 0.32187200 | 1.21113500 |
| H | 2.39713300 | 2.25922400 | 0.55809700 |
| H | 5.13975600 | -0.13838200 | -1.58434500 |
| H | 3.97198300 | 0.60927400 | -2.67000400 |
| H | 2.93440700 | -1.79713700 | 0.73498200 |
| H | 4.55998300 | -1.22640400 | 0.35779700 |
| H | 4.97691900 | 1.72822000 | -0.09330700 |
| H | 3.99637500 | 2.52673400 | -1.31159200 |
| H | 2.41316200 | 0.15085400 | 1.90814900 |
| H | 4.10865400 | 0.64471500 | 1.78444600 |

67

e-8-1 carbene fragment

| | | | |
|---|---|---|---|
| C | -2.72257600 | -1.15989000 | -0.39589500 |
| N | -0.82155100 | 0.09864800 | 0.47109200 |
| C | -0.67579100 | 0.13735900 | 1.96579500 |
| C | -4.00681300 | -1.16934000 | -0.93310800 |
| H | -4.48516900 | -2.11814000 | -1.14682000 |
| C | 0.25128700 | 0.07883500 | -0.27536100 |
| C | -1.45613100 | -2.89409700 | -1.63230800 |
| H | -0.83244800 | -2.11471900 | -2.07484000 |
| H | -2.30117100 | -3.08613600 | -2.29959600 |
| H | -0.86412000 | -3.80908200 | -1.55077700 |
| C | -2.73724600 | 1.28591300 | -0.50067100 |
| C | -4.66617800 | 0.01536200 | -1.22565300 |
| H | -5.66706500 | -0.00877700 | -1.63851200 |
| C | -2.12819500 | 0.08111000 | -0.13367200 |
| C | 1.43810600 | 0.01108900 | 0.69038400 |
| C | -1.59124300 | 2.95722400 | -1.91837400 |
| H | -0.97526000 | 2.16285600 | -2.34339700 |
| H | -1.02291700 | 3.88957800 | -1.93794600 |
| H | -2.47875200 | 3.08218900 | -2.54470800 |
| C | -1.95360000 | -2.45735700 | -0.25308600 |
| H | -1.06426200 | -2.26913000 | 0.34807000 |
| C | -1.99579300 | 2.60709300 | -0.48490600 |
| H | -1.07160800 | 2.47980900 | 0.07989500 |
| C | 0.84976300 | 0.04464200 | 2.11732600 |
| H | 1.12392200 | -0.83933400 | 2.68834600 |
| H | 1.22448900 | 0.88750800 | 2.69221700 |
| C | -2.75877000 | -3.56557300 | 0.41618800 |
| H | -3.15141300 | -3.24939600 | 1.38518700 |
| H | -2.12878800 | -4.44419700 | 0.56936000 |
| H | -3.60257100 | -3.87608300 | -0.20412100 |
| C | -4.02347400 | 1.22811200 | -1.03468500 |
| H | -4.51460500 | 2.14858300 | -1.32913700 |
| C | -2.79916100 | 3.74116700 | 0.14342300 |

| | | | |
|---|---|---|---|
| H | -3.12204300 | 3.49675300 | 1.15729600 |
| H | -3.68975400 | 3.96936500 | -0.44647100 |
| H | -2.19520200 | 4.64968100 | 0.18490100 |
| C | -1.41351600 | -1.01333900 | 2.64143200 |
| H | -2.47384600 | -1.00578600 | 2.37712000 |
| H | -1.33358500 | -0.89432500 | 3.72356900 |
| H | -0.99480000 | -1.98467600 | 2.37817900 |
| C | -1.23458200 | 1.44556100 | 2.51459700 |
| H | -0.69544000 | 2.30940600 | 2.12317100 |
| H | -1.13815000 | 1.44627200 | 3.60195600 |
| H | -2.29371400 | 1.54895100 | 2.26600100 |
| C | 2.32862400 | 1.19724000 | 0.33075300 |
| C | 2.31362900 | 2.40156300 | 1.03295700 |
| C | 3.12889700 | 1.10901100 | -0.81260700 |
| C | 3.10780800 | 3.47474700 | 0.64654200 |
| H | 1.67588600 | 2.52976400 | 1.89775100 |
| C | 3.93740300 | 2.17740900 | -1.18349500 |
| C | 3.93707700 | 3.36061600 | -0.45885200 |
| H | 3.07780000 | 4.39504400 | 1.21626400 |
| H | 4.56572000 | 2.07714900 | -2.06203300 |
| H | 4.57024400 | 4.18583700 | -0.75935200 |
| C | 2.09042500 | -1.31997900 | 0.30970400 |
| C | 1.83562200 | -2.51174000 | 0.98721200 |
| C | 2.88432200 | -1.36903800 | -0.84028600 |
| C | 2.38992000 | -3.71524700 | 0.56810200 |
| H | 1.19024800 | -2.52587500 | 1.85592600 |
| C | 3.45270000 | -2.57165700 | -1.24495200 |
| C | 3.21383500 | -3.74680900 | -0.54725000 |
| H | 2.17835200 | -4.62310400 | 1.11902300 |
| H | 4.08020300 | -2.58190200 | -2.12966200 |
| H | 3.65954100 | -4.67726900 | -0.87551100 |
| C | 3.06410300 | -0.12664400 | -1.65779000 |
| H | 3.94854500 | -0.20652500 | -2.29384600 |
| H | 2.18988800 | -0.03488700 | -2.31392100 |

68
e-8-1 carbene-Au fragment

| | | | |
|---|---|---|---|
| Au | -0.31812800 | -0.00009300 | -1.74839300 |
| C | 2.84185900 | 1.22706400 | 0.01461500 |
| N | 0.95531000 | 0.00022800 | 0.98720900 |
| C | 0.83595600 | 0.00032200 | 2.48322700 |
| C | 4.12034100 | 1.19951200 | -0.53723400 |
| H | 4.60040200 | 2.13570400 | -0.79799000 |
| C | -0.15626000 | 0.00023500 | 0.30877700 |
| C | 1.82168200 | 3.08137600 | -1.29230200 |
| H | 1.24831800 | 2.35447200 | -1.87277000 |
| H | 2.75396200 | 3.28910600 | -1.82435600 |
| H | 1.24823200 | 4.00904000 | -1.23339700 |
| C | 2.84263100 | -1.22587000 | 0.01522500 |
| C | 4.76869600 | 0.00098400 | -0.78725400 |
| H | 5.76334900 | 0.00118400 | -1.21512100 |
| C | 2.24673900 | 0.00049400 | 0.33856700 |
| C | -1.33854000 | -0.00015600 | 1.26062700 |
| C | 1.82293100 | -3.08076300 | -1.29110100 |
| H | 1.24911600 | -2.35417000 | -1.87151900 |
| H | 1.24991300 | -4.00867900 | -1.23196800 |
| H | 2.75518600 | -3.28810000 | -1.82335400 |
| C | 2.11766200 | 2.55503500 | 0.11403600 |
| H | 1.15408800 | 2.39327700 | 0.60173100 |
| C | 2.11903900 | -2.55413500 | 0.11510900 |
| H | 1.15553700 | -2.39265700 | 0.60302600 |
| C | -0.69308500 | -0.00008600 | 2.67181400 |
| H | -1.00758500 | 0.87074300 | 3.24386800 |
| H | -1.00717200 | -0.87115100 | 3.24374000 |
| C | 2.90499300 | 3.60060300 | 0.90168400 |
| H | 3.17831400 | 3.25341500 | 1.89914900 |
| H | 2.31562400 | 4.51420800 | 1.00322700 |
| H | 3.82569600 | 3.86465700 | 0.37626700 |
| C | 4.12110000 | -1.19783300 | -0.53658400 |

| | | | |
|---|---|---|---|
| H | 4.60175200 | -2.13385700 | -0.79685200 |
| C | 2.90693000 | -3.59926400 | 0.90274000 |
| H | 3.18015400 | -3.25186500 | 1.90016300 |
| H | 3.82771500 | -3.86296900 | 0.37729400 |
| H | 2.31797700 | -4.51312100 | 1.00441200 |
| C | 1.51521100 | 1.22614900 | 3.08127100 |
| H | 2.58079600 | 1.22523400 | 2.84170800 |
| H | 1.41040100 | 1.18630400 | 4.16675700 |
| H | 1.08235200 | 2.16137800 | 2.73020000 |
| C | 1.51602700 | -1.22479400 | 3.08175500 |
| H | 1.08360900 | -2.16050800 | 2.73143200 |
| H | 1.41150200 | -1.18433800 | 4.16724900 |
| H | 2.58154900 | -1.22341600 | 2.84192000 |
| C | -2.12385200 | -1.27301500 | 0.95043100 |
| C | -1.71096800 | -2.49956800 | 1.47505100 |
| C | -3.20428800 | -1.25690200 | 0.06933200 |
| C | -2.37059800 | -3.68007700 | 1.17283800 |
| H | -0.85366800 | -2.54333800 | 2.13455400 |
| C | -3.87764800 | -2.44442800 | -0.21426900 |
| C | -3.47380000 | -3.65170100 | 0.32909900 |
| H | -2.02656500 | -4.61458600 | 1.59745900 |
| H | -4.72628100 | -2.40962700 | -0.88880500 |
| H | -4.00730300 | -4.56308800 | 0.09112300 |
| C | -2.12482600 | 1.27213800 | 0.95069500 |
| C | -1.71294500 | 2.49890100 | 1.47561700 |
| C | -3.20527400 | 1.25535300 | 0.06962000 |
| C | -2.37355100 | 3.67894200 | 1.17370600 |
| H | -0.85568900 | 2.54323500 | 2.13514100 |
| C | -3.87960800 | 2.44239800 | -0.21368000 |
| C | -3.47674100 | 3.64987000 | 0.32997400 |
| H | -2.03028500 | 4.61362600 | 1.59856400 |
| H | -4.72822900 | 2.40707000 | -0.88820400 |
| H | -4.01100600 | 4.56087100 | 0.09223100 |
| C | -3.61867800 | -0.00086100 | -0.63024200 |

| | | | |
|---|---|---|---|
| H | -4.69866100 | -0.00128000 | -0.79917200 |
| H | -3.14497800 | -0.00055700 | -1.62396800 |

64

e-9-1 carbene fragment

| | | | |
|---|---|---|---|
| C | -2.76824100 | -1.22290600 | -0.43208700 |
| N | -0.83117800 | 0.00000800 | 0.43904300 |
| C | -0.65233300 | 0.00001500 | 1.93356800 |
| C | -4.06689000 | -1.19891600 | -0.93527400 |
| H | -4.55768100 | -2.13474800 | -1.17642200 |
| C | 0.22404300 | 0.00001200 | -0.33657200 |
| C | -1.59424000 | -2.92967600 | -1.78757000 |
| H | -0.97672500 | -2.14682500 | -2.23149100 |
| H | -2.47473200 | -3.08088900 | -2.41836800 |
| H | -1.02045600 | -3.85935500 | -1.77034600 |
| C | -2.76823500 | 1.22291300 | -0.43209900 |
| C | -4.72433700 | 0.00000400 | -1.16177600 |
| H | -5.73521100 | 0.00000400 | -1.55016800 |
| C | -2.15494500 | 0.00000400 | -0.13322700 |
| C | -1.59429300 | 2.92970100 | -1.78761300 |
| H | -0.97680700 | 2.14685400 | -2.23158000 |
| H | -1.02050200 | 3.85937600 | -1.77040200 |
| H | -2.47481600 | 3.08093200 | -2.41836400 |
| C | -2.01709300 | -2.53580700 | -0.37123700 |
| H | -1.10069900 | -2.37843600 | 0.19568900 |
| C | -2.01707800 | 2.53581000 | -0.37126600 |
| H | -1.10065600 | 2.37842300 | 0.19561100 |
| C | 0.87963800 | 0.00001500 | 2.04863600 |
| H | 1.22491200 | -0.88049500 | 2.59346400 |
| H | 1.22491100 | 0.88053200 | 2.59345200 |
| C | -2.81016900 | -3.65526800 | 0.29471200 |
| H | -3.14957600 | -3.37212000 | 1.29329900 |
| H | -2.19253200 | -4.55142400 | 0.38232000 |
| H | -3.68859500 | -3.92459200 | -0.29590700 |
| C | -4.06688500 | 1.19892300 | -0.93528500 |

| | | | |
|---|---|---|---|
| H | -4.55767100 | 2.13475400 | -1.17644600 |
| C | -2.81011200 | 3.65526400 | 0.29474400 |
| H | -3.14947000 | 3.37210100 | 1.29334300 |
| H | -3.68856600 | 3.92460500 | -0.29582500 |
| H | -2.19246400 | 4.55141300 | 0.38233600 |
| C | -1.28459600 | -1.23607100 | 2.56274300 |
| H | -2.34854900 | -1.29827200 | 2.32119600 |
| H | -1.18495900 | -1.17112200 | 3.64762400 |
| H | -0.79317900 | -2.15345700 | 2.23554400 |
| C | -1.28459000 | 1.23611100 | 2.56273100 |
| H | -0.79317300 | 2.15349100 | 2.23551700 |
| H | -1.18494300 | 1.17117600 | 3.64761200 |
| H | -2.34854400 | 1.29831400 | 2.32119100 |
| C | 1.42776900 | 0.00000600 | 0.60539600 |
| C | 2.30165300 | 1.17102100 | 0.21912300 |
| C | 2.30163000 | -1.17102800 | 0.21912700 |
| C | 2.04190600 | 2.52142700 | 0.37041500 |
| C | 3.48064400 | 0.73222800 | -0.39304700 |
| C | 2.04185600 | -2.52142900 | 0.37042300 |
| C | 3.48062900 | -0.73226000 | -0.39304500 |
| C | 2.97731900 | 3.44137300 | -0.09598900 |
| H | 1.12711300 | 2.85940300 | 0.84875700 |
| C | 4.41137900 | 1.64958100 | -0.86439200 |
| C | 2.97725100 | -3.44139500 | -0.09598000 |
| H | 1.12705600 | -2.85938500 | 0.84876600 |
| C | 4.41134600 | -1.64963400 | -0.86438900 |
| C | 4.14974100 | 3.00625700 | -0.71071600 |
| H | 2.79386100 | 4.50251500 | 0.01683800 |
| H | 5.32596600 | 1.31675600 | -1.34150500 |
| C | 4.14968100 | -3.00630400 | -0.71070900 |
| H | 2.79377200 | -4.50253300 | 0.01685100 |
| H | 5.32593800 | -1.31682700 | -1.34150300 |
| H | 4.86529400 | 3.73517800 | -1.07022800 |
| H | 4.86522000 | -3.73524000 | -1.07021900 |

65
e-9-1 carbene-Au fragment

| | | | |
|---|---|---|---|
| Au | 0.35792300 | 0.00079900 | -1.71082100 |
| C | -2.85536800 | -1.22594500 | 0.03558200 |
| N | -0.96980200 | -0.00020900 | 1.01544300 |
| C | -0.85294900 | -0.00049600 | 2.51325200 |
| C | -4.13279300 | -1.19822000 | -0.51859300 |
| H | -4.61198700 | -2.13433900 | -0.78099800 |
| C | 0.14521100 | -0.00004700 | 0.34150500 |
| C | -1.82572900 | -3.06957600 | -1.27666700 |
| H | -1.24680900 | -2.33834600 | -1.84614400 |
| H | -2.75427200 | -3.27142800 | -1.81737600 |
| H | -1.25474900 | -3.99912700 | -1.22201300 |
| C | -2.85536500 | 1.22576200 | 0.03582500 |
| C | -4.78086100 | -0.00002200 | -0.76975400 |
| H | -5.77463000 | 0.00001500 | -1.19959600 |
| C | -2.25943100 | -0.00012500 | 0.36212100 |
| C | -1.82595800 | 3.06989400 | -1.27596000 |
| H | -1.24710200 | 2.33885100 | -1.84574400 |
| H | -1.25495000 | 3.99941200 | -1.22104400 |
| H | -2.75455200 | 3.27196400 | -1.81650000 |
| C | -2.13263500 | -2.55460400 | 0.13153400 |
| H | -1.17422600 | -2.39302200 | 0.62809600 |
| C | -2.13269500 | 2.55443500 | 0.13210300 |
| H | -1.17421800 | 2.39274300 | 0.62849300 |
| C | 0.67334400 | -0.00067400 | 2.71118900 |
| H | 0.98648600 | -0.88197500 | 3.27300800 |
| H | 0.98667600 | 0.88025200 | 3.27349200 |
| C | -2.92277000 | -3.60604300 | 0.90812700 |
| H | -3.19646000 | -3.26774000 | 1.90853000 |
| H | -2.33528800 | -4.52161500 | 1.00240100 |
| H | -3.84295600 | -3.86434500 | 0.37918900 |
| C | -4.13279100 | 1.19812700 | -0.51836600 |
| H | -4.61199600 | 2.13429700 | -0.78056400 |

| | | | |
|---|---|---|---|
| C | -2.92281700 | 3.60557100 | 0.90913600 |
| H | -3.19634600 | 3.26692700 | 1.90946600 |
| H | -3.84309900 | 3.86395300 | 0.38040500 |
| H | -2.33540800 | 4.52116800 | 1.00362500 |
| C | -1.51543800 | -1.23688300 | 3.10248400 |
| H | -2.57524800 | -1.26969100 | 2.84145300 |
| H | -1.43178200 | -1.19539100 | 4.18968900 |
| H | -1.03630700 | -2.15490400 | 2.75924900 |
| C | -1.51524700 | 1.23578100 | 3.10293100 |
| H | -1.03595200 | 2.15385300 | 2.76006500 |
| H | -1.43164000 | 1.19385700 | 4.19012500 |
| H | -2.57503900 | 1.26886500 | 2.84187000 |
| C | 1.30831000 | -0.00039600 | 1.30768800 |
| C | 2.19547000 | 1.17412000 | 0.95246700 |
| C | 2.19515700 | -1.17496100 | 0.95189200 |
| C | 1.92744600 | 2.52277800 | 1.09719700 |
| C | 3.38486000 | 0.73192800 | 0.36514200 |
| C | 1.92672200 | -2.52361100 | 1.09590900 |
| C | 3.38466200 | -0.73280900 | 0.36478000 |
| C | 2.87109700 | 3.44222500 | 0.64724000 |
| H | 1.00119000 | 2.86043700 | 1.55322700 |
| C | 4.32231700 | 1.64940600 | -0.09012400 |
| C | 2.87011400 | -3.44310200 | 0.64549500 |
| H | 1.00033500 | -2.86122200 | 1.55171900 |
| C | 4.32186200 | -1.65032500 | -0.09094000 |
| C | 4.05456100 | 3.00572100 | 0.05582800 |
| H | 2.68388400 | 4.50326200 | 0.75200600 |
| H | 5.24375800 | 1.31729500 | -0.55338400 |
| C | 4.05371900 | -3.00663700 | 0.05433300 |
| H | 2.68258600 | -4.50413900 | 0.74970400 |
| H | 5.24340000 | -1.31824400 | -0.55402900 |
| H | 4.77419500 | 3.73460200 | -0.29498500 |
| H | 4.77314800 | -3.73554700 | -0.29683900 |

59

e-10-1 carbene fragment

| | | | |
|---|---|---|---|
| C | 1.48797800 | 1.79030100 | 0.06808500 |
| N | 0.39142700 | -0.40438900 | 0.11170900 |
| C | -0.06653100 | -0.91308500 | 1.45723200 |
| C | 2.67114000 | 2.52654100 | 0.03114800 |
| H | 2.62187000 | 3.60855300 | 0.07150600 |
| C | -0.34230000 | -0.68342900 | -0.93418100 |
| C | -0.07046400 | 3.00471600 | -1.41899700 |
| H | -0.01646400 | 2.17208800 | -2.12367300 |
| H | 0.69238800 | 3.73850900 | -1.69406800 |
| H | -1.05097200 | 3.48036200 | -1.50720100 |
| C | 2.80755000 | -0.25572000 | -0.18215400 |
| C | 3.90097300 | 1.89956800 | -0.08696200 |
| H | 4.80988600 | 2.48782900 | -0.11095500 |
| C | 1.58272500 | 0.39551700 | 0.00939100 |
| C | -1.49278800 | -1.54259300 | -0.41026100 |
| C | 3.05243800 | -1.91495900 | -2.00150900 |
| H | 2.21594900 | -1.45657100 | -2.53247100 |
| H | 3.09707900 | -2.97511300 | -2.26124200 |
| H | 3.97830100 | -1.44041600 | -2.33832700 |
| C | 0.15784700 | 2.51102700 | 0.01088000 |
| H | -0.63423800 | 1.79602800 | 0.23132400 |
| C | 2.88945500 | -1.73666100 | -0.49088100 |
| H | 1.94041200 | -2.20198300 | -0.21638500 |
| C | -1.02708100 | -1.99888600 | 0.98885900 |
| H | -1.83849800 | -2.15737200 | 1.70053200 |
| H | -0.47673200 | -2.94058600 | 0.88441900 |
| C | 0.05053500 | 3.66454400 | 1.00303700 |
| H | 0.26460800 | 3.34233200 | 2.02411900 |
| H | -0.95691100 | 4.08431300 | 0.97983600 |
| H | 0.74466900 | 4.47006800 | 0.75262900 |
| C | 3.96278300 | 0.52023800 | -0.21749800 |
| H | 4.92157500 | 0.03838100 | -0.36963000 |
| C | 4.01032100 | -2.44352000 | 0.26485400 |

| | | | |
|---|---|---|---|
| H | 3.95084000 | -2.25847800 | 1.33957200 |
| H | 4.99130600 | -2.10906000 | -0.07965600 |
| H | 3.95811600 | -3.52065000 | 0.09491700 |
| C | -0.77068200 | 0.19967300 | 2.22728000 |
| H | -0.08658500 | 1.03344100 | 2.40519600 |
| H | -1.09255400 | -0.18619200 | 3.19637100 |
| H | -1.64997400 | 0.56503600 | 1.69430900 |
| C | 1.07733400 | -1.44441500 | 2.30741300 |
| H | 1.58232500 | -2.28751300 | 1.83717900 |
| H | 0.67147300 | -1.78633300 | 3.26142800 |
| H | 1.81209600 | -0.66283900 | 2.51417600 |
| C | -2.77332600 | -0.71602100 | -0.36980500 |
| C | -3.89111300 | -1.20305500 | 0.30951700 |
| C | -2.88252900 | 0.51110900 | -1.02414000 |
| C | -5.07374600 | -0.47840300 | 0.35771400 |
| H | -3.83738300 | -2.16675800 | 0.80523500 |
| C | -4.06470600 | 1.24244600 | -0.97257700 |
| H | -2.03515700 | 0.88644600 | -1.58564000 |
| C | -5.16300700 | 0.75492900 | -0.27786200 |
| H | -5.92712900 | -0.87685300 | 0.89266500 |
| H | -4.12490200 | 2.19587200 | -1.48383100 |
| H | -6.08271600 | 1.32484900 | -0.23715400 |
| C | -1.69351000 | -2.73542400 | -1.34677800 |
| H | -2.00459800 | -2.39200700 | -2.33349800 |
| H | -0.75735900 | -3.29050200 | -1.45410900 |
| H | -2.45647400 | -3.41078800 | -0.95036400 |

60

e-10-1 carbene-Au fragment

| | | | |
|---|---|---|---|
| Au | 0.34708900 | -1.88163000 | -0.88462700 |
| C | 1.09019200 | 1.96186600 | -1.03631900 |
| N | 0.31377700 | 0.56041300 | 0.81402400 |
| C | -0.19732700 | 1.22694000 | 2.05891300 |
| C | 2.14838600 | 2.54515200 | -1.72891800 |
| H | 1.93226500 | 3.19818200 | -2.56658300 |

| | | | |
|---|---|---|---|
| C | -0.39703600 | -0.45950100 | 0.39391000 |
| C | -0.50472700 | 1.39011900 | -2.85190900 |
| H | -0.26598700 | 0.33210100 | -2.71335000 |
| H | 0.15289600 | 1.78775800 | -3.62966500 |
| H | -1.53754500 | 1.47595700 | -3.20040900 |
| C | 2.71841500 | 0.78016600 | 0.36376900 |
| C | 3.46370300 | 2.28424100 | -1.38152100 |
| H | 4.27318400 | 2.74985600 | -1.92960500 |
| C | 1.39634200 | 1.11773500 | 0.04149900 |
| C | -1.55074400 | -0.68139100 | 1.35850500 |
| C | 3.71996100 | -1.48429100 | 0.66437500 |
| H | 3.04850200 | -1.89505800 | -0.09290000 |
| H | 3.95267000 | -2.27058400 | 1.38569700 |
| H | 4.65178700 | -1.18648600 | 0.17638900 |
| C | -0.32004000 | 2.16882900 | -1.54838900 |
| H | -1.02082700 | 1.75391700 | -0.82438600 |
| C | 3.08756900 | -0.28569800 | 1.37629300 |
| H | 2.17114100 | -0.63822000 | 1.85764200 |
| C | -1.09668100 | 0.11734100 | 2.59427600 |
| H | -1.91153000 | 0.51621100 | 3.19741800 |
| H | -0.49961300 | -0.54663100 | 3.22770500 |
| C | -0.67159200 | 3.63933900 | -1.75960600 |
| H | -0.50105500 | 4.23502500 | -0.86113600 |
| H | -1.72314500 | 3.73243400 | -2.03856000 |
| H | -0.07918800 | 4.07400900 | -2.56794800 |
| C | 3.74007900 | 1.39268100 | -0.35829800 |
| H | 4.76927700 | 1.14533600 | -0.12471600 |
| C | 4.05440300 | 0.23450600 | 2.44059700 |
| H | 3.69626500 | 1.14368200 | 2.92441200 |
| H | 5.02592700 | 0.45699000 | 1.99303600 |
| H | 4.21631300 | -0.52632300 | 3.20683300 |
| C | -0.96744200 | 2.49206300 | 1.69339300 |
| H | -0.31420400 | 3.19006500 | 1.16467900 |
| H | -1.31040500 | 2.97494300 | 2.61009100 |

| | | | |
|---|---|---|---|
| H | -1.83816600 | 2.27721400 | 1.07300300 |
| C | 0.91905600 | 1.59370000 | 3.01980300 |
| H | 1.45767200 | 0.71400800 | 3.36937200 |
| H | 0.48247400 | 2.09201000 | 3.88720500 |
| H | 1.62564400 | 2.28398000 | 2.55316500 |
| C | -2.83919800 | -0.15938300 | 0.71885800 |
| C | -3.92058300 | 0.21875300 | 1.51465900 |
| C | -2.99404600 | -0.12092600 | -0.66751000 |
| C | -5.10798600 | 0.65999200 | 0.94578700 |
| H | -3.84479400 | 0.16244300 | 2.59443200 |
| C | -4.17931400 | 0.32808800 | -1.23888700 |
| H | -2.18148800 | -0.45218200 | -1.30495100 |
| C | -5.23858500 | 0.72841100 | -0.43585000 |
| H | -5.93272800 | 0.95021900 | 1.58482600 |
| H | -4.27290000 | 0.35566400 | -2.31781300 |
| H | -6.16133700 | 1.07891500 | -0.88047600 |
| C | -1.73889300 | -2.16424100 | 1.68160000 |
| H | -2.02737300 | -2.71283900 | 0.78397800 |
| H | -0.81016300 | -2.59589600 | 2.06163100 |
| H | -2.52351000 | -2.27773700 | 2.43417300 |

68

e-11-1 carbene fragment

| | | | |
|---|---|---|---|
| C | 2.62096200 | 1.59203800 | 0.07933600 |
| N | 0.87457200 | -0.15350900 | 0.18949400 |
| C | 0.38629200 | -0.54624500 | 1.55649100 |
| C | 3.97308300 | 1.90617700 | -0.05657400 |
| H | 4.28142100 | 2.94395100 | -0.00486900 |
| C | 0.03198700 | -0.27119300 | -0.80774600 |
| C | 1.42142900 | 3.31695700 | -1.22420200 |
| H | 1.12700400 | 2.54615200 | -1.93979100 |
| H | 2.35696500 | 3.76774800 | -1.56687800 |
| H | 0.65145600 | 4.09232100 | -1.20779700 |
| C | 3.17941000 | -0.76047200 | -0.27932200 |
| C | 4.91851900 | 0.92028000 | -0.27967900 |

| | | | |
|---|---|---|---|
| H | 5.96456500 | 1.18224500 | -0.37892500 |
| C | 2.24467500 | 0.24901100 | -0.00346400 |
| C | -1.17062900 | -1.06865200 | -0.26667500 |
| C | 2.60568000 | -2.37039900 | -2.07180900 |
| H | 1.88294600 | -1.65793800 | -2.47519000 |
| H | 2.27210800 | -3.38450300 | -2.30663100 |
| H | 3.56885900 | -2.20391200 | -2.56226900 |
| C | 1.60730500 | 2.70981500 | 0.16697300 |
| H | 0.65479600 | 2.27640000 | 0.46576400 |
| C | 2.75413700 | -2.18749900 | -0.56031700 |
| H | 1.76943400 | -2.34913200 | -0.11435700 |
| C | -0.73256700 | -1.50054900 | 1.16474000 |
| H | -1.53969500 | -1.45060800 | 1.89512800 |
| H | -0.34981400 | -2.52834500 | 1.14394000 |
| C | 1.97608300 | 3.78064400 | 1.18866500 |
| H | 2.15932000 | 3.34845500 | 2.17499800 |
| H | 1.16802700 | 4.50929900 | 1.27653900 |
| H | 2.87399800 | 4.32551000 | 0.88910800 |
| C | 4.51748900 | -0.40165700 | -0.40707100 |
| H | 5.25438300 | -1.16558600 | -0.62406800 |
| C | 3.70789300 | -3.22807600 | 0.01848300 |
| H | 3.89919700 | -3.05541200 | 1.08002400 |
| H | 4.66801100 | -3.22111500 | -0.50185400 |
| H | 3.28556300 | -4.22765100 | -0.09943100 |
| C | -0.17459300 | 0.68306500 | 2.26431900 |
| H | 0.61030000 | 1.41751500 | 2.46072600 |
| H | -0.60501500 | 0.38337600 | 3.22176300 |
| H | -0.96275700 | 1.14658700 | 1.66345700 |
| C | 1.46034000 | -1.17686100 | 2.42719200 |
| H | 1.88552100 | -2.07365600 | 1.97635300 |
| H | 1.01256200 | -1.45926400 | 3.38208000 |
| H | 2.26813100 | -0.46976400 | 2.62776700 |
| C | -2.56330300 | -0.42034300 | -0.27597700 |
| C | -3.65604000 | -1.16628900 | 0.21533200 |

| | | | |
|---|---|---|---|
| C | -2.82563900 | 0.87563500 | -0.76879600 |
| C | -4.93405100 | -0.60840800 | 0.23478000 |
| C | -4.12261000 | 1.38387400 | -0.73565100 |
| C | -5.19740700 | 0.66731400 | -0.23133800 |
| H | -5.75101800 | -1.20948200 | 0.62567300 |
| H | -4.29146400 | 2.38502100 | -1.12150400 |
| C | -1.16743700 | -2.25727500 | -1.25381900 |
| H | -1.40636100 | -1.89373600 | -2.25323400 |
| H | -0.17189300 | -2.70942300 | -1.29361100 |
| H | -1.88240200 | -3.02971800 | -0.97713300 |
| C | -3.60533400 | -2.59759900 | 0.70807900 |
| H | -4.25865900 | -2.70696800 | 1.57542600 |
| H | -3.98726100 | -3.26937600 | -0.06542200 |
| H | -2.62110100 | -2.95740800 | 0.98628400 |
| C | -6.57628300 | 1.25991000 | -0.17506400 |
| H | -6.78147400 | 1.86540200 | -1.05889200 |
| H | -7.33679700 | 0.48145500 | -0.10777800 |
| H | -6.68784200 | 1.90789900 | 0.69751600 |
| C | -1.77823500 | 1.78765000 | -1.34881200 |
| H | -0.98337900 | 1.99329400 | -0.63125800 |
| H | -1.29369600 | 1.35884700 | -2.22434700 |
| H | -2.23287800 | 2.74097700 | -1.62221300 |

69

e-11-1 carbene-Au fragment

| | | | |
|---|---|---|---|
| Au | 0.57060100 | 0.30272200 | -1.97803600 |
| C | 2.39426200 | 1.46449000 | 1.09903800 |
| N | 0.65811900 | -0.28628800 | 0.94242000 |
| C | 0.04730200 | -0.85015700 | 2.19160300 |
| C | 3.74016000 | 1.81164100 | 0.99855600 |
| H | 4.02915900 | 2.84399900 | 1.15837300 |
| C | -0.14032100 | -0.25826000 | -0.09434700 |
| C | 1.26590600 | 3.47204200 | 0.14526100 |
| H | 1.01883700 | 2.89970800 | -0.75348400 |
| H | 2.21256800 | 3.98904300 | -0.03221300 |

| | | | |
|---|---|---|---|
| H | 0.49070000 | 4.22504800 | 0.30707700 |
| C | 2.99378000 | -0.83770800 | 0.51784900 |
| C | 4.70152900 | 0.87078500 | 0.67443200 |
| H | 5.74160600 | 1.16070500 | 0.59446100 |
| C | 2.04073100 | 0.13183700 | 0.86816500 |
| C | -1.37709600 | -1.11159500 | 0.20182600 |
| C | 2.83165800 | -2.52663100 | -1.32732200 |
| H | 2.22008500 | -1.84905200 | -1.92873100 |
| H | 2.56358600 | -3.55749000 | -1.57131700 |
| H | 3.87747800 | -2.37335900 | -1.60657500 |
| C | 1.38063600 | 2.55454500 | 1.36207700 |
| H | 0.41173800 | 2.08304300 | 1.51416900 |
| C | 2.64596700 | -2.27241600 | 0.16916100 |
| H | 1.59195600 | -2.44348600 | 0.40651200 |
| C | -1.02562100 | -1.74225400 | 1.58118800 |
| H | -1.88366400 | -1.80516200 | 2.24863300 |
| H | -0.62388000 | -2.75129900 | 1.43318500 |
| C | 1.70853200 | 3.35663900 | 2.61942900 |
| H | 1.82597400 | 2.70947800 | 3.49176400 |
| H | 0.91344100 | 4.07556400 | 2.82614500 |
| H | 2.63624700 | 3.91945600 | 2.49552800 |
| C | 4.32434900 | -0.44027100 | 0.43105700 |
| H | 5.07375500 | -1.17056500 | 0.14875900 |
| C | 3.49081000 | -3.27137800 | 0.96253300 |
| H | 3.49000600 | -3.05873200 | 2.03297000 |
| H | 4.52839900 | -3.25002400 | 0.62245800 |
| H | 3.11965700 | -4.28622800 | 0.80673000 |
| C | -0.58333600 | 0.27796700 | 3.00218300 |
| H | 0.17520100 | 0.97220600 | 3.36950300 |
| H | -1.09678000 | -0.15104700 | 3.86430400 |
| H | -1.31768100 | 0.82508500 | 2.40486900 |
| C | 1.04275300 | -1.59663100 | 3.05917500 |
| H | 1.45386600 | -2.46708100 | 2.55180300 |
| H | 0.52768500 | -1.94120100 | 3.95756000 |

| | | | |
|---|---|---|---|
| H | 1.86310200 | -0.94462800 | 3.36697100 |
| C | -2.77048200 | -0.45819800 | 0.20687200 |
| C | -3.88464900 | -1.28447400 | 0.47932100 |
| C | -3.01342500 | 0.90914600 | -0.03856500 |
| C | -5.16774000 | -0.74221200 | 0.50254700 |
| C | -4.31948800 | 1.39815200 | -0.00864800 |
| C | -5.41771600 | 0.59652600 | 0.25339500 |
| H | -6.00032200 | -1.40390800 | 0.72565100 |
| H | -4.47302100 | 2.45681600 | -0.19459900 |
| C | -1.30087600 | -2.15337400 | -0.93913500 |
| H | -1.53057200 | -1.67062400 | -1.88954100 |
| H | -0.28619400 | -2.55779500 | -1.01245400 |
| H | -1.98821200 | -2.97955800 | -0.77899700 |
| C | -3.85076000 | -2.77775700 | 0.73195400 |
| H | -4.61221400 | -3.03420000 | 1.46979500 |
| H | -4.09952900 | -3.31951700 | -0.18423000 |
| H | -2.90286100 | -3.16802000 | 1.08706300 |
| C | -6.81356600 | 1.14956500 | 0.24895500 |
| H | -7.24370200 | 1.10806600 | -0.75439600 |
| H | -7.46637300 | 0.57896500 | 0.91024400 |
| H | -6.82570600 | 2.19236800 | 0.56742400 |
| C | -1.95414200 | 1.93153800 | -0.34782300 |
| H | -1.11687900 | 1.89368700 | 0.34686100 |
| H | -1.53305200 | 1.79702300 | -1.34756800 |
| H | -2.38367100 | 2.93235600 | -0.29125000 |

59

e-12-1 carbene fragment

| | | | |
|---|---|---|---|
| C | 2.99972600 | 1.58952100 | 0.17703900 |
| N | 1.28213100 | -0.18392500 | 0.18254900 |
| C | 0.81237400 | -0.67074200 | 1.52581700 |
| C | 4.34728300 | 1.93134000 | 0.06161200 |
| H | 4.63984900 | 2.96828500 | 0.18106800 |
| C | 0.42842500 | -0.22629100 | -0.81104900 |
| C | 1.82226200 | 3.42428300 | -0.99005300 |

| | | | |
|---|---|---|---|
| H | 1.55904600 | 2.72648600 | -1.78783700 |
| H | 2.76147700 | 3.91584600 | -1.25865700 |
| H | 1.04482300 | 4.18955800 | -0.92012400 |
| C | 3.59298800 | -0.72642400 | -0.33615900 |
| C | 5.30664400 | 0.97630500 | -0.22613900 |
| H | 6.34858200 | 1.25964600 | -0.30835400 |
| C | 2.64524000 | 0.24903100 | 0.00824000 |
| C | -0.75712300 | -1.07815800 | -0.30268500 |
| C | 3.03122100 | -2.21319500 | -2.23456400 |
| H | 2.30142200 | -1.48014000 | -2.58490800 |
| H | 2.70594400 | -3.21125800 | -2.53936300 |
| H | 3.99071200 | -2.00376700 | -2.71555500 |
| C | 1.97039100 | 2.68453100 | 0.34019700 |
| H | 1.01078900 | 2.21984100 | 0.55905000 |
| C | 3.18482500 | -2.13576900 | -0.71438700 |
| H | 2.20434400 | -2.34099300 | -0.27667600 |
| C | -0.30318400 | -1.60873100 | 1.08619300 |
| H | -1.09842700 | -1.63357200 | 1.82981100 |
| H | 0.08260800 | -2.62807900 | 0.97687000 |
| C | 2.30400900 | 3.65661100 | 1.46775500 |
| H | 2.46498900 | 3.13697000 | 2.41493600 |
| H | 1.48922400 | 4.37081300 | 1.60116000 |
| H | 3.20678700 | 4.22961700 | 1.24422300 |
| C | 4.92516100 | -0.34040400 | -0.44143600 |
| H | 5.67293600 | -1.07672100 | -0.71036300 |
| C | 4.15447200 | -3.20199100 | -0.21438100 |
| H | 4.34818800 | -3.10262300 | 0.85604500 |
| H | 5.11203900 | -3.14458600 | -0.73621300 |
| H | 3.74521700 | -4.19635200 | -0.40170800 |
| C | 0.25595100 | 0.50723200 | 2.31853600 |
| H | 1.04281600 | 1.22814200 | 2.55148000 |
| H | -0.16391100 | 0.14584500 | 3.25917000 |
| H | -0.53289000 | 1.01490700 | 1.75610900 |
| C | 1.89930300 | -1.35124800 | 2.34088600 |

| | | | |
|---|---|---|---|
| H | 2.32523400 | -2.21242700 | 1.82615800 |
| H | 1.46298100 | -1.70062500 | 3.27871000 |
| H | 2.70417000 | -0.65330900 | 2.58060200 |
| C | -2.14555900 | -0.44208300 | -0.24967700 |
| C | -3.26527000 | -1.20539700 | 0.13981800 |
| C | -2.45389200 | 0.88666500 | -0.57922400 |
| C | -4.56413100 | -0.72143900 | 0.15707200 |
| C | -3.74662500 | 1.39973100 | -0.57491900 |
| C | -4.80090700 | 0.58764500 | -0.21529100 |
| H | -5.37504500 | -1.36722100 | 0.46264400 |
| H | -3.91065200 | 2.43284700 | -0.84665200 |
| C | -0.75308800 | -2.18981300 | -1.37659500 |
| H | -1.04712300 | -1.76831300 | -2.33742000 |
| H | 0.26523600 | -2.57287200 | -1.48454200 |
| H | -1.40541600 | -3.02142500 | -1.12235800 |
| Cl | -3.13007100 | -2.84832400 | 0.67778200 |
| Cl | -1.24573000 | 2.04647500 | -0.98369100 |
| Cl | -6.40501300 | 1.20630100 | -0.20966800 |

60

e-12-1 carbene-Au fragment

| | | | |
|---|---|---|---|
| Au | -0.81044600 | -0.63274900 | -1.91936200 |
| C | -2.72084900 | -1.27339600 | 1.27574600 |
| N | -1.00681200 | 0.45487100 | 0.86499000 |
| C | -0.42032500 | 1.20973000 | 2.02265300 |
| C | -4.06529100 | -1.63972500 | 1.24385800 |
| H | -4.34469200 | -2.63736600 | 1.56270300 |
| C | -0.19175400 | 0.25900400 | -0.13749900 |
| C | -1.62196300 | -3.41481900 | 0.63321600 |
| H | -1.41033300 | -3.00365000 | -0.35791900 |
| H | -2.57127800 | -3.95429900 | 0.58049600 |
| H | -0.83940700 | -4.13270600 | 0.89241900 |
| C | -3.34452900 | 0.90882900 | 0.35285800 |
| C | -5.03675700 | -0.76556700 | 0.78922900 |
| H | -6.07529900 | -1.07074100 | 0.76627500 |

| | | | |
|---|---|---|---|
| C | -2.38330300 | 0.01291300 | 0.84578500 |
| C | 1.02989800 | 1.16752700 | 0.04420700 |
| C | -3.15890700 | 2.24901000 | -1.75120000 |
| H | -2.53004000 | 1.48124500 | -2.20975400 |
| H | -2.89363900 | 3.22253700 | -2.17063700 |
| H | -4.19769900 | 2.03639000 | -2.01751500 |
| C | -1.69378700 | -2.30537700 | 1.68269100 |
| H | -0.71892100 | -1.82169500 | 1.71334700 |
| C | -3.00251500 | 2.26659700 | -0.22995400 |
| H | -1.95469500 | 2.48848200 | -0.00713700 |
| C | 0.66889400 | 1.99881500 | 1.30579300 |
| H | 1.51595900 | 2.17058200 | 1.96736200 |
| H | 0.28276600 | 2.97434700 | 0.99373500 |
| C | -1.98384200 | -2.89285800 | 3.06241200 |
| H | -2.08729800 | -2.11577500 | 3.82310900 |
| H | -1.17866100 | -3.56611600 | 3.36279300 |
| H | -2.91019300 | -3.47173400 | 3.05303000 |
| C | -4.67229700 | 0.49354900 | 0.33853600 |
| H | -5.42950300 | 1.16437000 | -0.05019400 |
| C | -3.86772000 | 3.38013700 | 0.36212100 |
| H | -3.88114600 | 3.35857900 | 1.45334000 |
| H | -4.89968800 | 3.28990000 | 0.01656800 |
| H | -3.50142100 | 4.35539600 | 0.03582800 |
| C | 0.18310700 | 0.21685100 | 3.01024700 |
| H | -0.59060900 | -0.41106200 | 3.45568900 |
| H | 0.67932100 | 0.76752300 | 3.81106700 |
| H | 0.91941900 | -0.42485300 | 2.51937800 |
| C | -1.43020700 | 2.08418400 | 2.74139300 |
| H | -1.82832100 | 2.86460500 | 2.09583400 |
| H | -0.93140800 | 2.56511700 | 3.58457400 |
| H | -2.25849900 | 1.48871900 | 3.13101900 |
| C | 2.41479900 | 0.52436200 | 0.14674500 |
| C | 3.56126100 | 1.33908100 | 0.24932800 |
| C | 2.69496700 | -0.85076300 | 0.13183600 |

| | | | |
|---|---|---|---|
| C | 4.85746100 | 0.84926100 | 0.25852300 |
| C | 3.98518500 | -1.36982200 | 0.13327400 |
| C | 5.06504000 | -0.51463900 | 0.18113200 |
| H | 5.69025100 | 1.53425400 | 0.33049400 |
| H | 4.12736800 | -2.44082800 | 0.10647700 |
| C | 0.95789200 | 2.02153500 | -1.24506300 |
| H | 1.22126900 | 1.40960500 | -2.10815700 |
| H | -0.07094500 | 2.36325900 | -1.39137800 |
| H | 1.60568300 | 2.89151800 | -1.19140800 |
| Cl | 3.47037800 | 3.06136700 | 0.42490000 |
| Cl | 1.46478700 | -2.05540200 | 0.17149400 |
| Cl | 6.66412100 | -1.14120600 | 0.17631200 |

77

e-13-1 carbene fragment

| | | | |
|---|---|---|---|
| C | 3.07929000 | 1.06524000 | 0.33083700 |
| N | 1.03698000 | -0.31263700 | 0.18232400 |
| C | 0.44764400 | -0.78774600 | 1.48063300 |
| C | 4.47312000 | 1.11746100 | 0.30306900 |
| H | 4.97009100 | 2.05973900 | 0.50222900 |
| C | 0.19885200 | -0.07892100 | -0.80047100 |
| C | 2.31987000 | 3.05947600 | -0.88250500 |
| H | 1.88117200 | 2.42354300 | -1.65528000 |
| H | 3.35282100 | 3.28261700 | -1.16377400 |
| H | 1.76860300 | 4.00208500 | -0.84493700 |
| C | 3.20272400 | -1.29101000 | -0.31013000 |
| C | 5.22564500 | -0.00405900 | 0.00007900 |
| H | 6.30695300 | 0.05387100 | -0.01039900 |
| C | 2.46255600 | -0.15935400 | 0.06405200 |
| C | -1.14376800 | -0.70801700 | -0.37632500 |
| C | 2.39546000 | -2.45658000 | -2.33638300 |
| H | 1.84000100 | -1.56076400 | -2.62299800 |
| H | 1.88016300 | -3.33466400 | -2.73405200 |
| H | 3.38951000 | -2.40726600 | -2.78941900 |
| C | 2.29055000 | 2.34768700 | 0.47188800 |

| | | | |
|---|---|---|---|
| H | 1.25443400 | 2.09354000 | 0.69359700 |
| C | 2.52258700 | -2.54766700 | -0.81429900 |
| H | 1.50926200 | -2.58049700 | -0.40536300 |
| C | -0.81948100 | -1.44469800 | 0.95606400 |
| H | -1.61772500 | -1.37189700 | 1.69469700 |
| H | -0.62845000 | -2.50710700 | 0.76047600 |
| C | 2.79187800 | 3.26317500 | 1.58316400 |
| H | 2.84214700 | 2.74261600 | 2.54210600 |
| H | 2.11815900 | 4.11645700 | 1.69047800 |
| H | 3.78539200 | 3.65886700 | 1.36096900 |
| C | 4.59007800 | -1.19346400 | -0.32776000 |
| H | 5.18154300 | -2.05480200 | -0.61346200 |
| C | 3.23637800 | -3.83235100 | -0.40731900 |
| H | 3.42148300 | -3.86877700 | 0.66869500 |
| H | 4.19646500 | -3.93299400 | -0.91801400 |
| H | 2.63279200 | -4.69839800 | -0.68450700 |
| C | 0.11750200 | 0.42936900 | 2.33877300 |
| H | 1.02600500 | 0.97122300 | 2.61461200 |
| H | -0.37909900 | 0.10668100 | 3.25587200 |
| H | -0.55465000 | 1.10507000 | 1.80150400 |
| C | 1.35137600 | -1.72367600 | 2.26508400 |
| H | 1.61366700 | -2.61843300 | 1.70041600 |
| H | 0.82404500 | -2.03758400 | 3.16810300 |
| H | 2.27115900 | -1.21912000 | 2.56829200 |
| C | -2.40777800 | 0.16286500 | -0.27181900 |
| C | -3.63736600 | -0.48320900 | 0.02602000 |
| C | -2.42921300 | 1.55581600 | -0.48764900 |
| C | -4.82181400 | 0.24786600 | 0.03083200 |
| C | -3.64722400 | 2.24066000 | -0.49026800 |
| C | -4.84466400 | 1.60250200 | -0.24643800 |
| H | -5.75124900 | -0.26251600 | 0.25671300 |
| H | -3.64306500 | 3.30563800 | -0.69137600 |
| H | -5.77844700 | 2.15064500 | -0.25582700 |
| C | -1.32398700 | -1.68629800 | -1.56296700 |

| | | | |
|---|---|---|---|
| H | -1.45450100 | -1.10924100 | -2.47861400 |
| H | -0.42388500 | -2.29728100 | -1.68127200 |
| H | -2.17535200 | -2.35238400 | -1.44238900 |
| C | -3.80648400 | -1.95646500 | 0.39066300 |
| H | -2.84596300 | -2.46280600 | 0.36238900 |
| C | -1.20924700 | 2.42642100 | -0.72273700 |
| H | -0.31411000 | 1.83385800 | -0.53730600 |
| C | -4.34389100 | -2.10613600 | 1.81547700 |
| H | -4.40256200 | -3.16158800 | 2.09086200 |
| H | -3.70681200 | -1.59411000 | 2.53992100 |
| H | -5.34646500 | -1.68208200 | 1.90189900 |
| C | -4.72891100 | -2.68079600 | -0.59138100 |
| H | -4.77469100 | -3.74606500 | -0.35407800 |
| H | -5.74544400 | -2.28587400 | -0.53776600 |
| H | -4.38544400 | -2.56871700 | -1.62141700 |
| C | -1.17217000 | 3.62637300 | 0.22509100 |
| H | -1.94515400 | 4.35968600 | -0.01433800 |
| H | -1.31431500 | 3.31984600 | 1.26437400 |
| H | -0.20880900 | 4.13625500 | 0.14784100 |
| C | -1.13355300 | 2.86917700 | -2.18080800 |
| H | -0.28506300 | 3.53891200 | -2.33926500 |
| H | -1.01391000 | 2.00085100 | -2.83181600 |
| H | -2.04531300 | 3.39956600 | -2.46944400 |

78

e-13-1 carbene-Au fragment

| | | | |
|---|---|---|---|
| Au | -0.84933900 | -0.61796700 | -1.89813600 |
| C | -2.75294500 | -0.75036300 | 1.33959300 |
| N | -0.74033000 | 0.58211300 | 0.82475300 |
| C | 0.00526200 | 1.24665200 | 1.94261400 |
| C | -4.14446700 | -0.82458900 | 1.38046900 |
| H | -4.61068700 | -1.73089100 | 1.74950700 |
| C | 0.01096400 | 0.18074600 | -0.16832800 |
| C | -2.17319000 | -3.06917300 | 0.67309900 |
| H | -1.87478800 | -2.72578900 | -0.32194000 |

| | | | |
|---|---|---|---|
| H | -3.23041200 | -3.34374400 | 0.63246100 |
| H | -1.60382700 | -3.96753000 | 0.92328500 |
| C | -2.95221000 | 1.48500200 | 0.35643400 |
| C | -4.93318200 | 0.22075000 | 0.93372200 |
| H | -6.01238400 | 0.14147200 | 0.96941500 |
| C | -2.17548900 | 0.42496900 | 0.84986500 |
| C | 1.40103000 | 0.80994100 | -0.03111000 |
| C | -2.57544300 | 2.66384200 | -1.81562800 |
| H | -2.13159800 | 1.76501200 | -2.25190100 |
| H | -2.13544500 | 3.54352400 | -2.29155200 |
| H | -3.64529800 | 2.65471900 | -2.04098000 |
| C | -1.95087500 | -1.97587300 | 1.71940400 |
| H | -0.89337600 | -1.71223100 | 1.71215000 |
| C | -2.36128900 | 2.71697300 | -0.30215200 |
| H | -1.28243600 | 2.72637300 | -0.12066400 |
| C | 1.20839600 | 1.77479800 | 1.17440400 |
| H | 2.07916000 | 1.82217700 | 1.82675900 |
| H | 0.99386300 | 2.78520400 | 0.80709300 |
| C | -2.29303500 | -2.49504400 | 3.11403600 |
| H | -2.20788000 | -1.71419700 | 3.87286300 |
| H | -1.61924500 | -3.31234200 | 3.38220000 |
| H | -3.31318300 | -2.88408200 | 3.14912000 |
| C | -4.33637700 | 1.35811600 | 0.41246300 |
| H | -4.95487500 | 2.15939600 | 0.02533300 |
| C | -2.95835700 | 4.01113000 | 0.25281800 |
| H | -2.94571600 | 4.04030700 | 1.34380200 |
| H | -3.99565400 | 4.12480900 | -0.06921400 |
| H | -2.40630700 | 4.87308500 | -0.12666600 |
| C | 0.42644400 | 0.18308100 | 2.94956800 |
| H | -0.44403000 | -0.28373200 | 3.41498500 |
| H | 1.02796200 | 0.64651900 | 3.73339500 |
| H | 1.02915000 | -0.58611900 | 2.46091300 |
| C | -0.79348700 | 2.32650000 | 2.64664500 |
| H | -1.04850300 | 3.14720900 | 1.97899400 |

| | | | |
|---|---|---|---|
| H | -0.18569700 | 2.72865300 | 3.45907100 |
| H | -1.71114400 | 1.92072300 | 3.07766200 |
| C | 2.65411500 | -0.07750000 | 0.12692000 |
| C | 3.90887900 | 0.58890200 | 0.18970200 |
| C | 2.64990500 | -1.48777700 | 0.18166900 |
| C | 5.08579600 | -0.15244700 | 0.22398700 |
| C | 3.86355800 | -2.18152500 | 0.20327700 |
| C | 5.07968700 | -1.53465600 | 0.20846600 |
| H | 6.03304900 | 0.37267900 | 0.26408700 |
| H | 3.83842300 | -3.26461200 | 0.21725600 |
| H | 6.00667800 | -2.09384900 | 0.21852700 |
| C | 1.49578800 | 1.56416900 | -1.38133200 |
| H | 1.56650400 | 0.83798500 | -2.19207900 |
| H | 0.58726200 | 2.15263600 | -1.54665200 |
| H | 2.35236400 | 2.23051300 | -1.42747800 |
| C | 4.12775300 | 2.09967000 | 0.26677700 |
| H | 3.17723500 | 2.62376900 | 0.23719900 |
| C | 1.42670700 | -2.38591900 | 0.25021600 |
| H | 0.52774700 | -1.77338600 | 0.30560900 |
| C | 4.79793700 | 2.48383400 | 1.58794200 |
| H | 4.89021800 | 3.56944800 | 1.66461000 |
| H | 4.22806100 | 2.12313100 | 2.44727200 |
| H | 5.80068000 | 2.05783100 | 1.65762600 |
| C | 4.96141000 | 2.61308700 | -0.90863300 |
| H | 5.03934100 | 3.70182100 | -0.87075100 |
| H | 5.97456000 | 2.20779400 | -0.87705400 |
| H | 4.52282900 | 2.32916600 | -1.86691900 |
| C | 1.45568400 | -3.27715800 | 1.49505000 |
| H | 2.25352400 | -4.02003900 | 1.43225500 |
| H | 1.61223400 | -2.70184400 | 2.41021700 |
| H | 0.51264100 | -3.82060400 | 1.58871700 |
| C | 1.29291100 | -3.24332300 | -1.00731200 |
| H | 0.37368700 | -3.83275100 | -0.96881800 |
| H | 1.25647700 | -2.62201700 | -1.90397800 |

| | | | |
|---|---|---|---|
| H | 2.13766500 | -3.93247000 | -1.09413700 |

65

e-14-1 carbene fragment

| | | | |
|---|---|---|---|
| C | 2.84232000 | 1.53293300 | 0.56225200 |
| N | 1.73846400 | -0.59845900 | 0.04365900 |
| C | 1.26055800 | -1.42204500 | 1.21659900 |
| C | 4.03067600 | 2.23950200 | 0.74205300 |
| H | 3.98645900 | 3.27839500 | 1.04695200 |
| C | 1.02307400 | -0.60987000 | -1.04908800 |
| C | 1.34042400 | 3.06012200 | -0.66304600 |
| H | 1.38854800 | 2.39549800 | -1.52892300 |
| H | 2.13532000 | 3.80476000 | -0.76030400 |
| H | 0.38106500 | 3.58188300 | -0.66767200 |
| C | 4.15906100 | -0.40505500 | -0.15081200 |
| C | 5.25972200 | 1.64514600 | 0.50709300 |
| H | 6.17195200 | 2.20937600 | 0.65565100 |
| C | 2.93370500 | 0.19481200 | 0.16507500 |
| C | -0.11615900 | -1.59382100 | -0.78338200 |
| C | 4.41369200 | -1.55827400 | -2.32484300 |
| H | 3.58286700 | -0.97818100 | -2.73129400 |
| H | 4.45905500 | -2.51997000 | -2.84099400 |
| H | 5.34287300 | -1.01782300 | -2.52503600 |
| C | 1.51999500 | 2.26719300 | 0.63388500 |
| H | 0.71575600 | 1.53244900 | 0.68124100 |
| C | 4.23915200 | -1.76282800 | -0.81870500 |
| H | 3.28672500 | -2.27854900 | -0.67658600 |
| C | 0.31540100 | -2.36613100 | 0.48269200 |
| H | -0.51505200 | -2.68426000 | 1.11561000 |
| H | 0.86937700 | -3.25919200 | 0.17449400 |
| C | 1.39089700 | 3.18066300 | 1.84815900 |
| H | 1.57561300 | 2.64318200 | 2.78082800 |
| H | 0.38533300 | 3.60365600 | 1.88641700 |
| H | 2.09347300 | 4.01523300 | 1.79537800 |
| C | 5.31880500 | 0.34094600 | 0.03868900 |

| | | | |
|---|---|---|---|
| H | 6.27915500 | -0.09925900 | -0.20249800 |
| C | 5.35111600 | -2.64270000 | -0.25640100 |
| H | 5.28654300 | -2.72919400 | 0.83041300 |
| H | 6.33604700 | -2.24071400 | -0.50319300 |
| H | 5.29369400 | -3.64359200 | -0.68824500 |
| C | 0.53764100 | -0.52515900 | 2.21666800 |
| H | 1.21382300 | 0.24376100 | 2.59913700 |
| H | 0.19947000 | -1.12940500 | 3.06025400 |
| H | -0.33269200 | -0.04023200 | 1.77142500 |
| C | 2.38955400 | -2.14099200 | 1.93870900 |
| H | 2.91350700 | -2.84256400 | 1.29076200 |
| H | 1.96414100 | -2.70597900 | 2.77020600 |
| H | 3.11123000 | -1.43062900 | 2.34786100 |
| C | -1.41913600 | -0.83695600 | -0.58139300 |
| C | -2.56126700 | -1.52578300 | -0.17877300 |
| C | -1.52247100 | 0.53705800 | -0.78621200 |
| C | -3.75992200 | -0.86035400 | 0.03332100 |
| H | -2.52011100 | -2.59947100 | -0.02449800 |
| C | -2.72252300 | 1.19767000 | -0.55212100 |
| H | -0.65886100 | 1.09378200 | -1.13457200 |
| C | -3.85428200 | 0.51242800 | -0.14006300 |
| H | -4.78506400 | 1.03187700 | 0.03862900 |
| C | -0.24871100 | -2.52634600 | -1.98949600 |
| H | -0.54459300 | -1.96362700 | -2.87477600 |
| H | 0.71310200 | -3.00446200 | -2.19278900 |
| H | -0.98983400 | -3.30707300 | -1.79921100 |
| C | -4.94972100 | -1.67065800 | 0.46865000 |
| C | -2.74653400 | 2.69408700 | -0.69694200 |
| F | -6.03855900 | -0.92113700 | 0.64525900 |
| F | -3.98705100 | 3.18513600 | -0.70220600 |
| F | -2.14674400 | 3.09621600 | -1.82139800 |
| F | -2.09348800 | 3.28485600 | 0.31944000 |
| F | -5.25339400 | -2.61554200 | -0.43079600 |
| F | -4.70949300 | -2.30774900 | 1.62351400 |

66
e-14-1 carbene-Au fragment

| | | | |
|---|---|---|---|
| Au | 1.58150300 | 0.50636700 | -1.97703800 |
| C | 2.09404400 | 1.15396100 | 1.85939600 |
| N | 1.29904700 | -0.83077900 | 0.66043200 |
| C | 0.67900200 | -1.94872100 | 1.44752800 |
| C | 3.15408300 | 1.85504700 | 2.42974600 |
| H | 2.95058400 | 2.78414700 | 2.94915200 |
| C | 0.67928900 | -0.52222000 | -0.45671300 |
| C | 0.69252100 | 2.96221500 | 0.90760100 |
| H | 0.96506100 | 2.64645600 | -0.10354000 |
| H | 1.40877700 | 3.72406500 | 1.22642100 |
| H | -0.29865200 | 3.41897700 | 0.88112400 |
| C | 3.70286600 | -0.48461800 | 1.00123800 |
| C | 4.45752900 | 1.40003700 | 2.32022200 |
| H | 5.26742400 | 1.95749700 | 2.77367000 |
| C | 2.38485200 | -0.04383000 | 1.19035800 |
| C | -0.47370300 | -1.49316800 | -0.65705100 |
| C | 4.82403600 | -1.14435000 | -1.12651600 |
| H | 4.22477600 | -0.42065700 | -1.68331500 |
| H | 5.06646700 | -1.97790500 | -1.78909900 |
| H | 5.76021700 | -0.66365400 | -0.83089100 |
| C | 0.71486500 | 1.77959900 | 1.87835300 |
| H | -0.00708800 | 1.04482900 | 1.52022200 |
| C | 4.07677300 | -1.65206900 | 0.10930800 |
| H | 3.15740600 | -2.13194400 | -0.23872600 |
| C | -0.14813500 | -2.61230400 | 0.35124600 |
| H | -1.02454000 | -3.11655900 | 0.75895400 |
| H | 0.46898100 | -3.35977400 | -0.15686200 |
| C | 0.27334900 | 2.23080900 | 3.26823500 |
| H | 0.32877400 | 1.42171400 | 3.99898500 |
| H | -0.75674000 | 2.59088100 | 3.22828500 |
| H | 0.89270500 | 3.05404700 | 3.63084300 |
| C | 4.72591700 | 0.25273500 | 1.59205700 |

| | | | |
|---|---|---|---|
| H | 5.75164000 | -0.07097900 | 1.45807400 |
| C | 4.94543400 | -2.68129800 | 0.83364400 |
| H | 4.50952900 | -3.00841600 | 1.77807200 |
| H | 5.92956700 | -2.25990500 | 1.05104000 |
| H | 5.10034200 | -3.55685900 | 0.20004400 |
| C | -0.18114200 | -1.37018500 | 2.56705400 |
| H | 0.43090800 | -0.76189300 | 3.23685600 |
| H | -0.61128900 | -2.18871500 | 3.14633600 |
| H | -0.99683800 | -0.75731100 | 2.18067600 |
| C | 1.70955000 | -2.88424400 | 2.05232200 |
| H | 2.29963700 | -3.38541300 | 1.28665500 |
| H | 1.18837500 | -3.64711000 | 2.63329000 |
| H | 2.38010600 | -2.34559600 | 2.72558800 |
| C | -1.79881900 | -0.79160000 | -0.37209400 |
| C | -2.94326900 | -1.55198500 | -0.15066700 |
| C | -1.92429500 | 0.59769500 | -0.38546900 |
| C | -4.16925200 | -0.94569600 | 0.08593100 |
| H | -2.89165000 | -2.63542900 | -0.16939600 |
| C | -3.15351800 | 1.19387000 | -0.13483200 |
| H | -1.06027100 | 1.21709700 | -0.60343100 |
| C | -4.28806600 | 0.43390500 | 0.10899200 |
| H | -5.24177000 | 0.90584800 | 0.29996900 |
| C | -0.51456100 | -2.02643600 | -2.09158400 |
| H | -0.72663800 | -1.21927100 | -2.79376100 |
| H | 0.44847200 | -2.46562900 | -2.35941500 |
| H | -1.29278000 | -2.78920800 | -2.17902800 |
| C | -5.35991200 | -1.83312300 | 0.32613600 |
| C | -3.23895600 | 2.69483200 | -0.06579000 |
| F | -6.48325800 | -1.13837900 | 0.50460100 |
| F | -4.47025200 | 3.13859400 | -0.32901200 |
| F | -2.40233600 | 3.28512600 | -0.92023500 |
| F | -2.92016200 | 3.13741100 | 1.16310100 |
| F | -5.56004800 | -2.66904800 | -0.70048200 |
| F | -5.18355000 | -2.59859500 | 1.41250200 |

66
e-15-1 carbene fragment

| | | | |
|---|---|---|---|
| C | 2.51790700 | 1.29874100 | -0.42435700 |
| N | 0.73702200 | -0.16667400 | 0.38960900 |
| C | 0.41388200 | -0.11492600 | 1.85926200 |
| C | 3.84871500 | 1.45450600 | -0.80793800 |
| H | 4.20499000 | 2.43830300 | -1.09122200 |
| C | -0.24598100 | -0.26639200 | -0.46717200 |
| C | 1.25051100 | 2.69354300 | -2.03110300 |
| H | 0.82840500 | 1.78823800 | -2.47177100 |
| H | 2.16094000 | 2.95747900 | -2.57687600 |
| H | 0.52969800 | 3.50680900 | -2.14622900 |
| C | 2.91711700 | -1.10597500 | -0.19166000 |
| C | 4.71016300 | 0.37004900 | -0.86334800 |
| H | 5.74160200 | 0.51197400 | -1.16131100 |
| C | 2.08901100 | 0.01482800 | -0.06683800 |
| C | -1.53188200 | -0.27033200 | 0.37583500 |
| C | 2.13285900 | -3.07035700 | -1.47387900 |
| H | 1.44384500 | -2.42771500 | -2.02658400 |
| H | 1.71128400 | -4.07719400 | -1.42186700 |
| H | 3.07463900 | -3.12173700 | -2.02701500 |
| C | 1.56166200 | 2.46749800 | -0.55034700 |
| H | 0.61812600 | 2.20388100 | -0.07224000 |
| C | 2.37272300 | -2.51362400 | -0.06917700 |
| H | 1.39894200 | -2.46368500 | 0.42060400 |
| C | -1.04119100 | -0.58047200 | 1.80588800 |
| H | -1.64876900 | -0.15155400 | 2.60282400 |
| H | -1.06304200 | -1.66659300 | 1.93203900 |
| C | 2.08554000 | 3.74708600 | 0.09369700 |
| H | 2.34927600 | 3.59486500 | 1.14225500 |
| H | 1.32420600 | 4.52790800 | 0.04115400 |
| H | 2.97151100 | 4.11942300 | -0.42596600 |
| C | 4.23796100 | -0.90339700 | -0.58258600 |
| H | 4.89707500 | -1.75695500 | -0.69121200 |

| | | | |
|---|---|---|---|
| C | 3.27178600 | -3.43841100 | 0.74373100 |
| H | 3.48359800 | -3.02439700 | 1.73186100 |
| H | 4.22483300 | -3.60925700 | 0.23832600 |
| H | 2.79382300 | -4.41151100 | 0.87228300 |
| C | 0.60251100 | 1.30138300 | 2.39449100 |
| H | 1.64534000 | 1.60863100 | 2.27882700 |
| H | 0.36273100 | 1.31449400 | 3.45961700 |
| H | -0.03475700 | 2.02584000 | 1.89082200 |
| C | 1.29476400 | -1.04675400 | 2.68148900 |
| H | 1.14225700 | -2.09401200 | 2.42189800 |
| H | 1.04434000 | -0.92359300 | 3.73684200 |
| H | 2.35174800 | -0.79906600 | 2.55532000 |
| C | -2.11215700 | 1.12498500 | 0.16284400 |
| C | -2.54173400 | 1.95422600 | 1.19513100 |
| C | -2.23720200 | 1.58664200 | -1.15245600 |
| C | -3.05979000 | 3.21863400 | 0.92854400 |
| H | -2.47539800 | 1.62738300 | 2.22519300 |
| C | -2.74691900 | 2.84836900 | -1.42016000 |
| H | -1.92099900 | 0.93745900 | -1.95975500 |
| C | -3.15612800 | 3.67424200 | -0.37813300 |
| H | -3.38779600 | 3.84529500 | 1.74873500 |
| H | -2.82769500 | 3.18708000 | -2.44576800 |
| H | -3.55427000 | 4.65974200 | -0.58477600 |
| C | -2.48882400 | -1.34757900 | -0.10804500 |
| C | -1.96734600 | -2.57237800 | -0.52498100 |
| C | -3.86930300 | -1.17724200 | -0.09705000 |
| C | -2.80510900 | -3.60525900 | -0.91940700 |
| H | -0.88830800 | -2.70493100 | -0.55269300 |
| C | -4.71285500 | -2.21204100 | -0.48748400 |
| H | -4.28807000 | -0.22597700 | 0.21158600 |
| C | -4.18449800 | -3.42761900 | -0.89997900 |
| H | -2.38327700 | -4.54703000 | -1.24834900 |
| H | -5.78567900 | -2.06396600 | -0.47299200 |
| H | -4.84145500 | -4.23075600 | -1.20968100 |

67
e-15-1 carbene-Au fragment

| | | | |
|---|---|---|---|
| Au | -0.07245500 | -1.04135100 | -1.60523500 |
| C | 2.41024200 | 1.58405000 | -0.20507100 |
| N | 0.76926500 | 0.20917400 | 0.96586700 |
| C | 0.45908900 | 0.54775100 | 2.39321000 |
| C | 3.71701400 | 1.76400500 | -0.65116700 |
| H | 3.98131400 | 2.69304700 | -1.14305700 |
| C | -0.25709100 | -0.05116400 | 0.19613200 |
| C | 1.08509800 | 2.64063400 | -2.02486000 |
| H | 0.73564100 | 1.65711200 | -2.35001000 |
| H | 1.98730900 | 2.88937900 | -2.59037900 |
| H | 0.31568300 | 3.37993300 | -2.26041900 |
| C | 3.01849100 | -0.68160800 | 0.51331900 |
| C | 4.67033200 | 0.76970900 | -0.50011900 |
| H | 5.68251900 | 0.93067300 | -0.84946200 |
| C | 2.09753500 | 0.37052900 | 0.42614300 |
| C | -1.53800400 | 0.12573800 | 1.01139000 |
| C | 2.74649200 | -3.04453400 | -0.22848600 |
| H | 2.11722800 | -2.71531300 | -1.05863000 |
| H | 2.43080800 | -4.04611900 | 0.07173300 |
| H | 3.77965200 | -3.10730200 | -0.58014900 |
| C | 1.37652000 | 2.64730200 | -0.52310200 |
| H | 0.44070400 | 2.39915100 | -0.02103800 |
| C | 2.64714300 | -2.08253700 | 0.95845400 |
| H | 1.60307700 | -2.07502400 | 1.28420800 |
| C | -1.00643700 | 0.11203800 | 2.45983700 |
| H | -1.58905300 | 0.71288400 | 3.15664700 |
| H | -1.04582900 | -0.92027500 | 2.81482100 |
| C | 1.80010600 | 4.04394700 | -0.07412000 |
| H | 2.06640600 | 4.07165500 | 0.98395200 |
| H | 0.98223100 | 4.74842800 | -0.24157000 |
| H | 2.66043100 | 4.39768100 | -0.64709500 |
| C | 4.31335100 | -0.44788300 | 0.05526500 |

| | | | |
|---|---|---|---|
| H | 5.04350300 | -1.24701100 | 0.11314400 |
| C | 3.53111500 | -2.59389100 | 2.09549800 |
| H | 3.54219600 | -1.92358500 | 2.95513700 |
| H | 4.56121800 | -2.71060600 | 1.75054000 |
| H | 3.18628400 | -3.57539700 | 2.42704200 |
| C | 0.69415500 | 2.03210900 | 2.65291800 |
| H | 1.74726100 | 2.27486600 | 2.49202100 |
| H | 0.45181600 | 2.24813500 | 3.69531700 |
| H | 0.08639400 | 2.67320900 | 2.01802200 |
| C | 1.32776000 | -0.24082300 | 3.35934800 |
| H | 1.18900500 | -1.31576900 | 3.24446100 |
| H | 1.05167600 | 0.03209200 | 4.37962400 |
| H | 2.38260700 | 0.00318100 | 3.21670300 |
| C | -2.09540000 | 1.46859500 | 0.53247600 |
| C | -2.41155000 | 2.52144400 | 1.38514100 |
| C | -2.29751300 | 1.64389700 | -0.84104300 |
| C | -2.88234100 | 3.73146900 | 0.88169600 |
| H | -2.28928200 | 2.42075800 | 2.45564900 |
| C | -2.76327000 | 2.84839900 | -1.34494700 |
| H | -2.08148700 | 0.81982700 | -1.51387800 |
| C | -3.04901500 | 3.90364700 | -0.48428300 |
| H | -3.11820300 | 4.53778700 | 1.56495000 |
| H | -2.90279800 | 2.96252400 | -2.41264600 |
| H | -3.40917600 | 4.84669900 | -0.87560400 |
| C | -2.52233300 | -1.01311300 | 0.78686200 |
| C | -2.04486000 | -2.32491100 | 0.80822400 |
| C | -3.88462200 | -0.79997400 | 0.61512100 |
| C | -2.90639400 | -3.39859600 | 0.65211700 |
| H | -0.97920900 | -2.50193100 | 0.93313600 |
| C | -4.75410900 | -1.87655800 | 0.46526700 |
| H | -4.27304400 | 0.21112600 | 0.59190400 |
| C | -4.26887400 | -3.17562000 | 0.47997800 |
| H | -2.51550100 | -4.40811900 | 0.65514600 |
| H | -5.81300500 | -1.69382400 | 0.33137200 |

| | | | |
|---|---|---|---|
| H | -4.94509500 | -4.01166300 | 0.35371500 |

84

e-16-1 carbene fragment

| | | | |
|---|---|---|---|
| C | 3.08624900 | 0.52242400 | -0.46073900 |
| N | 1.09776100 | -0.62822900 | 0.40711000 |
| C | 0.88544000 | -0.57682100 | 1.89044000 |
| C | 4.42629000 | 0.46699300 | -0.84109000 |
| H | 4.93206500 | 1.38780400 | -1.10846500 |
| C | 0.06819100 | -0.43361800 | -0.38119600 |
| C | 2.19974400 | 2.19869400 | -2.04703800 |
| H | 1.56161900 | 1.45951100 | -2.53714200 |
| H | 3.17002000 | 2.21526500 | -2.55119000 |
| H | 1.73852100 | 3.18315600 | -2.15663100 |
| C | 3.10077500 | -1.91006100 | -0.25764300 |
| C | 5.11154300 | -0.73429200 | -0.90698900 |
| H | 6.15437500 | -0.75249700 | -1.19836600 |
| C | 2.44729300 | -0.67811500 | -0.11223800 |
| C | -1.14456800 | -0.08205700 | 0.54066900 |
| C | 2.08666600 | -3.71736400 | -1.59837400 |
| H | 1.57264200 | -2.95026700 | -2.18122500 |
| H | 1.46993500 | -4.61944900 | -1.58372300 |
| H | 3.02817200 | -3.95202800 | -2.10275900 |
| C | 2.37202000 | 1.85577100 | -0.56542100 |
| H | 1.37235400 | 1.76577000 | -0.14040900 |
| C | 2.35977600 | -3.22537500 | -0.17519800 |
| H | 1.39689300 | -3.03759600 | 0.29216300 |
| C | -0.64963500 | -0.53144300 | 1.93795200 |
| H | -1.01694600 | 0.04899000 | 2.77505500 |
| H | -1.01782300 | -1.54783600 | 2.09413300 |
| C | 3.09915800 | 2.98646800 | 0.15880200 |
| H | 3.31396000 | 2.73252800 | 1.19879200 |
| H | 2.47729000 | 3.88586000 | 0.14682500 |
| H | 4.04500300 | 3.23196900 | -0.33044200 |
| C | 4.43860300 | -1.91582700 | -0.64428700 |

| | | | |
|---|---|---|---|
| H | 4.94907900 | -2.86460500 | -0.76306900 |
| C | 3.08807900 | -4.29730000 | 0.62890200 |
| H | 3.33567900 | -3.95367000 | 1.63479500 |
| H | 4.01605600 | -4.59871200 | 0.13799500 |
| H | 2.46373500 | -5.18900700 | 0.71503200 |
| C | 1.62481200 | 0.62945800 | 2.46189200 |
| H | 2.70034500 | 0.49523000 | 2.32042900 |
| H | 1.43701700 | 0.70252800 | 3.53503900 |
| H | 1.33225600 | 1.56236100 | 1.98339600 |
| C | 1.42516700 | -1.81076500 | 2.60457400 |
| H | 0.92109600 | -2.72608500 | 2.29224200 |
| H | 1.26485000 | -1.68891000 | 3.67792700 |
| H | 2.49910700 | -1.91831600 | 2.43518800 |
| C | -1.15082400 | 1.43605300 | 0.25684500 |
| C | -0.96534100 | 2.45912000 | 1.21543400 |
| C | -1.27238600 | 1.82543700 | -1.10256600 |
| C | -0.67352000 | 3.75433600 | 0.78013000 |
| C | -0.99582300 | 3.13735700 | -1.47431900 |
| C | -0.62189500 | 4.10843700 | -0.55818500 |
| H | -0.50522600 | 4.52096300 | 1.53132100 |
| H | -1.05990300 | 3.39789800 | -2.52691600 |
| C | -2.46156000 | -0.78694500 | 0.16319400 |
| C | -2.43599300 | -2.13400200 | -0.26030100 |
| C | -3.72015400 | -0.15883200 | 0.24667200 |
| C | -3.60835100 | -2.78256000 | -0.63285900 |
| C | -4.86982900 | -0.85096400 | -0.14216100 |
| C | -4.84499700 | -2.15533100 | -0.60206600 |
| H | -3.54593900 | -3.81799000 | -0.95535000 |
| H | -5.82448600 | -0.33792500 | -0.06994900 |
| C | -1.17640900 | -2.95195800 | -0.33306100 |
| H | -0.50065500 | -2.57960200 | -1.10437800 |
| H | -0.62930400 | -2.93850200 | 0.61115700 |
| H | -1.41868800 | -3.99089200 | -0.55939900 |
| C | -6.09345400 | -2.86026600 | -1.04818500 |

| | | | |
|---|---|---|---|
| H | -6.98354600 | -2.37870500 | -0.64247300 |
| H | -6.17848100 | -2.85006500 | -2.13732900 |
| H | -6.09244100 | -3.90426700 | -0.73155500 |
| C | -3.96639900 | 1.24547800 | 0.73918900 |
| H | -3.42815200 | 1.46430500 | 1.65698900 |
| H | -3.67665400 | 2.00291400 | 0.00722400 |
| H | -5.03030100 | 1.36820400 | 0.94345400 |
| C | -0.20051600 | 5.47990600 | -0.99669900 |
| H | -0.34589000 | 6.21395000 | -0.20351000 |
| H | 0.86162300 | 5.48739500 | -1.26190300 |
| H | -0.75661100 | 5.80440500 | -1.87703500 |
| C | -1.80276700 | 0.93353700 | -2.19790900 |
| H | -1.52614200 | 1.34747200 | -3.16884800 |
| H | -1.42914300 | -0.08350600 | -2.13652100 |
| H | -2.89642600 | 0.90073300 | -2.14845700 |
| C | -1.18022800 | 2.35827100 | 2.71373400 |
| H | -2.04139100 | 1.74580800 | 2.97652800 |
| H | -0.32751100 | 1.96958300 | 3.26703400 |
| H | -1.37729400 | 3.36036100 | 3.09512600 |

85

e-16-1 carbene-Au fragment

| | | | |
|---|---|---|---|
| Au | 0.39475300 | -0.85035700 | -1.77713100 |
| C | 2.96443200 | 0.82277900 | 0.05927600 |
| N | 0.98644500 | -0.27718200 | 1.01642300 |
| C | 0.70031700 | -0.02860800 | 2.46317900 |
| C | 4.31606800 | 0.76628100 | -0.27342000 |
| H | 4.79797300 | 1.66150800 | -0.64897800 |
| C | -0.03388800 | -0.24204000 | 0.19737600 |
| C | 2.18870500 | 2.36906100 | -1.73231700 |
| H | 1.66316900 | 1.56345700 | -2.25240800 |
| H | 3.20298200 | 2.43457400 | -2.13478800 |
| H | 1.67703300 | 3.31142900 | -1.94255000 |
| C | 3.05971800 | -1.56369200 | 0.60235600 |
| C | 5.04374800 | -0.40566900 | -0.15849600 |

| | | | |
|---|---|---|---|
| H | 6.09373400 | -0.42350200 | -0.42226600 |
| C | 2.36102400 | -0.34816500 | 0.54980900 |
| C | -1.30540100 | 0.18833000 | 0.99214800 |
| C | 2.32516600 | -3.71937000 | -0.40355800 |
| H | 1.82722000 | -3.16405700 | -1.20308200 |
| H | 1.77828200 | -4.64880500 | -0.22619600 |
| H | 3.33185000 | -3.97700500 | -0.74389400 |
| C | 2.22643400 | 2.11916100 | -0.22244300 |
| H | 1.19267900 | 2.03142200 | 0.11695400 |
| C | 2.39645900 | -2.89514800 | 0.88431900 |
| H | 1.37412200 | -2.70064900 | 1.20107900 |
| C | -0.83603600 | -0.04076800 | 2.45043800 |
| H | -1.25842900 | 0.64488700 | 3.17269100 |
| H | -1.17549500 | -1.03460600 | 2.74632300 |
| C | 2.86649800 | 3.32238000 | 0.47133700 |
| H | 3.01439300 | 3.15966800 | 1.53991800 |
| H | 2.22853000 | 4.20106800 | 0.34242300 |
| H | 3.83951200 | 3.55261400 | 0.03033300 |
| C | 4.40764500 | -1.56380800 | 0.25270400 |
| H | 4.95552000 | -2.49854600 | 0.28139100 |
| C | 3.10719300 | -3.70706700 | 1.96601200 |
| H | 3.23428800 | -3.15067600 | 2.89523600 |
| H | 4.09718700 | -4.01863400 | 1.62534300 |
| H | 2.54008000 | -4.61456300 | 2.18347500 |
| C | 1.37927400 | 1.26867400 | 2.88734000 |
| H | 2.46162100 | 1.15358500 | 2.79316300 |
| H | 1.15570500 | 1.46977000 | 3.93657800 |
| H | 1.07089900 | 2.11910400 | 2.28325700 |
| C | 1.24967900 | -1.12906400 | 3.36113000 |
| H | 0.82166200 | -2.10647700 | 3.13878400 |
| H | 1.00232300 | -0.87911000 | 4.39484400 |
| H | 2.33642300 | -1.18503600 | 3.27811900 |
| C | -1.36308200 | 1.66833800 | 0.52943000 |
| C | -1.19240700 | 2.80036600 | 1.36501100 |

| | | | |
|---|---|---|---|
| C | -1.49628900 | 1.90608100 | -0.86125200 |
| C | -0.88052900 | 4.03012500 | 0.78215000 |
| C | -1.17776600 | 3.15578600 | -1.38686700 |
| C | -0.79095100 | 4.21969100 | -0.58876100 |
| H | -0.72694100 | 4.88176700 | 1.43883100 |
| H | -1.24117200 | 3.28928100 | -2.46277000 |
| C | -2.57232600 | -0.63362100 | 0.69416200 |
| C | -2.47982500 | -2.02550000 | 0.47940000 |
| C | -3.85455000 | -0.04808700 | 0.65147700 |
| C | -3.61162200 | -2.75482200 | 0.12439000 |
| C | -4.95630900 | -0.82315200 | 0.29196400 |
| C | -4.86107600 | -2.17177300 | -0.01010000 |
| H | -3.50319200 | -3.82218200 | -0.04400400 |
| H | -5.93065300 | -0.34438400 | 0.25964600 |
| C | -1.21444400 | -2.82734500 | 0.63197400 |
| H | -0.57382100 | -2.75822300 | -0.25281800 |
| H | -0.62394600 | -2.50898400 | 1.48981500 |
| H | -1.46547100 | -3.87813900 | 0.78006200 |
| C | -6.05670700 | -2.96658000 | -0.44823100 |
| H | -6.98021100 | -2.53847600 | -0.05719200 |
| H | -6.13432300 | -2.97886800 | -1.53782500 |
| H | -5.98678000 | -4.00216800 | -0.11369400 |
| C | -4.17060700 | 1.38511900 | 1.00058300 |
| H | -3.65841200 | 1.71051400 | 1.90211100 |
| H | -3.90555900 | 2.08692000 | 0.20748200 |
| H | -5.24195200 | 1.47553000 | 1.18106600 |
| C | -0.34104900 | 5.52514600 | -1.17413800 |
| H | -0.59270500 | 6.36182400 | -0.52143200 |
| H | 0.74504800 | 5.53060600 | -1.30951100 |
| H | -0.79170600 | 5.69770700 | -2.15176900 |
| C | -2.12894500 | 0.94948800 | -1.84114900 |
| H | -1.69304200 | 1.08470000 | -2.83168100 |
| H | -2.04924000 | -0.10173200 | -1.58500900 |
| H | -3.19760300 | 1.18763000 | -1.90651300 |

| | | | |
|---|---|---|---|
| C | -1.46195700 | 2.89583700 | 2.85623600 |
| H | -2.33160000 | 2.31860500 | 3.16616200 |
| H | -0.63076300 | 2.59683600 | 3.49126300 |
| H | -1.67940400 | 3.93808300 | 3.08960700 |

90

e-17-1 carbene fragment

| | | | |
|---|---|---|---|
| C | 3.44711600 | -0.55378600 | -0.50163500 |
| N | 1.23481400 | -1.05773800 | 0.44508100 |
| C | 1.11630700 | -0.90312100 | 1.93358800 |
| C | 4.68968700 | -1.02152000 | -0.92833200 |
| H | 5.45005400 | -0.30305300 | -1.21441200 |
| C | 0.22497000 | -0.70069800 | -0.31209000 |
| C | 3.12727000 | 1.33108100 | -2.07988800 |
| H | 2.34633700 | 0.78080800 | -2.60841100 |
| H | 4.08711700 | 1.11566800 | -2.55769600 |
| H | 2.92441400 | 2.40031900 | -2.17996100 |
| C | 2.70356600 | -2.87106500 | -0.30418200 |
| C | 4.95769800 | -2.37720000 | -1.02207500 |
| H | 5.93174200 | -2.71827300 | -1.35054800 |
| C | 2.48230000 | -1.49953500 | -0.12451700 |
| C | -0.78667500 | -0.00330500 | 0.64579800 |
| C | 1.04322400 | -4.15738400 | -1.59440200 |
| H | 0.65304900 | -3.24269600 | -2.04689500 |
| H | 0.24451600 | -4.90312700 | -1.56407200 |
| H | 1.84570200 | -4.54735200 | -2.22682100 |
| C | 3.16138200 | 0.93233200 | -0.60246700 |
| H | 2.16830200 | 1.13030400 | -0.19693600 |
| C | 1.57064900 | -3.86510500 | -0.18770700 |
| H | 0.76333300 | -3.38210300 | 0.35990500 |
| C | -0.35334100 | -0.49110100 | 2.04668900 |
| H | -0.53317900 | 0.21784300 | 2.84618200 |
| H | -0.93944600 | -1.38835300 | 2.25133500 |
| C | 4.17753400 | 1.79846700 | 0.13833900 |
| H | 4.28959000 | 1.50325100 | 1.18273300 |

| | | | |
|---|---|---|---|
| H | 3.85483400 | 2.84379400 | 0.10785400 |
| H | 5.16140000 | 1.74325500 | -0.33480800 |
| C | 3.95493100 | -3.29323900 | -0.74390300 |
| H | 4.13712900 | -4.35197400 | -0.88860800 |
| C | 1.95449100 | -5.15193700 | 0.53319600 |
| H | 2.38313400 | -4.94970000 | 1.51702800 |
| H | 2.68077400 | -5.73187900 | -0.04098500 |
| H | 1.07301100 | -5.78488400 | 0.66699100 |
| C | 2.12177000 | 0.12253000 | 2.44496200 |
| H | 3.13825300 | -0.26759000 | 2.34396200 |
| H | 1.93116900 | 0.31794400 | 3.50273800 |
| H | 2.05965700 | 1.06440500 | 1.90403900 |
| C | 1.37855700 | -2.21926000 | 2.65742400 |
| H | 0.63697500 | -2.97468400 | 2.39203000 |
| H | 1.31623100 | -2.04643800 | 3.73409300 |
| H | 2.37801800 | -2.60148100 | 2.43325800 |
| C | -0.43124200 | 1.43612400 | 0.24524100 |
| C | 0.09083800 | 2.43719100 | 1.07540400 |
| C | -0.51905700 | 1.76448000 | -1.12333900 |
| C | 0.70325900 | 3.58424600 | 0.55827000 |
| C | 0.07031300 | 2.90214400 | -1.65753500 |
| C | 0.72954600 | 3.78525300 | -0.81781500 |
| H | 1.13321900 | 4.31409700 | 1.22576300 |
| H | 0.02085700 | 3.11764800 | -2.71583200 |
| C | -2.23682900 | -0.36412700 | 0.38589200 |
| C | -2.60674900 | -1.69715500 | 0.13015400 |
| C | -3.26697000 | 0.56710000 | 0.39357500 |
| C | -3.90369600 | -2.04735700 | -0.21254100 |
| C | -4.58323500 | 0.24805900 | 0.04615300 |
| C | -4.88891900 | -1.06261300 | -0.27495400 |
| H | -4.18977200 | -3.06314000 | -0.44201900 |
| H | -5.33252200 | 1.02672100 | 0.06930200 |
| O | -6.12742200 | -1.48795400 | -0.63214000 |
| O | -2.98022800 | 1.83702300 | 0.79423600 |

| | | | |
|---|---|---|---|
| O | -1.62299200 | -2.62937200 | 0.25445800 |
| O | 1.34523900 | 4.84030300 | -1.40979700 |
| O | -1.29414400 | 0.97222800 | -1.90719500 |
| O | -0.02577800 | 2.27599000 | 2.41861800 |
| C | 2.03572100 | 5.74864700 | -0.58919600 |
| H | 2.82160900 | 5.24631300 | -0.01502200 |
| H | 2.48853200 | 6.48464000 | -1.24843600 |
| H | 1.35807800 | 6.25670100 | 0.10420000 |
| C | 0.65822200 | 3.14982200 | 3.27827200 |
| H | 0.50751100 | 2.77232300 | 4.28741800 |
| H | 1.73174100 | 3.16804300 | 3.05734400 |
| H | 0.26237200 | 4.16787400 | 3.21638800 |
| C | -3.39339100 | 2.89257300 | -0.04877100 |
| H | -3.33719300 | 2.59149400 | -1.09801900 |
| H | -4.41147400 | 3.21800800 | 0.18481700 |
| H | -2.70517900 | 3.72231500 | 0.12012800 |
| C | -7.14951300 | -0.52648800 | -0.70735400 |
| H | -6.90556200 | 0.25585700 | -1.43282300 |
| H | -8.04768700 | -1.04767000 | -1.02887100 |
| H | -7.33150500 | -0.06196600 | 0.26687500 |
| C | -1.98592000 | -3.98498800 | 0.15385700 |
| H | -2.79822600 | -4.22643800 | 0.84547500 |
| H | -2.29323500 | -4.24126200 | -0.86432100 |
| H | -1.10606800 | -4.56861200 | 0.41883700 |
| C | -0.77369400 | 0.53512200 | -3.14469500 |
| H | 0.12318500 | -0.06267000 | -2.96536100 |
| H | -1.53931200 | -0.09012800 | -3.59883600 |
| H | -0.55330000 | 1.36916100 | -3.81651400 |

91

e-17-1 carbene-Au fragment

| | | | |
|---|---|---|---|
| Au | 0.35992100 | -1.41885200 | -1.59055400 |
| C | 3.32049800 | 0.62845500 | -0.29214300 |
| N | 1.42629000 | -0.33304200 | 0.92901000 |
| C | 1.26220900 | 0.07409400 | 2.36099200 |

| | | | |
|---|---|---|---|
| C | 4.64440300 | 0.52446000 | -0.71301100 |
| H | 5.10048500 | 1.36748700 | -1.21925500 |
| C | 0.33481600 | -0.36859700 | 0.20390600 |
| C | 2.32526000 | 1.89320800 | -2.18262800 |
| H | 1.74258200 | 1.02046000 | -2.49266800 |
| H | 3.28178200 | 1.87230100 | -2.71214000 |
| H | 1.78856200 | 2.79915100 | -2.47525700 |
| C | 3.44024300 | -1.69064400 | 0.48177800 |
| C | 5.37570300 | -0.63525300 | -0.51859500 |
| H | 6.40672500 | -0.69081200 | -0.84501200 |
| C | 2.74860100 | -0.47635200 | 0.36114800 |
| C | -0.83644300 | 0.19323700 | 1.05565200 |
| C | 2.70034000 | -3.94561100 | -0.25466900 |
| H | 2.09998100 | -3.52394300 | -1.06542100 |
| H | 2.25499400 | -4.89426000 | 0.05717800 |
| H | 3.70058900 | -4.16125400 | -0.63890700 |
| C | 2.55097600 | 1.88102600 | -0.66887300 |
| H | 1.56750100 | 1.85672800 | -0.19800300 |
| C | 2.78608800 | -2.98081400 | 0.93233300 |
| H | 1.76714800 | -2.74476400 | 1.24920400 |
| C | -0.26225600 | 0.02695400 | 2.48040200 |
| H | -0.65659500 | 0.72685100 | 3.20576400 |
| H | -0.53235800 | -0.98263300 | 2.79344700 |
| C | 3.25320600 | 3.16762900 | -0.23822700 |
| H | 3.47980400 | 3.17436300 | 0.82989700 |
| H | 2.60931700 | 4.02331300 | -0.46166500 |
| H | 4.18978500 | 3.31024700 | -0.78261300 |
| C | 4.76394400 | -1.73875900 | 0.05039100 |
| H | 5.31147000 | -2.66991900 | 0.14209400 |
| C | 3.53460200 | -3.67069400 | 2.07277600 |
| H | 3.70632500 | -3.01566000 | 2.92634700 |
| H | 4.50628900 | -4.03145500 | 1.72650400 |
| H | 2.97029400 | -4.54108200 | 2.41589700 |
| C | 1.92979100 | 1.42542000 | 2.59804300 |

| | | | |
|---|---|---|---|
| H | 3.01365000 | 1.31858900 | 2.50259500 |
| H | 1.70482500 | 1.76089500 | 3.61242900 |
| H | 1.60094500 | 2.18868700 | 1.89663400 |
| C | 1.90440600 | -0.92440600 | 3.31160400 |
| H | 1.47663800 | -1.92061200 | 3.19667700 |
| H | 1.72297000 | -0.58956500 | 4.33524500 |
| H | 2.98419400 | -0.96955700 | 3.15701200 |
| C | -0.89779500 | 1.63100200 | 0.49560000 |
| C | -0.66510400 | 2.81340300 | 1.20864600 |
| C | -1.16163200 | 1.80589000 | -0.88303300 |
| C | -0.41358600 | 4.03381300 | 0.56804600 |
| C | -0.93045600 | 3.00634500 | -1.54219800 |
| C | -0.49214300 | 4.10449800 | -0.81601600 |
| H | -0.17445800 | 4.90976500 | 1.14911100 |
| H | -1.06819000 | 3.11008400 | -2.60819400 |
| C | -2.13591400 | -0.57987000 | 0.90852600 |
| C | -2.12416300 | -1.98368000 | 0.81969000 |
| C | -3.38036900 | 0.03384100 | 0.81700900 |
| C | -3.26436200 | -2.70710300 | 0.50559500 |
| C | -4.54824200 | -0.66805000 | 0.51024400 |
| C | -4.47441500 | -2.04010000 | 0.33066700 |
| H | -3.25575500 | -3.78078400 | 0.39450300 |
| H | -5.48344500 | -0.13062800 | 0.44601000 |
| O | -5.53955100 | -2.81864400 | 0.01969900 |
| O | -3.44749800 | 1.37014400 | 1.05899100 |
| O | -0.93891800 | -2.59364200 | 1.07905200 |
| O | -0.19781000 | 5.21832000 | -1.52859700 |
| O | -1.70226300 | 0.75291400 | -1.52005800 |
| O | -0.70464000 | 2.76281500 | 2.56368100 |
| C | 0.24568400 | 6.35461300 | -0.82815400 |
| H | 1.15251800 | 6.13702700 | -0.25445700 |
| H | 0.46574100 | 7.11474100 | -1.57292000 |
| H | -0.52551800 | 6.72960600 | -0.14819900 |
| C | -0.30717000 | 3.88373600 | 3.31095000 |

| | | | |
|---|---|---|---|
| H | -0.34211300 | 3.58513100 | 4.35648200 |
| H | 0.71381600 | 4.19062400 | 3.05838400 |
| H | -0.98505100 | 4.72844000 | 3.15914800 |
| C | -4.23490700 | 2.16325300 | 0.19892700 |
| H | -4.19210400 | 1.78172200 | -0.82550200 |
| H | -5.27739400 | 2.20295700 | 0.52777300 |
| H | -3.81405700 | 3.16937700 | 0.22332900 |
| C | -6.78055000 | -2.18679500 | -0.16987800 |
| H | -6.73114900 | -1.45026700 | -0.97784800 |
| H | -7.49090300 | -2.96518600 | -0.43625500 |
| H | -7.11780900 | -1.68905100 | 0.74470500 |
| C | -0.79950400 | -3.94512500 | 0.69423000 |
| H | -1.47706200 | -4.58863300 | 1.26171300 |
| H | -0.98181200 | -4.05567600 | -0.37887600 |
| H | 0.22558600 | -4.23009300 | 0.91329800 |
| C | -1.77976700 | 0.76278600 | -2.92857300 |
| H | -0.79473900 | 0.94249700 | -3.37007500 |
| H | -2.12070700 | -0.22725400 | -3.21995200 |
| H | -2.48759500 | 1.52251400 | -3.27653100 |

66

e-18-1 carbene fragment

| | | | |
|---|---|---|---|
| C | 3.09178100 | 0.76860500 | -0.50596600 |
| N | 1.18028600 | -0.45131700 | 0.41769600 |
| C | 0.96040400 | -0.37069700 | 1.90285900 |
| C | 4.41404300 | 0.75363200 | -0.94657200 |
| H | 4.87734100 | 1.68462700 | -1.25308600 |
| C | 0.15147800 | -0.37985200 | -0.38473400 |
| C | 2.01308200 | 2.35775100 | -2.07217800 |
| H | 1.42612700 | 1.55012600 | -2.51535200 |
| H | 2.94601700 | 2.45994900 | -2.63312000 |
| H | 1.45366400 | 3.29184300 | -2.16633400 |
| C | 3.19627000 | -1.66486700 | -0.26798600 |
| C | 5.13104100 | -0.42929000 | -1.02689700 |
| H | 6.15873500 | -0.41876500 | -1.36815100 |

| | | | |
|---|---|---|---|
| C | 2.51992000 | -0.44913700 | -0.11555400 |
| C | -1.07551200 | -0.13729300 | 0.54262000 |
| C | 2.09109000 | -3.49064300 | -1.51051200 |
| H | 1.46027500 | -2.75563100 | -2.01455200 |
| H | 1.54045800 | -4.43121100 | -1.43785900 |
| H | 2.98431900 | -3.65857600 | -2.11878000 |
| C | 2.30219100 | 2.05915900 | -0.59969500 |
| H | 1.33598400 | 1.91774800 | -0.11535000 |
| C | 2.48290900 | -2.99079700 | -0.11877500 |
| H | 1.55483000 | -2.82113000 | 0.42370900 |
| C | -0.56089300 | -0.53714300 | 1.94395000 |
| H | -1.02592000 | -0.01884100 | 2.77576200 |
| H | -0.77485100 | -1.60266600 | 2.05725600 |
| C | 3.00047900 | 3.24132800 | 0.06583100 |
| H | 3.25880200 | 3.02618500 | 1.10455800 |
| H | 2.34573100 | 4.11606600 | 0.04794500 |
| H | 3.91902600 | 3.50910300 | -0.46153900 |
| C | 4.51418600 | -1.63136300 | -0.71565100 |
| H | 5.05394400 | -2.56219400 | -0.84472600 |
| C | 3.29796900 | -4.03723200 | 0.63260700 |
| H | 3.62108000 | -3.67080400 | 1.60951700 |
| H | 4.18801800 | -4.32848000 | 0.07042700 |
| H | 2.70146000 | -4.93898900 | 0.78362900 |
| C | 1.49953200 | 0.95257800 | 2.43944600 |
| H | 2.58797600 | 0.96896100 | 2.33877700 |
| H | 1.25066800 | 1.04090100 | 3.49782600 |
| H | 1.09925500 | 1.82099000 | 1.92241900 |
| C | 1.66598200 | -1.48872200 | 2.66159300 |
| H | 1.26487600 | -2.47148000 | 2.41462700 |
| H | 1.52581500 | -1.32478100 | 3.73182200 |
| H | 2.73949600 | -1.47934500 | 2.45799100 |
| C | -1.30140300 | 1.34968800 | 0.26945800 |
| C | -1.26680700 | 2.39088000 | 1.19368400 |
| C | -1.49575100 | 1.77263600 | -1.05384600 |

| | | | |
|---|---|---|---|
| C | -1.27981000 | 3.73450300 | 0.85762700 |
| C | -1.52544500 | 3.09537000 | -1.45340500 |
| C | -1.38753100 | 4.06209100 | -0.47785700 |
| H | -1.23310500 | 4.49082000 | 1.62754200 |
| H | -1.66205100 | 3.35056400 | -2.49417700 |
| C | -2.26613000 | -0.98803600 | 0.14265900 |
| C | -2.08661100 | -2.32115500 | -0.22255600 |
| C | -3.59331100 | -0.56943300 | 0.14206600 |
| C | -3.10134400 | -3.17398500 | -0.60969100 |
| C | -4.65959600 | -1.36732800 | -0.23591800 |
| C | -4.38550900 | -2.66463100 | -0.61635000 |
| H | -2.89247700 | -4.19610300 | -0.88952800 |
| H | -5.66806100 | -0.98041700 | -0.22353300 |
| F | -5.39300200 | -3.45738400 | -0.98685900 |
| F | -0.84957200 | -2.83209200 | -0.16675100 |
| F | -3.89060700 | 0.67284900 | 0.54024800 |
| F | -1.23626600 | 2.12858800 | 2.50793000 |
| F | -1.72131400 | 0.86317700 | -1.99766900 |
| F | -1.39586700 | 5.34831100 | -0.83169400 |

67

e-18-1 carbene-Au fragment

| | | | |
|---|---|---|---|
| Au | 0.25295100 | -1.14264300 | -1.62354300 |
| C | 2.86831100 | 1.33090500 | -0.20124300 |
| N | 1.13259900 | 0.03408600 | 0.92711800 |
| C | 0.85997900 | 0.32039600 | 2.36957000 |
| C | 4.18782200 | 1.44309600 | -0.63158000 |
| H | 4.51381900 | 2.36940400 | -1.09048100 |
| C | 0.08880100 | -0.09488200 | 0.14119300 |
| C | 1.63957600 | 2.53504200 | -1.99580600 |
| H | 1.18489600 | 1.60342400 | -2.34401300 |
| H | 2.56868600 | 2.69194200 | -2.54992700 |
| H | 0.96348000 | 3.36317800 | -2.22248700 |
| C | 3.32705700 | -0.98869800 | 0.44814800 |
| C | 5.07764100 | 0.38904800 | -0.50322400 |

| | | | |
|---|---|---|---|
| H | 6.10179100 | 0.49853600 | -0.83725500 |
| C | 2.47110900 | 0.12085600 | 0.38997500 |
| C | -1.18389400 | 0.16810900 | 0.98727900 |
| C | 2.94037900 | -3.30692400 | -0.38130400 |
| H | 2.34799700 | -2.91449700 | -1.21066000 |
| H | 2.55748100 | -4.29598000 | -0.12080400 |
| H | 3.97540100 | -3.42171700 | -0.71454500 |
| C | 1.91644400 | 2.47552600 | -0.49232300 |
| H | 0.96219800 | 2.27982500 | -0.00261100 |
| C | 2.87157600 | -2.38155600 | 0.83720700 |
| H | 1.82548600 | -2.32799100 | 1.14390300 |
| C | -0.64462200 | 0.03645600 | 2.43237000 |
| H | -1.15823200 | 0.65090900 | 3.16135900 |
| H | -0.77528800 | -1.00211800 | 2.73303400 |
| C | 2.43455400 | 3.82211800 | 0.00811300 |
| H | 2.70227700 | 3.78784800 | 1.06584900 |
| H | 1.66651500 | 4.58752100 | -0.12915700 |
| H | 3.31679500 | 4.13971100 | -0.55219300 |
| C | 4.63897300 | -0.81970200 | 0.00985900 |
| H | 5.31696300 | -1.66455600 | 0.04926800 |
| C | 3.71043700 | -2.98876300 | 1.96202400 |
| H | 3.75247700 | -2.35906000 | 2.85042700 |
| H | 4.73583000 | -3.15443000 | 1.62269100 |
| H | 3.30266300 | -3.96033200 | 2.24823200 |
| C | 1.28090900 | 1.74799300 | 2.70579500 |
| H | 2.36546500 | 1.83917200 | 2.60798000 |
| H | 1.00673500 | 1.96847600 | 3.73815800 |
| H | 0.82013400 | 2.49324200 | 2.06240800 |
| C | 1.62486600 | -0.61439000 | 3.29262200 |
| H | 1.37057600 | -1.65873400 | 3.11171300 |
| H | 1.36664300 | -0.36966000 | 4.32489500 |
| H | 2.70062600 | -0.47607600 | 3.17161300 |
| C | -1.55795300 | 1.59330500 | 0.54798800 |
| C | -1.59085800 | 2.74137800 | 1.33851300 |

| | | | |
|---|---|---|---|
| C | -1.82228700 | 1.84601600 | -0.80673400 |
| C | -1.71048900 | 4.02975300 | 0.84037200 |
| C | -1.95831600 | 3.09983100 | -1.36605700 |
| C | -1.86680300 | 4.18721900 | -0.51943700 |
| H | -1.70370500 | 4.87389500 | 1.51444800 |
| H | -2.14458600 | 3.21028400 | -2.42430500 |
| C | -2.26186100 | -0.87149000 | 0.72415000 |
| C | -1.93285400 | -2.22960200 | 0.74900700 |
| C | -3.60834400 | -0.61779000 | 0.47743700 |
| C | -2.81710500 | -3.25828600 | 0.49989100 |
| C | -4.54861800 | -1.60039400 | 0.22095800 |
| C | -4.12643100 | -2.91307500 | 0.22670900 |
| H | -2.48900700 | -4.28696000 | 0.51859200 |
| H | -5.57748300 | -1.33565500 | 0.02582400 |
| F | -5.01120200 | -3.87859900 | -0.02115700 |
| F | -0.67747200 | -2.57517100 | 1.06824200 |
| F | -4.06112700 | 0.64060300 | 0.49236300 |
| F | -1.52288900 | 2.66357900 | 2.67499800 |
| F | -2.01275600 | 0.81093700 | -1.61843300 |
| F | -1.97410400 | 5.41550300 | -1.02386900 |

66

e-19-1 carbene fragment

| | | | |
|---|---|---|---|
| C | 3.46857400 | -0.20885000 | -0.47461500 |
| N | 1.31550400 | -0.96736400 | 0.43591300 |
| C | 1.12349600 | -0.85708000 | 1.92133400 |
| C | 4.77370600 | -0.51370600 | -0.85832300 |
| H | 5.43240800 | 0.29277600 | -1.15931400 |
| C | 0.33292500 | -0.62452300 | -0.36019500 |
| C | 2.87530000 | 1.54622000 | -2.11551900 |
| H | 2.10553200 | 0.92264800 | -2.57700700 |
| H | 3.82434100 | 1.36251200 | -2.62588500 |
| H | 2.61410100 | 2.59792100 | -2.25890900 |
| C | 3.05279600 | -2.59884000 | -0.19169700 |
| C | 5.23331600 | -1.81920700 | -0.88686800 |

| | | | |
|---|---|---|---|
| H | 6.25327700 | -2.03165600 | -1.18197100 |
| C | 2.63224000 | -1.26595900 | -0.08493400 |
| C | -0.79056600 | -0.05153400 | 0.57856700 |
| C | 1.68464500 | -4.20190900 | -1.47131700 |
| H | 1.26760100 | -3.36571600 | -2.03632100 |
| H | 0.93914100 | -5.00005100 | -1.42502400 |
| H | 2.55796100 | -4.57998100 | -2.00985600 |
| C | 3.00057900 | 1.22546800 | -0.62375500 |
| H | 2.00537300 | 1.31997700 | -0.18977900 |
| C | 2.08687700 | -3.75512900 | -0.06393900 |
| H | 1.18884500 | -3.38998500 | 0.42914700 |
| C | -0.38528000 | -0.58865000 | 1.96609500 |
| H | -0.69553100 | 0.00970100 | 2.81491100 |
| H | -0.88286100 | -1.55862700 | 2.05644600 |
| C | 3.91852700 | 2.23381400 | 0.06298300 |
| H | 4.07359200 | 1.99077100 | 1.11610500 |
| H | 3.48067700 | 3.23390800 | -0.00167500 |
| H | 4.89706900 | 2.27557800 | -0.42081800 |
| C | 4.36438700 | -2.85410000 | -0.58260200 |
| H | 4.69785300 | -3.88127200 | -0.67241000 |
| C | 2.63281500 | -4.93076100 | 0.73921000 |
| H | 2.98010400 | -4.62173300 | 1.72694100 |
| H | 3.46700500 | -5.41155800 | 0.22364800 |
| H | 1.85561500 | -5.68633200 | 0.86977500 |
| C | 2.04044900 | 0.23260400 | 2.46863900 |
| H | 3.07914900 | -0.09777900 | 2.38510800 |
| H | 1.81848000 | 0.40728900 | 3.52194400 |
| H | 1.94356300 | 1.17560600 | 1.93584500 |
| C | 1.46194800 | -2.14403000 | 2.66439800 |
| H | 0.80306600 | -2.96668900 | 2.38499600 |
| H | 1.34467200 | -1.96441000 | 3.73511200 |
| H | 2.49940100 | -2.43463300 | 2.48493800 |
| C | -0.55006600 | 1.43259500 | 0.24268800 |
| C | -0.11958700 | 2.45321600 | 1.10968700 |

| | | | |
|---|---|---|---|
| C | -0.61604000 | 1.82004900 | -1.11512000 |
| C | 0.41682600 | 3.65203100 | 0.65801500 |
| C | -0.10934700 | 3.02182100 | -1.58569000 |
| C | 0.46670600 | 3.90761400 | -0.69770400 |
| H | 0.76866000 | 4.38239600 | 1.37309000 |
| H | -0.14999700 | 3.24116600 | -2.64332100 |
| C | -2.21375900 | -0.49954400 | 0.24697300 |
| C | -2.49452600 | -1.77346800 | -0.26596400 |
| C | -3.34985300 | 0.28999900 | 0.47238400 |
| C | -3.76091800 | -2.18876100 | -0.64593000 |
| C | -4.63214400 | -0.09077000 | 0.10036700 |
| C | -4.82794900 | -1.32581600 | -0.48379800 |
| H | -3.90317400 | -3.17930700 | -1.05423300 |
| H | -5.46334000 | 0.57548200 | 0.28273200 |
| Cl | -1.26171600 | -2.96657600 | -0.41856000 |
| Cl | -6.40524400 | -1.80936400 | -0.96641500 |
| Cl | -3.26315700 | 1.80372300 | 1.29487600 |
| Cl | -1.39813700 | 0.86434800 | -2.31559300 |
| Cl | 1.21161400 | 5.34452300 | -1.27567200 |
| Cl | -0.31321700 | 2.41139200 | 2.82984600 |

67

e-19-1 carbene-Au fragment

| | | | |
|---|---|---|---|
| Au | 0.50271400 | -1.12203800 | -1.77948500 |
| C | 3.36441200 | 0.20001100 | 0.04225800 |
| N | 1.23110100 | -0.55269400 | 1.00367300 |
| C | 0.95639100 | -0.25007800 | 2.44454600 |
| C | 4.68955100 | -0.08042000 | -0.28382400 |
| H | 5.30835900 | 0.71549700 | -0.68149800 |
| C | 0.24994600 | -0.36866400 | 0.15711100 |
| C | 2.84841700 | 1.80885900 | -1.79100800 |
| H | 2.19575600 | 1.09053700 | -2.29446100 |
| H | 3.85861700 | 1.69204200 | -2.19098600 |
| H | 2.51096900 | 2.82177400 | -2.02594600 |
| C | 3.07922100 | -2.15533600 | 0.65303100 |

| | | | |
|---|---|---|---|
| C | 5.22027100 | -1.35007200 | -0.13564500 |
| H | 6.25331200 | -1.54240200 | -0.39648300 |
| C | 2.58300600 | -0.84634000 | 0.56031500 |
| C | -0.95280200 | 0.26065800 | 0.93985700 |
| C | 1.97279500 | -4.18582600 | -0.27513200 |
| H | 1.52755300 | -3.58229600 | -1.07069600 |
| H | 1.30495900 | -5.02188000 | -0.05184800 |
| H | 2.91727300 | -4.59504300 | -0.64394900 |
| C | 2.84449900 | 1.59016500 | -0.27577800 |
| H | 1.80952200 | 1.66974500 | 0.06137300 |
| C | 2.21401400 | -3.35230900 | 0.98533900 |
| H | 1.24795100 | -2.98175700 | 1.32193400 |
| C | -0.56512100 | -0.07120800 | 2.39678900 |
| H | -0.94565600 | 0.61785200 | 3.13996300 |
| H | -1.01012700 | -1.04655600 | 2.60527000 |
| C | 3.66106200 | 2.69170500 | 0.40034400 |
| H | 3.74348400 | 2.54630300 | 1.47858600 |
| H | 3.19611400 | 3.66393100 | 0.21409600 |
| H | 4.67276300 | 2.73115600 | -0.00995100 |
| C | 4.40897700 | -2.38007100 | 0.30739900 |
| H | 4.80161300 | -3.38847300 | 0.36608200 |
| C | 2.81032000 | -4.24030900 | 2.07638300 |
| H | 3.06870600 | -3.68335000 | 2.97780400 |
| H | 3.71589700 | -4.73553400 | 1.71903200 |
| H | 2.10003800 | -5.02383900 | 2.34806200 |
| C | 1.79394100 | 0.95088300 | 2.86948800 |
| H | 2.84959300 | 0.66862700 | 2.85859800 |
| H | 1.52455200 | 1.24309200 | 3.88456800 |
| H | 1.66793000 | 1.80988700 | 2.21611400 |
| C | 1.32386500 | -1.39884200 | 3.37355700 |
| H | 0.74222400 | -2.29825900 | 3.17211800 |
| H | 1.11890000 | -1.08221800 | 4.39817900 |
| H | 2.38798500 | -1.62757300 | 3.29887900 |
| C | -0.78685200 | 1.72409700 | 0.46124900 |

| | | | |
|---|---|---|---|
| C | -0.39858400 | 2.83352100 | 1.23507500 |
| C | -0.87545200 | 2.00407700 | -0.92142000 |
| C | 0.14287900 | 3.98655100 | 0.68039500 |
| C | -0.34648900 | 3.14746200 | -1.50061100 |
| C | 0.23094900 | 4.10685200 | -0.69241100 |
| H | 0.46627300 | 4.78865800 | 1.32900400 |
| H | -0.39415700 | 3.27344200 | -2.57309400 |
| C | -2.32702800 | -0.33762400 | 0.64098900 |
| C | -2.50671800 | -1.70201300 | 0.36811400 |
| C | -3.51699900 | 0.40322800 | 0.68257100 |
| C | -3.72397500 | -2.25382300 | -0.00070200 |
| C | -4.75126900 | -0.11884000 | 0.32558400 |
| C | -4.84242300 | -1.44467300 | -0.05078800 |
| H | -3.78906000 | -3.30859500 | -0.22676400 |
| H | -5.62823400 | 0.51214500 | 0.35256400 |
| Cl | -1.22707400 | -2.84099400 | 0.56036000 |
| Cl | -6.35759000 | -2.10102800 | -0.52260500 |
| Cl | -3.56188800 | 2.03752600 | 1.23317300 |
| Cl | -1.76366300 | 1.00305200 | -1.99885800 |
| Cl | 0.99051600 | 5.48038600 | -1.38768000 |
| Cl | -0.68971400 | 2.98301300 | 2.93552700 |

78

e-20-1 carbene fragment

| | | | |
|---|---|---|---|
| C | -1.22526000 | 3.87172800 | 0.45475400 |
| N | -1.28222800 | 1.66847600 | -0.60933900 |
| C | -0.92851700 | 1.57283700 | -2.07081600 |
| C | -1.92340000 | 4.98747800 | 0.91360300 |
| H | -1.37220500 | 5.81687800 | 1.34111600 |
| C | -0.87187900 | 0.72904000 | 0.19594700 |
| C | 0.52248300 | 3.47355100 | 2.16941900 |
| H | 0.01872200 | 2.55130700 | 2.46648900 |
| H | 0.14665000 | 4.28905100 | 2.79328000 |
| H | 1.59371200 | 3.36557400 | 2.35896400 |
| C | -3.37115300 | 2.81172700 | -0.07896700 |

| | | | |
|---|---|---|---|
| C | -3.30743000 | 5.03762500 | 0.86135300 |
| H | -3.83078700 | 5.91453600 | 1.22130700 |
| C | -1.97197900 | 2.81986900 | -0.08694800 |
| C | -0.08979800 | -0.26268600 | -0.68724000 |
| C | -4.56403500 | 0.86704400 | 0.88850300 |
| H | -3.68250300 | 0.64193200 | 1.49391900 |
| H | -5.09358000 | -0.06589900 | 0.67888500 |
| H | -5.22370800 | 1.51223500 | 1.47470000 |
| C | 0.26868500 | 3.77348100 | 0.69037200 |
| H | 0.65677500 | 2.92439900 | 0.12672600 |
| C | -4.16244800 | 1.56409400 | -0.41343900 |
| H | -3.50835100 | 0.87566300 | -0.95209700 |
| C | -0.51497400 | 0.10171600 | -2.12530000 |
| H | 0.23255000 | -0.09367000 | -2.89402000 |
| H | -1.39609100 | -0.49714800 | -2.37294300 |
| C | 1.02801500 | 5.02232600 | 0.25344800 |
| H | 0.82869500 | 5.27412900 | -0.79016500 |
| H | 2.10194900 | 4.86643100 | 0.37069400 |
| H | 0.75653500 | 5.88526500 | 0.86534300 |
| C | -4.02459800 | 3.94685000 | 0.39205400 |
| H | -5.10782300 | 3.96614000 | 0.41426700 |
| C | -5.37969200 | 1.83933600 | -1.28900600 |
| H | -5.10478000 | 2.37468600 | -2.20049800 |
| H | -6.12246000 | 2.43767300 | -0.75740700 |
| H | -5.86029000 | 0.90046300 | -1.56971300 |
| C | 0.19795600 | 2.54718100 | -2.40267300 |
| H | -0.12330500 | 3.57226800 | -2.20113200 |
| H | 0.43651600 | 2.46984500 | -3.46481200 |
| H | 1.10531000 | 2.34883200 | -1.83389500 |
| C | -2.10690600 | 1.88666400 | -2.98254000 |
| H | -2.91541000 | 1.16374900 | -2.88079900 |
| H | -1.76330100 | 1.86113300 | -4.01801700 |
| H | -2.49800400 | 2.88697500 | -2.78256000 |
| C | 1.37077000 | -0.00546500 | -0.34346200 |

| | | | |
|---|---|---|---|
| C | 2.37677900 | 0.09628400 | -1.29137300 |
| C | 1.71448400 | 0.14358400 | 1.00826500 |
| C | 3.68989300 | 0.36954900 | -0.90992300 |
| H | 2.16310700 | -0.02000600 | -2.34689800 |
| C | 3.01665100 | 0.42872500 | 1.37322000 |
| H | 0.94097100 | 0.05387800 | 1.76011500 |
| C | 4.02237500 | 0.54861400 | 0.41737900 |
| H | 5.03917400 | 0.77534600 | 0.71074400 |
| C | -0.50905700 | -1.67816600 | -0.33513000 |
| C | -1.85490700 | -1.91495400 | -0.06217400 |
| C | 0.37137600 | -2.75056600 | -0.30571800 |
| C | -2.30641800 | -3.19175700 | 0.22602300 |
| H | -2.55791600 | -1.08595600 | -0.06460700 |
| C | -0.09093300 | -4.03122500 | -0.01760200 |
| H | 1.42728200 | -2.59836600 | -0.50205300 |
| C | -1.42863900 | -4.26742700 | 0.25226000 |
| H | -1.78016700 | -5.26390600 | 0.47956800 |
| C | -3.76783200 | -3.36925500 | 0.53489200 |
| C | 0.90537000 | -5.15866800 | -0.02364700 |
| C | 4.72261900 | 0.48586100 | -1.99667900 |
| C | 3.39000100 | 0.62692100 | 2.81789200 |
| F | 4.80846100 | -0.64306400 | -2.71112100 |
| F | 4.40531700 | 1.46110100 | -2.86118200 |
| F | 5.93678100 | 0.75400700 | -1.51811900 |
| F | 3.97274900 | 1.82057300 | 3.00320500 |
| F | 4.27008900 | -0.29541100 | 3.22526400 |
| F | 2.33594700 | 0.56532700 | 3.62946000 |
| F | -4.11651000 | -4.65364000 | 0.61820100 |
| F | -4.53606600 | -2.80326600 | -0.40813200 |
| F | -4.09843700 | -2.78704900 | 1.69404500 |
| F | 0.37343500 | -6.30731500 | 0.39666200 |
| F | 1.95422100 | -4.88725600 | 0.76160600 |
| F | 1.39209600 | -5.37003500 | -1.25428900 |

e-20-1 carbene-Au fragment

| | | | |
|---|---|---|---|
| Au | 0.99459700 | 1.07379300 | -1.60201200 |
| C | -1.98734200 | 3.18840100 | -0.18588200 |
| N | -0.31546700 | 1.83555900 | 0.96346200 |
| C | -0.52540800 | 1.53264500 | 2.41590700 |
| C | -2.39425500 | 4.43051500 | -0.66528400 |
| H | -3.37661400 | 4.52527800 | -1.11337500 |
| C | 0.04388300 | 0.83058100 | 0.20332500 |
| C | -2.91334900 | 1.63236000 | -1.89350100 |
| H | -1.91135600 | 1.38066100 | -2.25246100 |
| H | -3.28672400 | 2.47371500 | -2.48275200 |
| H | -3.57284500 | 0.77815700 | -2.06849900 |
| C | 0.18697700 | 4.17549000 | 0.38684600 |
| C | -1.55785900 | 5.53378700 | -0.59990700 |
| H | -1.89515300 | 6.49255900 | -0.97298900 |
| C | -0.71042900 | 3.09950300 | 0.38993200 |
| C | 0.09876800 | -0.43588800 | 1.06368600 |
| C | 2.52230000 | 4.25440500 | -0.48180800 |
| H | 2.24847700 | 3.56146300 | -1.28043500 |
| H | 3.57386100 | 4.09679000 | -0.23341600 |
| H | 2.41181300 | 5.27539800 | -0.85611100 |
| C | -2.89087300 | 1.98931700 | -0.40562100 |
| H | -2.47486400 | 1.12838400 | 0.11947800 |
| C | 1.65105200 | 4.04637100 | 0.76031400 |
| H | 1.82856600 | 3.02634400 | 1.11353300 |
| C | 0.09172700 | 0.13118500 | 2.49799800 |
| H | -0.40937700 | -0.50317600 | 3.22810100 |
| H | 1.12970400 | 0.22762100 | 2.82334600 |
| C | -4.31122900 | 2.21071700 | 0.11029000 |
| H | -4.32250900 | 2.53255500 | 1.15306000 |
| H | -4.88200600 | 1.28199200 | 0.03211100 |
| H | -4.83266800 | 2.96637600 | -0.48139700 |
| C | -0.27304300 | 5.39692600 | -0.10108300 |
| H | 0.40121100 | 6.24552100 | -0.11248000 |

| | | | |
|---|---|---|---|
| C | 2.07718300 | 5.03971500 | 1.84068900 |
| H | 1.46857400 | 4.97193600 | 2.74234700 |
| H | 2.00150500 | 6.06244000 | 1.46416200 |
| H | 3.12021400 | 4.87040800 | 2.11457700 |
| C | -2.00866500 | 1.60124600 | 2.76789400 |
| H | -2.37949000 | 2.61615800 | 2.60734900 |
| H | -2.13112800 | 1.35924300 | 3.82521000 |
| H | -2.62276700 | 0.91687800 | 2.18627200 |
| C | 0.20969200 | 2.52004200 | 3.30745100 |
| H | 1.28393500 | 2.51416800 | 3.12211300 |
| H | 0.04001500 | 2.24497200 | 4.35000700 |
| H | -0.17569200 | 3.53013500 | 3.15665200 |
| C | -1.17075100 | -1.17486100 | 0.64670300 |
| C | -2.18953900 | -1.51039500 | 1.52172400 |
| C | -1.35223300 | -1.43856600 | -0.71820800 |
| C | -3.38929400 | -2.03794400 | 1.04414500 |
| H | -2.09509400 | -1.34104100 | 2.58665500 |
| C | -2.54206000 | -1.96707100 | -1.18032000 |
| H | -0.55939600 | -1.19227800 | -1.41746100 |
| C | -3.58440300 | -2.26014400 | -0.30374300 |
| H | -4.52436000 | -2.64868000 | -0.67233300 |
| C | 1.35865900 | -1.24129100 | 0.79834200 |
| C | 2.58290000 | -0.56362400 | 0.77674200 |
| C | 1.35390400 | -2.61193600 | 0.61423200 |
| C | 3.76093700 | -1.24930200 | 0.56545500 |
| H | 2.60575300 | 0.51277400 | 0.91503500 |
| C | 2.55115100 | -3.29781500 | 0.40343400 |
| H | 0.42287400 | -3.16721600 | 0.62540600 |
| C | 3.75727400 | -2.62949500 | 0.37218600 |
| H | 4.68320800 | -3.16265400 | 0.19996100 |
| C | 5.08185500 | -0.52987100 | 0.53182400 |
| C | 2.48517100 | -4.79021600 | 0.22565000 |
| C | -4.48091100 | -2.29356000 | 2.04558600 |
| C | -2.73305400 | -2.23157800 | -2.65117000 |

| | | | |
|---|---|---|---|
| F | -4.07104900 | -3.11265100 | 3.02089700 |
| F | -4.85722600 | -1.14969200 | 2.64049000 |
| F | -5.56542500 | -2.83180100 | 1.49169300 |
| F | -3.97840800 | -1.92502600 | -3.03727900 |
| F | -2.54323800 | -3.52362200 | -2.94333600 |
| F | -1.89092200 | -1.52321000 | -3.40062700 |
| F | 5.90217900 | -0.98409000 | 1.49005800 |
| F | 4.94710000 | 0.78525200 | 0.71431100 |
| F | 5.71044100 | -0.71618900 | -0.63198200 |
| F | 3.67105100 | -5.31650600 | -0.08052000 |
| F | 1.62812500 | -5.12787700 | -0.74425600 |
| F | 2.05570700 | -5.39162500 | 1.34388800 |

66

f-1-1 carbene fragment

| | | | |
|---|---|---|---|
| C | -1.19515400 | -1.31153800 | 1.20560000 |
| N | 0.02207500 | 0.20966800 | -0.30187200 |
| C | 0.03189500 | -0.17514600 | -1.76296600 |
| C | -1.17047300 | -2.35985500 | 2.12681300 |
| H | -2.11271500 | -2.74047100 | 2.50295600 |
| C | -0.02155400 | 1.48496300 | 0.00443700 |
| C | 1.23418300 | -1.29867700 | 1.20938100 |
| C | 0.02665600 | -2.88534800 | 2.58168700 |
| C | 0.01731700 | -0.80073700 | 0.72086700 |
| C | 0.25659600 | 1.19796700 | -2.40243500 |
| H | -0.36810000 | 1.33055300 | -3.28856200 |
| H | 1.30118000 | 1.28570900 | -2.71461500 |
| C | 1.21955000 | -2.34429500 | 2.13277300 |
| H | 2.16449200 | -2.71282300 | 2.51404700 |
| C | -1.29986400 | -0.80413500 | -2.15346000 |
| H | -1.45059200 | -1.75433700 | -1.63517100 |
| H | -1.29615400 | -1.00547900 | -3.22599500 |
| H | -2.14081800 | -0.14547900 | -1.92700300 |
| C | 1.15776200 | -1.14542600 | -2.08504300 |
| H | 2.13213000 | -0.70263800 | -1.87459800 |

| | | | |
|---|---|---|---|
| H | 1.11767500 | -1.38846900 | -3.14845100 |
| H | 1.05956700 | -2.07861200 | -1.52350000 |
| C | -0.04523100 | 2.23835400 | -1.30675400 |
| C | -1.44465400 | 2.84345200 | -1.44946900 |
| H | -1.67797500 | 3.47511600 | -0.59062100 |
| H | -1.49074400 | 3.45371800 | -2.35501300 |
| H | -2.21314000 | 2.07009100 | -1.52291300 |
| C | 0.99040200 | 3.35688800 | -1.27829100 |
| H | 0.96794100 | 3.91609800 | -2.21751200 |
| H | 0.78961300 | 4.04285900 | -0.45413100 |
| H | 1.99757300 | 2.95287900 | -1.14933500 |
| C | 2.55575700 | -0.76194800 | 0.79853500 |
| C | 3.54172200 | -1.63902800 | 0.36249700 |
| C | 2.85661100 | 0.60131100 | 0.88546300 |
| C | 4.79582900 | -1.16782700 | -0.01020600 |
| H | 3.32298600 | -2.69888400 | 0.28472600 |
| C | 4.11356100 | 1.05500700 | 0.52298800 |
| H | 2.10532300 | 1.29490400 | 1.24566300 |
| C | 5.09293500 | 0.18006300 | 0.06208600 |
| H | 6.07006500 | 0.54870300 | -0.22196200 |
| C | -2.52456500 | -0.78440400 | 0.80555900 |
| C | -3.49241500 | -1.66133300 | 0.33012700 |
| C | -2.84997000 | 0.56762400 | 0.94596100 |
| C | -4.75039200 | -1.19760400 | -0.03682600 |
| H | -3.25606700 | -2.71438800 | 0.21778100 |
| C | -4.11190300 | 1.01376700 | 0.58991200 |
| H | -2.10979500 | 1.26045800 | 1.32952800 |
| C | -5.07098400 | 0.14194700 | 0.08436900 |
| H | -6.05139900 | 0.50493600 | -0.19559400 |
| C | -5.74177900 | -2.19274300 | -0.57387400 |
| C | -4.48874700 | 2.46456800 | 0.73191000 |
| F | -6.91026300 | -1.62970900 | -0.88096500 |
| F | -5.27870700 | -2.78972700 | -1.68044900 |
| F | -5.98290500 | -3.16656700 | 0.31320600 |

| | | | |
|---|---|---|---|
| F | -4.68497000 | 3.03520300 | -0.46568700 |
| F | -3.55653700 | 3.17349100 | 1.36588100 |
| F | -5.63408000 | 2.60217700 | 1.41160000 |
| C | 5.80724600 | -2.16470200 | -0.50538900 |
| C | 4.46834600 | 2.51529500 | 0.61793600 |
| F | 5.37768700 | -2.78009900 | -1.61540000 |
| F | 6.02964600 | -3.12396700 | 0.40196000 |
| F | 6.98020400 | -1.59887700 | -0.78932800 |
| F | 5.59385100 | 2.69489300 | 1.32004500 |
| F | 3.51112300 | 3.23615300 | 1.19795600 |
| F | 4.68854200 | 3.03934300 | -0.59714100 |
| H | 0.03000200 | -3.69167800 | 3.30317000 |

67

f-1-1 carbene-Au fragment

| | | | |
|---|---|---|---|
| Au | -0.02859100 | -2.28609800 | -1.18927800 |
| C | -1.15071400 | 1.34683800 | -1.37261700 |
| N | 0.04529900 | 0.21740700 | 0.45830000 |
| C | 0.15540600 | 0.86787500 | 1.81461100 |
| C | -1.11526400 | 2.06764200 | -2.56591800 |
| H | -2.05431500 | 2.35914000 | -3.02068400 |
| C | -0.21604600 | -1.06976800 | 0.47205300 |
| C | 1.28200300 | 1.27784800 | -1.38353000 |
| C | 0.08713000 | 2.38634900 | -3.17384300 |
| C | 0.05779200 | 0.95628100 | -0.77828800 |
| C | 0.31261400 | -0.37230900 | 2.69998100 |
| H | -0.19395900 | -0.24586300 | 3.65864700 |
| H | 1.37724000 | -0.53544200 | 2.89658100 |
| C | 1.27404000 | 1.99270200 | -2.58152900 |
| H | 2.22179900 | 2.21804500 | -3.05531300 |
| C | -1.11959300 | 1.65437500 | 2.10096500 |
| H | -1.21024400 | 2.50139400 | 1.41671500 |
| H | -1.07158800 | 2.04723600 | 3.11756700 |
| H | -2.01409500 | 1.03695900 | 2.00648200 |
| C | 1.34250500 | 1.81001700 | 1.93199100 |

| | | | |
|---|---|---|---|
| H | 2.29246300 | 1.28512300 | 1.82667200 |
| H | 1.31583800 | 2.26772100 | 2.92250300 |
| H | 1.28933800 | 2.61301900 | 1.19253900 |
| C | -0.23556300 | -1.56326300 | 1.89399300 |
| C | -1.68130300 | -1.92916400 | 2.24555300 |
| H | -2.05396100 | -2.68217400 | 1.54741400 |
| H | -1.71419000 | -2.34452200 | 3.25536100 |
| H | -2.34977500 | -1.06756500 | 2.20832100 |
| C | 0.63830900 | -2.80487800 | 2.04674000 |
| H | 0.67940100 | -3.09343600 | 3.10001700 |
| H | 0.23024000 | -3.63398400 | 1.46646700 |
| H | 1.65616400 | -2.61661700 | 1.70005000 |
| C | 2.60073700 | 0.94610400 | -0.78750500 |
| C | 3.53198400 | 1.96906900 | -0.63730700 |
| C | 2.94282300 | -0.33829500 | -0.35899200 |
| C | 4.76030200 | 1.72933700 | -0.03492600 |
| H | 3.28249900 | 2.97464100 | -0.95905000 |
| C | 4.17179100 | -0.56423700 | 0.24079900 |
| H | 2.25615300 | -1.16413100 | -0.51967700 |
| C | 5.08881900 | 0.46524600 | 0.42113700 |
| H | 6.04352200 | 0.27743200 | 0.89366100 |
| C | -2.48909700 | 1.07813300 | -0.78095400 |
| C | -3.28647200 | 2.15938600 | -0.42381500 |
| C | -2.99312700 | -0.21350800 | -0.61244000 |
| C | -4.53835300 | 1.96048200 | 0.14675000 |
| H | -2.91577100 | 3.16944800 | -0.56467500 |
| C | -4.24610400 | -0.39832600 | -0.05053000 |
| H | -2.40772900 | -1.06796000 | -0.93262600 |
| C | -5.02548300 | 0.68191300 | 0.34895700 |
| H | -5.99949700 | 0.52333200 | 0.79370900 |
| C | -5.32830100 | 3.17212500 | 0.55810600 |
| C | -4.80279000 | -1.78225400 | 0.15589300 |
| F | -6.53297800 | 2.85532500 | 1.03162100 |
| F | -4.68947400 | 3.86334200 | 1.51301700 |

| | | | |
|---|---|---|---|
| F | -5.50344300 | 4.01183200 | -0.46918900 |
| F | -4.96710400 | -2.04416200 | 1.46196600 |
| F | -4.01326800 | -2.72892700 | -0.34621900 |
| F | -6.00690700 | -1.91130600 | -0.41294300 |
| C | 5.70721300 | 2.88729800 | 0.12571700 |
| C | 4.51519400 | -1.93433600 | 0.76293800 |
| F | 5.16624700 | 3.85305800 | 0.88101800 |
| F | 5.99972100 | 3.44391500 | -1.05576900 |
| F | 6.85554500 | 2.52677500 | 0.69777000 |
| F | 5.81547600 | -2.20522900 | 0.62230300 |
| F | 3.82799000 | -2.89586900 | 0.15091300 |
| F | 4.24165100 | -2.02467300 | 2.07661200 |
| H | 0.09826400 | 2.92923200 | -4.10961500 |

72

f-2-1 carbene fragment

| | | | |
|---|---|---|---|
| C | -1.14106600 | -1.64455700 | 1.14564300 |
| N | 0.01290900 | -0.13406000 | -0.40572700 |
| C | 0.18782900 | -0.56771500 | -1.84334800 |
| C | -1.09766300 | -2.71209800 | 2.03986500 |
| H | -2.03208700 | -3.11570600 | 2.41119300 |
| C | -0.27770600 | 1.12147500 | -0.16576100 |
| C | 1.28276200 | -1.56491800 | 1.15608100 |
| C | 0.11360300 | -3.22667600 | 2.47481500 |
| C | 0.05608900 | -1.11295800 | 0.64740600 |
| C | 0.07510100 | 0.77912500 | -2.57228800 |
| H | -0.71509800 | 0.73784200 | -3.32319400 |
| H | 1.00758800 | 0.99315100 | -3.10031000 |
| C | 1.29122500 | -2.63123000 | 2.05712900 |
| H | 2.24230600 | -2.96780000 | 2.45262400 |
| C | -0.92877400 | -1.53007100 | -2.22239300 |
| H | -0.89969400 | -2.43292700 | -1.60563500 |
| H | -0.80547100 | -1.83013000 | -3.26450900 |
| H | -1.90592000 | -1.05612600 | -2.11447100 |
| C | 1.53649600 | -1.22610500 | -2.08973400 |

| | | | |
|---|---|---|---|
| H | 2.35748000 | -0.55037600 | -1.83885600 |
| H | 1.61103400 | -1.47259600 | -3.15052100 |
| H | 1.64623700 | -2.15361000 | -1.52248000 |
| C | -0.19856100 | 1.84896400 | -1.49179800 |
| C | -1.48921100 | 2.64188300 | -1.71964000 |
| C | 1.01274000 | 2.78999100 | -1.33874000 |
| H | 0.89741900 | 3.35994100 | -0.41242700 |
| H | -1.37088700 | 3.28179500 | -2.59866800 |
| C | -2.72165000 | 1.76214000 | -1.89677800 |
| H | -2.74021300 | 1.27733000 | -2.87504300 |
| H | -2.74974600 | 0.97562000 | -1.13836100 |
| H | -3.63911700 | 2.34451900 | -1.80348000 |
| H | -1.62343000 | 3.29919100 | -0.85488000 |
| C | 1.24460000 | 3.73190000 | -2.51188500 |
| H | 0.48126000 | 4.50884300 | -2.56080500 |
| H | 2.21224500 | 4.22551700 | -2.41354700 |
| H | 1.23892100 | 3.19712000 | -3.46539500 |
| H | 1.89781100 | 2.15504900 | -1.20582600 |
| H | 0.13735200 | -4.05444000 | 3.17120900 |
| C | 2.57886800 | -0.92011000 | 0.83399000 |
| C | 3.67550400 | -1.69601300 | 0.47773700 |
| C | 2.73626500 | 0.46563800 | 0.93095100 |
| C | 4.89870500 | -1.10255300 | 0.18967300 |
| H | 3.57003900 | -2.77253700 | 0.39313200 |
| C | 3.96345200 | 1.04355600 | 0.65083100 |
| H | 1.89549500 | 1.08311600 | 1.22724700 |
| C | 5.05411500 | 0.26944700 | 0.26756000 |
| H | 6.00723900 | 0.73251700 | 0.04794600 |
| C | -2.47119600 | -1.05195400 | 0.84017700 |
| C | -3.41960700 | -1.73401700 | 0.09008600 |
| C | -2.81319900 | 0.17915400 | 1.40281800 |
| C | -4.67806700 | -1.18097400 | -0.12289300 |
| H | -3.17550700 | -2.69765200 | -0.34408500 |
| C | -4.07901600 | 0.70825500 | 1.20317100 |

| | | | |
|---|---|---|---|
| H | -2.07991400 | 0.72277400 | 1.98437800 |
| C | -5.02087400 | 0.04091300 | 0.43084100 |
| H | -6.00177300 | 0.46522300 | 0.26733000 |
| C | 6.03793300 | -1.99424600 | -0.22198700 |
| C | 4.15581700 | 2.53475000 | 0.74078800 |
| C | -5.64514100 | -1.94687300 | -0.98168000 |
| C | -4.39063800 | 2.06538500 | 1.77249600 |
| F | -5.88111500 | -3.16822800 | -0.48479100 |
| F | -6.81970600 | -1.32841200 | -1.10217900 |
| F | -5.70612600 | 2.28958900 | 1.83985000 |
| F | -3.89164200 | 2.21228400 | 3.00261000 |
| F | -5.15562000 | -2.12611300 | -2.21706500 |
| F | -3.86338900 | 3.04012200 | 1.01860000 |
| F | 5.75348100 | -2.65330500 | -1.35323800 |
| F | 6.28922700 | -2.92323200 | 0.70902300 |
| F | 7.16568900 | -1.31405200 | -0.42715700 |
| F | 5.25108700 | 2.83994400 | 1.44663900 |
| F | 3.12186800 | 3.14727000 | 1.31380200 |
| F | 4.32220800 | 3.07810000 | -0.47512500 |

73

f-2-1 carbene-Au fragment

| | | | |
|---|---|---|---|
| Au | -0.48606000 | -2.00712300 | -0.71182700 |
| C | -1.01361300 | 1.60264300 | -1.48984800 |
| N | 0.14277900 | 0.70361600 | 0.49158300 |
| C | 0.43841700 | 1.54215700 | 1.71677500 |
| C | -0.95724300 | 2.20653100 | -2.74403800 |
| H | -1.88808000 | 2.46806400 | -3.23212800 |
| C | -0.20839800 | -0.53358600 | 0.72382800 |
| C | 1.40900000 | 1.41333100 | -1.50833800 |
| C | 0.25527800 | 2.45329400 | -3.36668800 |
| C | 0.18178500 | 1.24945800 | -0.84603200 |
| C | 0.14009600 | 0.54599700 | 2.84854900 |
| H | -0.74063600 | 0.86930400 | 3.40635500 |
| H | 0.97487100 | 0.50833800 | 3.55110500 |

| | | | |
|---|---|---|---|
| C | 1.42460500 | 2.03733300 | -2.75653300 |
| H | 2.37565600 | 2.15322300 | -3.26234900 |
| C | -0.49539900 | 2.73915200 | 1.76801200 |
| H | -0.40257000 | 3.35850200 | 0.87179400 |
| H | -0.23330800 | 3.35217700 | 2.63182000 |
| H | -1.53002400 | 2.41520100 | 1.88434000 |
| C | 1.87949900 | 2.02850100 | 1.75070800 |
| H | 2.59149300 | 1.20174700 | 1.70499700 |
| H | 2.03172400 | 2.55930100 | 2.69197500 |
| H | 2.08520800 | 2.72694100 | 0.93770100 |
| C | -0.09420000 | -0.82692600 | 2.19398100 |
| C | -1.35699500 | -1.53206500 | 2.69888700 |
| C | 1.14798000 | -1.73056600 | 2.34601300 |
| H | 1.06057600 | -2.56373400 | 1.64120200 |
| H | -1.29254200 | -1.60928000 | 3.78820100 |
| C | -2.63993100 | -0.82448800 | 2.28618500 |
| H | -2.64950600 | 0.22644500 | 2.59498800 |
| H | -2.74902800 | -0.85627600 | 1.20030300 |
| H | -3.51665000 | -1.30310400 | 2.72284600 |
| H | -1.35710400 | -2.55191100 | 2.29869900 |
| C | 1.35606200 | -2.26216700 | 3.75613700 |
| H | 0.58085500 | -2.97905100 | 4.02802400 |
| H | 2.31807800 | -2.77039500 | 3.82520500 |
| H | 1.34786300 | -1.46063900 | 4.49922600 |
| H | 2.02408700 | -1.14748600 | 2.03704600 |
| H | 0.28440300 | 2.92725900 | -4.33879800 |
| C | 2.70387000 | 0.90231100 | -0.99178800 |
| C | 3.80551600 | 1.74800900 | -0.93497800 |
| C | 2.85619400 | -0.42969700 | -0.60017600 |
| C | 5.02345900 | 1.29140300 | -0.44671100 |
| H | 3.70618100 | 2.78453600 | -1.24040600 |
| C | 4.07791500 | -0.87480900 | -0.12201100 |
| H | 2.02054000 | -1.11894700 | -0.68681600 |
| C | 5.16992600 | -0.01860200 | -0.02682700 |

| | | | |
|---|---|---|---|
| H | 6.11815500 | -0.37602000 | 0.35308200 |
| C | -2.37143500 | 1.27083100 | -0.96578800 |
| C | -3.10079500 | 2.14220900 | -0.17115600 |
| C | -2.97547600 | 0.08374700 | -1.38832700 |
| C | -4.37840800 | 1.79968400 | 0.26160400 |
| H | -2.68585200 | 3.10219700 | 0.10875800 |
| C | -4.25613700 | -0.23768600 | -0.96809400 |
| H | -2.43354200 | -0.59066100 | -2.04279500 |
| C | -4.96509800 | 0.60800500 | -0.12325900 |
| H | -5.95832100 | 0.34511600 | 0.21471800 |
| C | 6.16525500 | 2.26783000 | -0.37206400 |
| C | 4.25708100 | -2.30064500 | 0.33131300 |
| C | -5.07851400 | 2.75405400 | 1.18681000 |
| C | -4.85191800 | -1.56490200 | -1.35684200 |
| F | -5.15474900 | 3.98361900 | 0.66325900 |
| F | -6.31621800 | 2.36353100 | 1.48496700 |
| F | -6.18737800 | -1.50361700 | -1.42064000 |
| F | -4.40816500 | -1.98372700 | -2.54088800 |
| F | -4.40546800 | 2.87490000 | 2.34401700 |
| F | -4.54716400 | -2.50814500 | -0.45728500 |
| F | 5.86816800 | 3.29749800 | 0.43302900 |
| F | 6.43928400 | 2.78902700 | -1.57408900 |
| F | 7.28248800 | 1.70740800 | 0.08959400 |
| F | 5.35907500 | -2.84308900 | -0.19779500 |
| F | 3.22487700 | -3.07194100 | 0.00604800 |
| F | 4.40374700 | -2.36496000 | 1.66626500 |

81

f-3-1 carbene fragment

| | | | |
|---|---|---|---|
| C | -1.16373500 | -2.05873200 | 1.14729500 |
| N | 0.04881500 | -0.32379200 | -0.11679000 |
| C | 0.04997100 | -0.46430500 | -1.61623300 |
| C | -1.13208100 | -3.21115100 | 1.93518700 |
| H | -2.07237300 | -3.64563400 | 2.25331600 |
| C | 0.04172500 | 0.88237000 | 0.39697000 |

| | | | |
|---|---|---|---|
| C | 1.26453200 | -2.02994800 | 1.15865800 |
| C | 0.06706700 | -3.77672000 | 2.33116300 |
| C | 0.04459000 | -1.47644000 | 0.74188500 |
| C | 0.30444000 | 0.98768100 | -2.02222100 |
| H | -0.32424200 | 1.26548100 | -2.86916700 |
| H | 1.34642800 | 1.09631600 | -2.34029400 |
| C | 1.25617800 | -3.18039100 | 1.94761400 |
| H | 2.20390900 | -3.59083300 | 2.27535600 |
| C | -1.29769200 | -0.97946200 | -2.10565300 |
| H | -1.47714900 | -2.00138700 | -1.76399100 |
| H | -1.29770000 | -0.98760400 | -3.19697600 |
| H | -2.11737800 | -0.34328500 | -1.76273400 |
| C | 1.15073700 | -1.39641800 | -2.09833400 |
| H | 2.13651100 | -1.01250600 | -1.83251700 |
| H | 1.09745400 | -1.47078100 | -3.18604400 |
| H | 1.03566100 | -2.40321600 | -1.68701700 |
| C | 2.59015800 | -1.46748500 | 0.79349600 |
| C | 3.53534000 | -2.30967600 | 0.21815800 |
| C | 2.94238200 | -0.13925500 | 1.05483400 |
| C | 4.79771500 | -1.83677700 | -0.12003200 |
| H | 3.27547100 | -3.34146400 | 0.00619400 |
| C | 4.21091300 | 0.31371300 | 0.72912700 |
| H | 2.22425500 | 0.52518200 | 1.52334400 |
| C | 5.14684100 | -0.52287800 | 0.12900500 |
| H | 6.13236500 | -0.15342800 | -0.12296600 |
| C | -2.50495300 | -1.53036800 | 0.78622600 |
| C | -3.41144600 | -2.38952600 | 0.17313800 |
| C | -2.91244600 | -0.23057900 | 1.09651800 |
| C | -4.68798700 | -1.95765700 | -0.15888300 |
| H | -3.10858800 | -3.40291000 | -0.06868100 |
| C | -4.20251500 | 0.17815600 | 0.78708100 |
| H | -2.22292900 | 0.44422500 | 1.59234200 |
| C | -5.09577400 | -0.67128900 | 0.14417400 |
| H | -6.09683300 | -0.33814500 | -0.09411200 |

| | | | |
|---|---|---|---|
| C | -5.60350800 | -2.92170700 | -0.86195500 |
| C | -4.67485500 | 1.56576300 | 1.13436500 |
| F | -6.83036700 | -2.42725600 | -1.02670200 |
| F | -5.13214300 | -3.23989700 | -2.07525800 |
| F | -5.71706200 | -4.06970200 | -0.18249900 |
| F | -5.96068900 | 1.56214900 | 1.50429300 |
| F | -4.58231900 | 2.39458000 | 0.08370500 |
| F | -3.97269100 | 2.10277400 | 2.13105300 |
| C | 5.75965800 | -2.78968200 | -0.77458100 |
| C | 4.63041600 | 1.73070900 | 1.01892600 |
| F | 5.30046400 | -3.20131200 | -1.96409300 |
| F | 5.93691100 | -3.88936600 | -0.03156500 |
| F | 6.95800100 | -2.24171500 | -0.97590400 |
| F | 5.80234500 | 1.76373500 | 1.66532800 |
| F | 3.74090200 | 2.38327400 | 1.76446700 |
| F | 4.79948500 | 2.43086300 | -0.11253300 |
| H | 0.07499800 | -4.66336000 | 2.95123200 |
| C | 0.06276800 | 1.85214300 | -0.76528200 |
| C | -1.27856900 | 2.62863600 | -0.78771800 |
| C | 1.19025300 | 2.89051400 | -0.57798700 |
| H | -2.10032600 | 1.90916600 | -0.90439700 |
| C | -1.29682800 | 3.62407600 | -1.95049200 |
| C | -1.46150700 | 3.41720200 | 0.51412100 |
| H | 2.14600200 | 2.35097800 | -0.55567800 |
| C | 1.18083800 | 3.86322100 | -1.75963900 |
| C | 1.00832800 | 3.67801500 | 0.71874700 |
| H | -2.25445000 | 4.15601700 | -1.94036700 |
| H | -1.22517000 | 3.11495200 | -2.91572700 |
| C | -0.14741000 | 4.62085800 | -1.79721000 |
| H | -1.47984800 | 2.74183500 | 1.37180500 |
| H | -2.42027500 | 3.94463400 | 0.47776400 |
| C | -0.32357500 | 4.42448200 | 0.68001200 |
| H | 1.33318900 | 3.32905200 | -2.70373200 |
| H | 2.01028500 | 4.56966800 | -1.64992900 |

| | | | |
|---|---|---|---|
| H | 1.03419700 | 3.00188500 | 1.57585100 |
| H | 1.83530000 | 4.38925400 | 0.82301100 |
| H | -0.14946800 | 5.31171800 | -2.64534600 |
| C | -0.32641200 | 5.39976800 | -0.49548800 |
| H | -0.46222300 | 4.97383600 | 1.61539200 |
| H | 0.48466100 | 6.12720700 | -0.38008600 |
| H | -1.27032900 | 5.95537700 | -0.52090300 |

82

f-3-1 carbene-Au fragment

| | | | |
|---|---|---|---|
| Au | 0.07811900 | 1.23068900 | -1.87366400 |
| C | 0.60036600 | -2.30307500 | -1.04673000 |
| N | -0.35427400 | -0.57887100 | 0.40817800 |
| C | -0.60571600 | -0.79454300 | 1.87542700 |
| C | 0.43295700 | -3.32615000 | -1.97689900 |
| H | 1.31087600 | -3.84971500 | -2.33515400 |
| C | 0.21211200 | 0.57027100 | 0.08560600 |
| C | -1.80220200 | -1.91763300 | -1.07902200 |
| C | -0.82584800 | -3.65785800 | -2.45230900 |
| C | -0.52672100 | -1.61388200 | -0.57632500 |
| C | -0.42021100 | 0.63060300 | 2.37806700 |
| H | -0.02460600 | 0.63577300 | 3.39225600 |
| H | -1.39807900 | 1.12721100 | 2.40074400 |
| C | -1.92850000 | -2.94888900 | -2.01052400 |
| H | -2.91149200 | -3.16709600 | -2.41023600 |
| C | 0.41480100 | -1.76940700 | 2.45094600 |
| H | 0.31082000 | -2.74935700 | 1.97869200 |
| H | 0.22007600 | -1.89153100 | 3.51747200 |
| H | 1.43904300 | -1.42246300 | 2.32820000 |
| C | -1.99127000 | -1.33370900 | 2.20023100 |
| H | -2.78579600 | -0.65456000 | 1.89238500 |
| H | -2.05237700 | -1.45283100 | 3.28358900 |
| H | -2.15585800 | -2.31537300 | 1.75003400 |
| C | -3.03234400 | -1.19908500 | -0.66479800 |
| C | -4.15571900 | -1.93249000 | -0.29656400 |

| | | | |
|---|---|---|---|
| C | -3.09606200 | 0.19374100 | -0.60112400 |
| C | -5.29889400 | -1.29359800 | 0.16707200 |
| H | -4.12766800 | -3.01650800 | -0.32960100 |
| C | -4.23546900 | 0.81892000 | -0.12274000 |
| H | -2.25235800 | 0.79024100 | -0.93397500 |
| C | -5.34716400 | 0.08571900 | 0.27542800 |
| H | -6.23182700 | 0.58147700 | 0.65137100 |
| C | 1.99901900 | -1.97503400 | -0.63914800 |
| C | 2.65538900 | -2.71789200 | 0.33148000 |
| C | 2.70980200 | -0.98006100 | -1.31557000 |
| C | 3.97068600 | -2.42274900 | 0.67870300 |
| H | 2.14155900 | -3.52714800 | 0.83658700 |
| C | 4.03186700 | -0.72230700 | -0.99135700 |
| H | 2.22007300 | -0.41643900 | -2.10249500 |
| C | 4.66946200 | -1.42387600 | 0.02615700 |
| H | 5.69521300 | -1.20228700 | 0.28999700 |
| C | 4.59211200 | -3.20861800 | 1.79880700 |
| C | 4.82992700 | 0.28189300 | -1.78083200 |
| F | 5.87292400 | -2.89631500 | 1.99165900 |
| F | 3.94617800 | -2.98680600 | 2.95484000 |
| F | 4.52446100 | -4.52578800 | 1.57198600 |
| F | 5.63298900 | -0.33269900 | -2.66081600 |
| F | 5.62203400 | 1.01465300 | -0.98801300 |
| F | 4.05600100 | 1.11870600 | -2.46977500 |
| C | -6.46719800 | -2.14804700 | 0.57722100 |
| C | -4.23539400 | 2.31343200 | 0.05738100 |
| F | -6.13548200 | -2.97343300 | 1.57954200 |
| F | -6.88293600 | -2.92137500 | -0.43323000 |
| F | -7.50817300 | -1.42194200 | 0.98414900 |
| F | -5.45440700 | 2.83587600 | -0.09812200 |
| F | -3.41366100 | 2.92618100 | -0.79139100 |
| F | -3.83056900 | 2.63698100 | 1.29977900 |
| H | -0.94230000 | -4.44806800 | -3.18211200 |
| C | 0.45635600 | 1.37100600 | 1.34544400 |

| | | | |
|---|---|---|---|
| C | 1.98584500 | 1.31884200 | 1.63436200 |
| C | 0.05402400 | 2.86263100 | 1.30070900 |
| H | 2.33208200 | 0.27807300 | 1.59220500 |
| C | 2.27307400 | 1.91884700 | 3.01162700 |
| C | 2.75612200 | 2.12885000 | 0.58582600 |
| H | -1.01274000 | 2.91906700 | 1.05035400 |
| C | 0.30013300 | 3.44971200 | 2.69806600 |
| C | 0.86022900 | 3.69219300 | 0.30316500 |
| H | 3.35417700 | 1.89080300 | 3.18545200 |
| H | 1.81209300 | 1.33412100 | 3.81159900 |
| C | 1.79064600 | 3.37082200 | 3.04758600 |
| H | 2.56287200 | 1.73535700 | -0.41645000 |
| H | 3.82856700 | 2.02602500 | 0.78491500 |
| C | 2.34599700 | 3.59895600 | 0.63224300 |
| H | -0.29738300 | 2.93419800 | 3.45553200 |
| H | -0.01819200 | 4.49699300 | 2.70024500 |
| H | 0.67882700 | 3.36048400 | -0.72392300 |
| H | 0.52333300 | 4.73250100 | 0.36956500 |
| H | 1.94941700 | 3.78180200 | 4.04850100 |
| C | 2.59433100 | 4.17278800 | 2.02527100 |
| H | 2.92247500 | 4.15766900 | -0.10949600 |
| H | 2.29338300 | 5.22549900 | 2.05205900 |
| H | 3.66081100 | 4.12542500 | 2.27114700 |

73

f-4-1 carbene fragment

| | | | |
|---|---|---|---|
| C | 1.14577600 | -1.83398500 | -1.08155000 |
| N | -0.04815800 | -0.05635100 | 0.13405400 |
| C | -0.05149000 | -0.16536700 | 1.63836900 |
| C | 1.10626000 | -3.02858200 | -1.80233600 |
| H | 2.04272600 | -3.47905600 | -2.10942700 |
| C | 0.00420200 | 1.14053400 | -0.40180100 |
| C | -1.28375400 | -1.79906400 | -1.07632700 |
| C | -0.09828900 | -3.61510300 | -2.15093100 |
| C | -0.05954400 | -1.23435900 | -0.68933100 |

| | | | |
|---|---|---|---|
| C | -0.22946000 | 1.30785700 | 2.02229600 |
| H | 0.43094700 | 1.57559900 | 2.84997600 |
| H | -1.25771000 | 1.47876400 | 2.35619000 |
| C | -1.28388500 | -2.99212000 | -1.79934200 |
| H | -2.23427500 | -3.41360800 | -2.10469000 |
| C | 1.26807700 | -0.74635100 | 2.13074500 |
| H | 1.39645600 | -1.77620600 | 1.78821900 |
| H | 1.26712300 | -0.75433000 | 3.22201700 |
| H | 2.11993700 | -0.15428100 | 1.78885500 |
| C | -1.20007300 | -1.02736100 | 2.13889400 |
| H | -2.16365700 | -0.60244900 | 1.85399700 |
| H | -1.15687400 | -1.07184900 | 3.22868600 |
| H | -1.13287600 | -2.05018100 | 1.75783600 |
| C | -2.59713100 | -1.18300100 | -0.76148000 |
| C | -3.58898500 | -1.95609200 | -0.16976500 |
| C | -2.88341400 | 0.14674100 | -1.08764600 |
| C | -4.83401700 | -1.40986100 | 0.12230900 |
| H | -3.38189900 | -2.98825700 | 0.09259500 |
| C | -4.13116300 | 0.67431300 | -0.80146700 |
| H | -2.12750700 | 0.75591400 | -1.57030800 |
| C | -5.11590900 | -0.09202700 | -0.18514200 |
| H | -6.08599400 | 0.33383300 | 0.03619800 |
| C | 2.48224600 | -1.25840900 | -0.78354400 |
| C | 3.44117100 | -2.04925600 | -0.16111900 |
| C | 2.82306200 | 0.04409700 | -1.15889100 |
| C | 4.70464500 | -1.54282400 | 0.11947000 |
| H | 3.19313200 | -3.06374500 | 0.13322900 |
| C | 4.09075500 | 0.53178300 | -0.88558100 |
| H | 2.09056600 | 0.66700600 | -1.65957600 |
| C | 5.04021300 | -0.24868300 | -0.23416900 |
| H | 6.02556500 | 0.14573500 | -0.02267900 |
| C | 5.68298200 | -2.43514900 | 0.83226500 |
| C | 4.48121600 | 1.93277600 | -1.27626800 |
| F | 6.88095300 | -1.86678500 | 0.96946100 |

| | | | |
|---|---|---|---|
| F | 5.24563800 | -2.75213100 | 2.05871500 |
| F | 5.85543100 | -3.59036400 | 0.17767100 |
| F | 4.62501700 | 2.71854500 | -0.19791500 |
| F | 3.58289700 | 2.50841400 | -2.07308500 |
| F | 5.65747800 | 1.94659400 | -1.91540600 |
| C | -5.85034300 | -2.28541200 | 0.80224900 |
| C | -4.46964900 | 2.09925400 | -1.15121500 |
| F | -5.44722700 | -2.63053700 | 2.03282300 |
| F | -6.03990600 | -3.42630000 | 0.12785700 |
| F | -7.03515200 | -1.68659800 | 0.92331900 |
| F | -5.59853700 | 2.16620700 | -1.86787600 |
| F | -3.50895000 | 2.69418500 | -1.85496000 |
| F | -4.67520300 | 2.83309500 | -0.04706600 |
| H | -0.11293700 | -4.53647200 | -2.71802300 |
| C | 0.04713200 | 2.12286700 | 0.74352400 |
| C | 1.45118200 | 2.75193500 | 0.73446300 |
| C | -0.99710200 | 3.21929400 | 0.51617400 |
| C | 1.58137600 | 3.90000400 | 1.72833100 |
| H | 1.64966500 | 3.12061100 | -0.27839200 |
| H | 2.19894800 | 1.97956500 | 0.94508600 |
| C | -0.87368500 | 4.33409000 | 1.54639400 |
| H | -0.85449300 | 3.62594700 | -0.49105700 |
| H | -1.99676700 | 2.77214700 | 0.54332200 |
| C | 0.51452400 | 4.95900000 | 1.48096700 |
| H | 2.58297400 | 4.33004300 | 1.65503900 |
| H | 1.47653800 | 3.52144200 | 2.75203200 |
| H | -1.64709600 | 5.08628100 | 1.37829100 |
| H | -1.04019100 | 3.92778200 | 2.55247500 |
| H | 0.60849000 | 5.77268200 | 2.20349200 |
| H | 0.66281900 | 5.39339100 | 0.48516300 |

74

f-4-1 carbene-Au fragment

| | | | |
|---|---|---|---|
| Au | -0.00740400 | -1.77143700 | -1.79358600 |
| C | -1.07039300 | 1.82219200 | -1.23765300 |

| | | | |
|---|---|---|---|
| N | 0.08492400 | 0.33153900 | 0.34444000 |
| C | 0.17344900 | 0.69353600 | 1.80408800 |
| C | -1.00701900 | 2.76652100 | -2.26199400 |
| H | -1.93505400 | 3.15647800 | -2.66238000 |
| C | -0.17364800 | -0.93006000 | 0.09313000 |
| C | 1.36166400 | 1.72024500 | -1.23155300 |
| C | 0.20849800 | 3.18318800 | -2.77750800 |
| C | 0.12446700 | 1.30325200 | -0.71838700 |
| C | 0.28637300 | -0.70033100 | 2.43187500 |
| H | -0.26126700 | -0.74882500 | 3.37461800 |
| H | 1.33892100 | -0.91495500 | 2.64628900 |
| C | 1.38138300 | 2.66121400 | -2.26118700 |
| H | 2.33915600 | 2.96252600 | -2.66788100 |
| C | -1.09482500 | 1.43092100 | 2.21978400 |
| H | -1.16673800 | 2.39396200 | 1.70881900 |
| H | -1.05608100 | 1.62166600 | 3.29324100 |
| H | -1.99404900 | 0.85276900 | 1.99995400 |
| C | 1.37484300 | 1.56631200 | 2.12845700 |
| H | 2.31617500 | 1.05281800 | 1.92968900 |
| H | 1.33993600 | 1.81151000 | 3.19144500 |
| H | 1.34976400 | 2.50464200 | 1.56862300 |
| C | 2.66678400 | 1.25159800 | -0.70049100 |
| C | 3.60706200 | 2.20599000 | -0.32578900 |
| C | 2.98786600 | -0.09961200 | -0.55096400 |
| C | 4.82369700 | 1.82637300 | 0.22693600 |
| H | 3.37399800 | 3.26032700 | -0.43080500 |
| C | 4.20560400 | -0.46511200 | 0.00059800 |
| H | 2.29422000 | -0.86248400 | -0.89158800 |
| C | 5.13124900 | 0.48988100 | 0.40705200 |
| H | 6.07710600 | 0.19229900 | 0.83954900 |
| C | -2.42147400 | 1.46094500 | -0.72949000 |
| C | -3.21454200 | 2.46451500 | -0.18445700 |
| C | -2.94053900 | 0.16728100 | -0.81623200 |
| C | -4.47735100 | 2.17669100 | 0.31969500 |

| | | | |
|---|---|---|---|
| H | -2.83182300 | 3.47816200 | -0.12519800 |
| C | -4.20446800 | -0.10550400 | -0.31715600 |
| H | -2.35787200 | -0.61811500 | -1.28497700 |
| C | -4.97990000 | 0.88894200 | 0.26870000 |
| H | -5.96278700 | 0.66142000 | 0.66075600 |
| C | -5.26098300 | 3.29687400 | 0.94608800 |
| C | -4.77356400 | -1.49867300 | -0.37807900 |
| F | -6.48481600 | 2.91691900 | 1.31198700 |
| F | -4.64207500 | 3.76602300 | 2.03916700 |
| F | -5.39047500 | 4.33105800 | 0.10666900 |
| F | -4.89909000 | -2.02221900 | 0.85284200 |
| F | -4.01465000 | -2.32861000 | -1.08915600 |
| F | -5.99763800 | -1.50041500 | -0.91769000 |
| C | 5.77922600 | 2.91029600 | 0.64542000 |
| C | 4.53363700 | -1.91878000 | 0.21799900 |
| F | 5.24420600 | 3.68401100 | 1.60000600 |
| F | 6.07806600 | 3.71787900 | -0.37913100 |
| F | 6.92377200 | 2.42082500 | 1.12125800 |
| F | 5.82473400 | -2.17250500 | -0.01397900 |
| F | 3.81253700 | -2.72298000 | -0.55894800 |
| F | 4.29441200 | -2.27564500 | 1.49248900 |
| H | 0.24062000 | 3.90225000 | -3.58525100 |
| C | -0.22732400 | -1.70126900 | 1.38028400 |
| C | -1.68753500 | -2.14066900 | 1.59767600 |
| C | 0.65123400 | -2.95402500 | 1.29355900 |
| C | -1.84231900 | -3.03628000 | 2.82132400 |
| H | -2.00418800 | -2.68496400 | 0.69917600 |
| H | -2.33597100 | -1.26402500 | 1.68443500 |
| C | 0.52514000 | -3.79893200 | 2.55390800 |
| H | 0.33169100 | -3.53844900 | 0.42317200 |
| H | 1.68888100 | -2.65702500 | 1.11479300 |
| C | -0.91974000 | -4.24567000 | 2.74022300 |
| H | -2.88600100 | -3.34669100 | 2.90649800 |
| H | -1.60995000 | -2.46807000 | 3.72967900 |

| | | | |
|---|---|---|---|
| H | 1.19257300 | -4.66004600 | 2.48948000 |
| H | 0.84475500 | -3.21608000 | 3.42720900 |
| H | -1.02321700 | -4.86428600 | 3.63405700 |
| H | -1.21251300 | -4.86419300 | 1.88401100 |

71

f-5-1 carbene fragment

| | | | |
|---|---|---|---|
| C | 1.15426700 | -1.78535500 | -1.09951500 |
| N | -0.04275300 | -0.00372700 | 0.10541700 |
| C | -0.03953300 | -0.10352000 | 1.61260600 |
| C | 1.11838300 | -2.98044900 | -1.81972800 |
| H | 2.05630800 | -3.42983600 | -2.12390800 |
| C | -0.00348400 | 1.19039300 | -0.43874700 |
| C | -1.27515100 | -1.75633000 | -1.09605800 |
| C | -0.08433000 | -3.56921300 | -2.17046400 |
| C | -0.05262500 | -1.18647300 | -0.71143500 |
| C | -0.24185700 | 1.36913600 | 1.98167500 |
| H | 0.38846400 | 1.66893900 | 2.82221200 |
| H | -1.28500700 | 1.52466000 | 2.27612000 |
| C | -1.27160200 | -2.94972500 | -1.81852800 |
| H | -2.22085200 | -3.37486300 | -2.12231800 |
| C | 1.28403700 | -0.67008500 | 2.11109300 |
| H | 1.41378900 | -1.70539300 | 1.78597200 |
| H | 1.28381500 | -0.66000700 | 3.20227400 |
| H | 2.13570700 | -0.08446500 | 1.75954200 |
| C | -1.17705700 | -0.97722100 | 2.11906900 |
| H | -2.14643500 | -0.56637200 | 1.83371500 |
| H | -1.13147000 | -1.01532900 | 3.20899300 |
| H | -1.09730500 | -2.00116200 | 1.74346500 |
| C | -2.59131100 | -1.14991400 | -0.77430600 |
| C | -3.57175800 | -1.93181800 | -0.17498900 |
| C | -2.89192400 | 0.17703600 | -1.09844700 |
| C | -4.81929600 | -1.39694300 | 0.12669000 |
| H | -3.35328100 | -2.96204100 | 0.08572100 |
| C | -4.14222500 | 0.69335100 | -0.80233400 |

| | | | |
|---|---|---|---|
| H | -2.14568000 | 0.79213300 | -1.58840700 |
| C | -5.11524900 | -0.08162200 | -0.17831800 |
| H | -6.08744900 | 0.33541700 | 0.05025600 |
| C | 2.48991900 | -1.21273500 | -0.79214800 |
| C | 3.43880700 | -2.00785700 | -0.15961100 |
| C | 2.84041600 | 0.08806500 | -1.16437400 |
| C | 4.70094100 | -1.50717200 | 0.13558300 |
| H | 3.18273200 | -3.02111200 | 0.13199700 |
| C | 4.10820000 | 0.56928300 | -0.87867400 |
| H | 2.11681300 | 0.71259500 | -1.67596500 |
| C | 5.04629300 | -0.21494300 | -0.21534500 |
| H | 6.03134900 | 0.17473500 | 0.00584600 |
| C | 5.66686600 | -2.40326400 | 0.86069700 |
| C | 4.51293300 | 1.96631700 | -1.26915900 |
| F | 6.86629100 | -1.84102000 | 1.00919900 |
| F | 5.21459200 | -2.71472400 | 2.08303300 |
| F | 5.83966900 | -3.56077900 | 0.21046100 |
| F | 4.62128000 | 2.76212300 | -0.19384900 |
| F | 3.64168500 | 2.53502900 | -2.10078200 |
| F | 5.70921500 | 1.97353300 | -1.86902800 |
| C | -5.82255300 | -2.28192000 | 0.81392800 |
| C | -4.49434200 | 2.11632700 | -1.14622300 |
| F | -5.40620800 | -2.62498000 | 2.04060000 |
| F | -6.00791800 | -3.42348700 | 0.13959700 |
| F | -7.01128800 | -1.69326900 | 0.94549800 |
| F | -5.64500700 | 2.17952400 | -1.82706200 |
| F | -3.55674500 | 2.71054100 | -1.88124100 |
| F | -4.66549000 | 2.85367600 | -0.03821700 |
| H | -0.09632600 | -4.49060200 | -2.73756500 |
| C | 0.05638500 | 2.17375600 | 0.70407400 |
| C | 1.47079400 | 2.77082700 | 0.70309300 |
| C | -0.94321400 | 3.31190700 | 0.49820500 |
| C | 1.56970500 | 3.98515500 | 1.57703700 |
| H | 1.75135700 | 3.02035600 | -0.32696300 |

| | | | |
|---|---|---|---|
| H | 2.19255900 | 2.01659100 | 1.03723800 |
| C | -0.89901500 | 4.28508000 | 1.66973800 |
| H | -0.69148900 | 3.82645500 | -0.43351200 |
| H | -1.95075100 | 2.90087400 | 0.38130000 |
| C | 0.51051100 | 4.65230800 | 2.02658900 |
| H | 2.56835200 | 4.31496700 | 1.84492000 |
| H | -1.46619100 | 5.18734600 | 1.42753400 |
| H | -1.39870200 | 3.85175900 | 2.54539300 |
| H | 0.65670300 | 5.51065200 | 2.67428300 |

72

f-5-1 carbene-Au fragment

| | | | |
|---|---|---|---|
| Au | -0.01107000 | -1.82332000 | -1.80609700 |
| C | -1.09014300 | 1.76823400 | -1.27541500 |
| N | 0.07111300 | 0.29059700 | 0.31143400 |
| C | 0.17813300 | 0.64827600 | 1.77187600 |
| C | -1.03305200 | 2.69438800 | -2.31620900 |
| H | -1.96304600 | 3.08071300 | -2.71557800 |
| C | -0.21553800 | -0.96846800 | 0.06304700 |
| C | 1.34183800 | 1.66479200 | -1.28068500 |
| C | 0.18013700 | 3.09842600 | -2.84775900 |
| C | 0.10690500 | 1.25552500 | -0.75585700 |
| C | 0.32454300 | -0.74890900 | 2.38221500 |
| H | -0.16699900 | -0.82486100 | 3.35419500 |
| H | 1.39112400 | -0.95990600 | 2.52212100 |
| C | 1.35588800 | 2.58636700 | -2.32789200 |
| H | 2.31119700 | 2.88116400 | -2.74502900 |
| C | -1.09337400 | 1.36923000 | 2.20873300 |
| H | -1.16826000 | 2.34403100 | 1.72071100 |
| H | -1.05339600 | 1.53450000 | 3.28628400 |
| H | -1.99257200 | 0.79736400 | 1.97534300 |
| C | 1.36777300 | 1.54089400 | 2.08763000 |
| H | 2.31796300 | 1.04265800 | 1.89345700 |
| H | 1.32885200 | 1.79437200 | 3.14851400 |
| H | 1.32657400 | 2.47459600 | 1.52101000 |

| | | | |
|---|---|---|---|
| C | 2.64713800 | 1.21526700 | -0.73423100 |
| C | 3.58491800 | 2.18553300 | -0.39299100 |
| C | 2.96750000 | -0.12709000 | -0.52135100 |
| C | 4.79591700 | 1.83168300 | 0.18700900 |
| H | 3.35242100 | 3.23420900 | -0.54479600 |
| C | 4.17787300 | -0.46683900 | 0.06375400 |
| H | 2.27804000 | -0.90550900 | -0.83425000 |
| C | 5.10012600 | 0.50470400 | 0.43477300 |
| H | 6.03972000 | 0.22807200 | 0.89328000 |
| C | -2.43494400 | 1.42121900 | -0.74136200 |
| C | -3.20420400 | 2.43361400 | -0.17969300 |
| C | -2.96875100 | 0.13224600 | -0.81395700 |
| C | -4.45645300 | 2.15913300 | 0.35764600 |
| H | -2.80985400 | 3.44338500 | -0.13236500 |
| C | -4.22254100 | -0.12664100 | -0.28286000 |
| H | -2.40595900 | -0.65834400 | -1.29728000 |
| C | -4.97225900 | 0.87646100 | 0.32212500 |
| H | -5.94698800 | 0.65907300 | 0.73955300 |
| C | -5.21208400 | 3.28755100 | 1.00333700 |
| C | -4.81313200 | -1.51102900 | -0.33298300 |
| F | -6.43140700 | 2.92140200 | 1.39721500 |
| F | -4.56232900 | 3.74760700 | 2.08227500 |
| F | -5.34874500 | 4.32429800 | 0.16834600 |
| F | -4.92847000 | -2.03027500 | 0.90035900 |
| F | -4.07702600 | -2.35353000 | -1.05407200 |
| F | -6.04444500 | -1.49540700 | -0.85524200 |
| C | 5.74964600 | 2.93364000 | 0.56058400 |
| C | 4.48075100 | -1.91011900 | 0.36736100 |
| F | 5.20206500 | 3.76305500 | 1.45933600 |
| F | 6.07122900 | 3.67968500 | -0.50301900 |
| F | 6.88290500 | 2.46691800 | 1.08404900 |
| F | 5.79005000 | -2.16534000 | 0.31550100 |
| F | 3.86767400 | -2.74209400 | -0.47193100 |
| F | 4.07337800 | -2.23041200 | 1.60989200 |

| | | | |
|---|---|---|---|
| H | 0.20819900 | 3.80215900 | -3.66904900 |
| C | -0.24603800 | -1.73746700 | 1.35132300 |
| C | -1.69540000 | -2.16019500 | 1.63435100 |
| C | 0.59175200 | -3.01622100 | 1.25545900 |
| C | -1.76783900 | -3.13857400 | 2.76993700 |
| H | -2.11291000 | -2.60023900 | 0.71982500 |
| H | -2.31505900 | -1.28683700 | 1.86054500 |
| C | 0.64861700 | -3.70816100 | 2.61038800 |
| H | 0.12793100 | -3.67253000 | 0.51281600 |
| H | 1.59555500 | -2.78071500 | 0.89336600 |
| C | -0.71522700 | -3.81256500 | 3.22495100 |
| H | -2.73946900 | -3.27911200 | 3.23152100 |
| H | 1.07965400 | -4.70569400 | 2.49668900 |
| H | 1.32553100 | -3.17296000 | 3.28836100 |
| H | -0.83629100 | -4.48409900 | 4.06844000 |

85

f-6-1 carbene fragment

| | | | |
|---|---|---|---|
| C | -0.83808200 | -2.30929700 | 0.99981500 |
| N | 0.13383800 | -0.44063200 | -0.23835600 |
| C | 0.22284900 | -0.54764400 | -1.74459700 |
| C | -0.70005000 | -3.54184900 | 1.63280100 |
| H | -1.59358100 | -4.06448800 | 1.95250200 |
| C | -0.27728800 | 0.69923100 | 0.27649100 |
| C | 1.57589300 | -2.11567300 | 0.88797400 |
| C | 0.55491000 | -4.07938100 | 1.87415700 |
| C | 0.30522100 | -1.61781600 | 0.56991500 |
| C | -0.04613400 | 0.90868900 | -2.13285600 |
| H | -0.66560500 | 0.96774300 | -3.03020800 |
| H | 0.90602900 | 1.39349500 | -2.35492800 |
| C | 1.68033800 | -3.35288900 | 1.52746600 |
| H | 2.66710000 | -3.72312000 | 1.77950200 |
| C | -0.80639100 | -1.52554800 | -2.29812600 |
| H | -0.68064800 | -2.51629700 | -1.85223900 |
| H | -0.65377100 | -1.62449900 | -3.37427600 |

| | | | |
|---|---|---|---|
| H | -1.82934100 | -1.18923800 | -2.13389900 |
| C | 1.60581000 | -0.98984400 | -2.19837100 |
| H | 2.38090600 | -0.33053500 | -1.80216900 |
| H | 1.64120900 | -0.94203900 | -3.28858700 |
| H | 1.82094900 | -2.01955100 | -1.90186700 |
| C | 2.82661500 | -1.35789000 | 0.65048300 |
| C | 3.92018400 | -1.98136800 | 0.06202100 |
| C | 2.95025900 | -0.03259500 | 1.07646500 |
| C | 5.11117100 | -1.29067400 | -0.12594900 |
| H | 3.83715800 | -3.00878300 | -0.27639200 |
| C | 4.15189700 | 0.63484500 | 0.90659100 |
| H | 2.10985300 | 0.45960300 | 1.55342900 |
| C | 5.23933900 | 0.02062000 | 0.29348500 |
| H | 6.17152100 | 0.55501100 | 0.16476100 |
| C | -2.20836500 | -1.74130100 | 0.86815500 |
| C | -3.12358700 | -2.25421700 | -0.03732900 |
| C | -2.60697400 | -0.70816300 | 1.72322800 |
| C | -4.39804100 | -1.70012000 | -0.13996500 |
| H | -2.83910900 | -3.06869200 | -0.69418900 |
| C | -3.89199700 | -0.20204000 | 1.64612900 |
| H | -1.90015500 | -0.30119200 | 2.43315100 |
| C | -4.79324900 | -0.67406000 | 0.69665400 |
| H | -5.78451400 | -0.24576600 | 0.61784500 |
| C | -5.30238200 | -2.21555600 | -1.22287900 |
| C | -4.34055400 | 0.91129200 | 2.55329200 |
| F | -6.50600300 | -1.64428800 | -1.19787300 |
| F | -4.77293200 | -1.98045000 | -2.43502600 |
| F | -5.47813000 | -3.53926100 | -1.13486400 |
| F | -5.48692700 | 0.60026600 | 3.17187300 |
| F | -4.57486300 | 2.03901800 | 1.86427800 |
| F | -3.44009200 | 1.19742500 | 3.49095400 |
| C | 6.24569800 | -2.00528100 | -0.80764500 |
| C | 4.32849200 | 2.04726400 | 1.39478400 |
| F | 5.94320000 | -2.29120700 | -2.08112500 |

| | | | |
|---|---|---|---|
| F | 6.51666700 | -3.17110100 | -0.20757500 |
| F | 7.36711900 | -1.28487100 | -0.81498900 |
| F | 5.45058700 | 2.17270400 | 2.11370600 |
| F | 3.31433500 | 2.45007400 | 2.15709900 |
| F | 4.43431500 | 2.90853900 | 0.37137300 |
| H | 0.65392900 | -5.03722100 | 2.36760800 |
| C | -0.68454400 | 1.56373700 | -0.90007800 |
| C | -2.22331500 | 1.43958400 | -0.90072100 |
| C | -0.34253800 | 3.04413700 | -0.68821200 |
| C | -2.91106200 | 2.29376600 | -1.95731000 |
| H | -2.58079100 | 1.76954900 | 0.08259500 |
| H | -2.52562000 | 0.39160700 | -1.00429600 |
| C | -1.00682800 | 3.91530300 | -1.75213300 |
| H | -0.81661800 | 3.29101300 | 0.27302100 |
| C | -2.52058400 | 3.74992000 | -1.74345800 |
| H | -2.56306900 | 1.98887700 | -2.95319400 |
| H | -0.73993400 | 4.96360500 | -1.58648900 |
| H | -0.61792700 | 3.64709700 | -2.74311600 |
| H | -2.98577400 | 4.38231000 | -2.50511000 |
| H | -2.91708900 | 4.07205700 | -0.77102600 |
| C | -4.41747200 | 2.09022200 | -1.88214700 |
| H | -4.68571400 | 1.04581400 | -2.06788700 |
| H | -4.78092900 | 2.35811100 | -0.88504300 |
| H | -4.94155800 | 2.71081500 | -2.61174800 |
| C | 1.16635400 | 3.33572300 | -0.51632000 |
| H | 1.64964200 | 2.39892200 | -0.19472400 |
| C | 1.34495800 | 4.37390600 | 0.58778900 |
| H | 0.98259500 | 3.99667700 | 1.54552400 |
| H | 2.39085900 | 4.66397400 | 0.70159900 |
| H | 0.77253400 | 5.27339200 | 0.33705500 |
| C | 1.88366500 | 3.84058200 | -1.76921200 |
| H | 1.52771100 | 4.83940300 | -2.03453200 |
| H | 2.95425300 | 3.91771500 | -1.57286700 |
| H | 1.74783700 | 3.20373100 | -2.64410600 |

86
f-6-1 carbene-Au fragment

| | | | |
|---|---|---|---|
| Au | -0.31891700 | 1.27759200 | -1.69298100 |
| C | 1.54771400 | -2.16071500 | -0.92917100 |
| N | 0.24478300 | -0.67486800 | 0.55480500 |
| C | 0.48703900 | -0.92159100 | 2.02638300 |
| C | 1.58514700 | -3.24954900 | -1.80154500 |
| H | 2.54634700 | -3.56573400 | -2.18862100 |
| C | -0.08351800 | 0.54919600 | 0.23498200 |
| C | -0.87485500 | -2.32913800 | -0.88916600 |
| C | 0.42636500 | -3.88391700 | -2.21016300 |
| C | 0.30826500 | -1.73362900 | -0.42716600 |
| C | 0.19065200 | 0.46228100 | 2.62630800 |
| H | 1.02332200 | 0.78505200 | 3.25358800 |
| H | -0.68930800 | 0.40876400 | 3.26546400 |
| C | -0.79680800 | -3.40236800 | -1.77419200 |
| H | -1.71932000 | -3.84728000 | -2.12649600 |
| C | 1.91448700 | -1.36706900 | 2.30327200 |
| H | 2.12276400 | -2.34268000 | 1.86024800 |
| H | 2.03590400 | -1.45842800 | 3.38391400 |
| H | 2.64348000 | -0.64113700 | 1.93603600 |
| C | -0.48305700 | -1.96490300 | 2.55161100 |
| H | -1.51032800 | -1.60350900 | 2.47937900 |
| H | -0.26305400 | -2.14942500 | 3.60445000 |
| H | -0.38560900 | -2.91165400 | 2.01361800 |
| C | -2.24456000 | -1.82834300 | -0.57126600 |
| C | -2.98287100 | -2.31986100 | 0.49559000 |
| C | -2.85048600 | -0.92402500 | -1.44701800 |
| C | -4.26798800 | -1.84715200 | 0.73978600 |
| H | -2.56178000 | -3.07370400 | 1.14794600 |
| C | -4.15023100 | -0.49691100 | -1.22208200 |
| H | -2.29779500 | -0.55682500 | -2.30586600 |
| C | -4.86362500 | -0.93476900 | -0.11330500 |
| H | -5.86561700 | -0.57346900 | 0.07575900 |

| | | | |
|---|---|---|---|
| C | 2.83940700 | -1.48694900 | -0.64301500 |
| C | 3.92241100 | -2.23983300 | -0.20514300 |
| C | 3.01168300 | -0.11726300 | -0.85813800 |
| C | 5.14449900 | -1.63437200 | 0.06072300 |
| H | 3.80504900 | -3.30531400 | -0.03635800 |
| C | 4.23848400 | 0.47153500 | -0.60071000 |
| H | 2.18988900 | 0.47904500 | -1.24535700 |
| C | 5.31302800 | -0.27487400 | -0.12740000 |
| H | 6.26382700 | 0.19989500 | 0.07781100 |
| C | 6.26540700 | -2.49786100 | 0.57101900 |
| C | 4.46399200 | 1.94199400 | -0.83657500 |
| F | 7.38586800 | -1.80603300 | 0.77473800 |
| F | 5.93556100 | -3.08218900 | 1.73126500 |
| F | 6.54863000 | -3.48507300 | -0.28742700 |
| F | 4.88076000 | 2.54443300 | 0.28894800 |
| F | 3.36764100 | 2.57095000 | -1.24803100 |
| F | 5.41882000 | 2.14393300 | -1.75180500 |
| C | -4.99291300 | -2.36831800 | 1.94908700 |
| C | -4.81214300 | 0.40762000 | -2.22970900 |
| F | -4.21158400 | -2.31466800 | 3.03932800 |
| F | -5.35018200 | -3.64961500 | 1.79969000 |
| F | -6.09841200 | -1.67179200 | 2.21479600 |
| F | -5.33008500 | -0.30583200 | -3.24017600 |
| F | -3.95628700 | 1.27671000 | -2.76279300 |
| F | -5.81900700 | 1.10157100 | -1.68839200 |
| H | 0.47273900 | -4.72063300 | -2.89443400 |
| C | -0.02856900 | 1.42958000 | 1.45011400 |
| C | 1.18332100 | 2.36679500 | 1.27384600 |
| C | -1.30220200 | 2.29171500 | 1.51565000 |
| C | 1.28836200 | 3.40631900 | 2.38585000 |
| H | 1.08966600 | 2.88256900 | 0.30931400 |
| H | 2.10386000 | 1.76783400 | 1.22906900 |
| C | -1.20779800 | 3.28994600 | 2.66174600 |
| H | -1.27018300 | 2.87261400 | 0.58066600 |

| | | | |
|---|---|---|---|
| C | -0.00608200 | 4.20470800 | 2.47353300 |
| H | 1.42539100 | 2.88751300 | 3.34459900 |
| H | -2.13011500 | 3.87590700 | 2.71491100 |
| H | -1.12185000 | 2.75475700 | 3.61700600 |
| H | 0.06345600 | 4.93253200 | 3.28669300 |
| H | -0.12768100 | 4.77318200 | 1.54225500 |
| C | 2.48570400 | 4.31379300 | 2.14705200 |
| H | 3.42041200 | 3.75154100 | 2.14283400 |
| H | 2.39188900 | 4.81129900 | 1.17750600 |
| H | 2.55229000 | 5.08493800 | 2.91719500 |
| C | -2.63316100 | 1.50406600 | 1.47680900 |
| H | -2.44568500 | 0.54648800 | 0.96795600 |
| C | -3.62506900 | 2.29954400 | 0.63206000 |
| H | -3.72725700 | 3.31265700 | 1.03446100 |
| H | -3.27836500 | 2.38000500 | -0.40187900 |
| H | -4.61687100 | 1.84273600 | 0.63618100 |
| C | -3.27734600 | 1.19896600 | 2.82901400 |
| H | -3.54223300 | 2.12134100 | 3.35194400 |
| H | -4.20412900 | 0.64080700 | 2.66943400 |
| H | -2.65374900 | 0.60321200 | 3.49734000 |

52

g-1-1 carbene fragment

| | | | |
|---|---|---|---|
| C | 1.24935900 | -0.97343400 | 0.27660900 |
| N | -0.01947600 | 1.15743200 | 0.18104700 |
| C | -0.09671600 | 1.97118200 | 1.45992900 |
| C | 1.24678700 | -2.34778700 | 0.51250100 |
| H | 2.18282300 | -2.88552400 | 0.56108200 |
| C | -0.05106200 | 1.78511000 | -0.96962000 |
| C | -1.16965700 | -1.00537900 | 0.27647300 |
| C | 0.06492300 | -3.04426000 | 0.66489200 |
| C | 0.03162600 | -0.27160900 | 0.23402100 |
| C | -0.55805300 | 3.30456700 | 0.87165000 |
| H | -0.09352100 | 4.14850800 | 1.38702100 |
| H | -1.64261700 | 3.39292600 | 0.98555800 |

| | | | |
|---|---|---|---|
| C | -1.13504000 | -2.37858500 | 0.50500400 |
| H | -2.05670500 | -2.94128100 | 0.54784200 |
| C | 1.26789600 | 2.06618100 | 2.13591200 |
| H | 1.64772600 | 1.07377600 | 2.38877900 |
| H | 1.15283800 | 2.62253100 | 3.06793900 |
| H | 2.00153800 | 2.57918500 | 1.51999100 |
| C | -1.06916900 | 1.39181900 | 2.47806500 |
| H | -2.09052800 | 1.36109500 | 2.10820400 |
| H | -1.05744300 | 2.03117600 | 3.36259400 |
| H | -0.76870100 | 0.38961600 | 2.79456700 |
| C | 2.60565400 | -0.37565800 | -0.06917600 |
| C | -2.54744600 | -0.43634900 | -0.04555700 |
| C | -0.20603900 | 3.25245900 | -0.63026900 |
| C | 1.11889400 | 3.94299800 | -0.96488200 |
| H | 1.35044800 | 3.80954600 | -2.02257000 |
| H | 1.03927200 | 5.01302800 | -0.75552800 |
| H | 1.95193900 | 3.53884100 | -0.38823700 |
| C | -1.31374900 | 3.85454100 | -1.48912000 |
| H | -1.46031600 | 4.90734700 | -1.23088000 |
| H | -1.05271500 | 3.78370600 | -2.54580600 |
| H | -2.25722400 | 3.32654200 | -1.33435500 |
| H | 0.07830400 | -4.10693900 | 0.86286500 |
| C | 3.72754500 | -0.77085600 | 0.94916700 |
| C | 3.02189500 | -0.79602600 | -1.51218600 |
| C | -3.65085500 | -0.90608500 | 0.96589700 |
| C | -2.96256300 | -0.82886600 | -1.49673900 |
| F | 2.19203400 | -0.24786300 | -2.37868200 |
| F | 3.00146900 | -2.11434600 | -1.68568800 |
| F | 4.25428700 | -0.36368800 | -1.76527600 |
| F | 4.26464100 | -1.95741900 | 0.66505600 |
| F | 4.69489500 | 0.12939200 | 0.93403300 |
| F | 3.24140600 | -0.83211600 | 2.18721900 |
| F | -4.17262000 | -2.08345700 | 0.62099600 |
| F | -3.15442000 | -1.02861900 | 2.19419900 |

| | | | |
|---|---|---|---|
| F | -4.63243000 | -0.02139600 | 1.00874200 |
| F | -2.88556600 | -2.13864800 | -1.71031000 |
| F | -4.21625400 | -0.44322000 | -1.72135600 |
| F | -2.17030800 | -0.21783000 | -2.35610900 |
| F | -2.62424900 | 0.92232200 | -0.04816200 |
| F | 2.62554100 | 0.98023400 | -0.11042000 |

53

g-1-1 carbene-Au fragment

| | | | |
|---|---|---|---|
| Au | 0.31533800 | 2.32863600 | -0.89352100 |
| C | 1.20307000 | -1.40283000 | -0.44030700 |
| N | -0.07489600 | 0.11218900 | 1.07396100 |
| C | -0.10278100 | -0.19507800 | 2.55412900 |
| C | 1.19843600 | -2.46522600 | -1.34394000 |
| H | 2.13394300 | -2.89995000 | -1.66270500 |
| C | -0.36895300 | 1.36629900 | 0.75479600 |
| C | -1.21663100 | -1.39210400 | -0.49574200 |
| C | 0.01858300 | -2.97563500 | -1.84165700 |
| C | -0.01491200 | -0.88826100 | 0.04817400 |
| C | -0.88130600 | 1.02677200 | 3.05892400 |
| H | -0.54523700 | 1.32413500 | 4.05407200 |
| H | -1.94504000 | 0.78432300 | 3.11729300 |
| C | -1.17734100 | -2.41920800 | -1.43634200 |
| H | -2.09827300 | -2.80694900 | -1.84639600 |
| C | 1.28161000 | -0.24650700 | 3.19796000 |
| H | 1.85842400 | -1.10299700 | 2.85735600 |
| H | 1.13507200 | -0.35941600 | 4.27398000 |
| H | 1.85689300 | 0.65840600 | 3.02568300 |
| C | -0.79510800 | -1.51937200 | 2.83700700 |
| H | -1.83666300 | -1.51813400 | 2.52667400 |
| H | -0.76736900 | -1.70050600 | 3.91257600 |
| H | -0.27458800 | -2.34778100 | 2.34861800 |
| C | 2.58689800 | -0.91397600 | -0.04484400 |
| C | -2.62885500 | -0.92576600 | -0.15014000 |
| C | -0.66723400 | 2.13960800 | 2.01573700 |

| | | | |
|---|---|---|---|
| C | 0.50739300 | 3.06902400 | 2.34908100 |
| H | 0.56325400 | 3.86982200 | 1.61004900 |
| H | 0.35294900 | 3.50909200 | 3.33776900 |
| H | 1.46644400 | 2.55081100 | 2.33648500 |
| C | -1.91827500 | 2.99254700 | 1.81788800 |
| H | -2.14564500 | 3.54025500 | 2.73637200 |
| H | -1.75070000 | 3.71177700 | 1.01316600 |
| H | -2.77974600 | 2.37669400 | 1.55536600 |
| H | 0.02987700 | -3.79559700 | -2.54604500 |
| C | 3.37068300 | -2.03164100 | 0.72889900 |
| C | 3.45276200 | -0.42178500 | -1.25319800 |
| C | -3.56542400 | -2.13496300 | 0.21755500 |
| C | -3.28375000 | -0.10616900 | -1.30513700 |
| F | 2.90743300 | 0.65753700 | -1.77645300 |
| F | 3.60084000 | -1.31816600 | -2.22375500 |
| F | 4.66398300 | -0.11180400 | -0.79614200 |
| F | 4.03642100 | -2.82281700 | -0.10990200 |
| F | 4.23249700 | -1.49643900 | 1.57566700 |
| F | 2.53691900 | -2.80704400 | 1.42381200 |
| F | -4.13190900 | -2.66899900 | -0.86402700 |
| F | -2.88591300 | -3.09619900 | 0.83709200 |
| F | -4.52879800 | -1.73216100 | 1.02850700 |
| F | -3.19356100 | -0.72079500 | -2.47856500 |
| F | -4.57293400 | 0.08689000 | -1.03301300 |
| F | -2.69805500 | 1.07158100 | -1.40004900 |
| F | -2.70755100 | -0.09343600 | 0.92376900 |
| F | 2.55905100 | 0.17674700 | 0.76051100 |

46

h-1-1 carbene fragment

| | | | |
|---|---|---|---|
| C | -0.53653500 | -0.74222400 | 0.84863300 |
| N | 1.55936200 | -0.05072300 | -0.18618900 |
| C | 2.29184800 | 1.13805700 | 0.36715600 |
| C | -1.92022600 | -0.76753000 | 0.87718500 |
| H | -2.41776700 | -1.30691400 | 1.67526500 |

| | | | |
|---|---|---|---|
| C | 2.24956300 | -1.10937600 | -0.53562800 |
| C | -0.60954200 | 0.57547300 | -1.15230300 |
| C | -2.68326000 | -0.12342400 | -0.10193800 |
| C | 0.12478700 | -0.06386200 | -0.16813200 |
| C | 3.73474600 | 0.75196800 | 0.03359600 |
| H | 4.41289300 | 0.99544600 | 0.85522400 |
| H | 4.06662100 | 1.31038800 | -0.84657700 |
| C | -2.00030800 | 0.54294900 | -1.11612100 |
| H | -2.54786400 | 1.04267300 | -1.90411200 |
| C | 2.02246500 | 1.23972900 | 1.86461500 |
| H | 0.95913600 | 1.40293800 | 2.05462500 |
| H | 2.57645900 | 2.08264800 | 2.28212800 |
| H | 2.33170600 | 0.33050300 | 2.38248900 |
| C | 1.86742100 | 2.42974000 | -0.31527200 |
| H | 1.98096200 | 2.35152100 | -1.39837500 |
| H | 2.50329900 | 3.24421200 | 0.03780800 |
| H | 0.83104700 | 2.68359600 | -0.08415700 |
| C | -4.20474000 | -0.18415900 | -0.02791300 |
| C | 3.70363400 | -0.75621600 | -0.28612400 |
| C | 4.15934000 | -1.61117500 | 0.89957900 |
| H | 4.04798400 | -2.67033800 | 0.66344400 |
| H | 5.21032400 | -1.40693900 | 1.12224700 |
| H | 3.57265500 | -1.40383800 | 1.79727000 |
| C | 4.54817100 | -1.10426300 | -1.50613900 |
| H | 5.59789300 | -0.85453500 | -1.32600300 |
| H | 4.47280700 | -2.17043700 | -1.72475000 |
| H | 4.21038000 | -0.55226500 | -2.38602900 |
| C | -4.67624500 | 0.43359600 | 1.29262600 |
| H | -5.76690600 | 0.39058000 | 1.35421100 |
| H | -4.26839600 | -0.10111200 | 2.15236500 |
| H | -4.36729100 | 1.47878800 | 1.36368900 |
| C | -4.66089000 | -1.64581900 | -0.08699000 |
| H | -4.25574700 | -2.22546400 | 0.74434900 |
| H | -5.75173800 | -1.69633800 | -0.03451700 |

| | | | |
|---|---|---|---|
| H | -4.33697600 | -2.11541400 | -1.01807400 |
| C | -4.87208400 | 0.57094700 | -1.17342100 |
| H | -5.95671400 | 0.49810100 | -1.06728500 |
| H | -4.60430300 | 1.63017100 | -1.16669500 |
| H | -4.60107900 | 0.14981100 | -2.14428000 |
| H | 0.05044300 | -1.25994700 | 1.59834800 |
| H | -0.09423900 | 1.08076500 | -1.95973700 |

47

h-1-1 carbene-Au fragment

| | | | |
|---|---|---|---|
| Au | 1.41035600 | -1.69252000 | -0.00669100 |
| C | -1.21618000 | 0.32779600 | 1.01628200 |
| N | 0.73254900 | 1.21662300 | -0.14775900 |
| C | 1.19424500 | 2.59573300 | 0.19748500 |
| C | -2.56070700 | 0.00117500 | 1.03700700 |
| H | -2.97609200 | -0.44366500 | 1.93367200 |
| C | 1.67456000 | 0.31653200 | -0.31890100 |
| C | -1.45164100 | 1.10118500 | -1.24617900 |
| C | -3.38249700 | 0.21457200 | -0.07445400 |
| C | -0.66253100 | 0.88532100 | -0.13070000 |
| C | 2.68470700 | 2.49028800 | -0.14503700 |
| H | 3.29856300 | 3.03806000 | 0.57277100 |
| H | 2.85753600 | 2.92445400 | -1.13379500 |
| C | -2.80199500 | 0.76744200 | -1.21328000 |
| H | -3.39700800 | 0.93752700 | -2.10039900 |
| C | 0.92667000 | 2.86027000 | 1.67496300 |
| H | -0.14568200 | 2.83411400 | 1.88017300 |
| H | 1.30161000 | 3.84944500 | 1.94369400 |
| H | 1.41858200 | 2.11822100 | 2.30516200 |
| C | 0.48155300 | 3.63806700 | -0.64913900 |
| H | 0.60255000 | 3.42347100 | -1.71263300 |
| H | 0.91602200 | 4.61832500 | -0.44454600 |
| H | -0.58379300 | 3.68024300 | -0.41527500 |
| C | -4.85407200 | -0.17582400 | -0.00218100 |
| C | 3.02102400 | 0.98711600 | -0.17420100 |

| | | | |
|---|---|---|---|
| C | 3.67846200 | 0.51121900 | 1.12546600 |
| H | 3.87208300 | -0.56150300 | 1.07868700 |
| H | 4.62500400 | 1.04057900 | 1.26334000 |
| H | 3.04577600 | 0.69553800 | 1.99542100 |
| C | 3.91772100 | 0.60950000 | -1.34908000 |
| H | 4.88824500 | 1.10345700 | -1.25464100 |
| H | 4.07167300 | -0.47139700 | -1.36477200 |
| H | 3.46806000 | 0.90619900 | -2.29876800 |
| C | -5.53084800 | 0.59333500 | 1.13718500 |
| H | -6.58598800 | 0.31405000 | 1.19637800 |
| H | -5.06905200 | 0.37061900 | 2.10071500 |
| H | -5.46802100 | 1.67082500 | 0.96955700 |
| C | -4.96659700 | -1.67936400 | 0.27190100 |
| H | -4.49545700 | -1.95083500 | 1.21812200 |
| H | -6.01975700 | -1.96784700 | 0.32313900 |
| H | -4.48769200 | -2.25541800 | -0.52233000 |
| C | -5.60056000 | 0.13211200 | -1.29659200 |
| H | -6.64655400 | -0.16146100 | -1.18518900 |
| H | -5.57457100 | 1.19870800 | -1.53239900 |
| H | -5.18560100 | -0.42190900 | -2.14164700 |
| H | -0.57705700 | 0.13148800 | 1.86933300 |
| H | -1.00724300 | 1.51257300 | -2.14413800 |

46

i-1-1 carbene fragment

| | | | |
|---|---|---|---|
| C | 1.12453300 | -0.74575700 | 0.78039700 |
| N | 3.22460100 | -0.00877700 | -0.19811700 |
| C | 3.99524500 | 1.15773400 | 0.35440300 |
| C | -0.25858500 | -0.74360700 | 0.82975600 |
| H | -0.74433000 | -1.36157200 | 1.56818400 |
| C | 3.87801500 | -1.09813100 | -0.52776200 |
| C | 1.06279700 | 0.74141600 | -1.09267700 |
| C | -1.01045100 | 0.02484100 | -0.06575300 |
| C | 1.79363200 | 0.00958800 | -0.17157900 |
| C | 5.42377500 | 0.71737000 | 0.02577800 |

| | | | |
|---|---|---|---|
| H | 6.10758700 | 0.94271400 | 0.84761500 |
| H | 5.77655700 | 1.25829400 | -0.85701200 |
| C | -0.32476400 | 0.74256100 | -1.04555300 |
| H | -0.84566400 | 1.30332200 | -1.80405000 |
| C | 3.72010000 | 1.26458300 | 1.85039000 |
| H | 2.66155900 | 1.46298800 | 2.03513100 |
| H | 4.29810300 | 2.08772200 | 2.27412900 |
| H | 3.99557900 | 0.34445900 | 2.36780100 |
| C | 3.62597000 | 2.46648800 | -0.32727500 |
| H | 3.73162800 | 2.38481400 | -1.41088800 |
| H | 4.30172100 | 3.24928900 | 0.02273200 |
| H | 2.60557400 | 2.77278700 | -0.08870000 |
| C | -2.54313400 | -0.04542600 | -0.01155500 |
| C | 5.34195200 | -0.79126600 | -0.28397500 |
| C | 5.76988300 | -1.65389300 | 0.90710900 |
| H | 5.62668800 | -2.71030400 | 0.67665100 |
| H | 6.82643900 | -1.48026200 | 1.12830900 |
| H | 5.19075700 | -1.42377800 | 1.80414300 |
| C | 6.17253900 | -1.17596500 | -1.50280600 |
| H | 7.22986800 | -0.96072100 | -1.32412100 |
| H | 6.06139600 | -2.24031200 | -1.71417900 |
| H | 5.85362200 | -0.61845500 | -2.38612200 |
| C | -3.11240600 | 0.39358500 | 1.37389300 |
| C | -2.97438200 | -1.52136100 | -0.28774000 |
| C | -3.22519000 | 0.85284200 | -1.08391500 |
| H | 1.70248400 | -1.35236200 | 1.46659400 |
| H | 1.57443800 | 1.29143700 | -1.87125100 |
| F | -2.22723600 | -2.05382900 | -1.24558600 |
| F | -4.24374500 | -1.62493400 | -0.66219800 |
| F | -2.81911800 | -2.26080400 | 0.81209500 |
| F | -4.36719400 | -0.01897100 | 1.52991500 |
| F | -2.41462200 | -0.08717700 | 2.39621300 |
| F | -3.09540700 | 1.71958400 | 1.47604300 |
| F | -4.53694800 | 0.93835100 | -0.89955400 |

| | | | |
|---|---|---|---|
| F | -2.75735000 | 2.09857100 | -1.07075800 |
| F | -3.01430900 | 0.34895800 | -2.30005500 |

47

i-1-1 carbene-Au fragment

| | | | |
|---|---|---|---|
| Au | -2.70821500 | -1.68809400 | 0.14362800 |
| C | -0.14422500 | 0.58471200 | -1.08956100 |
| N | -2.15974400 | 1.20734600 | 0.11043600 |
| C | -2.69806500 | 2.59010000 | 0.27351900 |
| C | 1.21205200 | 0.31510700 | -1.12226100 |
| H | 1.65132600 | 0.02043300 | -2.06188900 |
| C | -3.03015400 | 0.27962000 | -0.25197100 |
| C | -0.01013100 | 0.98497200 | 1.26890200 |
| C | 1.99326900 | 0.40615300 | 0.03566000 |
| C | -0.75744500 | 0.94328900 | 0.10327900 |
| C | -4.20695300 | 2.31747600 | 0.23349300 |
| H | -4.73374200 | 3.09050000 | -0.33015800 |
| H | -4.59983300 | 2.32084500 | 1.25376000 |
| C | 1.35151600 | 0.71501100 | 1.23427400 |
| H | 1.88231900 | 0.73075700 | 2.17177800 |
| C | -2.20392300 | 3.44910800 | -0.88564000 |
| H | -1.11384700 | 3.51961800 | -0.87175900 |
| H | -2.61207300 | 4.45707800 | -0.79575100 |
| H | -2.51107400 | 3.03370900 | -1.84628700 |
| C | -2.27490100 | 3.22016400 | 1.59223600 |
| H | -2.52361600 | 2.56899100 | 2.43224800 |
| H | -2.81203900 | 4.16220600 | 1.71844900 |
| H | -1.20577500 | 3.43877800 | 1.60885400 |
| C | 3.48420900 | 0.04755100 | -0.03600200 |
| C | -4.39284000 | 0.91559900 | -0.37834400 |
| C | -4.72064700 | 0.96463200 | -1.87531200 |
| H | -4.76441500 | -0.04840500 | -2.27878600 |
| H | -5.69114800 | 1.44529200 | -2.02243100 |
| H | -3.97086700 | 1.52245500 | -2.43949700 |
| C | -5.47497300 | 0.11882500 | 0.34087100 |

| | | | |
|---|---|---|---|
| H | -6.43096500 | 0.64329200 | 0.26008800 |
| H | -5.57744300 | -0.87307000 | -0.10233500 |
| H | -5.23136300 | -0.00941200 | 1.39709500 |
| C | 4.26500500 | 0.94560600 | -1.04612300 |
| C | 3.60622900 | -1.44342900 | -0.48725000 |
| C | 4.20258000 | 0.18512600 | 1.33794000 |
| H | -0.74774900 | 0.50105600 | -1.98525700 |
| H | -0.49290600 | 1.19129400 | 2.21428200 |
| F | 2.67910800 | -2.18495700 | 0.10189900 |
| F | 4.78994100 | -1.97045700 | -0.19961200 |
| F | 3.43316300 | -1.54028800 | -1.80696400 |
| F | 5.43363800 | 0.40058100 | -1.37012900 |
| F | 3.60193100 | 1.14735800 | -2.17899700 |
| F | 4.49765000 | 2.14054100 | -0.51014700 |
| F | 5.51966100 | 0.06863700 | 1.22361600 |
| F | 3.97292600 | 1.36453100 | 1.91080400 |
| F | 3.77739900 | -0.76429200 | 2.17082600 |

43

j-1-1 carbene fragment

| | | | |
|---|---|---|---|
| C | 1.41195600 | 1.22357600 | -0.15330800 |
| N | -0.66962700 | -0.07633300 | -0.09948700 |
| C | -1.36455100 | -0.21093500 | 1.22912400 |
| C | 2.80303700 | 1.24865800 | -0.18405500 |
| H | 3.30445000 | 2.21223000 | -0.18686900 |
| C | -1.40202200 | 0.05977900 | -1.17714400 |
| C | 1.50076400 | -1.20358500 | -0.30205100 |
| C | 3.56296100 | 0.08690100 | -0.24530400 |
| C | 0.76815500 | -0.02012300 | -0.16372300 |
| C | -2.81256200 | -0.39648000 | 0.76792400 |
| H | -3.50648200 | 0.17289800 | 1.39126600 |
| H | -3.08822000 | -1.45227900 | 0.84509400 |
| C | 2.89032300 | -1.12516300 | -0.32945600 |
| H | 3.45985900 | -2.04282300 | -0.44632500 |
| C | -1.15875500 | 1.04921800 | 2.06115400 |

| | | | |
|---|---|---|---|
| H | -0.09789600 | 1.19858800 | 2.27646700 |
| H | -1.68643100 | 0.94269800 | 3.01080200 |
| H | -1.54279200 | 1.93251700 | 1.55045300 |
| C | -0.84947700 | -1.40734500 | 2.01480800 |
| H | -1.02978700 | -2.33734000 | 1.47625800 |
| H | -1.37836600 | -1.45871800 | 2.96866100 |
| H | 0.21940400 | -1.31506500 | 2.22292200 |
| C | 0.66395000 | 2.52830900 | -0.18263900 |
| H | 0.53746500 | 2.94761400 | 0.81739500 |
| H | 1.22350700 | 3.25479800 | -0.77219300 |
| H | -0.32201300 | 2.41374100 | -0.63305600 |
| C | 0.85083500 | -2.54674200 | -0.49029400 |
| H | 1.39272300 | -3.11053600 | -1.25027200 |
| H | 0.87331200 | -3.13467100 | 0.42968700 |
| H | -0.18550100 | -2.45338100 | -0.81275800 |
| C | 5.06417700 | 0.14238000 | -0.24115800 |
| H | 5.45097600 | 0.11068500 | 0.78014800 |
| H | 5.49158700 | -0.70332400 | -0.78053900 |
| H | 5.42609800 | 1.06278700 | -0.70031100 |
| C | -2.84438500 | 0.04313700 | -0.70924600 |
| C | -3.37074200 | 1.47099600 | -0.87765600 |
| H | -3.28378500 | 1.78445300 | -1.91914500 |
| H | -4.42223500 | 1.51767300 | -0.58118900 |
| H | -2.81137200 | 2.18122900 | -0.26360700 |
| C | -3.66682200 | -0.90330900 | -1.57498200 |
| H | -4.70697700 | -0.92385500 | -1.23690600 |
| H | -3.64102000 | -0.58075700 | -2.61687700 |
| H | -3.27016900 | -1.92046800 | -1.52465200 |

44

j-1-1 carbene-Au fragment

| | | | |
|---|---|---|---|
| Au | 1.07339100 | -1.65461000 | -0.07606900 |
| C | -1.84951100 | 0.07367100 | 1.17739500 |
| N | 0.00399900 | 1.13326000 | -0.02772600 |
| C | 0.26551700 | 2.58396400 | 0.24022100 |

| | | | |
|---|---|---|---|
| C | -3.15120000 | -0.41790800 | 1.16422100 |
| H | -3.55731700 | -0.83122000 | 2.08260300 |
| C | 1.05932500 | 0.38937200 | -0.25588600 |
| C | -2.05146800 | 0.52356100 | -1.21106900 |
| C | -3.92492900 | -0.43312300 | 0.01061300 |
| C | -1.32886000 | 0.58891000 | -0.01532200 |
| C | 1.75574700 | 2.68026100 | -0.11378300 |
| H | 2.29260100 | 3.31902400 | 0.59059000 |
| H | 1.86330900 | 3.11753100 | -1.11034000 |
| C | -3.34711000 | 0.01927500 | -1.16934500 |
| H | -3.90959900 | -0.04457800 | -2.09613500 |
| C | -0.03266400 | 2.92515600 | 1.69467600 |
| H | -1.08585800 | 2.74458500 | 1.92074500 |
| H | 0.17374600 | 3.98410500 | 1.85964700 |
| H | 0.58024500 | 2.34316400 | 2.38226900 |
| C | -0.59335400 | 3.46117800 | -0.65810400 |
| H | -0.37362400 | 3.27853000 | -1.70953100 |
| H | -0.37537900 | 4.50825100 | -0.44021200 |
| H | -1.65703800 | 3.28787800 | -0.47920600 |
| C | -1.04827000 | -0.04046700 | 2.44541700 |
| H | -1.31827700 | 0.73604100 | 3.16478300 |
| H | -1.24659500 | -1.00657500 | 2.90952800 |
| H | 0.02382000 | 0.01540900 | 2.25948400 |
| C | -1.46923300 | 0.91597900 | -2.54089600 |
| H | -1.78022000 | 0.19848500 | -3.30033200 |
| H | -1.82327400 | 1.89981800 | -2.85652000 |
| H | -0.37974300 | 0.93278200 | -2.52291900 |
| C | -5.33697100 | -0.94416600 | 0.03397500 |
| H | -6.03728700 | -0.13601500 | 0.25758800 |
| H | -5.61916700 | -1.36673500 | -0.93049600 |
| H | -5.46535700 | -1.71117700 | 0.79792300 |
| C | 2.29878200 | 1.23956000 | -0.12257100 |
| C | 2.98696800 | 0.87053100 | 1.19612600 |
| H | 3.30380700 | -0.17352900 | 1.17724200 |

| | | | |
|---|---|---|---|
| H | 3.86273000 | 1.50904300 | 1.33804100 |
| H | 2.31964800 | 1.00188300 | 2.05105300 |
| C | 3.25233000 | 0.97403300 | -1.28136800 |
| H | 4.14198800 | 1.60239900 | -1.19051000 |
| H | 3.55742100 | -0.07445400 | -1.27810300 |
| H | 2.77296000 | 1.18802100 | -2.23948800 |

49

j-2-1 carbene fragment

| | | | |
|---|---|---|---|
| C | 1.93009000 | 1.16812800 | -0.12037300 |
| N | -0.24663300 | 0.03847700 | 0.04623100 |
| C | -0.85294300 | -0.01846600 | 1.42215600 |
| C | 3.31431400 | 1.08182300 | -0.24574000 |
| H | 3.89201600 | 2.00172400 | -0.25811200 |
| C | -1.04614200 | 0.06193100 | -0.99053600 |
| C | 1.80669900 | -1.25222300 | -0.33140700 |
| C | 3.96865100 | -0.13419300 | -0.38761600 |
| C | 1.18490200 | -0.01517400 | -0.11570900 |
| C | -2.34185900 | 0.13547100 | 1.09241500 |
| H | -2.70064700 | 1.11233100 | 1.42590200 |
| H | -2.93303100 | -0.61999700 | 1.61633600 |
| C | 3.19213700 | -1.28498000 | -0.45373200 |
| H | 3.67439400 | -2.24187800 | -0.63186900 |
| C | -0.33837900 | 1.11148300 | 2.30083800 |
| H | 0.74532300 | 1.05352500 | 2.42920000 |
| H | -0.80309400 | 1.03452000 | 3.28583000 |
| H | -0.59660100 | 2.08273300 | 1.87657100 |
| C | -0.53539100 | -1.35346100 | 2.08382200 |
| H | -0.94096500 | -2.18406500 | 1.50453500 |
| H | -0.98383600 | -1.37660400 | 3.07883600 |
| H | 0.54357900 | -1.48863500 | 2.19229900 |
| C | 1.29863700 | 2.53195100 | -0.06599000 |
| H | 1.39470700 | 2.97887900 | 0.92585500 |
| H | 1.80105400 | 3.19425000 | -0.77180400 |
| H | 0.24155900 | 2.49975900 | -0.32452600 |

| | | | |
|---|---|---|---|
| C | 1.03720100 | -2.53334500 | -0.50159900 |
| H | 1.54493600 | -3.16869700 | -1.22756800 |
| H | 0.97200100 | -3.09372200 | 0.43316800 |
| H | 0.02509000 | -2.34907200 | -0.86162600 |
| C | 5.46596700 | -0.20422300 | -0.48686300 |
| H | 5.91255200 | -0.37677100 | 0.49519400 |
| H | 5.78044600 | -1.02099900 | -1.13736200 |
| H | 5.87909900 | 0.72610100 | -0.87734900 |
| C | -2.45814500 | -0.00365100 | -0.44032800 |
| C | -3.31297800 | 1.10210900 | -1.06871900 |
| C | -2.99099200 | -1.39274800 | -0.83997600 |
| H | -2.80238600 | -1.53134100 | -1.90853600 |
| H | -4.28126400 | 1.13231600 | -0.55799100 |
| C | -2.64739300 | 2.47059500 | -1.01478700 |
| H | -2.33918700 | 2.72651900 | 0.00436700 |
| H | -1.75557300 | 2.47887300 | -1.64420900 |
| H | -3.31819700 | 3.25732500 | -1.36201500 |
| H | -3.50811800 | 0.83309300 | -2.11210000 |
| C | -4.46869500 | -1.60369600 | -0.53800400 |
| H | -5.09481900 | -0.96579000 | -1.16332000 |
| H | -4.75934100 | -2.63776200 | -0.72689100 |
| H | -4.70310800 | -1.37829200 | 0.50555600 |
| H | -2.39426000 | -2.15166300 | -0.31909500 |

50

j-2-1 carbene-Au fragment

| | | | |
|---|---|---|---|
| Au | 0.53983400 | -1.85678400 | -0.07288900 |
| C | -2.17681300 | 0.48069900 | 1.18043200 |
| N | -0.06724400 | 1.08160000 | 0.08199200 |
| C | 0.39153000 | 2.50607800 | 0.19244300 |
| C | -3.53081600 | 0.17651900 | 1.07597900 |
| H | -4.08522500 | -0.03670200 | 1.98494000 |
| C | 0.86902300 | 0.18205000 | -0.01920700 |
| C | -2.06322500 | 0.58671200 | -1.25334800 |
| C | -4.17682100 | 0.09585200 | -0.15035800 |

| | | | |
|---|---|---|---|
| C | -1.46690500 | 0.73826600 | 0.00448000 |
| C | 1.91559100 | 2.33253000 | 0.28903600 |
| H | 2.26016300 | 2.59971400 | 1.29019200 |
| H | 2.42095200 | 2.99652700 | -0.41578900 |
| C | -3.41819100 | 0.27812000 | -1.30082600 |
| H | -3.88532800 | 0.14539200 | -2.27196900 |
| C | -0.19548500 | 3.16724100 | 1.42885500 |
| H | -1.28737000 | 3.17287400 | 1.39235900 |
| H | 0.15144400 | 4.20104800 | 1.47454700 |
| H | 0.12919000 | 2.65554900 | 2.33570500 |
| C | -0.02759800 | 3.27881200 | -1.05054200 |
| H | 0.42737600 | 2.85386100 | -1.94637700 |
| H | 0.30302500 | 4.31424200 | -0.95373100 |
| H | -1.11399700 | 3.27387800 | -1.16530000 |
| C | -1.52838700 | 0.43782700 | 2.53604800 |
| H | -1.72907400 | 1.34644300 | 3.10807000 |
| H | -1.93023500 | -0.40291800 | 3.10146000 |
| H | -0.44928200 | 0.30644200 | 2.46810600 |
| C | -1.29328100 | 0.66883300 | -2.54255200 |
| H | -1.71761000 | -0.03213000 | -3.26082700 |
| H | -1.34607600 | 1.66562200 | -2.98512600 |
| H | -0.24315300 | 0.40769100 | -2.40693200 |
| C | -5.64498800 | -0.20818300 | -0.23451500 |
| H | -6.22913600 | 0.71392000 | -0.27964800 |
| H | -5.87635400 | -0.78744200 | -1.12867200 |
| H | -5.98141500 | -0.76957900 | 0.63712400 |
| C | 2.21619000 | 0.85078600 | -0.01780200 |
| C | 3.13729500 | 0.19875500 | 1.02058500 |
| C | 2.77839700 | 0.65873500 | -1.44107300 |
| H | 2.71549000 | -0.40671100 | -1.68495700 |
| H | 4.05028500 | 0.79852100 | 1.08575100 |
| C | 2.50023000 | 0.05489900 | 2.39616900 |
| H | 2.06267600 | 0.99757000 | 2.74151500 |
| H | 1.70824900 | -0.69777000 | 2.37063800 |

| | | | |
|---|---|---|---|
| H | 3.23361200 | -0.25738100 | 3.13968100 |
| H | 3.42113400 | -0.79106500 | 0.64659400 |
| C | 4.20793400 | 1.15350700 | -1.60980900 |
| H | 4.90852700 | 0.53784300 | -1.04508200 |
| H | 4.50539100 | 1.11427700 | -2.65780900 |
| H | 4.32160500 | 2.18638600 | -1.27053700 |
| H | 2.11684600 | 1.18042700 | -2.14360700 |

58

j-3-1 carbene fragment

| | | | |
|---|---|---|---|
| C | -2.61893300 | -1.07538800 | 0.66472000 |
| N | -0.72950700 | 0.38051500 | 0.09658000 |
| C | 2.48442800 | 0.65169100 | -1.18725400 |
| H | 1.99464100 | 1.39516400 | -1.82920500 |
| C | 3.45658300 | -1.64939500 | -1.10688100 |
| H | 3.65643600 | -2.55500000 | -1.68689900 |
| C | -0.36015000 | 1.46299200 | 1.06881600 |
| C | -3.95815200 | -1.41398900 | 0.49789600 |
| H | -4.35699900 | -2.24912000 | 1.06652000 |
| C | 2.23589500 | -0.72402800 | 0.87381800 |
| H | 1.57505400 | -0.97553900 | 1.71274000 |
| C | 0.23211100 | -0.25854200 | -0.51965700 |
| C | 2.78253100 | -0.60882700 | -1.99995200 |
| H | 3.44855700 | -0.35041300 | -2.83159100 |
| H | 1.85565000 | -1.01065500 | -2.41220800 |
| C | -2.88988400 | 0.63888100 | -1.04142100 |
| C | -4.78588500 | -0.74212000 | -0.39396000 |
| C | 2.53223000 | -1.99037000 | 0.06299500 |
| H | 3.01918700 | -2.72414500 | 0.71596800 |
| H | 1.60286900 | -2.42568900 | -0.31004600 |
| C | 4.77020800 | -1.07915700 | -0.57398800 |
| H | 5.27612300 | -1.82096400 | 0.05432500 |
| H | 5.43886500 | -0.83936400 | -1.40837600 |
| C | -2.10514300 | -0.00633600 | -0.08079600 |
| C | 1.53466800 | 0.33672700 | -0.01502800 |

| | | | |
|---|---|---|---|
| C | 3.79550500 | 1.22140400 | -0.64232400 |
| H | 4.45141500 | 1.48962200 | -1.47767800 |
| H | 3.60967500 | 2.13580300 | -0.06764700 |
| C | 1.12949400 | 1.61025400 | 0.75789300 |
| H | 1.70254900 | 1.77645900 | 1.67146100 |
| H | 1.28250500 | 2.48399500 | 0.11489100 |
| C | -4.22411300 | 0.26244600 | -1.17064500 |
| H | -4.83234300 | 0.75653100 | -1.92302400 |
| C | 3.56241800 | -0.17911500 | 1.40947700 |
| H | 3.40852800 | 0.69515000 | 2.04819900 |
| H | 4.03537500 | -0.94872300 | 2.02986800 |
| C | -0.62797900 | 1.00222800 | 2.49681300 |
| H | -1.69180000 | 0.79783400 | 2.64117000 |
| H | -0.33663500 | 1.79255600 | 3.19122800 |
| H | -0.05942300 | 0.10361400 | 2.73765800 |
| C | 4.48269100 | 0.18001100 | 0.24265900 |
| H | 5.41967200 | 0.59068900 | 0.63066300 |
| C | -1.13682100 | 2.74540200 | 0.81240200 |
| H | -0.92561300 | 3.14081600 | -0.18070100 |
| H | -0.83600500 | 3.49441100 | 1.54770600 |
| H | -2.21307200 | 2.58376200 | 0.91241800 |
| C | -6.23718000 | -1.10636700 | -0.52773400 |
| H | -6.84621100 | -0.54214800 | 0.18246200 |
| H | -6.60879700 | -0.88417600 | -1.52840100 |
| H | -6.39799300 | -2.16615700 | -0.32766600 |
| C | -2.33632400 | 1.66958300 | -1.98647700 |
| H | -2.73870100 | 1.50114300 | -2.98591100 |
| H | -2.61961600 | 2.68082900 | -1.68714400 |
| H | -1.24951100 | 1.62159700 | -2.04616300 |
| C | -1.76761900 | -1.91326300 | 1.57932500 |
| H | -1.80630200 | -1.55927100 | 2.61119800 |
| H | -2.13064000 | -2.94114800 | 1.57355000 |
| H | -0.72437100 | -1.91941500 | 1.26039400 |

59

j-3-1 carbene-Au fragment

| | | | |
|---|---|---|---|
| Au | -0.27385000 | -1.85195400 | -0.20900700 |
| C | -2.41036400 | 0.83762300 | 1.37636900 |
| N | -0.64459200 | 1.03423200 | -0.29741400 |
| C | 2.68883100 | -0.06223000 | -1.08180400 |
| H | 2.27445800 | -0.30099000 | -2.06853900 |
| C | 3.62320800 | -1.03664900 | 1.02370000 |
| H | 3.85717500 | -1.96745600 | 1.54728600 |
| C | -0.17725700 | 2.37125400 | -0.77463400 |
| C | -3.74840300 | 0.63658100 | 1.68678200 |
| H | -4.05391800 | 0.67726100 | 2.72779000 |
| C | 2.23326100 | 1.05192100 | 1.09971700 |
| H | 1.49205500 | 1.59774500 | 1.69269800 |
| C | 0.28942700 | 0.12461700 | -0.10628700 |
| C | 3.07203300 | -1.35476600 | -0.36221300 |
| H | 3.83441600 | -1.86991500 | -0.95755600 |
| H | 2.21217600 | -2.02667800 | -0.28419500 |
| C | -2.92865000 | 0.40286500 | -0.96480200 |
| C | -4.69687700 | 0.35039800 | 0.70794700 |
| C | 2.56474900 | -0.26468700 | 1.81136700 |
| H | 2.93819600 | -0.03589700 | 2.81564700 |
| H | 1.66462400 | -0.87818000 | 1.91889700 |
| C | 4.88745500 | -0.19214000 | 0.87646600 |
| H | 5.30966900 | 0.03774900 | 1.86118800 |
| H | 5.64267300 | -0.75140000 | 0.31364100 |
| C | -2.02401500 | 0.78013200 | 0.02940000 |
| C | 1.64452100 | 0.77146600 | -0.31369600 |
| C | 3.95921200 | 0.78507500 | -1.23254600 |
| H | 4.69343000 | 0.22204600 | -1.81796600 |
| H | 3.75365900 | 1.71044100 | -1.78014500 |
| C | 1.28072600 | 2.05089500 | -1.09795100 |
| H | 1.92333700 | 2.90431100 | -0.88516400 |
| H | 1.37343300 | 1.82938800 | -2.16699500 |
| C | -4.26073200 | 0.21078900 | -0.60089700 |

| | | | |
|---|---|---|---|
| H | -4.96768700 | -0.08975200 | -1.36850100 |
| C | 3.51854500 | 1.87351800 | 0.98307900 |
| H | 3.32916800 | 2.86040200 | 0.55277600 |
| H | 3.91886400 | 2.03993700 | 1.98931200 |
| C | -0.33350900 | 3.43013400 | 0.31303200 |
| H | -1.36787300 | 3.46922200 | 0.66363400 |
| H | -0.07964100 | 4.40605800 | -0.10422600 |
| H | 0.32531000 | 3.24556100 | 1.16053100 |
| C | 4.54325700 | 1.10588200 | 0.14729100 |
| H | 5.44501200 | 1.71324200 | 0.02720500 |
| C | -0.94751400 | 2.83155300 | -2.00352700 |
| H | -0.82907900 | 2.13311500 | -2.83014300 |
| H | -0.55537700 | 3.80088200 | -2.31693800 |
| H | -2.01080500 | 2.95001600 | -1.78170000 |
| C | -6.14345500 | 0.17051100 | 1.06905000 |
| H | -6.64808800 | 1.13685100 | 1.14039400 |
| H | -6.66615200 | -0.42124500 | 0.31764000 |
| H | -6.24971500 | -0.32577200 | 2.03427200 |
| C | -2.51698200 | 0.10017200 | -2.37850700 |
| H | -3.00511900 | -0.81937500 | -2.70227600 |
| H | -2.81402500 | 0.89487700 | -3.06647400 |
| H | -1.44158300 | -0.05444100 | -2.46240300 |
| C | -1.40467100 | 1.00327600 | 2.47961900 |
| H | -0.87997400 | 1.95706100 | 2.43158800 |
| H | -1.89881600 | 0.93793800 | 3.44818300 |
| H | -0.65029400 | 0.21259000 | 2.42229100 |

50

j-4-1 carbene fragment

| | | | |
|---|---|---|---|
| C | 2.11722500 | 1.24256000 | -0.18385500 |
| N | 0.10041300 | -0.13534000 | 0.05383400 |
| C | -0.46317000 | -0.30320700 | 1.43673600 |
| C | 3.49734100 | 1.32091300 | -0.34466500 |
| H | 3.95969400 | 2.30295200 | -0.38639000 |
| C | -0.73319900 | -0.01816100 | -0.95018000 |

| | | | |
|---|---|---|---|
| C | 2.28295900 | -1.17874100 | -0.36071900 |
| C | 4.29133000 | 0.18939900 | -0.48667900 |
| C | 1.52266200 | -0.02483700 | -0.14385500 |
| C | -1.94461100 | -0.52356200 | 1.11399200 |
| H | -2.58145900 | 0.03126600 | 1.80705100 |
| H | -2.19296400 | -1.58418400 | 1.22378400 |
| C | 3.65985500 | -1.04708800 | -0.51775900 |
| H | 4.24992500 | -1.94171900 | -0.69548500 |
| C | -0.21934900 | 0.95546700 | 2.26058000 |
| H | 0.85155400 | 1.14038200 | 2.37484900 |
| H | -0.64974900 | 0.82321000 | 3.25503300 |
| H | -0.68154500 | 1.82795000 | 1.79762000 |
| C | 0.15558800 | -1.49159900 | 2.15690300 |
| H | -0.04787200 | -2.42107600 | 1.62568400 |
| H | -0.28055900 | -1.56750200 | 3.15499500 |
| H | 1.23624200 | -1.37030000 | 2.26583200 |
| C | 1.32142300 | 2.51802200 | -0.13216500 |
| H | 1.27615500 | 2.92868800 | 0.87839900 |
| H | 1.79433000 | 3.26720600 | -0.76745300 |
| H | 0.30151100 | 2.36794100 | -0.48652400 |
| C | 1.66867800 | -2.54460900 | -0.49907200 |
| H | 2.16041700 | -3.08507300 | -1.30848300 |
| H | 1.79509400 | -3.13467300 | 0.41108800 |
| H | 0.60472500 | -2.48855900 | -0.72651300 |
| C | 5.78315900 | 0.30210100 | -0.62283100 |
| H | 6.26558600 | 0.27531200 | 0.35708200 |
| H | 6.18882800 | -0.52147800 | -1.21107300 |
| H | 6.06569100 | 1.24000700 | -1.10185600 |
| C | -2.12425600 | -0.09342300 | -0.35610600 |
| C | -2.73718600 | 1.31115100 | -0.49413800 |
| C | -2.97710700 | -1.08235500 | -1.15357600 |
| C | -4.20366900 | 1.34701600 | -0.07814500 |
| H | -2.64477700 | 1.61111900 | -1.54395500 |
| H | -2.15262300 | 2.02890000 | 0.09208900 |

| | | | |
|---|---|---|---|
| C | -4.42994400 | -1.06729100 | -0.69614100 |
| H | -2.91827600 | -0.80291700 | -2.21119200 |
| H | -2.54485400 | -2.08475900 | -1.06323400 |
| C | -5.01802500 | 0.32831800 | -0.86642200 |
| H | -4.60341500 | 2.35380900 | -0.22091900 |
| H | -4.29458400 | 1.12414200 | 0.99149800 |
| H | -5.01148700 | -1.80208100 | -1.25755600 |
| H | -4.48843700 | -1.35759900 | 0.36092700 |
| H | -6.06462400 | 0.35034500 | -0.55377100 |
| H | -4.99365600 | 0.59563200 | -1.92978200 |

51

j-4-1 carbene-Au fragment

| | | | |
|---|---|---|---|
| Au | 0.25605300 | -1.88837100 | -0.08720400 |
| C | -2.25493500 | 0.37332000 | 1.19304800 |
| N | -0.25321200 | 1.05493000 | -0.04556800 |
| C | 0.27505800 | 2.43513100 | 0.19230000 |
| C | -3.62491900 | 0.13025800 | 1.20048000 |
| H | -4.08885200 | -0.18770100 | 2.12936700 |
| C | 0.64370800 | 0.12394000 | -0.25584800 |
| C | -2.39984800 | 0.81630400 | -1.20106900 |
| C | -4.40232200 | 0.24075400 | 0.05451500 |
| C | -1.66359200 | 0.76668400 | -0.01306800 |
| C | 1.75994100 | 2.24575400 | -0.14850700 |
| H | 2.39341400 | 2.78405900 | 0.55884500 |
| H | 1.96242800 | 2.65244800 | -1.14423000 |
| C | -3.76559900 | 0.56011300 | -1.13854300 |
| H | -4.34139800 | 0.58564900 | -2.05895300 |
| C | 0.04362300 | 2.85473800 | 1.63835200 |
| H | -1.02461300 | 2.87410900 | 1.86544900 |
| H | 0.44094000 | 3.86084400 | 1.78357000 |
| H | 0.54126500 | 2.18257900 | 2.33737100 |
| C | -0.40064000 | 3.43809700 | -0.73015400 |
| H | -0.21521100 | 3.19402000 | -1.77577800 |
| H | 0.00934500 | 4.43016000 | -0.53253600 |

| | | | |
|---|---|---|---|
| H | -1.47861800 | 3.47133100 | -0.55560200 |
| C | -1.47242800 | 0.13064300 | 2.45469000 |
| H | -1.58672300 | 0.95296300 | 3.16473900 |
| H | -1.83905000 | -0.77659000 | 2.93490900 |
| H | -0.41042700 | -0.01347400 | 2.25817900 |
| C | -1.77025100 | 1.07102300 | -2.54269500 |
| H | -2.22448900 | 0.41748500 | -3.28749800 |
| H | -1.93024400 | 2.10062100 | -2.87008400 |
| H | -0.69801300 | 0.87579000 | -2.53597300 |
| C | -5.88397000 | -0.00053400 | 0.09989500 |
| H | -6.42018600 | 0.92551600 | 0.32007700 |
| H | -6.25181000 | -0.37463700 | -0.85569000 |
| H | -6.14176100 | -0.72193300 | 0.87549100 |
| C | 2.01933100 | 0.72692300 | -0.14841300 |
| C | 2.65841400 | 0.22876900 | 1.16212600 |
| C | 2.88804300 | 0.27088900 | -1.32358800 |
| C | 4.10147800 | 0.69820600 | 1.30645600 |
| H | 2.62707800 | -0.86677700 | 1.15111700 |
| H | 2.05357900 | 0.55141400 | 2.01636300 |
| C | 4.31792200 | 0.77634100 | -1.18484700 |
| H | 2.87855400 | -0.82557400 | -1.34070200 |
| H | 2.43539000 | 0.61150600 | -2.26078500 |
| C | 4.93581700 | 0.26041100 | 0.10890200 |
| H | 4.52070100 | 0.30255600 | 2.23399000 |
| H | 4.13848700 | 1.79087200 | 1.38874900 |
| H | 4.91038200 | 0.46500500 | -2.04754500 |
| H | 4.32287800 | 1.87412600 | -1.17669600 |
| H | 5.96580300 | 0.60867800 | 0.21231500 |
| H | 4.96647800 | -0.83482000 | 0.07547700 |

48

j-5-1 carbene fragment

| | | | |
|---|---|---|---|
| C | 2.06521300 | 1.23406900 | -0.16689300 |
| N | 0.03443500 | -0.12947500 | 0.00920100 |
| C | -0.57053900 | -0.29284700 | 1.37741800 |

| | | | |
|---|---|---|---|
| C | 3.44958800 | 1.30490700 | -0.28939900 |
| H | 3.91868700 | 2.28429400 | -0.31510700 |
| C | -0.76882600 | -0.00916000 | -1.01925300 |
| C | 2.22150100 | -1.18778600 | -0.34655800 |
| C | 4.24045700 | 0.16906700 | -0.41354200 |
| C | 1.46250700 | -0.02997200 | -0.14777700 |
| C | -2.04084400 | -0.50916700 | 1.00727400 |
| H | -2.71564400 | 0.02706700 | 1.67951000 |
| H | -2.27862100 | -1.57551700 | 1.08221900 |
| C | 3.60294100 | -1.06360200 | -0.46546300 |
| H | 4.19254200 | -1.96112400 | -0.62951100 |
| C | -0.33665200 | 0.95800500 | 2.21589600 |
| H | 0.73299900 | 1.12650500 | 2.36336900 |
| H | -0.79788700 | 0.82380600 | 3.19605400 |
| H | -0.77077200 | 1.84208800 | 1.74929600 |
| C | 0.02081000 | -1.48805900 | 2.11005300 |
| H | -0.17119900 | -2.41402700 | 1.56861900 |
| H | -0.44531800 | -1.56613900 | 3.09429700 |
| H | 1.09831800 | -1.37269200 | 2.25170900 |
| C | 1.27592200 | 2.51425800 | -0.13164100 |
| H | 1.21394400 | 2.92557100 | 0.87778900 |
| H | 1.76548800 | 3.26025400 | -0.75799300 |
| H | 0.26183300 | 2.37106200 | -0.50533800 |
| C | 1.60331100 | -2.54962800 | -0.50452200 |
| H | 2.11504500 | -3.09231600 | -1.29990800 |
| H | 1.69951600 | -3.14127700 | 0.40829600 |
| H | 0.54686700 | -2.48661700 | -0.76273700 |
| C | 5.73600700 | 0.27369900 | -0.50914600 |
| H | 6.19090600 | 0.25361200 | 0.48399200 |
| H | 6.15380100 | -0.55689200 | -1.07867400 |
| H | 6.03624600 | 1.20601600 | -0.98834400 |
| C | -2.17333300 | -0.05122500 | -0.45695200 |
| C | -2.73741000 | 1.37233000 | -0.57223000 |
| C | -3.06660600 | -0.98146200 | -1.27587400 |

| | | | |
|---|---|---|---|
| C | -4.22037200 | 1.40885700 | -0.34769700 |
| H | -2.48711300 | 1.77187000 | -1.56222600 |
| H | -2.23883400 | 2.02906100 | 0.14966500 |
| C | -4.46898000 | -1.04416600 | -0.68344700 |
| H | -3.10568000 | -0.59969500 | -2.30020100 |
| H | -2.61906100 | -1.97925700 | -1.31931300 |
| C | -4.99385200 | 0.32687400 | -0.37722600 |
| H | -4.66633100 | 2.37765900 | -0.14551500 |
| H | -5.14796200 | -1.55352800 | -1.37257700 |
| H | -4.47097700 | -1.64969900 | 0.23198400 |
| H | -6.05689100 | 0.42629300 | -0.18190200 |

49

j-5-1 carbene-Au fragment

| | | | |
|---|---|---|---|
| Au | 0.24123300 | -1.91747800 | -0.09957600 |
| C | -2.20130300 | 0.41236200 | 1.19241200 |
| N | -0.17886500 | 1.04043100 | -0.03783100 |
| C | 0.39288200 | 2.40277800 | 0.21254300 |
| C | -3.57786100 | 0.21003500 | 1.19719600 |
| H | -4.05202900 | -0.10037800 | 2.12345200 |
| C | 0.68845300 | 0.08222100 | -0.25345000 |
| C | -2.33062700 | 0.87640400 | -1.19872300 |
| C | -4.35043300 | 0.35137900 | 0.05127100 |
| C | -1.59748400 | 0.79622200 | -0.01060100 |
| C | 1.86946500 | 2.16667300 | -0.13398800 |
| H | 2.53922900 | 2.67946000 | 0.55979000 |
| H | 2.06876400 | 2.55526100 | -1.13784400 |
| C | -3.70342400 | 0.66032700 | -1.13899500 |
| H | -4.27736400 | 0.70951300 | -2.05959900 |
| C | 0.16750500 | 2.82494500 | 1.65883600 |
| H | -0.90130500 | 2.88280600 | 1.87709700 |
| H | 0.59981600 | 3.81556700 | 1.80967800 |
| H | 0.63285900 | 2.13505400 | 2.36199500 |
| C | -0.25079500 | 3.43115100 | -0.70542600 |
| H | -0.07172200 | 3.18751600 | -1.75222600 |

| | | | |
|---|---|---|---|
| H | 0.18905300 | 4.40903700 | -0.50162900 |
| H | -1.32746900 | 3.49620700 | -0.53178400 |
| C | -1.42712800 | 0.13861500 | 2.45291500 |
| H | -1.51665500 | 0.96009500 | 3.16744300 |
| H | -1.82147500 | -0.75965000 | 2.92797200 |
| H | -0.36962900 | -0.03662000 | 2.25625300 |
| C | -1.69214200 | 1.12227200 | -2.53773300 |
| H | -2.16530000 | 0.48898000 | -3.28821200 |
| H | -1.81993100 | 2.15890000 | -2.85696700 |
| H | -0.62643100 | 0.89420300 | -2.53128400 |
| C | -5.83855300 | 0.15330400 | 0.09363400 |
| H | -6.34755100 | 1.09275500 | 0.32147100 |
| H | -6.21631000 | -0.20160600 | -0.86538600 |
| H | -6.11806800 | -0.56688300 | 0.86280000 |
| C | 2.07875900 | 0.64257100 | -0.12293700 |
| C | 2.68219500 | 0.14499900 | 1.20005200 |
| C | 2.97796500 | 0.16165000 | -1.26188900 |
| C | 4.14767600 | 0.45286900 | 1.28258900 |
| H | 2.50823000 | -0.93508700 | 1.28408500 |
| H | 2.15436100 | 0.60014000 | 2.04529200 |
| C | 4.35069700 | 0.81603900 | -1.17104700 |
| H | 3.06550200 | -0.92674500 | -1.18239800 |
| H | 2.50563000 | 0.37968100 | -2.22452600 |
| C | 4.89190700 | 0.76889900 | 0.22642700 |
| H | 4.60494500 | 0.40878300 | 2.26552200 |
| H | 5.04625500 | 0.32070200 | -1.85320200 |
| H | 4.30091000 | 1.85897300 | -1.50938300 |
| H | 5.94377900 | 0.99676400 | 0.36442400 |

62

j-6-1 carbene fragment

| | | | |
|---|---|---|---|
| C | -2.68094900 | -1.33360600 | -0.41016100 |
| N | -0.62534900 | -0.14016500 | 0.20435800 |
| C | -0.12499100 | -0.31582100 | 1.61116600 |
| C | -4.05395100 | -1.31125700 | -0.63609500 |

| | | | |
|---|---|---|---|
| H | -4.54888600 | -2.23817200 | -0.91142700 |
| C | 0.25055200 | -0.07175500 | -0.76703600 |
| C | -2.74887000 | 1.07168500 | -0.05373100 |
| C | -4.79886400 | -0.14137400 | -0.55360900 |
| C | -2.04136800 | -0.13532000 | -0.06762500 |
| C | 1.39052100 | -0.20000800 | 1.40311900 |
| H | 1.90774700 | -1.01961600 | 1.90707000 |
| H | 1.76297400 | 0.72969700 | 1.84364200 |
| C | -4.12097100 | 1.04087100 | -0.28737300 |
| H | -4.66812000 | 1.97928900 | -0.28599200 |
| C | -0.52936900 | -1.68327300 | 2.14740500 |
| H | -1.61667500 | -1.78998900 | 2.16743500 |
| H | -0.15656800 | -1.79305100 | 3.16754200 |
| H | -0.10408300 | -2.48232400 | 1.53818100 |
| C | -0.67019900 | 0.76530400 | 2.53166100 |
| H | -0.35208900 | 1.75554900 | 2.20297600 |
| H | -0.28600100 | 0.60063600 | 3.54026800 |
| H | -1.76188000 | 0.73579200 | 2.57313100 |
| C | -1.93972400 | -2.62687500 | -0.61132700 |
| H | -1.96889600 | -3.25411000 | 0.28181300 |
| H | -2.40558700 | -3.19006700 | -1.42022900 |
| H | -0.89651000 | -2.45486200 | -0.87563400 |
| C | -2.08130200 | 2.40647200 | 0.13112200 |
| H | -2.54887400 | 3.14150000 | -0.52473200 |
| H | -2.18386700 | 2.77111900 | 1.15525400 |
| H | -1.02035300 | 2.36326500 | -0.11163000 |
| C | -6.28575500 | -0.15252700 | -0.76707600 |
| H | -6.81154300 | -0.33249100 | 0.17357100 |
| H | -6.63473300 | 0.80242400 | -1.16090000 |
| H | -6.57863800 | -0.94001600 | -1.46210900 |
| C | 1.61441300 | -0.21957800 | -0.12501900 |
| C | 2.15270500 | -1.57861900 | -0.60342900 |
| C | 2.56100300 | 0.89724800 | -0.60918700 |
| C | 3.58267500 | -1.85611800 | -0.15354200 |

| | | | |
|---|---|---|---|
| H | 2.11489100 | -1.59314700 | -1.70005600 |
| H | 1.48776800 | -2.37958900 | -0.25385800 |
| C | 3.96828400 | 0.63263400 | -0.07813600 |
| H | 2.60883700 | 0.78945200 | -1.70282500 |
| C | 4.48448800 | -0.70757700 | -0.58506200 |
| H | 3.60609400 | -1.90601800 | 0.94334900 |
| H | 4.64667800 | 1.43155400 | -0.38506600 |
| H | 3.96418000 | 0.63048000 | 1.01977700 |
| H | 5.50715900 | -0.88665000 | -0.23972500 |
| H | 4.51653300 | -0.68732700 | -1.68311200 |
| C | 4.07108100 | -3.18428800 | -0.71136700 |
| H | 5.09165800 | -3.39940900 | -0.38754400 |
| H | 3.43094500 | -4.01058300 | -0.39475000 |
| H | 4.06451100 | -3.15564800 | -1.80484300 |
| C | 1.97469500 | 2.30283500 | -0.31184200 |
| C | 2.95718700 | 3.29575300 | 0.30038500 |
| H | 3.76957300 | 3.52450000 | -0.39446000 |
| H | 2.44530400 | 4.23419200 | 0.52394300 |
| H | 3.40102000 | 2.92433200 | 1.22652700 |
| C | 1.36455200 | 2.89848000 | -1.57782600 |
| H | 2.15298900 | 3.10036700 | -2.30938000 |
| H | 0.65203600 | 2.20481200 | -2.02917300 |
| H | 0.85443200 | 3.84182800 | -1.36482200 |
| H | 1.16035200 | 2.17371400 | 0.41480800 |

63

j-6-1 carbene-Au fragment

| | | | |
|---|---|---|---|
| Au | 0.18360600 | -1.60809100 | -1.00898600 |
| C | 2.65090700 | 1.32194300 | -0.75905800 |
| N | 0.60803800 | 0.90883900 | 0.53037500 |
| C | 0.05950600 | 2.00646500 | 1.38886800 |
| C | 4.02427600 | 1.15959200 | -0.90440400 |
| H | 4.51106500 | 1.61032400 | -1.76399100 |
| C | -0.27291200 | 0.18616500 | -0.10941300 |
| C | 2.74067500 | -0.10451000 | 1.21536700 |

| | | | |
|---|---|---|---|
| C | 4.77970100 | 0.41119000 | -0.00943200 |
| C | 2.02747600 | 0.72518400 | 0.34431200 |
| C | -1.45437200 | 1.76420000 | 1.27366900 |
| H | -1.97668500 | 2.69845300 | 1.05694200 |
| H | -1.83612500 | 1.39217200 | 2.22376000 |
| C | 4.11362300 | -0.23173900 | 1.02504400 |
| H | 4.66933400 | -0.88464500 | 1.69133300 |
| C | 0.47614700 | 3.36130900 | 0.83364500 |
| H | 1.56439300 | 3.45170400 | 0.79889100 |
| H | 0.08673000 | 4.14677500 | 1.48346600 |
| H | 0.07321400 | 3.51288000 | -0.16855300 |
| C | 0.54961300 | 1.86439100 | 2.82091100 |
| H | 0.22351500 | 0.91413500 | 3.24744500 |
| H | 0.12831000 | 2.67291300 | 3.42117200 |
| H | 1.63896800 | 1.92859000 | 2.87422400 |
| C | 1.89375200 | 2.06587300 | -1.82412900 |
| H | 1.84173200 | 3.13621200 | -1.61594400 |
| H | 2.40135700 | 1.94331800 | -2.78050600 |
| H | 0.87680000 | 1.68808100 | -1.93591500 |
| C | 2.08427500 | -0.92628700 | 2.28913900 |
| H | 2.56661000 | -1.90222700 | 2.34011200 |
| H | 2.17587700 | -0.45944500 | 3.27212900 |
| H | 1.02804200 | -1.09245600 | 2.08120600 |
| C | 6.26640900 | 0.27922500 | -0.17455600 |
| H | 6.78625400 | 1.09158900 | 0.33866100 |
| H | 6.62732700 | -0.65992200 | 0.24490300 |
| H | 6.55114000 | 0.31978400 | -1.22616900 |
| C | -1.65668800 | 0.71727200 | 0.16124900 |
| C | -2.10678400 | 1.35928000 | -1.16563300 |
| C | -2.62702000 | -0.42312900 | 0.51870000 |
| C | -3.54875100 | 1.85289100 | -1.13856300 |
| H | -2.00332000 | 0.60582800 | -1.95850500 |
| H | -1.43056300 | 2.18551500 | -1.42010100 |
| C | -4.06238000 | 0.08787900 | 0.56908200 |

| | | | |
|---|---|---|---|
| H | -2.55911100 | -1.11448500 | -0.33492500 |
| C | -4.47163600 | 0.70205800 | -0.76111500 |
| H | -3.64320800 | 2.62712100 | -0.36462700 |
| H | -4.73228600 | -0.73673100 | 0.83033300 |
| H | -4.16324900 | 0.83837100 | 1.36446100 |
| H | -5.50803500 | 1.05014500 | -0.72799300 |
| H | -4.41312800 | -0.06171800 | -1.54776500 |
| C | -3.92860700 | 2.45418100 | -2.48303100 |
| H | -4.95803300 | 2.81783900 | -2.47605600 |
| H | -3.27393600 | 3.28679100 | -2.74898800 |
| H | -3.84468700 | 1.69745000 | -3.26806300 |
| C | -2.21234000 | -1.23128300 | 1.77378300 |
| C | -2.96371600 | -0.88796800 | 3.06142200 |
| H | -4.01580900 | -1.17199700 | 2.97966300 |
| H | -2.53933000 | -1.45571500 | 3.89194500 |
| H | -2.93060100 | 0.16843100 | 3.33161500 |
| C | -2.39336900 | -2.72033300 | 1.49529700 |
| H | -3.43955600 | -2.92200400 | 1.24066900 |
| H | -1.76775700 | -3.03933600 | 0.65793500 |
| H | -2.14087100 | -3.32248600 | 2.37081800 |
| H | -1.14225800 | -1.04700600 | 1.94677500 |

34

k-1-1 carbene fragment

| | | | |
|---|---|---|---|
| C | -1.41279100 | -1.18695800 | -0.01615800 |
| N | 0.66690200 | 0.10278800 | -0.11482800 |
| C | 1.37446400 | 0.36919700 | 1.18400200 |
| C | -2.78854600 | -1.30780800 | -0.07340500 |
| H | -3.26909800 | -2.26959700 | 0.02986800 |
| C | 1.37334200 | -0.16671200 | -1.18904400 |
| C | -1.54842200 | 1.14941100 | -0.36922200 |
| C | -3.51829200 | -0.15173600 | -0.28058500 |
| C | -0.75409100 | 0.03071100 | -0.14869500 |
| C | 2.81882400 | 0.46220900 | 0.68491200 |
| H | 3.50677500 | -0.05376600 | 1.35878500 |

| | | | |
|---|---|---|---|
| H | 3.12194300 | 1.51183800 | 0.63906100 |
| C | -2.92819000 | 1.08884700 | -0.43576200 |
| H | -3.51683800 | 1.97682200 | -0.61372900 |
| C | 1.13751600 | -0.78546400 | 2.14991200 |
| H | 0.07460800 | -0.88103500 | 2.38641600 |
| H | 1.67186800 | -0.59240300 | 3.08177000 |
| H | 1.48538700 | -1.73125700 | 1.73565700 |
| C | 0.88551900 | 1.66648200 | 1.80939300 |
| H | 1.01612800 | 2.50353500 | 1.12387600 |
| H | 1.45832800 | 1.86433200 | 2.71726400 |
| H | -0.16962800 | 1.59910100 | 2.08768900 |
| C | 2.81813500 | -0.14203800 | -0.73484800 |
| C | 3.27126500 | -1.60617100 | -0.73928000 |
| H | 3.20043400 | -2.01921400 | -1.74619800 |
| H | 4.30927400 | -1.67118900 | -0.40211900 |
| H | 2.65342300 | -2.22119100 | -0.08130700 |
| C | 3.67910400 | 0.65609200 | -1.70530700 |
| H | 4.72240000 | 0.65300100 | -1.37703700 |
| H | 3.62422800 | 0.22012400 | -2.70373500 |
| H | 3.34208300 | 1.69291300 | -1.76704400 |
| F | -0.95335900 | 2.32672700 | -0.54177200 |
| F | -4.84780000 | -0.23812300 | -0.34014500 |
| F | -0.68180300 | -2.28392700 | 0.16181600 |

35

k-1-1 carbene-Au fragment

| | | | |
|---|---|---|---|
| Au | 1.01231600 | -1.66139700 | -0.07674800 |
| C | -1.81861900 | 0.07476300 | 1.12892000 |
| N | 0.02827300 | 1.14836500 | -0.06712800 |
| C | 0.32021900 | 2.58067000 | 0.24535500 |
| C | -3.08412300 | -0.47546600 | 1.18619300 |
| H | -3.46224700 | -0.91022200 | 2.09961200 |
| C | 1.06530500 | 0.36947400 | -0.30758800 |
| C | -2.09566700 | 0.59712000 | -1.16948500 |
| C | -3.83344900 | -0.46604700 | 0.02363900 |

| | | | |
|---|---|---|---|
| C | -1.29555300 | 0.63349900 | -0.03402400 |
| C | 1.81159800 | 2.64331300 | -0.10977800 |
| H | 2.36477300 | 3.25681100 | 0.60392500 |
| H | 1.92757200 | 3.09216900 | -1.09982300 |
| C | -3.36827100 | 0.05952400 | -1.16832900 |
| H | -3.96744400 | 0.04008400 | -2.06671600 |
| C | 0.02839100 | 2.86985900 | 1.71233800 |
| H | -1.03135500 | 2.71894700 | 1.93080800 |
| H | 0.27154200 | 3.91178000 | 1.92714700 |
| H | 0.61094200 | 2.22897100 | 2.37282200 |
| C | -0.52255900 | 3.49093300 | -0.63464700 |
| H | -0.35740700 | 3.27524900 | -1.69008700 |
| H | -0.24662500 | 4.52876500 | -0.44081500 |
| H | -1.58675900 | 3.37846000 | -0.41245700 |
| C | 2.32073100 | 1.18923600 | -0.14124300 |
| C | 2.97551600 | 0.77643800 | 1.18324000 |
| H | 3.30981200 | -0.26075600 | 1.13084200 |
| H | 3.83725600 | 1.42143600 | 1.37345400 |
| H | 2.28148300 | 0.85771400 | 2.02180700 |
| C | 3.29281400 | 0.92721500 | -1.28533300 |
| H | 4.19882400 | 1.52374800 | -1.15238600 |
| H | 3.56714000 | -0.12953800 | -1.30315400 |
| H | 2.84645300 | 1.18270500 | -2.24808200 |
| F | -1.60424900 | 1.08686000 | -2.30409700 |
| F | -5.05715700 | -0.99090400 | 0.05141800 |
| F | -1.06224500 | 0.06763500 | 2.22051600 |

34

l-1-1 carbene fragment

| | | | |
|---|---|---|---|
| C | -1.10098400 | -1.18271500 | -0.10837900 |
| N | 1.00207800 | 0.09094800 | -0.03537900 |
| C | 1.74887800 | 0.16209600 | 1.27474000 |
| C | -2.48624300 | -1.24643300 | -0.13143600 |
| H | -2.98496500 | -2.20499900 | -0.15142600 |
| C | 1.68106000 | 0.01191300 | -1.15575700 |

| | | | |
|---|---|---|---|
| C | -1.18953000 | 1.20073600 | -0.16858800 |
| C | -3.21159200 | -0.06769400 | -0.15215000 |
| C | -0.42144700 | 0.03764700 | -0.07618500 |
| C | 3.17139400 | 0.37225000 | 0.74839200 |
| H | 3.89544700 | -0.21729100 | 1.31570200 |
| H | 3.44535100 | 1.42599000 | 0.85027700 |
| C | -2.57530900 | 1.16118900 | -0.19260700 |
| H | -3.14349100 | 2.07799600 | -0.26044500 |
| C | 1.58781400 | -1.13280400 | 2.06258400 |
| H | 0.53757900 | -1.31760600 | 2.30228400 |
| H | 2.13574700 | -1.04197900 | 3.00228000 |
| H | 1.97541900 | -1.99114700 | 1.51674300 |
| C | 1.26554900 | 1.31904400 | 2.13652700 |
| H | 1.42286600 | 2.27644800 | 1.64375200 |
| H | 1.82574600 | 1.31593400 | 3.07341600 |
| H | 0.20485300 | 1.21634400 | 2.38183000 |
| C | 3.13806400 | -0.01080400 | -0.74560900 |
| C | 3.61213500 | -1.44769100 | -0.98869700 |
| H | 3.51178500 | -1.70311700 | -2.04435600 |
| H | 4.66213500 | -1.54359500 | -0.69963100 |
| H | 3.02572400 | -2.16785000 | -0.41372500 |
| C | 3.95444200 | 0.94574600 | -1.60505300 |
| H | 5.00618500 | 0.92122900 | -1.30597800 |
| H | 3.88138400 | 0.66421100 | -2.65637700 |
| H | 3.59125800 | 1.97053500 | -1.50068600 |
| Cl | -4.92975300 | -0.13160300 | -0.16937400 |
| Cl | -0.23215000 | -2.65952000 | -0.20543400 |
| Cl | -0.42944800 | 2.72920500 | -0.34241000 |

35

l-1-1 carbene-Au fragment

| | | | |
|---|---|---|---|
| Au | 1.12702300 | -1.74460400 | -0.18250400 |
| C | -1.58726500 | 0.18696800 | 1.19763400 |
| N | 0.38697300 | 1.12530800 | 0.07013000 |
| C | 0.81060500 | 2.52541700 | 0.41067400 |

| | | | |
|---|---|---|---|
| C | -2.91541600 | -0.21085200 | 1.18579500 |
| H | -3.36868300 | -0.61185600 | 2.08105200 |
| C | 1.34513300 | 0.28510400 | -0.26741000 |
| C | -1.71329800 | 0.72687400 | -1.12916100 |
| C | -3.63521100 | -0.11458600 | 0.00707000 |
| C | -0.97404500 | 0.70909700 | 0.05589000 |
| C | 2.28081800 | 2.47483500 | -0.02763900 |
| H | 2.92035100 | 3.02933600 | 0.66188200 |
| H | 2.37605700 | 2.93017100 | -1.01720000 |
| C | -3.04116100 | 0.33270500 | -1.16169400 |
| H | -3.59388200 | 0.35892700 | -2.08990800 |
| C | 0.63926600 | 2.82138300 | 1.89546800 |
| H | -0.41113300 | 2.75768200 | 2.18721800 |
| H | 0.98096300 | 3.84053300 | 2.08539100 |
| H | 1.21459700 | 2.13968100 | 2.51879300 |
| C | 0.00398800 | 3.54241100 | -0.38361900 |
| H | 0.13421800 | 3.40658000 | -1.45551900 |
| H | 0.34567900 | 4.54379400 | -0.11647300 |
| H | -1.06026200 | 3.47627500 | -0.14327400 |
| C | 2.66942300 | 0.98734400 | -0.11326200 |
| C | 3.31693900 | 0.47456000 | 1.17943200 |
| H | 3.56518200 | -0.58319700 | 1.07959100 |
| H | 4.23148900 | 1.04028500 | 1.37467000 |
| H | 2.64716700 | 0.57649900 | 2.03573500 |
| C | 3.58321200 | 0.68466300 | -1.29417800 |
| H | 4.53656300 | 1.20593500 | -1.17664300 |
| H | 3.77309800 | -0.38913000 | -1.35043300 |
| H | 3.12597000 | 1.00175000 | -2.23365800 |
| Cl | -5.28402300 | -0.59563400 | -0.01649500 |
| Cl | -0.68493700 | -0.06343200 | 2.63305300 |
| Cl | -0.96391900 | 1.16893400 | -2.60739900 |

43

m-1-1 carbene fragment

| | | | |
|---|---|---|---|
| C | 0.65257900 | 1.21494400 | -0.13990100 |

| | | | |
|---|---|---|---|
| N | -1.50095400 | 0.00438500 | -0.00673800 |
| C | -2.20640900 | -0.02063300 | 1.32999100 |
| C | 2.04273700 | 1.19528500 | -0.13275900 |
| H | 2.58781700 | 2.12943200 | -0.17355300 |
| C | -2.21342900 | -0.02224000 | -1.10856200 |
| C | 0.63447700 | -1.18694000 | -0.12769100 |
| C | 2.72754600 | -0.00406100 | -0.10049900 |
| C | -0.07590600 | 0.02009400 | -0.08710800 |
| C | -3.61405700 | -0.39083000 | 0.85757500 |
| H | -4.37762600 | 0.15241200 | 1.41885600 |
| H | -3.77621700 | -1.46115000 | 1.01520800 |
| C | 2.02280300 | -1.19334300 | -0.12497500 |
| H | 2.55134000 | -2.13792400 | -0.15755500 |
| C | -2.13574200 | 1.34688900 | 1.99906600 |
| H | -1.10135700 | 1.62346800 | 2.21584200 |
| H | -2.66872300 | 1.30023300 | 2.95029500 |
| H | -2.58750500 | 2.12669700 | 1.38952000 |
| C | -1.60779500 | -1.04877300 | 2.27924500 |
| H | -1.68537800 | -2.06111200 | 1.88944500 |
| H | -2.15702500 | -1.01030000 | 3.22158600 |
| H | -0.56045400 | -0.82785500 | 2.50255400 |
| C | 0.06903900 | 2.61165300 | -0.25588200 |
| C | 0.03525400 | -2.58005800 | -0.21973200 |
| C | 4.23134600 | -0.02229800 | -0.03311500 |
| C | -3.65647300 | -0.08462400 | -0.65461000 |
| C | -4.28765800 | 1.27514600 | -0.96723800 |
| H | -4.20500100 | 1.49086700 | -2.03330600 |
| H | -5.34574500 | 1.25856000 | -0.69326000 |
| H | -3.80317500 | 2.08642100 | -0.42193000 |
| C | -4.39081400 | -1.17093600 | -1.43321600 |
| H | -5.42739400 | -1.24239200 | -1.09190400 |
| H | -4.38625700 | -0.94028100 | -2.49922000 |
| H | -3.91324600 | -2.14290100 | -1.29187300 |
| F | 4.76269900 | 1.07925100 | -0.56571700 |

| | | | |
|---|---|---|---|
| F | 4.65664100 | -0.09425900 | 1.23417700 |
| F | 4.73815000 | -1.07545500 | -0.67748000 |
| F | 0.22609900 | 3.28774600 | 0.89389100 |
| F | 0.73956300 | 3.29851800 | -1.18901700 |
| F | 0.32717400 | -3.27989400 | 0.88932300 |
| F | -1.27909200 | -2.64705000 | -0.39315800 |
| F | 0.59775800 | -3.23709300 | -1.24031200 |
| F | -1.21369900 | 2.66706800 | -0.58249500 |

44

m-1-1 carbene-Au fragment

| | | | |
|---|---|---|---|
| Au | -1.48810400 | -1.03283600 | -1.51751300 |
| C | 1.24777800 | 1.32508300 | -0.20547600 |
| N | -0.86768800 | 0.61314600 | 0.85897200 |
| C | -1.30353700 | 1.23040000 | 2.15993900 |
| C | 2.59545400 | 1.09908000 | -0.45922200 |
| H | 3.16058800 | 1.82811100 | -1.02507100 |
| C | -1.82199200 | 0.28055300 | 0.01429200 |
| C | 1.11850600 | -0.80584100 | 0.90525500 |
| C | 3.20854200 | -0.05709200 | -0.01438600 |
| C | 0.50247300 | 0.39344500 | 0.52602700 |
| C | -2.79574600 | 0.88290600 | 2.12673100 |
| H | -3.40045200 | 1.69303300 | 2.53892300 |
| H | -2.96685200 | -0.01199800 | 2.73122100 |
| C | 2.46632600 | -1.01746000 | 0.64618200 |
| H | 2.92635000 | -1.95022600 | 0.94661300 |
| C | -1.03723700 | 2.73143700 | 2.13257000 |
| H | 0.03338300 | 2.93363800 | 2.05769400 |
| H | -1.39219800 | 3.16718300 | 3.06770200 |
| H | -1.54484600 | 3.22451200 | 1.30629300 |
| C | -0.57985600 | 0.64019000 | 3.36104200 |
| H | -0.79989700 | -0.41479300 | 3.50093200 |
| H | -0.90886000 | 1.17372200 | 4.25422000 |
| H | 0.50187100 | 0.77553200 | 3.28007200 |
| C | 0.71639800 | 2.61386900 | -0.80647800 |

| | | | |
|---|---|---|---|
| C | 0.44644500 | -1.98980000 | 1.58185000 |
| C | 4.68360700 | -0.26215200 | -0.24107600 |
| C | -3.15220100 | 0.57369200 | 0.65765100 |
| C | -3.77551600 | 1.76284200 | -0.08053300 |
| H | -3.91156100 | 1.51347000 | -1.13430700 |
| H | -4.75114700 | 1.98958200 | 0.35586400 |
| H | -3.15307100 | 2.65637800 | -0.02236400 |
| C | -4.08252100 | -0.63082400 | 0.54684500 |
| H | -5.01142700 | -0.42370300 | 1.08470200 |
| H | -4.31646900 | -0.83813400 | -0.49857400 |
| H | -3.61907200 | -1.52290300 | 0.97321300 |
| F | 5.09554700 | 0.33288000 | -1.36137300 |
| F | 5.39904300 | 0.25347100 | 0.76634200 |
| F | 4.99356600 | -1.55617200 | -0.32373200 |
| F | 1.11547600 | 3.67294100 | -0.08390100 |
| F | 1.21989700 | 2.78115700 | -2.03227500 |
| F | 0.94558900 | -2.14605000 | 2.82090400 |
| F | -0.87637200 | -1.92487100 | 1.69960600 |
| F | 0.73040400 | -3.10750900 | 0.91410700 |
| F | -0.60469400 | 2.68155700 | -0.91675400 |

49

m-2-1 carbene fragment

| | | | |
|---|---|---|---|
| C | 1.02890500 | 1.18481200 | -0.08050300 |
| N | -1.16119500 | 0.05708200 | 0.10518200 |
| C | -1.78115900 | 0.07345400 | 1.48518200 |
| C | 2.41675500 | 1.13246400 | -0.12517200 |
| H | 2.98182200 | 2.05554500 | -0.14865500 |
| C | -1.94750200 | 0.03566000 | -0.94767000 |
| C | 0.94517400 | -1.21046700 | -0.16020300 |
| C | 3.07232500 | -0.08214600 | -0.15913200 |
| C | 0.26245600 | 0.01071000 | -0.04733100 |
| C | -3.24974000 | 0.29541500 | 1.10666100 |
| H | -3.52522200 | 1.33329300 | 1.30968100 |
| H | -3.90545100 | -0.34181600 | 1.70504800 |

| | | | |
|---|---|---|---|
| C | 2.33393400 | -1.24845400 | -0.20176200 |
| H | 2.83599200 | -2.20372100 | -0.28480200 |
| C | -1.22956300 | 1.19900900 | 2.34780000 |
| H | -0.15349200 | 1.09539800 | 2.51079400 |
| H | -1.71331200 | 1.15274600 | 3.32517700 |
| H | -1.43532600 | 2.17696600 | 1.91719400 |
| C | -1.53040400 | -1.24791500 | 2.20223800 |
| H | -1.93804100 | -2.09725000 | 1.65761400 |
| H | -2.00437700 | -1.21067300 | 3.18450100 |
| H | -0.46134000 | -1.41115500 | 2.36043700 |
| C | 0.49909200 | 2.60604900 | -0.11161900 |
| C | 0.32133400 | -2.59074500 | -0.27504300 |
| C | 4.57659700 | -0.13569200 | -0.14225000 |
| C | -3.36237500 | -0.00860200 | -0.40112400 |
| C | -4.26967800 | 0.97516300 | -1.14911200 |
| H | -4.44973000 | 0.56756300 | -2.14949100 |
| H | -5.23842000 | 1.00304700 | -0.63956600 |
| C | -3.86167500 | -1.45043800 | -0.64488900 |
| H | -3.62661500 | -1.72204300 | -1.67807600 |
| H | -3.29138600 | -2.13124400 | -0.00929700 |
| F | 5.11338400 | 0.94763500 | -0.70654200 |
| F | 5.04304500 | -0.20071700 | 1.11079100 |
| F | 5.03767300 | -1.20722800 | -0.78987900 |
| F | 0.79571900 | 3.24282100 | 1.03289900 |
| F | 1.10982900 | 3.28716900 | -1.08957500 |
| F | 0.51673200 | -3.28953300 | 0.85532100 |
| F | -0.97468800 | -2.61727200 | -0.54236600 |
| F | 0.93287200 | -3.27559900 | -1.24976800 |
| F | -0.80402900 | 2.72799600 | -0.31230200 |
| C | -5.34933800 | -1.64386600 | -0.37850900 |
| H | -5.96199700 | -1.12385000 | -1.11522500 |
| H | -5.60868900 | -2.70217200 | -0.42196800 |
| H | -5.63275500 | -1.27380000 | 0.61045200 |
| C | -3.70723700 | 2.38233400 | -1.27250800 |

| | | | |
|---|---|---|---|
| H | -2.79625800 | 2.37743200 | -1.87138100 |
| H | -4.42660100 | 3.04884300 | -1.74984900 |
| H | -3.46268100 | 2.80940200 | -0.29641000 |

50

m-2-1 carbene-Au fragment

| | | | |
|---|---|---|---|
| Au | -1.23709800 | 0.61568800 | -1.82996900 |
| C | 1.43358900 | 1.00544000 | 0.72664400 |
| N | -0.66804300 | -0.26003200 | 0.95901700 |
| C | -1.11648700 | -0.59004400 | 2.36016900 |
| C | 2.79612300 | 1.05113500 | 0.45698800 |
| H | 3.32682100 | 1.98810000 | 0.56530800 |
| C | -1.61032200 | -0.10711600 | 0.06168100 |
| C | 1.39469700 | -1.30978600 | 0.08652800 |
| C | 3.46310600 | -0.07898300 | 0.03005100 |
| C | 0.71843100 | -0.19176000 | 0.59586000 |
| C | -2.63381600 | -0.38998600 | 2.22201700 |
| H | -2.92462500 | 0.54156500 | 2.71148900 |
| H | -3.17349100 | -1.20314700 | 2.71215900 |
| C | 2.75666900 | -1.24793100 | -0.17922600 |
| H | 3.26116100 | -2.12185900 | -0.57053900 |
| C | -0.49774500 | 0.34255300 | 3.38949000 |
| H | 0.59324100 | 0.27820600 | 3.38572800 |
| H | -0.84100000 | 0.03612900 | 4.37895800 |
| H | -0.79721900 | 1.37664900 | 3.23271000 |
| C | -0.73085200 | -2.02337200 | 2.70398700 |
| H | -1.18142300 | -2.74355000 | 2.02340500 |
| H | -1.07478700 | -2.24382800 | 3.71551600 |
| H | 0.35392600 | -2.15234300 | 2.68782700 |
| C | 0.86410900 | 2.35706100 | 1.11672400 |
| C | 0.78527700 | -2.65847200 | -0.25495900 |
| C | 4.95103800 | -0.04980100 | -0.20299500 |
| C | -2.94868900 | -0.32707900 | 0.71368600 |
| C | -3.95286500 | 0.77318300 | 0.34540000 |
| H | -4.21785500 | 0.63854900 | -0.70930900 |

| | | | |
|---|---|---|---|
| H | -4.86015900 | 0.59288300 | 0.92969000 |
| C | -3.45560600 | -1.68653500 | 0.17591700 |
| H | -3.36030600 | -1.67453000 | -0.91452100 |
| H | -2.79085000 | -2.47397400 | 0.53828400 |
| F | 5.38421100 | 1.18174100 | -0.47111700 |
| F | 5.61229600 | -0.47773000 | 0.87963700 |
| F | 5.30295200 | -0.84140800 | -1.21696400 |
| F | 1.30746600 | 2.71389000 | 2.33413900 |
| F | 1.29829900 | 3.28883700 | 0.26650700 |
| F | 1.13782300 | -3.57754500 | 0.65873200 |
| F | -0.53787600 | -2.67778000 | -0.34738300 |
| F | 1.26424000 | -3.08469500 | -1.42579500 |
| F | -0.45971200 | 2.43148800 | 1.13811200 |
| C | -4.88902200 | -2.01177500 | 0.57479100 |
| H | -5.60525300 | -1.36405300 | 0.06982200 |
| H | -5.12908500 | -3.04078300 | 0.30684900 |
| H | -5.04266200 | -1.90547400 | 1.65201500 |
| C | -3.47131200 | 2.19866600 | 0.56666500 |
| H | -2.66663500 | 2.44900500 | -0.12662200 |
| H | -4.28574800 | 2.90460000 | 0.40267900 |
| H | -3.10219400 | 2.35513900 | 1.58366400 |

58

m-3-1 carbene fragment

| | | | |
|---|---|---|---|
| C | 1.71097900 | 1.20191000 | 0.01415000 |
| N | -0.33135400 | -0.11117900 | 0.47793300 |
| C | -0.78877600 | -0.22008600 | 1.90999600 |
| C | 3.07877500 | 1.24903400 | -0.22687500 |
| H | 3.56887200 | 2.20910000 | -0.32525700 |
| C | -1.22521800 | -0.11070100 | -0.48091400 |
| C | 1.79459800 | -1.19720000 | -0.07572100 |
| C | 3.80804300 | 0.08350300 | -0.36826500 |
| C | 1.05413100 | -0.02913000 | 0.14514300 |
| C | -2.24517500 | -0.61745600 | 1.66813400 |
| H | -2.89966800 | -0.15350500 | 2.40698200 |

| | | | |
|---|---|---|---|
| H | -2.34397900 | -1.70339800 | 1.77369400 |
| C | 3.16057800 | -1.13650800 | -0.31973700 |
| H | 3.71283100 | -2.05243600 | -0.48802400 |
| C | -0.64236900 | 1.11871000 | 2.62331900 |
| H | 0.40599800 | 1.42175200 | 2.67459300 |
| H | -1.00400100 | 1.01385200 | 3.64770200 |
| H | -1.21314100 | 1.90648400 | 2.13523700 |
| C | -0.00785700 | -1.26825700 | 2.68943500 |
| H | -0.12623900 | -2.26432000 | 2.26894400 |
| H | -0.38526900 | -1.29048200 | 3.71329600 |
| H | 1.05641800 | -1.02220900 | 2.73926400 |
| C | 1.05698200 | 2.57175500 | 0.06185800 |
| C | 1.24361000 | -2.61181300 | -0.12622000 |
| C | 5.29849400 | 0.14541900 | -0.57046000 |
| F | 5.66019200 | 1.25115400 | -1.22425100 |
| F | 5.94521500 | 0.15125300 | 0.60156700 |
| F | 5.74547200 | -0.90458000 | -1.26179800 |
| F | 1.36915600 | 3.20902200 | 1.20155900 |
| F | 1.53577500 | 3.32833500 | -0.93372100 |
| F | 1.74107700 | -3.33764300 | 0.88875900 |
| F | -0.07757400 | -2.72960100 | -0.08675200 |
| F | 1.65491700 | -3.20628300 | -1.25247800 |
| F | -0.26359800 | 2.57682200 | -0.05203000 |
| C | -2.57033400 | -0.22677500 | 0.20865100 |
| C | -3.30865400 | 1.13155300 | 0.07611600 |
| C | -3.43980500 | -1.29362100 | -0.48849600 |
| H | -2.70793600 | 1.91245300 | 0.55364400 |
| C | -4.68221000 | 1.05782700 | 0.74750000 |
| C | -3.51469100 | 1.48283400 | -1.40123500 |
| H | -2.92221700 | -2.25731000 | -0.40889100 |
| C | -4.79898100 | -1.37403700 | 0.20989600 |
| C | -3.65063400 | -0.94514600 | -1.96214300 |
| H | -5.17877400 | 2.02797300 | 0.63524500 |
| H | -4.59482600 | 0.86769400 | 1.82111700 |

| | | | |
|---|---|---|---|
| C | -5.52442000 | -0.03311600 | 0.08733100 |
| H | -2.55058500 | 1.57620700 | -1.90524500 |
| H | -4.02869800 | 2.44864400 | -1.46568400 |
| C | -4.36096000 | 0.40256900 | -2.07648200 |
| H | -4.67565000 | -1.63799900 | 1.26638600 |
| H | -5.39776800 | -2.16503500 | -0.25399700 |
| H | -2.68869300 | -0.90951600 | -2.47587600 |
| H | -4.26201900 | -1.72550500 | -2.42960800 |
| H | -6.49624300 | -0.09610600 | 0.58567000 |
| C | -5.72236100 | 0.31207000 | -1.38832800 |
| H | -4.49773300 | 0.65579500 | -3.13162400 |
| H | -6.33499900 | -0.45609100 | -1.87324600 |
| H | -6.25436300 | 1.26586900 | -1.47746900 |

59

m-3-1 carbene-Au fragment

| | | | |
|---|---|---|---|
| Au | 0.47164400 | 1.07816200 | -1.53906800 |
| C | -1.88704000 | -1.37272900 | -0.12895700 |
| N | 0.13797800 | -0.43138600 | 0.93148800 |
| C | 0.58565600 | -0.95738600 | 2.26259500 |
| C | -3.24729500 | -1.28544500 | -0.40191000 |
| H | -3.73335900 | -2.08386100 | -0.94706500 |
| C | 1.07748600 | -0.03565300 | 0.10009300 |
| C | -1.97349100 | 0.79963700 | 0.90308500 |
| C | -3.97219200 | -0.17824400 | -0.00471300 |
| C | -1.24216300 | -0.34786600 | 0.57103000 |
| C | 2.02581100 | -0.45197500 | 2.25331400 |
| H | 2.68145900 | -1.14119100 | 2.78328100 |
| H | 2.06514600 | 0.51216100 | 2.77156100 |
| C | -3.33133800 | 0.87211100 | 0.62564600 |
| H | -3.88062000 | 1.76819500 | 0.88541200 |
| C | 0.47659000 | -2.47845000 | 2.28888100 |
| H | -0.56531200 | -2.79254100 | 2.19683400 |
| H | 0.84683400 | -2.83866100 | 3.24995500 |
| H | 1.05491800 | -2.94915100 | 1.49692500 |

| | | | |
|---|---|---|---|
| C | -0.23034000 | -0.40430100 | 3.42180900 |
| H | -0.13517100 | 0.67372100 | 3.52030600 |
| H | 0.13795700 | -0.85887300 | 4.34293400 |
| H | -1.28711400 | -0.66705700 | 3.32830200 |
| C | -1.22509000 | -2.61842700 | -0.68914900 |
| C | -1.42096500 | 2.07102800 | 1.52663900 |
| C | -5.45715900 | -0.11869900 | -0.24976200 |
| F | -5.80645700 | -0.82056200 | -1.32855100 |
| F | -6.13474000 | -0.62538000 | 0.78823500 |
| F | -5.87724300 | 1.13525500 | -0.41991300 |
| F | -1.52719100 | -3.69318200 | 0.05707600 |
| F | -1.69303500 | -2.86565700 | -1.91510600 |
| F | -1.95560900 | 2.24644100 | 2.74827600 |
| F | -0.09963100 | 2.12783000 | 1.66726500 |
| F | -1.78760000 | 3.12392900 | 0.79723700 |
| F | 0.09846800 | -2.55912300 | -0.78340700 |
| C | 2.41942000 | -0.21988200 | 0.77527200 |
| C | 3.15027500 | -1.42606500 | 0.11879800 |
| C | 3.35622200 | 1.00306300 | 0.65148400 |
| H | 2.49805100 | -2.30310200 | 0.14614300 |
| C | 4.44875700 | -1.72409900 | 0.87270700 |
| C | 3.50090600 | -1.10895300 | -1.33876600 |
| H | 2.83876100 | 1.86984300 | 1.07988800 |
| C | 4.63495200 | 0.70526900 | 1.44466600 |
| C | 3.75881000 | 1.30450300 | -0.79136000 |
| H | 4.94737400 | -2.56660300 | 0.38143700 |
| H | 4.25674600 | -2.03100600 | 1.90409300 |
| C | 5.36089300 | -0.49855000 | 0.83673200 |
| H | 2.59371100 | -0.90134500 | -1.91438400 |
| H | 3.98141400 | -1.98845700 | -1.78065100 |
| C | 4.44772300 | 0.08972400 | -1.40473100 |
| H | 4.41080300 | 0.51649800 | 2.49957600 |
| H | 5.28557100 | 1.58477900 | 1.40749300 |
| H | 2.88988900 | 1.59257200 | -1.39034700 |

| | | | |
|---|---|---|---|
| H | 4.44486100 | 2.15886600 | -0.78651800 |
| H | 6.26900700 | -0.70227100 | 1.41111300 |
| C | 5.72359200 | -0.20537200 | -0.61837500 |
| H | 4.69449600 | 0.29838900 | -2.44893700 |
| H | 6.39942600 | 0.65484600 | -0.67226300 |
| H | 6.24663800 | -1.06623300 | -1.04927200 |

50

m-4-1 carbene fragment

| | | | |
|---|---|---|---|
| C | 1.23939900 | 1.21279500 | -0.11960900 |
| N | -0.89241600 | -0.00935100 | 0.16453400 |
| C | -1.50520100 | -0.03649300 | 1.54328000 |
| C | 2.62667900 | 1.20146300 | -0.20700000 |
| H | 3.16192600 | 2.13868000 | -0.28866200 |
| C | -1.67913000 | -0.03363700 | -0.88603700 |
| C | 1.23737100 | -1.18911200 | -0.09746800 |
| C | 3.31995000 | 0.00630700 | -0.21670500 |
| C | 0.52362700 | 0.01348800 | -0.01273000 |
| C | -2.94507400 | -0.39980800 | 1.17039100 |
| H | -3.65690600 | 0.15169800 | 1.78816700 |
| H | -3.10871700 | -1.46742000 | 1.34971900 |
| C | 2.62296200 | -1.18713800 | -0.18877400 |
| H | 3.15395000 | -2.12857100 | -0.25386900 |
| C | -1.38840100 | 1.33085200 | 2.20620700 |
| H | -0.34165400 | 1.60846300 | 2.35023500 |
| H | -1.85443900 | 1.28495700 | 3.19199800 |
| H | -1.88236500 | 2.10885800 | 1.62722000 |
| C | -0.84876800 | -1.06748400 | 2.45018100 |
| H | -0.95476500 | -2.07901000 | 2.06492800 |
| H | -1.33571100 | -1.02864400 | 3.42617600 |
| H | 0.21153000 | -0.85003800 | 2.60545300 |
| C | 0.64107300 | 2.60593600 | -0.20009800 |
| C | 0.64193700 | -2.58593000 | -0.14550900 |
| C | 4.82493000 | 0.00106700 | -0.25198500 |
| F | 5.30746700 | 1.08777300 | -0.85710300 |

| | | | |
|---|---|---|---|
| F | 5.33688200 | -0.02278700 | 0.98475200 |
| F | 5.29728100 | -1.06961400 | -0.89338300 |
| F | 0.87241800 | 3.28678400 | 0.93432900 |
| F | 1.24332000 | 3.29315500 | -1.17869100 |
| F | 1.00641300 | -3.27941000 | 0.94599400 |
| F | -0.67997200 | -2.66040000 | -0.23710500 |
| F | 1.14382800 | -3.24471700 | -1.19638200 |
| F | -0.66091800 | 2.65426600 | -0.43902300 |
| C | -3.08692700 | -0.10581700 | -0.33894600 |
| C | -3.76431900 | 1.24434300 | -0.63525800 |
| C | -3.85521700 | -1.21079900 | -1.07102200 |
| C | -5.24584900 | 1.23584200 | -0.27360900 |
| H | -3.64847900 | 1.44379200 | -1.70614500 |
| H | -3.24413800 | 2.04923700 | -0.10839700 |
| C | -5.32178200 | -1.23461400 | -0.66073600 |
| H | -3.77479900 | -1.02187600 | -2.14679800 |
| H | -3.37333400 | -2.17409500 | -0.87751700 |
| C | -5.97748800 | 0.10271400 | -0.98226500 |
| H | -5.69007900 | 2.20068300 | -0.52882300 |
| H | -5.36595200 | 1.11527200 | 0.80948500 |
| H | -5.84087300 | -2.05121100 | -1.16719700 |
| H | -5.40247400 | -1.42872400 | 0.41663200 |
| H | -7.03361200 | 0.09573100 | -0.70338600 |
| H | -5.93130100 | 0.26837400 | -2.06529500 |

51

m-4-1 carbene-Au fragment

| | | | |
|---|---|---|---|
| Au | -0.92550000 | -1.11737100 | -1.63266400 |
| C | 1.57860300 | 1.36154000 | -0.16146300 |
| N | -0.52887400 | 0.50394800 | 0.80725000 |
| C | -1.05835200 | 1.07606700 | 2.09173100 |
| C | 2.94738500 | 1.22496500 | -0.36074000 |
| H | 3.48700700 | 1.99510000 | -0.89618700 |
| C | -1.42365400 | 0.12290200 | -0.07988300 |
| C | 1.54095400 | -0.78630700 | 0.92289100 |

| | | | |
|---|---|---|---|
| C | 3.61470200 | 0.10530600 | 0.09878900 |
| C | 0.86529000 | 0.37603100 | 0.53002400 |
| C | -2.52593100 | 0.64477600 | 1.98970600 |
| H | -3.18492200 | 1.42035800 | 2.38360200 |
| H | -2.67851800 | -0.25906200 | 2.58745000 |
| C | 2.90888500 | -0.90851500 | 0.71837900 |
| H | 3.41485500 | -1.81409900 | 1.02806400 |
| C | -0.88576000 | 2.59098500 | 2.08905700 |
| H | 0.17158700 | 2.86198100 | 2.05267200 |
| H | -1.29947100 | 2.99253000 | 3.01534700 |
| H | -1.39702500 | 3.05883400 | 1.24997900 |
| C | -0.35603800 | 0.51558600 | 3.31920700 |
| H | -0.51679500 | -0.55275700 | 3.43686900 |
| H | -0.75921400 | 1.01542500 | 4.20139900 |
| H | 0.71781200 | 0.71820000 | 3.29137900 |
| C | 0.99135900 | 2.61977000 | -0.77527100 |
| C | 0.91746000 | -2.01972100 | 1.55605100 |
| C | 5.10782600 | -0.00446700 | -0.06733000 |
| F | 5.53087400 | 0.64670600 | -1.15160600 |
| F | 5.74683700 | 0.52234100 | 0.98478900 |
| F | 5.49899100 | -1.27489400 | -0.17068700 |
| F | 1.29753900 | 3.69563200 | -0.03207600 |
| F | 1.52936000 | 2.82709700 | -1.98009500 |
| F | 1.36953400 | -2.16086100 | 2.81480300 |
| F | -0.41064300 | -2.04115300 | 1.61344600 |
| F | 1.30249400 | -3.10789500 | 0.88989900 |
| F | -0.32616100 | 2.60785800 | -0.93509300 |
| C | -2.79471500 | 0.31828100 | 0.50467600 |
| C | -3.47845100 | 1.45877700 | -0.27367700 |
| C | -3.63491100 | -0.95435500 | 0.34278000 |
| C | -4.92057300 | 1.66976800 | 0.17411500 |
| H | -3.45722200 | 1.18540600 | -1.33503200 |
| H | -2.90282800 | 2.38278700 | -0.17490600 |
| C | -5.05475700 | -0.73672400 | 0.84842400 |

| | | | |
|---|---|---|---|
| H | -3.65783200 | -1.21642100 | -0.72108700 |
| H | -3.14135200 | -1.78134300 | 0.86219600 |
| C | -5.72601400 | 0.38152900 | 0.06052400 |
| H | -5.37367600 | 2.46250600 | -0.42490200 |
| H | -4.94225600 | 2.01578100 | 1.21410300 |
| H | -5.62418100 | -1.66427400 | 0.76453800 |
| H | -5.03552800 | -0.47301100 | 1.91361900 |
| H | -6.74982900 | 0.53990900 | 0.40583600 |
| H | -5.78325700 | 0.08617800 | -0.99359100 |

48

m-5-1 carbene fragment

| | | | |
|---|---|---|---|
| C | 1.18837700 | 1.20894400 | -0.11979300 |
| N | -0.94611800 | -0.01316600 | 0.11500900 |
| C | -1.58734900 | -0.04427500 | 1.48294100 |
| C | 2.57706200 | 1.20040700 | -0.17926300 |
| H | 3.11254000 | 2.13871500 | -0.24510100 |
| C | -1.71051100 | -0.02539700 | -0.95207900 |
| C | 1.18961300 | -1.19346100 | -0.11157000 |
| C | 3.27211400 | 0.00611400 | -0.18150800 |
| C | 0.47314200 | 0.00765200 | -0.03359800 |
| C | -3.02186900 | -0.39005900 | 1.07512700 |
| H | -3.75702800 | 0.15316200 | 1.67366300 |
| H | -3.18698300 | -1.46150400 | 1.22970200 |
| C | 2.57682500 | -1.18864600 | -0.17484100 |
| H | 3.11074400 | -2.12872300 | -0.23496400 |
| C | -1.45877500 | 1.31047300 | 2.16886600 |
| H | -0.40985900 | 1.55953300 | 2.34640000 |
| H | -1.95298800 | 1.26030500 | 3.14051100 |
| H | -1.91576800 | 2.11117100 | 1.59140300 |
| C | -0.96167900 | -1.09643200 | 2.38778400 |
| H | -1.07608200 | -2.10117000 | 1.98752600 |
| H | -1.46454800 | -1.06234700 | 3.35583000 |
| H | 0.09897400 | -0.89703600 | 2.56353500 |
| C | 0.58785700 | 2.60127900 | -0.20461400 |

| | | | |
|---|---|---|---|
| C | 0.59562900 | -2.58998600 | -0.17922400 |
| C | 4.77759300 | 0.00359000 | -0.18589800 |
| F | 5.27035700 | 1.09360100 | -0.77660000 |
| F | 5.26381600 | -0.02428400 | 1.06104100 |
| F | 5.26492000 | -1.06363800 | -0.82170100 |
| F | 0.78880200 | 3.27453700 | 0.93973700 |
| F | 1.21162900 | 3.29700500 | -1.16333000 |
| F | 0.92704600 | -3.28590900 | 0.92098900 |
| F | -0.72319300 | -2.66218300 | -0.31020000 |
| F | 1.12738000 | -3.24722400 | -1.21626200 |
| F | -0.70855600 | 2.64834100 | -0.47547300 |
| C | -3.12730100 | -0.06987100 | -0.42949500 |
| C | -3.76384600 | 1.30147200 | -0.69973000 |
| C | -3.93656100 | -1.12627200 | -1.18367200 |
| C | -5.25366500 | 1.26692500 | -0.52449400 |
| H | -3.50090400 | 1.62036000 | -1.71484200 |
| H | -3.33247200 | 2.05344000 | -0.03186500 |
| C | -5.35141300 | -1.21685100 | -0.62642800 |
| H | -3.96422700 | -0.84139400 | -2.23913300 |
| H | -3.42995000 | -2.09363600 | -1.12078600 |
| C | -5.96309200 | 0.14304500 | -0.46907900 |
| H | -5.75973300 | 2.22249900 | -0.43104100 |
| H | -5.97517600 | -1.82825300 | -1.28374200 |
| H | -5.35060500 | -1.73428900 | 0.34141900 |
| H | -7.03582800 | 0.19720100 | -0.31399700 |

49

m-5-1 carbene-Au fragment

| | | | |
|---|---|---|---|
| Au | -0.95661000 | -1.15344300 | -1.63789300 |
| C | 1.54556700 | 1.36159900 | -0.17019000 |
| N | -0.57136400 | 0.51102900 | 0.77789600 |
| C | -1.12247200 | 1.09471100 | 2.04976000 |
| C | 2.91685900 | 1.22613300 | -0.35137700 |
| H | 3.46188100 | 1.99215400 | -0.88719300 |
| C | -1.45142000 | 0.13227400 | -0.12652700 |

| | | | |
|---|---|---|---|
| C | 1.49878900 | -0.77615500 | 0.93448500 |
| C | 3.58057800 | 0.11202400 | 0.12713100 |
| C | 0.82611100 | 0.38105400 | 0.52249000 |
| C | -2.58957600 | 0.66978700 | 1.91870900 |
| H | -3.26910500 | 1.43788400 | 2.29382000 |
| H | -2.74628000 | -0.23997400 | 2.50668700 |
| C | 2.86955400 | -0.89727300 | 0.74796500 |
| H | 3.37370700 | -1.79862300 | 1.07253100 |
| C | -0.92949600 | 2.60722200 | 2.05000100 |
| H | 0.13279400 | 2.86137000 | 2.05493300 |
| H | -1.37262700 | 3.01659800 | 2.95898200 |
| H | -1.39816600 | 3.08368600 | 1.19165100 |
| C | -0.45082300 | 0.53116100 | 3.29334100 |
| H | -0.62852500 | -0.53412000 | 3.41396100 |
| H | -0.86311200 | 1.04190900 | 4.16502200 |
| H | 0.62608800 | 0.71873000 | 3.28440900 |
| C | 0.96224600 | 2.61449500 | -0.79936400 |
| C | 0.86870000 | -2.00380700 | 1.57214700 |
| C | 5.07584400 | 0.00398200 | -0.02027300 |
| F | 5.50959800 | 0.64193000 | -1.10821800 |
| F | 5.70140800 | 0.54632200 | 1.03193600 |
| F | 5.47127900 | -1.26666100 | -0.10250200 |
| F | 1.24675100 | 3.69451900 | -0.05418100 |
| F | 1.52204000 | 2.82126300 | -1.99437500 |
| F | 1.29658700 | -2.12669300 | 2.84104300 |
| F | -0.46007000 | -2.03021200 | 1.60329500 |
| F | 1.27122700 | -3.09899300 | 0.92777300 |
| F | -0.35229100 | 2.59436200 | -0.98544700 |
| C | -2.82914900 | 0.35197700 | 0.42944200 |
| C | -3.47885200 | 1.50975000 | -0.34467100 |
| C | -3.70962700 | -0.88793000 | 0.24788300 |
| C | -4.94185400 | 1.62549600 | -0.03169900 |
| H | -3.32297300 | 1.34227700 | -1.41721000 |
| H | -2.97352400 | 2.45158000 | -0.11221800 |

| | | | |
|---|---|---|---|
| C | -5.06364900 | -0.67326100 | 0.91178500 |
| H | -3.83657700 | -1.06142700 | -0.82504500 |
| H | -3.20127800 | -1.76566300 | 0.65705400 |
| C | -5.64702400 | 0.65946100 | 0.54992100 |
| H | -5.42764400 | 2.55901000 | -0.29556100 |
| H | -5.75058200 | -1.46953400 | 0.61489900 |
| H | -4.97617700 | -0.75389700 | 2.00260000 |
| H | -6.69677100 | 0.82408900 | 0.76931200 |

62

m-6-1 carbene fragment

| | | | |
|---|---|---|---|
| C | -1.74932500 | -1.22870200 | -0.21308900 |
| N | 0.36357800 | -0.03720500 | 0.27869500 |
| C | 0.88503400 | -0.12226700 | 1.69533800 |
| C | -3.13320500 | -1.23017600 | -0.34466200 |
| H | -3.64707000 | -2.16547200 | -0.52464900 |
| C | 1.22904400 | -0.03337200 | -0.71346500 |
| C | -1.79153600 | 1.15486900 | 0.03180900 |
| C | -3.85071200 | -0.05326300 | -0.26770300 |
| C | -1.04834400 | -0.03557500 | 0.02799800 |
| C | 2.37569800 | 0.11936300 | 1.42777500 |
| H | 2.99594300 | -0.49187600 | 2.08696300 |
| H | 2.59984500 | 1.16730900 | 1.63633300 |
| C | -3.17445300 | 1.13873300 | -0.10511000 |
| H | -3.71832000 | 2.07476200 | -0.09686000 |
| C | 0.57314200 | -1.48465200 | 2.30503400 |
| H | -0.50654700 | -1.63100100 | 2.39232600 |
| H | 0.99015000 | -1.52343300 | 3.31271900 |
| H | 0.99036900 | -2.30791800 | 1.73011200 |
| C | 0.28382300 | 0.94366200 | 2.60030300 |
| H | 0.52312200 | 1.94925500 | 2.26162700 |
| H | 0.70071700 | 0.81879400 | 3.60138300 |
| H | -0.80118900 | 0.83855200 | 2.68426100 |
| C | -1.15489000 | -2.61687800 | -0.37746200 |
| C | -1.24565500 | 2.56538400 | 0.14256100 |

| | | | |
|---|---|---|---|
| C | -5.35363900 | -0.06997000 | -0.35061200 |
| F | -5.79427800 | -1.09225700 | -1.08582900 |
| F | -5.90253900 | -0.19110400 | 0.86419800 |
| F | -5.82814800 | 1.05612100 | -0.88643500 |
| F | -1.43261700 | -3.37548700 | 0.69577200 |
| F | -1.72618300 | -3.22537600 | -1.42479900 |
| F | -1.62430100 | 3.12415600 | 1.30319500 |
| F | 0.07079800 | 2.68056000 | 0.05431200 |
| F | -1.76848900 | 3.32405500 | -0.82876600 |
| F | 0.15232500 | -2.66440600 | -0.57320600 |
| C | 2.59864000 | -0.17491200 | -0.06693400 |
| C | 3.02464500 | -1.63867200 | -0.31082700 |
| C | 3.62230100 | 0.72836600 | -0.78080600 |
| C | 4.45415000 | -1.94484500 | 0.12629400 |
| H | 2.93041300 | -1.84591600 | -1.38462600 |
| H | 2.34446000 | -2.32284300 | 0.19850900 |
| C | 5.04437300 | 0.44602300 | -0.30884200 |
| H | 3.56366900 | 0.40396400 | -1.83003400 |
| C | 5.42556200 | -1.00234200 | -0.56697300 |
| H | 4.53545700 | -1.77356700 | 1.20858900 |
| H | 5.73890100 | 1.12032500 | -0.81955400 |
| H | 5.13014700 | 0.66203800 | 0.76435600 |
| H | 6.44823300 | -1.20618100 | -0.23617900 |
| H | 5.39089600 | -1.20048000 | -1.64673100 |
| C | 4.78989600 | -3.40025500 | -0.16254400 |
| H | 5.80671400 | -3.64176800 | 0.15390800 |
| H | 4.10275600 | -4.07913900 | 0.34691700 |
| H | 4.71722400 | -3.59429900 | -1.23653300 |
| C | 3.26667900 | 2.23148900 | -0.77168500 |
| H | 2.19257800 | 2.30826600 | -0.58649200 |
| C | 3.99594000 | 3.07363800 | 0.27598300 |
| H | 3.90884600 | 2.68492000 | 1.29153700 |
| H | 5.06168000 | 3.14265500 | 0.04303400 |
| H | 3.59653800 | 4.08987600 | 0.27353000 |

| | | | |
|---|---|---|---|
| C | 3.54426600 | 2.82443600 | -2.14974600 |
| H | 4.59830500 | 2.68265200 | -2.41194500 |
| H | 2.93576000 | 2.33736300 | -2.91349000 |
| H | 3.33763500 | 3.89673400 | -2.17166600 |

63

m-6-1 carbene-Au fragment

| | | | |
|---|---|---|---|
| Au | -0.63106100 | -0.75781900 | -1.82456700 |
| C | 1.97529600 | 1.36308100 | 0.03915600 |
| N | -0.12636600 | 0.34857400 | 0.86926600 |
| C | -0.62252600 | 0.70124900 | 2.24556600 |
| C | 3.34566600 | 1.28452800 | -0.17614200 |
| H | 3.87072300 | 2.14376000 | -0.57246300 |
| C | -1.04772500 | 0.19836000 | -0.06156500 |
| C | 1.97553100 | -0.92931400 | 0.75427800 |
| C | 4.03600000 | 0.11833100 | 0.09225000 |
| C | 1.26999300 | 0.26584900 | 0.55694300 |
| C | -2.12203600 | 0.42337500 | 2.06456700 |
| H | -2.72343800 | 1.14207600 | 2.62413600 |
| H | -2.33527200 | -0.57208800 | 2.45615500 |
| C | 3.34648500 | -0.99180800 | 0.53441200 |
| H | 3.86657900 | -1.92726500 | 0.69547600 |
| C | -0.29516700 | 2.15693600 | 2.56202500 |
| H | 0.78635000 | 2.30705400 | 2.60301800 |
| H | -0.69909700 | 2.40018300 | 3.54581100 |
| H | -0.71489900 | 2.85018000 | 1.83713300 |
| C | -0.00036100 | -0.16330600 | 3.33257400 |
| H | -0.26248900 | -1.21284400 | 3.22656600 |
| H | -0.37463700 | 0.18216900 | 4.29774300 |
| H | 1.08791600 | -0.06219900 | 3.35003000 |
| C | 1.39266700 | 2.71037300 | -0.34903400 |
| C | 1.39671800 | -2.26590500 | 1.18008400 |
| C | 5.53050100 | 0.07071800 | -0.08866600 |
| F | 5.92505500 | 0.83879700 | -1.10529800 |
| F | 6.15971000 | 0.51282900 | 1.00720600 |

| | | | |
|---|---|---|---|
| F | 5.95975400 | -1.17012600 | -0.31848100 |
| F | 1.69492300 | 3.63765100 | 0.57411800 |
| F | 1.93925100 | 3.12579200 | -1.49509300 |
| F | 1.84039100 | -2.58861000 | 2.40730700 |
| F | 0.07262300 | -2.33638700 | 1.20844500 |
| F | 1.82558800 | -3.22402600 | 0.35716500 |
| F | 0.07804100 | 2.73144400 | -0.51872900 |
| C | -2.40577700 | 0.44927700 | 0.55065500 |
| C | -2.82981400 | 1.84906400 | 0.04789900 |
| C | -3.46178700 | -0.56154300 | 0.05498600 |
| C | -4.23388300 | 2.25828200 | 0.47996100 |
| H | -2.78582500 | 1.83716800 | -1.04938200 |
| H | -2.11644600 | 2.60467700 | 0.37965300 |
| C | -4.85752900 | -0.16038900 | 0.52210400 |
| H | -3.44124600 | -0.44664900 | -1.03977300 |
| C | -5.24008000 | 1.21877700 | 0.01272500 |
| H | -4.26992400 | 2.29344600 | 1.57753300 |
| H | -5.57817800 | -0.90833500 | 0.17905800 |
| H | -4.89648400 | -0.16967500 | 1.61949200 |
| H | -6.24622500 | 1.49410200 | 0.34135400 |
| H | -5.24946700 | 1.21260800 | -1.08521600 |
| C | -4.56493600 | 3.64025100 | -0.06318900 |
| H | -5.56537900 | 3.95343900 | 0.24158000 |
| H | -3.85125900 | 4.39014100 | 0.28421200 |
| H | -4.53459000 | 3.62952300 | -1.15630700 |
| C | -3.14792600 | -2.04833100 | 0.33922700 |
| H | -2.06432700 | -2.14261900 | 0.45223600 |
| C | -3.81904600 | -2.63904400 | 1.58085800 |
| H | -3.65388100 | -2.06640400 | 2.49452100 |
| H | -4.89961500 | -2.71567200 | 1.43550600 |
| H | -3.44306900 | -3.64970400 | 1.75055300 |
| C | -3.55751800 | -2.88330200 | -0.87074100 |
| H | -4.62224100 | -2.73927500 | -1.08378800 |
| H | -2.98500200 | -2.59180500 | -1.75484500 |

| | | | |
|---|---|---|---|
| H | -3.39470300 | -3.94765200 | -0.68943200 |

38

n-1-1 carbene fragment

| | | | |
|---|---|---|---|
| C | 0.61378100 | 1.18654100 | -0.02858400 |
| N | -1.39389700 | -0.16451300 | -0.14261900 |
| C | -2.13608900 | -0.69733900 | 1.05303400 |
| C | 1.99310400 | 1.28566900 | -0.03507200 |
| C | -2.07770400 | 0.40072400 | -1.11205200 |
| C | 0.82908000 | -1.20177100 | -0.23267000 |
| C | 2.82483200 | 0.18550300 | -0.13730400 |
| C | 0.03097000 | -0.07165500 | -0.12509900 |
| C | -3.56789500 | -0.66006700 | 0.51253500 |
| H | -4.26917400 | -0.31308100 | 1.27476700 |
| H | -3.87041600 | -1.66871700 | 0.21748300 |
| C | 2.20606700 | -1.04505600 | -0.23420300 |
| C | -1.90245000 | 0.24538500 | 2.22823700 |
| H | -0.84366000 | 0.27285400 | 2.49863700 |
| H | -2.46141300 | -0.10341800 | 3.09797500 |
| H | -2.22622600 | 1.25983600 | 1.99177600 |
| C | -1.70180400 | -2.10517600 | 1.43067000 |
| H | -1.76158700 | -2.77842500 | 0.57336200 |
| H | -2.37238300 | -2.48258400 | 2.20476300 |
| H | -0.68755500 | -2.12534900 | 1.83502500 |
| C | -3.53389900 | 0.25860100 | -0.72637000 |
| C | -4.03471500 | 1.67348400 | -0.41745600 |
| H | -3.92815900 | 2.31063000 | -1.29597600 |
| H | -5.08984100 | 1.63511100 | -0.13456200 |
| H | -3.47853900 | 2.13408600 | 0.40184700 |
| C | -4.33610500 | -0.31187700 | -1.89061800 |
| H | -5.38942700 | -0.40520700 | -1.61248200 |
| H | -4.25914900 | 0.34330500 | -2.75910600 |
| H | -3.96788900 | -1.29987800 | -2.17474800 |
| H | -0.00371600 | 2.07225400 | 0.03284100 |
| H | 3.89997100 | 0.28403300 | -0.14402400 |

| | | | |
|---|---|---|---|
| H | 0.40433400 | -2.18866400 | -0.34266600 |
| N | 2.60625700 | 2.62114800 | 0.07580200 |
| O | 3.81767500 | 2.68023500 | 0.03753300 |
| O | 1.85684800 | 3.56759700 | 0.20145700 |
| N | 3.04826700 | -2.24813800 | -0.35342800 |
| O | 4.25003100 | -2.08579500 | -0.37739200 |
| O | 2.48012000 | -3.32004500 | -0.41578500 |

39

n-1-1 carbene-Au fragment

| | | | |
|---|---|---|---|
| Au | 1.43738700 | -1.41855400 | -0.79120200 |
| C | -1.21053500 | -0.36415400 | 1.06123800 |
| N | 0.58923000 | 1.13260000 | 0.40828600 |
| C | 0.98129500 | 2.18103200 | 1.39621000 |
| C | -2.52630000 | -0.77253800 | 0.92305900 |
| C | 1.57713100 | 0.53385900 | -0.23731000 |
| C | -1.60579800 | 1.31115600 | -0.62977900 |
| C | -3.40333200 | -0.18643000 | 0.02903400 |
| C | -0.76158700 | 0.69514900 | 0.27956600 |
| C | 2.43357300 | 2.42582500 | 0.96776900 |
| H | 3.07585900 | 2.61590300 | 1.82953600 |
| H | 2.47061700 | 3.30629400 | 0.32049000 |
| C | -2.91174300 | 0.85615200 | -0.73219600 |
| C | 0.84962800 | 1.62730100 | 2.81057500 |
| H | -0.19467500 | 1.40931400 | 3.04594300 |
| H | 1.20694600 | 2.36703500 | 3.52871800 |
| H | 1.43190300 | 0.71330500 | 2.93392600 |
| C | 0.11302000 | 3.41858600 | 1.23790800 |
| H | 0.14056300 | 3.78546300 | 0.21009900 |
| H | 0.48832100 | 4.20589500 | 1.89348900 |
| H | -0.92408800 | 3.21688000 | 1.51487800 |
| C | 2.87974000 | 1.18208300 | 0.17193700 |
| C | 3.69771500 | 0.20744400 | 1.02587900 |
| H | 3.97795300 | -0.66754600 | 0.43762100 |
| H | 4.60579000 | 0.70933500 | 1.36958400 |

| | | | |
|---|---|---|---|
| H | 3.14193800 | -0.14386700 | 1.89652900 |
| C | 3.68708300 | 1.54694900 | -1.07089900 |
| H | 4.61899100 | 2.03856100 | -0.78092400 |
| H | 3.92786200 | 0.64454900 | -1.63617800 |
| H | 3.12673500 | 2.22111700 | -1.72134600 |
| H | -0.54933800 | -0.86674600 | 1.75452600 |
| H | -4.42360800 | -0.52693700 | -0.06794400 |
| H | -1.26107700 | 2.12081100 | -1.25758500 |
| N | -3.01186000 | -1.87965000 | 1.76206800 |
| O | -4.14812200 | -2.26234400 | 1.57351100 |
| O | -2.24219400 | -2.32775600 | 2.58708500 |
| N | -3.81309900 | 1.51622700 | -1.68974400 |
| O | -4.94188200 | 1.08156500 | -1.78266000 |
| O | -3.36323800 | 2.45324700 | -2.31900700 |

36

o-1-1 carbene fragment

| | | | |
|---|---|---|---|
| C | 1.03745000 | 1.21702600 | -0.02147900 |
| N | -0.96613700 | -0.13678300 | -0.15682000 |
| C | -1.68578900 | -0.68330300 | 1.04625300 |
| C | 2.42448000 | 1.34940100 | -0.03523100 |
| C | -1.66919000 | 0.42933500 | -1.11144400 |
| C | 1.25839100 | -1.15873200 | -0.32131600 |
| C | 3.23872600 | 0.22946800 | -0.18918800 |
| C | 0.45966900 | -0.03749200 | -0.15863800 |
| C | -3.12654800 | -0.65179900 | 0.53003500 |
| H | -3.81750900 | -0.31462700 | 1.30601900 |
| H | -3.42766000 | -1.66029500 | 0.23291600 |
| C | 2.64797900 | -1.02141000 | -0.33544100 |
| C | -1.44007000 | 0.25015400 | 2.22644100 |
| H | -0.37743900 | 0.28285400 | 2.48027400 |
| H | -1.98327100 | -0.11034100 | 3.10142300 |
| H | -1.77516300 | 1.26422600 | 2.00417600 |
| C | -1.23455400 | -2.09088000 | 1.40428200 |
| H | -1.31097500 | -2.75809800 | 0.54347700 |

| | | | |
|---|---|---|---|
| H | -1.88401900 | -2.47829300 | 2.19134900 |
| H | -0.21054700 | -2.10600200 | 1.78337700 |
| C | -3.11840500 | 0.27549300 | -0.70278700 |
| C | -3.62332300 | 1.68464700 | -0.37588100 |
| H | -3.53419100 | 2.32881200 | -1.25127300 |
| H | -4.67368400 | 1.63814100 | -0.07685600 |
| H | -3.05717800 | 2.14247200 | 0.43825500 |
| C | -3.93566800 | -0.29219500 | -1.85783900 |
| H | -4.98379400 | -0.39479800 | -1.56365700 |
| H | -3.87696800 | 0.36946100 | -2.72286100 |
| H | -3.56530900 | -1.27560900 | -2.15491400 |
| H | 4.31496500 | 0.33179200 | -0.20101700 |
| H | 0.39686100 | 2.08412700 | 0.07720500 |
| H | 0.81123200 | -2.13280200 | -0.46398800 |
| C | 3.01928000 | 2.64796100 | 0.11306500 |
| C | 3.47184400 | -2.18460900 | -0.50898700 |
| N | 3.49664400 | 3.68881500 | 0.23813500 |
| N | 4.12792500 | -3.12194700 | -0.64345500 |

37

o-1-1 carbene-Au fragment

| | | | |
|---|---|---|---|
| Au | 1.19330800 | -1.55002500 | -0.39973300 |
| C | -1.56396700 | -0.17364200 | 1.09092900 |
| N | 0.21319900 | 1.18199900 | 0.13778600 |
| C | 0.54689800 | 2.44800600 | 0.85559000 |
| C | -2.87190400 | -0.65651000 | 1.05339300 |
| C | 1.23717100 | 0.48365300 | -0.32827700 |
| C | -1.95879700 | 1.04277900 | -0.95216500 |
| C | -3.72689700 | -0.29652600 | 0.01434100 |
| C | -1.12268400 | 0.68341800 | 0.09113500 |
| C | 2.00028600 | 2.63985700 | 0.40565800 |
| H | 2.61414800 | 3.05355800 | 1.20780000 |
| H | 2.02433300 | 3.34240500 | -0.43192100 |
| C | -3.26536400 | 0.55133300 | -0.98817800 |
| C | 0.39809500 | 2.24025300 | 2.35869500 |

| | | | |
|---|---|---|---|
| H | -0.64458600 | 2.04659400 | 2.62094200 |
| H | 0.71415800 | 3.14128500 | 2.88681500 |
| H | 1.00588300 | 1.40326100 | 2.70486000 |
| C | -0.35577800 | 3.58168600 | 0.39640200 |
| H | -0.31108900 | 3.70016400 | -0.68796900 |
| H | -0.02301700 | 4.51331200 | 0.85673900 |
| H | -1.39312500 | 3.41157800 | 0.69276800 |
| C | 2.50688600 | 1.26081300 | -0.06326100 |
| C | 3.33798900 | 0.55178700 | 1.01128300 |
| H | 3.66373700 | -0.42607100 | 0.65387100 |
| H | 4.21853200 | 1.15798000 | 1.23935600 |
| H | 2.77357300 | 0.39577700 | 1.93183300 |
| C | 3.33163200 | 1.35042300 | -1.34444500 |
| H | 4.23937800 | 1.93219200 | -1.16587500 |
| H | 3.61578200 | 0.34923200 | -1.67426100 |
| H | 2.76486600 | 1.82859600 | -2.14558500 |
| H | -4.73946600 | -0.67474300 | -0.01430900 |
| H | -0.88597100 | -0.47197000 | 1.88068200 |
| H | -1.59469100 | 1.69170400 | -1.73779800 |
| C | -3.34140600 | -1.52907100 | 2.09211800 |
| C | -4.13695200 | 0.92889100 | -2.06435500 |
| N | -3.72320400 | -2.22019200 | 2.93077400 |
| N | -4.83356700 | 1.24022700 | -2.92744600 |

42

p-1-1 carbene fragment

| | | | |
|---|---|---|---|
| C | 0.00679500 | 1.14244000 | -0.01454800 |
| N | -1.98767200 | -0.23401800 | -0.12057200 |
| C | -2.74485100 | -0.64694200 | 1.11224500 |
| C | 1.38834900 | 1.26669300 | 0.02361300 |
| C | -2.65846900 | 0.19917600 | -1.16309300 |
| C | 0.24551500 | -1.24828100 | -0.11027700 |
| C | 2.22096300 | 0.15631900 | 0.00434000 |
| C | -0.56442800 | -0.12195200 | -0.08017900 |
| C | -4.16538600 | -0.69836500 | 0.54408100 |

| | | | |
|---|---|---|---|
| H | -4.88836600 | -0.28045600 | 1.24828600 |
| H | -4.44478800 | -1.73947300 | 0.35967400 |
| C | 1.62587200 | -1.09378700 | -0.07123300 |
| C | -2.55443200 | 0.42110500 | 2.18341400 |
| H | -1.50326400 | 0.50051800 | 2.47166300 |
| H | -3.12661600 | 0.15516700 | 3.07365100 |
| H | -2.89200700 | 1.39757400 | 1.83326500 |
| C | -2.28693400 | -1.99618800 | 1.64460300 |
| H | -2.33006000 | -2.76050500 | 0.86622000 |
| H | -2.95380700 | -2.29914000 | 2.45385000 |
| H | -1.27360000 | -1.95404800 | 2.04961000 |
| C | -4.12087700 | 0.07504700 | -0.79017100 |
| C | -4.65035200 | 1.50606300 | -0.65209500 |
| H | -4.53515400 | 2.04278800 | -1.59444800 |
| H | -5.71053500 | 1.48146200 | -0.38680700 |
| H | -4.11814700 | 2.06480800 | 0.12071100 |
| C | -4.89000900 | -0.63785400 | -1.89657400 |
| H | -5.94709900 | -0.71952000 | -1.62894700 |
| H | -4.80664300 | -0.08319200 | -2.83206000 |
| H | -4.49942900 | -1.64440200 | -2.06059200 |
| H | -0.63073300 | 2.01809800 | 0.01081500 |
| H | 3.29590700 | 0.26195200 | 0.07644500 |
| H | -0.18597100 | -2.23893800 | -0.16781000 |
| S | 2.08989000 | 2.87287600 | 0.13978900 |
| O | 1.22310800 | 3.67835200 | 0.91622200 |
| O | 3.47642800 | 2.73608300 | 0.42841400 |
| S | 2.63091600 | -2.53325500 | -0.09230900 |
| O | 3.95535400 | -2.16516800 | 0.27376100 |
| O | 1.92027400 | -3.57460900 | 0.55205100 |
| O | 2.65568600 | -2.91511700 | -1.62503900 |
| H | 3.45291500 | -2.55297000 | -2.04264200 |
| O | 1.96619000 | 3.41126800 | -1.33908000 |
| H | 2.79134700 | 3.24714900 | -1.82196200 |

43

p-1-1 carbene-Au fragment

| | | | |
|---|---|---|---|
| Au | -1.62899500 | 1.09236000 | -1.11663100 |
| C | 0.47970800 | 0.47985500 | 1.04633800 |
| N | -1.08353500 | -1.29514700 | 0.60289400 |
| C | -1.58863300 | -2.31207300 | 1.57909500 |
| C | 1.71591000 | 1.07102500 | 0.85374500 |
| C | -1.98569300 | -0.69530000 | -0.13216400 |
| C | 1.30305600 | -1.56195900 | 0.06612900 |
| C | 2.77282300 | 0.35765200 | 0.30422400 |
| C | 0.26757700 | -0.82144900 | 0.60650100 |
| C | -3.02489400 | -2.49542000 | 1.07051800 |
| H | -3.72767200 | -2.59054400 | 1.90005100 |
| H | -3.08314400 | -3.41047700 | 0.47559300 |
| C | 2.55331300 | -0.96288200 | -0.05644400 |
| C | -1.50424900 | -1.71364000 | 2.97807300 |
| H | -0.46757000 | -1.48724900 | 3.23798300 |
| H | -1.88615900 | -2.43069900 | 3.70587600 |
| H | -2.08985200 | -0.79649000 | 3.05414400 |
| C | -0.77789000 | -3.59534500 | 1.50960200 |
| H | -0.74991700 | -3.98783900 | 0.49148900 |
| H | -1.24861000 | -4.34278200 | 2.15001700 |
| H | 0.24334600 | -3.44648900 | 1.86539100 |
| C | -3.33777200 | -1.27686700 | 0.17819000 |
| C | -4.14650500 | -0.19750500 | 0.90747600 |
| H | -4.28995700 | 0.66844500 | 0.25898500 |
| H | -5.12349400 | -0.60417400 | 1.17796200 |
| H | -3.64944600 | 0.14334200 | 1.81749500 |
| C | -4.05812200 | -1.65622600 | -1.11179300 |
| H | -5.03662900 | -2.08216200 | -0.87874100 |
| H | -4.19942000 | -0.77104900 | -1.73500600 |
| H | -3.48749300 | -2.39130000 | -1.68228200 |
| H | -0.34196100 | 1.03935700 | 1.47685500 |
| H | 3.73721200 | 0.82521900 | 0.14947700 |
| H | 1.15003700 | -2.57122100 | -0.29293000 |

| | | | |
|---|---|---|---|
| S | 1.90701100 | 2.79093600 | 1.19271600 |
| O | 2.07074000 | 3.00250800 | 2.58373700 |
| O | 2.85499600 | 3.28318100 | 0.25564100 |
| S | 3.86332100 | -1.89999100 | -0.75738700 |
| O | 5.09360500 | -1.27020600 | -0.42565700 |
| O | 3.60676500 | -3.26814100 | -0.49616400 |
| O | 3.63379000 | -1.71183500 | -2.30692400 |
| H | 4.15000700 | -0.95773800 | -2.63413400 |
| O | 0.47469000 | 3.32487800 | 0.85729500 |
| H | 0.16230700 | 3.10605900 | -0.05022200 |

40

q-1-1 carbene fragment

| | | | |
|---|---|---|---|
| C | -0.31950200 | 1.12812800 | -0.14253000 |
| N | -2.38023400 | -0.17123700 | -0.09623300 |
| C | -3.11220100 | 0.04806600 | 1.19852200 |
| C | 1.07694400 | 1.18814500 | -0.11892500 |
| C | -3.06318300 | -0.45508600 | -1.17889500 |
| C | -0.21122100 | -1.27694200 | -0.08655200 |
| C | 1.81562900 | 0.02452900 | -0.08428300 |
| C | -0.95158200 | -0.09474500 | -0.11183400 |
| C | -4.52569100 | -0.36863500 | 0.77953400 |
| H | -5.27885700 | 0.31337900 | 1.18076500 |
| H | -4.74086100 | -1.36743300 | 1.16985900 |
| C | 1.16725700 | -1.21545400 | -0.07645100 |
| C | -3.01974400 | 1.50544100 | 1.63664200 |
| H | -1.99335900 | 1.77819500 | 1.89372100 |
| H | -3.63420700 | 1.65215400 | 2.52672000 |
| H | -3.37871100 | 2.17855500 | 0.85640000 |
| C | -2.53265700 | -0.84997200 | 2.28219400 |
| H | -2.53329700 | -1.89376500 | 1.96090200 |
| H | -3.14037200 | -0.76714000 | 3.18504700 |
| H | -1.50992000 | -0.56044800 | 2.53484400 |
| C | -4.52013800 | -0.40294200 | -0.76258500 |
| C | -5.09445600 | 0.87998800 | -1.37096300 |

| | | | |
|---|---|---|---|
| H | -4.98832700 | 0.86359900 | -2.45643000 |
| H | -6.15557700 | 0.96373900 | -1.12164500 |
| H | -4.58240500 | 1.76879700 | -0.99582500 |
| C | -5.27619200 | -1.60435000 | -1.31608200 |
| H | -6.32619400 | -1.56507300 | -1.01251300 |
| H | -5.22547000 | -1.61234900 | -2.40568200 |
| H | -4.84840800 | -2.53983300 | -0.94897500 |
| C | 1.70858700 | 2.56572800 | -0.12764900 |
| C | 2.04365200 | -2.45247300 | -0.04815700 |
| H | 2.89647300 | 0.05725600 | -0.06670600 |
| H | -0.90427200 | 2.03779300 | -0.20019600 |
| H | -0.73926200 | -2.22011500 | -0.08866900 |
| Cl | 1.12037100 | 3.48192200 | 1.27638600 |
| Cl | 1.22182800 | 3.42855800 | -1.59521000 |
| Cl | 3.46385500 | 2.52759200 | -0.06118000 |
| Cl | 3.05746500 | -2.41858400 | 1.40738000 |
| Cl | 1.12591100 | -3.95230400 | -0.04299400 |
| Cl | 3.10654900 | -2.45572700 | -1.46496400 |

41

q-1-1 carbene-Au fragment

| | | | |
|---|---|---|---|
| Au | -2.36148000 | 0.59006100 | -1.40828500 |
| C | 0.27770200 | 0.76876600 | 0.71106700 |
| N | -1.36292600 | -0.99886400 | 0.89179800 |
| C | -1.72401800 | -1.45945800 | 2.26610100 |
| C | 1.57456100 | 1.20260000 | 0.43028400 |
| C | -2.36475500 | -0.84648800 | 0.04472900 |
| C | 0.92094800 | -1.49498500 | 0.17874000 |
| C | 2.53015100 | 0.29158000 | 0.02852800 |
| C | -0.03384300 | -0.56877000 | 0.59144100 |
| C | -3.14276900 | -1.98289700 | 2.01577700 |
| H | -3.79972200 | -1.77385100 | 2.86217700 |
| H | -3.10471900 | -3.06738300 | 1.88021900 |
| C | 2.20325700 | -1.06166800 | -0.09979800 |
| C | -1.67315300 | -0.27409800 | 3.22436600 |

| | | | |
|---|---|---|---|
| H | -0.65506000 | 0.11457000 | 3.30565200 |
| H | -1.99425500 | -0.59147300 | 4.21789200 |
| H | -2.32561900 | 0.53306600 | 2.88862100 |
| C | -0.77850700 | -2.55075600 | 2.74117100 |
| H | -0.74994300 | -3.37863800 | 2.03007500 |
| H | -1.13039300 | -2.93437800 | 3.70029800 |
| H | 0.23467600 | -2.16826200 | 2.88136700 |
| C | -3.63609700 | -1.31627800 | 0.71570900 |
| C | -4.55234900 | -0.11871300 | 0.98763400 |
| H | -4.86497800 | 0.33889800 | 0.04795300 |
| H | -5.43790100 | -0.46219600 | 1.52885600 |
| H | -4.05755400 | 0.65152900 | 1.58122700 |
| C | -4.36544400 | -2.29639000 | -0.19918200 |
| H | -5.27477800 | -2.65775600 | 0.28779500 |
| H | -4.63945900 | -1.79976200 | -1.13207300 |
| H | -3.73522300 | -3.15506400 | -0.43879700 |
| C | 1.85355100 | 2.68496900 | 0.58088800 |
| C | 3.30648000 | -2.00045400 | -0.54747600 |
| H | 3.53810100 | 0.61508700 | -0.19373500 |
| H | -0.49372800 | 1.47246500 | 1.00297000 |
| H | 0.63990700 | -2.53288600 | 0.07195300 |
| Cl | 1.51388900 | 3.16687900 | 2.25750900 |
| Cl | 0.78051700 | 3.58991800 | -0.49044700 |
| Cl | 3.51716800 | 3.11788000 | 0.21234200 |
| Cl | 4.64327300 | -1.92994800 | 0.61641200 |
| Cl | 2.78016700 | -3.67481000 | -0.67580700 |
| Cl | 3.91638300 | -1.48688900 | -2.12767700 |

40

r-1-1 carbene fragment

| | | | |
|---|---|---|---|
| C | 0.25919900 | 1.15140400 | -0.06179900 |
| N | -1.75193900 | -0.20344500 | -0.12820500 |
| C | -2.48482500 | -0.57595600 | 1.13088800 |
| C | 1.64467300 | 1.26795700 | -0.05846500 |
| C | -2.44434900 | 0.19209900 | -1.17116400 |

| | | | |
|---|---|---|---|
| C | 0.47107500 | -1.24145800 | -0.18117100 |
| C | 2.44922200 | 0.14434300 | -0.11504200 |
| C | -0.32510500 | -0.10235800 | -0.11717900 |
| C | -3.91720900 | -0.64523900 | 0.59570700 |
| H | -4.62578900 | -0.20458900 | 1.30084800 |
| H | -4.20053100 | -1.69157900 | 0.45051800 |
| C | 1.84920800 | -1.10840000 | -0.18039000 |
| C | -2.27497100 | 0.52251200 | 2.16726300 |
| H | -1.21830300 | 0.61060300 | 2.43157700 |
| H | -2.83025300 | 0.28265800 | 3.07558400 |
| H | -2.62090400 | 1.48772000 | 1.79460100 |
| C | -2.01381100 | -1.90801900 | 1.69436800 |
| H | -2.07695500 | -2.69558700 | 0.94088000 |
| H | -2.65823600 | -2.18550200 | 2.53057600 |
| H | -0.98935000 | -1.85053700 | 2.06834700 |
| C | -3.89926100 | 0.08452200 | -0.76332100 |
| C | -4.42403700 | 1.51998900 | -0.66056200 |
| H | -4.32587700 | 2.02603100 | -1.62165100 |
| H | -5.47895600 | 1.50692000 | -0.37402600 |
| H | -3.87543700 | 2.10167700 | 0.08347900 |
| C | -4.69261900 | -0.66223200 | -1.82944800 |
| H | -5.74404700 | -0.73386300 | -1.53735500 |
| H | -4.62800500 | -0.13835500 | -2.78394400 |
| H | -4.30603600 | -1.67399800 | -1.96930100 |
| C | 2.23962200 | 2.64888600 | 0.00583900 |
| C | 2.74232900 | -2.31780200 | -0.24648000 |
| H | 3.52755700 | 0.23586700 | -0.11500600 |
| H | -0.37439300 | 2.03054100 | -0.03724000 |
| H | 0.01366300 | -2.21839900 | -0.25525100 |
| F | 1.88317000 | 3.38367000 | -1.05248700 |
| F | 1.81490800 | 3.30658400 | 1.09283000 |
| F | 3.57229900 | 2.62236000 | 0.04819400 |
| F | 2.05007400 | -3.45057900 | -0.38018700 |
| F | 3.59380500 | -2.24035500 | -1.27461000 |

| | | | |
|---|---|---|---|
| F | 3.48580700 | -2.43134600 | 0.86187500 |

41

r-1-1 carbene-Au fragment

| | | | |
|---|---|---|---|
| Au | 1.80197400 | -1.11501100 | -1.12628100 |
| C | -0.92649500 | -0.62108500 | 0.92840300 |
| N | 0.83573900 | 1.03328300 | 0.66951300 |
| C | 1.19099800 | 1.83006400 | 1.88154500 |
| C | -2.23494400 | -1.03333700 | 0.70593500 |
| C | 1.84399600 | 0.64030500 | -0.09013100 |
| C | -1.37534600 | 1.40879200 | -0.28962200 |
| C | -3.11598700 | -0.24073100 | -0.00950500 |
| C | -0.50641100 | 0.60491400 | 0.43604400 |
| C | 2.63487100 | 2.21745200 | 1.54074100 |
| H | 3.26814100 | 2.21672400 | 2.42987200 |
| H | 2.64407900 | 3.22795700 | 1.12282300 |
| C | -2.67527500 | 0.97969100 | -0.50691900 |
| C | 1.07316800 | 0.95272400 | 3.12291500 |
| H | 0.03679600 | 0.64683900 | 3.28306500 |
| H | 1.39735800 | 1.51417100 | 4.00072000 |
| H | 1.69042600 | 0.05776800 | 3.03506400 |
| C | 0.28332900 | 3.04218900 | 2.01598900 |
| H | 0.30412800 | 3.64674000 | 1.10717900 |
| H | 0.63172500 | 3.65890300 | 2.84608200 |
| H | -0.74779700 | 2.74824900 | 2.22330400 |
| C | 3.12305700 | 1.21449700 | 0.47639000 |
| C | 3.97070200 | 0.09109600 | 1.08302900 |
| H | 4.28209500 | -0.60894700 | 0.30644600 |
| H | 4.85951400 | 0.52549000 | 1.54815600 |
| H | 3.42318900 | -0.47562200 | 1.83769300 |
| C | 3.92185300 | 1.88857800 | -0.63552900 |
| H | 4.83768400 | 2.32435400 | -0.22826700 |
| H | 4.19075800 | 1.15368700 | -1.39687900 |
| H | 3.34217200 | 2.68131400 | -1.11233300 |
| C | -2.65854500 | -2.37118200 | 1.25163200 |

| | | | |
|---|---|---|---|
| C | -3.64558500 | 1.85880400 | -1.24941600 |
| H | -4.13113500 | -0.56963400 | -0.18687800 |
| H | -0.22725600 | -1.25385400 | 1.46311600 |
| H | -1.02794000 | 2.35125800 | -0.69230900 |
| F | -1.97183600 | -3.36791200 | 0.68970900 |
| F | -2.43673200 | -2.44173300 | 2.57130100 |
| F | -3.95561600 | -2.60747800 | 1.04943400 |
| F | -3.02038800 | 2.74473600 | -2.02784000 |
| F | -4.46619000 | 1.14360700 | -2.02208700 |
| F | -4.41595100 | 2.55443200 | -0.40121200 |

51

s-1-1 carbene fragment

| | | | |
|---|---|---|---|
| C | -0.04619200 | 1.18849000 | -0.14336000 |
| N | -2.10456400 | -0.12722200 | -0.07527200 |
| C | -2.80117000 | -0.18777000 | 1.26281400 |
| C | 1.35427900 | 1.21313000 | 0.00040700 |
| C | -2.83089700 | -0.10998900 | -1.16586800 |
| C | 0.05245600 | -1.25707900 | -0.19296500 |
| C | 2.11147800 | 0.04694600 | 0.18200300 |
| C | -0.67316900 | -0.05943100 | -0.12415600 |
| C | -4.22654300 | -0.50134200 | 0.80170900 |
| H | -4.96583500 | 0.05561400 | 1.38186700 |
| H | -4.42737100 | -1.56758500 | 0.94327100 |
| C | 1.44459900 | -1.17016600 | -0.04940500 |
| C | -2.68366500 | 1.15089200 | 1.98279400 |
| H | -1.63913100 | 1.38537400 | 2.20173500 |
| H | -3.21834300 | 1.09015100 | 2.93218000 |
| H | -3.11217000 | 1.96527400 | 1.39940700 |
| C | -2.21941700 | -1.27038200 | 2.16206600 |
| H | -2.32239700 | -2.26273400 | 1.72504100 |
| H | -2.76095600 | -1.26373200 | 3.10966600 |
| H | -1.16491900 | -1.08489100 | 2.38085200 |
| C | -0.86724600 | 2.43504400 | -0.32500600 |
| H | -0.91989900 | 3.00924000 | 0.59911300 |

| | | | |
|---|---|---|---|
| H | -0.42406800 | 3.08350600 | -1.07578800 |
| H | -1.87066500 | 2.18000100 | -0.65378600 |
| C | -0.67744900 | -2.55270400 | -0.41802000 |
| H | -0.42197100 | -2.95735700 | -1.39522700 |
| H | -0.40183500 | -3.30611400 | 0.31965300 |
| H | -1.75062400 | -2.39880400 | -0.38992700 |
| C | 3.53314400 | 0.07015100 | 0.68762900 |
| H | 3.64552700 | 0.85379900 | 1.43112900 |
| H | 3.78370000 | -0.87483900 | 1.15859300 |
| H | 4.25372800 | 0.26794700 | -0.10298900 |
| C | -4.27260200 | -0.16265500 | -0.70256100 |
| C | -4.87233400 | 1.21975600 | -0.97517200 |
| H | -4.80086900 | 1.45841700 | -2.03727000 |
| H | -5.92532500 | 1.23087800 | -0.68195700 |
| H | -4.35573400 | 2.00452300 | -0.41746700 |
| C | -5.03755000 | -1.21109500 | -1.50192100 |
| H | -6.07513800 | -1.26626500 | -1.16087300 |
| H | -5.02812400 | -0.95951000 | -2.56325100 |
| H | -4.58522800 | -2.19888600 | -1.38258500 |
| S | 2.13799000 | 2.80341200 | -0.04326800 |
| O | 1.47550800 | 3.67228800 | 0.85856600 |
| O | 3.55557200 | 2.68236200 | -0.03523400 |
| S | 2.40385900 | -2.66240000 | -0.09172900 |
| O | 2.80409000 | -2.99921300 | 1.22616400 |
| O | 1.77810200 | -3.62862200 | -0.93019200 |
| O | 3.70679300 | -2.21693900 | -0.87211500 |
| H | 3.62308000 | -2.50837000 | -1.79392900 |
| O | 1.76721900 | 3.30812700 | -1.50054600 |
| H | 2.50233700 | 3.10394100 | -2.09969000 |

52

s-1-1 carbene-Au fragment

| | | | |
|---|---|---|---|
| Au | -2.07238200 | 0.37414400 | -1.64015300 |
| C | 0.66457900 | 1.06962800 | 0.65492600 |
| N | -1.25265000 | -0.36898800 | 1.10715200 |

| | | | |
|---|---|---|---|
| C | -1.63515900 | -0.55073000 | 2.54719900 |
| C | 2.04713400 | 1.16587300 | 0.40992300 |
| C | -2.23057700 | -0.43509900 | 0.23134600 |
| C | 0.82284700 | -1.35425200 | 0.30860700 |
| C | 2.85832200 | 0.03453700 | 0.24042200 |
| C | 0.11293200 | -0.21220200 | 0.70335400 |
| C | -3.08023000 | -1.04069700 | 2.38314500 |
| H | -3.73062100 | -0.63771100 | 3.16181500 |
| H | -3.09866300 | -2.13201400 | 2.46118600 |
| C | 2.19360500 | -1.19369700 | 0.06588500 |
| C | -1.52683000 | 0.75740800 | 3.32230200 |
| H | -0.49647900 | 1.11866900 | 3.32939400 |
| H | -1.82435400 | 0.57393200 | 4.35610000 |
| H | -2.17014600 | 1.53575200 | 2.91454700 |
| C | -0.74081800 | -1.58140300 | 3.22334600 |
| H | -0.78192800 | -2.54891300 | 2.72465500 |
| H | -1.08302400 | -1.71629100 | 4.25063700 |
| H | 0.29702900 | -1.24278800 | 3.25718800 |
| C | -0.21528200 | 2.27434700 | 0.84020000 |
| H | 0.03688600 | 2.81351200 | 1.75270800 |
| H | -0.08736800 | 2.96446500 | 0.01006300 |
| H | -1.26160500 | 1.98507400 | 0.86117400 |
| C | 0.10306800 | -2.66623200 | 0.15581900 |
| H | 0.02475600 | -2.92253200 | -0.89997000 |
| H | 0.63621900 | -3.48064300 | 0.64430100 |
| H | -0.90332400 | -2.60968900 | 0.55790100 |
| C | 4.36321100 | 0.09317400 | 0.33026400 |
| H | 4.65714100 | 0.80559700 | 1.09574200 |
| H | 4.76437700 | -0.87750000 | 0.60386500 |
| H | 4.82388600 | 0.41153800 | -0.60220800 |
| C | -3.53146000 | -0.62594400 | 0.96934400 |
| C | -4.29401500 | 0.70343100 | 0.95585800 |
| H | -4.55368300 | 0.97631800 | -0.06823400 |
| H | -5.21115600 | 0.60120700 | 1.54110600 |

| | | | |
|---|---|---|---|
| H | -3.70197600 | 1.51860400 | 1.37678500 |
| C | -4.37302600 | -1.69785400 | 0.28615600 |
| H | -5.30388800 | -1.85498600 | 0.83655500 |
| H | -4.61341700 | -1.38904000 | -0.73315400 |
| H | -3.83394500 | -2.64692000 | 0.23815800 |
| S | 2.74149300 | 2.79736200 | 0.32659000 |
| O | 2.37068900 | 3.51779100 | 1.48889600 |
| O | 4.09476000 | 2.75289300 | -0.10827000 |
| S | 3.14408100 | -2.60736900 | -0.43291900 |
| O | 3.91759000 | -3.06403800 | 0.66403600 |
| O | 2.32641300 | -3.50797700 | -1.17280400 |
| O | 4.15203500 | -1.99418100 | -1.48525800 |
| H | 3.80275800 | -2.15676700 | -2.37619000 |
| O | 1.92633100 | 3.44098800 | -0.86638500 |
| H | 2.39768400 | 3.29126800 | -1.70116500 |

49

t-1-1 carbene fragment

| | | | |
|---|---|---|---|
| C | 0.17157400 | 1.19524400 | -0.12478700 |
| N | -1.89498200 | -0.10981400 | -0.06930100 |
| C | -2.63404600 | -0.14712900 | 1.24381700 |
| C | 1.57198900 | 1.22259700 | -0.09451300 |
| C | -2.58926600 | -0.07178400 | -1.18033500 |
| C | 0.26515700 | -1.25116500 | -0.12468600 |
| C | 2.33736800 | 0.05322700 | -0.16912800 |
| C | -0.45862400 | -0.05379900 | -0.09140400 |
| C | -4.06273800 | -0.38342900 | 0.74496900 |
| H | -4.78232900 | 0.22810400 | 1.29434100 |
| H | -4.33416600 | -1.43188400 | 0.89760600 |
| C | 1.66372300 | -1.17293100 | -0.10589500 |
| C | -2.47076700 | 1.17580800 | 1.98216200 |
| H | -1.42036200 | 1.36247900 | 2.21921600 |
| H | -3.02488700 | 1.13096200 | 2.92126700 |
| H | -2.85499000 | 2.01110300 | 1.39672400 |
| C | -2.13840800 | -1.27565400 | 2.13539800 |

| | | | |
|---|---|---|---|
| H | -2.29948900 | -2.24594300 | 1.66693600 |
| H | -2.69682400 | -1.25597400 | 3.07307900 |
| H | -1.07757900 | -1.16271400 | 2.37278300 |
| C | -0.64091700 | 2.44417600 | -0.35278300 |
| H | -0.82774800 | 2.99813500 | 0.56570400 |
| H | -0.11821000 | 3.11149700 | -1.03590300 |
| H | -1.59386500 | 2.18279200 | -0.80794800 |
| C | -0.45770700 | -2.55713900 | -0.33207000 |
| H | 0.07565200 | -3.16960400 | -1.05769500 |
| H | -0.53845100 | -3.13742300 | 0.58544700 |
| H | -1.45645400 | -2.37086300 | -0.72009000 |
| C | 3.81874500 | 0.11349900 | -0.46721800 |
| H | 4.42700600 | 0.13030500 | 0.43551300 |
| H | 4.11812200 | -0.74629500 | -1.06103200 |
| H | 4.04905300 | 1.00349800 | -1.04729000 |
| C | -4.04545400 | -0.06482500 | -0.76354500 |
| C | -4.57572200 | 1.33988100 | -1.06571000 |
| H | -4.45088100 | 1.57166700 | -2.12444500 |
| H | -5.63837600 | 1.39601600 | -0.81531200 |
| H | -4.04928100 | 2.10329100 | -0.48741100 |
| C | -4.82895100 | -1.08711200 | -1.57811600 |
| H | -5.87930400 | -1.09169500 | -1.27361700 |
| H | -4.77111400 | -0.84866600 | -2.64097500 |
| H | -4.42649400 | -2.09279900 | -1.43277000 |
| C | 2.48233100 | -2.44800100 | -0.05110300 |
| C | 2.28364700 | 2.55863100 | -0.01064200 |
| F | 3.43867000 | 2.47848800 | 0.66052200 |
| F | 1.56752400 | 3.48281000 | 0.63931900 |
| F | 3.62430400 | -2.28910300 | 0.62882400 |
| F | 1.84647100 | -3.44822200 | 0.56791900 |
| F | 2.81060500 | -2.89426100 | -1.27491500 |
| F | 2.56623200 | 3.06497100 | -1.22227800 |

50

t-1-1 carbene-Au fragment

| | | | |
|---|---|---|---|
| Au | -1.85751200 | -0.80512500 | -1.51766400 |
| C | 0.96287100 | 1.38236300 | 0.07760300 |
| N | -1.08990000 | 0.46698200 | 1.03482600 |
| C | -1.49714800 | 0.90076800 | 2.41022400 |
| C | 2.31512100 | 1.20610300 | -0.24132300 |
| C | -2.06186900 | 0.34814400 | 0.15284100 |
| C | 0.89474900 | -0.94051700 | 0.86285100 |
| C | 2.94836300 | -0.03589700 | -0.11567600 |
| C | 0.28917900 | 0.30659900 | 0.66570900 |
| C | -3.02080800 | 0.75103000 | 2.31849300 |
| H | -3.53216900 | 1.57025300 | 2.82823000 |
| H | -3.32136000 | -0.18298900 | 2.80086500 |
| C | 2.24457100 | -1.07044600 | 0.51363500 |
| C | -1.05357000 | 2.33947300 | 2.65047300 |
| H | 0.03423700 | 2.42318900 | 2.58945500 |
| H | -1.36120700 | 2.64421300 | 3.65192000 |
| H | -1.50047600 | 3.02365000 | 1.92978500 |
| C | -0.89453000 | 0.01290000 | 3.48792800 |
| H | -1.24376500 | -1.01438200 | 3.39630800 |
| H | -1.20128500 | 0.39071800 | 4.46477700 |
| H | 0.19747100 | 0.02668800 | 3.44904200 |
| C | 0.20823200 | 2.62237500 | -0.32822900 |
| H | 0.32926500 | 3.43650900 | 0.38499700 |
| H | 0.56283900 | 2.97768900 | -1.29402700 |
| H | -0.85144000 | 2.39891200 | -0.43137500 |
| C | 0.06385000 | -2.12155400 | 1.29318500 |
| H | 0.30623000 | -2.98829000 | 0.67969100 |
| H | 0.23844600 | -2.39332200 | 2.33305200 |
| H | -0.99401400 | -1.91022400 | 1.15366600 |
| C | 4.28498100 | -0.29269000 | -0.77373500 |
| H | 5.12322900 | -0.09863700 | -0.10686200 |
| H | 4.34775300 | -1.32569400 | -1.10559600 |
| H | 4.39731200 | 0.33746400 | -1.65232400 |
| C | -3.37266400 | 0.68404000 | 0.81948600 |

| | | | |
|---|---|---|---|
| C | -3.83114900 | 2.03357200 | 0.25574700 |
| H | -3.96615500 | 1.95872600 | -0.82486500 |
| H | -4.78349100 | 2.31629700 | 0.71088100 |
| H | -3.10750800 | 2.82681900 | 0.45465400 |
| C | -4.43911200 | -0.36669200 | 0.53149600 |
| H | -5.35353100 | -0.12047200 | 1.07781500 |
| H | -4.66239400 | -0.40310500 | -0.53601800 |
| H | -4.10329000 | -1.35976100 | 0.83797800 |
| C | 2.96709400 | -2.37780200 | 0.77883500 |
| C | 3.11100600 | 2.38455400 | -0.76828200 |
| F | 4.39956500 | 2.32683300 | -0.41339400 |
| F | 2.67086400 | 3.55430600 | -0.28990500 |
| F | 4.25802900 | -2.18681600 | 1.07887500 |
| F | 2.46428400 | -3.04871000 | 1.82004600 |
| F | 2.92375900 | -3.20469500 | -0.27619100 |
| F | 3.07272200 | 2.47061400 | -2.10698200 |

40

u-1-1 carbene fragment

| | | | |
|---|---|---|---|
| N | -0.35189800 | -0.16576200 | -0.14364100 |
| C | -0.95012400 | 1.04048800 | 0.52380500 |
| C | -1.17760400 | -1.08850900 | -0.57692400 |
| C | -2.38479900 | 0.54812300 | 0.74742200 |
| H | -3.11222800 | 1.34978600 | 0.59721600 |
| H | -2.48708100 | 0.19626900 | 1.77882800 |
| C | -0.92183600 | 2.26465300 | -0.39442000 |
| H | 0.07873400 | 2.68224000 | -0.49519500 |
| H | -1.56196600 | 3.03901500 | 0.03336800 |
| H | -1.29690000 | 2.02015400 | -1.38838200 |
| C | -0.28437300 | 1.38536600 | 1.84927400 |
| H | -0.26078600 | 0.51745000 | 2.51032300 |
| H | -0.86873500 | 2.16966700 | 2.33594200 |
| H | 0.73173100 | 1.76171500 | 1.71889300 |
| C | -2.57555100 | -0.63321700 | -0.21717200 |
| C | -3.28287100 | -0.23672100 | -1.51519700 |

| | | | |
|---|---|---|---|
| H | -3.30452200 | -1.08475200 | -2.20146400 |
| H | -4.31111500 | 0.06760500 | -1.30066100 |
| H | -2.77986300 | 0.58991200 | -2.01982100 |
| C | -3.34120000 | -1.78152800 | 0.43114400 |
| H | -4.34445400 | -1.45456000 | 0.72052000 |
| H | -3.43018300 | -2.61649600 | -0.26547600 |
| H | -2.82506000 | -2.13897800 | 1.32508900 |
| C | 1.09471900 | -0.38703000 | -0.33249000 |
| C | 1.88187600 | 0.80235100 | -0.86632600 |
| C | 1.76723600 | -0.97103500 | 0.90725000 |
| H | 1.11876100 | -1.16862800 | -1.09803400 |
| C | 3.29985000 | 0.35293400 | -1.20967800 |
| H | 1.93800200 | 1.59507200 | -0.11153600 |
| H | 1.38582400 | 1.21666000 | -1.74728200 |
| C | 3.18242800 | -1.41881400 | 0.55814500 |
| H | 1.81928200 | -0.22375400 | 1.70466500 |
| H | 1.16438400 | -1.80761300 | 1.26990100 |
| C | 3.99112900 | -0.25005600 | 0.00720400 |
| H | 3.87436900 | 1.19464000 | -1.60222100 |
| H | 3.25137400 | -0.39996100 | -2.00485600 |
| H | 3.67088500 | -1.84731400 | 1.43582800 |
| H | 3.13138300 | -2.21019900 | -0.19860800 |
| H | 5.00423800 | -0.56864600 | -0.24788100 |
| H | 4.08392400 | 0.51901000 | 0.78411100 |

41

u-1-1 carbene-Au fragment

| | | | |
|---|---|---|---|
| Au | -1.40607200 | -1.33194300 | -0.06358300 |
| N | 0.57594000 | 0.94811500 | -0.05112700 |
| C | 0.82663600 | 2.39889200 | 0.23170900 |
| C | -0.68353700 | 0.60677800 | -0.14511300 |
| C | -0.58967800 | 2.87899400 | 0.58305500 |
| H | -0.78487000 | 3.87433400 | 0.17864800 |
| H | -0.69099200 | 2.93407800 | 1.67062000 |
| C | 1.36731100 | 3.12492800 | -1.00069100 |

| | | | |
|---|---|---|---|
| H | 2.40361300 | 2.86688900 | -1.21023300 |
| H | 1.32373900 | 4.19978000 | -0.81565700 |
| H | 0.76733700 | 2.90511000 | -1.88426100 |
| C | 1.76993200 | 2.60307000 | 1.40903600 |
| H | 1.41469200 | 2.06394800 | 2.28846900 |
| H | 1.79675300 | 3.66856200 | 1.64728800 |
| H | 2.78858800 | 2.28514600 | 1.18390000 |
| C | -1.55796300 | 1.81842300 | 0.03925300 |
| C | -2.13888900 | 2.17470000 | -1.33263100 |
| H | -2.72516300 | 1.33657400 | -1.71416400 |
| H | -2.79083700 | 3.04652700 | -1.23828700 |
| H | -1.35894900 | 2.40363100 | -2.06062500 |
| C | -2.70290500 | 1.53962000 | 1.00698400 |
| H | -3.27165500 | 2.45829900 | 1.17507200 |
| H | -3.37040900 | 0.77783000 | 0.60061700 |
| H | -2.32752700 | 1.17917400 | 1.96682100 |
| C | 1.61743200 | -0.09245400 | -0.18174200 |
| C | 2.84191100 | 0.29280500 | -0.99844500 |
| C | 2.01103600 | -0.67593400 | 1.17212100 |
| H | 1.10555000 | -0.89455300 | -0.72554300 |
| C | 3.70048700 | -0.95317600 | -1.20818500 |
| H | 3.43731600 | 1.04865400 | -0.47411100 |
| H | 2.54001800 | 0.71227500 | -1.96078100 |
| C | 2.86529500 | -1.91844800 | 0.94986900 |
| H | 2.57872600 | 0.05600600 | 1.75546400 |
| C | 4.09971800 | -1.57725000 | 0.12358600 |
| H | 3.12718800 | -1.68392300 | -1.78966900 |
| H | 3.14923900 | -2.35599200 | 1.90870200 |
| H | 2.26311700 | -2.66696700 | 0.42137200 |
| H | 4.70972200 | -2.46752300 | -0.04262600 |
| H | 4.71991000 | -0.86488400 | 0.68192900 |
| H | 4.58428400 | -0.69922100 | -1.79683300 |
| H | 1.09799800 | -0.92272000 | 1.72197700 |

52

v-1-1 carbene fragment

| | | | |
|---|---|---|---|
| N | 0.73689000 | -0.39588600 | -0.22377100 |
| C | 1.32669400 | -0.96621500 | 1.03608000 |
| C | 1.57781500 | -0.00900500 | -1.15440700 |
| C | 2.76469500 | -0.45501700 | 0.91553900 |
| H | 3.48305600 | -1.17463400 | 1.31607100 |
| H | 2.86200400 | 0.47080200 | 1.49310600 |
| C | 1.27676100 | -2.49567900 | 1.00928700 |
| H | 0.26469600 | -2.87413700 | 1.14782700 |
| H | 1.89249800 | -2.88454700 | 1.82316800 |
| H | 1.66385000 | -2.88397300 | 0.06719600 |
| C | 0.67382600 | -0.46798100 | 2.31855000 |
| H | 0.64054500 | 0.62275700 | 2.35499600 |
| H | 1.27462900 | -0.81552200 | 3.16215400 |
| H | -0.33568700 | -0.86103800 | 2.44873300 |
| C | 2.97097500 | -0.14806600 | -0.57761000 |
| C | 3.72290000 | -1.24556100 | -1.33047100 |
| H | 3.73828000 | -1.01904700 | -2.39780500 |
| H | 4.75387900 | -1.30561600 | -0.97089500 |
| H | 3.26048600 | -2.22533400 | -1.20162300 |
| C | 3.69803800 | 1.17963200 | -0.77961500 |
| H | 4.68697200 | 1.14712900 | -0.31252200 |
| H | 3.81491800 | 1.39009600 | -1.84364600 |
| H | 3.13192200 | 2.00183100 | -0.33210700 |
| C | -0.70425700 | -0.22847700 | -0.46663400 |
| C | -1.53369400 | -1.48668800 | -0.24279800 |
| C | -1.32805300 | 0.96442500 | 0.27969600 |
| H | -0.73952500 | 0.00774100 | -1.53531200 |
| C | -2.95463900 | -1.29735900 | -0.76540900 |
| H | -1.59452100 | -1.72152400 | 0.82844100 |
| H | -1.06000400 | -2.33824800 | -0.74068700 |
| C | -2.73095800 | 1.16399500 | -0.29622400 |
| H | -1.45873400 | 0.68397900 | 1.33342900 |
| C | -3.58632300 | -0.08044800 | -0.10013500 |

| | | | |
|---|---|---|---|
| H | -2.89067400 | -1.09762400 | -1.84351100 |
| H | -3.20838700 | 2.03211900 | 0.16519800 |
| H | -2.65032500 | 1.37591000 | -1.36882900 |
| H | -4.59520200 | 0.07637500 | -0.49249900 |
| H | -3.69087200 | -0.28764000 | 0.97381900 |
| C | -3.78759700 | -2.54901500 | -0.53833100 |
| H | -3.86081300 | -2.76151200 | 0.53253000 |
| H | -4.80140800 | -2.42578700 | -0.92418200 |
| H | -3.34142600 | -3.41948300 | -1.02344500 |
| C | -0.46895800 | 2.23810400 | 0.24537400 |
| H | 0.55259900 | 1.94744200 | 0.52075500 |
| C | -0.94939200 | 3.24606600 | 1.28339000 |
| H | -0.29001400 | 4.11578300 | 1.31033200 |
| H | -1.95470500 | 3.60687900 | 1.05136500 |
| H | -0.97220300 | 2.80535000 | 2.28422000 |
| C | -0.40100600 | 2.87664200 | -1.13725000 |
| H | 0.31201300 | 3.70403300 | -1.13452400 |
| H | -0.07287300 | 2.15968600 | -1.89264400 |
| H | -1.37415800 | 3.28284500 | -1.42689500 |

53

v-1-1 carbene-Au fragment

| | | | |
|---|---|---|---|
| Au | -1.72786900 | -1.39555300 | 0.11164700 |
| N | 0.17968400 | 0.87086300 | -0.45429500 |
| C | 0.25155900 | 2.19625800 | -1.14872200 |
| C | -1.02518600 | 0.53728200 | -0.05420400 |
| C | -1.02916500 | 2.85370400 | -0.62201300 |
| H | -1.46859600 | 3.52627200 | -1.36144900 |
| H | -0.78192800 | 3.44349500 | 0.26696700 |
| C | 0.20030800 | 1.99533700 | -2.66440900 |
| H | 1.13608000 | 1.59081100 | -3.04846700 |
| H | 0.02730000 | 2.95967800 | -3.14635400 |
| H | -0.60615200 | 1.31673600 | -2.94270500 |
| C | 1.47466700 | 3.02512200 | -0.78676900 |
| H | 1.57335900 | 3.14655500 | 0.29349700 |

| | | | |
|---|---|---|---|
| H | 1.34932700 | 4.01615100 | -1.22839300 |
| H | 2.39576300 | 2.59892000 | -1.18606400 |
| C | -1.96751800 | 1.70234500 | -0.22499000 |
| C | -3.04478200 | 1.40439300 | -1.26894100 |
| H | -3.63287600 | 0.53796000 | -0.96123900 |
| H | -3.70810300 | 2.26852200 | -1.35904900 |
| H | -2.62301900 | 1.18876000 | -2.25104300 |
| C | -2.64306700 | 1.95499500 | 1.12313100 |
| H | -3.27589100 | 2.84460800 | 1.06508300 |
| H | -3.25940400 | 1.09683600 | 1.39864400 |
| H | -1.89782400 | 2.10720200 | 1.90895400 |
| C | 1.31295300 | -0.03492500 | -0.22072300 |
| C | 2.09322900 | -0.38304500 | -1.48060200 |
| C | 2.25416100 | 0.42862200 | 0.90276400 |
| H | 0.83039000 | -0.95838200 | 0.12397000 |
| C | 3.07783300 | -1.51465700 | -1.19911700 |
| H | 2.66224900 | 0.48655300 | -1.83617400 |
| H | 1.39927500 | -0.67664800 | -2.27439400 |
| C | 3.19543400 | -0.74149300 | 1.19403000 |
| H | 2.87790600 | 1.24639400 | 0.51815700 |
| C | 3.99391600 | -1.12193600 | -0.04585900 |
| H | 2.49465200 | -2.39101200 | -0.88672800 |
| H | 3.86600100 | -0.48808700 | 2.01892900 |
| H | 2.60254900 | -1.60550700 | 1.51604800 |
| H | 4.68340400 | -1.94134300 | 0.17528800 |
| H | 4.60692500 | -0.26728000 | -0.36397900 |
| C | 3.87325600 | -1.86227100 | -2.44723700 |
| H | 4.45980300 | -0.99759600 | -2.77235600 |
| H | 4.56657800 | -2.68374200 | -2.25770200 |
| H | 3.21749500 | -2.15307200 | -3.27005600 |
| C | 1.52001600 | 0.94905200 | 2.14831300 |
| H | 0.74887500 | 1.65043700 | 1.80311900 |
| C | 2.46891800 | 1.73070900 | 3.04985500 |
| H | 1.93216900 | 2.15331300 | 3.90124200 |

| | | | |
|---|---|---|---|
| H | 3.25602900 | 1.08567400 | 3.44819900 |
| H | 2.94771900 | 2.55108800 | 2.50766800 |
| C | 0.81832500 | -0.15382200 | 2.93338000 |
| H | 0.19392900 | 0.27941000 | 3.71750600 |
| H | 0.17257900 | -0.76620000 | 2.29764000 |
| H | 1.54815800 | -0.80785400 | 3.41788700 |

38

w-1-1 carbene fragment

| | | | |
|---|---|---|---|
| N | -0.27374200 | -0.12684000 | -0.15737400 |
| C | -0.91325700 | 1.06080800 | 0.50227700 |
| C | -1.06580400 | -1.08089200 | -0.58583600 |
| C | -2.32990100 | 0.52083600 | 0.73004100 |
| H | -3.08426900 | 1.29571200 | 0.57225200 |
| H | -2.42111400 | 0.17450300 | 1.76433500 |
| C | -0.93493400 | 2.27442800 | -0.42924000 |
| H | 0.05373000 | 2.70526400 | -0.57781800 |
| H | -1.56920400 | 3.04566900 | 0.01279300 |
| H | -1.34631500 | 2.01066800 | -1.40363300 |
| C | -0.25397800 | 1.43085600 | 1.82437100 |
| H | -0.18969800 | 0.56145700 | 2.48109400 |
| H | -0.86668500 | 2.18792500 | 2.31886900 |
| H | 0.74549700 | 1.84826500 | 1.69197200 |
| C | -2.47866700 | -0.67494800 | -0.22538800 |
| C | -3.21102900 | -0.32196900 | -1.52187700 |
| H | -3.20549500 | -1.17914500 | -2.19697400 |
| H | -4.24853300 | -0.05489500 | -1.30256200 |
| H | -2.74461900 | 0.51634800 | -2.04191800 |
| C | -3.19230400 | -1.84749100 | 0.43969600 |
| H | -4.20539000 | -1.55798500 | 0.73463800 |
| H | -3.25317100 | -2.69196000 | -0.24836400 |
| H | -2.65502200 | -2.17534000 | 1.33261000 |
| C | 1.17953400 | -0.31228300 | -0.31838500 |
| C | 1.95851200 | 0.89567500 | -0.81590800 |
| C | 1.85403600 | -0.87563900 | 0.92962600 |

| | | | |
|---|---|---|---|
| H | 1.24282100 | -1.08797800 | -1.08619000 |
| C | 3.41438000 | 0.53922300 | -0.93831700 |
| H | 1.85663700 | 1.74336300 | -0.13033000 |
| H | 1.56871100 | 1.22664200 | -1.78277700 |
| C | 3.19939600 | -1.46786700 | 0.52664700 |
| H | 2.01466800 | -0.08318300 | 1.66640800 |
| H | 1.20969300 | -1.63484300 | 1.37852800 |
| C | 3.96213100 | -0.52422500 | -0.35674900 |
| H | 4.03138600 | 1.19330700 | -1.54535200 |
| H | 3.79337600 | -1.70585400 | 1.41284000 |
| H | 3.04514600 | -2.41794300 | 0.00220900 |
| H | 5.01536000 | -0.73119700 | -0.51676000 |

39

w-1-1 carbene-Au fragment

| | | | |
|---|---|---|---|
| Au | -1.35603800 | -1.29842900 | -0.06941300 |
| N | 0.65017700 | 0.94650200 | -0.07775500 |
| C | 0.92441800 | 2.37959100 | 0.25658800 |
| C | -0.61554000 | 0.62880100 | -0.19305800 |
| C | -0.48752600 | 2.87939600 | 0.59821800 |
| H | -0.65589000 | 3.88835100 | 0.21583500 |
| H | -0.60560200 | 2.90949500 | 1.68504500 |
| C | 1.49591500 | 3.13226200 | -0.94464700 |
| H | 2.51153600 | 2.82478500 | -1.18538600 |
| H | 1.51800800 | 4.19774200 | -0.70827800 |
| H | 0.87163300 | 2.99146700 | -1.82739200 |
| C | 1.84774800 | 2.51685600 | 1.45992100 |
| H | 1.46549400 | 1.94253500 | 2.30545400 |
| H | 1.88530100 | 3.56924800 | 1.74900400 |
| H | 2.86655300 | 2.19306400 | 1.24502500 |
| C | -1.46908800 | 1.85273200 | 0.01261600 |
| C | -2.02684400 | 2.25784000 | -1.35537100 |
| H | -2.63050600 | 1.44491500 | -1.76321500 |
| H | -2.65771200 | 3.14326800 | -1.24546100 |
| H | -1.23467000 | 2.48426900 | -2.07069500 |

| | | | |
|---|---|---|---|
| C | -2.63163500 | 1.57427000 | 0.95949800 |
| H | -3.18208800 | 2.50090600 | 1.14430100 |
| H | -3.31100300 | 0.83807000 | 0.52659800 |
| H | -2.27586800 | 1.18118100 | 1.91398500 |
| C | 1.66821800 | -0.11483000 | -0.19718500 |
| C | 2.94946600 | 0.25296700 | -0.92698200 |
| C | 1.99826900 | -0.75319100 | 1.14888800 |
| H | 1.16770100 | -0.88625400 | -0.79133200 |
| C | 3.85431100 | -0.94982600 | -0.95639300 |
| H | 3.46923300 | 1.08314500 | -0.43792300 |
| H | 2.72780100 | 0.57718900 | -1.94770400 |
| C | 2.62381200 | -2.11411500 | 0.87597800 |
| H | 2.70056000 | -0.12459900 | 1.70519900 |
| H | 1.08108600 | -0.86074900 | 1.73367600 |
| C | 3.69338100 | -2.01183300 | -0.17147100 |
| H | 4.66505000 | -0.93292700 | -1.67662200 |
| H | 3.04096600 | -2.53685600 | 1.79298200 |
| H | 1.84094700 | -2.80873500 | 0.54609700 |
| H | 4.36782200 | -2.85469800 | -0.28091400 |

46

e-1-0 carbene fragment

| | | | |
|---|---|---|---|
| C | 1.25154800 | 1.38575600 | 0.03263000 |
| N | -0.64301200 | -0.14319600 | 0.24727600 |
| C | -1.18767800 | -0.27292200 | 1.61889600 |
| C | 2.62639600 | 1.56260900 | -0.10618300 |
| H | 3.03305700 | 2.56478200 | -0.16326800 |
| C | -1.49101000 | -0.19945400 | -0.74979700 |
| C | -0.11613700 | 2.92209900 | -1.37262300 |
| H | -0.58230200 | 2.06582500 | -1.86287500 |
| H | 0.76152300 | 3.21924400 | -1.95392300 |
| H | -0.82092600 | 3.75731300 | -1.36750000 |
| C | 1.60849300 | -1.03998900 | 0.03984500 |
| C | 3.48102300 | 0.47255700 | -0.17084300 |
| H | 4.54737100 | 0.62888600 | -0.27742400 |

| | | | |
|---|---|---|---|
| C | 0.76843100 | 0.07627000 | 0.09378300 |
| C | 0.70355200 | -2.86207300 | -1.38487800 |
| H | 0.01074900 | -2.15757700 | -1.84888200 |
| H | 0.26074700 | -3.86075600 | -1.40072200 |
| H | 1.62019100 | -2.88503600 | -1.98097700 |
| C | 0.30843800 | 2.57002700 | 0.05432900 |
| H | -0.58907200 | 2.27270000 | 0.60492800 |
| C | 1.03621600 | -2.44185600 | 0.04849900 |
| H | 0.09978900 | -2.42248600 | 0.61603000 |
| C | -2.56400500 | -0.84604000 | 1.33544500 |
| H | -3.31904000 | -0.53114800 | 2.05766300 |
| H | -2.51728000 | -1.93913400 | 1.34189800 |
| C | 0.89739900 | 3.79403100 | 0.74872400 |
| H | 1.29535400 | 3.54993600 | 1.73534600 |
| H | 0.12895500 | 4.56024700 | 0.86465200 |
| H | 1.70268600 | 4.23431000 | 0.15635900 |
| C | 2.97599200 | -0.81748000 | -0.09660400 |
| H | 3.65360700 | -1.66074400 | -0.14757800 |
| C | 1.95795300 | -3.46686400 | 0.70067900 |
| H | 2.29096700 | -3.14412700 | 1.68883700 |
| H | 2.84254200 | -3.64905600 | 0.08619300 |
| H | 1.43783200 | -4.42053900 | 0.80488000 |
| C | -2.85533500 | -0.35503700 | -0.09584700 |
| C | -3.45560200 | 1.05459100 | -0.07819800 |
| C | -3.75306000 | -1.30055100 | -0.87602700 |
| H | -3.49029400 | 1.46646900 | -1.08846300 |
| H | -2.86063600 | 1.73209000 | 0.54186800 |
| H | -3.91630300 | -0.92011700 | -1.88548900 |
| H | -3.29652400 | -2.28984700 | -0.95760000 |
| H | -4.47145200 | 1.02440900 | 0.32437900 |
| H | -4.72244800 | -1.40809600 | -0.38097000 |
| H | -0.53925200 | -0.91374700 | 2.21909500 |
| H | -1.21444700 | 0.71452300 | 2.09162600 |

47

e-1-0 carbene-Au fragment

| | | | |
|---|---|---|---|
| Au | -1.47525500 | 0.30984300 | -1.17479000 |
| C | 1.66557300 | 1.46252900 | 0.37598600 |
| N | 0.33778700 | -0.47025900 | 1.05630300 |
| C | 0.40001800 | -0.78693900 | 2.49195700 |
| C | 2.80955300 | 1.95504600 | -0.24906100 |
| H | 2.97804800 | 3.02548500 | -0.27569000 |
| C | -0.82831300 | -0.68734900 | 0.49157600 |
| C | 0.23187400 | 3.50670600 | 0.06197800 |
| H | -0.20292300 | 3.06794000 | -0.83835800 |
| H | 1.06623500 | 4.15024300 | -0.22778300 |
| H | -0.52274200 | 4.13630300 | 0.53719400 |
| C | 2.39799600 | -0.81219900 | -0.18472400 |
| C | 3.72735100 | 1.09843800 | -0.83415900 |
| H | 4.60850800 | 1.50025900 | -1.31858800 |
| C | 1.48571100 | 0.07825500 | 0.39192500 |
| C | 1.36320900 | -2.67489700 | -1.46458800 |
| H | 0.43282600 | -2.10860500 | -1.54738500 |
| H | 1.13104100 | -3.74220000 | -1.47659800 |
| H | 1.97456800 | -2.44753600 | -2.34217400 |
| C | 0.68944400 | 2.41717700 | 1.02894200 |
| H | -0.19968200 | 1.85085100 | 1.32155200 |
| C | 2.13392300 | -2.30307700 | -0.19588600 |
| H | 1.50156400 | -2.54203700 | 0.66643700 |
| C | -0.80311700 | -1.70337900 | 2.65054500 |
| H | -1.24425200 | -1.66743900 | 3.64705600 |
| H | -0.50654800 | -2.73475100 | 2.43867800 |
| C | 1.31322200 | 3.03792700 | 2.27963300 |
| H | 1.63622500 | 2.27532500 | 2.99223200 |
| H | 0.60100100 | 3.69949600 | 2.77675400 |
| H | 2.19085200 | 3.63057200 | 2.00810200 |
| C | 3.52155700 | -0.27418400 | -0.80356000 |
| H | 4.24347400 | -0.93405600 | -1.26849300 |
| C | 3.40534900 | -3.13878700 | -0.08643200 |

| | | | |
|---|---|---|---|
| H | 4.02425700 | -2.82851600 | 0.75733100 |
| H | 4.00621700 | -3.06310900 | -0.99526700 |
| H | 3.14775200 | -4.19146500 | 0.04107700 |
| C | -1.76871600 | -1.21182700 | 1.55753700 |
| C | -2.60329100 | -0.03031500 | 2.06407700 |
| C | -2.67619600 | -2.30252800 | 1.01237600 |
| H | -3.18754900 | 0.39947200 | 1.24894800 |
| H | -1.96936200 | 0.75948800 | 2.47771700 |
| H | -3.29742900 | -1.90188500 | 0.20859300 |
| H | -2.09017700 | -3.13144400 | 0.60900400 |
| H | -3.28211400 | -0.37456700 | 2.84840600 |
| H | -3.32689300 | -2.68690700 | 1.80182400 |
| H | 1.35934400 | -1.25111900 | 2.72533200 |
| H | 0.31751900 | 0.14080900 | 3.06967600 |

58

e-1-2 carbene fragment

| | | | |
|---|---|---|---|
| C | 1.35352000 | 1.46025300 | -0.32936100 |
| N | -0.49580000 | -0.15846500 | -0.30932000 |
| C | -1.35714300 | -0.18706500 | 0.93740400 |
| C | 2.71007100 | 1.71019300 | -0.12894600 |
| H | 3.07592400 | 2.72840500 | -0.19443600 |
| C | -1.04578500 | -0.46272900 | -1.45833500 |
| C | 0.74908000 | 2.84921600 | -2.27958100 |
| H | 0.59137500 | 1.94462300 | -2.86946300 |
| H | 1.78719400 | 3.16948600 | -2.40506200 |
| H | 0.09893600 | 3.63824200 | -2.66437600 |
| C | 1.80139900 | -0.93132000 | -0.07983800 |
| C | 3.59934700 | 0.67624600 | 0.11667800 |
| H | 4.64910700 | 0.88883300 | 0.27626500 |
| C | 0.90858600 | 0.13756100 | -0.23221300 |
| C | 1.77517100 | -2.90074900 | -1.58521400 |
| H | 1.30737800 | -2.30189800 | -2.36856700 |
| H | 1.46869400 | -3.94235700 | -1.70649200 |
| H | 2.86065700 | -2.84727900 | -1.70726200 |

| | | | |
|---|---|---|---|
| C | 0.45277600 | 2.58162400 | -0.80228400 |
| H | -0.58077200 | 2.24200300 | -0.74202800 |
| C | 1.37280900 | -2.37924300 | -0.20505500 |
| H | 0.28284200 | -2.42694700 | -0.15270900 |
| C | -2.56675700 | -0.94257000 | 0.38019200 |
| H | -3.50870700 | -0.56451500 | 0.78316400 |
| H | -2.48733600 | -1.99729700 | 0.66623900 |
| C | 0.59611200 | 3.86077100 | 0.01630900 |
| H | 0.45550500 | 3.67900800 | 1.08389000 |
| H | -0.14031300 | 4.59874300 | -0.30746500 |
| H | 1.58354800 | 4.30777900 | -0.11991000 |
| C | 3.14919600 | -0.63549400 | 0.10464900 |
| H | 3.85771900 | -1.44617400 | 0.22934600 |
| C | 1.94977300 | -3.26083600 | 0.89999900 |
| H | 1.75626400 | -2.84296200 | 1.89125400 |
| H | 3.03074700 | -3.37299100 | 0.79074900 |
| H | 1.51357300 | -4.26046000 | 0.85286300 |
| C | -1.69428000 | 1.24454600 | 1.36889800 |
| H | -0.75749900 | 1.77890700 | 1.55896900 |
| H | -2.18245300 | 1.75474500 | 0.53553300 |
| C | -0.71987600 | -0.95647700 | 2.10454900 |
| H | -0.35899100 | -1.91523200 | 1.72640500 |
| H | -1.54680300 | -1.20732500 | 2.77808400 |
| C | -2.48548400 | -0.82275000 | -1.15389700 |
| C | -3.37863100 | 0.27985600 | -1.72524200 |
| C | -2.82090400 | -2.14464800 | -1.83762100 |
| H | -3.22245900 | 0.36421500 | -2.80186800 |
| H | -3.16313900 | 1.25340700 | -1.27985800 |
| H | -2.73245200 | -2.04509700 | -2.92035400 |
| H | -2.13552300 | -2.93252500 | -1.51249500 |
| H | -4.43020700 | 0.04355000 | -1.54101900 |
| H | -3.84118000 | -2.45352300 | -1.59170300 |
| C | -2.59434800 | 1.33080000 | 2.59580800 |
| H | -2.12206900 | 0.91380500 | 3.48571900 |

| | | | |
|---|---|---|---|
| H | -2.83964700 | 2.37127600 | 2.81079400 |
| H | -3.53403400 | 0.79764200 | 2.44093800 |
| C | 0.37320500 | -0.26279300 | 2.91436200 |
| H | 1.22285500 | 0.04568600 | 2.30464900 |
| H | -0.00305800 | 0.62357800 | 3.42757600 |
| H | 0.74789500 | -0.94445500 | 3.68030600 |

59

e-1-2 carbene-Au fragment

| | | | |
|---|---|---|---|
| Au | 2.22740800 | -0.55284500 | 0.15859300 |
| C | -1.32400400 | -1.54024000 | 0.94043800 |
| N | -0.52462900 | 0.59027300 | -0.02740600 |
| C | -1.34908300 | 1.83464300 | 0.17957000 |
| C | -1.97711000 | -2.75437400 | 0.73108200 |
| H | -2.17044900 | -3.39935200 | 1.58066500 |
| C | 0.75126200 | 0.79694300 | -0.26343300 |
| C | 0.17545600 | -2.22527200 | 2.82014500 |
| H | 1.03505500 | -2.25968300 | 2.14735400 |
| H | -0.26713600 | -3.22343100 | 2.87311600 |
| H | 0.52921600 | -1.96046300 | 3.81879000 |
| C | -1.41552000 | -1.13635800 | -1.47485100 |
| C | -2.36402300 | -3.15408100 | -0.53612000 |
| H | -2.87461700 | -4.09860600 | -0.67600800 |
| C | -1.09801300 | -0.72251700 | -0.17116700 |
| C | 0.24907900 | -0.97983100 | -3.31613700 |
| H | 1.06620800 | -0.99869500 | -2.59100600 |
| H | 0.57077400 | -0.41587800 | -4.19509900 |
| H | 0.04234500 | -2.00782600 | -3.62629400 |
| C | -0.85702200 | -1.20390800 | 2.34010100 |
| H | -0.36314300 | -0.23011500 | 2.31243300 |
| C | -1.00789400 | -0.35465400 | -2.70903500 |
| H | -0.75298600 | 0.66570000 | -2.41006700 |
| C | -0.40779800 | 2.87293700 | -0.44446400 |
| H | -0.47431000 | 3.84153500 | 0.05293800 |
| H | -0.69486700 | 3.01872600 | -1.49188500 |

| | | | |
|---|---|---|---|
| C | -2.02940800 | -1.15938500 | 3.32242200 |
| H | -2.85622800 | -0.54577400 | 2.95682900 |
| H | -1.70542000 | -0.76928300 | 4.28916700 |
| H | -2.42069200 | -2.16635500 | 3.48784100 |
| C | -2.06101200 | -2.35881900 | -1.63144300 |
| H | -2.31835300 | -2.69885200 | -2.62742000 |
| C | -2.12094000 | -0.27349200 | -3.75174800 |
| H | -3.06145100 | 0.07271500 | -3.31626900 |
| H | -2.30379100 | -1.24712100 | -4.21101100 |
| H | -1.83534700 | 0.41196800 | -4.55167600 |
| C | -1.56068600 | 2.05396300 | 1.68071500 |
| H | -2.10208500 | 1.19334700 | 2.07699000 |
| H | -0.58471200 | 2.05486100 | 2.17233300 |
| C | -2.69069300 | 1.79713800 | -0.56314600 |
| H | -2.50370000 | 1.47992000 | -1.59141300 |
| H | -3.01780300 | 2.84004400 | -0.63506300 |
| C | 1.01099600 | 2.27753700 | -0.38447000 |
| C | 1.84960000 | 2.79462600 | 0.78603400 |
| C | 1.78386500 | 2.51551400 | -1.68229900 |
| H | 2.81794500 | 2.29130200 | 0.79860300 |
| H | 1.37217200 | 2.61824800 | 1.75033700 |
| H | 2.76226700 | 2.03382000 | -1.62671800 |
| H | 1.24718000 | 2.09906000 | -2.53937600 |
| H | 2.01071200 | 3.86945000 | 0.66908000 |
| H | 1.92311500 | 3.58753400 | -1.84558200 |
| C | -2.31755500 | 3.32920300 | 2.02829800 |
| H | -3.32415600 | 3.33241500 | 1.60735400 |
| H | -2.41377700 | 3.42604300 | 3.10988800 |
| H | -1.80352500 | 4.22054400 | 1.66527900 |
| C | -3.81679100 | 0.96441600 | 0.04222200 |
| H | -3.55233800 | -0.08996300 | 0.13730000 |
| H | -4.10529700 | 1.32745200 | 1.03066500 |
| H | -4.69990800 | 1.02562500 | -0.59537800 |

67

e-1-3 carbene fragment

| | | | |
|---|---|---|---|
| C | -1.88979100 | 1.40635300 | 0.18869400 |
| N | -0.31532800 | -0.43292700 | 0.66084200 |
| C | -2.90539400 | 1.92379800 | -0.61103600 |
| H | -3.30015900 | 2.90912100 | -0.39466300 |
| C | -0.67729000 | -1.08330100 | 1.74265400 |
| C | -2.41479200 | 1.80335400 | 2.57470700 |
| H | -2.43560500 | 0.72599400 | 2.74452600 |
| H | -3.42490900 | 2.13735700 | 2.32022500 |
| H | -2.11116700 | 2.30589800 | 3.49638500 |
| C | -1.97512500 | -0.65923200 | -1.11341900 |
| C | -3.43603500 | 1.18915100 | -1.66126800 |
| H | -4.22119900 | 1.61046000 | -2.27663700 |
| C | -1.38710100 | 0.13602700 | -0.12405000 |
| C | -2.76888500 | -2.97780400 | -0.74477000 |
| H | -2.93655000 | -2.75050200 | 0.30975900 |
| H | -2.53482000 | -4.04030900 | -0.84436900 |
| H | -3.69422200 | -2.77953900 | -1.29242800 |
| C | -1.45558400 | 2.15319100 | 1.43370300 |
| H | -0.46153600 | 1.79846600 | 1.71571100 |
| C | -1.62860000 | -2.12227500 | -1.29888700 |
| H | -0.74437500 | -2.34974300 | -0.70070500 |
| C | 1.62063900 | -1.57078800 | 1.21504500 |
| H | 2.63058300 | -1.44612700 | 1.60248500 |
| H | 1.61130500 | -2.49813900 | 0.62861600 |
| C | -1.39460400 | 3.66772500 | 1.25271000 |
| H | -0.81554800 | 3.95693500 | 0.37352400 |
| H | -0.93858100 | 4.12723300 | 2.13147800 |
| H | -2.39635200 | 4.09203200 | 1.15337600 |
| C | -2.99187300 | -0.10323900 | -1.88712800 |
| H | -3.45357300 | -0.70236700 | -2.66388100 |
| C | -1.33499600 | -2.47877100 | -2.75379000 |
| H | -0.56693200 | -1.82943400 | -3.18066800 |
| H | -2.23223100 | -2.38448800 | -3.36966700 |

| | | | |
|---|---|---|---|
| H | -0.99505600 | -3.51372300 | -2.82909400 |
| C | 0.57482500 | -1.68530400 | 2.32871100 |
| C | 0.93139000 | -0.88941100 | 3.58591800 |
| C | 0.31139200 | -3.13828400 | 2.70806000 |
| H | 0.10909400 | -0.93756100 | 4.30204800 |
| H | 1.12012300 | 0.16289200 | 3.36434300 |
| H | -0.45280500 | -3.19490800 | 3.48459500 |
| H | -0.04353800 | -3.70410000 | 1.84197700 |
| H | 1.82665200 | -1.30867900 | 4.05326900 |
| H | 1.22691300 | -3.61037200 | 3.07676600 |
| C | 1.17107500 | -0.42697800 | 0.29338600 |
| C | 1.50153700 | -0.75439400 | -1.17853400 |
| C | 1.85103900 | 0.92866200 | 0.60016300 |
| H | 1.02015100 | -1.69812700 | -1.44762800 |
| C | 3.02511400 | -0.94025500 | -1.28545800 |
| C | 1.10772100 | 0.34161000 | -2.17479400 |
| H | 1.59698900 | 1.23322500 | 1.61905000 |
| C | 3.37526000 | 0.79662200 | 0.48082300 |
| C | 1.41961100 | 2.02010100 | -0.38798900 |
| H | 3.26689000 | -1.22688500 | -2.31464400 |
| H | 3.36427300 | -1.75256700 | -0.63749200 |
| C | 3.75000600 | 0.35957000 | -0.93487600 |
| H | 0.02868900 | 0.50190100 | -2.20091500 |
| H | 1.41033800 | 0.01641400 | -3.17750500 |
| C | 1.81681200 | 1.64457800 | -1.81436900 |
| H | 3.78025900 | 0.10302600 | 1.21976800 |
| H | 3.82192600 | 1.77513800 | 0.68959100 |
| H | 0.34247800 | 2.17826800 | -0.35750500 |
| H | 1.89750200 | 2.96219400 | -0.09622800 |
| H | 4.83061300 | 0.20279300 | -0.99682900 |
| C | 3.32680200 | 1.45287800 | -1.91211600 |
| H | 1.49689300 | 2.43528100 | -2.49846500 |
| H | 3.84345700 | 2.38851100 | -1.67172600 |
| H | 3.60239700 | 1.17340700 | -2.93488600 |

68
e-1-3 carbene-Au fragment

| | | | |
|---|---|---|---|
| Au | 2.70328300 | -0.56268500 | 0.01375400 |
| C | 0.12769500 | 1.85133100 | -1.27320400 |
| N | -0.29020500 | -0.23215700 | -0.02391900 |
| C | 0.42145200 | 3.21194000 | -1.25486900 |
| H | 0.51186600 | 3.74362100 | -2.19450100 |
| C | 0.71583100 | -1.06233900 | -0.19368200 |
| C | 1.44793500 | 0.88766400 | -3.15660500 |
| H | 2.04420500 | 0.28446000 | -2.46908800 |
| H | 1.96345300 | 1.84065200 | -3.30397300 |
| H | 1.39328700 | 0.37713100 | -4.12103300 |
| C | 0.30330300 | 1.82816400 | 1.17054000 |
| C | 0.61910200 | 3.88887700 | -0.06195900 |
| H | 0.83921400 | 4.94897200 | -0.06831900 |
| C | 0.01188200 | 1.18658800 | -0.04237300 |
| C | 1.83513600 | 1.23184500 | 3.05699100 |
| H | 2.57080400 | 0.85244100 | 2.34415000 |
| H | 1.92353200 | 0.66420500 | 3.98590500 |
| H | 2.07489600 | 2.27514900 | 3.27769600 |
| C | 0.04044700 | 1.14295800 | -2.61234900 |
| H | -0.44322500 | 0.17558500 | -2.45888100 |
| C | 0.41113500 | 1.11553200 | 2.50641000 |
| H | 0.20644600 | 0.05197800 | 2.35178800 |
| C | -1.16296700 | -2.31486700 | 0.51436900 |
| H | -1.85031500 | -3.09527600 | 0.19494800 |
| H | -1.06516800 | -2.39074900 | 1.60407600 |
| C | -0.75657500 | 1.92360500 | -3.65940500 |
| H | -1.72213300 | 2.26747400 | -3.28675000 |
| H | -0.92923400 | 1.29483600 | -4.53481800 |
| H | -0.19606100 | 2.79722000 | -3.99948800 |
| C | 0.57933800 | 3.19351000 | 1.13407400 |
| H | 0.79189600 | 3.71048000 | 2.06277200 |
| C | -0.56305900 | 1.67382900 | 3.54543700 |

| | | | |
|---|---|---|---|
| H | -1.59984700 | 1.65653800 | 3.21001700 |
| H | -0.30769800 | 2.71017200 | 3.77998600 |
| H | -0.48928200 | 1.10143100 | 4.47248700 |
| C | 0.22583800 | -2.47853000 | -0.10478300 |
| C | 0.21731100 | -3.05548400 | -1.52401300 |
| C | 1.12886900 | -3.33762300 | 0.77356200 |
| H | 1.23399100 | -3.05638800 | -1.92256400 |
| H | -0.41029600 | -2.47767600 | -2.20401800 |
| H | 2.11869400 | -3.43914000 | 0.32578900 |
| H | 1.25311900 | -2.88906600 | 1.76183600 |
| H | -0.15100200 | -4.08416500 | -1.50117200 |
| H | 0.68587800 | -4.33082600 | 0.89053800 |
| C | -1.65062700 | -0.89506700 | 0.18573400 |
| C | -2.48968300 | -0.33285100 | 1.34967600 |
| C | -2.54433100 | -0.82278200 | -1.07548200 |
| H | -1.87762000 | -0.34306800 | 2.25558900 |
| C | -3.69081200 | -1.27271700 | 1.55506300 |
| C | -3.04977800 | 1.07104300 | 1.09798800 |
| H | -1.97251000 | -1.15908700 | -1.94343000 |
| C | -3.77077700 | -1.72895300 | -0.90534900 |
| C | -3.06538900 | 0.59770100 | -1.32035600 |
| H | -4.26668500 | -0.91116300 | 2.41343000 |
| H | -3.35790400 | -2.28515800 | 1.79715900 |
| C | -4.58143100 | -1.28492000 | 0.31164300 |
| H | -2.25614200 | 1.81127000 | 0.98454300 |
| H | -3.64323800 | 1.36397700 | 1.97217700 |
| C | -3.92731900 | 1.06416300 | -0.14972400 |
| H | -3.49095700 | -2.78069800 | -0.82434100 |
| H | -4.38773700 | -1.63782500 | -1.80588800 |
| H | -2.24267600 | 1.30013500 | -1.44428800 |
| H | -3.64494600 | 0.60161000 | -2.25008200 |
| H | -5.41611100 | -1.97364200 | 0.46825800 |
| C | -5.11004000 | 0.12357900 | 0.05505200 |
| H | -4.28679000 | 2.07754500 | -0.34698800 |

| | | | |
|---|---|---|---|
| H | -5.75340900 | 0.12492600 | -0.83136300 |
| H | -5.71311400 | 0.46212400 | 0.90441300 |

59

e-1-4 carbene fragment

| | | | |
|---|---|---|---|
| C | -1.62665600 | 1.36314900 | -0.22611100 |
| N | 0.21187700 | -0.05090500 | 0.57706900 |
| C | -2.88933400 | 1.45847800 | -0.80803100 |
| H | -3.30150500 | 2.43884800 | -1.01787800 |
| C | 0.42414800 | 0.00503500 | 1.86763900 |
| C | -1.63534100 | 3.13401300 | 1.49985700 |
| H | -1.62024000 | 2.36883100 | 2.27785500 |
| H | -2.67635300 | 3.38492000 | 1.27782200 |
| H | -1.14102100 | 4.03148100 | 1.87888200 |
| C | -1.89057400 | -1.06605200 | -0.15677800 |
| C | -3.63624100 | 0.32593900 | -1.08918600 |
| H | -4.61390700 | 0.41886800 | -1.54557300 |
| C | -1.11726000 | 0.08547600 | 0.03866800 |
| C | -2.27498400 | -2.72452900 | 1.63909300 |
| H | -2.11131500 | -1.94749200 | 2.38788400 |
| H | -1.97838500 | -3.68763200 | 2.06066400 |
| H | -3.34347200 | -2.76602500 | 1.41051400 |
| C | -0.93189100 | 2.62572900 | 0.23985700 |
| H | 0.08800700 | 2.37210300 | 0.52679200 |
| C | -1.47229900 | -2.42238200 | 0.37228800 |
| H | -0.42363800 | -2.37052300 | 0.66735100 |
| C | 2.49230500 | -0.53868600 | 0.67885700 |
| H | 3.42734000 | -0.02247200 | 0.44834000 |
| H | 2.70294100 | -1.61335800 | 0.67272600 |
| C | -0.87828600 | 3.71541200 | -0.82628600 |
| H | -0.41947700 | 3.35756700 | -1.75021000 |
| H | -0.30012000 | 4.56730700 | -0.46290600 |
| H | -1.87843400 | 4.08223400 | -1.06789700 |
| C | -3.14798800 | -0.92282800 | -0.73814100 |
| H | -3.76162300 | -1.80269300 | -0.89471100 |

| | | | |
|---|---|---|---|
| C | -1.63509600 | -3.53968000 | -0.65366800 |
| H | -1.11043300 | -3.31246000 | -1.58394400 |
| H | -2.68804000 | -3.70431600 | -0.89326500 |
| H | -1.24223000 | -4.47744400 | -0.25556800 |
| C | 1.92219900 | -0.12546500 | 2.05196000 |
| C | 2.42886500 | 1.24766200 | 2.50088100 |
| C | 2.22734500 | -1.15684200 | 3.13153600 |
| H | 1.93430200 | 1.54756600 | 3.42607600 |
| H | 2.22945000 | 2.01531700 | 1.74880600 |
| H | 1.79005000 | -0.84962700 | 4.08264800 |
| H | 1.81048500 | -2.13168400 | 2.86529500 |
| H | 3.50801000 | 1.20959000 | 2.67342800 |
| H | 3.30803400 | -1.26862700 | 3.25889400 |
| C | 1.39701400 | -0.22686400 | -0.34469100 |
| C | 1.17219200 | -1.37184200 | -1.33204900 |
| C | 1.64674900 | 1.06208600 | -1.13373100 |
| C | 2.34163600 | -1.51369000 | -2.29994700 |
| H | 0.25416800 | -1.18087900 | -1.90184500 |
| H | 1.03113000 | -2.30415700 | -0.78097700 |
| C | 2.78312300 | 0.92575500 | -2.14163900 |
| H | 0.72263000 | 1.31780800 | -1.66738500 |
| H | 1.86314700 | 1.88081800 | -0.44341200 |
| C | 2.53601800 | -0.23234800 | -3.09617400 |
| H | 2.16795200 | -2.36398600 | -2.96274900 |
| H | 3.25709100 | -1.73206400 | -1.73619200 |
| H | 2.89725900 | 1.86491700 | -2.68738000 |
| H | 3.72712400 | 0.75515500 | -1.61156500 |
| H | 3.36278400 | -0.33357700 | -3.80241400 |
| H | 1.63127800 | -0.03313500 | -3.68282100 |

60

e-1-4 carbene-Au fragment

| | | | |
|---|---|---|---|
| Au | -2.37766400 | -0.48698200 | -0.09932600 |
| C | 0.42465100 | 1.62229000 | 1.52045800 |
| N | 0.61710400 | -0.23195500 | -0.07392100 |

| | | | |
|---|---|---|---|
| C | 0.36927600 | 2.99541500 | 1.74799000 |
| H | 0.33417000 | 3.35794700 | 2.76884700 |
| C | -0.39821400 | -1.05200000 | -0.05712200 |
| C | -1.10974600 | 0.79493000 | 3.28782400 |
| H | -1.86418900 | 0.58015100 | 2.52715100 |
| H | -1.28597400 | 1.80381600 | 3.67014700 |
| H | -1.23491300 | 0.09188600 | 4.11414800 |
| C | 0.33317400 | 2.06661300 | -0.88782100 |
| C | 0.32668200 | 3.89662900 | 0.69720800 |
| H | 0.28797500 | 4.96048800 | 0.89489400 |
| C | 0.46856000 | 1.18041600 | 0.19024300 |
| C | -1.30990000 | 1.96258900 | -2.75302100 |
| H | -2.04669800 | 1.51253800 | -2.08334200 |
| H | -1.49387100 | 1.60109200 | -3.76699100 |
| H | -1.45502400 | 3.04607100 | -2.74799500 |
| C | 0.30196900 | 0.68665800 | 2.70690500 |
| H | 0.42920100 | -0.33920100 | 2.35796300 |
| C | 0.11672800 | 1.61604600 | -2.31921100 |
| H | 0.21259700 | 0.52837600 | -2.36024000 |
| C | 1.57128400 | -2.29071900 | -0.66511600 |
| H | 2.20768800 | -3.03693600 | -0.18539300 |
| H | 1.67757900 | -2.41466300 | -1.74721300 |
| C | 1.33662500 | 0.96391500 | 3.79546400 |
| H | 2.35863400 | 0.92830900 | 3.41477200 |
| H | 1.24223200 | 0.22841400 | 4.59681300 |
| H | 1.17864400 | 1.94956300 | 4.23892600 |
| C | 0.28135200 | 3.42956900 | -0.60587400 |
| H | 0.17784400 | 4.13174900 | -1.42507800 |
| C | 1.11309300 | 2.24563300 | -3.29197400 |
| H | 2.14951000 | 2.07752600 | -2.99656800 |
| H | 0.95484400 | 3.32478900 | -3.35467400 |
| H | 0.96839700 | 1.83725800 | -4.29416700 |
| C | 0.09214500 | -2.45924300 | -0.26837400 |
| C | -0.08681100 | -3.19720200 | 1.06207300 |

| | | | |
|---|---|---|---|
| C | -0.71860000 | -3.16276200 | -1.35127100 |
| H | -1.13828800 | -3.18315000 | 1.35604500 |
| H | 0.49798900 | -2.73692000 | 1.86202500 |
| H | -1.76271700 | -3.25331400 | -1.04680700 |
| H | -0.68864500 | -2.60153100 | -2.28813400 |
| H | 0.23705400 | -4.23501300 | 0.95205600 |
| H | -0.30910900 | -4.16086800 | -1.52802000 |
| C | 1.97072600 | -0.86267800 | -0.27391500 |
| C | 2.75181600 | -0.15294900 | -1.37498400 |
| C | 2.76975300 | -0.78472100 | 1.02914500 |
| C | 4.14637100 | -0.74741900 | -1.53708800 |
| H | 2.84031000 | 0.90931200 | -1.11696700 |
| H | 2.19886500 | -0.22247700 | -2.31425200 |
| C | 4.18470800 | -1.33368800 | 0.87925000 |
| H | 2.82346900 | 0.26981900 | 1.32466800 |
| H | 2.24184800 | -1.32000400 | 1.82212600 |
| C | 4.93248800 | -0.62534900 | -0.24030700 |
| H | 4.66375600 | -0.24639600 | -2.35751800 |
| H | 4.06375500 | -1.80567500 | -1.81378000 |
| H | 4.71327900 | -1.23029300 | 1.82906200 |
| H | 4.14478200 | -2.40656200 | 0.65971000 |
| H | 5.93684400 | -1.03807100 | -0.35371800 |
| H | 5.04509000 | 0.43541000 | 0.01280700 |

60

e-1-7 carbene fragment

| | | | |
|---|---|---|---|
| C | -1.77052400 | 1.22524800 | 0.03198700 |
| N | 0.29866700 | 0.00425300 | 0.54197400 |
| C | -3.12047400 | 1.18979400 | -0.30458700 |
| H | -3.65833500 | 2.11924400 | -0.44640500 |
| C | 0.60767400 | 0.07077300 | 1.81768100 |
| C | -1.29168200 | 2.93295000 | 1.77041600 |
| H | -0.92170700 | 2.15068700 | 2.43505600 |
| H | -2.35909100 | 3.07584100 | 1.96226600 |
| H | -0.77579500 | 3.86955400 | 1.99639300 |

| | | | |
|---|---|---|---|
| C | -1.76992500 | -1.21893800 | 0.12140900 |
| C | -3.79070500 | -0.01747200 | -0.44485600 |
| H | -4.84066400 | -0.02638300 | -0.70993200 |
| C | -1.10179300 | 0.00594300 | 0.19619300 |
| C | -1.58660400 | -2.97324400 | 1.86366800 |
| H | -1.36114800 | -2.20915400 | 2.60998400 |
| H | -1.10532300 | -3.90873600 | 2.15801900 |
| H | -2.66802100 | -3.13482900 | 1.85091900 |
| C | -1.08113500 | 2.54646900 | 0.30456600 |
| H | -0.00843600 | 2.40432700 | 0.14793700 |
| C | -1.10278600 | -2.52915000 | 0.48269300 |
| H | -0.02656800 | -2.36132600 | 0.55814900 |
| C | 2.55484300 | -0.51194900 | 0.52386300 |
| H | 3.52646500 | -0.12832600 | 0.20974900 |
| H | 2.63155000 | -1.60465300 | 0.58425400 |
| C | -1.55069200 | 3.67827200 | -0.60509800 |
| H | -1.51961700 | 3.40024700 | -1.66075000 |
| H | -0.91795100 | 4.55584300 | -0.46144000 |
| H | -2.57335800 | 3.97755500 | -0.36443000 |
| C | -3.12278700 | -1.20864600 | -0.21291800 |
| H | -3.66060800 | -2.14777500 | -0.27608500 |
| C | -1.34850800 | -3.61702000 | -0.55948900 |
| H | -1.07949800 | -3.27965200 | -1.56342200 |
| H | -2.39922900 | -3.91492800 | -0.57745100 |
| H | -0.76169500 | -4.50716200 | -0.32385500 |
| C | 2.11697200 | 0.03687100 | 1.88463300 |
| C | 2.55795300 | 1.48900500 | 2.09204900 |
| C | 2.59673300 | -0.82096200 | 3.04564800 |
| H | 2.12034900 | 1.88797500 | 3.00918500 |
| H | 2.23805200 | 2.12556000 | 1.26315800 |
| H | 2.23636300 | -0.41243000 | 3.99103700 |
| H | 2.21975600 | -1.84264900 | 2.95340200 |
| H | 3.64715600 | 1.54337200 | 2.17039300 |
| H | 3.68970500 | -0.85820800 | 3.07203100 |

| | | | |
|---|---|---|---|
| C | 1.42643400 | -0.15630300 | -0.44561500 |
| C | 1.70174200 | 1.02583200 | -1.41064800 |
| C | 1.24549400 | -1.16887600 | -1.59818000 |
| C | 2.90028200 | 0.48383900 | -2.20608400 |
| C | 0.50925400 | 0.99690800 | -2.37356000 |
| H | 1.88489100 | 1.99224200 | -0.94115000 |
| C | 2.61926700 | -1.04155600 | -2.27061300 |
| C | 0.23128100 | -0.51725600 | -2.54844600 |
| H | 1.00705400 | -2.18697600 | -1.29256100 |
| H | 2.93659800 | 0.94155400 | -3.19595500 |
| H | 3.85192700 | 0.69549800 | -1.71806000 |
| H | -0.36243500 | 1.50369400 | -1.96560300 |
| H | 0.76108900 | 1.48979100 | -3.31415100 |
| H | 2.59425200 | -1.42434200 | -3.29168300 |
| H | 3.37905700 | -1.60396200 | -1.72531000 |
| H | -0.80033900 | -0.76032000 | -2.29292600 |
| H | 0.40300200 | -0.85413000 | -3.57286000 |

61

e-1-7 carbene-Au fragment

| | | | |
|---|---|---|---|
| Au | -2.39934700 | -0.39029000 | -0.06303900 |
| C | 0.41975300 | 1.68728300 | 1.42526600 |
| N | 0.59614900 | -0.29442500 | -0.00804400 |
| C | 0.23424400 | 3.06091300 | 1.54503700 |
| H | 0.21235200 | 3.50790900 | 2.53163200 |
| C | -0.46881200 | -1.05523200 | 0.14548400 |
| C | -0.86553800 | 0.58840700 | 3.26190000 |
| H | -1.52413600 | 0.10149500 | 2.54000500 |
| H | -1.32103300 | 1.54136100 | 3.54528600 |
| H | -0.79556300 | -0.03526200 | 4.15633300 |
| C | 0.21280200 | 1.91400000 | -1.00690800 |
| C | 0.06344600 | 3.86061900 | 0.42507700 |
| H | -0.07688300 | 4.92837000 | 0.53735100 |
| C | 0.44095700 | 1.13680300 | 0.13572900 |
| C | -1.31520600 | 1.58972400 | -2.96526800 |

| | | | |
|---|---|---|---|
| H | -2.08457400 | 1.17001900 | -2.31327200 |
| H | -1.40926600 | 1.13684600 | -3.95468300 |
| H | -1.49891100 | 2.66230600 | -3.06822400 |
| C | 0.52599200 | 0.83756900 | 2.67678200 |
| H | 0.95474600 | -0.12775100 | 2.39573500 |
| C | 0.08714500 | 1.34095000 | -2.40527400 |
| H | 0.23364200 | 0.25821000 | -2.35085100 |
| C | 1.29460800 | -2.40309500 | -0.68611700 |
| H | 1.95212400 | -3.22933600 | -0.41576700 |
| H | 1.14479300 | -2.43209500 | -1.77184900 |
| C | 1.41821500 | 1.45934700 | 3.75120300 |
| H | 2.39361800 | 1.75927000 | 3.36456300 |
| H | 1.57428800 | 0.74369500 | 4.56043900 |
| H | 0.94459200 | 2.34035400 | 4.18967500 |
| C | 0.04312800 | 3.28653700 | -0.83433600 |
| H | -0.12888900 | 3.90829000 | -1.70546500 |
| C | 1.11788600 | 1.94272200 | -3.36292000 |
| H | 2.14042600 | 1.85337300 | -2.99449300 |
| H | 0.91258700 | 3.00489400 | -3.51672000 |
| H | 1.05674800 | 1.45351800 | -4.33733600 |
| C | -0.06685700 | -2.49801600 | 0.00597200 |
| C | 0.03178100 | -3.05618400 | 1.43040800 |
| C | -1.06952700 | -3.31665100 | -0.79377300 |
| H | -0.93782700 | -2.97834200 | 1.92687100 |
| H | 0.76445700 | -2.51099300 | 2.02905100 |
| H | -2.03235100 | -3.35062000 | -0.28115000 |
| H | -1.23259200 | -2.87913500 | -1.78081200 |
| H | 0.32554000 | -4.10820000 | 1.39533400 |
| H | -0.69662800 | -4.33729800 | -0.91702900 |
| C | 1.86731600 | -1.03486600 | -0.31039200 |
| C | 2.95650400 | -1.03586100 | 0.79469700 |
| C | 2.81385600 | -0.43975800 | -1.37185600 |
| C | 4.05414500 | -1.87646000 | 0.12337700 |
| C | 3.46594400 | 0.40816400 | 0.79501300 |

| | | | |
|---|---|---|---|
| H | 2.64926400 | -1.40950600 | 1.77091500 |
| C | 3.90376500 | -1.51990400 | -1.37988100 |
| C | 3.42852900 | 0.79784300 | -0.70306300 |
| H | 2.36327400 | -0.27108100 | -2.34861100 |
| H | 5.03310100 | -1.60237300 | 0.51908300 |
| H | 3.92503100 | -2.94491400 | 0.29777600 |
| H | 2.82340400 | 1.06665600 | 1.37461000 |
| H | 4.46750500 | 0.46653300 | 1.22386800 |
| H | 4.82719000 | -1.14144800 | -1.81927700 |
| H | 3.59404200 | -2.38955900 | -1.96182100 |
| H | 2.83333300 | 1.69791100 | -0.85286300 |
| H | 4.42348100 | 0.99165500 | -1.10885700 |

67

e-1-8 carbene fragment

| | | | |
|---|---|---|---|
| C | 1.22405700 | 1.88353100 | -0.05484600 |
| N | -0.00008200 | 0.08285000 | 1.05907600 |
| C | 0.00020600 | -1.25360500 | 0.30266400 |
| C | 1.20044500 | 2.96931400 | -0.92779800 |
| H | 2.13456200 | 3.42825900 | -1.22850600 |
| C | -0.00042300 | 0.07108100 | 2.36197400 |
| C | 2.75830800 | 2.40556100 | 1.80307400 |
| H | 1.92939800 | 2.34141700 | 2.51039000 |
| H | 2.84655200 | 3.44060600 | 1.46026300 |
| H | 3.68344300 | 2.13575800 | 2.31806500 |
| C | -1.22456800 | 1.88342200 | -0.05474300 |
| C | -0.00036900 | 3.48359800 | -1.39461300 |
| H | -0.00043000 | 4.31891500 | -2.08409800 |
| C | -0.00022300 | 1.30300900 | 0.29785700 |
| C | -2.75918200 | 2.40574900 | 1.80279100 |
| H | -1.93036900 | 2.34203700 | 2.51025400 |
| H | -3.68433500 | 2.13592900 | 2.31773700 |
| H | -2.84760800 | 3.44064800 | 1.45958900 |
| C | 2.52119700 | 1.47694600 | 0.60933200 |
| H | 2.40875100 | 0.46536700 | 1.00040200 |

| | | | |
|---|---|---|---|
| C | -2.52168400 | 1.47675000 | 0.60942600 |
| H | -2.40903400 | 0.46533800 | 1.00088100 |
| C | 0.00035100 | -2.22873100 | 1.49564300 |
| H | 0.86317700 | -2.88860900 | 1.46071600 |
| H | -0.86248200 | -2.88860300 | 1.46083500 |
| C | 3.71638100 | 1.49414800 | -0.33549700 |
| H | 3.50492600 | 0.94446000 | -1.25379800 |
| H | 4.58160900 | 1.03511400 | 0.14715600 |
| H | 3.99690900 | 2.51702600 | -0.59961800 |
| C | -1.20109100 | 2.96923400 | -0.92768300 |
| H | -2.13527500 | 3.42811700 | -1.22828800 |
| C | -3.71674700 | 1.49336700 | -0.33557900 |
| H | -3.50503600 | 0.94346100 | -1.25368700 |
| H | -3.99747400 | 2.51610200 | -0.60004200 |
| H | -4.58193600 | 1.03427100 | 0.14708500 |
| C | 0.00034800 | -1.37532000 | 2.78942200 |
| C | 1.24888300 | -1.61504600 | 3.63765500 |
| C | -1.24694800 | -1.61684300 | 3.63887300 |
| H | 1.22532100 | -0.98680000 | 4.52892000 |
| H | 2.15740400 | -1.36723900 | 3.08146000 |
| H | -1.22357000 | -0.98831000 | 4.52994200 |
| H | -2.15640100 | -1.37071600 | 3.08345900 |
| H | 1.30455900 | -2.66349500 | 3.94353400 |
| H | -1.30064400 | -2.66529000 | 3.94511000 |
| C | -1.25759900 | -1.36626800 | -0.56676400 |
| C | -2.46179300 | -1.85186100 | -0.04762500 |
| C | -1.23858600 | -0.98021000 | -1.90945600 |
| C | -3.58987900 | -2.00875300 | -0.83717600 |
| H | -2.53557600 | -2.10918100 | 1.00107000 |
| C | -2.37024400 | -1.15507300 | -2.70623900 |
| C | -3.54061900 | -1.67958100 | -2.18612300 |
| H | -4.50118000 | -2.39567000 | -0.39918700 |
| H | -2.31586000 | -0.87559800 | -3.75273700 |
| H | -4.40774600 | -1.81829400 | -2.81929400 |

| | | | |
|---|---|---|---|
| C | 1.25786700 | -1.36602400 | -0.56699200 |
| C | 2.46221400 | -1.85161100 | -0.04818500 |
| C | 1.23855000 | -0.97981500 | -1.90964300 |
| C | 3.59020200 | -2.00813500 | -0.83794700 |
| H | 2.53621200 | -2.10924500 | 1.00041100 |
| C | 2.37012700 | -1.15427700 | -2.70662800 |
| C | 3.54070000 | -1.67862600 | -2.18680100 |
| H | 4.50162200 | -2.39503500 | -0.40018800 |
| H | 2.31550500 | -0.87465000 | -3.75307400 |
| H | 4.40775900 | -1.81702400 | -2.82013100 |
| C | -0.00015600 | -0.38939700 | -2.50827900 |
| H | -0.00020600 | -0.53472400 | -3.59066000 |
| H | -0.00033400 | 0.69650200 | -2.33807300 |

68

e-1-8 carbene-Au fragment

| | | | |
|---|---|---|---|
| Au | -2.99545400 | -0.00002800 | -0.08160200 |
| C | 0.21738700 | -1.22662900 | 1.78101200 |
| N | 0.02693900 | 0.00004200 | -0.33180300 |
| C | 1.27349500 | 0.00005300 | -1.21418000 |
| C | 0.51739800 | -1.19897900 | 3.14136300 |
| H | 0.57777000 | -2.13420100 | 3.68516100 |
| C | -1.11786300 | 0.00009800 | -0.94492500 |
| C | -1.55198400 | -2.95799500 | 1.64941200 |
| H | -2.30365200 | -2.19848300 | 1.42001100 |
| H | -1.52873800 | -3.10159900 | 2.73329300 |
| H | -1.85618900 | -3.90006700 | 1.18795100 |
| C | 0.21731900 | 1.22638500 | 1.78120100 |
| C | 0.70616700 | -0.00025900 | 3.81066500 |
| H | 0.94862400 | -0.00033300 | 4.86607200 |
| C | 0.15798100 | -0.00007000 | 1.10539400 |
| C | -1.55214700 | 2.95764800 | 1.64975100 |
| H | -2.30374600 | 2.19808900 | 1.42027800 |
| H | -1.85642300 | 3.89973500 | 1.18836800 |
| H | -1.52892400 | 3.10115800 | 2.73364600 |

| | | | |
|---|---|---|---|
| C | -0.16490600 | -2.54825200 | 1.14793200 |
| H | -0.22384900 | -2.41588000 | 0.06494600 |
| C | -0.16503400 | 2.54806100 | 1.14827200 |
| H | -0.22389100 | 2.41581600 | 0.06526800 |
| C | 0.62331700 | 0.00004600 | -2.61787200 |
| H | 0.94477300 | -0.86401100 | -3.19236500 |
| H | 0.94483700 | 0.86406600 | -3.19238300 |
| C | 0.83967700 | -3.65547400 | 1.45143100 |
| H | 1.86223900 | -3.34057600 | 1.24581800 |
| H | 0.62139000 | -4.53828200 | 0.84721100 |
| H | 0.77695200 | -3.95580200 | 2.50036800 |
| C | 0.51730100 | 1.19854900 | 3.14155500 |
| H | 0.57755700 | 2.13368600 | 3.68551200 |
| C | 0.83948400 | 3.65529500 | 1.45193000 |
| H | 1.86205000 | 3.34047700 | 1.24623000 |
| H | 0.77679700 | 3.95546900 | 2.50091300 |
| H | 0.62112500 | 4.53818500 | 0.84785200 |
| C | -0.91031500 | 0.00010900 | -2.42980800 |
| C | -1.57738400 | -1.24692900 | -3.01230100 |
| C | -1.57731900 | 1.24716000 | -3.01231700 |
| H | -2.65372000 | -1.21169600 | -2.83452400 |
| H | -1.19435300 | -2.15889900 | -2.54709100 |
| H | -2.65366000 | 1.21195200 | -2.83455000 |
| H | -1.19428500 | 2.15912700 | -2.54710300 |
| H | -1.39183500 | -1.29704100 | -4.08804200 |
| H | -1.39175800 | 1.29726100 | -4.08805400 |
| C | 2.10906400 | 1.26033600 | -0.95678900 |
| C | 1.83013600 | 2.46139500 | -1.61523600 |
| C | 3.19650500 | 1.23447800 | -0.08035400 |
| C | 2.64222700 | 3.57575900 | -1.47687100 |
| H | 0.96060800 | 2.54133700 | -2.25488200 |
| C | 4.02325600 | 2.35083300 | 0.04238400 |
| C | 3.76656200 | 3.51401600 | -0.66316000 |
| H | 2.40039700 | 4.48521700 | -2.01168700 |

| | | | |
|---|---|---|---|
| H | 4.88190500 | 2.29108500 | 0.70181500 |
| H | 4.42398800 | 4.36857700 | -0.56700300 |
| C | 2.10915900 | -1.26015800 | -0.95683800 |
| C | 1.83035400 | -2.46121000 | -1.61534500 |
| C | 3.19660400 | -1.23422900 | -0.08040600 |
| C | 2.64252900 | -3.57551600 | -1.47699500 |
| H | 0.96084500 | -2.54120400 | -2.25500900 |
| C | 4.02344600 | -2.35052100 | 0.04230300 |
| C | 3.76684000 | -3.51371200 | -0.66326100 |
| H | 2.40077200 | -4.48498000 | -2.01183500 |
| H | 4.88209200 | -2.29071600 | 0.70173300 |
| H | 4.42433300 | -4.36822300 | -0.56712700 |
| C | 3.48919900 | 0.00011800 | 0.71403400 |
| H | 4.52930900 | 0.00014700 | 1.04525700 |
| H | 2.86823800 | 0.00006800 | 1.62127700 |

66

e-1-15 carbene fragment

| | | | |
|---|---|---|---|
| C | -0.12200500 | 2.37531500 | -0.72359200 |
| N | 0.07073300 | 0.44395400 | 0.80483900 |
| C | 0.42784400 | -1.02125400 | 0.49013500 |
| C | -0.92519100 | 3.11441800 | -1.59232100 |
| H | -0.48984000 | 3.94868100 | -2.12984600 |
| C | 0.42922900 | 0.89575400 | 1.98388000 |
| C | 1.20184000 | 4.03989700 | 0.53894000 |
| H | 0.70307800 | 3.73907100 | 1.46063200 |
| H | 0.64092700 | 4.86026100 | 0.08193700 |
| H | 2.20318800 | 4.40396200 | 0.78080800 |
| C | -2.09389200 | 1.06313100 | -0.10818700 |
| C | -2.26991100 | 2.82222600 | -1.75451400 |
| H | -2.87187300 | 3.40288400 | -2.44248600 |
| C | -0.71250800 | 1.29496800 | -0.05528300 |
| C | -3.20960500 | 0.98770800 | 2.08734200 |
| H | -2.35329800 | 1.50149500 | 2.53150900 |
| H | -3.67838100 | 0.35848200 | 2.84778700 |

| | | | |
|---|---|---|---|
| H | -3.93523800 | 1.74227400 | 1.77125000 |
| C | 1.28545900 | 2.85833700 | -0.42976100 |
| H | 1.83167000 | 2.05989800 | 0.07728900 |
| C | -2.77266700 | 0.14436000 | 0.88682000 |
| H | -2.04278500 | -0.57960900 | 1.24990700 |
| C | 0.57747800 | -1.49136000 | 1.93529200 |
| H | 1.20413100 | -2.37312100 | 2.05760200 |
| H | -0.41758000 | -1.73112200 | 2.32350000 |
| C | 2.05974400 | 3.26222300 | -1.68329000 |
| H | 2.02020500 | 2.49633800 | -2.46133900 |
| H | 3.10654800 | 3.44268500 | -1.43205800 |
| H | 1.66448000 | 4.18753400 | -2.10879500 |
| C | -2.85558300 | 1.82518100 | -0.98798400 |
| H | -3.92269300 | 1.65141300 | -1.05558900 |
| C | -3.94468500 | -0.63697000 | 0.30756200 |
| H | -3.65366100 | -1.17675600 | -0.59529900 |
| H | -4.78252000 | 0.02124700 | 0.06487300 |
| H | -4.30498500 | -1.36231100 | 1.03984100 |
| C | 1.08002100 | -0.26280000 | 2.70370900 |
| C | 2.59616300 | -0.05383300 | 2.63671900 |
| C | 0.63288700 | -0.29964800 | 4.15923900 |
| H | 2.86436800 | 0.83931100 | 3.20361400 |
| H | 2.95392600 | 0.07468000 | 1.61380900 |
| H | 0.95496900 | 0.60526600 | 4.67602900 |
| H | -0.45673200 | -0.35893800 | 4.22751600 |
| H | 3.11248900 | -0.91265100 | 3.07471200 |
| H | 1.05942600 | -1.16782400 | 4.67037000 |
| C | -0.60474300 | -1.78742400 | -0.33701700 |
| C | -1.16531900 | -2.97421700 | 0.13120000 |
| C | -0.91724100 | -1.38338800 | -1.63812700 |
| C | -2.02301000 | -3.72699400 | -0.66487500 |
| H | -0.92515900 | -3.33561900 | 1.12276200 |
| C | -1.77707900 | -2.12789200 | -2.43174400 |
| H | -0.48085700 | -0.47898700 | -2.04339300 |

| | | | |
|---|---|---|---|
| C | -2.33429900 | -3.30702600 | -1.94923100 |
| H | -2.43980800 | -4.64786700 | -0.27653200 |
| H | -2.00582600 | -1.78773700 | -3.43384700 |
| H | -2.99925900 | -3.89270200 | -2.57116800 |
| C | 1.73632000 | -1.13052500 | -0.30086800 |
| C | 2.57982700 | -2.22811200 | -0.12819100 |
| C | 2.07460100 | -0.19740700 | -1.27586900 |
| C | 3.75096000 | -2.35303500 | -0.86332700 |
| H | 2.32571400 | -3.00771900 | 0.57800300 |
| C | 3.24778200 | -0.31368900 | -2.01045700 |
| H | 1.40534100 | 0.62545600 | -1.47774300 |
| C | 4.10048300 | -1.38808500 | -1.79910000 |
| H | 4.39139900 | -3.21132800 | -0.70275500 |
| H | 3.48854200 | 0.43797800 | -2.75287400 |
| H | 5.01757600 | -1.48146700 | -2.36649900 |

67

e-1-15 carbene-Au fragment

| | | | |
|---|---|---|---|
| Au | -2.80624900 | -0.13949100 | -0.60241600 |
| C | -0.43248100 | -0.14437500 | 2.26638100 |
| N | 0.14356900 | -0.14762000 | -0.14009900 |
| C | 1.50052300 | -0.23793600 | -0.84116300 |
| C | -0.61998700 | 0.59436000 | 3.43366000 |
| H | -0.84427100 | 0.06829700 | 4.35437300 |
| C | -0.84963300 | -0.69280100 | -0.82251700 |
| C | -2.17082300 | -1.91752500 | 2.50834700 |
| H | -2.75302200 | -1.49993900 | 1.68438000 |
| H | -2.52738500 | -1.47081900 | 3.44028400 |
| H | -2.35137600 | -2.99377900 | 2.55589500 |
| C | -0.17711700 | 1.96493800 | 1.04298700 |
| C | -0.55375900 | 1.97742900 | 3.43358500 |
| H | -0.69503700 | 2.52888300 | 4.35466900 |
| C | -0.12976600 | 0.56099800 | 1.08918000 |
| C | -1.58427500 | 3.30620100 | -0.50940800 |
| H | -2.32685800 | 2.50650300 | -0.55879900 |

| | | | |
|---|---|---|---|
| H | -1.60299900 | 3.86072000 | -1.45046600 |
| H | -1.87209400 | 3.99095800 | 0.29312500 |
| C | -0.67584000 | -1.64264400 | 2.33300100 |
| H | -0.36240300 | -2.09490500 | 1.38867000 |
| C | -0.17612300 | 2.76227300 | -0.24863900 |
| H | 0.08657700 | 2.09532000 | -1.07418800 |
| C | 0.98693600 | -0.43237100 | -2.26612200 |
| H | 1.70773100 | -0.88688500 | -2.94263200 |
| H | 0.70899700 | 0.54593100 | -2.67083900 |
| C | 0.08161000 | -2.31284000 | 3.48412900 |
| H | 1.12361700 | -1.99468100 | 3.55225800 |
| H | 0.05861900 | -3.39774700 | 3.36447800 |
| H | -0.39308200 | -2.08147400 | 4.44041000 |
| C | -0.36541700 | 2.65211600 | 2.23886100 |
| H | -0.39298200 | 3.73534600 | 2.22216000 |
| C | 0.80553200 | 3.93184900 | -0.23492600 |
| H | 1.80918800 | 3.62898500 | 0.05681800 |
| H | 0.46674500 | 4.70680900 | 0.45738800 |
| H | 0.85520500 | 4.38471200 | -1.22731500 |
| C | -0.30624000 | -1.24128200 | -2.11786200 |
| C | -0.10505800 | -2.75177400 | -1.93444900 |
| C | -1.23522900 | -1.00615500 | -3.30119500 |
| H | -1.08211900 | -3.23503400 | -1.87847600 |
| H | 0.44863400 | -2.99858600 | -1.02817800 |
| H | -2.18019700 | -1.53225100 | -3.15764500 |
| H | -1.45898200 | 0.05584600 | -3.42122500 |
| H | 0.43860600 | -3.15969100 | -2.79072900 |
| H | -0.76182200 | -1.37478000 | -4.21588600 |
| C | 2.42651100 | 0.95895300 | -0.64030000 |
| C | 2.93265900 | 1.66520100 | -1.72814700 |
| C | 2.92209900 | 1.26003500 | 0.63181800 |
| C | 3.90696300 | 2.64397100 | -1.55494300 |
| H | 2.58809500 | 1.44546600 | -2.73039900 |
| C | 3.88918700 | 2.23746300 | 0.80757500 |

| | | | |
|---|---|---|---|
| H | 2.55942000 | 0.71678900 | 1.49563000 |
| C | 4.39240100 | 2.93105600 | -0.28841800 |
| H | 4.28962600 | 3.17390600 | -2.41807900 |
| H | 4.25571000 | 2.45278300 | 1.80318400 |
| H | 5.15509200 | 3.68725300 | -0.15330200 |
| C | 2.31838100 | -1.44547800 | -0.36494700 |
| C | 3.21269800 | -2.08168000 | -1.22522000 |
| C | 2.26312600 | -1.87548900 | 0.95461500 |
| C | 3.97524100 | -3.15727400 | -0.79004600 |
| H | 3.33491700 | -1.73740000 | -2.24403400 |
| C | 3.01792700 | -2.95466200 | 1.39523600 |
| H | 1.63183100 | -1.34690900 | 1.65093300 |
| C | 3.87003000 | -3.61166100 | 0.51850700 |
| H | 4.65582100 | -3.64083800 | -1.47955000 |
| H | 2.94021100 | -3.27482400 | 2.42747500 |
| H | 4.45823900 | -4.45618700 | 0.85366500 |

## References


1. Romanov, A.S.; Becker, C.R.; James, C.E.; Di, D.; Credgington, D.; Linnolahti, M.; Bochmann, M. Copper and Gold Cyclic (Alkyl)(amino)carbene Complexes with Sub-Microsecond Photoemissions: Structure and Substituent Effects on Redox and Luminescent Properties. *Chem. Eur. J.*, **2017**, *23*, 4625–4637.
2. Gritzner, G.; Kůta, J. Recommendations on reporting electrode potentials in nonaqueous solvents: IUPC commission on electrochemistry. *Electrochim. Acta.*, **1984**, *29*, 869–873.
3. Cardona, C.M.; Li, W.; Kaifer, A.E.; Stockdale, D.; Bazan, G.C. Electrochemical Considerations for Determining Absolute Frontier Orbital Energy Levels of Conjugated Polymers for Solar Cell Applications. *Adv. Mater.*, **2011**, *23*, 2367-2371.
